# Supplementary material for: HOXB8 Counteracts MAPK/ERK Oncogenic Signaling in a Chicken Embryo Model of Neoplasia
Source: Int J Mol Sci. 2021 Aug 18;22(16):8911. doi: 10.3390/ijms22168911 (PMC8396257; doi:10.3390/ijms22168911)
Supplement: Supplementary file 1 [file ijms-22-08911-s001.zip › ijms-1277141 SUPPLEMENTARY FIGURES.pdf]

Supplementary Materials

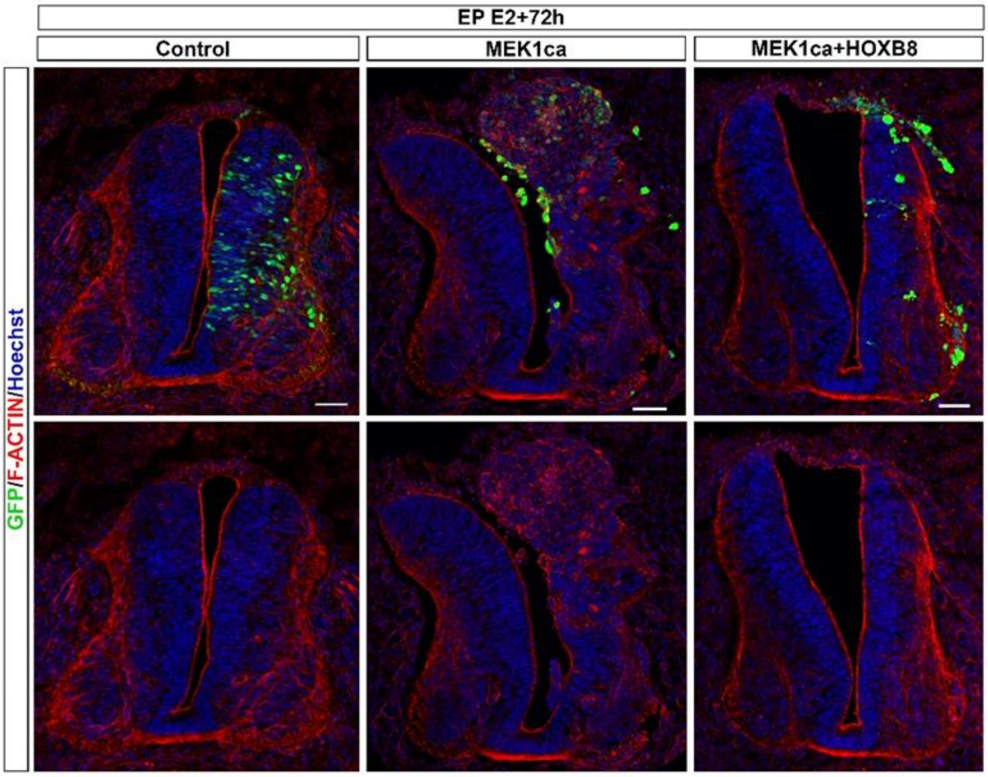

Figure S1. HOXB8 gain of function in the trunk neural tube of the chicken embryo transfected by MEK1ca expressing vector inhibits neoplasia formation. Immunofluorescences on transverse sections of chicken embryo with anti-GFP antibody with a co-staining against F-actin (phalloidin), 3 days day post-electroporation with PCIG, MEK1ca or MEK1ca+HOXB8 expressing vectors. Blue is Hoechst staining. Scale bar: 50µm.

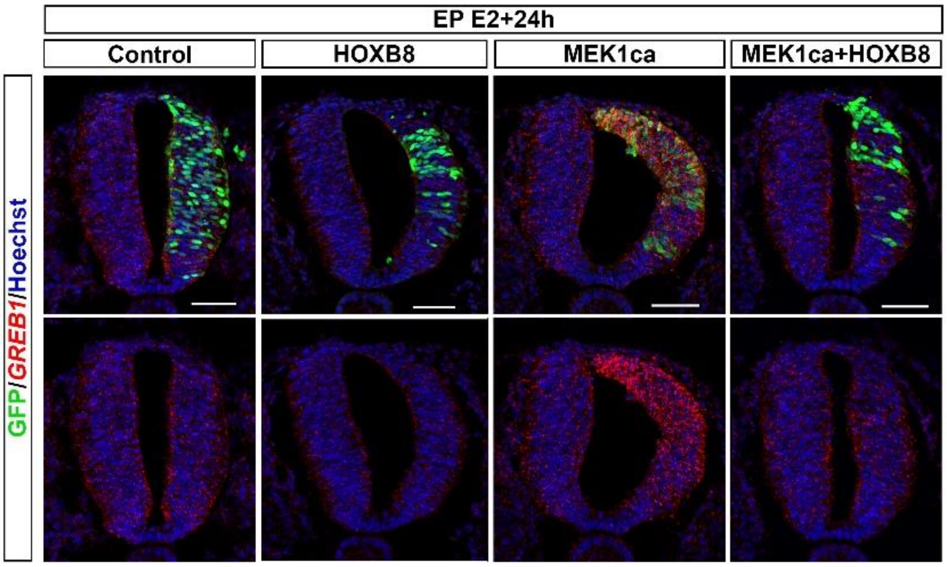

Figure S2: GREB1 is upregulated by MEK1ca in the chicken neural tube and reversed by HOXB8 co-expression. Fluorescent in situ hybridizations with GREB1 probe and immunofluorescences with anti-GFP antibody on trunk transverse sections of chicken embryo one day post-electroporation in the pCIG, HOXB8, MEK1ca and MEK1ca+HOXB8 conditions. Blue is Hoechst staining. Scale bar: 50µm.

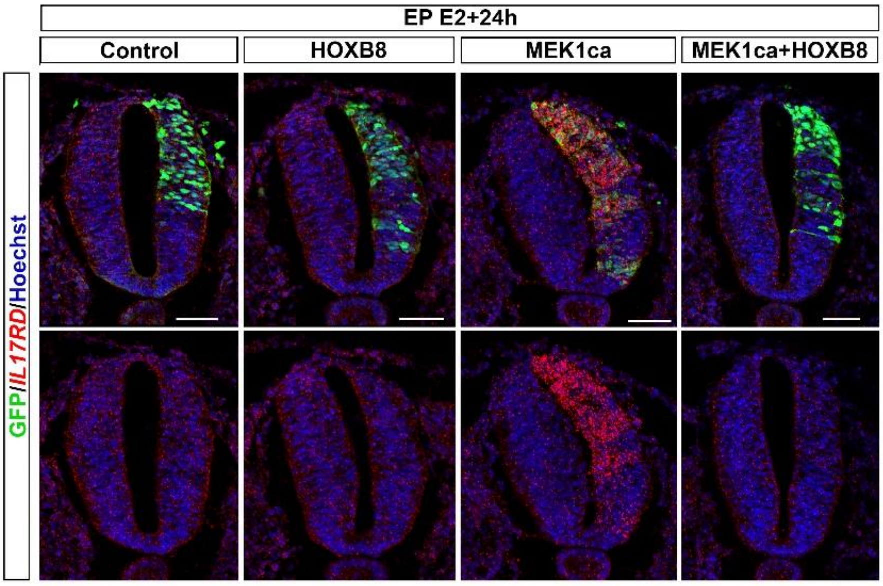

Figure S3: IL17RD is upregulated by MEK1ca in the chicken neural tube and reversed by HOXB8 co-expression. Fluorescent *in situ* hybridization with IL17RD probe and immunofluorescences with anti-

GFP antibody on trunk transverse sections of chicken embryo one day post-electroporation in the pCIG, HOXB8, MEK1ca and MEK1ca+HOXB8 conditions. Blue is Hoechst staining. Scale bar: 50µm.

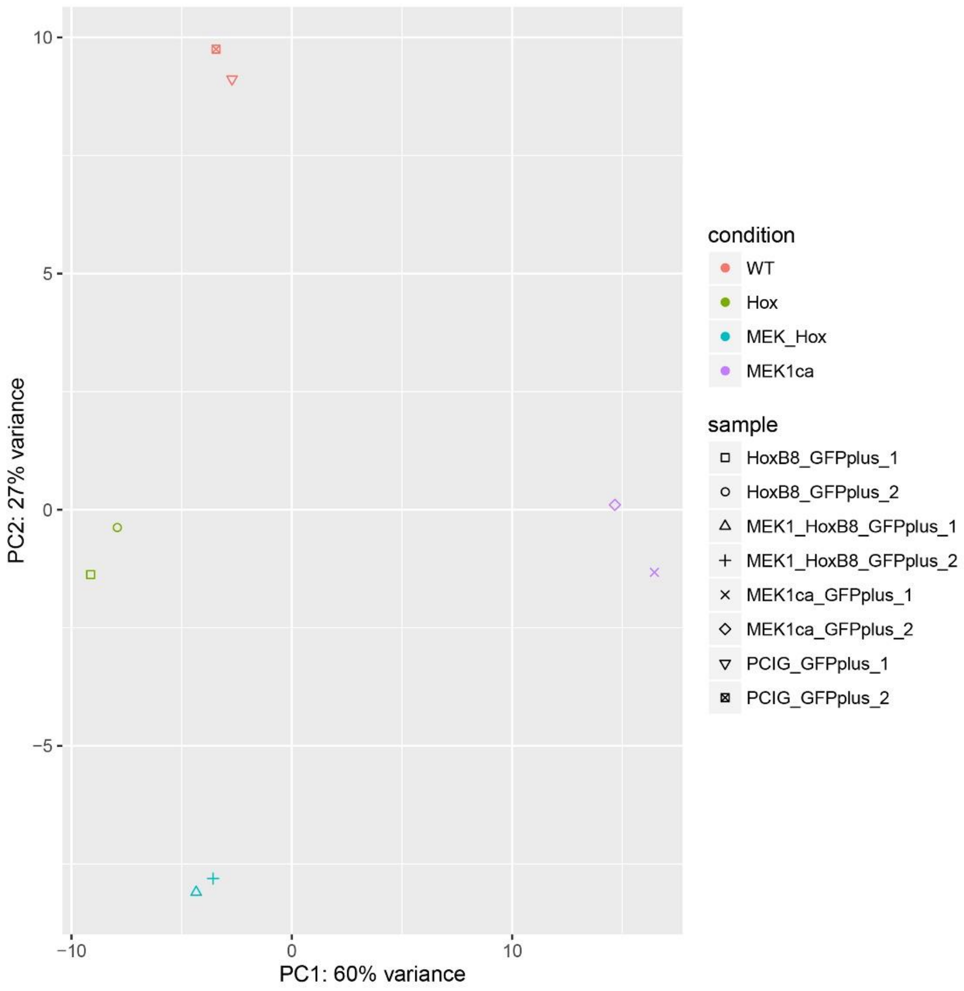

Figure S4: Principal component analysis (PCA) obtained from RNA-seq counting data using galgal4 assembly for the two replicates of the control (pCIG1, pCIG2), HOXB8 (HOXB8-1 and HOXB8-2), MEK1ca (MEK1ca-1 and MEK1ca-2) and MEK1ca+HOXB8 (MEK1ca+HOXB8-1 and MEK1ca+HOXB8-2) expressing samples, highlighting the quality of the biological replicates.

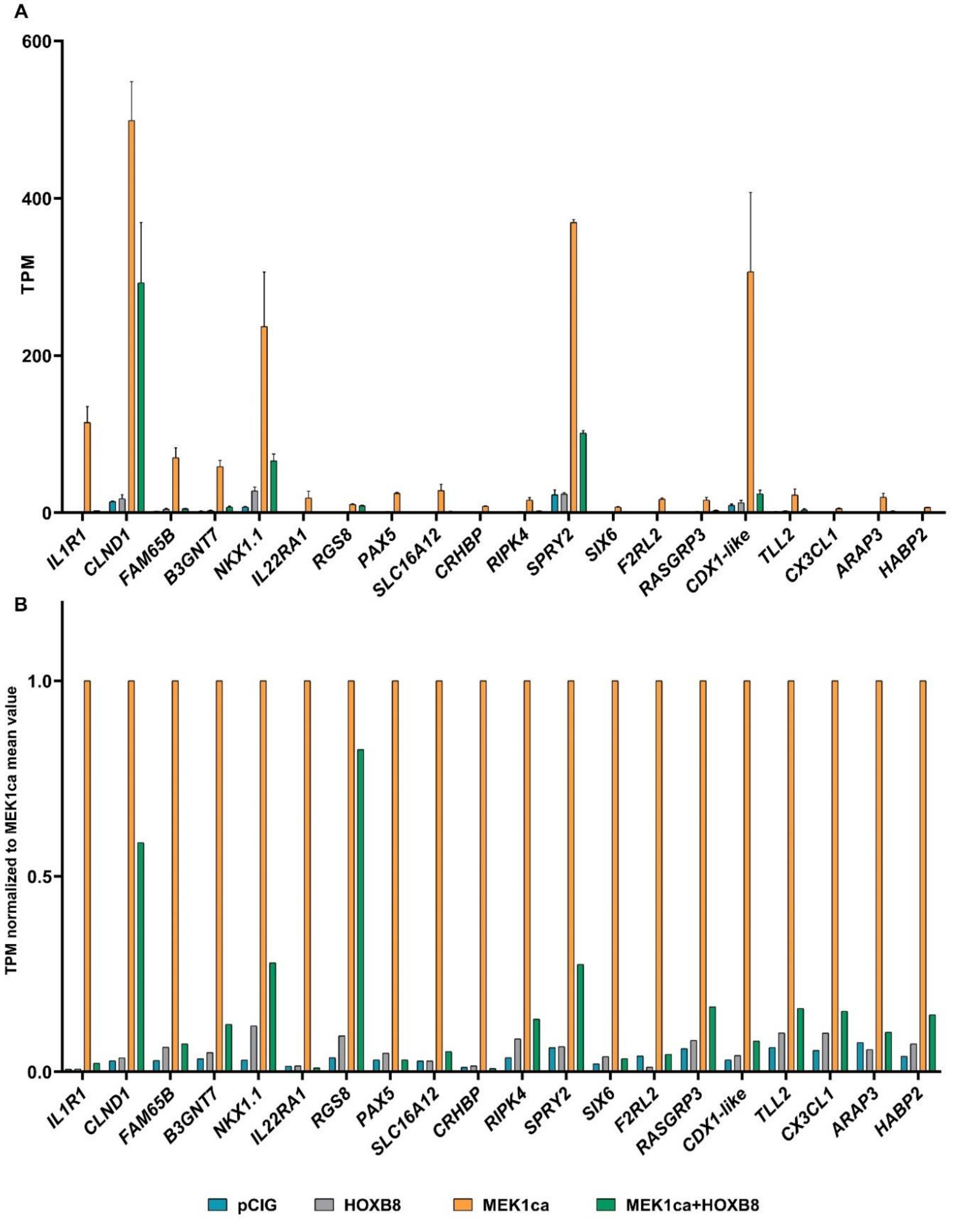

Figure S5: A and B- Graph of the mean of the TPM for the 20 genes most upregulated by MEK1ca, for the four RNAseq conditions. The mean of the TPM (transcripts per kilobase million) number for these 20 genes, obtained for the two replicates of the control (pCIG1, pCIG2), HOXB8 (HOXB8-1 and HOXB8-2), MEK1ca (MEK1ca-1 and MEK1ca-2) and MEK1ca+HOXB8 (MEK1ca+HOXB8-1 and MEK1ca+HOXB8-2) expressing samples (**A**) and normalized to MEK1ca value (**B**).

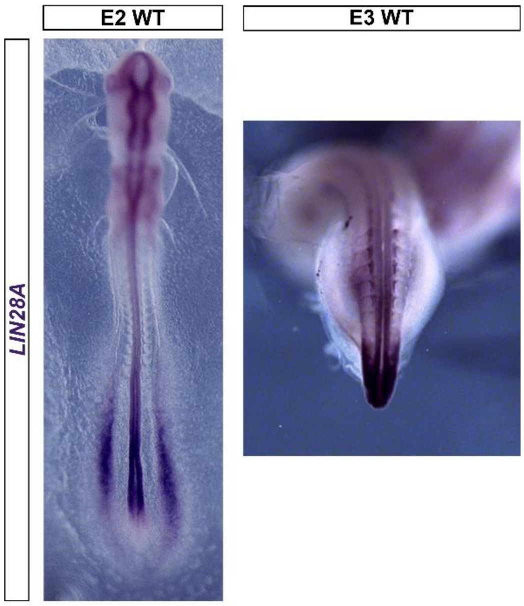

Figure S6: Expression pattern of *LIN28A* in the chicken embryo**.** Dorsal view of a two-days and three-days (only the most caudal part) old wild type (WT) chicken embryo after a whole mount *in situ* hybridization with LIN28A probe highlighting that its expression is the strongest in the most caudal part of the embryo.

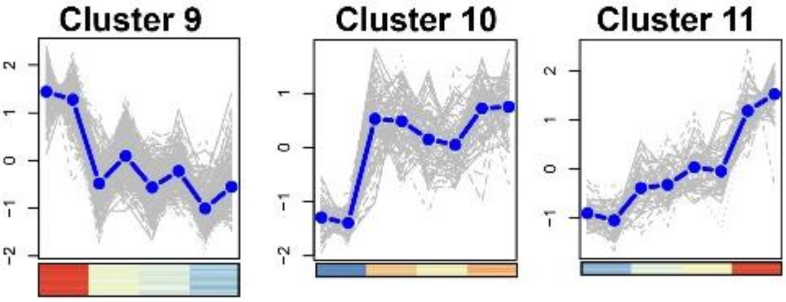

Figure S7: Plots of clusters 9, 10 and 11 (related to Figure 3), with the two replicates of each condition (control (pCIG1, pCIG2), HOXB8 (HOXB8-1 and HOXB8-2), MEK1ca (MEK1ca-1 and MEK1ca-2) and MEK1ca+HOXB8

(MEK1ca+HOXB8-1 and MEK1ca+HOXB8-2)), with the mean (in blue), and with the corresponding heat map on the bottom.

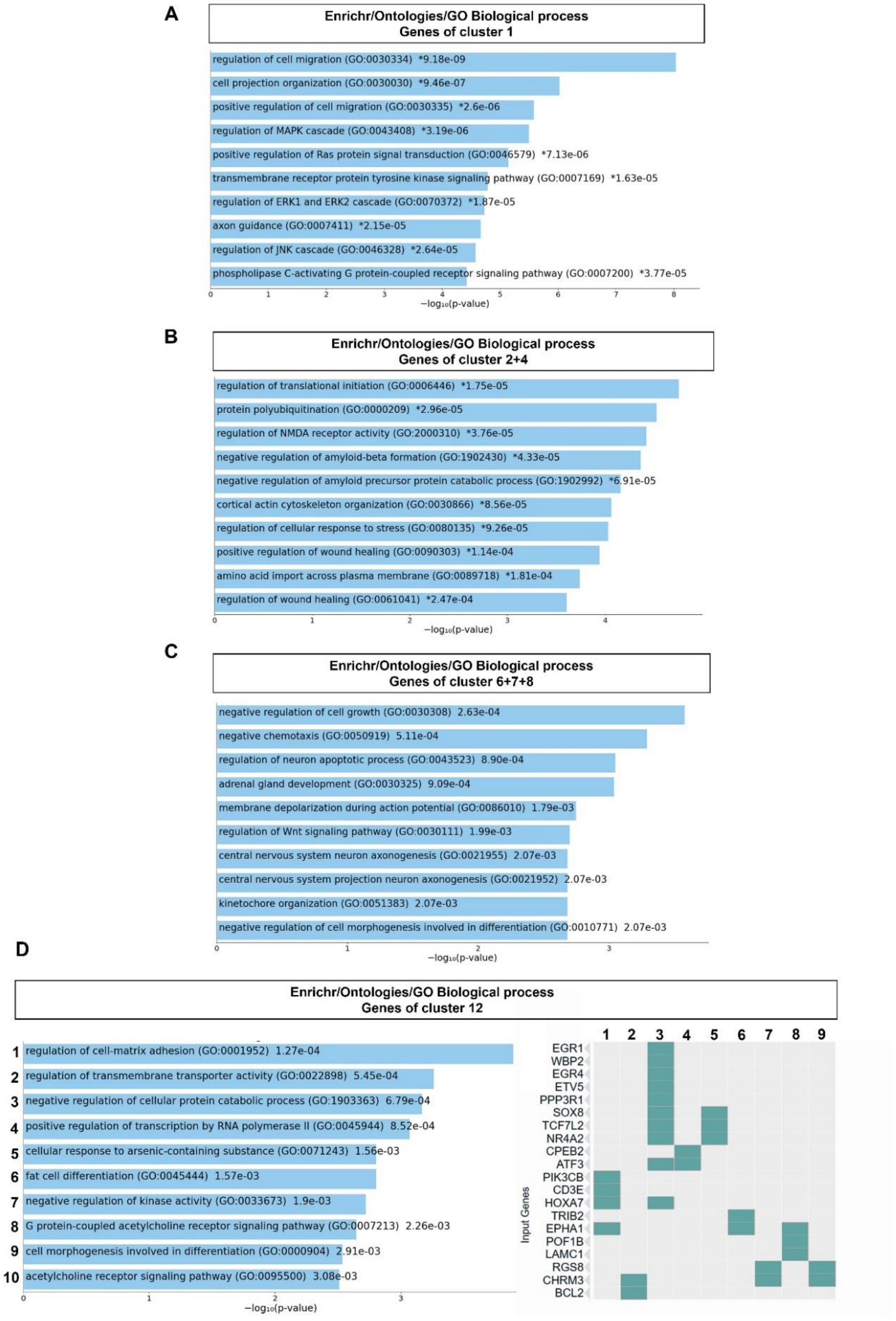

Figure S8: Gene ontology enrichment analysis of the genes in the clusters related to Figure 3, using the EnrichR analytical tool for biological processes (GO biological processes 2021), **A-** genes of cluster 1, **B-** genes of cluster 2 and 4, **C-** genes of cluster 6,7 and 8 and **D-** genes of cluster 12 with the clustergram with genes associated to each biological processes.

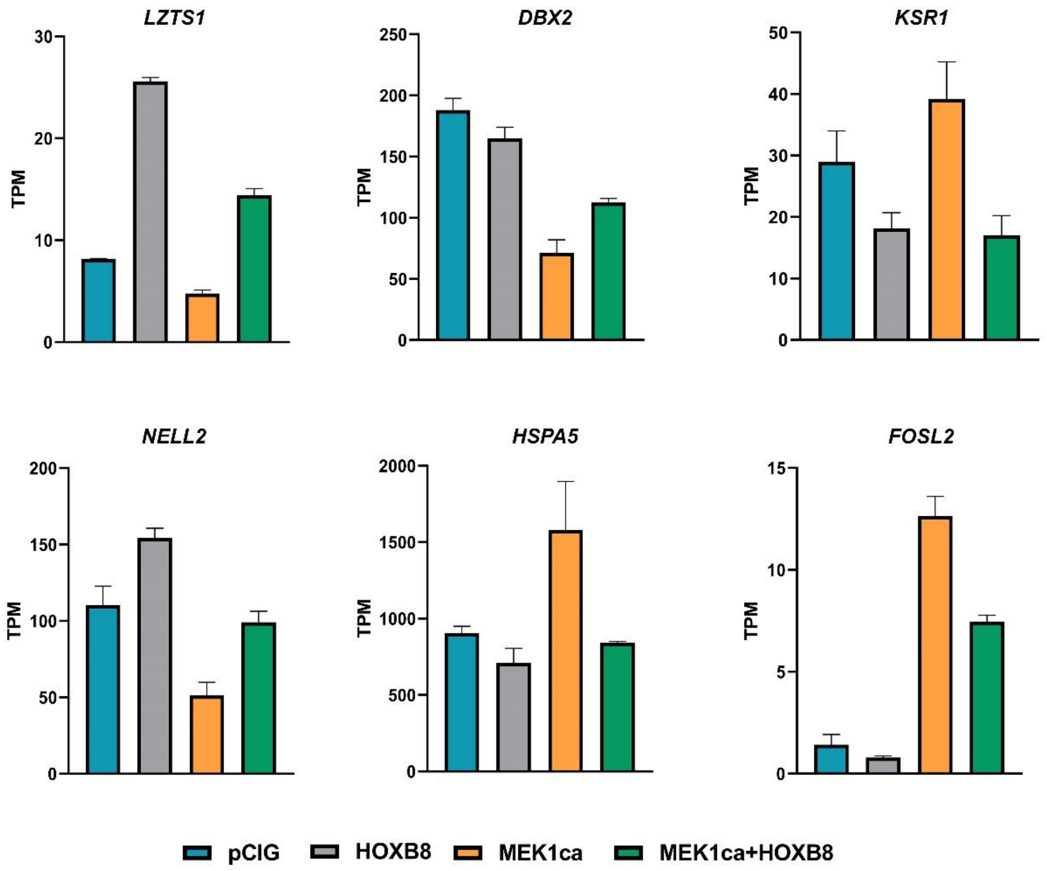

Figure S9: Graph of the mean of the number of the TPM for LZTS1, DBX2, KRS1, NELL2, HSPA5 and FOSL2 genes in the four RNAseq conditions, obtained for the two replicates of the control (pCIG1, pCIG2), HOXB8 (HOXB8-1 and HOXB8-2), MEK1ca (MEK1ca-1 and MEK1ca-2) and MEK1ca+HOXB8 (MEK1ca+HOXB8-1 and MEK1ca+HOXB8-2) expressing samples, with the corresponding p value according to the DGE (FDR= 5%5) of each pair.

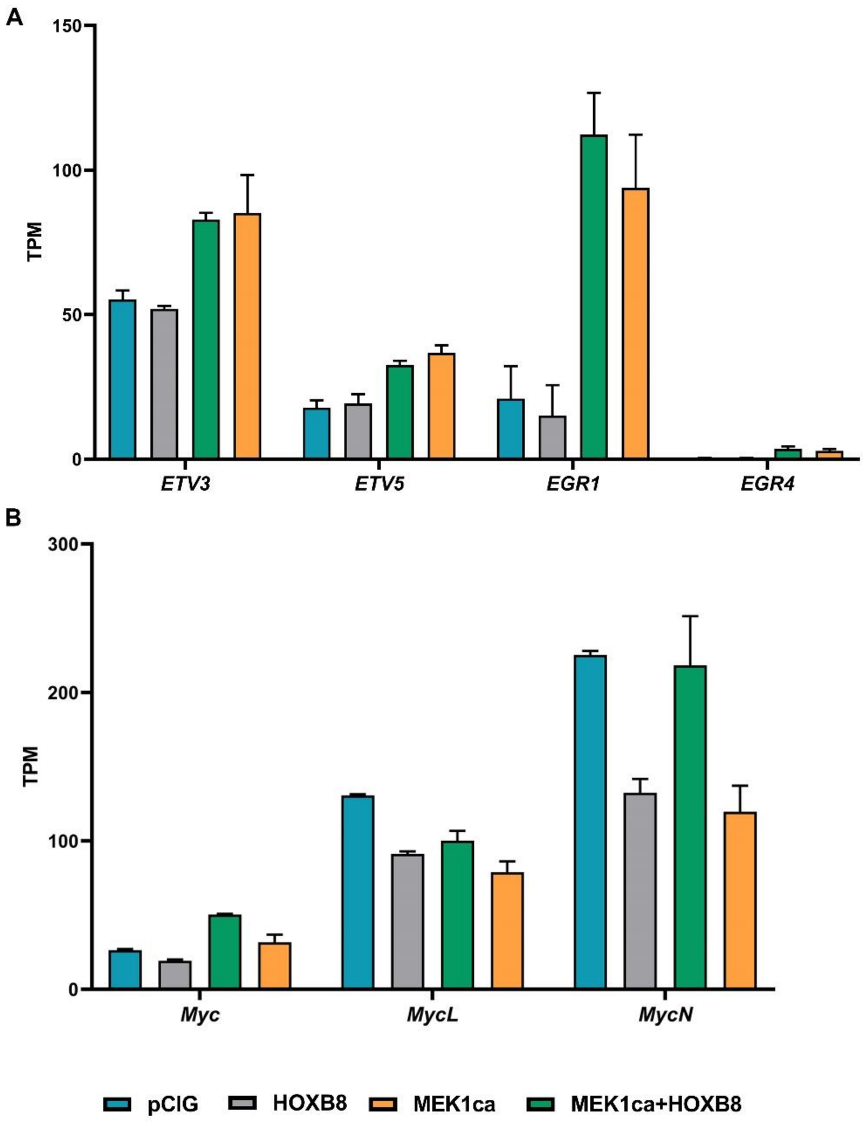

Figure S10: A and B - Graph of the mean of the number of the TPM of ERK early response genes in the four RNAseq conditions, (**A**) ETV3, ETV5, EGR1 and EGR4 genes, and (**B**) MYC, MYCL and MYCN genes, which were obtained for the two replicates of the control (pCIG1, pCIG2), HOXB8 (HOXB8-1 and HOXB8-2), MEK1ca (MEK1ca-1 and MEK1ca-2) and MEK1ca+HOXB8 (MEK1ca+HOXB8-1 and MEK1ca+HOXB8-2) expressing samples, with the corresponding p value according to the DGE (FDR= 5%) of each pair.

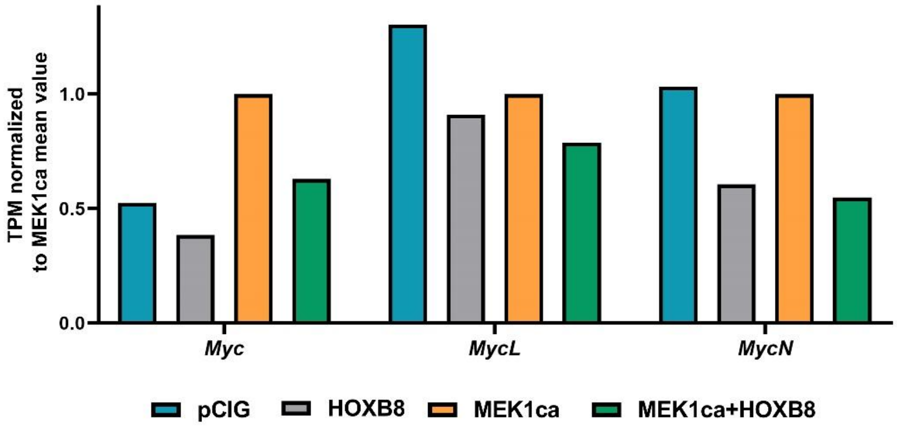

Figure S11: Graph of TPM normalized on MEK1ca value for pCIG, HOXB8 and MEK1ca+HOXB8 (mean of the two replicates of each condition) for MYC, MYCL and MYCN genes.

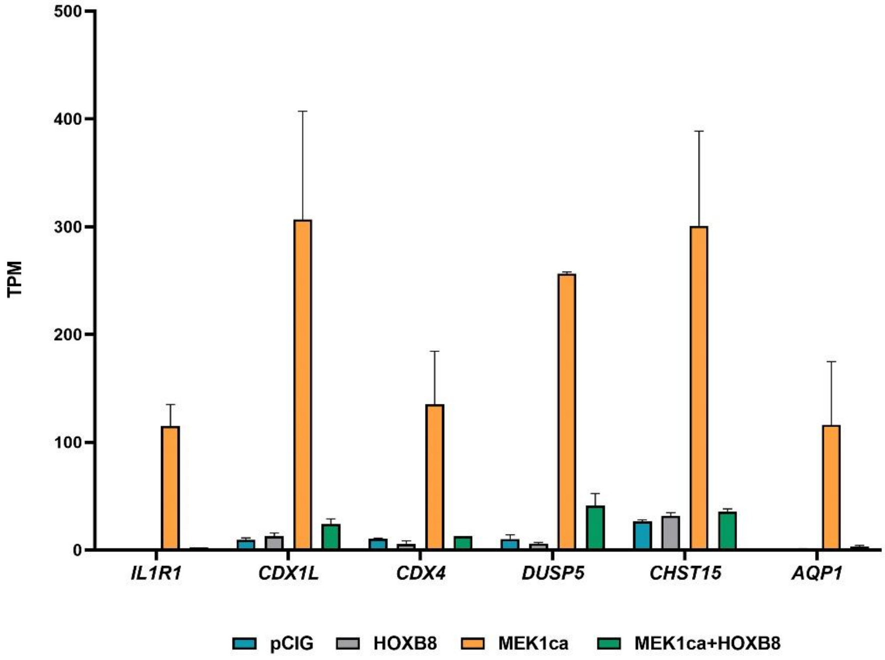

Figure S12: Graph of the mean of the number of the TPM for IL1R1, CDX1l, CDX4, DUSP5, CHST15 and AQP1 genes obtained in the four RNAseq, conditions, obtained for the two replicates of the control (pCIG1, pCIG2), HOXB8 (HOXB8-1 and HOXB8-2), MEK1ca (MEK1ca-1 and MEK1ca-2) and MEK1ca+HOXB8 (MEK1ca+HOXB8-1 and MEK1ca+HOXB8-2) expressing samples.

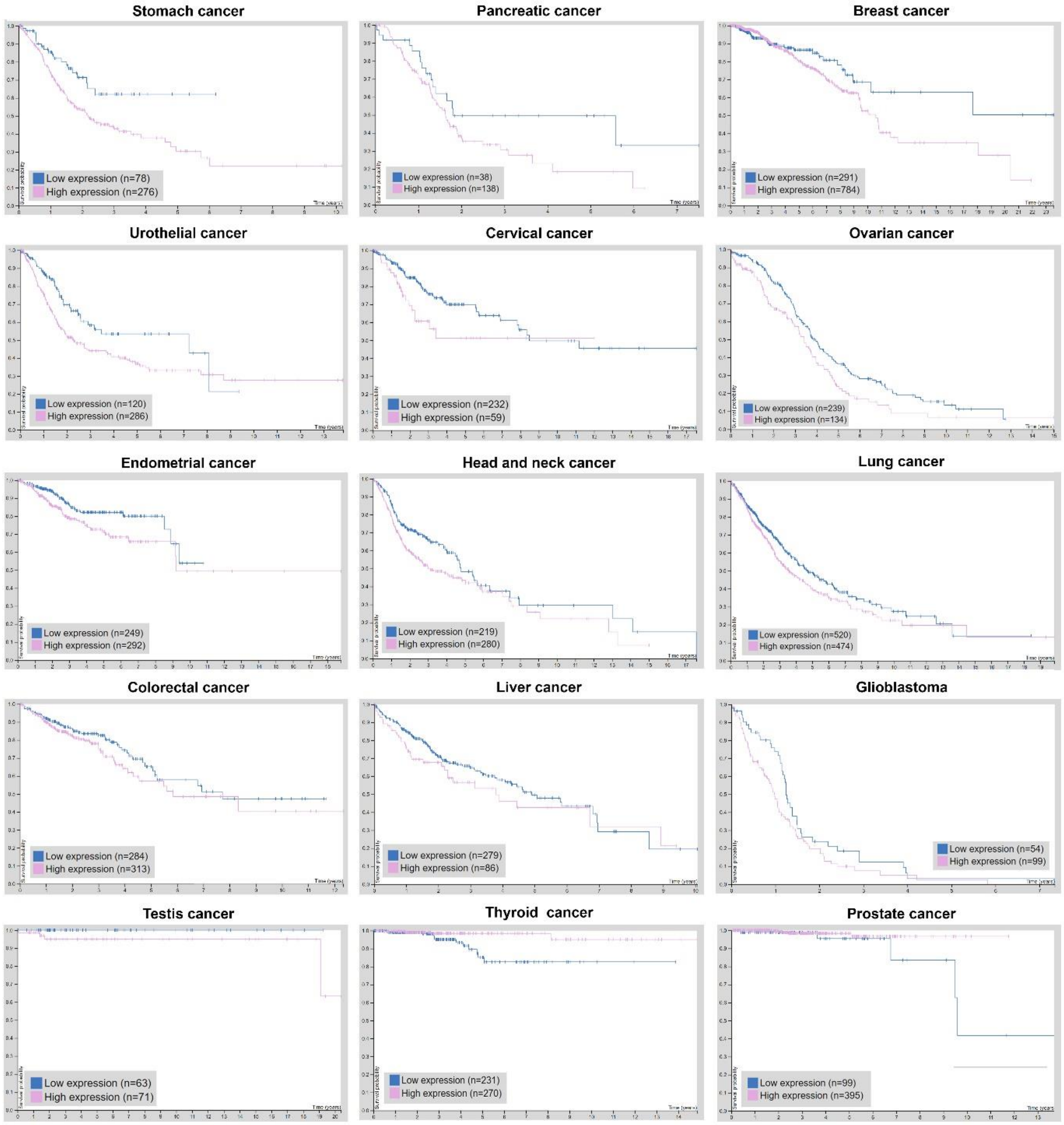

Supplementary Figure 13: Survival analysis data correlating to *CHST15* expression in several cancer types, from the Human Protein Atlas (Courtesy of Human Protein Atlas, www.proteinatlas.org, May 2021) (for all cancer types except Renal cancer and melanoma display Figure 6).
