# Supplementary material for: HOXB8 Counteracts MAPK/ERK Oncogenic Signaling in a Chicken Embryo Model of Neoplasia
Source: Int J Mol Sci. 2021 Aug 18;22(16):8911. doi: 10.3390/ijms22168911 (PMC8396257; doi:10.3390/ijms22168911)
Supplement: Supplementary file 1 [file ijms-22-08911-s001.zip › ijms-1277141 Table S1.pdf]

Table S1: MEK1ca+HOXB8 versus MEK1ca differentially regulated genes (False Discovery Rate (padj) <= 0.01)

D	baseMean	log2FoldChange	lfcSE	stat	pvalue	padj	Symbol
ENSGALG00000026853	1756,76051	5,751530736	0,19626668	29,3046727	9,04E-189	6,16E-185	FGF3
ENSGALG00000011433	901,861183	4,353428748	0,19799365	21,9877188	3,77E-107	1,28E-103	
ENSGALG00000009778	267,922713	4,29765073	0,29733506	14,4538982	2,37E-47	9,77E-45	
ENSGALG00000011582	175,170205	4,101846269	0,31827959	12,8875569	5,29E-38	1,53E-35	
ENSGALG00000027742	312,342696	3,96517198	0,26630875	14,8893793	3,86E-50	1,81E-47	KLHL33
ENSGALG00000028044	194,51466	3,90733608	0,30410231	12,848755	8,74E-38	2,48E-35	
ENSGALG00000002108	813,374631	3,799830272	0,19658831	19,328872	3,07E-83	5,23E-80	EVPL
ENSGALG00000021395	138,406928	3,681673719	0,32878732	11,1977363	4,18E-29	6,70E-27	
ENSGALG00000001080	589,095015	3,620989798	0,22311063	16,2295708	3,12E-59	2,02E-56	NR5A1
ENSGALG00000005292	233,995114	3,593969871	0,28192744	12,7478541	3,20E-37	8,90E-35	KLHL40
ENSGALG00000028376	1521,83584	3,47510163	0,19828778	17,525546	9,15E-69	7,78E-66	FGF19
ENSGALG00000023843	120,841933	3,393126537	0,33077585	10,2580842	1,09E-24	1,35E-22	FRMD7
ENSGALG00000006471	279,298959	3,309144864	0,28892507	11,4532978	2,26E-30	4,04E-28	SLCO2A1
ENSGALG00000028304	118,793101	3,251722149	0,33476041	9,71358044	2,64E-22	2,58E-20	
ENSGALG00000004526	421,691059	3,217263436	0,24373894	13,1996283	8,82E-40	2,93E-37	TNR
ENSGALG00000008069	109,411674	3,182915984	0,34138391	9,32356755	1,12E-20	9,51E-19	
ENSGALG00000004134	133,434282	3,164728278	0,32672711	9,6861515	3,45E-22	3,31E-20	GLRA1
ENSGALG00000012254	87,731989	3,162704218	0,3459847	9,14116788	6,18E-20	4,98E-18	KCNJ4
ENSGALG00000006944	469,144675	3,116432937	0,23983811	12,9939021	1,32E-38	4,01E-36	
ENSGALG00000028612	103,60478	3,070056483	0,34649284	8,86037489	7,97E-19	5,81E-17	
ENSGALG00000005285	100,237429	2,996752626	0,34025326	8,80741788	1,28E-18	8,94E-17	TBX4
ENSGALG00000007335	107,298878	2,985281892	0,34055773	8,76586154	1,85E-18	1,28E-16	KBTBD13
ENSGALG00000001256	266,944995	2,963677659	0,29793308	9,94746081	2,59E-23	2,71E-21	NLRC5
ENSGALG00000016685	93,4474561	2,945393761	0,33836846	8,70469363	3,18E-18	2,13E-16	ASMT
ENSGALG00000016721	160,3282	2,940591405	0,30420224	9,66656738	4,18E-22	3,93E-20	PLCXD1
ENSGALG00000026167	171,614092	2,925839411	0,31778334	9,20702576	3,35E-20	2,78E-18	PIK3R5
ENSGALG00000002926	76,2289496	2,900739748	0,35004614	8,28673542	1,16E-16	6,86E-15	
ENSGALG00000004700	97,8406537	2,88173675	0,33435932	8,61868224	6,77E-18	4,45E-16	NCF2
ENSGALG00000016722	73,4311203	2,829128945	0,35273145	8,0206314	1,05E-15	5,52E-14	
ENSGALG00000012830	227,950727	2,760094911	0,2851044	9,68099721	3,63E-22	3,44E-20	
ENSGALG00000027247	97,4363295	2,75413056	0,33274463	8,27700976	1,26E-16	7,41E-15	EOMES
ENSGALG00000002823	276,033949	2,746001119	0,25652175	10,7047499	9,67E-27	1,39E-24	
ENSGALG00000006614	540,926615	2,721769719	0,26010327	10,4641889	1,26E-25	1,70E-23	
ENSGALG00000006744	446,950734	2,709854582	0,26391565	10,2678814	9,83E-25	1,23E-22	
ENSGALG00000020485	352,825328	2,707785881	0,28649565	9,45140328	3,34E-21	2,90E-19	
ENSGALG00000015322	401,95748	2,694878773	0,24687156	10,9161165	9,65E-28	1,43E-25	IMPG2
ENSGALG00000025886	234,932728	2,681244585	0,27696529	9,68079628	3,64E-22	3,44E-20	SUSD3
ENSGALG00000013922	145,692651	2,651462599	0,31447645	8,43135507	3,42E-17	2,11E-15	
ENSGALG00000026590	256,390276	2,649598672	0,26552851	9,97858437	1,89E-23	2,01E-21	
ENSGALG00000016325	669,814955	2,632246576	0,2229691	11,8054319	3,66E-32	7,66E-30	
ENSGALG00000026627	138,559837	2,620280418	0,32529098	8,0551894	7,94E-16	4,29E-14	
ENSGALG00000001780	98,3971	2,613843166	0,33586268	7,78247584	7,11E-15	3,32E-13	PROC
ENSGALG00000002987	118,456359	2,607861451	0,32067745	8,13235054	4,21E-16	2,34E-14	SERPINF2
ENSGALG00000027059	200,391038	2,606173672	0,28900153	9,01785421	1,92E-19	1,48E-17	
ENSGALG00000023821	140,669435	2,605691323	0,31496616	8,2729246	1,31E-16	7,64E-15	
ENSGALG00000006032	296,771768	2,599165832	0,27225162	9,54692527	1,34E-21	1,19E-19	
ENSGALG00000002960	103,932494	2,574242623	0,33553695	7,67200941	1,69E-14	7,76E-13	HMGCS2
ENSGALG00000010841	110,200839	2,5587632	0,33214766	7,70369186	1,32E-14	6,10E-13	
ENSGALG00000002829	649,133449	2,553310959	0,20495773	12,4577442	1,27E-35	3,20E-33	
ENSGALG00000021198	61,5032288	2,552636185	0,35110067	7,27038252	3,58E-13	1,39E-11	
ENSGALG00000015141	58,9626486	2,548558749	0,35207483	7,23868481	4,53E-13	1,74E-11	GDA
ENSGALG00000005122	338,26914	2,528381619	0,24275499	10,415364	2,11E-25	2,79E-23	MYO1H
ENSGALG00000010145	278,247492	2,508391386	0,30141971	8,3219221	8,65E-17	5,15E-15	MDGA1
ENSGALG00000014372	67,9784132	2,495880293	0,34928387	7,14570731	8,95E-13	3,29E-11	SLC34A2
ENSGALG00000029144	72,3286127	2,46980754	0,34882621	7,08033811	1,44E-12	5,17E-11	
ENSGALG00000021399	89,3295109	2,464358792	0,33686101	7,31565468	2,56E-13	1,01E-11	
ENSGALG00000001111	752,305162	2,451755117	0,21130589	11,6028718	3,98E-31	7,64E-29	
ENSGALG00000012985	261,612669	2,44205884	0,26586807	9,18522799	4,11E-20	3,39E-18	SLC6A13
ENSGALG00000008105	47,87761	2,404806479	0,35395793	6,79404614	1,09E-11	3,44E-10	

ENSGALG00000012812	176,061446	2,39422427	0,29597013	8,08941196	6,00E-16	3,29E-14	SVOPL
ENSGALG00000002638	117,914057	2,388124012	0,32831388	7,27390518	3,49E-13	1,35E-11	
ENSGALG00000002318	63,4096631	2,376044719	0,3494777	6,79884494	1,05E-11	3,33E-10	LVRN
ENSGALG00000002015	214,323208	2,374226325	0,28640932	8,28962651	1,14E-16	6,72E-15	
ENSGALG00000026256	199,837668	2,357924834	0,33320574	7,0764832	1,48E-12	5,30E-11	
ENSGALG00000007395	402,677514	2,356587415	0,22616715	10,4196716	2,02E-25	2,69E-23	ABCC2
ENSGALG00000002046	463,721693	2,341658671	0,24228891	9,66473716	4,26E-22	3,97E-20	
ENSGALG00000007340	53,630639	2,338672246	0,3529687	6,62572131	3,46E-11	1,01E-09	
ENSGALG00000023934	52,5759497	2,337998666	0,35383511	6,60759382	3,91E-11	1,12E-09	
ENSGALG00000028341	324,387866	2,327426751	0,23601987	9,86114768	6,13E-23	6,23E-21	
ENSGALG00000002260	279,413842	2,326075861	0,254913	9,12497939	7,17E-20	5,71E-18	CISH
ENSGALG00000006402	199,281869	2,315345967	0,27769375	8,33776765	7,57E-17	4,56E-15	
ENSGALG00000008709	98,4372913	2,291854243	0,32683951	7,0121701	2,35E-12	8,17E-11	MCOLN3
ENSGALG00000013006	357,98483	2,275189997	0,25794845	8,8203283	1,14E-18	8,05E-17	ROPN1L
ENSGALG00000006992	182,223575	2,270300547	0,29844555	7,60708468	2,80E-14	1,27E-12	MMP9
ENSGALG00000011113	527,000372	2,26759493	0,2713629	8,35631896	6,47E-17	3,93E-15	SGIP1
ENSGALG00000023577	321,7197	2,257832359	0,26699218	8,45654861	2,75E-17	1,74E-15	
ENSGALG00000016852	56,0309211	2,256531595	0,35169224	6,41621099	1,40E-10	3,74E-09	
ENSGALG00000027617	219,458041	2,255993416	0,28513386	7,91205035	2,53E-15	1,25E-13	
ENSGALG00000003677	113,607816	2,250263529	0,34495968	6,52326543	6,88E-11	1,92E-09	NTNG2
ENSGALG00000023742	249,604133	2,24719921	0,28204891	7,96740977	1,62E-15	8,26E-14	
ENSGALG00000001589	111,257666	2,246033051	0,34415728	6,52618204	6,75E-11	1,89E-09	
ENSGALG00000016369	952,122279	2,242110682	0,1770401	12,6644225	9,31E-37	2,54E-34	
ENSGALG00000027703	404,868805	2,238234147	0,25355456	8,82742624	1,07E-18	7,64E-17	
ENSGALG00000003389	248,600828	2,230534118	0,27052364	8,24524646	1,65E-16	9,51E-15	
ENSGALG00000015244	123,748282	2,228224412	0,32046392	6,95312109	3,57E-12	1,20E-10	TBX19
ENSGALG00000001857	255,91037	2,203449137	0,25950427	8,49099387	2,05E-17	1,31E-15	FAM132A
ENSGALG00000019861	47,6042668	2,196772492	0,35394524	6,2065321	5,42E-10	1,33E-08	
ENSGALG00000008514	959,211971	2,19329695	0,17694652	12,3952535	2,77E-35	6,86E-33	DLL4
ENSGALG00000021714	88,4059279	2,185372855	0,33433945	6,53638944	6,30E-11	1,78E-09	
ENSGALG00000009499	136,985306	2,185164296	0,31520463	6,93252602	4,13E-12	1,37E-10	NPY5R
ENSGALG00000026467	67,1746865	2,184133106	0,34748728	6,28550519	3,27E-10	8,22E-09	
ENSGALG00000027532	181,565157	2,181261094	0,33533315	6,50475832	7,78E-11	2,16E-09	
ENSGALG00000002817	43,7219895	2,174112777	0,35312942	6,15670254	7,43E-10	1,79E-08	
ENSGALG00000028339	572,220786	2,143746023	0,25356336	8,45447874	2,80E-17	1,76E-15	
ENSGALG00000003548	717,979314	2,131798574	0,1846233	11,546747	7,67E-31	1,43E-28	BAIAP2L1
ENSGALG00000029168	574,122828	2,125452005	0,26338005	8,06990504	7,04E-16	3,85E-14	TMEM27
ENSGALG00000005062	695,377154	2,112402161	0,18917938	11,1661333	5,97E-29	9,35E-27	FoxN4
ENSGALG00000001879	730,682744	2,106018407	0,18389159	11,4524996	2,28E-30	4,04E-28	
ENSGALG00000028551	2508,94496	2,105237568	0,17723341	11,8783337	1,53E-32	3,32E-30	
ENSGALG00000001696	267,698482	2,102610472	0,25640045	8,20049448	2,39E-16	1,36E-14	SAG
ENSGALG00000008275	48,5714847	2,098791425	0,35395495	5,92954394	3,04E-09	6,67E-08	
ENSGALG00000021567	284,86115	2,081191963	0,24661499	8,43903259	3,20E-17	1,99E-15	EPHA8
ENSGALG00000001874	67,2449867	2,080511409	0,35389195	5,87894534	4,13E-09	8,88E-08	
ENSGALG00000028654	92,9286403	2,074935686	0,34576621	6,00097882	1,96E-09	4,45E-08	
ENSGALG00000006260	56,9564609	2,062296513	0,3527945	5,84560272	5,05E-09	1,06E-07	KLHL30
ENSGALG00000007570	256,243886	2,055518081	0,25713147	7,99403557	1,31E-15	6,73E-14	
ENSGALG00000019527	38,8970283	2,052781357	0,35219468	5,82854162	5,59E-09	1,16E-07	SLC6A19
ENSGALG00000000656	95,7621403	2,036711968	0,34147678	5,96442297	2,46E-09	5,51E-08	
ENSGALG00000007104	125,617756	2,032418742	0,32005632	6,35019094	2,15E-10	5,59E-09	
ENSGALG00000026541	175,990929	2,028653787	0,29769667	6,81449952	9,46E-12	3,00E-10	
ENSGALG00000014203	138,123283	2,025939839	0,30058823	6,7399175	1,58E-11	4,87E-10	PARVG
ENSGALG00000020195	143,592197	2,021370929	0,30337177	6,66301589	2,68E-11	7,92E-10	
ENSGALG00000012204	102,979277	2,019423041	0,32821004	6,1528374	7,61E-10	1,83E-08	
ENSGALG00000005797	87,7942641	2,014484064	0,33418994	6,02796146	1,66E-09	3,83E-08	COL20A1
ENSGALG00000000437	37,0932943	2,013731373	0,35182907	5,72360709	1,04E-08	2,04E-07	
ENSGALG00000002931	53,6926993	2,013598707	0,3534025	5,69774883	1,21E-08	2,37E-07	
ENSGALG00000006273	107,39879	2,011386223	0,32297819	6,22762252	4,74E-10	1,16E-08	MYLK2
ENSGALG00000000722	46,5643306	2,006522785	0,35375492	5,67207026	1,41E-08	2,72E-07	
ENSGALG00000028147	229,824659	2,001096858	0,26643674	7,51058915	5,89E-14	2,49E-12	CTSK
ENSGALG00000004248	56,9012525	1,998749091	0,35206496	5,67721676	1,37E-08	2,65E-07	TACR2
ENSGALG00000003075	93,0788327	1,997443704	0,3325728	6,0060344	1,90E-09	4,34E-08	SLC34A1

ENSGALG00000001571	57,1169097	1,994083312	0,35177003	5,66871286	1,44E-08	2,77E-07	MYO1F
ENSGALG00000010458	184,378204	1,990178775	0,28021881	7,10223132	1,23E-12	4,45E-11	
ENSGALG00000011889	230,923015	1,988400853	0,26765629	7,42893376	1,09E-13	4,48E-12	PRKCH
ENSGALG00000010924	209,968742	1,986898996	0,28742319	6,91279987	4,75E-12	1,56E-10	cNFI-A
ENSGALG00000015846	313,730622	1,982793598	0,24658548	8,04099914	8,91E-16	4,78E-14	SNAP91
ENSGALG00000006835	112,628775	1,974805269	0,3171624	6,22647971	4,77E-10	1,17E-08	TNNC2
ENSGALG00000013253	157,862049	1,972349113	0,31981557	6,16714546	6,95E-10	1,68E-08	INSRR
ENSGALG00000009302	367,162682	1,971912015	0,28798838	6,84719294	7,53E-12	2,42E-10	EMX2
ENSGALG00000004414	101,957841	1,959564216	0,32123225	6,10014788	1,06E-09	2,50E-08	
ENSGALG00000009489	127,740493	1,956908773	0,32047595	6,1062578	1,02E-09	2,42E-08	
ENSGALG00000005822	147,056462	1,951526015	0,30270273	6,44700496	1,14E-10	3,07E-09	KCNQ2
ENSGALG00000027147	228,058252	1,950896881	0,28014131	6,96397433	3,31E-12	1,13E-10	
ENSGALG00000021869	645,941994	1,950468731	0,24355446	8,00834733	1,16E-15	6,04E-14	PODN
ENSGALG00000028975	532,930635	1,945491073	0,20584343	9,45131484	3,35E-21	2,90E-19	
ENSGALG00000007666	61,9703125	1,945055405	0,35143193	5,53465757	3,12E-08	5,56E-07	KCHIP2
ENSGALG00000021656	37,5118215	1,943986445	0,3507973	5,5416231	3,00E-08	5,37E-07	PTPN22
ENSGALG00000008188	164,942808	1,939910796	0,29070864	6,67304152	2,51E-11	7,46E-10	TRIM36
ENSGALG00000010362	56,1305671	1,930559856	0,35381555	5,45640199	4,86E-08	8,33E-07	
ENSGALG00000000560	58,7581666	1,925570368	0,35333497	5,44970217	5,05E-08	8,61E-07	
ENSGALG00000015021	44,6818444	1,923323022	0,35395775	5,43376434	5,52E-08	9,36E-07	TMEM174
ENSGALG00000015551	1931,12818	1,921428632	0,17206371	11,1669603	5,92E-29	9,35E-27	GRIN3A
ENSGALG00000010867	80,2669926	1,919026004	0,34078038	5,63126911	1,79E-08	3,37E-07	TACSTD2
ENSGALG00000001004	271,271449	1,914258624	0,25960686	7,37368273	1,66E-13	6,69E-12	
ENSGALG00000011347	48,911798	1,909599526	0,35390611	5,39578011	6,82E-08	1,14E-06	IHH
ENSGALG00000006413	117,165789	1,90767902	0,31128952	6,12831113	8,88E-10	2,11E-08	CEMIP
ENSGALG00000003836	138,248419	1,907173269	0,34505436	5,52716754	3,25E-08	5,78E-07	
ENSGALG00000007345	111,972561	1,905774604	0,32456789	5,87172866	4,31E-09	9,20E-08	
ENSGALG00000008701	97,1365525	1,905747917	0,3400514	5,60429376	2,09E-08	3,87E-07	XDH
ENSGALG00000010853	484,768782	1,904256394	0,21726773	8,76456169	1,88E-18	1,29E-16	C8B
ENSGALG00000000221	51,6768021	1,900065815	0,35396003	5,3680237	7,96E-08	1,31E-06	
ENSGALG00000001617	1078,32932	1,898502237	0,21231379	8,9419639	3,82E-19	2,86E-17	CAMKK1
ENSGALG00000019290	78,1238371	1,892925943	0,34969839	5,41302448	6,20E-08	1,04E-06	SLC6A12
ENSGALG00000009265	88,1743252	1,891791729	0,34591457	5,46895654	4,53E-08	7,80E-07	KCNK18
ENSGALG00000003923	2335,33874	1,889033194	0,15037048	12,5625268	3,39E-36	8,88E-34	COL6A3
ENSGALG00000008995	104,546365	1,882652598	0,3282959	5,73462107	9,77E-09	1,93E-07	
ENSGALG00000000544	347,726242	1,877518925	0,23287565	8,06232383	7,49E-16	4,08E-14	RAB44
ENSGALG00000007706	86,703491	1,877381913	0,33476816	5,60800615	2,05E-08	3,82E-07	FGF8
ENSGALG00000015347	73,1732063	1,876981559	0,34452788	5,44798159	5,09E-08	8,66E-07	ZPLD1
ENSGALG00000009078	124,066106	1,868595491	0,33382944	5,59745563	2,18E-08	4,01E-07	GALNT14
ENSGALG00000000899	303,594278	1,867380532	0,25292797	7,38305256	1,55E-13	6,29E-12	
ENSGALG00000023459	62,5233629	1,863475253	0,35063414	5,31458595	1,07E-07	1,71E-06	
ENSGALG00000013953	153,510812	1,853929495	0,31615798	5,86393381	4,52E-09	9,60E-08	
ENSGALG00000020316	66,9879377	1,852228829	0,34370538	5,38900158	7,09E-08	1,17E-06	IL13RA2
ENSGALG00000014770	4689,68364	1,850573228	0,19171766	9,65259643	4,79E-22	4,41E-20	
ENSGALG00000015406	32,8242769	1,850052547	0,34954162	5,29279624	1,20E-07	1,90E-06	
ENSGALG00000007047	78,9873557	1,844593336	0,3380656	5,4563178	4,86E-08	8,33E-07	GAL
ENSGALG00000011421	155,787271	1,840566258	0,29419428	6,25629526	3,94E-10	9,81E-09	HAL
ENSGALG00000003751	52,9480261	1,835104614	0,35380373	5,18678703	2,14E-07	3,22E-06	
ENSGALG00000021693	69,3129231	1,830329106	0,34305164	5,33543326	9,53E-08	1,54E-06	
ENSGALG00000005634	92,2321464	1,826798031	0,32741142	5,57951828	2,41E-08	4,40E-07	CRYBA4
ENSGALG00000005644	54,6398918	1,819377424	0,3504577	5,19143234	2,09E-07	3,15E-06	MYO1G
ENSGALG00000029006	89,8196778	1,803532169	0,3296621	5,47085084	4,48E-08	7,73E-07	SYPL1
ENSGALG00000010126	116,195874	1,799221778	0,32988987	5,4540074	4,92E-08	8,42E-07	
ENSGALG00000019361	44,228613	1,792121861	0,35385967	5,06449882	4,09E-07	5,78E-06	FAM19A2
ENSGALG00000020982	53,8722608	1,789263062	0,35132044	5,09296598	3,53E-07	5,05E-06	
ENSGALG00000004841	236,709128	1,788099107	0,27635881	6,47020836	9,79E-11	2,66E-09	APLNR
ENSGALG00000008845	211,090699	1,784489653	0,26979829	6,61416227	3,74E-11	1,08E-09	HAO1
ENSGALG00000023354	87,3213221	1,782015396	0,34472234	5,16942237	2,35E-07	3,49E-06	FSCN2
ENSGALG00000010364	75,7123588	1,777216343	0,34965008	5,08284262	3,72E-07	5,29E-06	
ENSGALG00000027090	122,235387	1,772101833	0,32386305	5,47176288	4,46E-08	7,70E-07	
ENSGALG00000005043	168,034499	1,771501032	0,28869817	6,13617001	8,45E-10	2,02E-08	ACACB
ENSGALG00000005747	39,0007429	1,767653479	0,35360417	4,99896104	5,76E-07	7,89E-06	

ENSGALG00000014252	107,135531	1,765576737	0,34633901	5,09782814	3,44E-07	4,94E-06	
ENSGALG00000006007	80,5203103	1,763247242	0,34316546	5,13818391	2,77E-07	4,06E-06	SAMD10
ENSGALG00000000630	44,9860229	1,762139369	0,35277196	4,99512312	5,88E-07	8,02E-06	HAS3
ENSGALG00000013971	623,626799	1,754983652	0,22177863	7,91322269	2,51E-15	1,24E-13	SGK1
ENSGALG00000025804	57,8298229	1,753854275	0,34868015	5,02998031	4,91E-07	6,84E-06	CLCN2
ENSGALG00000016233	282,390757	1,751740847	0,25916092	6,75927863	1,39E-11	4,28E-10	
ENSGALG00000000299	146,622524	1,750555222	0,31737522	5,5157274	3,47E-08	6,13E-07	PKP1
ENSGALG00000015154	107,544575	1,749162012	0,31926267	5,47875512	4,28E-08	7,43E-07	TRPM6
ENSGALG00000028801	326,04496	1,749152851	0,23171525	7,54871702	4,40E-14	1,90E-12	YPEL2
ENSGALG00000003103	147,012955	1,745350116	0,2934725	5,94723559	2,73E-09	6,09E-08	MST1R
ENSGALG00000010408	48,6937618	1,74244975	0,35251743	4,94287544	7,70E-07	1,02E-05	
ENSGALG00000016580	156,416853	1,740943756	0,31375774	5,54868778	2,88E-08	5,20E-07	SCARA5
ENSGALG00000020779	287,434053	1,737619419	0,24470316	7,10092768	1,24E-12	4,48E-11	
ENSGALG00000015544	64,576519	1,719200832	0,35119647	4,89526802	9,82E-07	1,28E-05	ALDOB
ENSGALG00000015305	121,835301	1,715527315	0,30924605	5,547451	2,90E-08	5,21E-07	NR2E1
ENSGALG00000008926	1594,75407	1,713961304	0,15633781	10,9631913	5,74E-28	8,69E-26	CADPS2
ENSGALG00000002957	403,400084	1,713056332	0,23618065	7,25316116	4,07E-13	1,57E-11	SLC12A3
ENSGALG00000017212	76,5473495	1,710911672	0,34240676	4,9967228	5,83E-07	7,97E-06	GPR83
ENSGALG00000004827	60,764569	1,70800582	0,34662638	4,92751255	8,33E-07	1,10E-05	
ENSGALG00000003242	570,220114	1,707949225	0,21013146	8,12800356	4,36E-16	2,42E-14	STAR
ENSGALG00000001912	146,494381	1,699528733	0,30128418	5,64094911	1,69E-08	3,21E-07	
ENSGALG00000003003	42,6697686	1,694427776	0,35203153	4,81328409	1,48E-06	1,86E-05	CCL20
ENSGALG00000016261	158,706658	1,693178751	0,31450243	5,38367466	7,30E-08	1,20E-06	CYBB
ENSGALG00000011007	1150,32901	1,688733669	0,19077647	8,85189717	8,60E-19	6,20E-17	OSBPL3
ENSGALG00000015117	149,514374	1,688648354	0,29548541	5,71482827	1,10E-08	2,15E-07	DRD3
ENSGALG00000012429	6013,8279	1,680823476	0,15201043	11,0572901	2,02E-28	3,09E-26	BMP4
ENSGALG00000000458	427,66514	1,679795969	0,21744886	7,72501627	1,12E-14	5,20E-13	TMPRSS9
ENSGALG00000011622	28,6515601	1,67904306	0,34603089	4,85229247	1,22E-06	1,56E-05	
ENSGALG00000016572	77,2006942	1,678816771	0,34344316	4,88819394	1,02E-06	1,32E-05	
ENSGALG00000001084	353,564869	1,677708871	0,24186887	6,93643975	4,02E-12	1,34E-10	ATP8B3
ENSGALG00000026473	1522,15108	1,674638021	0,21127677	7,92627606	2,26E-15	1,13E-13	
ENSGALG00000008178	111,711666	1,67186128	0,32258264	5,18273795	2,19E-07	3,28E-06	
ENSGALG00000005140	240,214893	1,671709253	0,25797704	6,48006996	9,17E-11	2,52E-09	RRAD
ENSGALG00000001234	40,8626534	1,668621796	0,35391846	4,7147069	2,42E-06	2,87E-05	CETP
ENSGALG00000006514	1126,00554	1,665636549	0,17514671	9,50995042	1,91E-21	1,69E-19	
ENSGALG00000004936	57,472904	1,658043102	0,34946885	4,74446601	2,09E-06	2,51E-05	GLRA4
ENSGALG00000028174	599,327274	1,656109672	0,24081026	6,87723897	6,10E-12	1,98E-10	
ENSGALG00000011882	446,567699	1,655648124	0,21164389	7,82280161	5,17E-15	2,44E-13	
ENSGALG00000020143	148,781899	1,654526791	0,32189845	5,13990289	2,75E-07	4,02E-06	GABRG1
ENSGALG00000027314	27,9768099	1,648583002	0,34526179	4,77487828	1,80E-06	2,19E-05	SAXO1
ENSGALG00000019019	89,6714541	1,647447024	0,34390312	4,79043927	1,66E-06	2,05E-05	NTRK1
ENSGALG00000026161	123,209261	1,647025641	0,3125278	5,27001315	1,36E-07	2,13E-06	
ENSGALG00000024298	32,0306965	1,646495761	0,34885964	4,71965109	2,36E-06	2,81E-05	ADAMTSL5
ENSGALG00000004394	167,659182	1,640660466	0,28374074	5,7822521	7,37E-09	1,49E-07	PCBP3
ENSGALG00000016262	396,403367	1,640510999	0,22878691	7,17047578	7,47E-13	2,77E-11	XK
ENSGALG00000015905	84,004407	1,632452898	0,3358609	4,86050296	1,17E-06	1,51E-05	
ENSGALG00000005293	115,927539	1,631397185	0,32721191	4,98575121	6,17E-07	8,36E-06	
ENSGALG00000025946	137,584784	1,630278266	0,31187717	5,22730883	1,72E-07	2,64E-06	MOB3C
ENSGALG00000011806	33,3533195	1,625032877	0,34750572	4,67627665	2,92E-06	3,39E-05	
ENSGALG00000010721	516,489319	1,61713725	0,19825254	8,15695606	3,44E-16	1,92E-14	
ENSGALG00000008133	122,891283	1,61551597	0,32520429	4,96769575	6,78E-07	9,10E-06	AS3MT
ENSGALG00000000820	127,586167	1,614544959	0,31856994	5,06810204	4,02E-07	5,68E-06	HTR1D
ENSGALG00000002832	501,681621	1,610990532	0,21276938	7,57153378	3,69E-14	1,62E-12	IFI35
ENSGALG00000006606	3620,8517	1,610976489	0,12247757	13,1532366	1,63E-39	5,29E-37	
ENSGALG00000011200	907,828768	1,609360966	0,24666698	6,52442818	6,83E-11	1,91E-09	THBS2
ENSGALG00000007980	258,344877	1,607363167	0,27462432	5,85295284	4,83E-09	1,02E-07	KCNG1
ENSGALG00000001062	680,579051	1,606326071	0,19183595	8,37343595	5,60E-17	3,43E-15	GGT7
ENSGALG00000003106	661,981272	1,604428011	0,22228284	7,21795705	5,28E-13	2,01E-11	TOX2
ENSGALG00000007815	333,865811	1,603027021	0,27971291	5,73097251	9,99E-09	1,96E-07	ANKK1
ENSGALG00000015366	80,0727703	1,602600172	0,34326453	4,66870313	3,03E-06	3,52E-05	
ENSGALG00000005725	91,1085716	1,601523904	0,35202702	4,54943466	5,38E-06	5,90E-05	CSF1R
ENSGALG00000022644	26,8742275	1,595235245	0,34379409	4,64008918	3,48E-06	3,98E-05	

ENSGALG00000007759	856,866355	1,594985498	0,19890567	8,01880348	1,07E-15	5,57E-14	
ENSGALG00000008830	781,049383	1,591806155	0,21140245	7,52974324	5,08E-14	2,17E-12	
ENSGALG00000006412	53,0587145	1,591456503	0,35279252	4,51102674	6,45E-06	6,90E-05	VGLL1
ENSGALG00000029174	5348,15015	1,589321167	0,12909013	12,3117174	7,83E-35	1,87E-32	
ENSGALG00000006245	402,970032	1,589094556	0,22775101	6,97733278	3,01E-12	1,04E-10	
ENSGALG00000026733	77,5005998	1,588089103	0,34066443	4,6617403	3,14E-06	3,62E-05	FFAR4
ENSGALG00000016138	93,2512783	1,581494384	0,32966262	4,79731185	1,61E-06	2,00E-05	DSCAM
ENSGALG00000016498	94,7917055	1,580375213	0,32949342	4,7963786	1,62E-06	2,01E-05	MFSD2B
ENSGALG00000011343	80,565753	1,579403764	0,33480013	4,71745272	2,39E-06	2,83E-05	CNPPD1
ENSGALG00000000733	1063,13098	1,578595816	0,19876941	7,94184501	1,99E-15	1,00E-13	
ENSGALG00000028175	72,9348346	1,575180638	0,35396003	4,45016529	8,58E-06	9,01E-05	GJA9
ENSGALG00000015293	333,004659	1,574970296	0,24444617	6,4430147	1,17E-10	3,15E-09	TMEM45A
ENSGALG00000012115	228,294388	1,572479232	0,27344667	5,75058837	8,89E-09	1,77E-07	WT1
ENSGALG00000004692	57,025385	1,559793633	0,35370154	4,40991479	1,03E-05	0,0001064	
ENSGALG00000005958	558,635772	1,558583818	0,23299183	6,68943537	2,24E-11	6,70E-10	TRIM66
ENSGALG00000000826	97,953466	1,551558986	0,32368653	4,79339992	1,64E-06	2,03E-05	
ENSGALG00000007690	2070,9973	1,54950396	0,17555383	8,82637523	1,08E-18	7,67E-17	TFAP2C
ENSGALG00000006964	225,191012	1,546629935	0,29302707	5,27811285	1,31E-07	2,05E-06	
ENSGALG00000005967	735,636675	1,545091384	0,22167917	6,96994379	3,17E-12	1,09E-10	
ENSGALG00000008440	429,095272	1,543692993	0,21647003	7,13120884	9,95E-13	3,64E-11	CHST1
ENSGALG00000001882	38,7098275	1,540579881	0,35211396	4,37523089	1,21E-05	0,00012243	
ENSGALG00000015373	27,6017686	1,538791481	0,34451153	4,46658922	7,95E-06	8,37E-05	PDE6B
ENSGALG00000011577	399,56293	1,531134988	0,21728602	7,0466337	1,83E-12	6,48E-11	CNTNAP5
ENSGALG00000025774	5724,96959	1,527733684	0,12704021	12,0255916	2,61E-33	5,82E-31	
ENSGALG00000005473	107,686637	1,527501434	0,32073027	4,76257338	1,91E-06	2,31E-05	
ENSGALG00000000479	244,695616	1,527264642	0,25520876	5,984374	2,17E-09	4,90E-08	
ENSGALG00000011071	944,33324	1,527069671	0,16947196	9,01075121	2,05E-19	1,57E-17	
ENSGALG00000020459	25,5154221	1,526419374	0,34167333	4,46748172	7,91E-06	8,34E-05	
ENSGALG00000014267	99,3352174	1,525149591	0,31972846	4,77014025	1,84E-06	2,24E-05	RBM47
ENSGALG00000007803	165,807287	1,522432144	0,29566688	5,14914667	2,62E-07	3,86E-06	
ENSGALG00000001569	88,7153734	1,518412601	0,34222784	4,43684706	9,13E-06	9,51E-05	RHOC
ENSGALG00000020745	195,274039	1,515524715	0,27430933	5,52487479	3,30E-08	5,85E-07	RHO
ENSGALG00000011227	778,979814	1,515331444	0,18180249	8,3350423	7,75E-17	4,65E-15	CDS1
ENSGALG00000007594	126,922974	1,512071087	0,30201682	5,00657906	5,54E-07	7,63E-06	ASB12
ENSGALG00000002314	85,1314877	1,511687395	0,33330967	4,53538412	5,75E-06	6,23E-05	CDHR1
ENSGALG00000002353	221,898313	1,511078773	0,26237238	5,75929059	8,45E-09	1,69E-07	C1orf216
ENSGALG00000024397	46,3770198	1,510326295	0,3539532	4,2670226	1,98E-05	0,00018598	
ENSGALG00000023866	254,336531	1,510055693	0,28025871	5,38807762	7,12E-08	1,18E-06	
ENSGALG00000026089	41,8112969	1,509966763	0,35044188	4,30875091	1,64E-05	0,00015808	SLC26A6
ENSGALG00000028386	982,725203	1,507238343	0,16997711	8,86730176	7,49E-19	5,49E-17	
ENSGALG00000006479	34,3468527	1,50588664	0,34819791	4,32480087	1,53E-05	0,00014837	CCM2L
ENSGALG00000005297	304,659703	1,505251197	0,24557254	6,12955833	8,81E-10	2,10E-08	HHATL
ENSGALG00000004448	547,114848	1,504790559	0,23230258	6,4777179	9,31E-11	2,55E-09	BRINP2
ENSGALG00000026983	590,103546	1,50209184	0,21370636	7,02876535	2,08E-12	7,31E-11	CHRD
ENSGALG00000024264	40,6812127	1,501303133	0,34955904	4,2948485	1,75E-05	0,00016679	
ENSGALG00000028347	76,9725475	1,501152953	0,34262878	4,38128098	1,18E-05	0,00011917	SDCBP2
ENSGALG00000016556	93,5327309	1,500831481	0,32530567	4,6136038	3,96E-06	4,47E-05	
ENSGALG00000008424	31,5559973	1,500581439	0,34950735	4,29341889	1,76E-05	0,00016751	CALML3
ENSGALG00000016287	253,693238	1,497802744	0,250661	5,9754121	2,30E-09	5,17E-08	
ENSGALG00000028069	276,234024	1,497716991	0,25564361	5,858613	4,67E-09	9,87E-08	WNT16
ENSGALG00000015410	210,477674	1,497100835	0,26827858	5,58039641	2,40E-08	4,38E-07	BVES
ENSGALG00000014074	676,433259	1,49566297	0,19193599	7,79250911	6,57E-15	3,08E-13	
ENSGALG00000003713	62,0071346	1,494159857	0,34951056	4,2750063	1,91E-05	0,00018034	
ENSGALG00000007409	87,0801504	1,493981037	0,32899137	4,54109494	5,60E-06	6,09E-05	SCN4B
ENSGALG00000028823	172,118142	1,491796906	0,2989787	4,98964273	6,05E-07	8,21E-06	
ENSGALG00000026585	53,5711009	1,487448109	0,3507492	4,24077402	2,23E-05	0,00020575	
ENSGALG00000027548	35,7639328	1,48608799	0,3490327	4,25773286	2,07E-05	0,00019271	
ENSGALG00000028770	169,214437	1,483894729	0,30979476	4,78992848	1,67E-06	2,06E-05	INHBB
ENSGALG00000015237	27,2937688	1,481694783	0,34447168	4,30135446	1,70E-05	0,00016265	PPIL6
ENSGALG00000015084	1284,76928	1,480304896	0,18534154	7,98690291	1,38E-15	7,11E-14	IGSF11
ENSGALG00000027357	83,3635346	1,478068348	0,33426191	4,4218869	9,78E-06	0,000101	
ENSGALG00000007908	523,875729	1,477721596	0,21194377	6,97223428	3,12E-12	1,08E-10	

ENSGALG00000008888	216,095633	1,475198435	0,26634382	5,5386996	3,05E-08	5,45E-07	
ENSGALG00000000984	31,631294	1,47391651	0,3504148	4,2062051	2,60E-05	0,00023462	
ENSGALG00000007862	431,761835	1,47265082	0,23340463	6,30943289	2,80E-10	7,11E-09	CNTN3
ENSGALG00000016538	1671,27665	1,472170836	0,14445232	10,1913963	2,17E-24	2,59E-22	RAI2
ENSGALG00000002442	183,2009	1,464263461	0,28517222	5,13466379	2,83E-07	4,12E-06	ANKS4B
ENSGALG00000010107	1860,62891	1,462273804	0,14178721	10,3131576	6,14E-25	7,75E-23	
ENSGALG00000026677	510,78911	1,461550873	0,27216608	5,37006991	7,87E-08	1,29E-06	F10
ENSGALG00000000703	748,407891	1,459568013	0,22886918	6,37730267	1,80E-10	4,77E-09	SLC45A3
ENSGALG00000026069	33,9757189	1,459502097	0,34690094	4,20725901	2,58E-05	0,00023392	
ENSGALG00000001319	188,5387	1,458388464	0,27459113	5,31112739	1,09E-07	1,73E-06	CAMK1G
ENSGALG00000014297	511,060783	1,457150717	0,20851139	6,98835066	2,78E-12	9,64E-11	IRF7
ENSGALG00000004565	123,010215	1,456247274	0,33891637	4,29677471	1,73E-05	0,00016569	
ENSGALG00000010024	105,10355	1,453185654	0,31957147	4,5472947	5,43E-06	5,94E-05	
ENSGALG00000014941	729,891458	1,451697765	0,1864124	7,78756017	6,83E-15	3,20E-13	
ENSGALG00000018878	40,6695287	1,44967545	0,35380851	4,09734482	4,18E-05	0,00035297	
ENSGALG00000015128	44,3465543	1,44940013	0,35395981	4,09481559	4,23E-05	0,00035587	AQP4
ENSGALG00000006751	459,348637	1,449311054	0,21417443	6,76696594	1,32E-11	4,09E-10	THY1
ENSGALG00000017041	876,052013	1,44806939	0,20136843	7,19114421	6,43E-13	2,41E-11	
ENSGALG00000007211	323,240319	1,44654086	0,24592568	5,88202449	4,05E-09	8,74E-08	CDH22
ENSGALG00000001727	728,893661	1,445847057	0,18026579	8,02064026	1,05E-15	5,52E-14	LZTS1
ENSGALG00000006239	29,3987017	1,443346989	0,34917352	4,13360955	3,57E-05	0,00030929	
ENSGALG00000023395	151,597821	1,441830671	0,29992743	4,80726505	1,53E-06	1,91E-05	PLIN1
ENSGALG00000013640	91,8229663	1,440450208	0,32437446	4,44070167	8,97E-06	9,38E-05	
ENSGALG00000014962	26,4645058	1,440003001	0,34319536	4,19586969	2,72E-05	0,00024358	FAM26F
ENSGALG00000008267	52,9646856	1,439464257	0,35338111	4,07340464	4,63E-05	0,0003844	IL5RA
ENSGALG00000011202	37,1408249	1,438867662	0,3522352	4,08496273	4,41E-05	0,00036755	COLQ
ENSGALG00000012652	778,180225	1,438231617	0,2169215	6,63019384	3,35E-11	9,79E-10	
ENSGALG00000001710	36,6972134	1,437606263	0,3472289	4,14022641	3,47E-05	0,00030127	
ENSGALG00000014695	237,142454	1,436054042	0,28464919	5,0449961	4,54E-07	6,35E-06	CLCN1
ENSGALG00000024368	80,858185	1,435967902	0,33830768	4,24456188	2,19E-05	0,00020286	
ENSGALG00000003678	3218,39181	1,429238494	0,12475534	11,4563312	2,19E-30	3,97E-28	
ENSGALG00000012313	84,3836892	1,428353126	0,34635998	4,1238977	3,73E-05	0,00031938	
ENSGALG00000000914	24,8181662	1,42659313	0,3378824	4,22215874	2,42E-05	0,00022111	
ENSGALG00000006565	175,499575	1,424795357	0,32613752	4,36869506	1,25E-05	0,00012543	
ENSGALG00000011888	40,65783	1,420709802	0,353895	4,01449525	5,96E-05	0,00047852	
ENSGALG00000010213	97,7805158	1,420151903	0,3251042	4,36829761	1,25E-05	0,00012548	
ENSGALG00000008901	121,607035	1,417468594	0,30853867	4,59413595	4,35E-06	4,87E-05	FP
ENSGALG00000009596	108,17405	1,416089625	0,32143686	4,40549862	1,06E-05	0,00010813	TMEM117
ENSGALG00000014412	49,0629688	1,415817045	0,35185351	4,02388215	5,72E-05	0,00046283	CSTA
ENSGALG00000000589	81,385408	1,415474788	0,33862107	4,18011435	2,91E-05	0,00025877	
ENSGALG00000023834	58,0264688	1,413727582	0,35080504	4,02995233	5,58E-05	0,00045266	
ENSGALG00000018998	220,946842	1,412120959	0,28106361	5,02420412	5,06E-07	7,03E-06	
ENSGALG00000013081	81,6196368	1,411618895	0,34436277	4,09922035	4,15E-05	0,00035078	VWC2
ENSGALG00000027520	176,34242	1,409426755	0,294554	4,78495208	1,71E-06	2,10E-05	
ENSGALG00000020423	91,6730687	1,409349068	0,34445303	4,09155661	4,28E-05	0,00035977	
ENSGALG00000026957	482,71907	1,407844107	0,20929005	6,72676095	1,73E-11	5,28E-10	SEMA4G
ENSGALG00000010486	221,460325	1,406315246	0,26026568	5,40338331	6,54E-08	1,10E-06	
ENSGALG00000028936	318,091175	1,405167114	0,23410156	6,0023824	1,94E-09	4,43E-08	
ENSGALG00000002637	48,9524533	1,404353552	0,35337987	3,97406211	7,07E-05	0,00055575	RBP7
ENSGALG00000026948	265,898108	1,40406892	0,28394262	4,94490374	7,62E-07	1,01E-05	ADD2
ENSGALG00000016782	106,880394	1,400781577	0,32848891	4,26431921	2,01E-05	0,00018802	
ENSGALG00000015918	313,695882	1,399804901	0,23804553	5,88040816	4,09E-09	8,82E-08	
ENSGALG00000011594	42,2108128	1,39976735	0,35390556	3,95520025	7,65E-05	0,00059291	NR1H4
ENSGALG00000009390	538,734312	1,398258988	0,20555082	6,80249786	1,03E-11	3,26E-10	
ENSGALG00000004252	60,1197029	1,3977567	0,34729606	4,02468343	5,71E-05	0,00046153	
ENSGALG00000019864	58,8923664	1,392548079	0,34877295	3,9927067	6,53E-05	0,00051768	
ENSGALG00000029083	179,607144	1,390642479	0,28146869	4,94066488	7,79E-07	1,03E-05	NXPH2
ENSGALG00000012875	68,4672034	1,389711553	0,3440597	4,03915817	5,36E-05	0,0004376	WEE2
ENSGALG00000023689	74,9775085	1,388126911	0,34057145	4,07587575	4,58E-05	0,0003808	ASS1
ENSGALG00000026536	52,5121501	1,387507758	0,35369019	3,92294664	8,75E-05	0,00067057	IP6K3
ENSGALG00000014857	696,584571	1,387229623	0,18283909	7,58716103	3,27E-14	1,45E-12	SELENOP
ENSGALG00000002319	151,603804	1,386034084	0,29385541	4,71672129	2,40E-06	2,84E-05	TEKT2

ENSGALG00000015776	28,4892202	1,382386109	0,34372541	4,02177453	5,78E-05	0,00046644	GABRR2
ENSGALG00000007636	33,4789778	1,381489214	0,3508105	3,93799273	8,22E-05	0,00063274	PCK1
ENSGALG00000007014	150,696913	1,378668928	0,28986414	4,75625908	1,97E-06	2,38E-05	
ENSGALG00000027973	59,7315947	1,378158223	0,3525539	3,90907098	9,27E-05	0,0007038	CATHL1
ENSGALG00000017099	99,5011022	1,377440865	0,3228202	4,26689803	1,98E-05	0,00018598	RASL11A
ENSGALG00000005493	91,8625446	1,375327372	0,3276333	4,19776428	2,70E-05	0,00024241	ITGB1BP2
ENSGALG00000006522	45,3533386	1,373172	0,35333674	3,88629841	0,00010178	0,00076226	HCK
ENSGALG00000012956	1426,50489	1,372804533	0,18631922	7,36802439	1,73E-13	6,93E-12	TMTC1
ENSGALG00000002105	44,5411838	1,371897103	0,35367265	3,8790025	0,00010489	0,00078376	FOXI1
ENSGALG00000016584	157,587524	1,371284929	0,29956385	4,57760477	4,70E-06	5,25E-05	EGFL6
ENSGALG00000000730	181,141324	1,369179792	0,28016862	4,88698482	1,02E-06	1,33E-05	
ENSGALG00000009151	104,263114	1,368816021	0,32143562	4,25844529	2,06E-05	0,00019223	
ENSGALG00000027420	141,879195	1,367365407	0,29420706	4,64762942	3,36E-06	3,86E-05	POPDC3
ENSGALG00000012971	180,161283	1,365874431	0,27502418	4,96637936	6,82E-07	9,15E-06	CCDC170
ENSGALG00000005852	42,716737	1,364112477	0,35370786	3,85660773	0,00011497	0,00084385	SRMS
ENSGALG00000020458	27,1299508	1,361629377	0,33978429	4,00733472	6,14E-05	0,00049065	
ENSGALG00000013221	46,5848114	1,36089575	0,35262592	3,85931853	0,0001137	0,00083635	ARHGEF33
ENSGALG00000016155	67,5195979	1,359832396	0,3425225	3,97005278	7,19E-05	0,00056355	
ENSGALG00000004840	833,065062	1,358453061	0,16895662	8,04024766	8,97E-16	4,78E-14	RPH3A
ENSGALG00000014913	89,5865526	1,358196979	0,33321545	4,07603243	4,58E-05	0,00038078	ROS1
ENSGALG00000028822	179,379351	1,357789969	0,27926636	4,86198902	1,16E-06	1,50E-05	RNF152
ENSGALG00000001932	150,41219	1,357009459	0,31045596	4,37102081	1,24E-05	0,00012454	SRRM3
ENSGALG00000006104	74,2255943	1,356707559	0,33688447	4,02721902	5,64E-05	0,00045713	ADAMTSL3
ENSGALG00000013914	741,463509	1,355434386	0,22319941	6,0727508	1,26E-09	2,95E-08	
ENSGALG00000001863	95,5730164	1,354146203	0,34957981	3,87363962	0,00010722	0,00079659	
ENSGALG00000024064	33,3700028	1,353791502	0,35292071	3,83596505	0,00012507	0,00090819	
ENSGALG00000017036	3151,42236	1,353726483	0,14349833	9,43374367	3,96E-21	3,41E-19	LHFP
ENSGALG00000016342	64,6889555	1,353678127	0,34807358	3,88905739	0,00010063	0,00075407	MYOM2
ENSGALG00000028162	25,784822	1,353451729	0,34077076	3,97173668	7,14E-05	0,00056055	
ENSGALG00000025857	83,4628776	1,352908608	0,3353198	4,03468155	5,47E-05	0,0004451	
ENSGALG00000005898	30,8597554	1,351366295	0,34832633	3,87959844	0,00010463	0,00078228	
ENSGALG00000026817	190,25856	1,350039834	0,30726986	4,39366176	1,11E-05	0,00011375	
ENSGALG00000011259	318,18554	1,346953088	0,23787295	5,66248952	1,49E-08	2,86E-07	RPE65
ENSGALG00000015186	1129,15289	1,345677898	0,16699384	8,05824872	7,74E-16	4,20E-14	
ENSGALG00000027568	132,010856	1,344832291	0,31080687	4,32690662	1,51E-05	0,00014748	
ENSGALG00000003671	47,5471462	1,341551712	0,35388746	3,79089929	0,0001501	0,00106718	
ENSGALG00000007106	977,161228	1,340640592	0,20719828	6,47032681	9,78E-11	2,66E-09	
ENSGALG00000002466	67,363583	1,337320513	0,34594234	3,86573239	0,00011076	0,00081776	SLC2A5
ENSGALG00000008283	62,9561587	1,336619904	0,34809582	3,83980453	0,00012313	0,00089506	
ENSGALG00000009058	854,57904	1,336327117	0,17615198	7,58621684	3,29E-14	1,46E-12	
ENSGALG00000014523	712,216802	1,335395904	0,18088858	7,38242249	1,55E-13	6,30E-12	
ENSGALG00000027624	90,7091995	1,332865286	0,32442701	4,10836716	3,98E-05	0,00033971	
ENSGALG00000020380	160,77545	1,332253662	0,29757918	4,47697203	7,57E-06	8,01E-05	
ENSGALG00000014117	36,1408998	1,330198207	0,35376021	3,76016911	0,0001698	0,00119227	
ENSGALG00000005330	2519,77115	1,328511787	0,13424164	9,89642129	4,31E-23	4,45E-21	SNCAIP
ENSGALG00000013481	89,3340529	1,327640637	0,33153417	4,00453641	6,21E-05	0,00049533	IKZF3
ENSGALG00000008434	453,347477	1,327566577	0,22347729	5,94049892	2,84E-09	6,30E-08	SORCS3
ENSGALG00000006134	56,0911679	1,326807835	0,34869604	3,80505559	0,00014177	0,00101484	
ENSGALG00000004903	45,997304	1,326402261	0,35371776	3,74988883	0,00017691	0,00123332	CHST8
ENSGALG00000015309	102,618363	1,321942011	0,33018568	4,00363218	6,24E-05	0,00049694	SCML4
ENSGALG00000016551	5221,05508	1,321564075	0,11774551	11,2239022	3,11E-29	5,11E-27	AP1S2
ENSGALG00000013302	789,25879	1,321288665	0,17573754	7,51853389	5,54E-14	2,35E-12	
ENSGALG00000009315	90,4745544	1,318764229	0,33354287	3,9538073	7,69E-05	0,00059603	PAPLN
ENSGALG00000027575	153,600714	1,316495304	0,2966273	4,43821352	9,07E-06	9,46E-05	
ENSGALG00000003050	83,7827729	1,314582894	0,33514644	3,92241347	8,77E-05	0,00067168	
ENSGALG00000002792	26,9329424	1,31306542	0,34508002	3,80510415	0,00014174	0,00101484	SPDEF
ENSGALG00000016368	37,7471182	1,308737431	0,35393883	3,69763729	0,00021762	0,00147112	
ENSGALG00000002389	532,146018	1,308458099	0,19760492	6,62158668	3,55E-11	1,03E-09	ITGB4
ENSGALG00000000164	213,826705	1,307977843	0,27864581	4,69405176	2,68E-06	3,15E-05	MYBPH
ENSGALG00000007732	613,17499	1,306274335	0,18526209	7,0509534	1,78E-12	6,32E-11	RTN4R
ENSGALG00000013838	384,542651	1,305966304	0,21582461	6,05105365	1,44E-09	3,34E-08	
ENSGALG00000003316	80,9265708	1,303670454	0,33354405	3,90854055	9,29E-05	0,0007043	

ENSGALG00000005802	409,595368	1,302158184	0,2262217	5,75611525	8,61E-09	1,72E-07	FLT4
ENSGALG00000016109	151,672095	1,299400996	0,28890898	4,49761372	6,87E-06	7,31E-05	
ENSGALG00000009700	477,109619	1,299326338	0,23266824	5,58445936	2,34E-08	4,28E-07	PDK4
ENSGALG00000006045	136,045181	1,297721233	0,30150221	4,30418474	1,68E-05	0,0001609	SH3GL3
ENSGALG00000006165	67,1698457	1,296804529	0,34433853	3,76607445	0,00016583	0,00116563	WDFY4
ENSGALG00000002234	407,001304	1,296274176	0,21729503	5,96550322	2,44E-09	5,48E-08	UNC13D
ENSGALG00000002051	21,0390889	1,294841211	0,33239734	3,89546205	9,80E-05	0,00073766	
ENSGALG00000008022	294,029309	1,293915137	0,23960701	5,40015561	6,66E-08	1,11E-06	
ENSGALG00000000775	136,777503	1,291374316	0,31998815	4,03569419	5,44E-05	0,00044358	CTSS
ENSGALG00000010540	264,704566	1,289706596	0,24325268	5,30192147	1,15E-07	1,81E-06	TTC39A
ENSGALG00000005790	146,077421	1,289660896	0,31496039	4,09467652	4,23E-05	0,00035587	
ENSGALG00000004859	354,90162	1,288425333	0,24778178	5,19983886	1,99E-07	3,02E-06	ZNFX1
ENSGALG00000003118	754,378386	1,286216079	0,18775875	6,85036558	7,37E-12	2,38E-10	ADGRA2
ENSGALG00000006779	194,184542	1,285794374	0,29046865	4,42662009	9,57E-06	9,92E-05	PDZD3
ENSGALG00000012362	940,671677	1,285048698	0,17252552	7,44845563	9,44E-14	3,88E-12	THSD7B
ENSGALG00000004044	637,181076	1,285008651	0,19785383	6,49473719	8,32E-11	2,30E-09	CSRP3
ENSGALG00000003580	1991,2953	1,284312011	0,19422271	6,61257377	3,78E-11	1,09E-09	MMP2
ENSGALG00000002855	1183,64483	1,278753967	0,16211473	7,88795656	3,07E-15	1,50E-13	SARDH
ENSGALG00000016564	80,407544	1,275019755	0,34133276	3,73541576	0,00018741	0,00129256	PTK2B
ENSGALG00000001028	434,619355	1,272121671	0,22824463	5,5735011	2,50E-08	4,54E-07	RNF43
ENSGALG00000007078	141,279073	1,270778013	0,30664146	4,14418193	3,41E-05	0,00029631	
ENSGALG00000009689	798,333776	1,269454305	0,17898437	7,09254266	1,32E-12	4,74E-11	
ENSGALG00000012740	435,216388	1,269186578	0,21598232	5,8763447	4,19E-09	8,99E-08	
ENSGALG00000000909	237,543246	1,268775969	0,26254608	4,83258398	1,35E-06	1,70E-05	GFAP
ENSGALG00000016967	712,9034	1,267335006	0,21530236	5,88630325	3,95E-09	8,55E-08	ENOX1
ENSGALG00000019797	403,10583	1,266041829	0,28583293	4,42930704	9,45E-06	9,81E-05	
ENSGALG00000007562	30,6870844	1,263491411	0,34960809	3,61402221	0,00030148	0,00194997	FGF4
ENSGALG00000008953	507,742329	1,263384491	0,20357748	6,20591478	5,44E-10	1,33E-08	AASS
ENSGALG00000001795	978,865317	1,261084777	0,18732211	6,73217257	1,67E-11	5,13E-10	
ENSGALG00000012978	566,09757	1,261073108	0,1899643	6,63847421	3,17E-11	9,30E-10	CDYL2
ENSGALG00000006725	183,577155	1,259943343	0,32489293	3,87802635	0,00010531	0,00078648	ACAN
ENSGALG00000014398	578,994538	1,259804122	0,2168754	5,80888439	6,29E-09	1,29E-07	
ENSGALG00000015598	190,820863	1,259472248	0,27786596	4,53266109	5,82E-06	6,30E-05	RASGRF2
ENSGALG00000002647	518,746939	1,258418808	0,24512541	5,13377553	2,84E-07	4,13E-06	PLS1
ENSGALG00000003490	147,804607	1,25835771	0,29722539	4,23368174	2,30E-05	0,0002112	
ENSGALG00000005458	78,5157022	1,258264066	0,3397845	3,70312374	0,00021296	0,00144324	RPL3L
ENSGALG00000005843	363,336884	1,25767053	0,2724846	4,61556557	3,92E-06	4,43E-05	EEF1A2
ENSGALG00000006809	89,4927086	1,25736391	0,32504634	3,86826053	0,00010961	0,00081065	POU2AF1
ENSGALG00000014014	690,531459	1,255177749	0,17796048	7,05312653	1,75E-12	6,24E-11	
ENSGALG00000001348	34,354329	1,253857774	0,350669	3,57561625	0,0003494	0,00221571	TEKT3
ENSGALG00000021855	271,814276	1,251787198	0,25254197	4,9567492	7,17E-07	9,57E-06	CCDC39
ENSGALG00000007193	236,114906	1,251769992	0,27435374	4,56261315	5,05E-06	5,59E-05	
ENSGALG00000018557	36,5653316	1,251460959	0,35373058	3,53789302	0,00040333	0,00250291	SOD3
ENSGALG00000025896	117,288757	1,251281202	0,32116119	3,89611589	9,77E-05	0,00073608	PLPPR4
ENSGALG00000012547	219,756071	1,25117298	0,29086976	4,30148871	1,70E-05	0,00016265	
ENSGALG00000013356	85,2230662	1,250931573	0,33961603	3,68337025	0,00023017	0,00154304	IKBKE
ENSGALG00000003404	2332,06775	1,24748878	0,13904188	8,97203598	2,91E-19	2,21E-17	
ENSGALG00000001206	49,0370075	1,247009104	0,35307655	3,53183782	0,00041268	0,00255627	
ENSGALG00000028496	22,1201797	1,24376929	0,33591389	3,70264323	0,00021336	0,00144525	
ENSGALG00000007065	27,8385164	1,239888336	0,34784058	3,5645304	0,00036451	0,00229546	GJD4
ENSGALG00000015345	537,385222	1,237723953	0,20004279	6,18729599	6,12E-10	1,49E-08	
ENSGALG00000028448	68,098086	1,236909029	0,34651471	3,5695715	0,00035757	0,00225696	SH2D1B
ENSGALG00000001564	906,154794	1,234344815	0,19698595	6,26615653	3,70E-10	9,23E-09	
ENSGALG00000023036	562,73194	1,234170444	0,20582667	5,99616393	2,02E-09	4,57E-08	FRMPD1
ENSGALG00000010927	22,2125847	1,233723332	0,33533284	3,67910081	0,00023406	0,00156441	
ENSGALG00000028681	168,618009	1,232657979	0,29055151	4,2424766	2,21E-05	0,00020433	
ENSGALG00000015573	43,775019	1,232214926	0,3535094	3,48566378	0,00049092	0,00295484	FHL5
ENSGALG00000005298	800,818564	1,231219861	0,17189685	7,16255038	7,92E-13	2,92E-11	ST6GALNAC6
ENSGALG00000016112	539,87209	1,228482119	0,20939423	5,86683837	4,44E-09	9,46E-08	
ENSGALG00000000107	237,729231	1,227291307	0,27709903	4,42907111	9,46E-06	9,81E-05	TRIM7
ENSGALG00000002086	83,650156	1,225708617	0,33362046	3,67396119	0,00023882	0,00159076	RASL10B
ENSGALG00000002667	699,199625	1,225466735	0,17683961	6,92982057	4,21E-12	1,40E-10	OLFML2B

ENSGALG00000013431	35,4490725	1,223926576	0,35351054	3,46220673	0,00053577	0,00317564	
ENSGALG00000028031	210,360934	1,22342747	0,2837755	4,31125118	1,62E-05	0,00015642	C1QL1
ENSGALG00000012019	150,440096	1,222353012	0,29815258	4,09975655	4,14E-05	0,00035053	
ENSGALG00000000048	677,127073	1,221920851	0,19907606	6,13795972	8,36E-10	2,00E-08	
ENSGALG00000028946	391,056645	1,217039733	0,25108871	4,84705086	1,25E-06	1,59E-05	
ENSGALG00000014407	118,55781	1,216198172	0,31990769	3,80171597	0,0001437	0,00102593	
ENSGALG00000012658	2563,96357	1,214376265	0,15238934	7,96890584	1,60E-15	8,19E-14	TCP11L2
ENSGALG00000028402	33,811586	1,211737057	0,34956854	3,46637901	0,00052752	0,00313769	
ENSGALG00000003345	1065,51677	1,211546204	0,16093702	7,52807643	5,15E-14	2,19E-12	
ENSGALG00000005083	47,8682724	1,210240985	0,35126406	3,44538807	0,00057024	0,00334551	SLC5A10
ENSGALG00000003803	110,857566	1,208011558	0,33977968	3,55527901	0,00037758	0,0023603	DAB2
ENSGALG00000026578	55,333978	1,20748664	0,35388721	3,41206638	0,00064472	0,00370533	
ENSGALG00000014126	18,9923327	1,207239433	0,3279747	3,6808919	0,00023242	0,00155583	
ENSGALG00000015764	5967,75227	1,20416113	0,19086925	6,30882741	2,81E-10	7,13E-09	
ENSGALG00000015528	170,233046	1,204136401	0,30064358	4,00519575	6,20E-05	0,00049428	JAKMIP1
ENSGALG00000002355	234,006388	1,203134529	0,25489167	4,72017987	2,36E-06	2,80E-05	
ENSGALG00000004641	146,280836	1,20257315	0,29654538	4,05527534	5,01E-05	0,0004122	
ENSGALG00000026903	111,361959	1,200671289	0,32831415	3,65708057	0,0002551	0,00168686	CALB2
ENSGALG00000004745	1802,35745	1,199713581	0,14187699	8,45601213	2,77E-17	1,74E-15	NATD1
ENSGALG00000013890	33,5607486	1,1996045	0,35201412	3,40783067	0,00065482	0,00374908	
ENSGALG00000019280	990,724576	1,199070585	0,17339981	6,91506298	4,68E-12	1,54E-10	
ENSGALG00000004066	55,6194601	1,199060923	0,35390165	3,38811906	0,00070374	0,00399391	HTR6
ENSGALG00000002082	57,1027996	1,198569946	0,34848133	3,43940933	0,00058299	0,00340658	AMPD1
ENSGALG00000013090	44,7148094	1,197838739	0,35381435	3,38550073	0,00071049	0,00402652	LOXL4
ENSGALG00000014696	222,596022	1,197793931	0,25975439	4,61125584	4,00E-06	4,51E-05	
ENSGALG00000006922	19,3844584	1,194740346	0,3271281	3,65220948	0,00025999	0,0017142	
ENSGALG00000011489	4656,68128	1,193784841	0,12166568	9,8120096	1,00E-22	9,93E-21	
ENSGALG00000016590	18,8906226	1,192009768	0,32708105	3,64438646	0,00026803	0,00175697	TLR7
ENSGALG00000002407	144,980361	1,191296249	0,30414176	3,9169112	8,97E-05	0,00068373	HYDIN
ENSGALG00000012890	273,965985	1,191168201	0,26407391	4,51073784	6,46E-06	6,90E-05	DGKI
ENSGALG00000027218	239,44089	1,190798556	0,27577811	4,317959	1,57E-05	0,00015239	
ENSGALG00000017040	33,0805894	1,190245244	0,3523261	3,37824885	0,00072949	0,00411775	
ENSGALG00000008362	175,023576	1,190011885	0,27541961	4,32072321	1,56E-05	0,00015103	SSTR4
ENSGALG00000004521	401,353263	1,187877806	0,21973208	5,40602807	6,44E-08	1,08E-06	GPX3
ENSGALG00000006074	272,240776	1,186152058	0,28359005	4,18262934	2,88E-05	0,00025631	SOX6
ENSGALG00000003842	39,7811882	1,185854219	0,35383776	3,35140662	0,00080402	0,00446624	GHRH
ENSGALG00000021882	58,6974367	1,185518376	0,34682245	3,41822852	0,0006303	0,00363471	
ENSGALG00000002744	252,461162	1,184999187	0,25185371	4,70510904	2,54E-06	3,00E-05	PCDH15
ENSGALG00000006768	37,969199	1,18499895	0,35365822	3,35068965	0,00080611	0,00447275	
ENSGALG00000014812	711,71818	1,183271769	0,18701629	6,32710549	2,50E-10	6,39E-09	SIDT1
ENSGALG00000017331	630,733948	1,182854332	0,18865205	6,27003173	3,61E-10	9,05E-09	
ENSGALG00000005547	144,897217	1,180323703	0,29897868	3,94785238	7,89E-05	0,00060932	PIK3AP1
ENSGALG00000017291	662,461807	1,180080414	0,20904403	5,64512838	1,65E-08	3,14E-07	FAR2
ENSGALG00000006308	28,0784724	1,179461013	0,33709473	3,4989008	0,00046718	0,00283376	
ENSGALG00000013112	320,804038	1,179282262	0,23305967	5,06000147	4,19E-07	5,90E-06	RERGL
ENSGALG00000007333	325,518561	1,178440858	0,25844246	4,55978035	5,12E-06	5,65E-05	UBAP1L
ENSGALG00000001021	191,271341	1,17756894	0,28381375	4,14909056	3,34E-05	0,00029114	MYOCD
ENSGALG00000003821	322,044025	1,177115475	0,23475754	5,01417543	5,33E-07	7,38E-06	RAMP1
ENSGALG00000003164	243,863169	1,17697714	0,25907141	4,54306075	5,54E-06	6,04E-05	
ENSGALG00000016651	946,900962	1,174964335	0,18486316	6,35585995	2,07E-10	5,41E-09	
ENSGALG00000002435	70,1914893	1,174947125	0,34690999	3,38689333	0,00070689	0,00400845	
ENSGALG00000001331	1782,64917	1,174179888	0,1437468	8,16839003	3,13E-16	1,75E-14	ST14
ENSGALG00000009114	216,592643	1,170782693	0,26080261	4,48915246	7,15E-06	7,60E-05	
ENSGALG00000028580	59,1862765	1,170297276	0,34946527	3,34882286	0,00081156	0,00449343	
ENSGALG00000006099	3185,42607	1,169037408	0,1372144	8,51978685	1,60E-17	1,03E-15	ZFPM1
ENSGALG00000014261	23192,4244	1,166708788	0,14807674	7,87908222	3,30E-15	1,59E-13	
ENSGALG00000006627	44,7235286	1,164076921	0,35292296	3,29838819	0,00097242	0,00521033	UPB1
ENSGALG00000012668	622,845457	1,1628752	0,24480349	4,75023949	2,03E-06	2,44E-05	
ENSGALG00000011446	368,843846	1,161895934	0,22151576	5,24520663	1,56E-07	2,42E-06	TNFAIP2
ENSGALG00000005630	38,0970872	1,161740711	0,34283622	3,38861721	0,00070246	0,00398832	CRYBB1
ENSGALG00000023879	439,210587	1,160823609	0,21167651	5,48395108	4,16E-08	7,24E-07	
ENSGALG00000007596	99,2163667	1,160341621	0,32378836	3,58364221	0,00033884	0,00216077	

ENSGALG00000005721	236,127639	1,159323873	0,25145197	4,61051812	4,02E-06	4,52E-05	DGKG
ENSGALG00000013911	546,725872	1,159209839	0,2024827	5,72498212	1,03E-08	2,03E-07	
ENSGALG00000011418	18,2422739	1,158885244	0,32516535	3,56398753	0,00036526	0,00229703	CCR6
ENSGALG00000028489	20,5369622	1,158414063	0,33177044	3,4916132	0,00048011	0,00290006	
ENSGALG00000001031	327,10314	1,156740289	0,24403187	4,74011969	2,14E-06	2,56E-05	HSF5
ENSGALG00000002016	953,709981	1,156233761	0,16494826	7,0096752	2,39E-12	8,30E-11	PRKCD
ENSGALG00000001095	18,2554746	1,156162616	0,32498658	3,55757036	0,0003743	0,00234627	
ENSGALG00000013980	288,264915	1,155390063	0,2983628	3,87243334	0,00010775	0,00079949	SLC2A12
ENSGALG00000026241	639,218946	1,154849081	0,23028217	5,01493056	5,31E-07	7,36E-06	GRIN3B
ENSGALG00000006208	100,690133	1,154488936	0,32195187	3,58590535	0,00033591	0,00214413	OTOG
ENSGALG00000009641	18352,4117	1,153845642	0,14589909	7,90851867	2,60E-15	1,28E-13	COL1A2
ENSGALG00000012288	52,9340022	1,152071153	0,35012037	3,29050023	0,00100009	0,00532927	
ENSGALG00000015932	523,705524	1,152042382	0,22404186	5,14208532	2,72E-07	3,98E-06	
ENSGALG00000003789	56,0437149	1,151405842	0,3500328	3,28942274	0,00100393	0,00534762	FAM78A
ENSGALG00000010365	313,078692	1,151056068	0,26208135	4,39198002	1,12E-05	0,0001143	
ENSGALG00000017691	74,6333149	1,149429932	0,34278483	3,35321124	0,0007988	0,00444266	
ENSGALG00000017374	478,615413	1,149174538	0,22225681	5,17048077	2,33E-07	3,48E-06	
ENSGALG00000028710	21,1011731	1,148265888	0,3321095	3,45749187	0,00054523	0,00322331	
ENSGALG00000012374	515,33143	1,147327656	0,19490406	5,88662778	3,94E-09	8,55E-08	FRMD6
ENSGALG00000016328	5259,3238	1,146952103	0,14040122	8,16910377	3,11E-16	1,75E-14	
ENSGALG00000025721	19,2431462	1,146921853	0,32414703	3,53827662	0,00040275	0,00250042	
ENSGALG00000012365	9658,53942	1,146353139	0,11013775	10,4083585	2,27E-25	2,97E-23	PYGL
ENSGALG00000005540	392,975435	1,145405784	0,21951203	5,21796369	1,81E-07	2,76E-06	MICAL2
ENSGALG00000028702	48,7217309	1,145044679	0,35222211	3,25091653	0,00115034	0,0060053	
ENSGALG00000022819	93,4187666	1,14482978	0,33650375	3,40213085	0,00066863	0,00382494	PCP4
ENSGALG00000001968	55,5701756	1,14394352	0,35227777	3,2472771	0,00116515	0,00606403	SYT6
ENSGALG00000012505	1173,09399	1,142096363	0,156235	7,31011862	2,67E-13	1,05E-11	LRFN5
ENSGALG00000015758	1198,98398	1,141437782	0,17077903	6,68371174	2,33E-11	6,96E-10	
ENSGALG00000003876	41,5449157	1,140755556	0,34478607	3,30858949	0,00093767	0,00507207	
ENSGALG00000009791	992,573884	1,140657464	0,21268775	5,3630614	8,18E-08	1,34E-06	PROX1
ENSGALG00000028130	1683,17579	1,140619832	0,15899173	7,17408269	7,28E-13	2,72E-11	
ENSGALG00000010673	22,7777978	1,139845847	0,34030069	3,34952553	0,0008095	0,00448387	SLC1A7
ENSGALG00000006436	58,7768458	1,138634151	0,35302664	3,22534908	0,00125819	0,00646426	
ENSGALG00000000620	143,16144	1,137872957	0,29367405	3,8746119	0,00010679	0,00079541	
ENSGALG00000013818	524,978654	1,136933045	0,21089775	5,39092064	7,01E-08	1,17E-06	CITED2
ENSGALG00000007236	26,5853166	1,136243459	0,34408993	3,30217003	0,0009594	0,00516702	
ENSGALG00000023643	36,5223482	1,136155947	0,35390776	3,2103166	0,00132589	0,00675092	
ENSGALG00000007129	549,585731	1,134513156	0,27598889	4,11072037	3,94E-05	0,00033711	
ENSGALG00000001442	382,78952	1,131898366	0,21564935	5,24879104	1,53E-07	2,37E-06	OPCML
ENSGALG00000000217	558,865853	1,131400306	0,21968434	5,15011819	2,60E-07	3,84E-06	PPFIA4
ENSGALG00000000911	18,8021132	1,131377385	0,32322345	3,50029482	0,00046474	0,00282099	IL19
ENSGALG00000003028	252,863378	1,131167716	0,25365391	4,45949245	8,22E-06	8,64E-05	
ENSGALG00000027626	26,2635541	1,129092221	0,34723307	3,251684	0,00114723	0,00599848	
ENSGALG00000008063	112,836606	1,129089778	0,31997995	3,52862665	0,00041772	0,00258161	NOX5
ENSGALG00000016592	75,5093263	1,127895523	0,34582316	3,26148062	0,00110832	0,00582167	PRPS2
ENSGALG00000021529	183,029289	1,127864143	0,27306603	4,13037145	3,62E-05	0,00031229	GP1BB
ENSGALG00000006503	1322,53017	1,127315674	0,17398315	6,47945327	9,21E-11	2,52E-09	VDAC1
ENSGALG00000026333	170,514796	1,126889713	0,28096298	4,01081206	6,05E-05	0,00048433	
ENSGALG00000015348	3709,65535	1,126202791	0,16010935	7,03395999	2,01E-12	7,08E-11	ALCAM
ENSGALG00000027872	562,415563	1,125834202	0,2065498	5,45066721	5,02E-08	8,57E-07	
ENSGALG00000007964	712,005282	1,125506336	0,19390731	5,80435241	6,46E-09	1,32E-07	GATSL3
ENSGALG00000008940	122,073119	1,125469701	0,30445466	3,69667421	0,00021844	0,00147524	SPTBN5
ENSGALG00000012252	31,0664734	1,124245419	0,34638552	3,24564781	0,00117184	0,00608953	
ENSGALG00000009237	283,646852	1,124183808	0,25265321	4,44951324	8,61E-06	9,03E-05	
ENSGALG00000008629	362,430604	1,122527446	0,28746439	3,90492695	9,43E-05	0,00071253	
ENSGALG00000004649	115,318908	1,122312882	0,33908753	3,30980286	0,00093362	0,00506019	TRPV1
ENSGALG00000016821	91,586414	1,121951764	0,32755694	3,42521141	0,00061432	0,00356825	
ENSGALG00000000994	36,3375502	1,120119947	0,35335281	3,1699761	0,00152451	0,00753677	
ENSGALG00000009943	1812,23536	1,119992507	0,14209817	7,8818219	3,23E-15	1,56E-13	FAM117A
ENSGALG00000028795	3146,83309	1,118039364	0,16042604	6,96918862	3,19E-12	1,09E-10	
ENSGALG00000026758	74,5546131	1,11741437	0,33538362	3,33174999	0,00086302	0,0047277	
ENSGALG00000001204	1443,12547	1,117076992	0,14668872	7,61528921	2,63E-14	1,19E-12	ENKD1

ENSGALG00000012055	30,6319383	1,115864912	0,34954847	3,19230382	0,00141143	0,00709885	
ENSGALG00000015469	3626,20782	1,115704157	0,1321025	8,44574565	3,02E-17	1,89E-15	
ENSGALG00000009963	76,7800387	1,114244364	0,33758559	3,30062774	0,00096469	0,00518524	LYZ
ENSGALG00000011414	712,898703	1,112714675	0,17966211	6,19337424	5,89E-10	1,44E-08	AMDHD1
ENSGALG00000015454	87,0613625	1,111985108	0,33768276	3,29298749	0,00099129	0,00529478	
ENSGALG00000010233	149,38519	1,111330421	0,289789	3,83496413	0,00012558	0,00091141	SYNDIG1L
ENSGALG00000024411	95,5715091	1,110994905	0,32259096	3,44397412	0,00057323	0,00335823	
ENSGALG00000003016	111,975898	1,109545556	0,31007137	3,57835536	0,00034576	0,00219771	MASP2
ENSGALG00000006792	447,856144	1,109047707	0,21887998	5,06692165	4,04E-07	5,71E-06	VSTM2L
ENSGALG00000009823	207,863068	1,108978484	0,26701827	4,15319326	3,28E-05	0,00028633	PTPRE
ENSGALG00000027501	1178,23042	1,108782799	0,15555529	7,12790156	1,02E-12	3,72E-11	ANPEP
ENSGALG00000004017	335,243952	1,108435951	0,22759443	4,87022448	1,11E-06	1,44E-05	TOR1AIP2
ENSGALG00000025912	62,6339712	1,108425226	0,34376932	3,22432853	0,00126268	0,00648	
ENSGALG00000000158	209,106145	1,107475494	0,2643096	4,19006906	2,79E-05	0,00024946	
ENSGALG00000002610	604,590631	1,105862871	0,18967796	5,83021282	5,54E-09	1,15E-07	
ENSGALG00000023621	702,550009	1,105773477	0,19719495	5,60751405	2,05E-08	3,82E-07	SFXN3
ENSGALG00000009574	24,9756977	1,105678443	0,34380548	3,21600002	0,00129991	0,00664829	
ENSGALG00000006000	170,909865	1,105166021	0,29241804	3,77940435	0,0001572	0,00111071	
ENSGALG00000001011	623,472513	1,104831537	0,2206424	5,00733998	5,52E-07	7,61E-06	
ENSGALG00000011473	347,60043	1,104703027	0,22754114	4,85495953	1,20E-06	1,54E-05	RPS6KA2
ENSGALG00000026834	233,708518	1,10373431	0,25983013	4,24790728	2,16E-05	0,00020012	
ENSGALG00000006003	58,9954831	1,10329195	0,3504724	3,14801378	0,00164384	0,00802229	
ENSGALG00000001281	270,657774	1,101845994	0,24895157	4,42594511	9,60E-06	9,94E-05	
ENSGALG00000006407	926,885262	1,100961832	0,17822617	6,17732963	6,52E-10	1,58E-08	
ENSGALG00000011003	180,050652	1,099169303	0,28561474	3,84843338	0,00011888	0,00086689	SLC35F3
ENSGALG00000000168	20,2372176	1,099055711	0,33636253	3,26747371	0,00108512	0,00571524	ADORA1
ENSGALG00000014798	1444,6552	1,098754437	0,18532469	5,92880749	3,05E-09	6,69E-08	SERINC5
ENSGALG00000015076	118,818737	1,094512369	0,30475359	3,59146666	0,00032882	0,00210678	
ENSGALG00000002579	438,862484	1,09231668	0,24079762	4,53624361	5,73E-06	6,21E-05	RIMBP2
ENSGALG00000027102	27,9313615	1,090659115	0,34855662	3,12907298	0,00175359	0,00846159	
ENSGALG00000007740	159,049731	1,089704597	0,29794304	3,6574259	0,00025476	0,00168541	
ENSGALG00000004972	200,694244	1,089536909	0,30285765	3,59752149	0,00032126	0,00206127	
ENSGALG00000004538	131,202356	1,088596778	0,30030855	3,62492773	0,00028904	0,00188202	TNN
ENSGALG00000005470	915,927743	1,087631823	0,1770332	6,14366031	8,06E-10	1,93E-08	PLPPR5
ENSGALG00000006189	81,0476646	1,08750661	0,33966232	3,20172876	0,00136606	0,00691664	CRYGN
ENSGALG00000001716	219,086022	1,084481493	0,27662697	3,92037508	8,84E-05	0,00067587	C15orf59
ENSGALG00000017106	609,784044	1,082115941	0,19278194	5,61316028	1,99E-08	3,72E-07	ATP8A2
ENSGALG00000014264	24,119483	1,081789708	0,34218632	3,16140549	0,0015701	0,00773684	NSUN7
ENSGALG00000001470	804,498578	1,081645785	0,18189855	5,94642334	2,74E-09	6,10E-08	STOML1
ENSGALG00000023636	80,78387	1,079731402	0,33574779	3,21590026	0,00130036	0,00664829	
ENSGALG00000021303	70,1790621	1,078464744	0,33870785	3,18405595	0,00145227	0,00725779	
ENSGALG00000011353	167,024074	1,077984355	0,29480885	3,65655358	0,00025563	0,00168869	
ENSGALG00000026651	469,211499	1,076820994	0,26273591	4,09849186	4,16E-05	0,00035167	SCARA3
ENSGALG00000005031	222,866745	1,076697936	0,27611031	3,89952093	9,64E-05	0,00072701	DRP2
ENSGALG00000005076	658,308514	1,075685444	0,20135322	5,34228092	9,18E-08	1,49E-06	
ENSGALG00000013180	23,7835918	1,075255786	0,33793034	3,18188592	0,00146319	0,0072892	SLC6A18
ENSGALG00000004043	1743,12209	1,074636101	0,18109836	5,93399142	2,96E-09	6,52E-08	
ENSGALG00000000420	275,786063	1,072751202	0,24334062	4,40843449	1,04E-05	0,00010699	
ENSGALG00000003640	1881,52679	1,071788322	0,14728433	7,2770018	3,41E-13	1,33E-11	
ENSGALG00000009355	164,050555	1,070935457	0,2815733	3,80339851	0,00014272	0,00102045	CLDN11
ENSGALG00000026748	19,4954498	1,069944148	0,32787916	3,26322706	0,00110151	0,00578815	
ENSGALG00000004406	128,12145	1,066534863	0,31159618	3,42281114	0,00061977	0,00358919	
ENSGALG00000006370	31,1681716	1,066397762	0,34558448	3,08578021	0,00203019	0,00956436	
ENSGALG00000012377	68,8818144	1,065846022	0,33849137	3,14881303	0,00163935	0,00801428	HNMT
ENSGALG00000010752	133,532542	1,063752645	0,31701468	3,35553118	0,00079213	0,00441384	CDCP2
ENSGALG00000021049	25,1605315	1,063749147	0,33499236	3,17544297	0,00149608	0,00741774	
ENSGALG00000006374	154,22163	1,06372742	0,34611406	3,07334361	0,00211675	0,00991383	TBX6
ENSGALG00000007780	1769,76781	1,063721106	0,16415536	6,47996553	9,17E-11	2,52E-09	EIF4E3
ENSGALG00000021542	348,907918	1,061917275	0,24470276	4,3396212	1,43E-05	0,00014102	SNCB
ENSGALG00000002055	5059,12603	1,061032342	0,12896244	8,22745271	1,91E-16	1,10E-14	hairy1
ENSGALG00000028742	318,003255	1,06018982	0,24476244	4,33150539	1,48E-05	0,0001453	
ENSGALG00000001040	379,37277	1,060173693	0,22651608	4,68034629	2,86E-06	3,34E-05	

ENSGALG00000008734	1277,14965	1,060092794	0,17848485	5,93939919	2,86E-09	6,33E-08	PCSK2
ENSGALG00000025771	418,454799	1,059868939	0,21305921	4,97452772	6,54E-07	8,82E-06	
ENSGALG00000017311	141,945952	1,059578058	0,29313349	3,61466049	0,00030074	0,00194647	
ENSGALG00000002554	211,299245	1,058725863	0,27265415	3,88303593	0,00010316	0,00077214	TSPAN2
ENSGALG00000011898	1941,78412	1,056447793	0,1400944	7,54097066	4,66E-14	2,00E-12	FAM109B
ENSGALG00000012545	20,3956262	1,051934631	0,33312924	3,15773734	0,00158999	0,00780659	CYTIP
ENSGALG00000010497	795,792907	1,048579632	0,17878894	5,86490213	4,49E-09	9,56E-08	SLC29A4
ENSGALG00000000503	90,5216838	1,0483641	0,32883068	3,18815785	0,00143182	0,00718021	
ENSGALG00000007959	24,9928179	1,04699816	0,3339903	3,13481605	0,00171962	0,00832534	SLC26A3
ENSGALG00000012045	94,1610251	1,046487546	0,33468277	3,1268044	0,00176717	0,00850781	
ENSGALG00000009340	441,508564	1,044750001	0,25059513	4,16907546	3,06E-05	0,00027021	
ENSGALG00000007617	161,132474	1,044420437	0,30323964	3,44420813	0,00057273	0,00335677	
ENSGALG00000007837	153,44217	1,044277791	0,31727304	3,29141673	0,00099684	0,0053161	KAZALD1
ENSGALG00000016020	82,6868728	1,041815502	0,33102199	3,14726979	0,00164803	0,0080365	
ENSGALG00000016608	1071,03802	1,041630113	0,16148809	6,45019776	1,12E-10	3,01E-09	ADCY3
ENSGALG00000014258	1564,45283	1,040436704	0,17049678	6,10238319	1,04E-09	2,47E-08	LIMCH1
ENSGALG00000027096	1851,9247	1,040113834	0,13778609	7,54875793	4,39E-14	1,90E-12	LGALSL
ENSGALG00000024369	197,407512	1,038738473	0,26515652	3,91745396	8,95E-05	0,00068257	
ENSGALG00000028234	617,735502	1,03829388	0,19109872	5,43328543	5,53E-08	9,37E-07	
ENSGALG00000007219	1265,62807	1,038287687	0,15486967	6,70426727	2,02E-11	6,12E-10	
ENSGALG00000007253	1268,2995	1,036667095	0,15805846	6,55875735	5,43E-11	1,54E-09	IL1RAP
ENSGALG00000005460	763,147126	1,036332157	0,18413261	5,6281837	1,82E-08	3,43E-07	SLCO4A1
ENSGALG00000026714	2323,49209	1,035206481	0,15670753	6,60597808	3,95E-11	1,13E-09	
ENSGALG00000024122	36,985836	1,03453695	0,33443583	3,09337955	0,00197891	0,00935579	
ENSGALG00000023779	766,128835	1,033222085	0,19891303	5,19434077	2,05E-07	3,11E-06	
ENSGALG00000028318	1679,83733	1,033139386	0,18498818	5,584894	2,34E-08	4,28E-07	CDKN1A
ENSGALG00000005114	245,746736	1,032666035	0,25457852	4,05637544	4,98E-05	0,00041076	
ENSGALG00000028628	176,658931	1,032210406	0,27931003	3,6955723	0,00021939	0,00148092	
ENSGALG00000011875	520,49686	1,032103241	0,20932071	4,93072682	8,19E-07	1,08E-05	TRPC3
ENSGALG00000001304	107,91968	1,031984172	0,31646483	3,26097581	0,0011103	0,0058298	
ENSGALG00000004860	237,1229	1,03038994	0,25186264	4,09107893	4,29E-05	0,00035997	RASD1
ENSGALG00000010566	1230,63642	1,029946325	0,16433565	6,26733359	3,67E-10	9,18E-09	
ENSGALG00000012791	100,570938	1,028853172	0,32574707	3,15844184	0,00158615	0,00779056	TBXAS1
ENSGALG00000024024	333,882358	1,028182094	0,2283532	4,5025956	6,71E-06	7,16E-05	TGFA
ENSGALG00000009739	1255,18637	1,027863615	0,18332662	5,60673425	2,06E-08	3,83E-07	AMIGO2
ENSGALG00000010078	138,046016	1,027109659	0,2951454	3,48001248	0,00050139	0,00300724	GLP1R
ENSGALG00000003349	284,814958	1,025286613	0,23608456	4,34287875	1,41E-05	0,00013925	PLEKHA2
ENSGALG00000009722	2700,68769	1,024969081	0,12748481	8,03993117	8,99E-16	4,78E-14	DYNC1I1
ENSGALG00000013200	141,95986	1,024952745	0,30040339	3,4119214	0,00064507	0,00370573	GABBR2
ENSGALG00000029169	143,486281	1,024627176	0,29797907	3,43858777	0,00058476	0,00341401	
ENSGALG00000025906	86,1054184	1,019071961	0,32995746	3,08849502	0,00201173	0,00949054	
ENSGALG00000012564	122,738702	1,018929434	0,30201581	3,37376189	0,00074148	0,00416817	
ENSGALG00000009686	819,704721	1,017336386	0,20309235	5,00923036	5,46E-07	7,54E-06	
ENSGALG00000001698	101,577734	1,017252154	0,31962329	3,18265971	0,00145929	0,00727774	GABRA1
ENSGALG00000002991	1547,8463	1,016425478	0,15188757	6,69195943	2,20E-11	6,60E-10	
ENSGALG00000001191	726,030438	1,014738695	0,17607486	5,76310952	8,26E-09	1,66E-07	MEOX1
ENSGALG00000009066	83,1568975	1,013658719	0,32821816	3,08836875	0,00201259	0,00949129	
ENSGALG00000016223	759,19748	1,013131958	0,18257274	5,54919615	2,87E-08	5,19E-07	
ENSGALG00000010203	639,913614	1,012100046	0,1964193	5,15275255	2,57E-07	3,79E-06	
ENSGALG00000008874	19,4465425	1,010524458	0,32352202	3,1235106	0,00178707	0,00857934	SLC13A1
ENSGALG00000022718	221,713706	1,010197798	0,26186126	3,85775962	0,00011443	0,00084033	
ENSGALG00000014455	15,3755596	1,009369671	0,31516038	3,20271755	0,00136137	0,0068955	LPAR5
ENSGALG00000008080	251,329122	1,008867324	0,25119635	4,01624997	5,91E-05	0,00047553	C1QTNF4
ENSGALG00000001951	315,95633	1,007959576	0,24129054	4,17736875	2,95E-05	0,00026123	
ENSGALG00000027712	289,192222	1,006836187	0,24626896	4,08836006	4,34E-05	0,00036327	
ENSGALG00000028186	897,733771	1,005437673	0,18601887	5,40503063	6,48E-08	1,09E-06	RERG
ENSGALG00000003816	91,3783387	1,005135037	0,32711976	3,07268213	0,00212144	0,009926	NT5C1A
ENSGALG00000007713	153,549096	1,002069085	0,29743797	3,36900193	0,00075441	0,00423331	
ENSGALG00000016251	6835,37033	1,000647408	0,15469938	6,46833505	9,91E-11	2,69E-09	
ENSGALG00000023953	637,175356	0,998916427	0,18661623	5,35278435	8,66E-08	1,41E-06	
ENSGALG00000005887	163,632495	0,998359123	0,30751037	3,24658685	0,00116798	0,00607411	
ENSGALG00000012480	541,532617	0,99597856	0,2064035	4,82539577	1,40E-06	1,76E-05	

ENSGALG00000014681	398,451477	0,995892967	0,23114252	4,30856669	1,64E-05	0,0001581	PCSK1
ENSGALG00000017316	218,980953	0,995598244	0,30126263	3,30475185	0,00095061	0,0051298	UCP3
ENSGALG00000001453	365,617866	0,994068241	0,24549624	4,04921983	5,14E-05	0,00042072	
ENSGALG00000002437	722,53208	0,993124174	0,18855357	5,26706645	1,39E-07	2,17E-06	FAM69B
ENSGALG00000012613	121,972608	0,992227725	0,31630972	3,13688661	0,00170752	0,00827328	FBP1
ENSGALG00000028682	130,825911	0,990483134	0,3063312	3,23337336	0,00122338	0,00631397	CBLN2
ENSGALG00000013183	389,466708	0,988585254	0,21644464	4,56738155	4,94E-06	5,48E-05	TERT
ENSGALG00000009638	342,698465	0,988233263	0,2260661	4,3714351	1,23E-05	0,00012439	ANO6
ENSGALG00000016224	196,097983	0,988198095	0,27726141	3,56413865	0,00036505	0,00229676	MAOA
ENSGALG00000009052	3269,15476	0,987910707	0,16241285	6,08271266	1,18E-09	2,78E-08	LBH
ENSGALG00000026042	849,913797	0,987626868	0,23717847	4,16406621	3,13E-05	0,00027514	
ENSGALG00000012536	763,863052	0,987580114	0,21162614	4,66662634	3,06E-06	3,54E-05	GALNT13
ENSGALG00000016599	16,0014025	0,987073109	0,31315435	3,15203387	0,00162137	0,00793209	
ENSGALG00000009463	2838,70473	0,986691121	0,14031176	7,03213428	2,03E-12	7,16E-11	
ENSGALG00000005030	1142,72142	0,984706358	0,17789423	5,53534734	3,11E-08	5,55E-07	DOCK10
ENSGALG00000027429	108,061421	0,983232181	0,31233583	3,14799676	0,00164394	0,00802229	HSD17B1
ENSGALG00000023847	205,80734	0,981888346	0,26827405	3,66001979	0,0002522	0,00166925	
ENSGALG00000012552	632,633121	0,981819434	0,18202425	5,39389345	6,89E-08	1,15E-06	TOM1
ENSGALG00000027739	223,979623	0,981670308	0,28088257	3,49494923	0,00047415	0,00287042	
ENSGALG00000012715	380,224027	0,978917656	0,24539961	3,98907579	6,63E-05	0,00052445	CAP2
ENSGALG00000012709	399,326921	0,978132572	0,259146	3,77444597	0,00016036	0,0011301	ATXN1
ENSGALG00000028301	585,856129	0,976931086	0,20530374	4,75846719	1,95E-06	2,36E-05	
ENSGALG00000006882	2766,70659	0,976472658	0,13081296	7,46464785	8,35E-14	3,47E-12	ST8SIA2
ENSGALG00000024059	15,053333	0,976199065	0,31251247	3,12371231	0,00178585	0,00857649	
ENSGALG00000010797	412,002343	0,975073157	0,21003242	4,64248879	3,44E-06	3,95E-05	
ENSGALG00000000902	273,817902	0,974915117	0,24422686	3,99184233	6,56E-05	0,00051867	KDF1
ENSGALG00000004591	574,278704	0,974905454	0,19696934	4,949529	7,44E-07	9,89E-06	SERPINC1
ENSGALG00000018875	14,7404116	0,973272591	0,3123576	3,11589219	0,00183389	0,00876086	
ENSGALG00000003886	1023,87733	0,971801068	0,17739435	5,47819617	4,30E-08	7,44E-07	SGCD
ENSGALG00000005844	1501,38821	0,969445519	0,15286931	6,34166216	2,27E-10	5,87E-09	
ENSGALG00000016820	787,309833	0,968970008	0,17832491	5,43373323	5,52E-08	9,36E-07	GAS6
ENSGALG00000027287	736,986058	0,968636988	0,18931485	5,11653998	3,11E-07	4,50E-06	
ENSGALG00000003170	1633,73146	0,967045053	0,17416807	5,55236702	2,82E-08	5,10E-07	
ENSGALG00000026870	2578,97026	0,966911139	0,16329066	5,92141112	3,19E-09	6,99E-08	
ENSGALG00000027511	3914,70702	0,966828969	0,15258887	6,33616983	2,36E-10	6,06E-09	
ENSGALG00000001209	151,276779	0,963839438	0,28852453	3,34058053	0,00083603	0,00460089	
ENSGALG00000012681	687,549661	0,96179459	0,19337383	4,97375784	6,57E-07	8,84E-06	MBOAT1
ENSGALG00000026914	303,146434	0,960831377	0,2341299	4,10383883	4,06E-05	0,00034533	DOK3
ENSGALG00000016597	546,580903	0,96044418	0,2115615	4,53978708	5,63E-06	6,12E-05	
ENSGALG00000028041	172,712765	0,96010795	0,29281701	3,27886677	0,00104225	0,00552416	DKK2
ENSGALG00000008317	155,968503	0,958791686	0,29825236	3,21469944	0,00130581	0,00666615	SDSL
ENSGALG00000006192	588,22456	0,958606154	0,19869528	4,82450397	1,40E-06	1,77E-05	
ENSGALG00000009896	1725,9341	0,956710434	0,138947	6,88543432	5,76E-12	1,88E-10	TRERF1
ENSGALG00000008936	346,610654	0,956545148	0,23935184	3,99639774	6,43E-05	0,00051087	
ENSGALG00000012577	1716,87461	0,956309243	0,13789127	6,93524147	4,06E-12	1,35E-10	FRMD3
ENSGALG00000016945	3627,68031	0,953551228	0,1270066	7,5078871	6,01E-14	2,53E-12	LECT1
ENSGALG00000014843	607,093989	0,953307805	0,21113346	4,51519047	6,33E-06	6,80E-05	TPD52L1
ENSGALG00000029150	2513,11501	0,953288378	0,14860816	6,41477801	1,41E-10	3,76E-09	AK1
ENSGALG00000000295	158,361127	0,951838119	0,2874721	3,31106259	0,00092942	0,00504148	
ENSGALG00000002008	770,021515	0,951550674	0,26369144	3,60857625	0,00030788	0,00198664	RNF157
ENSGALG00000017926	117,345158	0,950771836	0,30495213	3,11777407	0,00182222	0,00871735	7SK
ENSGALG00000009123	138,335079	0,94996493	0,29437793	3,22702502	0,00125085	0,00643402	
ENSGALG00000011046	918,666805	0,946061534	0,22454083	4,21331632	2,52E-05	0,00022872	SNX10
ENSGALG00000004792	282,665704	0,945718297	0,26094655	3,62418393	0,00028988	0,00188564	
ENSGALG00000006988	545,963197	0,944077449	0,23809277	3,96516647	7,33E-05	0,00057357	
ENSGALG00000009002	180,825204	0,943061124	0,27453332	3,43514266	0,00059224	0,00345327	
ENSGALG00000003370	6860,89746	0,941055859	0,11588753	8,12042401	4,65E-16	2,56E-14	KIAA1191
ENSGALG00000015183	541,002513	0,940741895	0,24692948	3,80975932	0,0001391	0,00099941	RAB13
ENSGALG00000010977	629,762637	0,94045823	0,18538133	5,07310108	3,91E-07	5,55E-06	FAM221A
ENSGALG00000017169	2527,40437	0,94013642	0,1550564	6,06319017	1,33E-09	3,11E-08	ELMOD1
ENSGALG00000006351	260,931586	0,940035694	0,25660603	3,66334215	0,00024895	0,00165256	FAS
ENSGALG00000007415	599,165563	0,938743099	0,23705941	3,95994876	7,50E-05	0,0005829	

ENSGALG00000002678	150,077896	0,938682309	0,29361496	3,19698392	0,00138873	0,00700797	CSPG4
ENSGALG00000003832	1422,76359	0,938218604	0,14754036	6,35906414	2,03E-10	5,31E-09	TEX264
ENSGALG00000009725	763,677984	0,938023358	0,22425701	4,182805	2,88E-05	0,00025631	SCG5
ENSGALG00000015967	204,249989	0,936813576	0,2634911	3,55538981	0,00037742	0,0023603	LRRTM1
ENSGALG00000002473	480,317696	0,93617222	0,20261658	4,62041265	3,83E-06	4,35E-05	GPR157
ENSGALG00000015926	1110,05542	0,934566237	0,15987762	5,84550999	5,05E-09	1,06E-07	
ENSGALG00000001398	801,871514	0,933503925	0,17565694	5,31435844	1,07E-07	1,71E-06	
ENSGALG00000028292	453,150865	0,931661788	0,25026444	3,72270944	0,0001971	0,00134712	
ENSGALG00000008735	158,650282	0,931477748	0,29513805	3,15607472	0,00159908	0,00784557	
ENSGALG00000010139	381,674313	0,931031468	0,2175137	4,28033488	1,87E-05	0,00017632	SSTR1
ENSGALG00000002843	586,866611	0,930974563	0,19321147	4,81842275	1,45E-06	1,82E-05	RILP
ENSGALG00000023626	1834,26904	0,929476271	0,16548518	5,61667362	1,95E-08	3,65E-07	NTN1
ENSGALG00000028091	182,303874	0,927849672	0,28991773	3,20038958	0,00137242	0,00694328	MYF5
ENSGALG00000023517	259,64579	0,925400404	0,25687314	3,60255801	0,0003151	0,00202459	AGPAT2
ENSGALG00000005626	609,645166	0,92496767	0,21590969	4,2840488	1,84E-05	0,00017352	TPST2
ENSGALG00000000311	558,408468	0,92295118	0,21535867	4,2856468	1,82E-05	0,00017251	
ENSGALG00000015207	1291,12938	0,92039826	0,14720064	6,25267856	4,03E-10	1,00E-08	
ENSGALG00000023936	805,049393	0,918539777	0,20126409	4,56385321	5,02E-06	5,56E-05	
ENSGALG00000011709	513,362717	0,918333355	0,23178437	3,96201584	7,43E-05	0,0005792	
ENSGALG00000027622	398,876868	0,916634803	0,26586468	3,44774944	0,00056528	0,00332452	
ENSGALG00000015795	539,939541	0,91628777	0,2153216	4,25543833	2,09E-05	0,00019456	ADAMTS5
ENSGALG00000005120	2531,22001	0,916224882	0,15496532	5,91245108	3,37E-09	7,37E-08	
ENSGALG00000013071	1399,47707	0,914850391	0,17423001	5,25081979	1,51E-07	2,35E-06	
ENSGALG00000010382	346,13588	0,914779385	0,22801207	4,01197789	6,02E-05	0,00048223	
ENSGALG00000004309	938,335478	0,913060877	0,18553484	4,92123686	8,60E-07	1,13E-05	GDAP1L1
ENSGALG00000028622	281,675272	0,912974481	0,25598956	3,56645202	0,00036185	0,00228081	PEAR1
ENSGALG00000005331	677,48087	0,912514017	0,17989387	5,07251308	3,93E-07	5,56E-06	GJC2
ENSGALG00000027462	1468,67221	0,912376278	0,14323943	6,36958898	1,90E-10	4,98E-09	P3H4
ENSGALG00000015789	559,929943	0,912066241	0,21238691	4,29436183	1,75E-05	0,00016704	
ENSGALG00000010551	919,316948	0,911984879	0,18322857	4,97730716	6,45E-07	8,70E-06	CDC42EP3
ENSGALG00000012298	550,04797	0,911513817	0,22249482	4,09678677	4,19E-05	0,00035361	SLC39A8
ENSGALG00000003588	1398,60382	0,911216657	0,1584852	5,74953779	8,95E-09	1,78E-07	
ENSGALG00000005814	212,002946	0,910795359	0,27185501	3,35029823	0,00080725	0,00447521	
ENSGALG00000002581	512,901786	0,910753526	0,19519764	4,66580184	3,07E-06	3,56E-05	HTATIP2
ENSGALG00000004676	1678,58309	0,910530695	0,14330969	6,35358792	2,10E-10	5,48E-09	
ENSGALG00000023195	260,809859	0,909179266	0,24717134	3,67833616	0,00023476	0,00156833	DLX6
ENSGALG00000015725	901,983472	0,907979483	0,17014105	5,33662782	9,47E-08	1,53E-06	
ENSGALG00000007114	305,818525	0,907573399	0,27679712	3,27883977	0,00104235	0,00552416	APOA1
ENSGALG00000003018	309,190261	0,906093174	0,24885213	3,64109068	0,00027149	0,00177706	
ENSGALG00000013886	841,136602	0,904236576	0,1804703	5,01044535	5,43E-07	7,51E-06	LDLRAD4
ENSGALG00000021442	473,215804	0,903637173	0,22582203	4,00154571	6,29E-05	0,00050105	CARMIL2
ENSGALG00000005014	159,559707	0,903243848	0,28943129	3,12075394	0,00180389	0,00864482	AIPL1
ENSGALG00000002963	189,406665	0,902926335	0,26991157	3,34526731	0,00082203	0,00453545	PID1
ENSGALG00000000836	482,381548	0,902335572	0,20852451	4,32723978	1,51E-05	0,00014747	RFX5
ENSGALG00000011124	436,071233	0,902068067	0,20974945	4,30069344	1,70E-05	0,000163	ARHGAP24
ENSGALG00000010827	4357,48343	0,901311467	0,11466976	7,86006222	3,84E-15	1,84E-13	
ENSGALG00000008461	387,656717	0,900341356	0,24810826	3,62882455	0,00028471	0,0018574	
ENSGALG00000010119	1290,75662	0,900097678	0,1879287	4,78957	1,67E-06	2,06E-05	CDKL2
ENSGALG00000020905	199,737945	0,899806675	0,28234109	3,18694908	0,00143782	0,00720498	KSR2
ENSGALG00000006793	399,969639	0,898163749	0,21225276	4,23157623	2,32E-05	0,0002129	
ENSGALG00000008632	774,98804	0,897411708	0,17277465	5,19411687	2,06E-07	3,11E-06	ACSS1
ENSGALG00000001896	633,555504	0,897398544	0,18298068	4,90433493	9,37E-07	1,23E-05	
ENSGALG00000006083	883,458305	0,896818662	0,18476425	4,85385374	1,21E-06	1,55E-05	
ENSGALG00000007052	507,965349	0,895651499	0,20347805	4,40171068	1,07E-05	0,00010986	PXK
ENSGALG00000015977	788,487319	0,89563673	0,20705245	4,32565141	1,52E-05	0,00014811	SMOX
ENSGALG00000008262	171,789815	0,894529697	0,28118642	3,18126918	0,00146631	0,0072994	RASGRF1
ENSGALG00000000374	1105,84307	0,894239416	0,18770457	4,76407913	1,90E-06	2,30E-05	RNPEP
ENSGALG00000017204	419,662559	0,891856915	0,22247021	4,00888239	6,10E-05	0,00048802	
ENSGALG00000002854	440,265325	0,890941536	0,2420509	3,68080247	0,0002325	0,00155583	
ENSGALG00000005203	773,684672	0,889220424	0,2133641	4,16761968	3,08E-05	0,00027177	OLFM3
ENSGALG00000027603	933,535269	0,889190298	0,16664995	5,33567704	9,52E-08	1,54E-06	
ENSGALG00000003932	494,661014	0,888085177	0,22917231	3,87518533	0,00010654	0,00079397	SDC4

ENSGALG00000010269	1110,19342	0,886301871	0,16512328	5,36751612	7,98E-08	1,31E-06	KCNAB1
ENSGALG00000004093	635,621253	0,885654514	0,20975364	4,2223558	2,42E-05	0,00022106	TBX22
ENSGALG00000015788	630,459367	0,884593035	0,2015792	4,38831513	1,14E-05	0,00011598	
ENSGALG00000004970	368,256584	0,884420332	0,25182556	3,51203558	0,00044469	0,00271743	
ENSGALG00000026152	376,28373	0,884418189	0,23205448	3,8112524	0,00013826	0,00099391	
ENSGALG00000025733	660,61374	0,882587352	0,1955092	4,51430076	6,35E-06	6,83E-05	
ENSGALG00000006008	1270,65336	0,882382705	0,1743852	5,05996316	4,19E-07	5,90E-06	HOMER2
ENSGALG00000027027	1969,08091	0,882215122	0,16312702	5,40814829	6,37E-08	1,07E-06	
ENSGALG00000003270	387,810885	0,881994372	0,21359889	4,12920868	3,64E-05	0,00031348	
ENSGALG00000008349	209,566568	0,879571782	0,26093775	3,37081072	0,00074947	0,00420787	LMCD1
ENSGALG00000009601	8743,38719	0,878730197	0,13764581	6,38399544	1,73E-10	4,59E-09	NELL2
ENSGALG00000027920	386,752165	0,874011674	0,22844437	3,82592791	0,00013028	0,00094249	
ENSGALG00000026059	270,416326	0,873870501	0,23970989	3,64553371	0,00026684	0,00175077	
ENSGALG00000016017	590,769047	0,873175292	0,21544948	4,05280753	5,06E-05	0,00041599	SLC4A11
ENSGALG00000005974	256,914188	0,872920311	0,25210037	3,46259042	0,000535	0,00317388	COL6A1
ENSGALG00000001129	1785,04064	0,871868135	0,15304444	5,69682996	1,22E-08	2,38E-07	
ENSGALG00000009867	212,190543	0,869748432	0,26750592	3,25132407	0,00114869	0,00599972	
ENSGALG00000027364	6291,5882	0,867638443	0,1162491	7,46361451	8,42E-14	3,48E-12	
ENSGALG00000011624	479,035185	0,867273579	0,2018671	4,29626011	1,74E-05	0,00016596	PNLDC1
ENSGALG00000016384	268,592265	0,867172927	0,26856807	3,2288758	0,00124278	0,00640113	MYT1L
ENSGALG00000026172	493,174963	0,865691508	0,21117972	4,09931168	4,14E-05	0,00035078	
ENSGALG00000003895	788,021122	0,861640305	0,25898235	3,32702328	0,00087779	0,00479965	PRDM12
ENSGALG00000012072	201,724233	0,861021311	0,27532993	3,12723472	0,00176459	0,00849837	
ENSGALG00000000953	1344,71246	0,860062109	0,16163754	5,32093044	1,03E-07	1,66E-06	
ENSGALG00000009902	340,496837	0,859561208	0,24909364	3,4507553	0,00055902	0,00329341	CFAP57
ENSGALG00000005655	299,935919	0,858231064	0,25571117	3,3562518	0,00079007	0,0044067	TSPEAR
ENSGALG00000017072	1099,84084	0,857400203	0,18100217	4,73696081	2,17E-06	2,59E-05	N4BP2L1
ENSGALG00000025919	2494,48854	0,857090747	0,142817	6,00132156	1,96E-09	4,45E-08	
ENSGALG00000015768	2710,70193	0,855142896	0,12974855	6,59077029	4,38E-11	1,25E-09	
ENSGALG00000010664	712,177105	0,852579615	0,22881793	3,72601745	0,00019453	0,00133284	KIF26B
ENSGALG00000001189	452,192377	0,851541498	0,20637637	4,12615796	3,69E-05	0,00031686	NCF1
ENSGALG00000009536	446,627716	0,851130737	0,22757849	3,73994373	0,00018406	0,00127273	
ENSGALG00000003612	268,623077	0,850682606	0,26697919	3,18632551	0,00144092	0,00721787	
ENSGALG00000023172	1255,76162	0,849498162	0,15375274	5,52509283	3,29E-08	5,85E-07	
ENSGALG00000026671	2116,56921	0,847898773	0,14258676	5,94654652	2,74E-09	6,10E-08	
ENSGALG00000002742	2787,33871	0,847416181	0,14490214	5,84819627	4,97E-09	1,04E-07	TMEM132B
ENSGALG00000005791	520,608854	0,84592391	0,20398465	4,14699797	3,37E-05	0,00029344	
ENSGALG00000009603	307,914805	0,844955114	0,23876567	3,53884673	0,00040188	0,00249616	
ENSGALG00000006379	1670,52626	0,844378171	0,18274684	4,62048036	3,83E-06	4,35E-05	SHH
ENSGALG00000005895	481,788237	0,844174027	0,20498441	4,11823536	3,82E-05	0,00032712	
ENSGALG00000004593	2963,70805	0,839985137	0,15590672	5,38774176	7,13E-08	1,18E-06	SULF2
ENSGALG00000010857	11562,2517	0,839106931	0,11368089	7,38124899	1,57E-13	6,34E-12	Dab1
ENSGALG00000026505	1579,97582	0,837091894	0,15268322	5,48254005	4,19E-08	7,29E-07	
ENSGALG00000003354	276,830006	0,837083394	0,24777884	3,37834895	0,00072922	0,00411775	KCNH4
ENSGALG00000011205	1172,74221	0,836341019	0,20119253	4,15691891	3,23E-05	0,00028261	SMOC2
ENSGALG00000005161	2738,71244	0,836184904	0,13858143	6,03388843	1,60E-09	3,70E-08	
ENSGALG00000006014	961,400691	0,836085862	0,15943435	5,24407593	1,57E-07	2,43E-06	
ENSGALG00000007770	193,517852	0,835610241	0,26849664	3,11218139	0,0018571	0,00885001	
ENSGALG00000028582	1565,14033	0,832821984	0,15076847	5,52384707	3,32E-08	5,87E-07	
ENSGALG00000005481	1783,10991	0,83266607	0,14481077	5,75002865	8,92E-09	1,77E-07	ARHGEF3
ENSGALG00000015660	2889,81934	0,831570906	0,12282495	6,77037458	1,28E-11	4,00E-10	SNX30
ENSGALG00000028125	921,395648	0,831550629	0,18036279	4,6104334	4,02E-06	4,52E-05	
ENSGALG00000025776	414,67908	0,83014723	0,21462899	3,86782432	0,00010981	0,00081166	
ENSGALG00000027903	10650,6436	0,829170411	0,11053296	7,50156702	6,31E-14	2,64E-12	
ENSGALG00000010500	3026,41246	0,828235743	0,13002656	6,36974259	1,89E-10	4,98E-09	NKX6-2
ENSGALG00000007015	336,010164	0,825119263	0,22964958	3,59294921	0,00032696	0,00209581	CD40
ENSGALG00000015720	662,971603	0,824601233	0,20777276	3,96876479	7,22E-05	0,00056596	CHODL
ENSGALG00000011744	1298,60197	0,824149013	0,17298432	4,76429887	1,90E-06	2,30E-05	
ENSGALG00000016558	512,367292	0,822973317	0,20099047	4,09458879	4,23E-05	0,00035587	VEGFD
ENSGALG00000001688	9554,36542	0,822044682	0,12651935	6,49738304	8,17E-11	2,27E-09	sept-05
ENSGALG00000015941	330,156486	0,819153887	0,23360451	3,50658414	0,0004539	0,00276255	
ENSGALG00000019768	3223,32241	0,819142269	0,12221578	6,70242617	2,05E-11	6,19E-10	ACSF2

ENSGALG00000010698	1578,35111	0,816916741	0,1404823	5,81508672	6,06E-09	1,25E-07	
ENSGALG00000012628	1115,10605	0,816609796	0,18822357	4,33850978	1,43E-05	0,00014153	HABP4
ENSGALG00000027534	844,147038	0,815389343	0,17402749	4,68540539	2,79E-06	3,27E-05	
ENSGALG00000010397	282,533201	0,813527008	0,25347167	3,20953824	0,00132948	0,0067667	GRID2
ENSGALG00000027338	409,810985	0,813282814	0,21988916	3,6986034	0,00021679	0,00146626	
ENSGALG00000015297	1260,54646	0,81224071	0,16743713	4,8510192	1,23E-06	1,57E-05	FOXO3
ENSGALG00000000954	405,070556	0,812029159	0,24204134	3,35491929	0,00079388	0,00442073	
ENSGALG00000006480	1820,43736	0,810690895	0,14795092	5,47945836	4,27E-08	7,41E-07	TCF7
ENSGALG00000011861	430,597577	0,810665245	0,21257164	3,81360956	0,00013695	0,00098656	SYT16
ENSGALG00000009680	349,521496	0,809243098	0,22080925	3,6648968	0,00024744	0,00164472	PAQR7
ENSGALG00000015051	777,652051	0,807158582	0,18251219	4,42249142	9,76E-06	0,00010087	CDK19
ENSGALG00000013051	1092,69729	0,806546944	0,19222288	4,19589469	2,72E-05	0,00024358	
ENSGALG00000005147	486,928416	0,80606613	0,20058324	4,0186116	5,85E-05	0,00047135	
ENSGALG00000017016	265,337815	0,804980828	0,247719	3,24957242	0,00115579	0,00602451	FAM124A
ENSGALG00000015608	746,798973	0,802634493	0,1733654	4,62972702	3,66E-06	4,17E-05	ADRA2C
ENSGALG00000011394	463,382496	0,801850887	0,20513291	3,9089335	9,27E-05	0,0007038	
ENSGALG00000007565	677,702896	0,798729286	0,22132239	3,60889512	0,0003075	0,00198514	PPP2R2B
ENSGALG00000012073	470,076315	0,797159786	0,20392037	3,90917193	9,26E-05	0,0007038	
ENSGALG00000008408	1323,03511	0,796075417	0,15213548	5,23267422	1,67E-07	2,57E-06	GAB3
ENSGALG00000014122	10545,592	0,795943411	0,13498611	5,89648381	3,71E-09	8,08E-08	CYB5R3
ENSGALG00000013489	630,058844	0,794845244	0,18721113	4,24571568	2,18E-05	0,00020195	
ENSGALG00000015233	5666,23676	0,794464087	0,15751312	5,0437964	4,56E-07	6,39E-06	ATP1B1
ENSGALG00000006507	1217,4322	0,793097237	0,15342058	5,16943198	2,35E-07	3,49E-06	CLMP
ENSGALG00000026605	1028,74302	0,792369586	0,15815822	5,00998056	5,44E-07	7,52E-06	
ENSGALG00000007720	244,442979	0,792327844	0,25557464	3,1001818	0,00193402	0,009178	AIFM3
ENSGALG00000006319	831,267138	0,791303644	0,17376438	4,55388859	5,27E-06	5,80E-05	
ENSGALG00000028576	735,470308	0,790565069	0,23138694	3,4166365	0,000634	0,00365294	
ENSGALG00000010708	2290,46376	0,790532368	0,15028519	5,26021489	1,44E-07	2,24E-06	ICA1
ENSGALG00000021589	1830,74644	0,790216265	0,177386	4,45478374	8,40E-06	8,83E-05	RTN4RL1
ENSGALG00000027458	914,195785	0,790135397	0,16348734	4,83300661	1,34E-06	1,70E-05	
ENSGALG00000014300	310,956459	0,790002156	0,25045648	3,15424928	0,00160912	0,00788629	
ENSGALG00000013862	6295,07557	0,789845326	0,14390944	5,48848856	4,05E-08	7,08E-07	OLIG3
ENSGALG00000026899	678,260512	0,789824953	0,18654004	4,2340774	2,29E-05	0,00021097	
ENSGALG00000020522	2265,96477	0,78974705	0,15263795	5,17398877	2,29E-07	3,42E-06	
ENSGALG00000009039	1670,74492	0,789311851	0,14682451	5,375886	7,62E-08	1,25E-06	
ENSGALG00000003933	4489,5312	0,788744436	0,13110215	6,01625856	1,78E-09	4,11E-08	QSOX1
ENSGALG00000013642	1029,71125	0,788560165	0,16199374	4,86784356	1,13E-06	1,46E-05	
ENSGALG00000006453	2069,6286	0,788464487	0,15333542	5,14208958	2,72E-07	3,98E-06	
ENSGALG00000007623	3327,22263	0,788173273	0,13967263	5,64300463	1,67E-08	3,18E-07	CACNA1G
ENSGALG00000024097	498,632385	0,787003033	0,25367596	3,10239499	0,00191962	0,00911918	
ENSGALG00000005849	1033,38166	0,784505411	0,16373067	4,79143821	1,66E-06	2,05E-05	PPDPF
ENSGALG00000000076	922,879923	0,7843985	0,16221807	4,83545708	1,33E-06	1,68E-05	
ENSGALG00000012116	675,676944	0,783832372	0,18759243	4,17837954	2,94E-05	0,00026058	
ENSGALG00000011635	890,924795	0,783625313	0,17783173	4,40655507	1,05E-05	0,00010776	TFCP2L1
ENSGALG00000027788	2064,81163	0,782858625	0,18478838	4,23651437	2,27E-05	0,00020926	CPE
ENSGALG00000009866	599,485373	0,782677583	0,20855076	3,75293566	0,00017478	0,00122029	
ENSGALG00000020376	2337,04095	0,781084057	0,12991704	6,01217582	1,83E-09	4,20E-08	
ENSGALG00000007357	1727,63044	0,780757803	0,17882552	4,36603111	1,27E-05	0,00012639	BCL6
ENSGALG00000009407	420,644478	0,780277328	0,21471092	3,63408309	0,00027897	0,00182342	
ENSGALG00000021620	400,193416	0,780112352	0,21582196	3,61461062	0,0003008	0,00194647	TMEM59L
ENSGALG00000005657	1485,50445	0,780089243	0,20993525	3,71585638	0,00020252	0,00137863	CRHR2
ENSGALG00000017065	605,990734	0,779643959	0,22129554	3,52308929	0,00042655	0,00263018	
ENSGALG00000015379	3926,96098	0,779239634	0,17207727	4,5284285	5,94E-06	6,41E-05	TAGLN3
ENSGALG00000005539	507,508669	0,77704495	0,19834408	3,9176614	8,94E-05	0,00068237	
ENSGALG00000009612	1284,26197	0,776962988	0,15672769	4,95740715	7,14E-07	9,55E-06	
ENSGALG00000005421	527,433616	0,776882668	0,19330399	4,01896854	5,85E-05	0,00047091	
ENSGALG00000026326	2889,79677	0,77526969	0,14658762	5,28878011	1,23E-07	1,94E-06	
ENSGALG00000002708	2271,23083	0,772455755	0,1693238	4,56200355	5,07E-06	5,60E-05	LINGO1
ENSGALG00000013404	465,070427	0,770980594	0,20453334	3,76946168	0,0001636	0,00115171	
ENSGALG00000002283	1172,58396	0,770174106	0,17180246	4,482905	7,36E-06	7,80E-05	MAPKAPK3
ENSGALG00000017208	521,560672	0,769802089	0,19597474	3,92806787	8,56E-05	0,0006572	AMOTL1
ENSGALG00000027592	1174,35152	0,765780883	0,16676307	4,59202906	4,39E-06	4,91E-05	ERN2

ENSGALG00000005779	2301,58905	0,765244932	0,12900388	5,93195293	2,99E-09	6,58E-08	
ENSGALG00000006900	414,726982	0,764663976	0,24206393	3,15893399	0,00158347	0,00778022	
ENSGALG00000004323	3702,95548	0,7637615	0,15205572	5,02290526	5,09E-07	7,07E-06	RASAL2
ENSGALG00000007744	1224,40316	0,76351713	0,16453915	4,64033711	3,48E-06	3,98E-05	CASS4
ENSGALG00000002069	610,168852	0,761037525	0,20960313	3,63085008	0,00028249	0,00184376	
ENSGALG00000013948	736,872876	0,760675086	0,17278143	4,40252804	1,07E-05	0,00010953	RASL11B
ENSGALG00000007956	2266,7266	0,759404394	0,14550515	5,2190893	1,80E-07	2,75E-06	PLXDC2
ENSGALG00000007748	2468,69748	0,758936347	0,12655699	5,9967954	2,01E-09	4,56E-08	SLC16A2
ENSGALG00000004170	385,321336	0,75875998	0,23438122	3,23729002	0,00120671	0,00624926	ADA
ENSGALG00000015688	1188,53051	0,758515207	0,17302156	4,38393456	1,17E-05	0,00011807	MXD4
ENSGALG00000003115	505,204817	0,756684773	0,20221429	3,74199456	0,00018257	0,00126624	
ENSGALG00000007889	769,261981	0,756666335	0,18466224	4,09756933	4,18E-05	0,00035285	
ENSGALG00000006346	1339,32033	0,756405715	0,1832878	4,12687423	3,68E-05	0,00031627	CXCL14
ENSGALG00000026153	524,420919	0,755983234	0,23146908	3,26602248	0,0010907	0,00574239	
ENSGALG00000002958	2112,95038	0,755313619	0,16744802	4,51073495	6,46E-06	6,90E-05	DNER
ENSGALG00000001311	1301,90092	0,752137925	0,15185177	4,95310607	7,30E-07	9,72E-06	
ENSGALG00000027905	3422,01429	0,751625166	0,13298701	5,65186918	1,59E-08	3,03E-07	CSRNP1
ENSGALG00000009439	631,532608	0,751125582	0,2024985	3,70928955	0,00020784	0,00141276	GPR85
ENSGALG00000029047	339,90745	0,749178885	0,22666604	3,30521004	0,00094905	0,00512549	TMEM204
ENSGALG00000015729	1439,09217	0,747405326	0,14976	4,99068712	6,02E-07	8,18E-06	LPAR1
ENSGALG00000001969	1252,39504	0,746864271	0,15500447	4,81834019	1,45E-06	1,82E-05	
ENSGALG00000004420	2554,14464	0,745430944	0,15350993	4,85591338	1,20E-06	1,54E-05	SDK1
ENSGALG00000018733	341,104932	0,744919582	0,22358708	3,33167538	0,00086325	0,0047277	
ENSGALG00000005063	630,430329	0,74466275	0,19563887	3,8063128	0,00014105	0,0010113	ST6GALNAC4
ENSGALG00000026919	497,57567	0,743491927	0,20017411	3,71422615	0,00020383	0,00138686	
ENSGALG00000025937	424,74226	0,743354667	0,21925587	3,39035244	0,00069803	0,00397144	
ENSGALG00000012712	2097,8069	0,742584862	0,14980046	4,95716018	7,15E-07	9,56E-06	RBM24
ENSGALG00000015994	1103,99088	0,741778145	0,15501143	4,78531262	1,71E-06	2,10E-05	
ENSGALG00000001449	3789,5374	0,740070536	0,14515669	5,09842522	3,42E-07	4,93E-06	STRA6
ENSGALG00000026449	3878,57981	0,737442369	0,13363533	5,51831886	3,42E-08	6,04E-07	
ENSGALG00000002539	465,932804	0,737264559	0,21127231	3,4896412	0,00048367	0,00291895	KCNN2
ENSGALG00000008339	615,635733	0,736061451	0,19489251	3,77675604	0,00015888	0,00112083	
ENSGALG00000013002	3217,45336	0,734684533	0,14696976	4,99888241	5,77E-07	7,89E-06	DAP1
ENSGALG00000019087	2799,71591	0,734240753	0,12953887	5,66811147	1,44E-08	2,78E-07	RFXAP
ENSGALG00000011059	526,882943	0,733913318	0,20211997	3,6310777	0,00028224	0,00184302	
ENSGALG00000010326	286,241823	0,733495449	0,23722679	3,09195881	0,0019884	0,00938825	FLVCR2
ENSGALG00000008433	2910,77768	0,732487	0,13031486	5,62090169	1,90E-08	3,57E-07	
ENSGALG00000008076	5047,29607	0,731953468	0,12742676	5,74411123	9,24E-09	1,83E-07	TMEM164
ENSGALG00000006897	1904,48459	0,73176707	0,13708377	5,33810137	9,39E-08	1,52E-06	
ENSGALG00000025916	1511,63504	0,730226914	0,19711664	3,70454229	0,00021177	0,0014359	
ENSGALG00000002651	2712,11427	0,730025866	0,12886096	5,6652212	1,47E-08	2,82E-07	ST3GAL2
ENSGALG00000012191	7671,58272	0,72968259	0,12563695	5,80786629	6,33E-09	1,30E-07	
ENSGALG00000002451	1225,15716	0,728956851	0,15337655	4,75272686	2,01E-06	2,41E-05	
ENSGALG00000019317	6925,16498	0,728083244	0,12000428	6,06714384	1,30E-09	3,05E-08	
ENSGALG00000000400	352,092636	0,727285624	0,21940045	3,31487749	0,00091683	0,00498112	
ENSGALG00000014910	3784,53082	0,72588259	0,16231129	4,47216332	7,74E-06	8,18E-05	COLEC12
ENSGALG00000004875	3335,27386	0,724796191	0,1381226	5,24748459	1,54E-07	2,39E-06	PEMT
ENSGALG00000015123	798,450057	0,722653298	0,17223363	4,19577343	2,72E-05	0,00024358	ZDHHC23
ENSGALG00000026864	531,42097	0,721498621	0,20303604	3,55354952	0,00038007	0,00237478	ZCCHC24
ENSGALG00000011426	2063,22886	0,721230387	0,16133795	4,4703085	7,81E-06	8,24E-05	
ENSGALG00000000723	8396,32677	0,72079059	0,14050982	5,12982366	2,90E-07	4,21E-06	STX12
ENSGALG00000002558	389,45186	0,720642154	0,22908371	3,14575908	0,00165656	0,00806657	
ENSGALG00000001459	1037,33334	0,719793089	0,17879018	4,02590944	5,68E-05	0,00045941	TNNC1
ENSGALG00000007981	2067,61165	0,719546729	0,14285858	5,0367766	4,73E-07	6,61E-06	CLN6
ENSGALG00000009042	1726,61695	0,719468223	0,14937565	4,81650257	1,46E-06	1,83E-05	ISM1
ENSGALG00000003855	2094,24658	0,719100031	0,13684545	5,25483329	1,48E-07	2,31E-06	
ENSGALG00000015403	10307,4906	0,718054416	0,13178716	5,44859159	5,08E-08	8,65E-07	EPHA3
ENSGALG00000007383	515,088556	0,717575013	0,20175921	3,55659118	0,0003757	0,00235287	HSPB8
ENSGALG00000012792	5661,79807	0,717520705	0,12510356	5,73541388	9,73E-09	1,92E-07	HIPK2
ENSGALG00000013583	619,651043	0,717484093	0,2169479	3,30717227	0,00094243	0,00509578	FAM114A1
ENSGALG00000004812	420,513925	0,714148656	0,22761509	3,13752767	0,00170379	0,00826109	FAM129A
ENSGALG00000003340	440,9962	0,714011033	0,21324869	3,34825521	0,00081322	0,00449533	GBGT1

ENSGALG00000001233	1022,31515	0,713516886	0,17513042	4,07420311	4,62E-05	0,00038331	
ENSGALG00000007244	345,610478	0,711922899	0,2206692	3,22619966	0,00125446	0,00644774	SIRT4
ENSGALG00000009628	330,245656	0,711918795	0,2307632	3,08506204	0,0020351	0,00958086	
ENSGALG00000003282	5419,41789	0,711820753	0,11116608	6,40321912	1,52E-10	4,05E-09	STAT5B
ENSGALG00000007196	4102,69674	0,710478048	0,12809281	5,54658791	2,91E-08	5,23E-07	
ENSGALG00000003805	6589,80394	0,710406685	0,12973331	5,47590036	4,35E-08	7,53E-07	PRKG1
ENSGALG00000001130	1241,25137	0,709264893	0,16240514	4,36725635	1,26E-05	0,00012586	WBSCR17
ENSGALG00000002038	769,11793	0,706334316	0,17558941	4,02264751	5,75E-05	0,00046499	DENND2C
ENSGALG00000028443	5716,4355	0,705249503	0,16905495	4,17171751	3,02E-05	0,00026727	
ENSGALG00000023738	705,328876	0,704601132	0,2001565	3,52025109	0,00043114	0,00265129	
ENSGALG00000010320	1626,02269	0,704383823	0,13846801	5,0869789	3,64E-07	5,19E-06	PAWR
ENSGALG00000026437	5573,48841	0,703536871	0,11568829	6,0813142	1,19E-09	2,80E-08	
ENSGALG00000004735	2180,59814	0,703327732	0,14882879	4,72575054	2,29E-06	2,73E-05	
ENSGALG00000026973	1340,81752	0,702510997	0,14635529	4,80003844	1,59E-06	1,97E-05	KIF3C
ENSGALG00000026873	606,998571	0,701267796	0,1971895	3,55631416	0,00037609	0,00235426	
ENSGALG00000023266	1017,69934	0,69953934	0,17755104	3,93993386	8,15E-05	0,00062871	
ENSGALG00000027058	1118,35276	0,699526976	0,185719	3,76658817	0,00016549	0,00116401	EMP2
ENSGALG00000020497	375,964711	0,698148301	0,21726001	3,21342288	0,00131163	0,00669232	
ENSGALG00000004647	2581,30282	0,697164089	0,1609537	4,33145725	1,48E-05	0,0001453	
ENSGALG00000015181	1358,59834	0,696928823	0,1980876	3,51828604	0,00043434	0,00266338	GAREM1
ENSGALG00000028945	1325,88323	0,696399862	0,19729376	3,52976114	0,00041594	0,0025729	
ENSGALG00000007349	388,214699	0,696333265	0,21422064	3,25054234	0,00115185	0,00601091	RASL12
ENSGALG00000008455	4612,74446	0,695894902	0,1608753	4,32567893	1,52E-05	0,00014811	
ENSGALG00000010094	8338,3892	0,695566561	0,11023706	6,30973457	2,80E-10	7,11E-09	B4GALT2
ENSGALG00000010331	1143,01041	0,693784562	0,19974706	3,47331558	0,00051407	0,00306708	MME
ENSGALG00000010406	684,710723	0,693540735	0,17759525	3,9051762	9,42E-05	0,00071219	TMEM63C
ENSGALG00000001889	934,570663	0,693259058	0,17268521	4,01458276	5,96E-05	0,00047852	CARD9
ENSGALG00000008651	2483,20041	0,693028969	0,15778512	4,39223263	1,12E-05	0,00011425	APMAP
ENSGALG00000005103	1726,78257	0,692645693	0,13835836	5,00617158	5,55E-07	7,63E-06	GDPD1
ENSGALG00000023188	975,838184	0,692540652	0,16000524	4,32823725	1,50E-05	0,00014706	NANP
ENSGALG00000003636	432,590451	0,690941222	0,20908855	3,3045388	0,00095133	0,00513167	FBLIM1
ENSGALG00000020575	447,180257	0,690014438	0,22107615	3,12116177	0,00180139	0,00863589	
ENSGALG00000023655	726,630068	0,689103212	0,1781324	3,86848893	0,00010951	0,00081033	
ENSGALG00000003122	2359,00023	0,68839827	0,16356115	4,20881277	2,57E-05	0,0002327	ZFAND2A
ENSGALG00000002749	1561,25313	0,687223145	0,13989637	4,91237283	9,00E-07	1,18E-05	
ENSGALG00000016844	1336,96906	0,685906484	0,15616188	4,39227854	1,12E-05	0,00011425	RAB20
ENSGALG00000016123	816,293249	0,685692574	0,20965616	3,27055776	0,00107336	0,00566864	
ENSGALG00000002204	670,701965	0,684953864	0,19602604	3,49419842	0,00047549	0,00287595	SLC7A4
ENSGALG00000014559	1808,90594	0,684328525	0,1507966	4,53808981	5,68E-06	6,17E-05	
ENSGALG00000009075	1175,32124	0,683878573	0,15401179	4,44042999	8,98E-06	9,38E-05	CD99L2
ENSGALG00000010770	1614,38779	0,683740122	0,16437692	4,15958702	3,19E-05	0,00028005	SCIN
ENSGALG00000009523	834,574184	0,681144754	0,18607441	3,66060408	0,00025162	0,00166626	CNTN1
ENSGALG00000007251	3608,08828	0,681060329	0,12144401	5,60801928	2,05E-08	3,82E-07	PCYT2
ENSGALG00000019702	11617,3005	0,678512546	0,13266103	5,11463354	3,14E-07	4,54E-06	
ENSGALG00000000674	418,548479	0,67836471	0,21301294	3,18461735	0,00144946	0,00725001	MYO1D
ENSGALG00000020688	589,72193	0,678030575	0,21326458	3,17929294	0,00147635	0,00733594	
ENSGALG00000026986	1312,77213	0,67793682	0,16864646	4,01986992	5,82E-05	0,00046939	
ENSGALG00000004240	977,710505	0,676367039	0,17381981	3,89119644	9,98E-05	0,00074827	ORAI1
ENSGALG00000025764	5164,94672	0,675250138	0,12251808	5,51143253	3,56E-08	6,27E-07	FKBP10
ENSGALG00000002028	1196,67299	0,675138088	0,19853735	3,40055964	0,00067248	0,00384375	GPRC5B
ENSGALG00000012583	448,709518	0,674342421	0,20797125	3,24247904	0,00118495	0,00615061	IDNK
ENSGALG00000016399	1323,25053	0,673456474	0,16094579	4,18436848	2,86E-05	0,00025497	
ENSGALG00000004472	11283,0726	0,672299012	0,12223168	5,50020247	3,79E-08	6,65E-07	ASTN1
ENSGALG00000005041	1267,24433	0,67175632	0,1650486	4,07005162	4,70E-05	0,00038926	TMEM35A
ENSGALG00000011896	1990,38793	0,671754963	0,13345151	5,03370069	4,81E-07	6,71E-06	
ENSGALG00000007038	2302,52795	0,670533206	0,13522368	4,95869653	7,10E-07	9,50E-06	
ENSGALG00000005069	1045,94718	0,669583836	0,15806848	4,23603637	2,27E-05	0,00020957	
ENSGALG00000000040	692,575173	0,669045681	0,18677509	3,58209261	0,00034085	0,00217059	
ENSGALG00000000920	727,437453	0,668851002	0,19789956	3,37974989	0,00072552	0,00410213	CGN
ENSGALG00000016415	1383,10646	0,668618298	0,18669663	3,58130896	0,00034188	0,00217443	MAP7D2
ENSGALG00000016617	4902,5535	0,667497538	0,12877865	5,18329341	2,18E-07	3,27E-06	NCOA1
ENSGALG00000008970	735,022626	0,666872911	0,18064169	3,69168889	0,00022277	0,00150001	

ENSGALG00000006449	910,146444	0,666661271	0,19615763	3,39859966	0,00067732	0,00386978	
ENSGALG00000003457	3064,26122	0,662964808	0,14313215	4,63183715	3,62E-06	4,14E-05	CNP
ENSGALG00000013766	661,354996	0,662853472	0,19762543	3,35409001	0,00079626	0,00443219	
ENSGALG00000010316	7099,3915	0,662660688	0,11945249	5,54748328	2,90E-08	5,21E-07	FRAS1
ENSGALG00000002775	509,116953	0,661278251	0,20564659	3,21560519	0,0013017	0,00665263	FA2H
ENSGALG00000012710	1279,29723	0,660750331	0,15866836	4,16434841	3,12E-05	0,00027501	NCS1
ENSGALG00000016411	1960,74491	0,657856223	0,15713168	4,18665555	2,83E-05	0,00025274	
ENSGALG00000006152	1208,2078	0,657637784	0,1593989	4,12573602	3,70E-05	0,00031724	
ENSGALG00000018967	1911,68524	0,656123394	0,18491847	3,54817659	0,00038791	0,00241489	
ENSGALG00000016543	3236,91171	0,654951402	0,17013318	3,84963956	0,00011829	0,00086402	NHS
ENSGALG00000012023	986,869481	0,654468757	0,16148919	4,0527095	5,06E-05	0,00041599	DACT1
ENSGALG00000003845	2106,1901	0,653837192	0,13713933	4,76768555	1,86E-06	2,26E-05	MKNK2
ENSGALG00000003589	5654,7984	0,652278144	0,1207315	5,40271723	6,56E-08	1,10E-06	VTN
ENSGALG00000001426	1341,21178	0,651807691	0,14847266	4,39008564	1,13E-05	0,00011521	
ENSGALG00000002403	2862,69893	0,651643976	0,15300671	4,25892429	2,05E-05	0,00019195	ULK1
ENSGALG00000001956	2538,00503	0,650817237	0,17613054	3,69508455	0,00021981	0,00148303	OLFML3
ENSGALG00000008120	6095,80519	0,649872522	0,12621953	5,14874782	2,62E-07	3,86E-06	TLE3
ENSGALG00000024104	1479,60024	0,64969951	0,15049285	4,31714531	1,58E-05	0,00015284	
ENSGALG00000024488	3112,83733	0,649250333	0,1855727	3,49863056	0,00046765	0,00283487	CD99
ENSGALG00000008763	548,608658	0,64905793	0,2030908	3,19590022	0,00139395	0,00702134	SSX2IP
ENSGALG00000007848	1247,63563	0,647083546	0,1839624	3,51747722	0,00043567	0,00266711	PTS
ENSGALG00000010422	5513,26975	0,646628908	0,1246783	5,1863789	2,14E-07	3,22E-06	
ENSGALG00000010719	1138,21283	0,646215963	0,15983765	4,04295223	5,28E-05	0,00043187	NEIL3
ENSGALG00000006036	6926,72973	0,644947298	0,14183609	4,54713104	5,44E-06	5,94E-05	
ENSGALG00000003655	1118,25271	0,643488267	0,18487926	3,48058661	0,00050032	0,00300212	GAS2
ENSGALG00000000402	743,970165	0,642116084	0,18043203	3,55876987	0,0003726	0,00233882	LOXL2
ENSGALG00000006947	23413,053	0,64187784	0,11085732	5,79012569	7,03E-09	1,43E-07	BDH1
ENSGALG00000011335	567,87108	0,64086738	0,205863	3,11307701	0,00185148	0,00882628	
ENSGALG00000026159	2624,11695	0,639768169	0,13669036	4,68041921	2,86E-06	3,34E-05	PDLIM7
ENSGALG00000026295	3751,17129	0,638993968	0,12561216	5,08703909	3,64E-07	5,19E-06	CDR2L
ENSGALG00000000935	1038,82432	0,638135684	0,19518262	3,26942888	0,00107765	0,0056891	
ENSGALG00000028506	1185,60599	0,637581477	0,16691697	3,81975226	0,00013359	0,00096486	NHSL2
ENSGALG00000003527	1639,11059	0,636215476	0,14708776	4,32541425	1,52E-05	0,00014816	ARRDC2
ENSGALG00000028756	1081,22564	0,636133999	0,16633022	3,82452444	0,00013102	0,00094737	
ENSGALG00000004585	1609,34206	0,634633954	0,16521991	3,84114698	0,00012246	0,00089065	
ENSGALG00000008057	2849,96428	0,63255389	0,14696671	4,30406235	1,68E-05	0,0001609	LLGL2
ENSGALG00000011053	10065,6567	0,630484727	0,10769501	5,85435431	4,79E-09	1,01E-07	
ENSGALG00000008151	5862,81637	0,629127593	0,11865044	5,30236208	1,14E-07	1,81E-06	WSB2
ENSGALG00000027658	594,253982	0,628365542	0,19060887	3,29662282	0,00097855	0,005237	TESK1
ENSGALG00000002664	1165,54673	0,627944771	0,15316524	4,09978643	4,14E-05	0,00035053	
ENSGALG00000029000	1142,4251	0,627547484	0,15842199	3,96123969	7,46E-05	0,00058075	HYAL3
ENSGALG00000028204	8948,35307	0,626531339	0,12182055	5,14306756	2,70E-07	3,97E-06	
ENSGALG00000001100	796,408366	0,626475621	0,18020964	3,47637138	0,00050825	0,00303934	
ENSGALG00000002313	2718,417	0,626374122	0,13464234	4,65213353	3,29E-06	3,79E-05	
ENSGALG00000026762	1809,40153	0,625670937	0,1367941	4,5738152	4,79E-06	5,34E-05	
ENSGALG00000008728	894,547623	0,625207283	0,17070647	3,66246966	0,0002498	0,00165578	PTER
ENSGALG00000011856	1033,65184	0,624422163	0,15756316	3,96299593	7,40E-05	0,00057782	FMC1
ENSGALG00000007267	4451,86283	0,623851268	0,11702334	5,33099856	9,77E-08	1,57E-06	MVD
ENSGALG00000014890	1413,33309	0,620763059	0,14626866	4,24399216	2,20E-05	0,00020309	
ENSGALG00000000426	554,757236	0,620684473	0,19913485	3,11690528	0,0018276	0,00873694	
ENSGALG00000006114	909,882198	0,61972927	0,17107416	3,62257682	0,00029168	0,0018965	
ENSGALG00000011900	1365,07707	0,61861917	0,17981254	3,44035605	0,00058095	0,00339761	NAGA
ENSGALG00000007076	2564,35145	0,617550884	0,14376263	4,29562882	1,74E-05	0,00016632	CCNY
ENSGALG00000007381	1220,44249	0,615500074	0,15532009	3,96278456	7,41E-05	0,000578	SERPING1
ENSGALG00000028543	1301,49454	0,614319385	0,1647996	3,72767515	0,00019325	0,00132563	FGFR4
ENSGALG00000015734	903,95178	0,61429478	0,19839853	3,09626685	0,00195974	0,00928387	
ENSGALG00000016728	5650,40168	0,613362339	0,11515602	5,32635913	1,00E-07	1,61E-06	RP2
ENSGALG00000016092	954,718038	0,611645343	0,16439834	3,72050802	0,00019882	0,00135687	
ENSGALG00000011975	2830,82544	0,61116717	0,12275633	4,97870188	6,40E-07	8,65E-06	
ENSGALG00000016143	5447,09101	0,607480677	0,17132527	3,54577386	0,00039146	0,0024359	
ENSGALG00000008512	665,497169	0,606896507	0,19319454	3,14137499	0,00168157	0,00816786	
ENSGALG00000008272	1496,82423	0,6063144	0,15709843	3,8594554	0,00011364	0,00083633	TMED3

ENSGALG00000015414	1285,1197	0,605396806	0,18042117	3,35546434	0,00079232	0,00441384	NSUN3
ENSGALG00000002930	5292,49241	0,603605896	0,13766556	4,38458178	1,16E-05	0,00011781	
ENSGALG00000005303	729,474387	0,603106981	0,18273634	3,30042173	0,0009654	0,00518701	CCDC13
ENSGALG00000006742	8579,7495	0,60288418	0,10750963	5,60772266	2,05E-08	3,82E-07	
ENSGALG00000013964	1158,34472	0,602864404	0,163189	3,69427107	0,00022052	0,00148632	SPATA18
ENSGALG00000000739	566,84692	0,602546305	0,18860198	3,19480372	0,00139926	0,00704546	
ENSGALG00000009047	3574,12193	0,601534497	0,13925729	4,3195906	1,56E-05	0,0001517	YPEL5
ENSGALG00000005628	1589,65421	0,601333554	0,18106388	3,3211128	0,00089659	0,00488488	COL9A3
ENSGALG00000029021	4810,26751	0,600451025	0,1243158	4,83004601	1,37E-06	1,72E-05	
ENSGALG00000023435	807,96294	0,600270173	0,18996812	3,15984693	0,00157852	0,0077671	GATM
ENSGALG00000023876	5804,17122	0,598475769	0,12582855	4,75627963	1,97E-06	2,38E-05	
ENSGALG00000008475	1732,98273	0,598095279	0,145561	4,10889791	3,98E-05	0,00033935	ARRDC1
ENSGALG00000013167	14635,2035	0,597899753	0,11791074	5,07078291	3,96E-07	5,61E-06	SDHA
ENSGALG00000007801	1966,88294	0,597201094	0,14607757	4,08824628	4,35E-05	0,00036327	
ENSGALG00000027450	1958,10116	0,596865465	0,13602137	4,3880272	1,14E-05	0,00011605	
ENSGALG00000002818	12498,9505	0,595523293	0,12527412	4,75376167	2,00E-06	2,41E-05	
ENSGALG00000008285	2205,39659	0,594659875	0,13357092	4,4520161	8,51E-06	8,94E-05	NAPEPLD
ENSGALG00000026756	608,701473	0,593058479	0,18596131	3,1891498	0,00142692	0,00716353	
ENSGALG00000011145	3513,15874	0,5922185	0,11951587	4,95514543	7,23E-07	9,63E-06	TRIL
ENSGALG00000026180	4122,30438	0,591162305	0,14254212	4,14728156	3,36E-05	0,00029326	
ENSGALG00000005999	5083,90869	0,591144605	0,12187871	4,85026945	1,23E-06	1,57E-05	ABTB1
ENSGALG00000013436	9895,67369	0,590008237	0,1280495	4,60765752	4,07E-06	4,58E-05	
ENSGALG00000013742	6102,59896	0,589905166	0,14728573	4,0051753	6,20E-05	0,00049428	EZR
ENSGALG00000025745	2020,21836	0,589609597	0,16557075	3,56107338	0,00036934	0,0023216	
ENSGALG00000026081	3861,36621	0,588777417	0,1223416	4,81256939	1,49E-06	1,86E-05	RBM38
ENSGALG00000000464	6973,12042	0,587838297	0,11000425	5,34377783	9,10E-08	1,48E-06	
ENSGALG00000005102	2254,46112	0,58732124	0,16766068	3,50303501	0,00045999	0,00279338	CDH4
ENSGALG00000016594	1080,56836	0,58674351	0,17030861	3,44517825	0,00057068	0,00334619	
ENSGALG00000010934	813,601759	0,586239182	0,17064205	3,43549068	0,00059148	0,00345031	
ENSGALG00000025822	1186,62395	0,586217768	0,19001165	3,08516751	0,00203438	0,00958078	CYP1B1
ENSGALG00000017558	835,379673	0,585776262	0,17286518	3,38863076	0,00070243	0,00398832	
ENSGALG00000002169	5307,04919	0,58477373	0,13930071	4,19792355	2,69E-05	0,0002424	PLEKHB2
ENSGALG00000025738	2313,93547	0,584187633	0,13782283	4,23868547	2,25E-05	0,00020753	
ENSGALG00000015938	2888,98507	0,583396297	0,14475468	4,03024131	5,57E-05	0,00045237	
ENSGALG00000001574	3769,69235	0,583342794	0,12963845	4,49976697	6,80E-06	7,24E-05	
ENSGALG00000014242	1647,30473	0,583190799	0,15565274	3,74674285	0,00017915	0,0012457	
ENSGALG00000008999	3276,84717	0,583129446	0,15516573	3,75810727	0,0001712	0,00119904	AFAP1L2
ENSGALG00000010706	6618,96251	0,582943162	0,11294938	5,16110101	2,46E-07	3,64E-06	
ENSGALG00000004844	6201,27995	0,581688828	0,14767508	3,93897768	8,18E-05	0,00063051	
ENSGALG00000009604	5450,24379	0,581479374	0,13237918	4,39252905	1,12E-05	0,00011425	DBX2
ENSGALG00000001446	6585,16039	0,581431635	0,15010679	3,8734532	0,0001073	0,00079659	
ENSGALG00000023768	4728,02752	0,58136874	0,11646418	4,99182452	5,98E-07	8,14E-06	
ENSGALG00000002707	937,598421	0,58133458	0,18027574	3,22469665	0,00126106	0,00647656	CHRNB2
ENSGALG00000029020	1577,39916	0,581217481	0,17198927	3,37938213	0,00072649	0,00410592	PLPP4
ENSGALG00000028478	1030,91271	0,580829913	0,18242777	3,18388976	0,0014531	0,00725779	
ENSGALG00000007531	904,342369	0,57752222	0,17317755	3,33485613	0,00085344	0,00468152	ZFYVE27
ENSGALG00000006054	759,821789	0,576882549	0,18206756	3,1685082	0,00153223	0,00756944	CALCA
ENSGALG00000022926	4686,66572	0,576442566	0,12615332	4,56938097	4,89E-06	5,45E-05	GPR162
ENSGALG00000013185	3965,04447	0,575558846	0,14856351	3,87416023	0,00010699	0,00079645	
ENSGALG00000000869	855,333756	0,574690811	0,17351236	3,31210309	0,00092597	0,00502477	
ENSGALG00000006885	6383,54265	0,574664846	0,12640826	4,54610186	5,46E-06	5,96E-05	
ENSGALG00000015136	2417,80657	0,573614313	0,16092955	3,56438149	0,00036472	0,0022957	ILDR1
ENSGALG00000015744	3529,13316	0,573305804	0,12554323	4,56660089	4,96E-06	5,50E-05	
ENSGALG00000001489	8334,32619	0,572451499	0,12945603	4,42197621	9,78E-06	0,000101	SSU72
ENSGALG00000014911	1071,97212	0,572338387	0,15722716	3,6402005	0,00027243	0,00178235	ARL15
ENSGALG00000015351	2719,2778	0,572206068	0,17042313	3,35756104	0,00078633	0,00439308	CBLB
ENSGALG00000025818	8546,90555	0,571475138	0,10995302	5,19744834	2,02E-07	3,06E-06	
ENSGALG00000005549	1034,75448	0,569387138	0,15615315	3,64633795	0,000266	0,0017479	CUEDC1
ENSGALG00000024153	1062,25024	0,56872295	0,17097205	3,326409	0,00087973	0,00480831	
ENSGALG00000000186	787,093258	0,568415238	0,17712851	3,20905553	0,00133172	0,00677301	MTCH1
ENSGALG00000001146	1103,97969	0,568231367	0,16866857	3,36892268	0,00075463	0,00423331	UBXN6
ENSGALG00000012788	1309,52475	0,567748724	0,16133631	3,51903868	0,00043311	0,00265983	

ENSGALG00000006873	2079,27658	0,565449117	0,13107514	4,3139312	1,60E-05	0,00015464	PHRF1
ENSGALG00000007093	1910,94783	0,563647311	0,15313163	3,68080257	0,0002325	0,00155583	CYTH1
ENSGALG00000002778	10757,9738	0,563240809	0,10999952	5,12039322	3,05E-07	4,42E-06	
ENSGALG00000028605	2035,83961	0,561167945	0,15780973	3,55597819	0,00037658	0,00235619	
ENSGALG00000002681	1896,59526	0,559637304	0,14922639	3,75025697	0,00017665	0,00123277	
ENSGALG00000016152	7208,33853	0,558712272	0,12592253	4,43695234	9,12E-06	9,51E-05	
ENSGALG00000010307	1309,92001	0,558082008	0,16022503	3,48311375	0,00049562	0,00297918	ACYP1
ENSGALG00000010614	5022,37444	0,557460992	0,12556393	4,4396588	9,01E-06	9,41E-05	SLC25A4
ENSGALG00000001682	1516,35079	0,557286636	0,15010501	3,71264502	0,0002051	0,00139485	FBXL20
ENSGALG00000001238	3616,01439	0,556401234	0,12857642	4,32739711	1,51E-05	0,00014747	STX8
ENSGALG00000002286	73998,0221	0,555324016	0,11102707	5,00169946	5,68E-07	7,79E-06	
ENSGALG00000025787	2442,89077	0,554206894	0,13003561	4,2619625	2,03E-05	0,00018949	
ENSGALG00000004849	4017,82732	0,553445556	0,11594023	4,77354206	1,81E-06	2,21E-05	WSCD2
ENSGALG00000001766	1421,20567	0,552758547	0,17371857	3,1819197	0,00146302	0,0072892	
ENSGALG00000010982	4422,55083	0,55035484	0,13935769	3,94922475	7,84E-05	0,00060618	
ENSGALG00000000316	1699,55338	0,549437781	0,1376171	3,99251106	6,54E-05	0,00051769	
ENSGALG00000027194	1293,57158	0,548094206	0,15171291	3,61270652	0,00030302	0,00195897	
ENSGALG00000016166	3894,34548	0,547970517	0,13938637	3,93130636	8,45E-05	0,0006489	
ENSGALG00000006663	9717,81848	0,547489923	0,11710931	4,67503318	2,94E-06	3,41E-05	ARHGEF12
ENSGALG00000001966	2313,19503	0,545673399	0,14563945	3,74674179	0,00017915	0,0012457	
ENSGALG00000001864	1163,99304	0,545343287	0,16861317	3,23428649	0,00121947	0,00630098	C9orf72
ENSGALG00000006598	2327,66634	0,544504824	0,13759012	3,95744117	7,58E-05	0,00058794	SORL1
ENSGALG00000015107	1737,16005	0,542439326	0,13565302	3,99872658	6,37E-05	0,00050646	PIP5K1B
ENSGALG00000008860	2251,91448	0,541261109	0,1571898	3,44336028	0,00057453	0,00336442	LONRF3
ENSGALG00000005315	1118,21521	0,540760838	0,15986155	3,38268231	0,00071782	0,00406365	RPUSD1
ENSGALG00000004033	1001,88457	0,540669975	0,16471038	3,28254945	0,00102873	0,00546475	GALNT10
ENSGALG00000022713	4245,74271	0,539793648	0,1498998	3,60102975	0,00031696	0,00203557	
ENSGALG00000025800	976,781593	0,538353026	0,16267943	3,30928757	0,00093534	0,00506145	
ENSGALG00000028513	5510,29846	0,53711532	0,11230935	4,7824632	1,73E-06	2,12E-05	
ENSGALG00000015437	3953,66098	0,536831128	0,13281019	4,04209288	5,30E-05	0,00043291	
ENSGALG00000007389	2084,84126	0,53455468	0,13272068	4,02766676	5,63E-05	0,00045653	ANKRD40
ENSGALG00000016167	2827,43941	0,530005093	0,12787412	4,14474083	3,40E-05	0,00029577	
ENSGALG00000023189	809,880728	0,529094048	0,16742381	3,16020792	0,00157657	0,00776028	UMAD1
ENSGALG00000009433	12972,2996	0,528259009	0,13672663	3,8636145	0,00011172	0,00082355	BAG3
ENSGALG00000015372	6118,12247	0,526646329	0,10985979	4,79380423	1,64E-06	2,03E-05	ATP5I
ENSGALG00000024346	1302,16175	0,525612903	0,1486838	3,53510544	0,00040761	0,00252716	
ENSGALG00000015306	3107,54178	0,524586766	0,14678095	3,57394315	0,00035165	0,00222785	OSTM1
ENSGALG00000026545	5534,24488	0,524540794	0,12628499	4,15362728	3,27E-05	0,00028597	
ENSGALG00000002777	12410,374	0,524514209	0,1049816	4,99624903	5,85E-07	7,98E-06	
ENSGALG00000015326	10708,8753	0,524363909	0,11790787	4,44723436	8,70E-06	9,12E-05	CLTA
ENSGALG00000014846	1408,26305	0,524315684	0,14757312	3,55292141	0,00038098	0,00237937	
ENSGALG00000011945	1101,05327	0,524157211	0,16753074	3,12872254	0,00175568	0,00846159	
ENSGALG00000006901	2767,40121	0,523326668	0,12428389	4,21073609	2,55E-05	0,00023088	AATK
ENSGALG00000026113	4499,07114	0,523056553	0,14658052	3,56839051	0,00035918	0,0022661	
ENSGALG00000000433	145504,42	0,523011809	0,10274265	5,09050316	3,57E-07	5,11E-06	
ENSGALG00000016886	6214,42839	0,522545779	0,11023413	4,7403267	2,13E-06	2,56E-05	FARP1
ENSGALG00000008367	12605,915	0,522250337	0,11987187	4,35673805	1,32E-05	0,00013139	MDK
ENSGALG00000008911	1167,8871	0,520705327	0,16373625	3,18014692	0,001472	0,00732237	RNF208
ENSGALG00000023338	5372,42979	0,520605081	0,12794507	4,06897339	4,72E-05	0,00039083	CBX2
ENSGALG00000001944	1567,50719	0,519218499	0,15022432	3,45628783	0,00054767	0,00323494	TBC1D2
ENSGALG00000000059	125389,821	0,519152064	0,12052089	4,30756896	1,65E-05	0,00015871	TUBB3
ENSGALG00000005346	2351,81671	0,518505546	0,12959656	4,00092074	6,31E-05	0,00050208	PPIC
ENSGALG00000028033	2016,60758	0,517359519	0,13377729	3,86731945	0,00011004	0,0008129	
ENSGALG00000013024	1376,29845	0,516485359	0,15574832	3,31615365	0,00091266	0,00496041	
ENSGALG00000028567	2485,76766	0,515072598	0,12787307	4,02799904	5,63E-05	0,00045616	MYL9
ENSGALG00000001998	1765,57571	0,514536405	0,13830499	3,72030241	0,00019898	0,00135687	R3HDM4
ENSGALG00000012089	5360,59071	0,513995299	0,12096528	4,24911439	2,15E-05	0,00019918	C14orf37
ENSGALG00000013708	5397,09415	0,513478256	0,13150987	3,90448458	9,44E-05	0,00071344	CYB5A
ENSGALG00000029159	2974,1732	0,51305649	0,12916503	3,97210068	7,12E-05	0,00056002	
ENSGALG00000005901	2799,23507	0,510684045	0,13243521	3,85610467	0,00011521	0,00084513	PCMTD2
ENSGALG00000023786	1008,22899	0,510469642	0,16220256	3,14711206	0,00164892	0,00803796	
ENSGALG00000017053	1433,26653	0,50974214	0,14651068	3,47921488	0,00050289	0,00301355	SPG20

ENSGALG00000004741	5108,39092	0,509578858	0,14467631	3,52219976	0,00042798	0,00263783	EPHB2
ENSGALG00000012644	1843,51714	0,509005651	0,13724043	3,70886091	0,00020819	0,00141445	
ENSGALG00000029036	2011,06708	0,506773529	0,16255236	3,11760184	0,00182329	0,00871938	
ENSGALG00000011324	77589,574	0,50624037	0,10393882	4,8705612	1,11E-06	1,44E-05	
ENSGALG00000005757	2287,1727	0,505955901	0,14803896	3,41772137	0,00063148	0,00363995	IRF8
ENSGALG00000012670	2710,27627	0,505329169	0,13956275	3,62080263	0,00029369	0,00190591	
ENSGALG00000028145	4237,96065	0,505320547	0,15549944	3,24966153	0,00115542	0,00602451	
ENSGALG00000027891	10377,4455	0,504324315	0,10846652	4,64958504	3,33E-06	3,83E-05	
ENSGALG00000024295	3217,43815	0,504249612	0,13242093	3,80792978	0,00014014	0,00100577	
ENSGALG00000008631	3955,10092	0,503418102	0,13183167	3,81864307	0,00013419	0,00096819	TYRO3
ENSGALG00000005811	1644,05046	0,503226947	0,1577089	3,19085955	0,0014185	0,00712916	
ENSGALG00000025977	2572,61094	0,502103804	0,12961035	3,87394827	0,00010709	0,00079659	GADD45A
ENSGALG00000024398	21096,2288	0,500531072	0,11622841	4,30644352	1,66E-05	0,0001594	RPS28
ENSGALG00000004851	3943,15715	0,500191453	0,14446169	3,4624504	0,00053528	0,00317415	
ENSGALG00000008341	3732,2674	0,499807137	0,12743693	3,92199613	8,78E-05	0,00067247	FURIN
ENSGALG00000011326	2703,92213	0,4983963	0,1426307	3,49431285	0,00047528	0,00287595	STK16
ENSGALG00000000842	2625,68946	0,49824929	0,14971545	3,3279751	0,0008748	0,0047852	
ENSGALG00000007370	4047,93897	0,4969378	0,12075859	4,11513402	3,87E-05	0,00033124	CLP1
ENSGALG00000012947	2224,14952	0,495800698	0,13770491	3,60045778	0,00031766	0,00203909	
ENSGALG00000013067	8815,63453	0,494753671	0,11635485	4,25211024	2,12E-05	0,00019721	
ENSGALG00000000527	6471,78085	0,494652161	0,11959043	4,13621848	3,53E-05	0,00030619	
ENSGALG00000012540	1788,92176	0,494557337	0,13808033	3,58166386	0,00034141	0,00217314	RBFOX2
ENSGALG00000011778	2592,19321	0,494085012	0,14681864	3,36527439	0,00076468	0,00428615	
ENSGALG00000027824	1654,69459	0,493225579	0,14677677	3,36037904	0,00077836	0,00435387	
ENSGALG00000005897	4766,71418	0,493064333	0,12620964	3,90670892	9,36E-05	0,00070927	HMCES
ENSGALG00000012816	2261,40613	0,492973169	0,15659783	3,14802046	0,0016438	0,00802229	SLC22A23
ENSGALG00000005071	6694,01614	0,492854493	0,11371723	4,33403516	1,46E-05	0,00014402	MPP1
ENSGALG00000000336	3779,81285	0,492268059	0,13944285	3,53024962	0,00041517	0,00257049	PP1M M21 subunit
ENSGALG00000004902	4281,00793	0,491426093	0,11754092	4,18089353	2,90E-05	0,00025805	TOM1L2
ENSGALG00000011894	1400,22178	0,491394089	0,14545816	3,37825034	0,00072949	0,00411775	
ENSGALG00000017076	6428,3314	0,490620681	0,14677761	3,34261258	0,00082994	0,00457043	B3GLCT
ENSGALG00000008409	5336,4487	0,490103959	0,14311325	3,42458839	0,00061573	0,00357339	
ENSGALG00000011531	2214,44297	0,486736013	0,15147264	3,2133593	0,00131192	0,00669232	APAF1
ENSGALG00000006915	1392,13905	0,485309324	0,15555376	3,11988167	0,00180924	0,00866613	
ENSGALG00000000947	2256,10973	0,482847185	0,14942146	3,23144465	0,00123166	0,00635192	
ENSGALG00000014581	2314,87998	0,482286067	0,13822045	3,48925269	0,00048437	0,0029219	BORCS8
ENSGALG00000011738	6621,78437	0,482221251	0,11823294	4,07856946	4,53E-05	0,00037711	ARHGDIB
ENSGALG00000027688	6057,20574	0,481915396	0,11816406	4,07835855	4,54E-05	0,00037722	NDUFB4
ENSGALG00000005713	4508,08916	0,480930735	0,11431181	4,2071834	2,59E-05	0,00023392	NCKIPSD
ENSGALG00000003405	5095,98918	0,480657067	0,11244145	4,27473194	1,91E-05	0,00018043	BSDC1
ENSGALG00000002033	6624,42072	0,480537464	0,11110056	4,32524788	1,52E-05	0,00014817	NDUFB6
ENSGALG00000029135	7403,52828	0,479980361	0,12965053	3,70210873	0,00021381	0,00144758	GAS41
ENSGALG00000011436	3916,77111	0,477975314	0,1192676	4,00758713	6,13E-05	0,00049041	TMBIM1
ENSGALG00000003466	4818,27592	0,477695903	0,13420724	3,55939007	0,00037172	0,00233545	AKR1B1L
ENSGALG00000001606	1726,36227	0,477443775	0,14690617	3,2499912	0,00115409	0,00602026	INPP5B
ENSGALG00000000785	1523,67138	0,476932316	0,14689834	3,24668282	0,00116758	0,00607411	HYAL1
ENSGALG00000014765	2914,68173	0,476665637	0,12457546	3,82632052	0,00013007	0,00094149	TRIM23
ENSGALG00000000943	4846,50588	0,473716041	0,11278203	4,20027936	2,67E-05	0,00024021	WDTC1
ENSGALG00000006588	1949,86566	0,473632802	0,13867563	3,41540052	0,00063688	0,00366533	LRRC75B
ENSGALG00000023157	3625,87384	0,472554642	0,14223538	3,32234238	0,00089265	0,00487307	REEP5
ENSGALG00000006702	2572,80575	0,471728545	0,1314835	3,58773935	0,00033356	0,00213052	MFGE8
ENSGALG00000003359	6429,54572	0,469661247	0,12003738	3,91262507	9,13E-05	0,00069482	
ENSGALG00000027377	1588,86326	0,469161575	0,14290687	3,28298835	0,00102713	0,00545838	
ENSGALG00000013511	6097,6262	0,469095656	0,12052644	3,89205602	9,94E-05	0,00074604	ANKRA2
ENSGALG00000003348	1706,40697	0,468370054	0,13812202	3,39098761	0,00069641	0,0039639	
ENSGALG00000028342	3684,92319	0,4672884	0,14052674	3,32526324	0,00088335	0,00482617	
ENSGALG00000004581	7502,73386	0,466855623	0,11693534	3,99242555	6,54E-05	0,00051769	NDST1
ENSGALG00000007238	3294,46471	0,466556765	0,1396865	3,34002762	0,0008377	0,0046082	
ENSGALG00000001556	1997,40951	0,466292104	0,13625952	3,42208821	0,00062142	0,00359569	
ENSGALG00000009655	1343,41122	0,465196113	0,14630894	3,17954671	0,00147506	0,0073322	AADAT
ENSGALG00000002485	4126,42787	0,464623616	0,12758859	3,64157666	0,00027097	0,00177456	PHKG1

ENSGALG00000011474	2398,5188	0,463345487	0,1386209	3,34253707	0,00083016	0,00457043	
ENSGALG00000013698	1914,38769	0,462883571	0,13997841	3,30682108	0,00094361	0,00510015	
ENSGALG00000007582	9089,3986	0,462276159	0,11185396	4,13285464	3,58E-05	0,00030983	DPYSL3
ENSGALG00000003976	1632,25763	0,461042882	0,14423767	3,19641107	0,00139149	0,0070167	BCORL1
ENSGALG00000024391	9415,82069	0,460947942	0,11472094	4,01799317	5,87E-05	0,00047231	CACTIN
ENSGALG00000024034	3571,45047	0,460548751	0,12312106	3,74061704	0,00018357	0,00127126	PI4KB
ENSGALG00000014226	4001,88511	0,460133597	0,13949225	3,29863207	0,00097157	0,00520949	
ENSGALG00000022980	7902,73972	0,459059074	0,1123607	4,08558383	4,40E-05	0,00036679	CDKN1B
ENSGALG00000014324	2616,41443	0,458208088	0,14844789	3,08665942	0,00202419	0,00953942	
ENSGALG00000001881	2571,72261	0,456046301	0,14749617	3,09191952	0,00198867	0,00938825	
ENSGALG00000000921	4858,40124	0,454646053	0,11600766	3,9191038	8,89E-05	0,00067906	
ENSGALG00000004537	5387,82075	0,454616982	0,11547716	3,93685619	8,26E-05	0,00063539	
ENSGALG00000016870	5615,99638	0,454001604	0,11901986	3,81450277	0,00013646	0,00098404	TMTC4
ENSGALG00000027963	10511,1952	0,451771641	0,1053151	4,28971367	1,79E-05	0,00016974	COX7C
ENSGALG00000009054	1742,52432	0,451368563	0,14445222	3,12469106	0,00177992	0,00855102	
ENSGALG00000017351	2392,614	0,45115395	0,12979024	3,47602367	0,00050891	0,0030416	
ENSGALG00000007286	2211,13478	0,450759437	0,14338367	3,14372922	0,0016681	0,00811112	SIRT7
ENSGALG00000001376	9938,31883	0,45012042	0,10543845	4,26903467	1,96E-05	0,00018459	ARID3B
ENSGALG00000017126	2105,45067	0,449836372	0,14456606	3,11163202	0,00186056	0,00886339	
ENSGALG00000004353	9460,54723	0,448918917	0,11266612	3,98450692	6,76E-05	0,00053309	TBCA
ENSGALG00000015780	22777,7944	0,447991765	0,11059391	4,05078157	5,10E-05	0,00041817	PNRC1
ENSGALG00000012218	3810,36969	0,44776649	0,1283039	3,48988984	0,00048322	0,00291753	CBY1
ENSGALG00000008274	1660,02758	0,446767639	0,14203363	3,14550613	0,001658	0,00807066	CRBN
ENSGALG00000005608	3810,24681	0,44662731	0,13008707	3,43329509	0,00059629	0,00347391	SLC9A3R2
ENSGALG00000017329	3706,96729	0,44555284	0,13631333	3,26859342	0,00108083	0,00570371	FCHSD2
ENSGALG00000015987	4807,19259	0,445391941	0,12455693	3,57581017	0,00034914	0,00221571	
ENSGALG00000000067	2534,63139	0,444870546	0,12860379	3,45923359	0,00054171	0,00320811	SPR
ENSGALG00000011921	3797,77855	0,444716768	0,14148109	3,14329478	0,00167058	0,00812027	DHRS7
ENSGALG00000027337	6395,16083	0,441351335	0,11482343	3,84373949	0,00012117	0,00088176	
ENSGALG00000004907	2377,26928	0,441232207	0,13371969	3,29968027	0,00096795	0,00519663	KCTD15
ENSGALG00000017096	2040,45459	0,440278375	0,13379312	3,29073997	0,00099924	0,00532681	
ENSGALG00000012043	13548,0358	0,439717314	0,10426627	4,21725362	2,47E-05	0,00022551	
ENSGALG00000012265	3656,88094	0,439419657	0,1289098	3,40873745	0,00065264	0,00373978	MICALL1
ENSGALG00000002986	2686,67203	0,439006087	0,12512502	3,50853962	0,00045057	0,00274477	
ENSGALG00000005820	8527,7428	0,438136271	0,11284846	3,88251872	0,00010338	0,00077336	RNF130
ENSGALG00000026116	1987,2825	0,437155594	0,13561904	3,22340863	0,00126675	0,00649596	
ENSGALG00000013091	2461,62995	0,436802578	0,1334788	3,27244902	0,0010662	0,00564179	
ENSGALG00000008444	16562,7434	0,436671101	0,11266409	3,87586767	0,00010625	0,00079262	TUBAL3
ENSGALG00000012821	60743,3322	0,436526282	0,11022595	3,96028605	7,49E-05	0,00058241	
ENSGALG00000006399	2888,16792	0,435019992	0,12865618	3,38125997	0,00072154	0,00408283	MESDC1
ENSGALG00000010134	2529,19577	0,434710794	0,12612196	3,44674955	0,00056737	0,00333397	ZFAND3
ENSGALG00000016059	2071,29674	0,434358409	0,13768426	3,15474273	0,0016064	0,0078758	ETS2
ENSGALG00000021365	7390,77997	0,432965219	0,11720328	3,69413922	0,00022063	0,00148635	DCTN3
ENSGALG00000003447	2587,36522	0,432921053	0,13816024	3,1334707	0,00172752	0,00835531	
ENSGALG00000004725	7054,54942	0,431307897	0,12989304	3,32048515	0,00089861	0,00489384	
ENSGALG00000000551	4770,60214	0,428841133	0,11757382	3,64742036	0,00026489	0,00174139	C6orf89
ENSGALG00000023612	4284,86865	0,427551312	0,12223705	3,4977227	0,00046925	0,00284327	LZTS2
ENSGALG00000010838	5580,71709	0,427079258	0,13104043	3,25914125	0,0011175	0,00585859	
ENSGALG00000015304	5360,75819	0,425237788	0,11685875	3,63890423	0,0002738	0,00179049	SNX3
ENSGALG00000020528	5739,52001	0,424819785	0,11091716	3,83006366	0,00012811	0,00092926	INIP
ENSGALG00000015603	3809,74973	0,424497436	0,13385318	3,171366	0,00151724	0,00750352	ZCCHC9
ENSGALG00000026692	7703,25537	0,424103985	0,13618907	3,11408229	0,00184518	0,00880243	NRAS
ENSGALG00000012009	3507,12317	0,422977808	0,12358938	3,42244456	0,00062061	0,0035925	JKAMP
ENSGALG00000027710	12308,995	0,421872733	0,10758636	3,92124726	8,81E-05	0,00067418	
ENSGALG00000000436	3616,92165	0,421563067	0,1280766	3,29149164	0,00099658	0,0053161	RABIF
ENSGALG00000008613	2544,37784	0,420884082	0,1331062	3,16201722	0,0015668	0,00772899	NDUFA1
ENSGALG00000007054	5550,73084	0,419262331	0,12272606	3,41624533	0,00063491	0,00365664	TBC1D16
ENSGALG00000017675	6606,96745	0,417757976	0,11206995	3,72765371	0,00019327	0,00132563	
ENSGALG00000008818	5178,18834	0,417517144	0,12931442	3,22869741	0,00124355	0,00640113	IDH1
ENSGALG00000026394	6922,09548	0,417110916	0,11027253	3,78254597	0,00015523	0,00109906	
ENSGALG00000007220	48744,1762	0,415111704	0,10489641	3,95734916	7,58E-05	0,00058794	FTH1
ENSGALG00000001816	2247,2353	0,414935997	0,13272784	3,12621686	0,00177071	0,00851282	MAGI3

ENSGALG00000026802	39202,809	0,413746284	0,11461572	3,60985629	0,00030637	0,00197874	
ENSGALG00000015915	2924,59886	0,413737551	0,13087631	3,16128683	0,00157074	0,00773719	
ENSGALG00000012426	3567,37244	0,412512543	0,12505498	3,29864939	0,00097151	0,00520949	DDHD1
ENSGALG00000001201	7498,13604	0,411468635	0,10775001	3,81873406	0,00013414	0,00096819	GFOD2
ENSGALG00000024081	3055,10774	0,410810718	0,12976304	3,16585318	0,00154629	0,0076361	TGIF2
ENSGALG00000003750	7887,37064	0,409049332	0,1094937	3,73582537	0,0001871	0,00129184	PLCG1
ENSGALG00000008597	7184,86692	0,404910294	0,11988781	3,37740998	0,00073172	0,0041252	MRPL2
ENSGALG00000006505	3147,8767	0,40439474	0,12215456	3,31051698	0,00093124	0,00504931	ZFAND6
ENSGALG00000008143	14658,924	0,402789538	0,10639446	3,78581321	0,00015321	0,00108585	
ENSGALG00000003924	3485,26038	0,402077112	0,12622532	3,18539184	0,00144558	0,00723588	ACBD6
ENSGALG00000008508	3096,67282	0,401158456	0,12032341	3,33400181	0,00085606	0,00469403	PYGB
ENSGALG00000010704	15057,8876	0,401049242	0,10647647	3,76655286	0,00016552	0,00116401	
ENSGALG00000003371	6971,35561	0,400787786	0,11743488	3,4128513	0,00064287	0,00369623	
ENSGALG00000001096	5395,30498	0,399476875	0,12102428	3,30079943	0,0009641	0,00518412	
ENSGALG00000003412	4305,48949	0,398169298	0,11885899	3,34992998	0,00080832	0,00447915	
ENSGALG00000014970	13935,5595	0,397421157	0,10749714	3,69703948	0,00021813	0,00147386	FSTL1
ENSGALG00000003422	9411,16928	0,397187449	0,11024485	3,60277551	0,00031484	0,00202385	
ENSGALG00000001572	4170,73825	0,394480266	0,11780929	3,34846484	0,00081261	0,00449376	COMMD4
ENSGALG00000028470	5093,19173	0,39443368	0,12418131	3,17627247	0,00149181	0,00740195	
ENSGALG00000021047	9190,88912	0,393972396	0,12289699	3,20571241	0,00134729	0,00683431	KIFAP3
ENSGALG00000010490	10248,5665	0,389494511	0,11178754	3,4842389	0,00049354	0,00296799	DPYSL4
ENSGALG00000013000	7034,84966	0,388948819	0,1248778	3,11463547	0,00184172	0,00878902	
ENSGALG00000000955	5256,56158	0,388613492	0,11456174	3,39217531	0,0006934	0,00395006	TMEM222
ENSGALG00000015311	2435,46157	0,387705603	0,12617408	3,07278335	0,00212072	0,009926	RNF38
ENSGALG00000004011	4239,17688	0,385892717	0,12240581	3,15256855	0,00161841	0,00792043	
ENSGALG00000005337	6236,10371	0,385644449	0,10969262	3,51568283	0,00043862	0,00268278	SNX2
ENSGALG00000004086	6417,78519	0,385423377	0,12458188	3,09373553	0,00197654	0,00935043	
ENSGALG00000026102	19802,423	0,385336727	0,12172597	3,1656082	0,00154759	0,00763976	
ENSGALG00000016639	3515,28691	0,384196385	0,12279677	3,1287174	0,00175571	0,00846159	
ENSGALG00000026188	5641,89638	0,384179427	0,11525662	3,33325247	0,00085837	0,00470479	
ENSGALG00000008736	6339,84871	0,384117634	0,1174006	3,27185401	0,00106845	0,00564929	PNPLA7
ENSGALG00000008297	4909,6132	0,379216929	0,11488546	3,30082615	0,00096401	0,00518412	SEMA4B
ENSGALG00000007205	14498,1893	0,37857906	0,10495892	3,60692592	0,00030985	0,00199614	
ENSGALG00000028128	3864,66289	0,378077792	0,11727019	3,22398887	0,00126418	0,00648525	
ENSGALG00000015436	20607,2425	0,377556464	0,11268107	3,35066444	0,00080618	0,00447275	SDCBP
ENSGALG00000002053	9601,26321	0,376194926	0,11159082	3,37119972	0,00074842	0,00420366	
ENSGALG00000004191	5196,69877	0,374652449	0,11676506	3,20860067	0,00133383	0,0067774	RIC8A
ENSGALG00000003717	5316,70071	0,373543889	0,11309691	3,30286557	0,00095702	0,00515652	MYO9B
ENSGALG00000026291	5117,15968	0,37266418	0,11704722	3,18387886	0,00145316	0,00725779	
ENSGALG00000026421	20179,8154	0,372144283	0,11694539	3,18220567	0,00146158	0,00728649	PSMB2
ENSGALG00000006857	3516,26088	0,36847671	0,11911862	3,09335936	0,00197904	0,00935579	SPATA20
ENSGALG00000028249	8201,39743	0,366515679	0,10750136	3,40940496	0,00065105	0,00373536	
ENSGALG00000021692	6544,92461	0,361385734	0,11652843	3,10126669	0,00192695	0,00914762	
ENSGALG00000007589	8938,41629	0,360072922	0,10858113	3,31616491	0,00091262	0,00496041	
ENSGALG00000011435	8069,4783	0,35897775	0,11230455	3,19646662	0,00139122	0,0070167	
ENSGALG00000001330	11236,9836	0,358718136	0,11464643	3,12890804	0,00175457	0,00846159	ATP5G1
ENSGALG00000026517	6681,39607	0,355711572	0,1112537	3,1973011	0,0013872	0,00700287	RFC5
ENSGALG00000023073	19350,4834	0,353608475	0,10573559	3,34427114	0,00082499	0,00454598	
ENSGALG00000026565	5799,2794	0,352044819	0,11138453	3,16062577	0,00157431	0,00775196	
ENSGALG00000012229	22179,4003	0,350504617	0,10217867	3,43031096	0,00060289	0,00350934	RPS29
ENSGALG00000010347	11028,0806	0,346318448	0,1080262	3,20587467	0,00134653	0,0068343	CCNG2
ENSGALG00000026300	11402,689	0,345400821	0,1081778	3,19289927	0,00140852	0,00708947	
ENSGALG00000023447	10483,1653	0,344836567	0,10464307	3,29535984	0,00098296	0,00525853	
ENSGALG00000013784	13631,601	0,339967639	0,10798288	3,14834763	0,00164196	0,00802129	CDT1
ENSGALG00000015998	9692,12229	0,339150714	0,10588553	3,20299389	0,00136007	0,00689145	
ENSGALG00000024261	6604,44476	0,336614329	0,10865736	3,09794308	0,00194869	0,00924117	
ENSGALG00000000905	28204,5829	0,330518761	0,10726487	3,0813327	0,00206076	0,00967826	PSMB4
ENSGALG00000007272	8940,93056	0,327852784	0,10564224	3,10342513	0,00191295	0,00909384	RNF166
ENSGALG00000008443	36760,133	0,321038212	0,10104157	3,17728858	0,00148659	0,00737876	ARHGDIA
ENSGALG00000015664	24898,2726	0,319058165	0,10032221	3,18033435	0,00147105	0,00732031	ADD1
ENSGALG00000012546	35449,6881	0,318249627	0,09997702	3,18322776	0,00145643	0,0072688	MCM5
ENSGALG00000011238	46142,4631	0,316616268	0,10217962	3,09862447	0,00194421	0,00922316	WLS

ENSGALG00000020078	34717,1709	0,313921783	0,09887982	3,17478105	0,0014995	0,00743198	
ENSGALG00000028600	142500,088	0,313870865	0,09855851	3,18461456	0,00144947	0,00725001	
ENSGALG00000010961	89733,2273	-0,310634298	0,10095703	-3,076896	0,00209168	0,00980319	IGF2BP3
ENSGALG00000003815	34063,9206	-0,31144656	0,10097526	-3,0843849	0,00203974	0,00959938	RPN2
ENSGALG00000004375	15903,9039	-0,317187722	0,10291656	-3,0819891	0,00205622	0,00966361	VPS35
ENSGALG00000010748	12682,3509	-0,319116199	0,1031995	-3,0922261	0,00198661	0,00938507	EXO1
ENSGALG00000005668	17427,2491	-0,321274618	0,10214198	-3,145373	0,00165875	0,00807145	WSB1
ENSGALG00000001817	20809,4267	-0,321828859	0,10400477	-3,0943663	0,00197234	0,00933381	CTCF
ENSGALG00000000235	25074,3258	-0,321994098	0,10263119	-3,1373902	0,00170459	0,00826202	PPP2R2A
ENSGALG00000010178	15181,2565	-0,322485727	0,10328298	-3,1223512	0,00179413	0,00861017	PUM3
ENSGALG00000005758	19288,8311	-0,323694502	0,10277128	-3,149659	0,00163461	0,00799398	
ENSGALG00000006477	10679,2726	-0,324083678	0,10402567	-3,1154203	0,00183683	0,00877181	
ENSGALG00000002222	13238,2952	-0,324594377	0,10274095	-3,1593475	0,00158123	0,00777748	NOC2L
ENSGALG00000000814	43324,2546	-0,325022823	0,09923062	-3,2754289	0,00105502	0,00558913	HNRNPR
ENSGALG00000014575	10529,4114	-0,325103377	0,10553605	-3,0804959	0,00206656	0,00969881	SEPHS1
ENSGALG00000004780	17002,1156	-0,326184617	0,10404378	-3,1350709	0,00171813	0,0083217	
ENSGALG00000011091	12877,9103	-0,326829517	0,1036675	-3,152671	0,00161784	0,00792043	NUP133
ENSGALG00000001262	15946,3219	-0,32743844	0,10659706	-3,0717398	0,00212815	0,00994712	CLIC4
ENSGALG00000004161	24484,0776	-0,327725215	0,10319068	-3,1759188	0,00149363	0,00740828	CCNB2
ENSGALG00000014286	15937,5586	-0,329230926	0,10635463	-3,0955955	0,00196418	0,00930168	
ENSGALG00000002062	16195,7787	-0,329944458	0,10510199	-3,139279	0,00169364	0,00821945	SRP68
ENSGALG00000001962	54240,9734	-0,330389801	0,10379904	-3,1829756	0,0014577	0,00727247	PTBP1
ENSGALG00000010576	13964,8988	-0,330632554	0,10260922	-3,2222498	0,00127188	0,00651983	GTF2A1
ENSGALG00000010481	12612,5746	-0,330642085	0,10591126	-3,1218786	0,00179701	0,00862096	CMPK1
ENSGALG00000010341	71633,3369	-0,33081711	0,10337066	-3,2002997	0,00137285	0,00694328	XPO5
ENSGALG00000007387	14223,8054	-0,330955786	0,10651466	-3,1071383	0,00188908	0,00898666	GTF3C1
ENSGALG00000003547	13605,0703	-0,331189406	0,10621784	-3,1180207	0,0018207	0,00871312	SKP2
ENSGALG00000014445	19258,1997	-0,331954776	0,10745587	-3,0892197	0,00200683	0,00947071	
ENSGALG00000007590	10666,5636	-0,332146464	0,10813379	-3,0716251	0,00212897	0,00994712	MTMR8
ENSGALG00000004108	32339,0897	-0,332634184	0,10036764	-3,3141577	0,0009192	0,00499197	CCAR1
ENSGALG00000007528	28367,4799	-0,33311917	0,10609529	-3,1398111	0,00169057	0,00820866	TCERG1
ENSGALG00000005177	21314,8021	-0,333333735	0,1014573	-3,2854583	0,00101817	0,00541709	
ENSGALG00000001976	9937,86887	-0,333600989	0,10865793	-3,0701945	0,00213919	0,00998662	TRIM33
ENSGALG00000009332	24425,5213	-0,334253024	0,10229188	-3,26764	0,00108448	0,0057141	RBM25
ENSGALG00000014298	14009,4127	-0,335955725	0,10618376	-3,1639087	0,00155666	0,00768172	RFC1
ENSGALG00000003231	28849,9366	-0,336908133	0,1031629	-3,2657877	0,0010916	0,00574493	
ENSGALG00000014327	7067,9269	-0,338948318	0,10818125	-3,1331523	0,0017294	0,00836141	
ENSGALG00000002302	11281,3267	-0,339317581	0,10876186	-3,1198214	0,00180961	0,00866613	
ENSGALG00000005966	13999,7426	-0,340533213	0,11012393	-3,0922727	0,0019863	0,00938507	SEC61A1
ENSGALG00000012202	8130,08702	-0,341191282	0,10806076	-3,1574023	0,00159182	0,00781275	
ENSGALG00000007989	28672,5886	-0,341827011	0,10925868	-3,1286027	0,0017564	0,0084619	ADNP
ENSGALG00000005237	11277,3975	-0,342909039	0,10563626	-3,24613	0,00116985	0,00608154	FGD3
ENSGALG00000016314	22102,7105	-0,343364339	0,10699123	-3,2092755	0,0013307	0,00677035	ELOVL5
ENSGALG00000012239	9046,42319	-0,343596083	0,10589827	-3,2445865	0,00117621	0,0061076	METAP1
ENSGALG00000001236	5792,05952	-0,345759524	0,11038031	-3,1324386	0,00173361	0,00837771	
ENSGALG00000012837	7840,94956	-0,345878883	0,10966121	-3,1540676	0,00161012	0,00788836	EXOC2
ENSGALG00000006475	6864,19423	-0,347016477	0,10794054	-3,2148856	0,00130497	0,00666433	
ENSGALG00000015553	10445,9765	-0,347410904	0,11270003	-3,082616	0,0020519	0,00964994	
ENSGALG00000011831	9083,64488	-0,347478626	0,11085227	-3,1346101	0,00172083	0,00832589	ANKRD50
ENSGALG00000004269	64393,6529	-0,347526191	0,10378498	-3,348521	0,00081244	0,00449376	
ENSGALG00000004200	11378,582	-0,347970886	0,10751163	-3,2365883	0,00120968	0,00626227	DPP9
ENSGALG00000016887	64639,1062	-0,348222743	0,09787488	-3,5578355	0,00037392	0,00234499	
ENSGALG00000013649	18631,1873	-0,348311839	0,10201524	-3,4143117	0,00063943	0,00367803	CNOT7
ENSGALG00000017139	10651,9101	-0,349554954	0,10704704	-3,265433	0,00109297	0,00574991	
ENSGALG00000002719	17659,4459	-0,349571816	0,10180837	-3,4336254	0,00059557	0,00347116	CSNK1D
ENSGALG00000008454	36878,682	-0,351028215	0,10381886	-3,3811602	0,0007218	0,00408283	NOP58
ENSGALG00000017026	9366,38767	-0,352330056	0,10866839	-3,2422496	0,0011859	0,00615322	VPS36
ENSGALG00000007861	11084,8862	-0,352469383	0,10824618	-3,2561832	0,00112921	0,0059177	MAGT1
ENSGALG00000005569	11340,4378	-0,352585966	0,10935005	-3,2243787	0,00126246	0,00648	USP47
ENSGALG00000009913	62516,8642	-0,353547892	0,09880506	-3,5782367	0,00034592	0,00219771	SMARCA5
ENSGALG00000011620	9603,38362	-0,353615473	0,10554783	-3,3502865	0,00080728	0,00447521	AKT1
ENSGALG00000010177	12046,3189	-0,353917428	0,10807412	-3,2747656	0,0010575	0,00560008	ZFC3H1

ENSGALG00000015043	11145,3654	-0,35411601	0,10996307	-3,2203176	0,00128049	0,006559	
ENSGALG00000008036	5782,62739	-0,355406874	0,1116416	-3,1834627	0,00145525	0,00726557	TMEM209
ENSGALG00000005125	7959,32707	-0,356020767	0,10674059	-3,3353833	0,00085182	0,00467642	LSM14B
ENSGALG00000001811	14773,9745	-0,357025162	0,11419157	-3,1265456	0,00176873	0,00851143	
ENSGALG00000008957	22456,5253	-0,357191503	0,11566926	-3,0880418	0,0020148	0,00949845	MSH6
ENSGALG00000009330	6639,49466	-0,358006963	0,1142065	-3,1347337	0,0017201	0,00832534	DEGS1
ENSGALG00000010763	6453,8983	-0,358059956	0,11268149	-3,1776289	0,00148485	0,00737279	TRIP11
ENSGALG00000008507	23518,146	-0,358201653	0,10237686	-3,4988536	0,00046726	0,00283376	THOC2
ENSGALG00000009839	30979,5758	-0,358257804	0,10121654	-3,5395182	0,00040086	0,00249096	PPIG
ENSGALG00000010349	7048,15428	-0,358393493	0,11290442	-3,1743089	0,00150194	0,00744137	POLR1C
ENSGALG00000007721	11622,16	-0,358457512	0,10981787	-3,2641091	0,00109809	0,00577239	ARHGAP21
ENSGALG00000005694	30867,6614	-0,359162398	0,10218229	-3,5149181	0,00043989	0,00268931	GARS
ENSGALG00000008254	17757,2659	-0,35964551	0,10795091	-3,3315652	0,00086359	0,0047277	
ENSGALG00000007683	31803,1359	-0,359759566	0,1106419	-3,251567	0,00114771	0,00599848	
ENSGALG00000004670	7571,3318	-0,360055666	0,10915595	-3,2985436	0,00097188	0,00520949	
ENSGALG00000024039	12181,8658	-0,36099515	0,10495725	-3,4394494	0,0005829	0,00340658	
ENSGALG00000004379	20460,5219	-0,361272684	0,10336925	-3,4949725	0,00047411	0,00287042	BCL7A
ENSGALG00000012763	12297,8834	-0,362574608	0,10840365	-3,3446715	0,0008238	0,00454275	GNPTAB
ENSGALG00000001942	6053,36315	-0,362623052	0,11586077	-3,1298174	0,00174915	0,00844792	SPDL1
ENSGALG00000012916	8111,10575	-0,362729901	0,10919421	-3,3218786	0,00089414	0,00487922	DROSHA
ENSGALG00000002139	98917,1679	-0,362887161	0,10160459	-3,5715625	0,00035486	0,00224229	CSDE1
ENSGALG00000009325	21860,6433	-0,363294006	0,11100817	-3,2726781	0,00106534	0,00563941	
ENSGALG00000003826	8394,88602	-0,363869123	0,10711719	-3,3969255	0,00068148	0,00389027	ANAPC7
ENSGALG00000001790	14734,6451	-0,363943316	0,1104661	-3,2946155	0,00098556	0,00526834	
ENSGALG00000004553	9745,08279	-0,363967467	0,10494768	-3,4680848	0,00052418	0,00312057	SGPL1
ENSGALG00000001663	10259,5734	-0,364202029	0,10990467	-3,3137995	0,00092037	0,00499637	MAK16
ENSGALG00000010900	12043,9228	-0,364291872	0,11281721	-3,2290452	0,00124204	0,00640061	SPC25
ENSGALG00000003563	13287,7659	-0,364389392	0,11010466	-3,3094822	0,00093469	0,00506145	YARS
ENSGALG00000002137	15220,8927	-0,364569255	0,10476883	-3,4797493	0,00050188	0,00300887	PGAM5
ENSGALG00000001126	13435,9211	-0,365650894	0,10394749	-3,5176502	0,00043539	0,00266657	CHAF1A
ENSGALG00000015874	13228,0992	-0,365980992	0,10796169	-3,3899153	0,00069914	0,00397612	TTK
ENSGALG00000003339	10767,0615	-0,366040447	0,11772436	-3,1093007	0,00187531	0,00892426	MTOR
ENSGALG00000010400	7431,8392	-0,366106792	0,10709024	-3,4186756	0,00062927	0,00363171	
ENSGALG00000014867	5553,43093	-0,366420731	0,11739665	-3,1212197	0,00180104	0,00863589	PAIP1
ENSGALG00000006193	20925,4656	-0,366551091	0,11528348	-3,179563	0,00147497	0,0073322	
ENSGALG00000005353	4596,56596	-0,367159893	0,11954477	-3,0713171	0,00213117	0,00995398	FAR1
ENSGALG00000015683	11433,1929	-0,367498221	0,1101651	-3,335886	0,00085028	0,00466985	GNG10
ENSGALG00000003171	20304,0548	-0,367614474	0,10192128	-3,6068472	0,00030994	0,00199614	
ENSGALG00000028828	22676,6926	-0,367651199	0,10280751	-3,5761124	0,00034874	0,00221461	
ENSGALG00000024159	24561,8039	-0,368265263	0,10651918	-3,4572673	0,00054568	0,0032246	
ENSGALG00000003881	9972,69972	-0,368271023	0,11805058	-3,1196037	0,00181095	0,00866949	ERCC6L
ENSGALG00000001619	9403,98709	-0,368532524	0,1142023	-3,2270149	0,00125089	0,00643402	ATG16L1
ENSGALG00000004115	26356,0947	-0,369026879	0,10774308	-3,4250635	0,00061466	0,00356867	DNAJA2
ENSGALG00000012266	5845,05186	-0,369135559	0,11234414	-3,2857571	0,00101709	0,00541346	
ENSGALG00000008526	11139,8409	-0,369313202	0,10883113	-3,3934519	0,00069018	0,00393499	
ENSGALG00000001485	7897,80461	-0,369653264	0,10794906	-3,4243306	0,00061632	0,00357526	
ENSGALG00000012263	9464,40856	-0,36996383	0,10644059	-3,4757776	0,00050937	0,00304173	NEMF
ENSGALG00000007509	10510,1509	-0,37005709	0,10740052	-3,4455801	0,00056983	0,00334551	
ENSGALG00000008807	7903,608	-0,370129883	0,10704402	-3,4577352	0,00054474	0,0032218	DCUN1D1
ENSGALG00000014582	21606,21	-0,370326496	0,10926121	-3,3893683	0,00070054	0,00398239	CADM1
ENSGALG00000010296	6483,94047	-0,370634079	0,11535942	-3,2128637	0,00131419	0,00670137	MRPS18A
ENSGALG00000011215	4111,7689	-0,370718656	0,11588474	-3,1990291	0,00137891	0,00696619	
ENSGALG00000004254	3591,44306	-0,370755667	0,11847879	-3,1292998	0,00175223	0,00845981	
ENSGALG00000015649	13211,5423	-0,370890117	0,10670353	-3,4758935	0,00050915	0,00304173	NOP14
ENSGALG00000008557	15377,869	-0,371010327	0,10757228	-3,4489398	0,00056279	0,00331419	ARL5B
ENSGALG00000005935	6194,93972	-0,371684146	0,110711	-3,3572469	0,00078723	0,00439447	
ENSGALG00000004834	12710,539	-0,372460145	0,10851246	-3,4324184	0,00059822	0,00348367	COPS3
ENSGALG00000016578	7057,34653	-0,372483757	0,11837004	-3,1467739	0,00165083	0,00804438	
ENSGALG00000003039	8776,91996	-0,372512284	0,10936842	-3,4060314	0,00065915	0,00377229	SRM
ENSGALG00000015705	13014,2962	-0,372615903	0,12101628	-3,0790561	0,00207658	0,00974245	WHSC1
ENSGALG00000011713	7918,94572	-0,372947939	0,11560014	-3,2261893	0,0012545	0,00644774	
ENSGALG00000000412	5015,41988	-0,373361237	0,1191944	-3,1323723	0,001734	0,00837771	

ENSGALG00000003942	9873,26393	-0,373719716	0,10787221	-3,4644669	0,00053128	0,00315594	LONP2
ENSGALG00000016236	30922,9868	-0,374432078	0,11350948	-3,2986854	0,00097139	0,00520949	
ENSGALG00000005618	12381,3407	-0,374714579	0,10648923	-3,5188027	0,0004335	0,002661	CTR9
ENSGALG00000010744	5078,21857	-0,375014992	0,11759876	-3,1889367	0,00142797	0,00716617	GALNT7
ENSGALG00000009626	5852,86848	-0,375704266	0,12017862	-3,1262155	0,00177072	0,00851282	THBS1
ENSGALG00000009677	24959,7858	-0,376298569	0,10417999	-3,6120043	0,00030384	0,00196335	UBP
ENSGALG00000004867	10358,5874	-0,376546151	0,10686348	-3,5236186	0,0004257	0,00262613	
ENSGALG00000000085	18552,0192	-0,37666785	0,10734971	-3,5087925	0,00045015	0,00274339	
ENSGALG00000011281	6523,20431	-0,37690737	0,11786547	-3,1977759	0,00138492	0,00699394	
ENSGALG00000008247	7600,6846	-0,377692943	0,10953801	-3,4480537	0,00056464	0,00332221	ARFGAP2
ENSGALG00000005006	11070,7539	-0,377708377	0,10759882	-3,5103394	0,00044754	0,00273115	UBA2
ENSGALG00000000978	4782,69426	-0,377763607	0,11791314	-3,2037448	0,00135653	0,00687606	CEP104
ENSGALG00000002985	14604,5417	-0,378678888	0,10567575	-3,5834037	0,00033915	0,00216174	UTP18
ENSGALG00000016851	8342,75174	-0,378897954	0,12010288	-3,1547783	0,0016062	0,0078758	
ENSGALG00000008195	5727,11476	-0,378910892	0,11315331	-3,3486506	0,00081206	0,00449376	NPHP1
ENSGALG00000024047	6900,30697	-0,380223912	0,11575902	-3,2846159	0,00102121	0,00543119	MYCL
ENSGALG00000007036	8649,57445	-0,380541953	0,10600071	-3,5899944	0,00033069	0,00211673	PITRM1
ENSGALG00000005573	47811,3213	-0,380592325	0,10049182	-3,7872967	0,0001523	0,00108107	
ENSGALG00000008187	22960,7008	-0,380821067	0,1023642	-3,7202565	0,00019902	0,00135687	EIF3J
ENSGALG00000000864	4682,06821	-0,380939683	0,11598398	-3,2844163	0,00102194	0,00543291	FBXW2
ENSGALG00000004696	6529,78459	-0,381141883	0,10996498	-3,4660297	0,0005282	0,0031404	LEO1
ENSGALG00000011330	86092,558	-0,381207584	0,11735831	-3,2482368	0,00116123	0,00605055	DYNC1H1
ENSGALG00000012953	7564,61929	-0,381744469	0,11014956	-3,4656922	0,00052887	0,00314297	IPO8
ENSGALG00000007559	4279,21636	-0,381904484	0,1214524	-3,1444788	0,00166383	0,00809326	PDSS1
ENSGALG00000008587	12026,0733	-0,382953864	0,1040771	-3,679521	0,00023367	0,0015626	NDUFAF1
ENSGALG00000001596	12885,5925	-0,383722949	0,10902521	-3,51958	0,00043223	0,00265657	MLLT1
ENSGALG00000004839	5677,61086	-0,383869776	0,11257961	-3,409763	0,00065019	0,00373203	GPATCH1
ENSGALG00000002346	7982,38435	-0,383971209	0,11217493	-3,422968	0,00061941	0,00358865	CLSPN
ENSGALG00000016040	7227,33788	-0,384424184	0,11393306	-3,3741233	0,00074051	0,00416572	
ENSGALG00000011881	17920,0092	-0,384788496	0,10123697	-3,8008694	0,00014419	0,0010289	CCNA2
ENSGALG00000017105	3524,63272	-0,385190009	0,11906547	-3,235111	0,00121595	0,0062852	RNF6
ENSGALG00000015010	9588,37587	-0,38521292	0,11536985	-3,3389394	0,00084099	0,00462442	
ENSGALG00000015040	4593,81042	-0,38608617	0,11721149	-3,2939277	0,00098798	0,00527917	SLC16A10
ENSGALG00000013061	7957,2214	-0,38617661	0,11279114	-3,4238206	0,00061747	0,00358045	PAPD7
ENSGALG00000011357	6554,71543	-0,387039211	0,1110437	-3,4854674	0,00049128	0,0029557	VEZT
ENSGALG00000006259	19676,996	-0,387217866	0,1021071	-3,7922717	0,00014928	0,00106185	
ENSGALG00000027774	12094,1246	-0,387231267	0,11361387	-3,4083098	0,00065367	0,00374408	
ENSGALG00000008280	7969,07752	-0,387606949	0,11109303	-3,4890303	0,00048478	0,00292304	PMPCB
ENSGALG00000012724	10174,9584	-0,387874274	0,1052864	-3,6839923	0,00022961	0,00154105	PAK1IP1
ENSGALG00000015247	7003,54363	-0,387982807	0,10807809	-3,5898377	0,00033088	0,002117	GPR161
ENSGALG00000027262	21153,6502	-0,388074983	0,10134615	-3,8292029	0,00012856	0,00093152	CAB39
ENSGALG00000005575	18548,7794	-0,388395592	0,10808819	-3,5933214	0,00032649	0,00209381	AKAP1
ENSGALG00000010609	3709,28931	-0,388677075	0,12431812	-3,1264715	0,00176918	0,00851143	NDUFAF7
ENSGALG00000005012	6065,17104	-0,388993034	0,12158189	-3,1994324	0,00137698	0,00695904	
ENSGALG00000005904	32126,5427	-0,389017981	0,1104816	-3,5211109	0,00042974	0,0026439	TMED5
ENSGALG00000011103	30281,6359	-0,389199345	0,10207015	-3,8130574	0,00013726	0,0009872	
ENSGALG00000007788	4541,30869	-0,389226557	0,11718497	-3,3214717	0,00089544	0,00488438	ABCB7
ENSGALG00000003166	4550,92062	-0,38962163	0,1147713	-3,3947653	0,00068687	0,0039178	
ENSGALG00000002129	6971,21336	-0,390332447	0,11138482	-3,5043595	0,00045771	0,00278449	WDR47
ENSGALG00000007485	13617,2364	-0,391583137	0,1247391	-3,1392172	0,001694	0,00821945	TFRC
ENSGALG00000008983	6819,43432	-0,391807233	0,11487703	-3,4106665	0,00064804	0,00372126	GNB4
ENSGALG00000009364	15815,3872	-0,391850394	0,10698859	-3,6625439	0,00024972	0,00165578	PRKCI
ENSGALG00000028035	5338,91852	-0,391942986	0,12309173	-3,1841536	0,00145178	0,00725779	
ENSGALG00000017064	15165,1186	-0,392266132	0,10750673	-3,6487589	0,00026351	0,00173402	RFC3
ENSGALG00000013410	8555,69148	-0,392331865	0,10734883	-3,6547383	0,00025744	0,00169986	UBA3
ENSGALG00000003277	6528,68057	-0,393093094	0,11637559	-3,3777968	0,00073069	0,00412281	SUZ12
ENSGALG00000002682	5643,05491	-0,393160876	0,12282679	-3,2009375	0,00136981	0,00693308	BRD3
ENSGALG00000004481	5167,72741	-0,39336646	0,12164503	-3,2337242	0,00122187	0,00630861	
ENSGALG00000015865	4560,35143	-0,393610955	0,11603457	-3,3921868	0,00069337	0,00395006	
ENSGALG00000009569	11508,232	-0,393918558	0,10692863	-3,6839392	0,00022966	0,00154105	TLK1
ENSGALG00000009201	11168,2886	-0,39393497	0,11185482	-3,5218417	0,00042856	0,00263991	
ENSGALG00000028133	7664,26535	-0,394703045	0,11653188	-3,387082	0,0007064	0,00400736	UBE2V2

ENSGALG00000028898	2507,09906	-0,395098922	0,12861106	-3,0720448	0,00212598	0,00994339	CPTP
ENSGALG00000016467	8711,55629	-0,395365049	0,11946994	-3,3093265	0,00093521	0,00506145	SMC6
ENSGALG00000003043	3489,75229	-0,395518744	0,12292663	-3,2175188	0,00129305	0,00661835	
ENSGALG00000002076	11878,1242	-0,395918706	0,11403726	-3,4718362	0,00051691	0,00308268	
ENSGALG00000008521	16274,1776	-0,395949661	0,12761742	-3,1026303	0,00191809	0,00911512	
ENSGALG00000014688	9710,78641	-0,396059354	0,12498284	-3,16891	0,00153012	0,00756173	ZYX
ENSGALG00000004256	8236,22082	-0,396589984	0,11400406	-3,4787357	0,00050379	0,00301496	
ENSGALG00000010643	4501,85662	-0,396627508	0,11751597	-3,3750945	0,0007379	0,00415399	ZYG11B
ENSGALG00000012759	9679,72927	-0,396656849	0,1130301	-3,5093027	0,00044928	0,00274028	NUP37
ENSGALG00000004435	2804,79298	-0,396797105	0,12438966	-3,1899524	0,00142296	0,00714894	RAB27A
ENSGALG00000009608	5107,67868	-0,396825966	0,12732928	-3,1165335	0,00182991	0,0087449	
ENSGALG00000009838	16688,775	-0,397053502	0,10163734	-3,9065711	9,36E-05	0,00070928	AQR
ENSGALG00000009533	7127,79412	-0,397081701	0,12818092	-3,0978221	0,00194948	0,00924172	NMD3
ENSGALG00000015435	3490,23228	-0,397319834	0,12169857	-3,2647865	0,00109547	0,00576082	DCAF6
ENSGALG00000015215	6704,29174	-0,397390834	0,12048616	-3,2982281	0,00097297	0,00521125	MAPRE2
ENSGALG00000013776	3256,83732	-0,397455529	0,12774216	-3,1113889	0,0018621	0,00886759	CEP135
ENSGALG00000002675	3557,32889	-0,397531672	0,12525226	-3,1738483	0,00150432	0,00744777	SCUBE3
ENSGALG00000009322	5962,2024	-0,397973054	0,11357624	-3,5040168	0,0004583	0,00278683	NVL
ENSGALG00000001576	13166,5037	-0,398019961	0,10682503	-3,7259054	0,00019462	0,00133284	
ENSGALG00000008889	7462,94615	-0,398667809	0,10789505	-3,6949593	0,00021992	0,00148303	TTC14
ENSGALG00000010674	9662,45958	-0,399166032	0,11134741	-3,5848703	0,00033725	0,00215164	DCTD
ENSGALG00000006483	5623,94704	-0,399267586	0,12106372	-3,2979954	0,00097378	0,00521352	
ENSGALG00000004368	3768,91401	-0,39990946	0,1253746	-3,1897167	0,00142412	0,00715213	
ENSGALG00000001377	9703,5691	-0,400093128	0,10731064	-3,7283641	0,00019273	0,0013239	POLRMT
ENSGALG00000020251	2658,23573	-0,40015479	0,12471527	-3,2085468	0,00133408	0,0067774	FNIP2
ENSGALG00000007663	14617,8334	-0,400171791	0,11471241	-3,4884785	0,00048578	0,00292778	KDM3B
ENSGALG00000008289	17537,4534	-0,401142997	0,11170507	-3,5910904	0,0003293	0,00210884	RBM19
ENSGALG00000000332	20852,8348	-0,401294712	0,10538837	-3,8077702	0,00014023	0,00100589	
ENSGALG00000016747	15836,0603	-0,401466819	0,10405	-3,8584028	0,00011413	0,00083858	
ENSGALG00000001094	14665,6234	-0,40156915	0,12409768	-3,2359118	0,00121255	0,00627312	ADGRD2
ENSGALG00000011493	4936,29609	-0,401631833	0,1189649	-3,3760533	0,00073534	0,00414216	TMEM169
ENSGALG00000010304	4399,91638	-0,401743756	0,11989741	-3,3507292	0,00080599	0,00447275	MLH3
ENSGALG00000001422	12037,3785	-0,402405365	0,1260368	-3,1927608	0,0014092	0,00709025	
ENSGALG00000005516	8167,22794	-0,402406965	0,10796988	-3,7270299	0,00019375	0,00132825	HEATR6
ENSGALG00000012138	29874,2309	-0,402996238	0,10409408	-3,8714616	0,00010818	0,00080225	
ENSGALG00000011379	3502,29926	-0,403264726	0,12185227	-3,3094561	0,00093477	0,00506145	SLC44A5
ENSGALG00000004486	7663,27363	-0,403537703	0,11899283	-3,3912775	0,00069568	0,00396137	ZCCHC8
ENSGALG00000011505	19437,7359	-0,403647787	0,1041549	-3,8754564	0,00010643	0,00079353	MARK3
ENSGALG00000007610	8753,25449	-0,403710794	0,10808926	-3,734976	0,00018773	0,00129417	CDC23
ENSGALG00000003219	2817,88024	-0,403748967	0,12502192	-3,2294255	0,00124039	0,00639452	
ENSGALG00000003033	4208,03288	-0,4040823	0,12394506	-3,2601726	0,00111344	0,00583958	
ENSGALG00000009887	7499,04151	-0,404219345	0,11161371	-3,6215922	0,0002928	0,00190191	DIEXF
ENSGALG00000016070	5235,92577	-0,404284672	0,11391617	-3,5489664	0,00038675	0,00240876	WRB
ENSGALG00000005558	18603,5366	-0,404537586	0,10210074	-3,9621417	7,43E-05	0,0005792	TBL3
ENSGALG00000003358	9790,28902	-0,404595333	0,1105122	-3,6610919	0,00025114	0,0016639	UBIAD1
ENSGALG00000010428	7926,19447	-0,404704607	0,10820488	-3,7401697	0,0001839	0,00127223	FAM98A
ENSGALG00000012509	6088,11777	-0,40489721	0,11727746	-3,4524724	0,00055547	0,00327393	
ENSGALG00000016034	11941,1216	-0,4051791	0,11639003	-3,4812184	0,00049914	0,00299769	ATRN
ENSGALG00000011405	5639,0186	-0,405273633	0,11718169	-3,4585065	0,00054318	0,00321538	SLC4A7
ENSGALG00000002042	9110,02017	-0,405517273	0,11009443	-3,6833588	0,00023018	0,00154304	
ENSGALG00000011118	5098,98573	-0,405615226	0,11354185	-3,5723853	0,00035374	0,00224011	MIER1
ENSGALG00000004300	11426,3375	-0,405827457	0,10825338	-3,7488663	0,00017764	0,00123772	PTDSS2
ENSGALG00000002107	25522,2013	-0,406162719	0,10189792	-3,9859767	6,72E-05	0,00053042	
ENSGALG00000011571	3230,47914	-0,406488263	0,12109769	-3,3566972	0,00078879	0,00440141	AGPAT4
ENSGALG00000014404	9644,71084	-0,407214942	0,11242179	-3,6222066	0,0002921	0,00189831	
ENSGALG00000004001	29564,3469	-0,407260268	0,12203749	-3,3371735	0,00084635	0,00465015	
ENSGALG00000000275	5245,75511	-0,407767805	0,11961336	-3,409049	0,0006519	0,00373771	KCTD9
ENSGALG00000013661	4102,81323	-0,40778269	0,12930218	-3,1537185	0,00161205	0,00789496	MICU3
ENSGALG00000009534	2852,25345	-0,408065762	0,12826207	-3,1814998	0,00146515	0,00729626	
ENSGALG00000003143	15625,0529	-0,408123735	0,10675451	-3,8230117	0,00013183	0,0009527	ZC3H7A
ENSGALG00000001616	13431,8281	-0,408530697	0,10735755	-3,8053281	0,00014162	0,00101479	CDC45
ENSGALG00000003995	3806,36291	-0,408610387	0,12214717	-3,3452299	0,00082214	0,00453545	ERAL1

ENSGALG00000011100	3743,98602	-0,408960809	0,12437236	-3,2881968	0,00100831	0,00536886	
ENSGALG00000008523	12531,5109	-0,409027447	0,10500568	-3,8952888	9,81E-05	0,00073778	XPNPEP1
ENSGALG00000012607	5903,92354	-0,409282973	0,12766873	-3,20582	0,00134678	0,0068343	ZCCHC6
ENSGALG00000009483	9325,77474	-0,409303762	0,10929848	-3,7448259	0,00018052	0,0012537	MARK1
ENSGALG00000013858	2841,13225	-0,410414198	0,12330011	-3,3285793	0,0008729	0,00477675	
ENSGALG00000002673	4173,32355	-0,41052388	0,11457704	-3,5829508	0,00033973	0,00216448	GRPEL2
ENSGALG00000016345	4179,81268	-0,410542245	0,1255035	-3,2711616	0,00107107	0,00566094	KLHL15
ENSGALG00000006404	23302,9151	-0,411265326	0,10229476	-4,020395	5,81E-05	0,00046863	DDX46
ENSGALG00000011662	25762,1153	-0,412100057	0,1171723	-3,5170434	0,00043638	0,00267027	
ENSGALG00000006157	6346,9334	-0,412112134	0,11052549	-3,7286613	0,0001925	0,00132315	
ENSGALG00000000780	20148,9101	-0,413093901	0,1091845	-3,7834483	0,00015467	0,00109565	
ENSGALG00000010734	12330,7124	-0,413140268	0,11515498	-3,5876892	0,00033362	0,00213052	PPP4R3A
ENSGALG00000027373	11590,4668	-0,413207621	0,12758753	-3,2386208	0,00120109	0,00622289	
ENSGALG00000014811	9624,08099	-0,413279775	0,10683153	-3,8685189	0,0001095	0,00081033	SMCHD1
ENSGALG00000009300	6311,47255	-0,413699825	0,11749092	-3,5211215	0,00042973	0,0026439	NUMB
ENSGALG00000014978	5258,7361	-0,41400107	0,1181716	-3,5033889	0,00045938	0,00279153	
ENSGALG00000004947	6549,15547	-0,414737624	0,12292028	-3,3740375	0,00074074	0,00416572	POLR3A
ENSGALG00000001613	28424,6252	-0,414922322	0,10503517	-3,9503179	7,80E-05	0,0006041	GNL3
ENSGALG00000013943	3049,80064	-0,414996341	0,12653671	-3,2796518	0,00103935	0,00551258	
ENSGALG00000012241	2635,25809	-0,415005332	0,12984246	-3,1962221	0,0013924	0,0070187	KLHDC1
ENSGALG00000027593	7745,80903	-0,415214085	0,12333575	-3,3665347	0,00076119	0,00426837	
ENSGALG00000000800	3898,75043	-0,415275292	0,12002346	-3,4599509	0,00054027	0,00320097	
ENSGALG00000009634	10679,8347	-0,415471196	0,1072605	-3,8734781	0,00010729	0,00079659	GFM1
ENSGALG00000016972	5736,72836	-0,415815188	0,11849243	-3,5092131	0,00044943	0,00274028	NUFIP1
ENSGALG00000026470	13532,2225	-0,41655555	0,11205019	-3,7175802	0,00020114	0,00136995	BOD1L1
ENSGALG00000003737	3492,31762	-0,417242803	0,13295765	-3,1381631	0,0017001	0,00824613	
ENSGALG00000014386	4214,12952	-0,417751127	0,12151585	-3,4378325	0,00058639	0,00342208	SEPSECS
ENSGALG00000014013	11695,5327	-0,418753457	0,10545846	-3,9707907	7,16E-05	0,00056246	
ENSGALG00000015132	49891,3971	-0,418923213	0,10073836	-4,1585273	3,20E-05	0,00028099	CDH2
ENSGALG00000004121	4190,99049	-0,419252057	0,12228908	-3,4283687	0,00060722	0,00353303	LPAR4
ENSGALG00000009961	27537,6751	-0,419402034	0,1063815	-3,9424339	8,07E-05	0,0006229	RLI
ENSGALG00000010201	4830,06159	-0,41947829	0,13602431	-3,0838479	0,00204342	0,0096134	ABHD18
ENSGALG00000019228	2351,71501	-0,419863222	0,13563777	-3,0954741	0,00196499	0,00930225	SFT2D2
ENSGALG00000007227	17010,7893	-0,420130207	0,12508196	-3,3588394	0,00078271	0,0043764	MSI1
ENSGALG00000000975	4091,69565	-0,420207875	0,11994528	-3,5033297	0,00045948	0,00279153	
ENSGALG00000003985	6692,40436	-0,42022626	0,11526594	-3,6457107	0,00026665	0,00175048	TARSL2
ENSGALG00000007736	8209,23878	-0,420323444	0,11023063	-3,8131273	0,00013722	0,0009872	
ENSGALG00000008989	27837,9247	-0,420708851	0,10920494	-3,852471	0,00011693	0,00085547	
ENSGALG00000017119	1864,30027	-0,420708881	0,13656883	-3,0805629	0,0020661	0,00969881	TNFRSF19
ENSGALG00000010630	14099,6	-0,420914921	0,11268105	-3,7354544	0,00018738	0,00129256	AHCTF1
ENSGALG00000004310	3386,86871	-0,4211147	0,1211839	-3,4750055	0,00051084	0,00304916	TTYH3
ENSGALG00000010671	83381,23	-0,421121888	0,1031007	-4,0845686	4,42E-05	0,00036794	HNRNPU
ENSGALG00000008466	2872,73209	-0,421406429	0,13329064	-3,1615605	0,00156926	0,00773552	
ENSGALG00000014937	3129,4872	-0,421603891	0,12557883	-3,3572847	0,00078712	0,00439447	KPNA5
ENSGALG00000018803	3405,63382	-0,421885923	0,12639678	-3,3377901	0,00084448	0,00464172	
ENSGALG00000015692	1953,56891	-0,422243248	0,13365312	-3,1592472	0,00158177	0,00777748	POLN
ENSGALG00000013607	7250,98124	-0,422561681	0,11201001	-3,7725349	0,0001616	0,0011382	
ENSGALG00000006002	2601,77756	-0,422978689	0,1302324	-3,247876	0,0011627	0,00605359	WHAMM
ENSGALG00000002763	8455,88563	-0,423193935	0,11582995	-3,6535794	0,00025861	0,00170672	FRZB
ENSGALG00000000962	2645,42242	-0,423212428	0,12644055	-3,3471258	0,00081654	0,00451002	PUS3
ENSGALG00000008708	9031,55021	-0,423296325	0,10630235	-3,9820033	6,83E-05	0,00053843	
ENSGALG00000014568	6675,25211	-0,423460012	0,11373368	-3,7232595	0,00019667	0,00134486	
ENSGALG00000001850	22801,4935	-0,423798382	0,1070003	-3,9607213	7,47E-05	0,00058168	RARS
ENSGALG00000016701	2038,86851	-0,423807866	0,1319527	-3,2118167	0,00131899	0,00671829	
ENSGALG00000003288	22428,6978	-0,423843105	0,12398262	-3,4185687	0,00062951	0,00363171	TARS
ENSGALG00000009177	7539,35878	-0,423896895	0,11006703	-3,8512612	0,00011751	0,00085924	FMR1
ENSGALG00000012852	33011,4945	-0,42431457	0,11899849	-3,5657139	0,00036287	0,00228618	MKRN1
ENSGALG00000002288	13379,2923	-0,424413539	0,10575851	-4,0130439	5,99E-05	0,0004809	LIG3
ENSGALG00000011177	6032,23061	-0,424449663	0,11472261	-3,699791	0,00021578	0,00146014	CEP290
ENSGALG00000005945	41976,0293	-0,425016747	0,10489477	-4,0518393	5,08E-05	0,00041729	
ENSGALG00000002119	6930,58517	-0,425364527	0,1209038	-3,5182064	0,00043447	0,00266338	C16orf62
ENSGALG00000000033	3918,69493	-0,425866952	0,12660413	-3,3637683	0,00076886	0,00430606	

ENSGALG00000004181	1761,48747	-0,425938272	0,13751656	-3,0973599	0,00195253	0,00925292	
ENSGALG00000016911	5511,46419	-0,426013919	0,11550644	-3,6882267	0,00022582	0,00151831	RNF219
ENSGALG00000005205	5076,63735	-0,426364463	0,13358149	-3,191793	0,00141393	0,00710879	RNFT1
ENSGALG00000016955	4576,70807	-0,426960619	0,13063542	-3,2683373	0,00108181	0,00570666	VWA8
ENSGALG00000008616	22133,5216	-0,427012968	0,11420797	-3,738907	0,00018482	0,00127734	
ENSGALG00000004104	16560,1522	-0,427862285	0,12883118	-3,3211081	0,00089661	0,00488488	INTS1
ENSGALG00000002262	28425,4355	-0,428265663	0,10466808	-4,0916548	4,28E-05	0,00035977	ETF1
ENSGALG00000004776	7278,29032	-0,428490556	0,1147583	-3,7338524	0,00018857	0,00129865	
ENSGALG00000012227	10485,4738	-0,428492425	0,10916783	-3,9250796	8,67E-05	0,00066503	TSPAN5
ENSGALG00000017111	14958,6545	-0,428517877	0,1112294	-3,8525595	0,00011689	0,00085547	NUP58
ENSGALG00000008844	4547,72028	-0,428880605	0,13511839	-3,1741098	0,00150297	0,00744376	SLC31A1
ENSGALG00000023571	6639,21372	-0,428973189	0,12324229	-3,4807305	0,00050005	0,00300183	TRIM59
ENSGALG00000014953	10323,6261	-0,429472364	0,13438161	-3,1959161	0,00139388	0,00702134	RWDD1
ENSGALG00000016454	3850,19078	-0,429504414	0,12203619	-3,5194838	0,00043239	0,00265657	E2F6
ENSGALG00000000346	4943,52317	-0,429865287	0,11696755	-3,6750815	0,00023777	0,00158535	
ENSGALG00000004133	28687,5841	-0,430003398	0,1149324	-3,7413591	0,00018303	0,00126815	SRSF10
ENSGALG00000006730	17262,8448	-0,430049755	0,10295568	-4,1770378	2,95E-05	0,00026144	
ENSGALG00000027835	5051,56121	-0,430117051	0,11513346	-3,7358127	0,00018711	0,00129184	PCBD1
ENSGALG00000001501	5273,66434	-0,430190725	0,11395127	-3,7752164	0,00015987	0,0011272	MAPK1
ENSGALG00000006500	13286,4752	-0,430344251	0,11868069	-3,626068	0,00028777	0,00187553	
ENSGALG00000004986	14224,0481	-0,430376609	0,11894558	-3,6182647	0,00029658	0,00192378	CERCAM
ENSGALG00000004756	2781,91816	-0,43117271	0,12469583	-3,4577958	0,00054461	0,0032218	CEP89
ENSGALG00000015184	7792,59624	-0,431383947	0,12288745	-3,5103988	0,00044744	0,00273115	
ENSGALG00000007705	251805,658	-0,431935045	0,09889184	-4,3677523	1,26E-05	0,00012567	
ENSGALG00000011555	11516,3317	-0,431962973	0,11422487	-3,7816893	0,00015577	0,0011017	QKI
ENSGALG00000014222	26624,5153	-0,431998929	0,09984019	-4,3269043	1,51E-05	0,00014748	
ENSGALG00000011013	3992,5007	-0,43257266	0,11842255	-3,6527896	0,00025941	0,00171115	
ENSGALG00000008413	12232,7391	-0,432788919	0,11510536	-3,7599371	0,00016996	0,00119276	
ENSGALG00000007481	6576,27254	-0,43296248	0,10915554	-3,966473	7,29E-05	0,00057077	HACD3
ENSGALG00000006378	6236,19115	-0,43312035	0,13117722	-3,3017955	0,00096068	0,00517187	
ENSGALG00000013920	7232,43287	-0,433479079	0,1210408	-3,5812642	0,00034194	0,00217443	SUV39H2
ENSGALG00000002877	3840,76126	-0,433897954	0,13612607	-3,1874713	0,00143523	0,00719463	LRRC45
ENSGALG00000011548	3329,63059	-0,434202614	0,12811304	-3,3892149	0,00070093	0,00398296	PPP1R13B
ENSGALG00000010976	5423,33644	-0,434636867	0,12461543	-3,4878255	0,00048697	0,00293365	CCDC126
ENSGALG00000009930	17651,9175	-0,434701609	0,10108014	-4,300564	1,70E-05	0,000163	
ENSGALG00000006221	21172,6562	-0,434757063	0,10200869	-4,2619612	2,03E-05	0,00018949	KIF1A
ENSGALG00000028462	7479,58699	-0,434862527	0,12695407	-3,4253531	0,000614	0,00356791	
ENSGALG00000008355	3052,82011	-0,434887931	0,13359721	-3,2552171	0,00113306	0,0059333	
ENSGALG00000011543	9465,14039	-0,435034048	0,12352777	-3,521751	0,00042871	0,00263991	SDAD1
ENSGALG00000009519	11233,6738	-0,43521067	0,10594254	-4,1079878	3,99E-05	0,00034005	ARG2
ENSGALG00000009598	50606,5602	-0,435652779	0,12077169	-3,6072425	0,00030947	0,00199538	EPRS
ENSGALG00000006409	11343,9424	-0,436770571	0,12587936	-3,4697554	0,00052093	0,00310258	
ENSGALG00000001717	40955,9308	-0,437024576	0,1153453	-3,7888373	0,00015135	0,00107551	SLC16A1
ENSGALG00000012680	2735,45258	-0,437301833	0,12310563	-3,5522488	0,00038195	0,00238436	E2F3
ENSGALG00000011475	4517,36266	-0,437819648	0,12412869	-3,5271432	0,00042007	0,00259376	
ENSGALG00000014375	10299,4805	-0,437825336	0,10798758	-4,0544047	5,03E-05	0,00041349	
ENSGALG00000017038	6592,44922	-0,437833021	0,13661218	-3,204934	0,00135093	0,00685027	PROSER1
ENSGALG00000012642	14397,9617	-0,437930988	0,11769371	-3,720938	0,00019848	0,00135573	
ENSGALG00000008796	7379,05533	-0,438293488	0,11574409	-3,7867461	0,00015263	0,00108291	
ENSGALG00000007999	6686,69473	-0,438325223	0,11125069	-3,9399777	8,15E-05	0,00062871	MRPS7
ENSGALG00000009448	19270,6318	-0,43880669	0,10703455	-4,0996732	4,14E-05	0,00035053	IFRD1
ENSGALG00000008163	17633,4055	-0,439350797	0,12629148	-3,4788633	0,00050355	0,00301485	PSME4
ENSGALG00000004522	17438,7774	-0,439397212	0,11296651	-3,8896238	0,0001004	0,00075272	
ENSGALG00000005854	8969,91159	-0,439557852	0,11347852	-3,8734895	0,00010729	0,00079659	CLUH
ENSGALG00000014870	12682,5875	-0,439767118	0,11699794	-3,7587596	0,00017076	0,00119653	
ENSGALG00000009731	11305,3134	-0,439893222	0,10611405	-4,1454759	3,39E-05	0,00029501	
ENSGALG00000006427	15012,1637	-0,439960428	0,10348	-4,251647	2,12E-05	0,00019748	CARS
ENSGALG00000012497	3402,65629	-0,440007974	0,12129309	-3,6276427	0,00028602	0,00186503	
ENSGALG00000022802	18215,8301	-0,44018485	0,113024	-3,8946139	9,84E-05	0,00073869	
ENSGALG00000000430	10996,8493	-0,440297246	0,11045127	-3,9863485	6,71E-05	0,00052989	SPPL2B
ENSGALG00000007145	45254,0897	-0,440432711	0,0991199	-4,4434336	8,85E-06	9,27E-05	ITGB1
ENSGALG00000003071	6465,89293	-0,441229907	0,11836541	-3,7276929	0,00019324	0,00132563	

ENSGALG00000015049	15802,9479	-0,441268065	0,11099473	-3,9755768	7,02E-05	0,00055286	
ENSGALG00000008988	11231,5561	-0,442984401	0,11261589	-3,933587	8,37E-05	0,00064373	ZZZ3
ENSGALG00000006403	20700,7028	-0,442987653	0,10779744	-4,1094449	3,97E-05	0,00033876	MESDC2
ENSGALG00000010957	23588,3054	-0,443064247	0,10206261	-4,3411023	1,42E-05	0,00014017	USP1
ENSGALG00000020879	1971,7439	-0,443502872	0,13705801	-3,2358771	0,0012127	0,00627312	SLC30A7
ENSGALG00000012696	9654,978	-0,443665071	0,1168806	-3,7958828	0,00014712	0,00104761	
ENSGALG00000012000	7157,91614	-0,444031788	0,10961123	-4,0509697	5,10E-05	0,00041817	METTL14
ENSGALG00000005206	7037,81003	-0,444288079	0,11301214	-3,9313307	8,45E-05	0,0006489	ACSL3
ENSGALG00000003861	8032,14284	-0,444434747	0,10819238	-4,1078193	3,99E-05	0,00034009	HERC4
ENSGALG00000005241	3696,95899	-0,444802963	0,12608477	-3,5278089	0,00041901	0,00258842	
ENSGALG00000029011	22651,8846	-0,444941401	0,10030346	-4,4359527	9,17E-06	9,53E-05	SLC35F1
ENSGALG00000026971	4341,37635	-0,445130127	0,11930931	-3,7308917	0,0001908	0,00131268	RRAGD
ENSGALG00000007704	19378,5947	-0,445645488	0,1426064	-3,1250034	0,00177803	0,00854496	
ENSGALG00000003400	9822,18573	-0,446090625	0,10733726	-4,155972	3,24E-05	0,0002836	AIMP2
ENSGALG00000009020	9304,45489	-0,446477137	0,11401531	-3,9159402	9,01E-05	0,0006861	JAG1
ENSGALG00000013131	1864,58441	-0,447051346	0,13334241	-3,3526569	0,0008004	0,00444793	C18orf21
ENSGALG00000002536	10005,1999	-0,447408092	0,11487944	-3,894588	9,84E-05	0,00073869	STX2
ENSGALG00000002863	21477,0924	-0,447764784	0,12646631	-3,5405856	0,00039924	0,00248203	ERGIC1
ENSGALG00000007437	4980,32711	-0,447866955	0,11297477	-3,9643095	7,36E-05	0,00057531	DPP8
ENSGALG00000010612	2716,32593	-0,44797699	0,14444805	-3,1013018	0,00192672	0,00914762	PRKD3
ENSGALG00000016385	1906,4631	-0,448510168	0,13792552	-3,2518287	0,00114665	0,00599848	TSSC1
ENSGALG00000011642	18424,3446	-0,448893503	0,1458916	-3,0768974	0,00209167	0,00980319	ANKRD17
ENSGALG00000001845	10040,8917	-0,449444654	0,10707839	-4,1973421	2,70E-05	0,00024254	PHTF1
ENSGALG00000012568	2033,21553	-0,449796718	0,13848137	-3,2480667	0,00116192	0,00605185	
ENSGALG00000006324	5540,59909	-0,450353768	0,14477251	-3,1107684	0,00186601	0,00888313	
ENSGALG00000012598	6595,51322	-0,450640697	0,12253012	-3,6777953	0,00023526	0,00157089	NAA35
ENSGALG00000002519	3426,12944	-0,450983038	0,12415844	-3,6323189	0,00028089	0,00183506	SLC25A33
ENSGALG00000007652	11015,3194	-0,45121126	0,11033188	-4,0895819	4,32E-05	0,00036185	SUCLG2
ENSGALG00000005959	3317,97301	-0,451515508	0,12097245	-3,732383	0,00018968	0,00130559	GLMN
ENSGALG00000016803	4775,60706	-0,451555414	0,13299179	-3,395363	0,00068538	0,0039109	ST6GAL2
ENSGALG00000002663	8380,31752	-0,451591009	0,11688065	-3,8636934	0,00011169	0,00082355	ATR
ENSGALG00000007075	11514,1233	-0,451627608	0,12690022	-3,5589189	0,00037238	0,00233857	ATP13A3
ENSGALG00000004049	11640,7727	-0,45169714	0,10780601	-4,1899069	2,79E-05	0,00024948	ITFG1
ENSGALG00000013233	9834,12864	-0,4517759	0,11414632	-3,9578665	7,56E-05	0,00058733	
ENSGALG00000006336	7051,08462	-0,452052929	0,12470158	-3,6250777	0,00028887	0,00188183	
ENSGALG00000007659	35189,3993	-0,45246564	0,10270533	-4,4054738	1,06E-05	0,00010813	HYOU1
ENSGALG00000008088	3813,14193	-0,452924609	0,11705672	-3,8692748	0,00010916	0,00080882	ACSL4
ENSGALG00000008848	8858,46011	-0,453382128	0,10839768	-4,1825813	2,88E-05	0,00025631	
ENSGALG00000009835	14808,4285	-0,453500739	0,11172389	-4,0591207	4,93E-05	0,00040645	XPOT
ENSGALG00000015458	9174,0129	-0,453606012	0,13072342	-3,4699674	0,00052052	0,00310149	ILDR2
ENSGALG00000005840	6869,83939	-0,454270613	0,11585326	-3,9210861	8,82E-05	0,00067425	DENND5A
ENSGALG00000006094	10694,3183	-0,454736184	0,1116856	-4,0715741	4,67E-05	0,00038696	PHF6
ENSGALG00000004713	12581,373	-0,454819325	0,1044249	-4,355468	1,33E-05	0,00013206	
ENSGALG00000005160	9338,8368	-0,454934871	0,12551054	-3,6246746	0,00028933	0,00188297	VMP1
ENSGALG00000021883	1337,96899	-0,455471836	0,14673539	-3,1040354	0,001909	0,00907827	
ENSGALG00000005489	5406,3342	-0,455551792	0,12948295	-3,5182378	0,00043442	0,00266338	
ENSGALG00000016196	5946,1092	-0,455745009	0,13219264	-3,4475824	0,00056563	0,00332514	
ENSGALG00000011191	11563,8832	-0,455971006	0,10723132	-4,2522187	2,12E-05	0,00019721	
ENSGALG00000008839	9872,74847	-0,456849267	0,12866693	-3,5506348	0,0003843	0,00239683	
ENSGALG00000008415	6540,80438	-0,456904989	0,11868052	-3,8498736	0,00011818	0,00086366	ALS2
ENSGALG00000000884	7194,47798	-0,456928071	0,11254093	-4,0601057	4,91E-05	0,00040499	CXXC5
ENSGALG00000002338	29369,7857	-0,457290151	0,14291025	-3,1998415	0,00137503	0,00695175	
ENSGALG00000001179	4301,53132	-0,45735712	0,11540169	-3,9631753	7,40E-05	0,00057772	
ENSGALG00000002954	6734,12402	-0,457794297	0,12058072	-3,7965796	0,00014671	0,00104576	MBTD1
ENSGALG00000008497	15581,4745	-0,457962816	0,11566741	-3,9593072	7,52E-05	0,00058414	SLC38A2
ENSGALG00000007777	3900,17866	-0,45814234	0,12874776	-3,5584491	0,00037305	0,0023406	SLC39A10
ENSGALG00000026322	5686,93416	-0,458344915	0,11820551	-3,8775258	0,00010552	0,00078767	
ENSGALG00000013647	1849,99539	-0,458404091	0,13582178	-3,3750411	0,00073805	0,00415399	TFB1M
ENSGALG00000026639	1529,36894	-0,458562471	0,13972096	-3,2819876	0,00103078	0,00547351	SMCO4
ENSGALG00000004531	7125,02349	-0,458962037	0,11174171	-4,1073476	4,00E-05	0,00034057	PLOD1
ENSGALG00000007842	5019,26998	-0,459124954	0,11745791	-3,9088467	9,27E-05	0,0007038	
ENSGALG00000016930	13372,7628	-0,459461549	0,10645494	-4,3160189	1,59E-05	0,0001534	DIS3

ENSGALG00000008705	14944,0116	-0,4597037	0,10963126	-4,1931808	2,75E-05	0,00024622	
ENSGALG00000015773	9016,27354	-0,459782065	0,10646265	-4,318717	1,57E-05	0,00015208	
ENSGALG00000008482	22772,2201	-0,459855147	0,11052584	-4,1606122	3,17E-05	0,00027898	STAG2
ENSGALG00000010411	14781,2197	-0,460452211	0,1181671	-3,8966194	9,75E-05	0,00073496	RNF13
ENSGALG00000002501	3377,42917	-0,46100497	0,12275816	-3,7553917	0,00017307	0,00121087	
ENSGALG00000006281	5170,2004	-0,461106682	0,11489632	-4,0132414	5,99E-05	0,00048078	
ENSGALG00000006078	2849,4853	-0,461126659	0,1296477	-3,5567671	0,00037545	0,00235237	PALB2
ENSGALG00000013893	1508,86891	-0,461829974	0,1439203	-3,2089287	0,00133231	0,00677346	MNS1
ENSGALG00000015633	9547,40157	-0,462141421	0,10577029	-4,3692935	1,25E-05	0,00012525	
ENSGALG00000016980	3122,81674	-0,462357479	0,12582273	-3,6746738	0,00023815	0,00158711	COG3
ENSGALG00000013136	2283,14809	-0,462408191	0,13185399	-3,5069714	0,00045324	0,00275976	
ENSGALG00000000687	3686,83705	-0,462411333	0,12645225	-3,6568058	0,00025538	0,00168785	
ENSGALG00000016445	7095,59053	-0,463408352	0,11403536	-4,0637251	4,83E-05	0,000399	NOL10
ENSGALG00000009899	35232,7937	-0,46345514	0,10855368	-4,2693637	1,96E-05	0,00018445	
ENSGALG00000011794	10368,9097	-0,463824908	0,11474911	-4,0420785	5,30E-05	0,00043291	DDX47
ENSGALG00000007057	3861,26178	-0,46473909	0,12088959	-3,8443267	0,00012088	0,0008806	LRRC28
ENSGALG00000012655	11727,4649	-0,4651119	0,10973943	-4,2383297	2,25E-05	0,00020772	POLR3B
ENSGALG00000015138	14137,444	-0,465157078	0,12418565	-3,7456587	0,00017992	0,00125045	
ENSGALG00000002942	14672,6332	-0,465828236	0,13435921	-3,4670361	0,00052623	0,0031314	JMJD1C
ENSGALG00000014217	2291,12491	-0,46602463	0,13899423	-3,3528343	0,00079989	0,0044469	
ENSGALG00000010054	3266,33312	-0,466429533	0,12708508	-3,6702148	0,00024235	0,00161268	
ENSGALG00000004158	2874,23978	-0,467330867	0,12292142	-3,8018669	0,00014361	0,00102584	
ENSGALG00000028849	26828,63	-0,467375912	0,11688987	-3,9984296	6,38E-05	0,0005068	SCAF11
ENSGALG00000011309	5857,33759	-0,467663025	0,12262631	-3,8137249	0,00013689	0,00098656	PLXNC1
ENSGALG00000013728	12921,1551	-0,467853342	0,10806143	-4,3295129	1,49E-05	0,00014648	PPAT
ENSGALG00000013765	8392,68097	-0,468026469	0,11553655	-4,0508954	5,10E-05	0,00041817	
ENSGALG00000002282	2493,12249	-0,468163907	0,13046007	-3,5885608	0,00033251	0,0021254	TMEM200A
ENSGALG00000000895	27062,2913	-0,468166268	0,11013528	-4,2508292	2,13E-05	0,00019793	NUDC
ENSGALG00000002655	6693,97945	-0,468798244	0,13255859	-3,5365361	0,00040541	0,00251466	ITGAV
ENSGALG00000006121	11011,1981	-0,468808823	0,11657303	-4,0215891	5,78E-05	0,00046653	PIK3C2A
ENSGALG00000011232	2855,76217	-0,469477808	0,12392263	-3,7884753	0,00015157	0,00107652	
ENSGALG00000006638	2375,89839	-0,469621749	0,13927345	-3,3719401	0,00074641	0,00419411	GRIK4
ENSGALG00000009954	1375,37198	-0,469791805	0,14501118	-3,2396936	0,00119658	0,00620391	DYNC2LI1
ENSGALG00000007058	9479,72971	-0,469857249	0,10779741	-4,3587065	1,31E-05	0,00013031	LSG1
ENSGALG00000011101	5741,10347	-0,47005983	0,1114966	-4,2159117	2,49E-05	0,00022671	URB2
ENSGALG00000000843	7211,68859	-0,471124949	0,14308635	-3,2925919	0,00099268	0,00530016	DOT1L
ENSGALG00000011246	5026,82702	-0,471179653	0,11657189	-4,0419664	5,30E-05	0,00043291	
ENSGALG00000007564	1255,86877	-0,471313446	0,14906226	-3,1618562	0,00156767	0,00773047	
ENSGALG00000016836	6379,66167	-0,471449962	0,11875041	-3,9700911	7,18E-05	0,00056355	TUBGCP3
ENSGALG00000002439	1784,31098	-0,471657703	0,15336793	-3,0753347	0,00210266	0,00985127	
ENSGALG00000000721	4145,80621	-0,47173717	0,14279654	-3,3035616	0,00095465	0,00514755	SLC41A1
ENSGALG00000008837	13730,8475	-0,471745405	0,10804461	-4,3662094	1,26E-05	0,00012637	PELI1
ENSGALG00000026412	3599,37504	-0,471881516	0,13799409	-3,4195778	0,00062718	0,00362134	
ENSGALG00000016195	2664,10393	-0,472549861	0,14052256	-3,3628042	0,00077155	0,00431935	
ENSGALG00000004228	2475,78192	-0,472593441	0,12611515	-3,747317	0,00017874	0,00124475	USP40
ENSGALG00000012647	9993,64528	-0,472701385	0,12593301	-3,7535939	0,00017432	0,00121772	RFX4
ENSGALG00000001627	4772,90031	-0,472962819	0,1154681	-4,0960473	4,20E-05	0,00035452	
ENSGALG00000026192	2515,18021	-0,473341595	0,1470736	-3,2183994	0,00128908	0,00660055	ST8SIA4
ENSGALG00000028267	15487,1518	-0,473386218	0,11459396	-4,1309877	3,61E-05	0,00031165	
ENSGALG00000006296	11149,2649	-0,47366212	0,10934983	-4,3316217	1,48E-05	0,0001453	
ENSGALG00000004055	3994,96157	-0,474043343	0,11949327	-3,9671132	7,27E-05	0,00056956	C7orf50
ENSGALG00000027440	2934,02695	-0,474361212	0,12732099	-3,7257111	0,00019477	0,0013332	
ENSGALG00000004889	8112,95247	-0,474641509	0,1093142	-4,3419931	1,41E-05	0,00013971	SLC2A1
ENSGALG00000005211	6813,79977	-0,474686708	0,11553568	-4,1085724	3,98E-05	0,00033962	
ENSGALG00000010182	7219,05659	-0,474781283	0,11239386	-4,2242636	2,40E-05	0,00021949	SLC29A1
ENSGALG00000015925	3585,11623	-0,475263897	0,12827839	-3,7049413	0,00021144	0,00143436	
ENSGALG00000026989	16347,2783	-0,47543634	0,12391447	-3,8368104	0,00012464	0,00090555	HOXA7
ENSGALG00000002783	1895,83684	-0,475653855	0,15057219	-3,1589755	0,00158325	0,00778022	
ENSGALG00000016409	21993,092	-0,475709899	0,1119047	-4,2510269	2,13E-05	0,00019789	KIDINS220
ENSGALG00000008686	2315,39071	-0,476496754	0,12925913	-3,6863683	0,00022748	0,00152868	TRDMT1
ENSGALG00000006906	7509,2168	-0,477103418	0,10793556	-4,4202615	9,86E-06	0,0001016	CHM
ENSGALG00000009841	1397,50609	-0,477667905	0,15523553	-3,0770527	0,00209058	0,00980319	FASTKD1

ENSGALG00000011342	3208,67383	-0,478233256	0,14250555	-3,3558921	0,00079109	0,00441063	FGD6
ENSGALG00000002045	30343,4023	-0,478318973	0,09981877	-4,7918739	1,65E-06	2,05E-05	
ENSGALG00000002460	17009,9997	-0,480154381	0,10514203	-4,5667216	4,95E-06	5,50E-05	
ENSGALG00000002862	5434,26011	-0,480739106	0,11106412	-4,3284827	1,50E-05	0,00014706	
ENSGALG00000012484	32855,9599	-0,4809575	0,12116285	-3,969513	7,20E-05	0,00056451	RIF1
ENSGALG00000010884	11252,8159	-0,481115067	0,11488944	-4,1876351	2,82E-05	0,00025182	DDX24
ENSGALG00000015901	15729,8858	-0,481643321	0,10542744	-4,5684816	4,91E-06	5,46E-05	SENP6
ENSGALG00000023516	18924,6811	-0,48179177	0,1052391	-4,578068	4,69E-06	5,25E-05	
ENSGALG00000002294	3404,11815	-0,482168758	0,12824623	-3,7597109	0,00017011	0,00119322	SEMA6A
ENSGALG00000003078	20488,0185	-0,483195985	0,10630098	-4,5455457	5,48E-06	5,97E-05	MMD
ENSGALG00000008765	5344,75739	-0,483352119	0,12788071	-3,779711	0,00015701	0,00110992	C1D
ENSGALG00000005134	3683,92085	-0,483437706	0,1457126	-3,3177481	0,00090746	0,00493612	ZSWIM8
ENSGALG00000009967	51604,1361	-0,483675694	0,10307537	-4,6924466	2,70E-06	3,17E-05	LRPPRC
ENSGALG00000012702	17854,6411	-0,483830731	0,11942579	-4,0513085	5,09E-05	0,00041799	JARID2
ENSGALG00000015830	24016,1217	-0,484314141	0,10300764	-4,7017304	2,58E-06	3,04E-05	SYNCRIP
ENSGALG00000004887	17300,5109	-0,484420703	0,10687429	-4,5326215	5,83E-06	6,30E-05	
ENSGALG00000002781	17608,1718	-0,484707648	0,12726008	-3,8087958	0,00013965	0,00100278	
ENSGALG00000001950	14346,8445	-0,484721287	0,11288002	-4,2941281	1,75E-05	0,0001671	PMPCA
ENSGALG00000001777	2307,31017	-0,484938734	0,14695286	-3,2999612	0,00096698	0,00519347	MFSD11
ENSGALG00000002198	1337,36066	-0,485078535	0,14899049	-3,2557685	0,00113086	0,00592407	FHOD1
ENSGALG00000004416	1683,22849	-0,485858255	0,14199597	-3,4216342	0,00062246	0,00359944	TTC19
ENSGALG00000012258	6299,78758	-0,485912726	0,11533315	-4,2131229	2,52E-05	0,00022876	
ENSGALG00000013745	3956,92601	-0,486562498	0,1295758	-3,7550415	0,00017331	0,00121132	RTTN
ENSGALG00000008862	12336,5298	-0,486712741	0,10633346	-4,5772303	4,71E-06	5,26E-05	DNAJC10
ENSGALG00000008670	2394,2538	-0,486758564	0,13455087	-3,6176545	0,00029728	0,00192648	
ENSGALG00000009157	10495,9323	-0,48696748	0,11167801	-4,36046	1,30E-05	0,00012946	
ENSGALG00000002906	23481,0393	-0,487686151	0,1087191	-4,4857451	7,27E-06	7,71E-05	
ENSGALG00000005198	7882,46709	-0,488262054	0,11634671	-4,1966123	2,71E-05	0,00024316	RPS6KB1
ENSGALG00000004750	7577,76657	-0,488295007	0,10928295	-4,4681721	7,89E-06	8,32E-05	NOL8
ENSGALG00000010570	52092,719	-0,488515596	0,10051531	-4,8601111	1,17E-06	1,51E-05	BTF3L4
ENSGALG00000016909	4324,69907	-0,488526858	0,11625721	-4,2021211	2,64E-05	0,00023858	NDFIP2
ENSGALG00000004638	3370,76137	-0,48870476	0,14148157	-3,4541939	0,00055194	0,00325875	
ENSGALG00000001036	9684,70052	-0,488799588	0,1105545	-4,4213448	9,81E-06	0,00010117	ELAC2
ENSGALG00000009981	1405,74497	-0,49003231	0,14652981	-3,3442499	0,00082505	0,00454598	PREPL
ENSGALG00000010638	13156,3134	-0,49033693	0,12087564	-4,0565407	4,98E-05	0,00041072	CASP3
ENSGALG00000006252	14647,7489	-0,49037706	0,13939868	-3,5178027	0,00043514	0,00266623	LMO4
ENSGALG00000000272	5267,8402	-0,490419343	0,13544359	-3,6208384	0,00029365	0,00190591	
ENSGALG00000008553	5356,62728	-0,490452503	0,13463719	-3,6427714	0,00026972	0,00176719	INO80
ENSGALG00000016404	5806,16288	-0,492286998	0,11929847	-4,1265157	3,68E-05	0,00031657	
ENSGALG00000010335	1726,88898	-0,492481433	0,14371477	-3,4267976	0,00061074	0,0035505	
ENSGALG00000002141	14007,0584	-0,492600599	0,10621413	-4,6378068	3,52E-06	4,03E-05	UGGT1
ENSGALG00000008871	16063,5897	-0,492812674	0,13495076	-3,6517962	0,00026041	0,00171613	
ENSGALG00000015059	7575,55005	-0,49315511	0,1131114	-4,3599064	1,30E-05	0,00012969	
ENSGALG00000005652	5954,57786	-0,493244478	0,11326504	-4,3547812	1,33E-05	0,0001323	
ENSGALG00000023576	3299,29916	-0,493564294	0,12078163	-4,0864186	4,38E-05	0,0003657	PIM3
ENSGALG00000008798	4574,76494	-0,493864419	0,11829778	-4,1747565	2,98E-05	0,0002639	ZMYND19
ENSGALG00000020742	13114,1447	-0,493871262	0,13939629	-3,5429299	0,00039571	0,0024612	PPRC1
ENSGALG00000011962	7036,62107	-0,494326456	0,13488463	-3,6648093	0,00024752	0,00164472	
ENSGALG00000015478	5554,56829	-0,494614804	0,13028443	-3,796423	0,0001468	0,00104588	
ENSGALG00000007326	7076,35907	-0,494698301	0,1094474	-4,5199637	6,19E-06	6,66E-05	PMM2
ENSGALG00000005683	5447,0767	-0,494902817	0,11790098	-4,1976141	2,70E-05	0,00024241	ARHGAP29
ENSGALG00000000795	1504,93647	-0,495778483	0,1425043	-3,4790423	0,00050321	0,00301416	MFSD12
ENSGALG00000004273	11441,3705	-0,495936282	0,1511882	-3,2802578	0,00103712	0,00550504	RALGPS2
ENSGALG00000014998	1987,96101	-0,495971981	0,15420457	-3,2163248	0,00129844	0,00664345	
ENSGALG00000015684	4408,68885	-0,496145153	0,11398344	-4,3527826	1,34E-05	0,00013339	
ENSGALG00000004570	5026,79729	-0,496270556	0,13511313	-3,6730002	0,00023972	0,00159598	NCOA3
ENSGALG00000013257	123007,221	-0,496798558	0,10262669	-4,8408318	1,29E-06	1,64E-05	
ENSGALG00000027635	9027,18129	-0,49702612	0,11896336	-4,1779765	2,94E-05	0,00026087	NME3
ENSGALG00000003596	25796,9116	-0,497856616	0,162082	-3,0716343	0,0021289	0,00994712	TRRAP
ENSGALG00000010624	9648,37586	-0,498764877	0,10861449	-4,5920656	4,39E-06	4,91E-05	MEMO1
ENSGALG00000026865	1245,99167	-0,49909443	0,16196027	-3,0815855	0,00205901	0,00967338	XRCC2
ENSGALG00000007691	3206,91346	-0,499115113	0,12945293	-3,8555722	0,00011546	0,00084651	ARMC9

ENSGALG00000007155	2000,50129	-0,499370494	0,14915389	-3,3480218	0,00081391	0,00449729	RMI2
ENSGALG00000003874	8943,2552	-0,499635075	0,10978836	-4,5508931	5,34E-06	5,87E-05	
ENSGALG00000005695	3266,30309	-0,500082186	0,13148632	-3,803302	0,00014278	0,00102045	CHEK2
ENSGALG00000025792	3188,10638	-0,500292681	0,12184936	-4,1058293	4,03E-05	0,0003426	FRRS1L
ENSGALG00000011264	1925,3077	-0,500518992	0,14795759	-3,3828544	0,00071737	0,00406279	
ENSGALG00000016758	1465,78964	-0,5005603	0,14656111	-3,4153691	0,00063696	0,00366533	LIPT1
ENSGALG00000010716	5501,72957	-0,50085931	0,11885573	-4,2140106	2,51E-05	0,00022817	AGA
ENSGALG00000009269	3597,54557	-0,501724842	0,13020726	-3,8532784	0,00011655	0,00085403	PTGR2
ENSGALG00000011857	24467,2306	-0,502240381	0,10517202	-4,775418	1,79E-06	2,19E-05	
ENSGALG00000003719	6864,04349	-0,502933526	0,135236	-3,7189322	0,00020007	0,00136332	
ENSGALG00000006524	12378,2851	-0,503792354	0,10770154	-4,677671	2,90E-06	3,38E-05	
ENSGALG00000016781	17546,5065	-0,504088725	0,12484349	-4,0377654	5,40E-05	0,00043994	MAP4K4
ENSGALG00000014922	15076,9971	-0,504425665	0,11868898	-4,2499788	2,14E-05	0,00019855	ROCK1
ENSGALG00000004690	52340,4796	-0,504461763	0,10076346	-5,006396	5,55E-07	7,63E-06	
ENSGALG00000002728	6868,77576	-0,504726866	0,12336417	-4,0913571	4,29E-05	0,00035977	SLC16A3
ENSGALG00000011877	4219,79352	-0,504776099	0,12198763	-4,1379284	3,50E-05	0,00030411	
ENSGALG00000005533	4875,77262	-0,504976403	0,12223566	-4,1311708	3,61E-05	0,0003116	ZMYM3
ENSGALG00000013209	5984,63909	-0,505418476	0,11156134	-4,5304087	5,89E-06	6,36E-05	
ENSGALG00000015252	4947,94416	-0,505578034	0,14235024	-3,5516487	0,00038283	0,00238871	ST3GAL6
ENSGALG00000007234	4564,3675	-0,505755971	0,11795303	-4,2877742	1,80E-05	0,00017099	CLCN5
ENSGALG00000010034	2578,09523	-0,50624459	0,13221838	-3,8288518	0,00012874	0,00093236	
ENSGALG00000003540	2816,51357	-0,506324474	0,14263664	-3,5497505	0,0003856	0,0024027	CEP95
ENSGALG00000005992	1391,45334	-0,506625581	0,15329789	-3,3048438	0,00095029	0,0051298	PDE8A
ENSGALG00000006935	12264,1833	-0,506724591	0,10942458	-4,6308116	3,64E-06	4,15E-05	
ENSGALG00000019867	6166,97553	-0,506841982	0,11192678	-4,5283354	5,95E-06	6,41E-05	WDCP
ENSGALG00000008420	3322,24803	-0,507225725	0,1221905	-4,1511061	3,31E-05	0,00028877	CAND2
ENSGALG00000009782	45976,8535	-0,507579083	0,10626981	-4,7763242	1,79E-06	2,18E-05	NAA15
ENSGALG00000002424	2020,65689	-0,508419582	0,16029303	-3,1718134	0,0015149	0,0074947	RECQL5
ENSGALG00000002652	8163,31793	-0,509052755	0,13652522	-3,7286352	0,00019252	0,00132315	
ENSGALG00000005509	6460,5427	-0,509428544	0,14277078	-3,5681568	0,0003595	0,00226707	DPYD
ENSGALG00000004990	1688,4348	-0,509590179	0,1471207	-3,4637558	0,00053269	0,00316154	
ENSGALG00000010735	16013,9368	-0,510060882	0,1046006	-4,8762709	1,08E-06	1,40E-05	
ENSGALG00000003628	14552,598	-0,510073233	0,14143166	-3,6064997	0,00031036	0,00199787	
ENSGALG00000015787	3935,81115	-0,510253054	0,14104041	-3,6177791	0,00029714	0,00192647	RNGTT
ENSGALG00000008778	6210,22008	-0,51039033	0,14919074	-3,4210591	0,00062378	0,00360473	SPRED2
ENSGALG00000012135	48183,5814	-0,511735314	0,10797482	-4,7393951	2,14E-06	2,56E-05	ATF4
ENSGALG00000017085	4116,94944	-0,511832688	0,12507401	-4,0922387	4,27E-05	0,00035928	SLC7A1
ENSGALG00000002709	2938,4248	-0,511853662	0,14024529	-3,6497031	0,00026254	0,0017285	MYO1C
ENSGALG00000004621	8629,88481	-0,512109985	0,12302965	-4,1624925	3,15E-05	0,00027687	PREX1
ENSGALG00000005278	9525,24365	-0,512823817	0,15834712	-3,2386054	0,00120116	0,00622289	
ENSGALG00000014791	22071,9343	-0,513191503	0,12375943	-4,1466861	3,37E-05	0,00029365	WDR3
ENSGALG00000009825	16041,5695	-0,51401966	0,11051134	-4,6512843	3,30E-06	3,80E-05	SRGAP1
ENSGALG00000001441	10679,0004	-0,514329303	0,10523513	-4,8874299	1,02E-06	1,33E-05	UBE2G1
ENSGALG00000026468	7333,85705	-0,514962132	0,10964748	-4,6965248	2,65E-06	3,11E-05	CHSY1
ENSGALG00000002646	11173,6378	-0,515104787	0,14774595	-3,4864225	0,00048953	0,00294777	FAM171B
ENSGALG00000005653	3018,77554	-0,515161656	0,12312595	-4,1840218	2,86E-05	0,00025519	NFKB2
ENSGALG00000011707	2975,95888	-0,516018775	0,13531464	-3,8134734	0,00013703	0,00098658	
ENSGALG00000003566	2456,14829	-0,516245691	0,13569542	-3,8044444	0,00014212	0,00101682	SMURF2
ENSGALG00000003047	12574,6527	-0,516252465	0,13206797	-3,9089908	9,27E-05	0,0007038	SUGP2
ENSGALG00000007527	4564,36351	-0,516973735	0,11834809	-4,3682476	1,25E-05	0,00012548	ADARB1
ENSGALG00000012550	2418,32562	-0,517745286	0,13904231	-3,7236527	0,00019636	0,00134344	HMOX1
ENSGALG00000009207	1636,75887	-0,518081559	0,15117707	-3,426985	0,00061032	0,00354957	TRIM2
ENSGALG00000012207	4193,90666	-0,518404651	0,11552573	-4,4873522	7,21E-06	7,66E-05	CCNT2
ENSGALG00000009451	10542,6813	-0,518444547	0,1054026	-4,9187072	8,71E-07	1,14E-05	PPID
ENSGALG00000010189	6061,82346	-0,518843565	0,11081881	-4,6819089	2,84E-06	3,32E-05	
ENSGALG00000011596	8210,15954	-0,519655791	0,1247256	-4,1663925	3,09E-05	0,00027306	
ENSGALG00000006622	2389,74327	-0,520113735	0,13317469	-3,9054999	9,40E-05	0,00071163	FRA10AC1
ENSGALG00000014784	4045,40608	-0,520975246	0,14051541	-3,7076021	0,00020923	0,00142008	MAST4
ENSGALG00000009992	11680,4728	-0,521017168	0,11356065	-4,588008	4,47E-06	5,01E-05	ARHGAP5
ENSGALG00000015889	13285,7099	-0,52134607	0,14846895	-3,5114822	0,00044562	0,00272187	PHIP
ENSGALG00000007752	6790,46143	-0,521363763	0,1083691	-4,8110001	1,50E-06	1,88E-05	COPS7B
ENSGALG00000005850	15672,1065	-0,521743975	0,10948222	-4,7655589	1,88E-06	2,29E-05	FNBP1L

ENSGALG00000012354	2395,65855	-0,521764337	0,15908224	-3,2798402	0,00103866	0,00551105	
ENSGALG00000015314	12934,4444	-0,521997148	0,10929519	-4,7760302	1,79E-06	2,19E-05	BEND3
ENSGALG00000004397	3452,74049	-0,523035736	0,1275508	-4,1006073	4,12E-05	0,00034977	MLXIP
ENSGALG00000012592	3142,27379	-0,523397807	0,14066624	-3,7208488	0,00019855	0,00135573	RMI1
ENSGALG00000002487	10163,3626	-0,523537895	0,1313011	-3,9873079	6,68E-05	0,00052806	SFSWAP
ENSGALG00000003260	9438,41253	-0,523953501	0,11054819	-4,7395938	2,14E-06	2,56E-05	UTP6
ENSGALG00000012720	24474,404	-0,524679114	0,11154136	-4,7038973	2,55E-06	3,01E-05	NUP153
ENSGALG00000005425	38874,994	-0,524696803	0,11835069	-4,4334072	9,28E-06	9,63E-05	OGT
ENSGALG00000010861	2392,2986	-0,524736464	0,13282156	-3,9506874	7,79E-05	0,00060351	OMA1
ENSGALG00000004489	1483,19946	-0,524803254	0,14557684	-3,6049914	0,00031216	0,00200761	
ENSGALG00000011770	14710,9194	-0,525890593	0,11884576	-4,4249841	9,64E-06	9,98E-05	UMPS
ENSGALG00000002850	6570,40738	-0,526275555	0,13474436	-3,9057334	9,39E-05	0,00071134	RASA2
ENSGALG00000023562	1492,39213	-0,526424205	0,1608921	-3,2719084	0,00106824	0,00564929	
ENSGALG00000015836	3297,85416	-0,526441345	0,13246596	-3,9741633	7,06E-05	0,00055575	CEP162
ENSGALG00000014426	14003,0497	-0,526522361	0,12072956	-4,361172	1,29E-05	0,00012913	KPNA1
ENSGALG00000011982	5512,8717	-0,527666168	0,14035943	-3,7593924	0,00017033	0,00119413	PDE5
ENSGALG00000011149	879,074202	-0,527681437	0,16261977	-3,2448788	0,00117501	0,00610367	PLA2R1
ENSGALG00000008096	6070,98948	-0,528190931	0,1247684	-4,2333711	2,30E-05	0,00021135	SUFU
ENSGALG00000008847	3493,18836	-0,528756478	0,12631689	-4,1859522	2,84E-05	0,00025336	VPS54
ENSGALG00000013177	2545,01825	-0,528877548	0,14123176	-3,7447493	0,00018057	0,0012537	BCAT1
ENSGALG00000017392	1036,56961	-0,528892969	0,15858794	-3,3350138	0,00085295	0,00468075	SPDYA
ENSGALG00000012979	10526,2614	-0,528975634	0,14809894	-3,5717718	0,00035457	0,00224223	
ENSGALG00000000208	3421,17661	-0,529383362	0,13520345	-3,9154575	9,02E-05	0,00068709	MCC
ENSGALG00000004474	1133,2959	-0,529777367	0,15342984	-3,4528967	0,0005546	0,0032709	PLEKHF1
ENSGALG00000013203	7478,18622	-0,52983334	0,11632258	-4,5548622	5,24E-06	5,78E-05	
ENSGALG00000011419	3053,01947	-0,529859697	0,12366202	-4,2847409	1,83E-05	0,0001731	DUSP4
ENSGALG00000010159	4422,59589	-0,529906457	0,12230696	-4,3325945	1,47E-05	0,00014486	
ENSGALG00000002974	1015,77625	-0,529999669	0,16832703	-3,1486308	0,00164037	0,0080164	SPHKAP
ENSGALG00000002624	31172,8318	-0,530323004	0,11239841	-4,7182429	2,38E-06	2,82E-05	BMS1
ENSGALG00000006826	4092,14666	-0,530661584	0,1454636	-3,6480713	0,00026422	0,00173783	KLHL25
ENSGALG00000002253	1425,48713	-0,530784268	0,14560305	-3,6454199	0,00026696	0,00175077	HEMK1
ENSGALG00000011569	5815,29092	-0,530885029	0,12042999	-4,408246	1,04E-05	0,000107	SCYL2
ENSGALG00000003944	1246,48403	-0,531232705	0,1600895	-3,3183482	0,00090552	0,00492749	FAM189A1
ENSGALG00000004347	6495,16049	-0,533321296	0,11036285	-4,832435	1,35E-06	1,70E-05	NEDD4
ENSGALG00000000891	3716,06871	-0,533422492	0,12306892	-4,3343397	1,46E-05	0,00014396	NSUN5
ENSGALG00000015818	5198,53941	-0,53353462	0,12660774	-4,2140758	2,51E-05	0,00022817	
ENSGALG00000015448	2486,28528	-0,533672096	0,16395778	-3,2549362	0,00113418	0,00593689	
ENSGALG00000008312	1844,09907	-0,534378374	0,13847665	-3,8589783	0,00011386	0,00083706	GSAP
ENSGALG00000004506	11897,6421	-0,534405871	0,11012391	-4,8527686	1,22E-06	1,55E-05	URI1
ENSGALG00000013085	2436,82685	-0,535478159	0,13668214	-3,9176895	8,94E-05	0,00068237	PTPRO
ENSGALG00000008098	5171,85115	-0,536763264	0,11526046	-4,6569592	3,21E-06	3,70E-05	NAMPT
ENSGALG00000006856	8733,4208	-0,537151634	0,11626332	-4,6201299	3,83E-06	4,35E-05	CD151
ENSGALG00000011024	13428,0492	-0,537183599	0,10486491	-5,122625	3,01E-07	4,37E-06	CACHD1
ENSGALG00000010564	3014,7883	-0,537373895	0,12339974	-4,354741	1,33E-05	0,0001323	TBCK
ENSGALG00000015360	9022,70642	-0,537653684	0,10556349	-5,0931783	3,52E-07	5,05E-06	KIAA1524
ENSGALG00000026253	1652,9426	-0,5379573	0,15060806	-3,5719024	0,0003544	0,00224216	TMEM144
ENSGALG00000003705	3639,3367	-0,538250704	0,12318514	-4,3694452	1,25E-05	0,00012525	VPS13C
ENSGALG00000012468	13779,9504	-0,538390713	0,13666178	-3,9395852	8,16E-05	0,00062927	
ENSGALG00000016440	2000,28995	-0,538612864	0,16061769	-3,3533845	0,0007983	0,00444169	KLF11
ENSGALG00000016835	6008,91026	-0,539714594	0,14683337	-3,6756945	0,0002372	0,00158232	
ENSGALG00000002873	15190,7659	-0,539979366	0,10838996	-4,9818208	6,30E-07	8,52E-06	
ENSGALG00000015701	9337,57881	-0,540112853	0,12997092	-4,1556437	3,24E-05	0,00028383	
ENSGALG00000015180	15097,1011	-0,540189844	0,11292112	-4,7837805	1,72E-06	2,11E-05	PSAT1
ENSGALG00000001389	3052,28108	-0,540945617	0,12602912	-4,2922273	1,77E-05	0,00016818	TTLL11
ENSGALG00000015491	4074,13394	-0,541085652	0,12196746	-4,4363116	9,15E-06	9,53E-05	
ENSGALG00000012121	12053,9223	-0,541445831	0,10454307	-5,179165	2,23E-07	3,34E-06	RCN1
ENSGALG00000013438	21891,8795	-0,541667727	0,11204746	-4,8342706	1,34E-06	1,69E-05	TEX10
ENSGALG00000001957	8870,63197	-0,541703645	0,13410352	-4,0394438	5,36E-05	0,00043733	
ENSGALG00000016460	6190,66257	-0,541890883	0,14841996	-3,6510648	0,00026116	0,00172019	
ENSGALG00000014573	17671,9053	-0,541989088	0,11034447	-4,9117921	9,02E-07	1,18E-05	NAT10
ENSGALG00000025884	6669,8373	-0,543082298	0,11342421	-4,7880633	1,68E-06	2,07E-05	NABP1
ENSGALG00000007835	1738,05901	-0,543517994	0,14833732	-3,6640679	0,00024824	0,00164868	PUS10

ENSGALG00000006895	13372,5266	-0,543915173	0,10588883	-5,1366627	2,80E-07	4,09E-06	CEP131
ENSGALG00000004364	7914,97638	-0,544307466	0,11563233	-4,7072254	2,51E-06	2,97E-05	ABR
ENSGALG00000026452	7763,72006	-0,545277299	0,13192983	-4,1330858	3,58E-05	0,0003098	MURC
ENSGALG00000012223	1554,61989	-0,545359079	0,13992319	-3,8975604	9,72E-05	0,00073252	ZRANB3
ENSGALG00000003440	1101,0238	-0,546469024	0,16030168	-3,4090037	0,00065201	0,00373771	
ENSGALG00000011351	157820,465	-0,547374715	0,15800979	-3,4641822	0,00053185	0,00315791	
ENSGALG00000016768	7527,22871	-0,54750013	0,11986999	-4,5674497	4,94E-06	5,48E-05	LONRF2
ENSGALG00000009503	5606,80387	-0,547842813	0,11865192	-4,6172267	3,89E-06	4,41E-05	RAB3GAP2
ENSGALG00000015746	3303,51188	-0,547863301	0,14036111	-3,9032414	9,49E-05	0,00071632	JAM2
ENSGALG00000003937	13547,5021	-0,548529686	0,13001502	-4,2189717	2,45E-05	0,0002241	GEMIN5
ENSGALG00000005727	12274,5514	-0,548590838	0,11540717	-4,7535249	2,00E-06	2,41E-05	
ENSGALG00000017083	1252,48764	-0,54907192	0,15967948	-3,4385878	0,00058476	0,00341401	
ENSGALG00000006971	10409,6787	-0,549657911	0,11864179	-4,6329201	3,61E-06	4,12E-05	
ENSGALG00000003026	1987,5595	-0,550107166	0,16872812	-3,260317	0,00111288	0,00583885	MKL2
ENSGALG00000011400	12123,5934	-0,550294288	0,10312199	-5,3363428	9,48E-08	1,53E-06	USP44
ENSGALG00000028501	7019,46403	-0,550742687	0,11797631	-4,6682481	3,04E-06	3,52E-05	DCK
ENSGALG00000007097	2035,76357	-0,551520351	0,16963125	-3,2512898	0,00114883	0,00599972	RPS6KA6
ENSGALG00000017006	4626,73679	-0,551986159	0,11796255	-4,6793338	2,88E-06	3,35E-05	SETDB2
ENSGALG00000004071	3249,44294	-0,552437495	0,12317196	-4,4850915	7,29E-06	7,73E-05	NETO2
ENSGALG00000000684	3181,04311	-0,552570682	0,14354544	-3,849448	0,00011838	0,00086424	KLHDC8A
ENSGALG00000003835	27823,2079	-0,552761335	0,10615776	-5,20698	1,92E-07	2,92E-06	
ENSGALG00000016883	3231,09542	-0,555002491	0,12790983	-4,3390135	1,43E-05	0,00014131	DOCK9
ENSGALG00000004813	26912,1889	-0,555221474	0,11731903	-4,732578	2,22E-06	2,65E-05	IARS
ENSGALG00000005774	2418,90539	-0,555555373	0,13336098	-4,1658014	3,10E-05	0,00027359	
ENSGALG00000013635	10619,1615	-0,555662229	0,12215986	-4,5486483	5,40E-06	5,91E-05	SCAF8
ENSGALG00000016468	6793,09113	-0,555855719	0,10894695	-5,1020767	3,36E-07	4,84E-06	
ENSGALG00000013430	2775,68087	-0,557212283	0,1237629	-4,5022564	6,72E-06	7,16E-05	
ENSGALG00000021487	6790,2413	-0,55890103	0,12790718	-4,3695829	1,24E-05	0,00012525	
ENSGALG00000016231	123947,503	-0,55907395	0,10983383	-5,0901798	3,58E-07	5,11E-06	
ENSGALG00000019178	3990,13341	-0,559313492	0,12498595	-4,475011	7,64E-06	8,08E-05	PRDM15
ENSGALG00000010022	5530,32804	-0,559381952	0,11898015	-4,7014729	2,58E-06	3,04E-05	PRMT9
ENSGALG00000011692	9047,47741	-0,559417827	0,11608282	-4,8191269	1,44E-06	1,81E-05	CDCA4
ENSGALG00000000447	760,920402	-0,559669779	0,17125604	-3,2680295	0,00108299	0,00571066	
ENSGALG00000011541	6975,19884	-0,559795067	0,14049051	-3,9845755	6,76E-05	0,00053309	BIN1
ENSGALG00000003510	5135,23749	-0,562160497	0,117815	-4,771553	1,83E-06	2,23E-05	UCK2
ENSGALG00000012508	4886,27256	-0,562203872	0,11484296	-4,8954143	9,81E-07	1,28E-05	
ENSGALG00000027231	9354,14873	-0,562988244	0,15371607	-3,6625204	0,00024975	0,00165578	NRARP
ENSGALG00000016897	2920,76606	-0,563110321	0,12347462	-4,560535	5,10E-06	5,64E-05	GPR180
ENSGALG00000010081	2535,91839	-0,563235953	0,1656002	-3,4011791	0,00067096	0,00383667	SH3D19
ENSGALG00000003074	3831,59863	-0,563796907	0,1286586	-4,382116	1,18E-05	0,0001188	HOMER3
ENSGALG00000012256	15406,5047	-0,563809375	0,16328803	-3,4528519	0,00055469	0,0032709	UTRN
ENSGALG00000026438	2075,6069	-0,563826313	0,13056342	-4,3184093	1,57E-05	0,00015218	
ENSGALG00000008132	7657,49338	-0,564518166	0,11630408	-4,8538123	1,21E-06	1,55E-05	
ENSGALG00000022808	24156,7928	-0,565497585	0,10524059	-5,3733791	7,73E-08	1,27E-06	
ENSGALG00000011228	7018,88755	-0,565699287	0,12484784	-4,5311101	5,87E-06	6,35E-05	WARS
ENSGALG00000014940	2607,30528	-0,566659105	0,15847617	-3,575674	0,00034933	0,00221571	ZUFSP
ENSGALG00000016917	1018,50229	-0,567495374	0,16489149	-3,4416292	0,00057822	0,00338311	
ENSGALG00000015431	2610,45813	-0,567537408	0,12799629	-4,4340144	9,25E-06	9,61E-05	
ENSGALG00000009855	7273,65346	-0,567662024	0,12230573	-4,6413362	3,46E-06	3,97E-05	TBC1D9
ENSGALG00000017079	12990,6555	-0,56791639	0,12482195	-4,5498117	5,37E-06	5,89E-05	USPL1
ENSGALG00000000195	1824,65996	-0,568283448	0,16007893	-3,5500202	0,0003852	0,00240134	SVCT2
ENSGALG00000003906	12475,0381	-0,568387401	0,10947502	-5,1919368	2,08E-07	3,14E-06	MPHOSPH10
ENSGALG00000016281	11421,2288	-0,569360911	0,1081146	-5,2662723	1,39E-07	2,17E-06	DMD
ENSGALG00000016391	2197,60679	-0,569812042	0,1354173	-4,2078232	2,58E-05	0,00023357	CNKSR2
ENSGALG00000015142	3202,10584	-0,570285644	0,13342262	-4,2742801	1,92E-05	0,00018068	DSG2
ENSGALG00000009714	1150,55938	-0,570305895	0,15558254	-3,6656163	0,00024674	0,00164114	CBR4
ENSGALG00000014783	10636,0999	-0,572122303	0,12610507	-4,5368701	5,71E-06	6,20E-05	
ENSGALG00000016910	23108,1995	-0,572230903	0,11163907	-5,1257228	2,96E-07	4,30E-06	RBM26
ENSGALG00000012849	5113,46952	-0,57279907	0,14885384	-3,8480636	0,00011906	0,00086774	SLC37A3
ENSGALG00000001363	21664,3518	-0,573517858	0,10988778	-5,2191233	1,80E-07	2,75E-06	MYBBP1A
ENSGALG00000003180	15412,6584	-0,573999724	0,10818558	-5,3056952	1,12E-07	1,78E-06	
ENSGALG00000013031	1094,94036	-0,574014118	0,17360499	-3,306438	0,0009449	0,0051051	CECR1

ENSGALG00000016807	10081,6726	-0,574724846	0,11386342	-5,0474932	4,48E-07	6,28E-06	
ENSGALG00000016841	10674,7371	-0,576008454	0,1276781	-4,5114115	6,44E-06	6,90E-05	
ENSGALG00000020057	1737,42699	-0,576621955	0,13651224	-4,2239578	2,40E-05	0,00021964	PIGF
ENSGALG00000007992	6680,6643	-0,5769061	0,10933512	-5,2764942	1,32E-07	2,06E-06	GGA3
ENSGALG00000003591	1041,85294	-0,577610116	0,17930224	-3,2214327	0,00127551	0,00653599	
ENSGALG00000014939	2493,31868	-0,579384653	0,17435114	-3,3230906	0,00089026	0,00486197	GFM2
ENSGALG00000004612	6633,12124	-0,579433401	0,10947713	-5,2927345	1,21E-07	1,90E-06	MTHFR
ENSGALG00000007008	8124,26296	-0,579649516	0,11753702	-4,9316337	8,15E-07	1,08E-05	POLR3E
ENSGALG00000004140	1503,56079	-0,579940212	0,16796039	-3,4528392	0,00055472	0,0032709	SH3BP4
ENSGALG00000015629	16787,8501	-0,581629702	0,10138489	-5,736848	9,65E-09	1,91E-07	
ENSGALG00000010202	1508,60268	-0,581722546	0,16884284	-3,4453492	0,00057032	0,00334551	ENTPD5
ENSGALG00000006713	2292,89242	-0,58181715	0,14500758	-4,0123223	6,01E-05	0,00048209	
ENSGALG00000011464	79055,7934	-0,582311331	0,10582388	-5,5026456	3,74E-08	6,56E-07	EIF5
ENSGALG00000017130	3473,80286	-0,582570563	0,12916331	-4,510341	6,47E-06	6,91E-05	
ENSGALG00000015000	15510,2528	-0,584898632	0,10441804	-5,6015092	2,12E-08	3,93E-07	LYAR
ENSGALG00000020899	644,115695	-0,585391733	0,1791404	-3,2677818	0,00108394	0,00571345	
ENSGALG00000027517	979,679294	-0,585667855	0,16395158	-3,5722001	0,00035399	0,00224065	gga-mir-3064
ENSGALG00000007677	1856,87137	-0,585818002	0,14520033	-4,03455	5,47E-05	0,0004451	THNSL1
ENSGALG00000003426	1636,16491	-0,587203746	0,15658355	-3,7500986	0,00017677	0,00123292	RHBDL3
ENSGALG00000015765	12998,2541	-0,587270267	0,1455632	-4,0344695	5,47E-05	0,0004451	
ENSGALG00000015310	4463,74355	-0,587354563	0,1636253	-3,5896318	0,00033115	0,00211768	
ENSGALG00000004144	27007,8593	-0,588131949	0,10975399	-5,3586383	8,39E-08	1,37E-06	
ENSGALG00000012946	11580,0518	-0,588849778	0,11469761	-5,1339326	2,84E-07	4,13E-06	FAM60A
ENSGALG00000016187	8513,83254	-0,588903063	0,11295401	-5,2136534	1,85E-07	2,82E-06	WDR4
ENSGALG00000000284	7651,65914	-0,589684267	0,12270008	-4,8058996	1,54E-06	1,92E-05	HOXB4
ENSGALG00000015556	4802,19905	-0,591646833	0,12793817	-4,6244747	3,76E-06	4,27E-05	AFAP1
ENSGALG00000012147	18627,0418	-0,591734529	0,11258805	-5,255749	1,47E-07	2,30E-06	DDX18
ENSGALG00000008376	8424,82548	-0,592197559	0,12144073	-4,8764326	1,08E-06	1,40E-05	EIF2B5
ENSGALG00000005803	3271,76516	-0,592808251	0,15820978	-3,746976	0,00017898	0,0012457	
ENSGALG00000002326	890,069728	-0,593338649	0,18376433	-3,2288021	0,0012431	0,00640113	
ENSGALG00000011449	1934,52119	-0,594721435	0,14281353	-4,1643215	3,12E-05	0,00027501	
ENSGALG00000007536	1357,15347	-0,59551277	0,15673099	-3,7995852	0,00014494	0,0010337	
ENSGALG00000014213	4954,50424	-0,596053951	0,12959904	-4,5992158	4,24E-06	4,76E-05	PRR5
ENSGALG00000009458	5058,56826	-0,596085417	0,1247109	-4,7797377	1,76E-06	2,15E-05	
ENSGALG00000005171	1357,22705	-0,597406416	0,14458	-4,1320128	3,60E-05	0,00031066	AP1S3
ENSGALG00000005243	13547,5044	-0,597738338	0,10284356	-5,8121126	6,17E-09	1,27E-07	PPP3CB
ENSGALG00000020438	1854,20177	-0,597817303	0,14024952	-4,2625265	2,02E-05	0,00018927	
ENSGALG00000027159	3433,75807	-0,598718251	0,16764923	-3,5712556	0,00035527	0,00224353	CNKSR3
ENSGALG00000014615	13865,3358	-0,598774766	0,14500125	-4,1294456	3,64E-05	0,00031335	LAMA1
ENSGALG00000011210	4763,25316	-0,598839459	0,11293547	-5,3024922	1,14E-07	1,81E-06	FAM175A
ENSGALG00000020003	15165,0611	-0,59891041	0,11112336	-5,3895993	7,06E-08	1,17E-06	MAP3K4
ENSGALG00000015409	17031,8673	-0,599227418	0,14738119	-4,0658336	4,79E-05	0,00039565	PROS1
ENSGALG00000011710	2961,63072	-0,599591029	0,18819478	-3,186013	0,00144248	0,00722302	
ENSGALG00000001003	4385,75735	-0,600081977	0,12148953	-4,9393718	7,84E-07	1,04E-05	SEPN1
ENSGALG00000006256	7733,33351	-0,600800071	0,11264625	-5,3335117	9,63E-08	1,55E-06	
ENSGALG00000011280	7822,14955	-0,603497491	0,12276846	-4,9157374	8,84E-07	1,16E-05	EEA1
ENSGALG00000010603	12309,7524	-0,604746743	0,10826314	-5,585897	2,32E-08	4,26E-07	
ENSGALG00000005807	96623,5673	-0,604834873	0,10467707	-5,7781028	7,55E-09	1,52E-07	IPO7
ENSGALG00000007030	1042,03468	-0,605544059	0,16028433	-3,7779368	0,00015813	0,00111611	
ENSGALG00000010623	6764,86118	-0,606701235	0,11188195	-5,4226907	5,87E-08	9,92E-07	ORC1
ENSGALG00000006147	7732,277	-0,607224855	0,1099367	-5,5234047	3,32E-08	5,88E-07	NUCB2
ENSGALG00000009348	14314,7884	-0,607303929	0,15062704	-4,0318387	5,53E-05	0,00044957	
ENSGALG00000011522	2524,77962	-0,607850232	0,13426355	-4,5272914	5,97E-06	6,44E-05	TRMT61A
ENSGALG00000010538	11960,1749	-0,60879058	0,11003246	-5,5328272	3,15E-08	5,61E-07	
ENSGALG00000011618	1230,20769	-0,609057453	0,18958218	-3,2126303	0,00131525	0,0067043	ADSSL1
ENSGALG00000015689	13523,3466	-0,609077567	0,14382998	-4,2347051	2,29E-05	0,00021053	KIAA0368
ENSGALG00000005492	6703,97246	-0,609713298	0,14208413	-4,2912131	1,78E-05	0,00016883	
ENSGALG00000009698	6354,41268	-0,610790424	0,15890149	-3,8438306	0,00012113	0,00088176	SLC38A1
ENSGALG00000016302	7538,43287	-0,61104581	0,14235289	-4,2924722	1,77E-05	0,00016811	
ENSGALG00000000438	3178,97206	-0,61235395	0,14457738	-4,2354755	2,28E-05	0,00020995	ERMP1
ENSGALG00000004169	13748,9297	-0,613558741	0,10706722	-5,7305937	1,00E-08	1,97E-07	
ENSGALG00000013760	3088,02148	-0,614432788	0,12107074	-5,0749899	3,88E-07	5,50E-06	

ENSGALG00000016574	1715,06369	-0,614768808	0,14203902	-4,3281683	1,50E-05	0,00014706	GEMIN8
ENSGALG00000007703	1248,99291	-0,614958252	0,15352984	-4,005464	6,19E-05	0,00049426	
ENSGALG00000013480	4896,2242	-0,615680556	0,12423491	-4,9557774	7,20E-07	9,61E-06	
ENSGALG00000016808	40422,889	-0,615970468	0,12307828	-5,0047047	5,59E-07	7,68E-06	RANBP2
ENSGALG00000006237	12767,8147	-0,616678741	0,12728487	-4,8448706	1,27E-06	1,61E-05	PKN2
ENSGALG00000016951	1808,55603	-0,617422601	0,13578215	-4,5471559	5,44E-06	5,94E-05	MTRF1
ENSGALG00000008265	23793,5701	-0,619236444	0,10979734	-5,639813	1,70E-08	3,22E-07	
ENSGALG00000017388	6991,3984	-0,619582917	0,11595165	-5,3434592	9,12E-08	1,48E-06	
ENSGALG00000005815	11029,7481	-0,619938317	0,10990838	-5,6405009	1,70E-08	3,22E-07	TMEM41B
ENSGALG00000012533	37098,6017	-0,619977689	0,14353496	-4,3193496	1,56E-05	0,00015175	
ENSGALG00000001192	22737,8249	-0,620466491	0,15464988	-4,0120722	6,02E-05	0,00048223	
ENSGALG00000016653	1523,27891	-0,621295808	0,14742536	-4,2143076	2,51E-05	0,00022817	
ENSGALG00000012447	5839,1863	-0,62156312	0,12782967	-4,8624324	1,16E-06	1,49E-05	
ENSGALG00000008539	7630,14576	-0,622137491	0,12115192	-5,1351847	2,82E-07	4,11E-06	ALG12
ENSGALG00000023419	9218,5287	-0,622730836	0,13105342	-4,7517328	2,02E-06	2,42E-05	HOXD4
ENSGALG00000000234	2924,36008	-0,623968223	0,14846856	-4,2026959	2,64E-05	0,00023813	EPB41L4A
ENSGALG00000005438	938,768709	-0,624083264	0,1616022	-3,8618489	0,00011253	0,00082862	PARVA
ENSGALG00000010975	6618,99977	-0,624280901	0,11571015	-5,3952131	6,84E-08	1,14E-06	TMTC2
ENSGALG00000005504	1081,65735	-0,624552568	0,17314094	-3,6071917	0,00030953	0,00199538	HNF1B
ENSGALG00000006609	9375,25541	-0,624834648	0,13736708	-4,5486491	5,40E-06	5,91E-05	EPHB1
ENSGALG00000014860	10686,5811	-0,625239897	0,10707403	-5,8393235	5,24E-09	1,09E-07	YES1
ENSGALG00000017159	5806,67135	-0,626252438	0,14290102	-4,3824212	1,17E-05	0,00011872	ATM
ENSGALG00000004046	1117,46957	-0,627070394	0,16993702	-3,6900164	0,00022424	0,00150916	LARP6
ENSGALG00000014855	4504,15014	-0,627700184	0,1611556	-3,8949947	9,82E-05	0,00073827	
ENSGALG00000010702	11951,0445	-0,628390454	0,10835766	-5,7992249	6,66E-09	1,36E-07	ADSS
ENSGALG00000015472	37144,6363	-0,629536006	0,14753725	-4,266963	1,98E-05	0,00018598	
ENSGALG00000012966	5358,75757	-0,62976328	0,1160975	-5,4244346	5,81E-08	9,83E-07	ZBTB2
ENSGALG00000005450	5500,73037	-0,632586778	0,12083814	-5,2349926	1,65E-07	2,54E-06	CCDC66
ENSGALG00000005256	11273,3374	-0,63393871	0,16414924	-3,8619656	0,00011248	0,00082862	EPHA4
ENSGALG00000014337	1369,70172	-0,634337238	0,14622858	-4,3379841	1,44E-05	0,00014177	NWD2
ENSGALG00000009850	11171,8574	-0,63459607	0,10804099	-5,8736605	4,26E-09	9,11E-08	NOVA1
ENSGALG00000004937	10776,4972	-0,634904509	0,10951564	-5,7973865	6,74E-09	1,37E-07	VGLL4
ENSGALG00000017368	7609,78676	-0,635358308	0,10950994	-5,8018322	6,56E-09	1,34E-07	TRNT1
ENSGALG00000007392	3781,92945	-0,635704603	0,14088967	-4,5120739	6,42E-06	6,88E-05	
ENSGALG00000003767	4407,98301	-0,636798593	0,11771515	-5,4096572	6,31E-08	1,06E-06	NKD1
ENSGALG00000007710	1737,58327	-0,637223247	0,15253964	-4,1774273	2,95E-05	0,00026123	
ENSGALG00000012813	15846,5259	-0,637913408	0,12201058	-5,228345	1,71E-07	2,63E-06	
ENSGALG00000005521	7067,27493	-0,637995859	0,13170481	-4,844135	1,27E-06	1,62E-05	PER2
ENSGALG00000022490	838,24845	-0,638026939	0,20482582	-3,1149732	0,00183962	0,00878204	
ENSGALG00000023451	10451,6138	-0,638494594	0,10528216	-6,0646041	1,32E-09	3,09E-08	TRPM7
ENSGALG00000007056	3476,50953	-0,638790551	0,18102964	-3,5286518	0,00041768	0,00258161	
ENSGALG00000003891	23052,983	-0,638963678	0,10013766	-6,3808527	1,76E-10	4,67E-09	ZNF423
ENSGALG00000012662	3593,37854	-0,639523486	0,12409213	-5,1536182	2,56E-07	3,78E-06	NUAK1
ENSGALG00000014138	4048,40305	-0,640267154	0,14067976	-4,5512386	5,33E-06	5,86E-05	NFXL1
ENSGALG00000011162	1836,73645	-0,640538648	0,15156233	-4,2262391	2,38E-05	0,00021772	CYP46A1
ENSGALG00000009240	3768,49084	-0,641416186	0,11670261	-5,4961599	3,88E-08	6,79E-07	NFE2L2
ENSGALG00000007352	21915,6386	-0,641667347	0,10923519	-5,8741815	4,25E-09	9,10E-08	
ENSGALG00000009445	3221,29491	-0,642383978	0,12265432	-5,2373531	1,63E-07	2,51E-06	INPP5F
ENSGALG00000010642	1388,2464	-0,643267076	0,1679801	-3,8294242	0,00012844	0,00093118	IRF2
ENSGALG00000005321	14326,6827	-0,645095033	0,14628713	-4,4097866	1,03E-05	0,0001064	
ENSGALG00000005024	4274,06304	-0,645541534	0,13739879	-4,6983057	2,62E-06	3,09E-05	EFCC1
ENSGALG00000026181	1587,87421	-0,645581251	0,15765694	-4,0948483	4,22E-05	0,00035587	
ENSGALG00000010536	11449,0338	-0,646034394	0,1106982	-5,8359971	5,35E-09	1,11E-07	FAF1
ENSGALG00000016480	10063,5827	-0,647091328	0,12228762	-5,2915523	1,21E-07	1,91E-06	SDC1
ENSGALG00000000258	23273,6972	-0,649481888	0,12291472	-5,2840043	1,26E-07	1,98E-06	
ENSGALG00000000761	2678,90106	-0,649687901	0,13200757	-4,9215959	8,58E-07	1,13E-05	TSKU
ENSGALG00000014902	5870,32391	-0,650325285	0,12934166	-5,0279647	4,96E-07	6,90E-06	
ENSGALG00000006864	655,136383	-0,651213169	0,19973556	-3,2603768	0,00111264	0,00583885	
ENSGALG00000014873	10860,5943	-0,6514166	0,10605415	-6,1423019	8,13E-10	1,95E-08	GJA1
ENSGALG00000012232	769,021092	-0,652989979	0,19777702	-3,3016474	0,00096119	0,00517256	DNAAF2
ENSGALG00000003154	3509,17671	-0,653496705	0,12218795	-5,3482908	8,88E-08	1,45E-06	TXNDC11
ENSGALG00000005845	8323,59423	-0,655210424	0,12474397	-5,2524419	1,50E-07	2,33E-06	SLC7A5

ENSGALG00000015241	1466,0595	-0,655397927	0,14176198	-4,6232279	3,78E-06	4,29E-05	
ENSGALG00000015653	733,019478	-0,655959247	0,20273574	-3,2355382	0,00121414	0,00627819	
ENSGALG00000016681	817,923627	-0,656368052	0,1696389	-3,8692071	0,00010919	0,00080882	DHRSX
ENSGALG00000000690	5466,00123	-0,657633862	0,14151675	-4,6470389	3,37E-06	3,87E-05	GDPD4
ENSGALG00000016817	6729,25411	-0,658902643	0,1108429	-5,944473	2,77E-09	6,16E-08	RASA3
ENSGALG00000006975	7158,07962	-0,659116429	0,117355	-5,6164324	1,95E-08	3,65E-07	
ENSGALG00000006840	2751,68484	-0,659559733	0,17118981	-3,8527979	0,00011678	0,00085525	
ENSGALG00000006681	619,278693	-0,660452265	0,21494413	-3,0726695	0,00212153	0,009926	BRSK2
ENSGALG00000010204	972,360103	-0,660708753	0,16806539	-3,9312602	8,45E-05	0,0006489	KCNC2
ENSGALG00000008551	7057,17591	-0,661341847	0,12495655	-5,2925743	1,21E-07	1,90E-06	CRELD2
ENSGALG00000007870	12818,5577	-0,66197661	0,11665024	-5,6748844	1,39E-08	2,68E-07	
ENSGALG00000005796	13227,6265	-0,664735985	0,10635028	-6,2504396	4,09E-10	1,01E-08	XBP1
ENSGALG00000008293	829,335943	-0,66560113	0,20054725	-3,3189242	0,00090365	0,00491931	SH3PXD2A
ENSGALG00000009915	13723,0991	-0,66741216	0,11515882	-5,7955799	6,81E-09	1,39E-07	EML4
ENSGALG00000005512	8645,01109	-0,667653797	0,11848963	-5,6347026	1,75E-08	3,31E-07	
ENSGALG00000008418	2809,25172	-0,668553187	0,12270622	-5,4484051	5,08E-08	8,65E-07	LARGE2
ENSGALG00000011139	4725,82862	-0,66929694	0,13400223	-4,9946703	5,89E-07	8,03E-06	
ENSGALG00000011686	12938,7332	-0,670528172	0,14703222	-4,5604165	5,11E-06	5,64E-05	SEMA5B
ENSGALG00000008240	2340,29769	-0,670953542	0,12956854	-5,1783675	2,24E-07	3,35E-06	CFLAR
ENSGALG00000006080	30589,3035	-0,672288685	0,10183892	-6,6014906	4,07E-11	1,17E-09	GPC4
ENSGALG00000028291	15611,0719	-0,672824764	0,13419575	-5,0137561	5,34E-07	7,39E-06	
ENSGALG00000004715	1867,94138	-0,672888292	0,15250355	-4,4122795	1,02E-05	0,00010534	
ENSGALG00000015912	3388,05891	-0,674238434	0,14603918	-4,6168326	3,90E-06	4,41E-05	
ENSGALG00000015708	22628,5619	-0,674375402	0,17848955	-3,7782347	0,00015794	0,00111536	FGFR3
ENSGALG00000008684	70609,009	-0,674536207	0,12880685	-5,236804	1,63E-07	2,52E-06	
ENSGALG00000010049	749,198004	-0,6748978	0,20825089	-3,2407919	0,00119198	0,00618241	PTPRB
ENSGALG00000003249	7468,82123	-0,675105103	0,11166729	-6,0456838	1,49E-09	3,45E-08	CWF19L1
ENSGALG00000006035	6729,77158	-0,676290578	0,10833566	-6,2425484	4,30E-10	1,06E-08	PDE3B
ENSGALG00000004494	4756,9197	-0,676838081	0,11957995	-5,66013	1,51E-08	2,89E-07	CCNE1
ENSGALG00000016451	7945,90073	-0,679900325	0,13943898	-4,8759701	1,08E-06	1,40E-05	ROCK2
ENSGALG00000006437	3771,99053	-0,682364759	0,12175344	-5,6044805	2,09E-08	3,87E-07	
ENSGALG00000009156	5048,98485	-0,682556058	0,16761459	-4,0721757	4,66E-05	0,0003862	CDAN1
ENSGALG00000014274	3304,6353	-0,683648491	0,13949356	-4,9009322	9,54E-07	1,25E-05	
ENSGALG00000012390	2074,46154	-0,684899295	0,14739748	-4,6466147	3,37E-06	3,87E-05	
ENSGALG00000009152	8451,92517	-0,685418448	0,13463812	-5,0908202	3,57E-07	5,10E-06	ATRNL1
ENSGALG00000004631	4926,28212	-0,686947368	0,19666311	-3,4930159	0,0004776	0,00288615	DRAXIN
ENSGALG00000010836	1260,23388	-0,688393097	0,16687599	-4,1251777	3,70E-05	0,00031781	
ENSGALG00000015879	11968,4164	-0,689623134	0,14800439	-4,6594775	3,17E-06	3,66E-05	HUNK
ENSGALG00000008234	5250,56204	-0,689849994	0,12730961	-5,4186797	6,00E-08	1,01E-06	ORC2
ENSGALG00000016308	5133,40137	-0,690314419	0,12814377	-5,3870307	7,16E-08	1,18E-06	MYC
ENSGALG00000014686	1912,60456	-0,691021625	0,17304639	-3,9932738	6,52E-05	0,00051675	FBN2
ENSGALG00000025627	543,306297	-0,691903941	0,1957541	-3,5345567	0,00040846	0,00253126	SNORD47
ENSGALG00000008292	9417,47651	-0,69263042	0,12226746	-5,6648794	1,47E-08	2,83E-07	
ENSGALG00000011679	1487,18993	-0,695574487	0,17407076	-3,9959294	6,44E-05	0,00051158	
ENSGALG00000007371	25835,4514	-0,695805113	0,11656083	-5,9694591	2,38E-09	5,36E-08	IGDCC3
ENSGALG00000017220	8610,42217	-0,695886193	0,12577786	-5,5326606	3,15E-08	5,61E-07	
ENSGALG00000008758	3528,49517	-0,696392948	0,11831828	-5,8857597	3,96E-09	8,56E-08	
ENSGALG00000028415	2970,11444	-0,698216592	0,20411152	-3,4207604	0,00062446	0,00360716	
ENSGALG00000016834	1336,22512	-0,698351437	0,16279355	-4,2897979	1,79E-05	0,00016974	MCF2L
ENSGALG00000002316	6016,72913	-0,700708149	0,10987572	-6,3772792	1,80E-10	4,77E-09	NUBP2
ENSGALG00000002689	3193,98974	-0,701352786	0,13477202	-5,2039941	1,95E-07	2,96E-06	
ENSGALG00000007501	495,825919	-0,702573548	0,22104241	-3,1784559	0,00148062	0,00735447	
ENSGALG00000006592	563,467935	-0,703616422	0,19089215	-3,6859369	0,00022786	0,00153051	
ENSGALG00000021316	17378,3782	-0,704586437	0,10231836	-6,8862169	5,73E-12	1,88E-10	
ENSGALG00000012153	842,909942	-0,706186244	0,17364706	-4,0667908	4,77E-05	0,00039426	METTL15
ENSGALG00000002762	6873,84376	-0,70662137	0,1320656	-5,3505333	8,77E-08	1,43E-06	UHRF1BP1
ENSGALG00000002726	8639,35675	-0,708755608	0,13953348	-5,0794664	3,78E-07	5,38E-06	KIF1B
ENSGALG00000007141	2245,3684	-0,70907976	0,1459375	-4,8587906	1,18E-06	1,52E-05	LRRK1
ENSGALG00000011680	3554,59737	-0,709602993	0,18365321	-3,8638203	0,00011163	0,00082355	EPHA5
ENSGALG00000012848	3745,19397	-0,711139877	0,17381561	-4,0913464	4,29E-05	0,00035977	
ENSGALG00000022857	997,971589	-0,711204976	0,16756929	-4,2442441	2,19E-05	0,000203	
ENSGALG00000015733	14565,4266	-0,711632203	0,17400694	-4,0896772	4,32E-05	0,00036185	

ENSGALG00000017365	2855,56643	-0,712049377	0,15174035	-4,6925512	2,70E-06	3,17E-05	
ENSGALG00000009562	5126,97314	-0,714412903	0,12876119	-5,548356	2,88E-08	5,20E-07	
ENSGALG00000003970	25720,8179	-0,714513793	0,12407312	-5,7588122	8,47E-09	1,70E-07	
ENSGALG00000003161	1569,92422	-0,715518291	0,16581373	-4,3151933	1,59E-05	0,00015387	
ENSGALG00000016927	1190,18904	-0,715628819	0,17643861	-4,0559649	4,99E-05	0,00041123	
ENSGALG00000013615	780,849369	-0,715808204	0,18333875	-3,9042931	9,45E-05	0,00071361	
ENSGALG00000010036	359,240321	-0,717787007	0,2219662	-3,2337671	0,00122169	0,00630861	FOSL2
ENSGALG00000015122	2528,57664	-0,719672556	0,13908654	-5,174279	2,29E-07	3,42E-06	TAF4B
ENSGALG00000020805	5789,80169	-0,719835862	0,11391762	-6,318916	2,63E-10	6,72E-09	LRRC8D
ENSGALG00000005430	950,090619	-0,72235333	0,20225367	-3,5715216	0,00035491	0,00224229	ALS2CL
ENSGALG00000002553	2782,54105	-0,723490416	0,16062171	-4,5043127	6,66E-06	7,10E-05	PCDH1
ENSGALG00000007242	1612,83074	-0,724284111	0,14362964	-5,0427204	4,59E-07	6,42E-06	SLITRK4
ENSGALG00000008078	7758,49805	-0,725207523	0,12521944	-5,7914932	6,98E-09	1,42E-07	
ENSGALG00000006978	1693,58452	-0,725851222	0,16965998	-4,2782701	1,88E-05	0,00017784	APOOL
ENSGALG00000004004	8836,32885	-0,725989536	0,12198937	-5,9512525	2,66E-09	5,95E-08	PHKB
ENSGALG00000005246	9658,47999	-0,7272373	0,12335529	-5,8954691	3,74E-09	8,11E-08	
ENSGALG00000028294	3609,41474	-0,727698928	0,13043446	-5,5790387	2,42E-08	4,40E-07	VWA5B2
ENSGALG00000006939	26463,2292	-0,728311968	0,1092521	-6,6663427	2,62E-11	7,76E-10	PFKP
ENSGALG00000006555	1381,69164	-0,729109536	0,15543069	-4,6908983	2,72E-06	3,19E-05	
ENSGALG00000025743	3141,82765	-0,729896626	0,1215881	-6,0030268	1,94E-09	4,42E-08	CDR2
ENSGALG00000016078	860,370977	-0,73004938	0,19386389	-3,7657832	0,00016603	0,00116639	
ENSGALG00000014419	9837,23866	-0,730417334	0,1131065	-6,4577842	1,06E-10	2,87E-09	
ENSGALG00000020479	3560,77871	-0,731996794	0,12990873	-5,6347005	1,75E-08	3,31E-07	
ENSGALG00000003378	7303,91386	-0,732735156	0,12423155	-5,8981405	3,68E-09	8,01E-08	USP42
ENSGALG00000015627	1040,22039	-0,73285779	0,21291454	-3,4420279	0,00057737	0,00337958	
ENSGALG00000026349	29196,624	-0,736634172	0,09993715	-7,3709745	1,69E-13	6,80E-12	
ENSGALG00000007069	592,208375	-0,738101541	0,23259838	-3,1732876	0,00150723	0,00745945	
ENSGALG00000016537	11629,8341	-0,739933749	0,1619397	-4,5691932	4,90E-06	5,45E-05	SCML2
ENSGALG00000010448	40598,9396	-0,742255902	0,1099244	-6,7524215	1,45E-11	4,48E-10	LTBP1
ENSGALG00000002679	1869,60302	-0,743662338	0,15155473	-4,9068896	9,25E-07	1,21E-05	GLT1D1
ENSGALG00000007331	18072,4344	-0,744880346	0,10843853	-6,8691486	6,46E-12	2,09E-10	SVIL
ENSGALG00000027639	27019,6011	-0,74689172	0,10811506	-6,9083041	4,90E-12	1,61E-10	
ENSGALG00000010522	11132,1004	-0,747786716	0,11992122	-6,2356496	4,50E-10	1,11E-08	
ENSGALG00000003975	3354,11348	-0,748226558	0,14508752	-5,1570704	2,51E-07	3,71E-06	
ENSGALG00000008910	360,623018	-0,749041615	0,22125446	-3,3854305	0,00071067	0,00402652	CERKL
ENSGALG00000003149	1878,4222	-0,749443102	0,15295343	-4,8998124	9,59E-07	1,25E-05	
ENSGALG00000007011	11491,2627	-0,750635431	0,11252384	-6,6709014	2,54E-11	7,56E-10	
ENSGALG00000023464	2176,79492	-0,751478603	0,16284203	-4,6147706	3,94E-06	4,45E-05	
ENSGALG00000019961	8882,89233	-0,7532835	0,12923015	-5,8290072	5,58E-09	1,16E-07	SLC18B1
ENSGALG00000003430	9094,42264	-0,754857044	0,10828451	-6,9710529	3,15E-12	1,08E-10	PMS2
ENSGALG00000015862	12207,7026	-0,756350316	0,14990125	-5,0456572	4,52E-07	6,34E-06	
ENSGALG00000008318	18620,5264	-0,756789619	0,11792666	-6,4174599	1,39E-10	3,71E-09	SLK
ENSGALG00000010412	643,758245	-0,756929566	0,17972006	-4,2117143	2,53E-05	0,00023004	WWTR1
ENSGALG00000004981	3521,90259	-0,757070103	0,13293065	-5,6952262	1,23E-08	2,40E-07	TSEN2
ENSGALG00000006981	1290,89613	-0,757579596	0,16920706	-4,477234	7,56E-06	8,01E-05	
ENSGALG00000007667	1024,27903	-0,757882718	0,16652285	-4,5512235	5,33E-06	5,86E-05	GPR158
ENSGALG00000000742	7852,97756	-0,757900463	0,13118075	-5,777528	7,58E-09	1,53E-07	
ENSGALG00000001419	2086,70105	-0,758492411	0,14074025	-5,389307	7,07E-08	1,17E-06	DAB2IP
ENSGALG00000016294	651,260147	-0,761040192	0,2034016	-3,7415644	0,00018288	0,00126776	HMGCLL1
ENSGALG00000009748	12417,6034	-0,762363604	0,12194724	-6,2515854	4,06E-10	1,01E-08	ASNS
ENSGALG00000006167	4665,12691	-0,763429455	0,16136845	-4,7309711	2,23E-06	2,66E-05	LRRC8B
ENSGALG00000017462	3834,70932	-0,763892042	0,11771244	-6,4894761	8,61E-11	2,38E-09	FNIP1
ENSGALG00000028254	428,450125	-0,764100303	0,21188759	-3,6061588	0,00031076	0,00199955	KIAA1033
ENSGALG00000007300	11025,5462	-0,764508198	0,12386685	-6,1720162	6,74E-10	1,63E-08	
ENSGALG00000026862	22574,7451	-0,764882325	0,16241479	-4,7094378	2,48E-06	2,94E-05	CLDN1
ENSGALG00000008621	9873,26199	-0,76612038	0,13164901	-5,8194163	5,91E-09	1,22E-07	NRP2
ENSGALG00000027389	3852,81541	-0,766788012	0,13085522	-5,8598199	4,63E-09	9,83E-08	RGS7BP
ENSGALG00000013782	3281,28093	-0,769533335	0,1760353	-4,3714718	1,23E-05	0,00012439	CDH7
ENSGALG00000026897	591,300069	-0,770711311	0,20311525	-3,7944532	0,00014797	0,00105311	
ENSGALG00000028737	5045,35121	-0,771568179	0,14540814	-5,3062243	1,12E-07	1,78E-06	
ENSGALG00000026518	766,815675	-0,773283921	0,17841103	-4,3342831	1,46E-05	0,00014396	RUNDC3A
ENSGALG00000005610	640,252491	-0,773621314	0,24264802	-3,1882449	0,00143139	0,00718021	SLC44A3

ENSGALG00000004594	1038,5627	-0,774792529	0,20891066	-3,7087267	0,0002083	0,00141449	CD74
ENSGALG00000013627	453,51839	-0,775913381	0,21080288	-3,6807532	0,00023255	0,00155583	SLC7A2
ENSGALG00000015887	12708,1034	-0,776552733	0,12924098	-6,0085643	1,87E-09	4,28E-08	URB1
ENSGALG00000015208	6752,93068	-0,778489641	0,1611505	-4,8308236	1,36E-06	1,72E-05	ROR2
ENSGALG00000006258	9759,66383	-0,778635815	0,12249805	-6,3563117	2,07E-10	5,40E-09	SPOCK1
ENSGALG00000009514	5928,6059	-0,779501664	0,11478642	-6,7908877	1,11E-11	3,50E-10	NRCAM
ENSGALG00000001020	7177,33389	-0,780112538	0,1084839	-7,1910444	6,43E-13	2,41E-11	
ENSGALG00000013683	18236,9052	-0,780228434	0,15315117	-5,0944986	3,50E-07	5,02E-06	ARID1B
ENSGALG00000008961	32098,1427	-0,780468343	0,16688611	-4,6766524	2,92E-06	3,39E-05	
ENSGALG00000012834	693,301303	-0,78090617	0,24310402	-3,2122306	0,00131709	0,00671113	AKR1D1
ENSGALG00000009393	806,584777	-0,782303357	0,19134913	-4,088356	4,34E-05	0,00036327	
ENSGALG00000012329	10571,2971	-0,782765701	0,1633862	-4,7908925	1,66E-06	2,05E-05	
ENSGALG00000026970	1108,65361	-0,785311616	0,15637208	-5,0220706	5,11E-07	7,09E-06	
ENSGALG00000009252	7419,33117	-0,788959505	0,12427971	-6,3482569	2,18E-10	5,65E-09	
ENSGALG00000012808	5957,05996	-0,789210521	0,13604537	-5,8010834	6,59E-09	1,35E-07	CDYL
ENSGALG00000006908	14243,3267	-0,789724754	0,14863119	-5,3133179	1,08E-07	1,72E-06	DLG1
ENSGALG00000000639	611,516533	-0,79001541	0,22610591	-3,4940061	0,00047583	0,00287674	LRRN2
ENSGALG00000016412	2161,43136	-0,791360693	0,13463882	-5,8776562	4,16E-09	8,94E-08	MBOAT2
ENSGALG00000005785	2650,42523	-0,791416097	0,18516638	-4,274081	1,92E-05	0,00018071	
ENSGALG00000009428	6528,61969	-0,792261942	0,14165832	-5,5927667	2,23E-08	4,11E-07	SUSD6
ENSGALG00000004662	3631,94871	-0,792400882	0,16649406	-4,7593343	1,94E-06	2,35E-05	DISP3
ENSGALG00000001995	9089,14914	-0,794307619	0,11001846	-7,2197668	5,21E-13	1,99E-11	LIMS2
ENSGALG00000012317	261,455312	-0,794799886	0,25524866	-3,1138259	0,00184678	0,008807	
ENSGALG00000025464	234,066267	-0,795229903	0,25312021	-3,1417085	0,00167965	0,00816148	SNORA68
ENSGALG00000004132	38489,9584	-0,797075856	0,11456459	-6,9574364	3,47E-12	1,17E-10	
ENSGALG00000026486	578,46167	-0,797913674	0,2123431	-3,7576624	0,00017151	0,00120056	
ENSGALG00000008378	25019,9708	-0,798096236	0,12248113	-6,5160751	7,22E-11	2,01E-09	SRGAP3
ENSGALG00000009932	6037,71039	-0,798314026	0,13917513	-5,7360394	9,69E-09	1,92E-07	THADA
ENSGALG00000011045	4900,98782	-0,799075807	0,1382254	-5,7809621	7,43E-09	1,50E-07	
ENSGALG00000014907	2917,0076	-0,80019622	0,15045066	-5,3186621	1,05E-07	1,68E-06	DCBLD1
ENSGALG00000010910	3629,98348	-0,801671392	0,12110467	-6,619657	3,60E-11	1,04E-09	
ENSGALG00000012576	1359,57449	-0,801916632	0,1553997	-5,1603485	2,46E-07	3,65E-06	RASEF
ENSGALG00000000583	9340,93564	-0,802342978	0,11958733	-6,7092639	1,96E-11	5,94E-10	SOX13
ENSGALG00000002900	2509,77542	-0,803716995	0,14588715	-5,5091691	3,61E-08	6,34E-07	NOTUM
ENSGALG00000027521	307,657411	-0,803725098	0,23936001	-3,3578085	0,00078563	0,00439095	
ENSGALG00000013212	21085,1244	-0,805085082	0,11564313	-6,9618063	3,36E-12	1,14E-10	
ENSGALG00000001883	2479,1556	-0,805297687	0,12694065	-6,3438913	2,24E-10	5,80E-09	ST6GALNAC2
ENSGALG00000003105	820,425544	-0,805784417	0,19045019	-4,2309456	2,33E-05	0,00021336	ANKFN1
ENSGALG00000002461	9290,02388	-0,808165073	0,1405519	-5,7499407	8,93E-09	1,77E-07	
ENSGALG00000002242	344,643988	-0,808545932	0,22942797	-3,5241821	0,00042479	0,00262173	
ENSGALG00000017103	2337,4348	-0,811501513	0,12799906	-6,3399023	2,30E-10	5,93E-09	WASF3
ENSGALG00000014957	24709,1325	-0,81285735	0,11248513	-7,226354	4,96E-13	1,90E-11	WDR1
ENSGALG00000003427	8550,66088	-0,813323099	0,12341029	-6,5903992	4,39E-11	1,25E-09	USP3
ENSGALG00000011089	415,157392	-0,814043658	0,22510112	-3,6163466	0,00029879	0,00193531	
ENSGALG00000011643	20679,5817	-0,816325927	0,14686948	-5,5581725	2,73E-08	4,94E-07	UTP20
ENSGALG00000010785	498,938482	-0,818155142	0,1993699	-4,1037045	4,07E-05	0,00034533	ACOT11
ENSGALG00000001220	3875,78907	-0,818155857	0,12990789	-6,2979691	3,02E-10	7,62E-09	HERPUD1
ENSGALG00000002598	7963,93283	-0,82047281	0,10859443	-7,5553857	4,18E-14	1,82E-12	TPST1
ENSGALG00000011511	101055,806	-0,820640197	0,12654102	-6,4851715	8,86E-11	2,44E-09	CKB
ENSGALG00000003160	1417,99184	-0,823423786	0,17588287	-4,68166	2,85E-06	3,32E-05	
ENSGALG00000006872	8998,32455	-0,823650254	0,11488863	-7,1691189	7,55E-13	2,79E-11	PISD
ENSGALG00000007039	11318,9545	-0,823801989	0,1338606	-6,1541783	7,55E-10	1,82E-08	
ENSGALG00000004569	2222,44652	-0,82424563	0,16546211	-4,9814767	6,31E-07	8,53E-06	UNC5B
ENSGALG00000010507	1443,23999	-0,824303692	0,1501991	-5,4880735	4,06E-08	7,08E-07	
ENSGALG00000003391	10590,4517	-0,827807721	0,10949157	-7,5604701	4,02E-14	1,76E-12	EIF2AK1
ENSGALG00000009546	1643,01363	-0,832251183	0,15248225	-5,4580202	4,81E-08	8,27E-07	
ENSGALG00000007717	242,860186	-0,834765713	0,25520347	-3,270981	0,00107175	0,00566236	GCNT7
ENSGALG00000014954	7661,41015	-0,835213371	0,11582931	-7,2107259	5,57E-13	2,10E-11	ZNF518B
ENSGALG00000008469	4008,42168	-0,83875822	0,14975785	-5,6007628	2,13E-08	3,94E-07	SPINT1
ENSGALG00000002543	5887,09033	-0,840502021	0,12400471	-6,7779847	1,22E-11	3,81E-10	CSGALNACT2
ENSGALG00000015980	410,785738	-0,841274637	0,25003277	-3,3646576	0,00076639	0,00429398	
ENSGALG00000011388	12926,4854	-0,842306194	0,12106876	-6,9572544	3,47E-12	1,17E-10	RABGGTB

ENSGALG00000015519	4471,93343	-0,844394446	0,12450731	-6,7818863	1,19E-11	3,71E-10	ROBO2
ENSGALG00000015656	12813,5339	-0,849003497	0,12458324	-6,814749	9,44E-12	3,00E-10	
ENSGALG00000012998	2617,34593	-0,849149352	0,17683084	-4,8020434	1,57E-06	1,96E-05	WNT5B
ENSGALG00000024984	247,007719	-0,849437052	0,24634113	-3,4482145	0,00056431	0,00332167	SNORD74
ENSGALG00000023609	5841,30372	-0,855145185	0,13510752	-6,3293679	2,46E-10	6,31E-09	
ENSGALG00000014178	3963,51548	-0,855544377	0,15025645	-5,6938946	1,24E-08	2,41E-07	SCUBE1
ENSGALG00000012394	13948,8666	-0,855781498	0,10361657	-8,259118	1,47E-16	8,54E-15	NID2
ENSGALG00000003668	1594,44053	-0,85921128	0,14225789	-6,0398144	1,54E-09	3,57E-08	
ENSGALG00000007846	1019,28883	-0,863598394	0,23089134	-3,7402805	0,00018381	0,00127223	
ENSGALG00000010311	430,972494	-0,865329105	0,20595707	-4,2015023	2,65E-05	0,00023907	NAV3
ENSGALG00000015681	7286,43618	-0,869123123	0,13859875	-6,270786	3,59E-10	9,02E-09	NRIP1
ENSGALG00000010010	1250,74925	-0,869227214	0,15347637	-5,6635898	1,48E-08	2,84E-07	RHOQ
ENSGALG00000008815	1450,48158	-0,86979829	0,16636094	-5,2283804	1,71E-07	2,63E-06	LRRN4
ENSGALG00000011376	402,726863	-0,870171475	0,21157345	-4,1128575	3,91E-05	0,00033421	ANKRD9
ENSGALG00000016896	2175,65472	-0,874558686	0,16022347	-5,4583681	4,81E-08	8,26E-07	ABCC4
ENSGALG00000023581	218,882989	-0,876769713	0,27869933	-3,145934	0,00165557	0,00806463	
ENSGALG00000009129	1209,82081	-0,878296092	0,17155705	-5,1195568	3,06E-07	4,43E-06	DLX5
ENSGALG00000004542	7447,83595	-0,879244031	0,1445917	-6,0808747	1,20E-09	2,81E-08	ZMYND8
ENSGALG00000016462	9552,69875	-0,880157248	0,13995983	-6,2886421	3,20E-10	8,07E-09	MYCN
ENSGALG00000028602	5443,19542	-0,881510014	0,15763284	-5,5921725	2,24E-08	4,12E-07	
ENSGALG00000011120	494,969816	-0,88227849	0,23574872	-3,7424529	0,00018223	0,00126458	
ENSGALG00000003657	764,546995	-0,88270712	0,19877995	-4,4406246	8,97E-06	9,38E-05	
ENSGALG00000028320	417,903769	-0,88451445	0,27202551	-3,2515863	0,00114763	0,00599848	
ENSGALG00000001749	4549,2907	-0,88875485	0,12757912	-6,9663033	3,25E-12	1,11E-10	ACSBG2
ENSGALG00000005723	2016,64963	-0,890647194	0,1398661	-6,3678562	1,92E-10	5,03E-09	
ENSGALG00000009360	602,385427	-0,890830846	0,19246128	-4,6286237	3,68E-06	4,19E-05	CAPN2
ENSGALG00000002555	1358,14983	-0,892902927	0,21167678	-4,2182374	2,46E-05	0,00022468	RET
ENSGALG00000000307	8913,08352	-0,894951246	0,11346269	-7,8876258	3,08E-15	1,50E-13	
ENSGALG00000012012	5383,1455	-0,895703636	0,17255692	-5,1907721	2,09E-07	3,15E-06	
ENSGALG00000017870	493,118342	-0,896459218	0,20520273	-4,3686516	1,25E-05	0,00012543	SNORD24
ENSGALG00000008547	11178,1998	-0,896850751	0,12624913	-7,1038172	1,21E-12	4,41E-11	
ENSGALG00000008509	336,231933	-0,897122538	0,26693729	-3,3607988	0,00077717	0,00434905	
ENSGALG00000012814	2520,13193	-0,898069575	0,12792713	-7,0201654	2,22E-12	7,76E-11	PXDC1
ENSGALG00000017838	224,751439	-0,898451215	0,26076736	-3,4454129	0,00057019	0,00334551	SNORA62
ENSGALG00000009687	2155,9656	-0,89897016	0,15726738	-5,7161896	1,09E-08	2,13E-07	KCNK2
ENSGALG00000014645	469,375441	-0,89901657	0,21753348	-4,1327733	3,58E-05	0,00030983	MEF2C
ENSGALG00000006498	20254,306	-0,899416586	0,16914983	-5,3172776	1,05E-07	1,69E-06	
ENSGALG00000026790	419,691292	-0,903869706	0,25606475	-3,5298482	0,0004158	0,0025729	DMRT2
ENSGALG00000002704	377,643334	-0,906514113	0,2414033	-3,7551852	0,00017321	0,00121125	
ENSGALG00000026258	289,161398	-0,909500707	0,24047052	-3,7821713	0,00015547	0,00110014	MAF
ENSGALG00000004231	640,948749	-0,911405982	0,18347202	-4,9675475	6,78E-07	9,10E-06	IFNLR1
ENSGALG00000013861	1063,53974	-0,913164571	0,17955855	-5,0856088	3,66E-07	5,22E-06	TNFAIP3
ENSGALG00000020723	828,748724	-0,913543779	0,17554467	-5,204053	1,95E-07	2,96E-06	
ENSGALG00000005994	969,598357	-0,91862167	0,17763077	-5,1715232	2,32E-07	3,46E-06	DERL3
ENSGALG00000010683	599,988497	-0,919156764	0,20193219	-4,551809	5,32E-06	5,85E-05	
ENSGALG00000001656	26148,0576	-0,922800056	0,11046642	-8,3536705	6,62E-17	4,00E-15	
ENSGALG00000009540	5865,4242	-0,9229711	0,11652612	-7,9207228	2,36E-15	1,17E-13	YAF2
ENSGALG00000025166	165,346098	-0,924288426	0,28745972	-3,2153668	0,00130278	0,00665566	SNORD75
ENSGALG00000001000	110996,795	-0,924720639	0,13746595	-6,726907	1,73E-11	5,28E-10	
ENSGALG00000008305	1229,58651	-0,925580955	0,18545643	-4,9908269	6,01E-07	8,18E-06	
ENSGALG00000016843	7409,4478	-0,926939677	0,12855412	-7,2105013	5,57E-13	2,10E-11	COL4A2
ENSGALG00000012911	418,92558	-0,929872331	0,26011606	-3,5748363	0,00035045	0,00222129	SYT10
ENSGALG00000008811	6177,80088	-0,931412058	0,14890526	-6,255065	3,97E-10	9,87E-09	SLC1A4
ENSGALG00000007952	17655,501	-0,932963194	0,1344416	-6,9395427	3,93E-12	1,32E-10	AMOT
ENSGALG00000023241	1184,67586	-0,938819916	0,18825868	-4,9868614	6,14E-07	8,32E-06	
ENSGALG00000028708	6182,01115	-0,938854547	0,12586313	-7,4593292	8,70E-14	3,59E-12	
ENSGALG00000009415	20765,0987	-0,939656238	0,14021273	-6,7016471	2,06E-11	6,21E-10	SMOC1
ENSGALG00000012726	95084,9683	-0,941672551	0,12611408	-7,4668311	8,21E-14	3,42E-12	
ENSGALG00000015622	657,059398	-0,944352456	0,21737746	-4,344298	1,40E-05	0,00013845	
ENSGALG00000015028	404,3337	-0,946739996	0,25007245	-3,7858628	0,00015318	0,00108585	
ENSGALG00000011258	44996,7983	-0,946927699	0,14493435	-6,5334939	6,43E-11	1,81E-09	
ENSGALG00000011068	1649,85658	-0,947751489	0,14451976	-6,5579373	5,46E-11	1,54E-09	COBLL1

ENSGALG00000005601	522,730238	-0,948098308	0,22540074	-4,2062786	2,60E-05	0,00023462	
ENSGALG00000006808	9236,5969	-0,948868926	0,10714219	-8,8561653	8,28E-19	6,00E-17	TSPAN6
ENSGALG00000013193	667,613336	-0,948945842	0,21648961	-4,3833321	1,17E-05	0,00011831	IRX2
ENSGALG00000023608	3676,98571	-0,950348621	0,13455102	-7,0631096	1,63E-12	5,82E-11	
ENSGALG00000005805	4566,75731	-0,954718819	0,14551669	-6,5608886	5,35E-11	1,52E-09	PLCD1
ENSGALG00000023463	1598,1146	-0,956997326	0,17129068	-5,5869784	2,31E-08	4,24E-07	
ENSGALG00000004393	1366,86861	-0,962441655	0,2126961	-4,5249615	6,04E-06	6,51E-05	
ENSGALG00000004284	19106,5125	-0,967926178	0,15442375	-6,2679877	3,66E-10	9,15E-09	
ENSGALG00000027342	11330,7864	-0,968330352	0,11546028	-8,3866967	5,00E-17	3,08E-15	
ENSGALG00000015908	1062,96226	-0,970529728	0,20345544	-4,7702325	1,84E-06	2,24E-05	COL12A1
ENSGALG00000011833	13327,6665	-0,971207285	0,11374653	-8,5383464	1,36E-17	8,83E-16	
ENSGALG00000009381	15338,368	-0,972226453	0,1330298	-7,3083359	2,70E-13	1,06E-11	
ENSGALG00000015940	2397,4582	-0,972506334	0,14243979	-6,8274905	8,64E-12	2,76E-10	
ENSGALG00000007367	230,098228	-0,973082329	0,26469761	-3,6762037	0,00023673	0,00157994	WFIKKN2
ENSGALG00000010597	2446,53537	-0,973101915	0,12722398	-7,6487303	2,03E-14	9,27E-13	
ENSGALG00000027497	292,355516	-0,973179721	0,23408863	-4,157313	3,22E-05	0,00028231	
ENSGALG00000019397	12676,4129	-0,975439675	0,11488543	-8,4905432	2,06E-17	1,31E-15	CERK
ENSGALG00000002103	289,282544	-0,979001464	0,28612607	-3,4215737	0,0006226	0,00359944	TPPP3
ENSGALG00000015624	34457,4291	-0,97910183	0,17589785	-5,5663091	2,60E-08	4,72E-07	VCAN
ENSGALG00000015164	3228,28619	-0,982829392	0,14802221	-6,6397428	3,14E-11	9,24E-10	
ENSGALG00000020788	1411,73564	-0,986044977	0,14888196	-6,6229983	3,52E-11	1,02E-09	
ENSGALG00000001371	2323,32831	-0,990350626	0,1647412	-6,0115541	1,84E-09	4,21E-08	
ENSGALG00000009758	793,28474	-0,991410492	0,19270405	-5,1447309	2,68E-07	3,94E-06	SLC7A11
ENSGALG00000015440	170,656307	-0,996123586	0,28603433	-3,4825316	0,0004967	0,00298434	
ENSGALG00000010692	6955,66893	-0,996294098	0,13891979	-7,1717217	7,41E-13	2,76E-11	LRP8
ENSGALG00000026582	1194,48697	-0,997100015	0,19077348	-5,2266176	1,73E-07	2,64E-06	
ENSGALG00000002532	116,305922	-0,997428466	0,31689231	-3,1475313	0,00164655	0,00803219	GJA4
ENSGALG00000010163	10928,5236	-0,998496131	0,12690773	-7,8678908	3,61E-15	1,74E-13	LGR5
ENSGALG00000007483	18280,1601	-0,999963194	0,14992811	-6,669618	2,56E-11	7,61E-10	LRIG1
ENSGALG00000005483	1190,77186	-1,005472997	0,15529553	-6,4745779	9,51E-11	2,59E-09	CDH13
ENSGALG00000011295	1389,14492	-1,007421144	0,15807983	-6,3728633	1,86E-10	4,90E-09	SOCS2
ENSGALG00000016627	15863,8272	-1,008110823	0,10822868	-9,3146368	1,22E-20	1,03E-18	
ENSGALG00000008281	355,49596	-1,009962725	0,2355243	-4,2881467	1,80E-05	0,00017082	NEURL1
ENSGALG00000015078	5440,75757	-1,010765666	0,18233191	-5,5435478	2,96E-08	5,32E-07	MLLT3
ENSGALG00000015112	8576,86268	-1,013051509	0,1332664	-7,6017026	2,92E-14	1,31E-12	
ENSGALG00000000949	297,083499	-1,013784064	0,23293191	-4,3522764	1,35E-05	0,0001336	HBEGF
ENSGALG00000016616	1435,92487	-1,01551489	0,1675974	-6,0592521	1,37E-09	3,18E-08	
ENSGALG00000009450	2394,04421	-1,016064164	0,12964787	-7,8371065	4,61E-15	2,19E-13	DUSP10
ENSGALG00000001009	527,817322	-1,018620159	0,24676578	-4,1278826	3,66E-05	0,00031509	
ENSGALG00000015896	2100,38648	-1,023057651	0,16795043	-6,0914263	1,12E-09	2,64E-08	
ENSGALG00000010156	274,639072	-1,025083823	0,24796248	-4,134028	3,56E-05	0,00030892	
ENSGALG00000013124	3974,90345	-1,026819624	0,16230106	-6,3266353	2,51E-10	6,40E-09	FHOD3
ENSGALG00000006138	2396,05253	-1,026936244	0,15045199	-6,825674	8,75E-12	2,79E-10	
ENSGALG00000010579	4573,02342	-1,028169899	0,12570944	-8,1789393	2,86E-16	1,62E-14	STON2
ENSGALG00000012439	1314,57587	-1,028638845	0,22792485	-4,5130614	6,39E-06	6,86E-05	
ENSGALG00000019077	11786,6675	-1,030078317	0,1385218	-7,436218	1,04E-13	4,25E-12	
ENSGALG00000000977	7324,72056	-1,032464943	0,10973117	-9,4090394	5,01E-21	4,29E-19	MAPKAP1
ENSGALG00000016754	1717,85966	-1,034769759	0,17703324	-5,8450593	5,06E-09	1,06E-07	
ENSGALG00000012412	7272,99248	-1,036824012	0,14525293	-7,1380593	9,47E-13	3,47E-11	MTHFD1L
ENSGALG00000027213	84,2176986	-1,037130822	0,33646244	-3,0824565	0,002053	0,00965178	ALX1
ENSGALG00000007555	13407,2897	-1,038877738	0,11478529	-9,050617	1,42E-19	1,11E-17	CCND1
ENSGALG00000008465	8001,13375	-1,045042375	0,12988092	-8,0461579	8,54E-16	4,60E-14	SORCS1
ENSGALG00000022800	1489,66503	-1,045072381	0,14566903	-7,1742935	7,27E-13	2,72E-11	
ENSGALG00000017714	665,00414	-1,052113718	0,20833312	-5,050151	4,41E-07	6,20E-06	
ENSGALG00000015975	5069,41908	-1,056406602	0,12845648	-8,2238485	1,97E-16	1,13E-14	
ENSGALG00000011581	10286,8368	-1,059360643	0,33858403	-3,1287969	0,00175524	0,00846159	KIF26A
ENSGALG00000015736	279,628079	-1,060236168	0,31923414	-3,3211867	0,00089636	0,00488488	
ENSGALG00000003295	2261,70268	-1,063705711	0,15858273	-6,7075762	1,98E-11	6,00E-10	
ENSGALG00000009740	783,871387	-1,063900656	0,18158266	-5,8590432	4,66E-09	9,86E-08	RASGRP1
ENSGALG00000013604	164,158201	-1,065244459	0,28526693	-3,7342024	0,00018831	0,0012975	VIP
ENSGALG00000011236	190,29168	-1,070413941	0,31505499	-3,3975464	0,00067993	0,00388308	BEGAIN
ENSGALG00000017135	1406,42375	-1,071209509	0,16120082	-6,6451865	3,03E-11	8,92E-10	CRYL1

ENSGALG00000002988	7561,04394	-1,072357906	0,15209899	-7,0503948	1,78E-12	6,33E-11	PHGDH
ENSGALG00000001370	1171,23107	-1,073396904	0,19159469	-5,6024356	2,11E-08	3,91E-07	ADAMTS8
ENSGALG00000027757	10827,9262	-1,076478944	0,10801659	-9,9658664	2,15E-23	2,27E-21	ARL4A
ENSGALG00000010891	1573,41259	-1,079203445	0,18453813	-5,8481327	4,97E-09	1,04E-07	ABCB11
ENSGALG00000027707	112,772942	-1,082314729	0,31181061	-3,4710644	0,0005184	0,0030902	ESM1
ENSGALG00000016899	32,6413126	-1,08275528	0,35267355	-3,0701346	0,00213962	0,00998662	DCT
ENSGALG00000000177	4145,02133	-1,083701806	0,12196683	-8,8852173	6,38E-19	4,70E-17	
ENSGALG00000025770	345,961225	-1,089960517	0,25250889	-4,3165234	1,59E-05	0,00015316	
ENSGALG00000028046	6843,87336	-1,098357134	0,11398468	-9,6360065	5,63E-22	5,11E-20	INSIG2
ENSGALG00000012917	4548,57829	-1,102419391	0,19071062	-5,7805874	7,44E-09	1,50E-07	CDH6
ENSGALG00000005842	443,936227	-1,104947048	0,2118757	-5,215072	1,84E-07	2,80E-06	GDPD2
ENSGALG00000012095	202,109683	-1,108243378	0,27163773	-4,079858	4,51E-05	0,00037525	C14orf105
ENSGALG00000002518	128,674403	-1,109129449	0,33692493	-3,2919186	0,00099506	0,00531078	
ENSGALG00000009567	5248,50516	-1,113206952	0,1654659	-6,7277124	1,72E-11	5,27E-10	TLL1
ENSGALG00000011439	1241,39048	-1,116853678	0,16224057	-6,8839359	5,82E-12	1,90E-10	
ENSGALG00000013548	35,2312467	-1,118469658	0,33863762	-3,3028512	0,00095707	0,00515652	GZMA
ENSGALG00000014852	15743,6418	-1,119019515	0,11136376	-10,048327	9,34E-24	1,05E-21	
ENSGALG00000009179	4347,97295	-1,119360692	0,13379231	-8,3664053	5,94E-17	3,63E-15	TNFSF10
ENSGALG00000011172	2541,5225	-1,121518184	0,12741815	-8,8018714	1,35E-18	9,35E-17	
ENSGALG00000013892	1340,40625	-1,122648268	0,1612643	-6,9615422	3,37E-12	1,14E-10	MAP3K5
ENSGALG00000001079	117,890946	-1,122757301	0,33560094	-3,345513	0,00082131	0,0045345	WNT3
ENSGALG00000016680	793,768127	-1,12428894	0,23420199	-4,8005098	1,58E-06	1,97E-05	TFAP2B
ENSGALG00000008014	284,580595	-1,126068911	0,26416635	-4,2627266	2,02E-05	0,00018923	CEBPB
ENSGALG00000006337	35,9815104	-1,128489411	0,34919354	-3,2317018	0,00123055	0,00634861	
ENSGALG00000020151	65,1037326	-1,129234483	0,35223689	-3,205895	0,00134643	0,0068343	NMU
ENSGALG00000006376	107,4182	-1,138122247	0,32739087	-3,4763409	0,00050831	0,00303934	TMC3
ENSGALG00000027210	288,324353	-1,142415301	0,23806373	-4,7987792	1,60E-06	1,98E-05	
ENSGALG00000001085	2988,78652	-1,143093103	0,13119308	-8,7130596	2,96E-18	1,98E-16	
ENSGALG00000001276	18773,5176	-1,144775826	0,13161296	-8,6980477	3,38E-18	2,24E-16	HOXB9
ENSGALG00000004155	218,309793	-1,147734088	0,27623338	-4,1549435	3,25E-05	0,00028451	MYOM3
ENSGALG00000027107	1206,95445	-1,149521147	0,15677875	-7,3321234	2,27E-13	9,02E-12	
ENSGALG00000014864	769,880572	-1,150010609	0,17012701	-6,7597182	1,38E-11	4,28E-10	
ENSGALG00000027885	31,9835607	-1,151437329	0,34943778	-3,295114	0,00098382	0,00526107	C4orf19
ENSGALG00000010926	145,649999	-1,152009571	0,31597679	-3,6458677	0,00026649	0,00175025	
ENSGALG00000008875	1752,66896	-1,156831558	0,15899216	-7,2760291	3,44E-13	1,34E-11	PLCB1
ENSGALG00000007268	3702,32926	-1,168892699	0,14170635	-8,2486969	1,60E-16	9,28E-15	
ENSGALG00000006584	535,759637	-1,169466644	0,23137239	-5,054478	4,32E-07	6,07E-06	
ENSGALG00000016114	1343,20194	-1,174926553	0,19801604	-5,9334919	2,97E-09	6,53E-08	
ENSGALG00000022871	818,020575	-1,176605905	0,18677065	-6,2997367	2,98E-10	7,55E-09	
ENSGALG00000026763	724,902739	-1,177313255	0,20694833	-5,6889236	1,28E-08	2,48E-07	
ENSGALG00000017411	73,3262064	-1,17758282	0,34864579	-3,3775909	0,00073124	0,00412419	RXFP3
ENSGALG00000016998	449,62073	-1,177970615	0,21440175	-5,494221	3,92E-08	6,86E-07	LPAR6
ENSGALG00000008941	29,8088387	-1,184874519	0,3508426	-3,3772253	0,00073221	0,00412626	FEZF1
ENSGALG00000002509	14719,4349	-1,185807324	0,13269584	-8,9362812	4,02E-19	2,98E-17	
ENSGALG00000012357	5240,54414	-1,187472008	0,12252712	-9,6915034	3,28E-22	3,16E-20	CXCR4
ENSGALG00000015665	1052,14248	-1,188036426	0,17158557	-6,9238716	4,39E-12	1,45E-10	SH3BP2
ENSGALG00000005619	863,255886	-1,189698364	0,2239703	-5,3118577	1,09E-07	1,73E-06	
ENSGALG00000005685	3302,12612	-1,189953127	0,15706062	-7,576394	3,55E-14	1,57E-12	KSR1
ENSGALG00000016083	216,722511	-1,190269979	0,27117041	-4,38938	1,14E-05	0,0001155	
ENSGALG00000000936	1054,13064	-1,193444435	0,15734156	-7,5850553	3,32E-14	1,47E-12	
ENSGALG00000006170	3710,82448	-1,194070483	0,14887865	-8,0204281	1,05E-15	5,52E-14	LRRC8C
ENSGALG00000015433	4115,11204	-1,199115509	0,12542332	-9,560547	1,17E-21	1,05E-19	ABCA1
ENSGALG00000002923	1146,0005	-1,199213505	0,15769125	-7,6048196	2,85E-14	1,29E-12	
ENSGALG00000011104	29,5888843	-1,203717219	0,35160223	-3,4235198	0,00061816	0,00358289	GCG
ENSGALG00000012224	1928,96823	-1,20528636	0,15139325	-7,961295	1,70E-15	8,65E-14	
ENSGALG00000003582	6407,42119	-1,218636528	0,17846722	-6,8283493	8,59E-12	2,75E-10	SLC1A3
ENSGALG00000009667	660,393085	-1,219318734	0,24952478	-4,8865638	1,03E-06	1,33E-05	MFAP3L
ENSGALG00000002632	1307,71653	-1,220611399	0,17395739	-7,0167265	2,27E-12	7,93E-11	CALCRL
ENSGALG00000009215	122,787447	-1,220936874	0,30573548	-3,9934419	6,51E-05	0,00051668	
ENSGALG00000000141	479,975266	-1,223136562	0,25876918	-4,7267474	2,28E-06	2,72E-05	BLB1
ENSGALG00000006473	340,04191	-1,223226887	0,26548467	-4,6075237	4,07E-06	4,58E-05	PLXNA4
ENSGALG00000007660	3604,79456	-1,228207524	0,16378808	-7,4987599	6,44E-14	2,69E-12	

ENSGALG00000013239	67578,5287	-1,231185989	0,13442151	-9,1591442	5,23E-20	4,24E-18	
ENSGALG00000008615	1056,6531	-1,233824562	0,18606143	-6,6312754	3,33E-11	9,74E-10	
ENSGALG00000003739	39854,6748	-1,237172502	0,12123912	-10,2044	1,89E-24	2,28E-22	
ENSGALG00000029023	187,057616	-1,242039059	0,26924919	-4,6129723	3,97E-06	4,48E-05	DMRTB1
ENSGALG00000009532	9821,7952	-1,242374232	0,11357599	-10,938705	7,53E-28	1,13E-25	GXYLT1
ENSGALG00000011867	2059,31955	-1,242989678	0,14218556	-8,7420246	2,29E-18	1,56E-16	SERHL2
ENSGALG00000005427	736,096188	-1,25622691	0,18500374	-6,7902783	1,12E-11	3,51E-10	
ENSGALG00000013237	693,51796	-1,260226776	0,21528589	-5,8537361	4,81E-09	1,01E-07	
ENSGALG00000010858	12929,1308	-1,268608971	0,32118282	-3,9498033	7,82E-05	0,00060506	LRP2
ENSGALG00000003353	12512,0835	-1,269046359	0,17509801	-7,2476344	4,24E-13	1,63E-11	RAI14
ENSGALG00000019552	24,9825451	-1,269295425	0,34407756	-3,6889806	0,00022515	0,00151456	SERPINB2
ENSGALG00000004361	87800,4537	-1,276561341	0,12683773	-10,064524	7,93E-24	8,99E-22	
ENSGALG00000008352	151,362805	-1,283975751	0,29199529	-4,3972481	1,10E-05	0,00011206	
ENSGALG00000002397	837,302305	-1,291814757	0,2176946	-5,9340689	2,96E-09	6,52E-08	PSPH
ENSGALG00000006401	4187,88984	-1,299022844	0,12943055	-10,036447	1,05E-23	1,16E-21	OSBPL5
ENSGALG00000014866	52452,7894	-1,312013425	0,13134051	-9,9894042	1,70E-23	1,83E-21	
ENSGALG00000005442	270,987771	-1,327990169	0,28317276	-4,6896819	2,74E-06	3,20E-05	PALMD
ENSGALG00000016602	1659,68203	-1,339565689	0,16372183	-8,1819615	2,79E-16	1,58E-14	
ENSGALG00000021879	40,0432745	-1,34129965	0,34849488	-3,8488361	0,00011868	0,00086593	
ENSGALG00000002443	492,983426	-1,353430658	0,22722109	-5,9564481	2,58E-09	5,77E-08	FBXL16
ENSGALG00000007771	2716,44612	-1,353742442	0,15148338	-8,9365738	4,01E-19	2,98E-17	FAM210B
ENSGALG00000008491	3635,92779	-1,354708919	0,1393335	-9,7227798	2,41E-22	2,38E-20	
ENSGALG00000009947	4729,08145	-1,358616298	0,11919082	-11,398666	4,25E-30	7,32E-28	PLEKHH2
ENSGALG00000001346	421,970817	-1,362109597	0,2357782	-5,7770803	7,60E-09	1,53E-07	
ENSGALG00000028048	17805,9742	-1,362450241	0,10389675	-13,113502	2,76E-39	8,73E-37	
ENSGALG00000028047	613,76602	-1,369008166	0,2271663	-6,026458	1,68E-09	3,86E-08	RHOV
ENSGALG00000010148	96,0680074	-1,379685752	0,32859473	-4,1987459	2,68E-05	0,00024168	
ENSGALG00000003706	2880,21855	-1,390323026	0,15220073	-9,1347989	6,55E-20	5,25E-18	
ENSGALG00000009405	351,467766	-1,391691484	0,2252283	-6,1790258	6,45E-10	1,57E-08	GRIA2
ENSGALG00000014983	1427,7077	-1,39278689	0,17525642	-7,9471375	1,91E-15	9,66E-14	F2R
ENSGALG00000002993	1938,95885	-1,397301236	0,15816908	-8,834225	1,01E-18	7,22E-17	CA10
ENSGALG00000015256	9909,97899	-1,397562325	0,12338907	-11,326468	9,70E-30	1,61E-27	PAM
ENSGALG00000003114	426,741711	-1,398035927	0,21620264	-6,4663221	1,00E-10	2,72E-09	NOG
ENSGALG00000011306	2909,70938	-1,403496478	0,13240055	-10,600382	2,97E-26	4,17E-24	DES
ENSGALG00000027133	162,089324	-1,405319409	0,31973	-4,3953317	1,11E-05	0,00011297	
ENSGALG00000001768	6648,81239	-1,406520646	0,15682598	-8,9686713	3,00E-19	2,27E-17	TENM2
ENSGALG00000005123	593,996105	-1,408198856	0,22248629	-6,3293737	2,46E-10	6,31E-09	WNT7A
ENSGALG00000000680	6447,75978	-1,411570576	0,1125835	-12,537988	4,63E-36	1,19E-33	
ENSGALG00000009530	697,310671	-1,411853584	0,1982451	-7,1217577	1,07E-12	3,88E-11	PDZRN4
ENSGALG00000014343	783,229712	-1,412639292	0,1787261	-7,9039341	2,70E-15	1,32E-13	
ENSGALG00000026557	168,826381	-1,412974627	0,2911389	-4,8532664	1,21E-06	1,55E-05	PGF
ENSGALG00000011319	10478,7818	-1,42017882	0,12658897	-11,218819	3,30E-29	5,34E-27	ADGRL3
ENSGALG00000014823	40,5162501	-1,422401118	0,34565713	-4,1150637	3,87E-05	0,00033124	
ENSGALG00000011468	1760,29824	-1,424650779	0,18752858	-7,5969793	3,03E-14	1,36E-12	IGFBP5
ENSGALG00000016297	52,4647782	-1,427639697	0,35390644	-4,0339466	5,48E-05	0,00044582	HCRTR2
ENSGALG00000019054	529,716619	-1,427749976	0,24150691	-5,911839	3,38E-09	7,38E-08	
ENSGALG00000011110	296,257233	-1,431359085	0,24880708	-5,7528873	8,77E-09	1,75E-07	
ENSGALG00000011207	13680,733	-1,434035643	0,1207094	-11,880066	1,50E-32	3,30E-30	DUSP6
ENSGALG00000012018	2349,85185	-1,435600968	0,18691445	-7,6805242	1,58E-14	7,29E-13	UGT8
ENSGALG00000013402	51,692715	-1,446510354	0,35385338	-4,0878805	4,35E-05	0,00036362	AHRR
ENSGALG00000011164	2326,41675	-1,446841804	0,20831238	-6,9455393	3,77E-12	1,26E-10	CHN2
ENSGALG00000028821	11178,852	-1,46102831	0,14885453	-9,8151415	9,69E-23	9,70E-21	GLIPR2
ENSGALG00000001169	20569,632	-1,469628578	0,14537593	-10,10916	5,03E-24	5,85E-22	CRB2
ENSGALG00000006072	43,451296	-1,471939488	0,35393821	-4,1587471	3,20E-05	0,0002809	SCN10A
ENSGALG00000005855	6149,83129	-1,488520056	0,16245344	-9,1627487	5,06E-20	4,12E-18	
ENSGALG00000026262	5433,56566	-1,4922838	0,15452733	-9,6570863	4,59E-22	4,25E-20	
ENSGALG00000001621	458,454887	-1,495017381	0,22098058	-6,7653791	1,33E-11	4,12E-10	CLDN5
ENSGALG00000011940	614,502923	-1,496267605	0,21912179	-6,8284747	8,58E-12	2,75E-10	
ENSGALG00000008978	15989,3853	-1,505709648	0,13184171	-11,420587	3,30E-30	5,76E-28	
ENSGALG00000025773	391,159032	-1,506718569	0,21938214	-6,8680093	6,51E-12	2,11E-10	
ENSGALG00000015388	3557,58163	-1,507384513	0,13257512	-11,370041	5,90E-30	1,00E-27	
ENSGALG00000007171	156,082312	-1,518198388	0,29373826	-5,1685414	2,36E-07	3,50E-06	CIITA

ENSGALG00000010258	1087,70771	-1,528410178	0,23204878	-6,5865901	4,50E-11	1,28E-09	LTBP2
ENSGALG00000006084	149,653293	-1,531133293	0,30821845	-4,9676886	6,78E-07	9,10E-06	
ENSGALG00000005499	52125,7921	-1,531899327	0,15487524	-9,8911829	4,55E-23	4,65E-21	IL17RD
ENSGALG00000001383	88,9612133	-1,543511015	0,34200221	-4,5131609	6,39E-06	6,86E-05	HTR4
ENSGALG00000017179	397,23584	-1,548130217	0,2756174	-5,6169539	1,94E-08	3,65E-07	PDGFD
ENSGALG00000000388	10384,9656	-1,555950834	0,15057476	-10,333411	4,98E-25	6,33E-23	LIN28A
ENSGALG00000016293	1486,8847	-1,561397543	0,15239221	-10,245914	1,23E-24	1,51E-22	BMP5
ENSGALG00000014657	1240,68069	-1,561923173	0,21313827	-7,3282155	2,33E-13	9,26E-12	
ENSGALG00000003957	1745,61798	-1,565814231	0,14958689	-10,46759	1,22E-25	1,66E-23	
ENSGALG00000017242	1988,33091	-1,579394776	0,13593267	-11,618949	3,30E-31	6,42E-29	FZD4
ENSGALG00000009170	1955,30232	-1,581054934	0,13559524	-11,660106	2,04E-31	4,02E-29	NCEH1
ENSGALG00000003323	100,589751	-1,591926379	0,32090266	-4,9607765	7,02E-07	9,41E-06	OSGIN1
ENSGALG00000001645	733,714958	-1,608468631	0,20509442	-7,8425762	4,41E-15	2,10E-13	KCNT1
ENSGALG00000011609	3466,23734	-1,61436812	0,3265448	-4,9437876	7,66E-07	1,02E-05	
ENSGALG00000003126	1209,63641	-1,615974863	0,16174479	-9,990893	1,67E-23	1,82E-21	ERBB4
ENSGALG00000019716	86,528621	-1,618712644	0,32858792	-4,9262694	8,38E-07	1,10E-05	KRT20
ENSGALG00000016102	256,085293	-1,630912312	0,24814594	-6,5723916	4,95E-11	1,41E-09	
ENSGALG00000010468	1107,42968	-1,6309453	0,15736024	-10,364405	3,60E-25	4,67E-23	ISM2
ENSGALG00000023521	1102,90608	-1,651708778	0,19829188	-8,3296843	8,11E-17	4,84E-15	ADRA2A
ENSGALG00000019261	1018,73464	-1,655584971	0,22406874	-7,3887368	1,48E-13	6,04E-12	
ENSGALG00000026892	80,0076125	-1,6734567	0,34940022	-4,7895124	1,67E-06	2,06E-05	
ENSGALG00000008353	2780,09542	-1,679225856	0,19226914	-8,7337255	2,46E-18	1,67E-16	
ENSGALG00000006997	745,789097	-1,684979264	0,17360387	-9,7058852	2,85E-22	2,77E-20	BRINP1
ENSGALG00000029175	12384,2511	-1,685259625	0,16604046	-10,149693	3,32E-24	3,90E-22	EFNB2
ENSGALG00000008918	724,315184	-1,685987772	0,1865069	-9,0398142	1,57E-19	1,22E-17	LAMP5
ENSGALG00000014930	4308,58674	-1,696897635	0,15640418	-10,849439	2,01E-27	2,94E-25	ENC1
ENSGALG00000005498	1151,84629	-1,707286081	0,21532932	-7,928721	2,21E-15	1,11E-13	
ENSGALG00000014235	549,444331	-1,721709963	0,26416786	-6,5174846	7,15E-11	1,99E-09	
ENSGALG00000013102	1811,9189	-1,724325032	0,13966812	-12,345874	5,13E-35	1,25E-32	COBL
ENSGALG00000007218	6209,27212	-1,732428983	0,17353793	-9,9829988	1,81E-23	1,94E-21	MB21D2
ENSGALG00000014908	2604,03468	-1,732825245	0,16330677	-10,61086	2,65E-26	3,76E-24	FST
ENSGALG00000005476	213,71162	-1,752170936	0,31806755	-5,5088013	3,61E-08	6,35E-07	PTH1R
ENSGALG00000011148	2471,49648	-1,754606037	0,1499315	-11,702718	1,23E-31	2,51E-29	
ENSGALG00000027874	793,364535	-1,754956403	0,23265502	-7,54317	4,59E-14	1,97E-12	CHAC1
ENSGALG00000012635	1110,85351	-1,755097361	0,16645491	-10,543981	5,42E-26	7,45E-24	
ENSGALG00000006995	175,827248	-1,759714805	0,30239363	-5,8192853	5,91E-09	1,22E-07	APOD
ENSGALG00000027316	126,113792	-1,764476803	0,31211141	-5,6533557	1,57E-08	3,00E-07	
ENSGALG00000005519	2075,75095	-1,767947956	0,33351026	-5,3010302	1,15E-07	1,82E-06	TLL2
ENSGALG00000009282	5108,51903	-1,769054515	0,16415004	-10,777058	4,42E-27	6,40E-25	ELMSAN1
ENSGALG00000007113	2619,56625	-1,77145981	0,19094943	-9,277115	1,74E-20	1,45E-18	
ENSGALG00000015148	101,324198	-1,772924708	0,34687715	-5,1111027	3,20E-07	4,62E-06	ANXA1
ENSGALG00000023406	3748,56647	-1,774894708	0,1767754	-10,040394	1,01E-23	1,12E-21	
ENSGALG00000004930	259,836288	-1,782009807	0,32603449	-5,4657095	4,61E-08	7,94E-07	HRH1
ENSGALG00000009920	1810,73161	-1,80046145	0,20594812	-8,742306	2,28E-18	1,56E-16	COCH
ENSGALG00000014159	18203,792	-1,81424576	0,10616787	-17,088464	1,81E-65	1,30E-62	PACSIN2
ENSGALG00000016875	1624,38522	-1,825016077	0,18553233	-9,836647	7,83E-23	7,89E-21	
ENSGALG00000004306	1596,76443	-1,830041848	0,15669253	-11,67919	1,63E-31	3,26E-29	SYT12
ENSGALG00000011708	13494,0054	-1,833902608	0,12124784	-15,12524	1,10E-51	6,01E-49	MYLK
ENSGALG00000002714	18305,7311	-1,839377527	0,12313583	-14,937793	1,87E-50	9,10E-48	TMEM132C
ENSGALG00000007963	269,370346	-1,84239576	0,2523089	-7,3021434	2,83E-13	1,11E-11	NFATC2
ENSGALG00000014935	24043,958	-1,864724119	0,16406086	-11,366051	6,17E-30	1,04E-27	GREB1L
ENSGALG00000015089	1244,38805	-1,867848339	0,21666451	-8,6209242	6,64E-18	4,39E-16	GAP43
ENSGALG00000016906	16521,3421	-1,870006943	0,10236052	-18,268829	1,47E-74	2,00E-71	SPRY2
ENSGALG00000012392	124,624801	-1,879813042	0,30530777	-6,1571084	7,41E-10	1,79E-08	IYD
ENSGALG00000007786	809,873559	-1,895122662	0,25833391	-7,3359423	2,20E-13	8,79E-12	DOK5
ENSGALG00000003437	453,52356	-1,896224168	0,2236049	-8,4802444	2,25E-17	1,43E-15	
ENSGALG00000009693	162,98636	-1,908196694	0,28491477	-6,69743	2,12E-11	6,38E-10	CPXM2
ENSGALG00000026366	659,496965	-1,956428809	0,25593066	-7,6443705	2,10E-14	9,56E-13	
ENSGALG00000016377	39318,7205	-1,984049771	0,15160151	-13,087269	3,89E-39	1,20E-36	PXDN
ENSGALG00000007646	1162,43082	-1,999886692	0,2341543	-8,5408926	1,33E-17	8,72E-16	
ENSGALG00000014727	707,495402	-2,02826974	0,22579434	-8,982819	2,64E-19	2,02E-17	PDE4D
ENSGALG00000025941	508,274649	-2,032822244	0,21082252	-9,6423394	5,30E-22	4,84E-20	RGS20

ENSGALG00000016455	36148,3013	-2,034647249	0,14156144	-14,372892	7,66E-47	3,07E-44	GREB1
ENSGALG00000015332	1189,98649	-2,044938557	0,2318737	-8,8191915	1,15E-18	8,09E-17	CPLX1
ENSGALG00000001608	473,981577	-2,081100043	0,22971307	-9,059563	1,31E-19	1,03E-17	UNC5D
ENSGALG00000006797	719,73345	-2,093089949	0,18254835	-11,465949	1,96E-30	3,60E-28	ADARB2
ENSGALG00000005347	2547,96798	-2,102469906	0,17281317	-12,166144	4,71E-34	1,11E-31	ADAMTS18
ENSGALG00000005061	4294,17572	-2,114741441	0,15753482	-13,423962	4,38E-41	1,53E-38	PPM1E
ENSGALG00000006822	6099,69686	-2,117548167	0,19086302	-11,094596	1,33E-28	2,06E-26	PCDH19
ENSGALG00000002540	1690,5022	-2,132167559	0,1444055	-14,765141	2,46E-49	1,08E-46	
ENSGALG00000014872	706,849976	-2,137267919	0,21173492	-10,094074	5,87E-24	6,77E-22	FGF10
ENSGALG00000005000	10982,0355	-2,158647655	0,15123051	-14,27389	3,18E-46	1,24E-43	COMTD1
ENSGALG00000002816	1397,74927	-2,170634895	0,17254974	-12,579763	2,73E-36	7,28E-34	STK10
ENSGALG00000024083	2014,38425	-2,175833689	0,16411916	-13,257646	4,07E-40	1,39E-37	C8orf4
ENSGALG00000017244	1712,46975	-2,189436741	0,14753276	-14,840343	8,03E-50	3,65E-47	PRSS23
ENSGALG00000012106	326,650164	-2,236831516	0,28466041	-7,8578946	3,91E-15	1,87E-13	SCTR
ENSGALG00000005689	443,468063	-2,271345118	0,23720344	-9,575515	1,01E-21	9,14E-20	Pax-2
ENSGALG00000000600	217,113311	-2,27272638	0,28275559	-8,0377769	9,15E-16	4,85E-14	
ENSGALG00000019426	136,015643	-2,280006749	0,30033938	-7,5914345	3,16E-14	1,41E-12	
ENSGALG00000012594	465,978763	-2,283939186	0,22484628	-10,157781	3,06E-24	3,62E-22	NTRK2
ENSGALG00000012732	358,709185	-2,298252682	0,22771061	-10,092866	5,94E-24	6,80E-22	PHACTR1
ENSGALG00000004376	1102,6462	-2,323750078	0,19821753	-11,723232	9,69E-32	2,00E-29	KCNJ2
ENSGALG00000008118	2184,0618	-2,340544818	0,13264091	-17,645724	1,10E-69	1,15E-66	
ENSGALG00000010435	1040,62618	-2,340611141	0,22142593	-10,570628	4,08E-26	5,66E-24	RASGRP3
ENSGALG00000007346	9971,3513	-2,361764382	0,15659212	-15,082268	2,12E-51	1,11E-48	KIAA1462
ENSGALG00000017091	828,298355	-2,363903034	0,17074536	-13,844611	1,37E-43	5,05E-41	FLT1
ENSGALG00000012278	11240,1658	-2,383840075	0,16389202	-14,545187	6,27E-48	2,67E-45	
ENSGALG00000016785	93,1133081	-2,405348378	0,32963393	-7,2970291	2,94E-13	1,15E-11	IL1RL1
ENSGALG00000013033	107,445867	-2,410715686	0,31956021	-7,5438544	4,56E-14	1,97E-12	
ENSGALG00000007657	4917,86859	-2,412172788	0,33438082	-7,2138491	5,44E-13	2,06E-11	CDX4
ENSGALG00000012927	231,318015	-2,419967236	0,26578914	-9,1048386	8,64E-20	6,84E-18	
ENSGALG00000002183	431,198329	-2,438499895	0,26607955	-9,1645522	4,98E-20	4,08E-18	PRR16
ENSGALG00000015947	963,910906	-2,438977585	0,18916847	-12,893151	4,92E-38	1,46E-35	
ENSGALG00000008893	301,746209	-2,444595373	0,24691931	-9,9003817	4,15E-23	4,31E-21	
ENSGALG00000008890	114,859766	-2,449553325	0,32616483	-7,5101701	5,91E-14	2,49E-12	ADGRL4
ENSGALG00000008905	317,218524	-2,456637668	0,23731673	-10,351726	4,11E-25	5,28E-23	HABP2
ENSGALG00000026663	524,422448	-2,478656591	0,20922664	-11,846755	2,24E-32	4,76E-30	CX3CL1
ENSGALG00000012623	104,28468	-2,482707417	0,32178291	-7,7154733	1,21E-14	5,58E-13	
ENSGALG00000015826	274,530912	-2,484029553	0,31827956	-7,8045526	5,97E-15	2,81E-13	HTR1E
ENSGALG00000010224	221,988072	-2,486198557	0,29117325	-8,5385541	1,36E-17	8,83E-16	
ENSGALG00000007940	128,052277	-2,542363859	0,32047998	-7,9329879	2,14E-15	1,07E-13	
ENSGALG00000001560	195,37323	-2,555527127	0,33829668	-7,5541005	4,22E-14	1,83E-12	
ENSGALG00000008581	16731,9435	-2,566439645	0,14745274	-17,405167	7,54E-68	5,70E-65	DUSP5
ENSGALG00000016784	1039,85153	-2,607549769	0,17414233	-14,97367	1,09E-50	5,50E-48	
ENSGALG00000006112	988,653043	-2,692857061	0,18990629	-14,179926	1,22E-45	4,61E-43	SCN5A
ENSGALG00000016154	140,890158	-2,742713554	0,30649601	-8,948611	3,60E-19	2,71E-17	ARC
ENSGALG00000016145	1265,10116	-2,754137035	0,17459535	-15,774401	4,67E-56	2,76E-53	
ENSGALG00000011099	581,916765	-2,760215905	0,26996697	-10,224273	1,54E-24	1,88E-22	FAP
ENSGALG00000005209	4151,24313	-2,791291062	0,34877784	-8,0030631	1,21E-15	6,28E-14	AQP1
ENSGALG00000011562	1031,63999	-2,817896047	0,18091223	-15,576039	1,06E-54	6,01E-52	PARK2
ENSGALG00000007656	17151,759	-2,823200316	0,30126808	-9,3710569	7,18E-21	6,11E-19	
ENSGALG00000012051	829,367255	-2,878544854	0,24824835	-11,595424	4,35E-31	8,22E-29	
ENSGALG00000028250	102,796791	-2,884651456	0,33080912	-8,7199877	2,78E-18	1,88E-16	ANKRD22
ENSGALG00000007701	2491,57183	-2,906313035	0,16540669	-17,57071	4,13E-69	3,75E-66	B3GNT7
ENSGALG00000014984	5902,58959	-2,931476354	0,14956936	-19,599445	1,56E-85	3,04E-82	F2RL1
ENSGALG00000009704	18346,8673	-2,934403789	0,16761639	-17,506664	1,27E-68	1,02E-65	CHST15
ENSGALG00000016140	355,674118	-2,998033733	0,24925729	-12,027868	2,54E-33	5,76E-31	FAM3B
ENSGALG00000015358	3246,18076	-3,063209848	0,14576096	-21,015297	4,75E-98	1,29E-94	MYH15
ENSGALG00000002562	2027,1289	-3,12939254	0,17645164	-17,735129	2,25E-70	2,55E-67	ARAP3
ENSGALG00000007538	106,892168	-3,275953369	0,34614117	-9,4642118	2,96E-21	2,60E-19	
ENSGALG00000022962	106,156937	-3,362304357	0,3346563	-10,047037	9,47E-24	1,06E-21	
ENSGALG00000002594	1003,24058	-3,661637709	0,2050909	-17,853731	2,70E-71	3,35E-68	TFPI
ENSGALG00000013624	4838,43058	-3,6799039	0,14627576	-25,157305	1,18E-139	5,34E-136	
ENSGALG00000006421	916,754331	-3,742853665	0,23704674	-15,789517	3,67E-56	2,27E-53	SLC16A12

ENSGALG00000020359	246,794372	-3,821393345	0,28450232	-13,431853	3,93E-41	1,41E-38	SIX6
ENSGALG00000003456	187,124669	-3,934712358	0,32478027	-12,114998	8,80E-34	2,03E-31	CA12
ENSGALG00000023379	453,271455	-3,949811074	0,23138338	-17,070418	2,46E-65	1,68E-62	F2RL2
ENSGALG00000016431	670,260054	-4,37340061	0,23581962	-18,545533	8,86E-77	1,34E-73	Pax5B
ENSGALG00000014994	310,707645	-5,043766073	0,28682666	-17,584718	3,23E-69	3,14E-66	CRHBP
ENSGALG00000016783	9121,10854	-5,301446621	0,14503512	-36,552847	1,61E-292	2,19E-288	IL1R1
ENSGALG00000004221	898,014845	-5,338632777	0,26991661	-19,778823	4,53E-87	1,03E-83	IL22RA1
