# Supplementary material for: HOXB8 Counteracts MAPK/ERK Oncogenic Signaling in a Chicken Embryo Model of Neoplasia
Source: Int J Mol Sci. 2021 Aug 18;22(16):8911. doi: 10.3390/ijms22168911 (PMC8396257; doi:10.3390/ijms22168911)
Supplement: Supplementary file 1 [file ijms-22-08911-s001.zip › ijms-1277141 Table S2.pdf]

Table S2: MEK1ca vs pCIG differentially regulated genes (False Discovery Rate (padj) <= 0.01).

ID	baseMean	log2FoldChange	lfcSE	stat	pvalue	padj	Symbol
ENSGALG00000016783	8238,73046	6,569509059	0,14790027	44,4185063	0	0	IL1R1
ENSGALG00000026862	13555,7342	4,954899999	0,09919193	49,9526499	0	0	CLDN1
ENSGALG00000013624	4270,69482	4,748606334	0,13766019	34,4951317	9,49E-261	3,96E-257	FAM65b
ENSGALG00000007701	2111,8366	4,453082303	0,15415453	28,8871327	1,73E-183	5,42E-180	B3GNT7
ENSGALG00000023406	2791,74825	4,424329106	0,17768451	24,899914	7,46E-137	1,04E-133	NKX1-2
ENSGALG00000004221	826,015749	4,334100391	0,24063814	18,0108626	1,60E-72	9,10E-70	IL22RA1
ENSGALG00000026263	1163,05249	4,280447225	0,16395242	26,107863	2,97E-150	5,30E-147	RGS8
ENSGALG00000016431	614,504481	4,191515764	0,19877752	21,0864675	1,06E-98	1,02E-95	Pax5
ENSGALG00000006421	822,905958	4,186231336	0,20967318	19,9655067	1,10E-88	8,59E-86	SLC16A12
ENSGALG00000014994	285,756349	4,160137851	0,2463521	16,886959	5,61E-64	2,51E-61	CRHBP
ENSGALG00000016145	1063,31727	3,924548833	0,20804729	18,8637341	2,27E-79	1,35E-76	RIPK4
ENSGALG00000016906	12704,3465	3,743251462	0,13733846	27,2556682	1,42E-163	3,56E-160	SPRY2
ENSGALG00000020359	223,508759	3,572883143	0,25377029	14,0792017	5,10E-45	1,39E-42	SIX6
ENSGALG00000023379	414,317659	3,494464883	0,23081766	15,139504	8,89E-52	3,09E-49	F2RL2
ENSGALG00000010435	869,92769	3,471549614	0,19291102	17,9956001	2,11E-72	1,15E-69	RASGRP3
ENSGALG00000007656	15062,1816	3,445291545	0,25219864	13,6610236	1,74E-42	4,43E-40	CDX-1
ENSGALG00000005519	1750,48505	3,391598976	0,19583928	17,3182775	3,42E-67	1,65E-64	TLL2
ENSGALG00000026663	441,393045	3,38430006	0,21308555	15,8823535	8,40E-57	3,18E-54	CX3CL1
ENSGALG00000002562	1820,09922	3,3452383	0,1690746	19,785576	3,96E-87	2,92E-84	ARAP3
ENSGALG00000008905	264,878628	3,342984543	0,2406955	13,8888536	7,40E-44	1,93E-41	HABP2
ENSGALG00000016784	893,438424	3,229015878	0,19216246	16,803573	2,30E-63	9,91E-61	IL1RL2
ENSGALG00000008581	13805,345	3,209755496	0,25216846	12,7286158	4,10E-37	9,50E-35	DUSP5
ENSGALG00000011099	516,017104	3,19842177	0,21907483	14,5996772	2,82E-48	8,82E-46	FAP
ENSGALG00000003456	170,923754	3,130034846	0,27207277	11,5044031	1,25E-30	1,98E-28	CA12
ENSGALG00000015358	2958,75231	3,121559149	0,12071098	25,8597779	1,89E-147	2,95E-144	MYH15
ENSGALG00000011940	461,321244	2,983749675	0,21913664	13,6159322	3,22E-42	8,06E-40	
ENSGALG00000007963	212,091292	2,963911322	0,25006993	11,8523298	2,09E-32	3,59E-30	NFATC2
ENSGALG00000023521	856,317445	2,942428455	0,19797705	14,8624723	5,78E-50	1,85E-47	ADRA2A
ENSGALG00000007171	114,627355	2,91119837	0,27426227	10,6146514	2,55E-26	3,32E-24	CIITA
ENSGALG00000005000	9346,89113	2,843605103	0,13435603	21,1646995	2,02E-99	2,11E-96	COMTD1
ENSGALG00000000600	182,137118	2,828234018	0,26246019	10,7758591	4,48E-27	6,15E-25	
ENSGALG00000012018	1787,86628	2,812280515	0,16831135	16,7087992	1,13E-62	4,72E-60	UGT8
ENSGALG00000000177	2934,9664	2,765911854	0,13662864	20,2440117	4,01E-91	3,35E-88	
ENSGALG00000007346	8739,08087	2,763402401	0,14295509	19,330563	2,97E-83	1,96E-80	KIAA1462
ENSGALG00000001621	341,359461	2,731716978	0,25022736	10,9169398	9,57E-28	1,38E-25	CLDN5
ENSGALG00000010224	190,389423	2,688243072	0,2692821	9,9829994	1,81E-23	2,06E-21	
ENSGALG00000009704	16406,1404	2,653508455	0,23537258	11,2736518	1,77E-29	2,70E-27	CHST15
ENSGALG00000005209	3731,94501	2,631256548	0,27714025	9,49431393	2,22E-21	2,10E-19	AQP1
ENSGALG00000014984	5578,94086	2,599579307	0,10540138	24,6636185	2,63E-134	3,29E-131	F2RL1
ENSGALG00000022962	95,9619859	2,592879602	0,27689482	9,3641318	7,67E-21	7,00E-19	
ENSGALG00000002183	379,587253	2,571301428	0,24362434	10,5543701	4,85E-26	6,25E-24	PRR16
ENSGALG00000015388	2544,04823	2,539489467	0,27173379	9,34550488	9,15E-21	8,29E-19	
ENSGALG00000016140	327,169323	2,527534269	0,2417696	10,4543098	1,40E-25	1,79E-23	FAM3B
ENSGALG00000013102	1492,03268	2,517151592	0,12944514	19,4457017	3,17E-84	2,20E-81	COBL
ENSGALG00000005855	4852,14054	2,515154305	0,15070039	16,6897667	1,56E-62	6,28E-60	
ENSGALG00000002816	1217,9424	2,498779812	0,17668612	14,1424792	2,08E-45	5,78E-43	STK10
ENSGALG00000026258	189,462612	2,487122645	0,26495328	9,3870233	6,17E-21	5,68E-19	MAF
ENSGALG00000003126	980,359046	2,475370264	0,15056385	16,440668	9,78E-61	3,82E-58	ERBB4
ENSGALG00000002540	1485,05869	2,44573463	0,13676571	17,8826589	1,61E-71	8,39E-69	
ENSGALG00000015826	238,755372	2,399148661	0,27605492	8,69083812	3,60E-18	2,53E-16	HTR1E
ENSGALG00000007538	97,0573797	2,386772965	0,27690143	8,61957623	6,72E-18	4,64E-16	
ENSGALG00000019054	408,32875	2,38528849	0,2379735	10,0233366	1,20E-23	1,38E-21	
ENSGALG00000026366	561,445489	2,348234721	0,23749642	9,88745309	4,72E-23	5,13E-21	
ENSGALG00000004306	1355,5371	2,335846949	0,15683954	14,893227	3,65E-50	1,20E-47	SYT12
ENSGALG00000009393	548,007386	2,335695464	0,18992639	12,2978985	9,30E-35	1,85E-32	
ENSGALG00000007657	4493,01812	2,332413704	0,26632361	8,75781796	1,99E-18	1,44E-16	CDX4
ENSGALG00000017091	755,513643	2,329587636	0,16022975	14,5390453	6,85E-48	2,09E-45	FLT1
ENSGALG00000011562	980,279996	2,318757284	0,17351326	13,3635736	9,87E-41	2,42E-38	PARK2
ENSGALG00000012051	782,419586	2,272220282	0,23077218	9,84616192	7,12E-23	7,61E-21	

ENSGALG00000011207	11039,4244	2,267210819	0,10235954	22,1494829	1,06E-108	1,20E-105	DUSP6
ENSGALG00000010036	233,218083	2,253052375	0,24656449	9,13778123	6,37E-20	5,35E-18	FOSL2
ENSGALG00000000949	209,314356	2,249487158	0,24647672	9,12657061	7,07E-20	5,90E-18	HBEGF
ENSGALG00000004376	1011,34575	2,243095428	0,17712966	12,6635789	9,41E-37	2,14E-34	KCNJ2
ENSGALG00000007543	311,281076	2,235076903	0,23707019	9,42791198	4,18E-21	3,91E-19	APCDD1L
ENSGALG00000014159	15753,2456	2,234266769	0,08275046	27,0000523	1,48E-160	3,08E-157	PACSIN2
ENSGALG00000016086	151,68942	2,227609215	0,26302517	8,46918638	2,47E-17	1,63E-15	EGR4
ENSGALG00000017244	1556,01813	2,226234853	0,13126312	16,9600944	1,62E-64	7,51E-62	PRSS23
ENSGALG00000007786	691,837015	2,190066734	0,24318575	9,00573619	2,14E-19	1,66E-17	DOK5
ENSGALG00000017242	1658,38116	2,189728881	0,12383488	17,6826506	5,70E-70	2,85E-67	FZD4
ENSGALG00000005061	3913,87544	2,1754071	0,11345305	19,1745135	6,04E-82	3,78E-79	PPM1E
ENSGALG00000005176	768,829888	2,156988496	0,17567574	12,2782377	1,19E-34	2,32E-32	
ENSGALG00000003437	391,254589	2,148196892	0,22078047	9,73001328	2,25E-22	2,28E-20	
ENSGALG00000027275	448,763372	2,082192136	0,23055734	9,03112478	1,70E-19	1,34E-17	
ENSGALG00000005499	41654,758	2,068323361	0,23020452	8,98472101	2,59E-19	1,99E-17	IL17RD
ENSGALG00000006995	146,518321	2,052973523	0,26905137	7,63041466	2,34E-14	1,21E-12	APOD
ENSGALG00000004594	723,032621	2,028493881	0,21261457	9,54070945	1,42E-21	1,37E-19	CD74
ENSGALG00000006112	965,473662	2,027013529	0,17450249	11,6159577	3,42E-31	5,48E-29	SCN5A
ENSGALG00000025941	463,056002	1,970690155	0,19459556	10,1271078	4,19E-24	4,99E-22	RGS20
ENSGALG00000012732	335,645133	1,970234077	0,21232377	9,2793854	1,70E-20	1,49E-18	PHACTR1
ENSGALG00000017179	331,83407	1,969058193	0,24335518	8,09129369	5,90E-16	3,53E-14	PDGFD
ENSGALG00000007940	119,525715	1,939184933	0,27051161	7,16858298	7,58E-13	3,26E-11	
ENSGALG00000011833	10182,5258	1,93191024	0,0952049	20,2921299	1,51E-91	1,35E-88	
ENSGALG00000003323	80,456143	1,918109692	0,2769549	6,92571127	4,34E-12	1,69E-10	OSGIN1
ENSGALG00000012635	985,437941	1,911001153	0,1578461	12,1067365	9,73E-34	1,79E-31	
ENSGALG00000022490	573,744074	1,907440028	0,20504083	9,30273285	1,37E-20	1,21E-18	
ENSGALG00000026154	1182,06867	1,903399885	0,15185043	12,534702	4,82E-36	1,02E-33	CARD19
ENSGALG00000008461	127,705778	1,900360149	0,2708458	7,0163913	2,28E-12	9,25E-11	
ENSGALG00000007415	201,020232	1,899486225	0,25905889	7,33225653	2,26E-13	1,04E-11	
ENSGALG00000000544	59,7220256	1,895252672	0,27550462	6,87920476	6,02E-12	2,30E-10	RAB44
ENSGALG00000016785	81,8603384	1,892879929	0,27682278	6,83787639	8,04E-12	3,02E-10	IL1RL1
ENSGALG00000006447	1443,0601	1,885131113	0,20851752	9,04063661	1,56E-19	1,24E-17	
ENSGALG00000005498	1010,21765	1,873960213	0,20189609	9,28180559	1,67E-20	1,47E-18	
ENSGALG00000008353	2472,76845	1,848707427	0,15762664	11,7283947	9,12E-32	1,50E-29	
ENSGALG00000010005	472,644462	1,841664237	0,22183955	8,30178484	1,03E-16	6,45E-15	EPAS1
ENSGALG00000010597	1857,71605	1,8345583	0,17099128	10,728958	7,44E-27	1,01E-24	
ENSGALG00000012106	297,149921	1,828065766	0,26618963	6,86753188	6,53E-12	2,48E-10	SCTR
ENSGALG00000000141	377,173549	1,818659764	0,24878968	7,31002901	2,67E-13	1,21E-11	BLB1
ENSGALG00000026262	4692,72087	1,788029436	0,15007014	11,9146249	9,93E-33	1,73E-30	
ENSGALG00000010468	963,638886	1,7857319	0,19155321	9,32238057	1,14E-20	1,02E-18	ISM2
ENSGALG00000027365	919,719868	1,784528584	0,14750129	12,0983934	1,08E-33	1,95E-31	HIVEP3
ENSGALG00000003706	2459,1448	1,784490953	0,12794473	13,9473579	3,26E-44	8,69E-42	
ENSGALG00000011581	7771,72761	1,763989645	0,27657569	6,37796348	1,79E-10	5,80E-09	KIF26A
ENSGALG00000023241	912,035255	1,749836088	0,17358051	10,080833	6,72E-24	7,85E-22	
ENSGALG00000027521	220,81723	1,741041239	0,24159087	7,20656876	5,74E-13	2,50E-11	
ENSGALG00000012623	98,1782216	1,738546704	0,2738672	6,3481377	2,18E-10	6,97E-09	
ENSGALG00000029175	11184,2893	1,731471147	0,15062573	11,4951888	1,39E-30	2,18E-28	EFNB2
ENSGALG00000027331	92,8137096	1,731139271	0,27587874	6,27500053	3,50E-10	1,10E-08	
ENSGALG00000001560	177,821121	1,726930021	0,27637082	6,24859752	4,14E-10	1,27E-08	
ENSGALG00000002203	108,392731	1,726038051	0,27079159	6,37404609	1,84E-10	5,92E-09	FGF-18
ENSGALG00000024083	1953,45291	1,719739442	0,15521106	11,0800061	1,57E-28	2,31E-26	C8orf4
ENSGALG00000005069	457,203952	1,702946767	0,18881513	9,01912232	1,90E-19	1,48E-17	
ENSGALG00000005689	438,331804	1,702270541	0,18963661	8,97648701	2,80E-19	2,13E-17	Pax-2
ENSGALG00000009693	145,589702	1,692012083	0,26448256	6,39744289	1,58E-10	5,13E-09	CPXM2
ENSGALG00000015332	921,622061	1,689640626	0,27053693	6,2455083	4,22E-10	1,29E-08	CPLX1
ENSGALG00000006084	123,749559	1,684603389	0,27421003	6,14347832	8,07E-10	2,35E-08	
ENSGALG00000028250	102,045127	1,684255106	0,27227076	6,18595672	6,17E-10	1,83E-08	ANKRD22
ENSGALG00000010258	947,034185	1,674285408	0,222721	7,5174116	5,59E-14	2,75E-12	LTBP2
ENSGALG00000003488	483,411427	1,668824259	0,21466802	7,77397695	7,61E-15	4,14E-13	
ENSGALG00000008281	275,533181	1,659542176	0,23132804	7,1739776	7,28E-13	3,14E-11	NEURL1
ENSGALG00000005263	1264,63713	1,654921404	0,1861074	8,89229227	5,99E-19	4,46E-17	SOX8
ENSGALG00000016500	476,307952	1,652658269	0,1912173	8,64282823	5,48E-18	3,81E-16	FKBP12.6

ENSGALG00000013033	98,9033207	1,649436663	0,27685165	5,95783573	2,56E-09	6,80E-08	
ENSGALG00000009740	628,788388	1,648366239	0,18242751	9,03573286	1,63E-19	1,29E-17	RASGRP1
ENSGALG00000011076	175,435833	1,644995721	0,24871447	6,61399281	3,74E-11	1,29E-09	SYNE3
ENSGALG00000010910	2761,33893	1,643397512	0,13038008	12,6046669	1,99E-36	4,45E-34	
ENSGALG00000001188	5705,77087	1,635057494	0,1139957	14,3431509	1,18E-46	3,42E-44	
ENSGALG00000016602	1430,32585	1,632391617	0,15056739	10,8416017	2,19E-27	3,04E-25	
ENSGALG00000026989	11486,0599	1,621656649	0,10265821	15,7966585	3,28E-56	1,21E-53	HOXA7
ENSGALG00000016377	35584,2442	1,612690665	0,24226856	6,65662393	2,80E-11	9,79E-10	PXDN
ENSGALG00000027218	62,1190209	1,609222298	0,27648022	5,82038856	5,87E-09	1,49E-07	
ENSGALG00000011058	222,479144	1,607120374	0,23535625	6,82845848	8,58E-12	3,20E-10	LEPR
ENSGALG00000008118	2195,31441	1,603231134	0,14691442	10,9126874	1,00E-27	1,43E-25	
ENSGALG00000009282	4772,45996	1,600131022	0,1549655	10,3257244	5,39E-25	6,61E-23	ELMSAN1
ENSGALG00000027707	84,429158	1,597314136	0,27648459	5,77722663	7,59E-09	1,89E-07	ESM1
ENSGALG00000028281	147,102379	1,585304805	0,26452499	5,99302471	2,06E-09	5,54E-08	
ENSGALG00000005347	2499,3111	1,58404156	0,16400145	9,65870473	4,52E-22	4,56E-20	ADAMTS18
ENSGALG00000001383	75,1252537	1,582310757	0,27707894	5,71068578	1,13E-08	2,76E-07	HTR4
ENSGALG00000026484	105,284801	1,578601549	0,27180452	5,80785612	6,33E-09	1,60E-07	
ENSGALG00000008890	110,363216	1,553993643	0,27546286	5,64139075	1,69E-08	4,04E-07	ADGRL4
ENSGALG00000017714	531,051528	1,553191397	0,22067465	7,038377	1,94E-12	8,03E-11	
ENSGALG00000011608	1442,66872	1,548805995	0,27671663	5,59708325	2,18E-08	5,17E-07	INF2
ENSGALG00000011148	2343,39868	1,54566024	0,12322461	12,5434379	4,32E-36	9,31E-34	
ENSGALG00000004730	427,286464	1,54214284	0,20259329	7,61201356	2,70E-14	1,38E-12	
ENSGALG00000011962	5021,1701	1,540068054	0,12375377	12,4446156	1,50E-35	3,12E-33	
ENSGALG00000008875	1473,68406	1,539400497	0,15531993	9,91115904	3,72E-23	4,09E-21	PLCB1
ENSGALG00000001189	175,140381	1,519846307	0,24672052	6,1601942	7,27E-10	2,13E-08	NCF1
ENSGALG00000012847	213,214453	1,514705906	0,24982981	6,06295113	1,34E-09	3,74E-08	
ENSGALG00000016754	1417,60122	1,508945346	0,15769298	9,56888107	1,08E-21	1,06E-19	
ENSGALG00000006997	697,291921	1,507910301	0,16647993	9,05761005	1,33E-19	1,08E-17	BRINP1
ENSGALG00000019426	134,849102	1,491625247	0,25823534	5,77622423	7,64E-09	1,90E-07	
ENSGALG00000016348	4059,56984	1,488129704	0,14344251	10,3743978	3,24E-25	4,06E-23	SAT1
ENSGALG00000011236	148,702178	1,484958654	0,273941	5,42072431	5,94E-08	1,31E-06	BEGAIN
ENSGALG00000002594	917,561594	1,484249601	0,27345121	5,42784068	5,70E-08	1,27E-06	TFPI
ENSGALG00000023751	646,755681	1,484039491	0,2418537	6,13610416	8,46E-10	2,45E-08	
ENSGALG00000014930	4123,64273	1,479606742	0,09900477	14,9448018	1,68E-50	5,69E-48	ENC1
ENSGALG00000010683	477,522338	1,466981653	0,20015393	7,32926728	2,31E-13	1,06E-11	
ENSGALG00000026795	85,4265267	1,459031178	0,27670179	5,27293727	1,34E-07	2,78E-06	
ENSGALG00000007660	3126,72301	1,456310727	0,1563383	9,31512467	1,22E-20	1,09E-18	
ENSGALG00000026251	159,900115	1,452153197	0,27592059	5,26293896	1,42E-07	2,93E-06	
ENSGALG00000028130	618,2391	1,44989129	0,1712474	8,46664711	2,53E-17	1,65E-15	
ENSGALG00000015865	3211,05522	1,445378264	0,11980727	12,0641947	1,63E-33	2,92E-31	
ENSGALG00000009667	556,468113	1,442248321	0,24008414	6,00726204	1,89E-09	5,13E-08	MFAP3L
ENSGALG00000005442	233,433914	1,437319869	0,25444764	5,64878444	1,62E-08	3,89E-07	PALMD
ENSGALG00000007771	2433,98465	1,43708626	0,1405035	10,2281172	1,48E-24	1,78E-22	FAM210B
ENSGALG00000026790	326,80396	1,433302155	0,24589155	5,82900134	5,58E-09	1,42E-07	DMRT2
ENSGALG00000007530	576,29845	1,423961501	0,20873713	6,82179313	8,99E-12	3,33E-10	
ENSGALG00000027210	241,661292	1,422887936	0,22890179	6,2161502	5,10E-10	1,54E-08	
ENSGALG00000008914	65,3744448	1,411144988	0,27696481	5,09503356	3,49E-07	6,83E-06	NRAP
ENSGALG00000007218	5981,23565	1,41095016	0,1549608	9,10520721	8,61E-20	7,09E-18	MB21D2
ENSGALG00000012702	12990,3378	1,407925441	0,12617763	11,1582809	6,52E-29	9,72E-27	JARID2
ENSGALG00000009546	1312,83637	1,402581457	0,15185477	9,23633455	2,55E-20	2,20E-18	
ENSGALG00000006314	830,004543	1,402020858	0,15483246	9,05508371	1,36E-19	1,09E-17	
ENSGALG00000020719	239,712039	1,399243921	0,25114775	5,57139732	2,53E-08	5,93E-07	
ENSGALG00000011468	1596,02721	1,39801296	0,18393319	7,60065615	2,95E-14	1,49E-12	IGFBP5
ENSGALG00000015665	918,715368	1,394518864	0,15230743	9,15594797	5,39E-20	4,55E-18	SH3BP2
ENSGALG00000011680	2741,59968	1,389021273	0,17555553	7,91214743	2,53E-15	1,43E-13	EPHA5
ENSGALG00000010470	502,578729	1,388958129	0,18969021	7,32224451	2,44E-13	1,11E-11	TCERG1L
ENSGALG00000011419	2270,32976	1,382981881	0,11393773	12,1380504	6,64E-34	1,26E-31	DUSP4
ENSGALG00000014852	13662,8714	1,380781721	0,0883531	15,6279945	4,69E-55	1,68E-52	
ENSGALG00000010049	550,482195	1,375590195	0,23963948	5,74024862	9,45E-09	2,33E-07	PTPRB
ENSGALG00000006401	3753,95769	1,372021166	0,13289053	10,3244463	5,46E-25	6,64E-23	OSBPL5
ENSGALG00000002326	668,309268	1,369220123	0,17190144	7,96514611	1,65E-15	9,51E-14	
ENSGALG00000007846	625,227196	1,3613354	0,26060484	5,22375324	1,75E-07	3,55E-06	

ENSGALG00000011120	395,765388	1,356774191	0,22365321	6,06641944	1,31E-09	3,68E-08	
ENSGALG00000004209	184,595238	1,355480435	0,24429813	5,54846835	2,88E-08	6,70E-07	ELFN1
ENSGALG00000014516	1387,85858	1,351178171	0,18357479	7,36036878	1,83E-13	8,47E-12	CPEB2
ENSGALG00000004569	1782,95199	1,348974691	0,16732365	8,06206833	7,50E-16	4,45E-14	UNC5B
ENSGALG00000012911	336,285764	1,345449733	0,24276482	5,54219392	2,99E-08	6,93E-07	SYT10
ENSGALG00000015028	333,710492	1,342064123	0,2166346	6,19505895	5,83E-10	1,74E-08	
ENSGALG00000005476	194,019797	1,338834069	0,27604471	4,85006238	1,23E-06	2,21E-05	PTH1R
ENSGALG00000000107	64,6686363	1,334266505	0,27712884	4,81460712	1,47E-06	2,60E-05	TRIM7
ENSGALG00000006258	7855,034	1,330333906	0,11339109	11,7322618	8,71E-32	1,45E-29	SPOCK1
ENSGALG00000015089	1227,41331	1,327665029	0,20978484	6,32869871	2,47E-10	7,85E-09	GAP43
ENSGALG00000027764	201,777427	1,324450715	0,26757908	4,94975438	7,43E-07	1,38E-05	
ENSGALG00000011110	269,499781	1,322132253	0,23248021	5,6870744	1,29E-08	3,16E-07	
ENSGALG00000008893	314,010022	1,318238187	0,24175367	5,45281554	4,96E-08	1,12E-06	
ENSGALG00000011220	1013,85256	1,316265785	0,15106079	8,7134842	2,95E-18	2,11E-16	SLC25A29
ENSGALG00000005805	3857,09426	1,313014625	0,12265981	10,7045222	9,69E-27	1,29E-24	PLCD1
ENSGALG00000022909	468,481699	1,309497831	0,23797027	5,50277912	3,74E-08	8,55E-07	PTPN3
ENSGALG00000007140	2510,20659	1,309470163	0,26722383	4,90027474	9,57E-07	1,75E-05	NRP1
ENSGALG00000023347	339,64918	1,299000587	0,20361197	6,37978494	1,77E-10	5,75E-09	PTCHD3
ENSGALG00000010311	349,190305	1,29508602	0,2066853	6,26598025	3,70E-10	1,15E-08	NAV3
ENSGALG00000004118	1210,82766	1,294260658	0,15836962	8,17240512	3,02E-16	1,85E-14	
ENSGALG00000002993	1805,52188	1,293663972	0,14239974	9,0847354	1,04E-19	8,44E-18	CA10
ENSGALG00000009808	160,258227	1,293465516	0,26116788	4,952621	7,32E-07	1,36E-05	
ENSGALG00000015256	9263,94872	1,284509548	0,10864322	11,8231908	2,96E-32	5,01E-30	PAM
ENSGALG00000006729	4860,44055	1,281458961	0,27186573	4,713573	2,43E-06	4,09E-05	
ENSGALG00000015908	893,550112	1,276423655	0,19668679	6,48962586	8,60E-11	2,85E-09	COL12A1
ENSGALG00000014727	724,352277	1,275557809	0,20623272	6,18504092	6,21E-10	1,84E-08	PDE4D
ENSGALG00000008263	465,678921	1,273175208	0,20291488	6,27443008	3,51E-10	1,10E-08	CNTN4
ENSGALG00000016817	5327,13148	1,2720896	0,0882221	14,4191717	3,92E-47	1,17E-44	RASA3
ENSGALG00000007253	507,277126	1,27114813	0,17906175	7,09893728	1,26E-12	5,28E-11	IL1RAP
ENSGALG00000009920	1787,73878	1,271106358	0,20301633	6,26110393	3,82E-10	1,18E-08	COCH
ENSGALG00000002632	1174,8881	1,269452785	0,16354378	7,76215854	8,35E-15	4,52E-13	CALCRL
ENSGALG00000016102	216,026386	1,263113327	0,27712691	4,55788769	5,17E-06	8,21E-05	
ENSGALG00000001608	486,701914	1,262511696	0,21788918	5,79428346	6,86E-09	1,72E-07	UNC5D
ENSGALG00000016998	394,580006	1,256730923	0,21467796	5,85402864	4,80E-09	1,23E-07	LPAR6
ENSGALG00000013503	631,301741	1,248703074	0,19424591	6,42846519	1,29E-10	4,21E-09	FAM149A
ENSGALG00000011831	6588,41321	1,24831638	0,0954127	13,0833358	4,10E-39	9,86E-37	ANKRD50
ENSGALG00000000054	9908,86346	1,246870327	0,12340391	10,1039772	5,30E-24	6,26E-22	
ENSGALG00000028047	547,475566	1,243898501	0,24576817	5,06126779	4,16E-07	8,03E-06	RHOV
ENSGALG00000010010	1045,78332	1,237630354	0,14486806	8,54315544	1,31E-17	8,74E-16	RHOQ
ENSGALG00000009947	4438,8697	1,236965236	0,09832671	12,5801542	2,71E-36	5,96E-34	PLEKHH2
ENSGALG00000014983	1347,23546	1,23637443	0,14656523	8,43565994	3,29E-17	2,12E-15	F2R
ENSGALG00000005619	769,181048	1,235271948	0,20759912	5,95027545	2,68E-09	7,08E-08	
ENSGALG00000015653	570,869024	1,234972794	0,196909	6,27179447	3,57E-10	1,11E-08	
ENSGALG00000003114	396,293229	1,234804889	0,20823968	5,92972908	3,03E-09	7,97E-08	NOG
ENSGALG00000019716	79,9556086	1,234170903	0,27584604	4,47412944	7,67E-06	0,00011733	KRT20
ENSGALG00000019261	997,670343	1,230099163	0,20297117	6,06046252	1,36E-09	3,79E-08	
ENSGALG00000028821	10673,5529	1,225133338	0,13787775	8,88564911	6,35E-19	4,70E-17	GLIPR2
ENSGALG00000013902	978,003089	1,221242084	0,22061929	5,53551816	3,10E-08	7,19E-07	
ENSGALG00000008999	1657,80989	1,220108565	0,15349781	7,94870323	1,88E-15	1,07E-13	AFAP1L2
ENSGALG00000014823	35,5546103	1,217853522	0,25543674	4,76773037	1,86E-06	3,21E-05	
ENSGALG00000000307	7560,3549	1,215832976	0,11045819	11,0071785	3,53E-28	5,13E-26	
ENSGALG00000007268	3351,96385	1,214609385	0,1221963	9,93982107	2,79E-23	3,09E-21	
ENSGALG00000000721	3131,94473	1,214440274	0,12625936	9,61861552	6,67E-22	6,57E-20	SLC41A1
ENSGALG00000011061	859,770333	1,212027759	0,15686437	7,72659688	1,10E-14	5,93E-13	
ENSGALG00000007669	6031,34854	1,205315602	0,27706455	4,35030608	1,36E-05	0,00019547	EGR1
ENSGALG00000026557	144,536518	1,203253046	0,27652705	4,35130324	1,35E-05	0,00019503	PGF
ENSGALG00000002555	1133,81707	1,202502668	0,20625382	5,83020812	5,54E-09	1,41E-07	RET
ENSGALG00000027133	142,021318	1,199491369	0,27660527	4,33647334	1,45E-05	0,00020682	
ENSGALG00000024035	1486,34939	1,198706359	0,15977826	7,50231181	6,27E-14	3,08E-12	
ENSGALG00000027070	3188,10078	1,19854946	0,13384164	8,95498217	3,40E-19	2,58E-17	TIMP2
ENSGALG00000001160	934,02292	1,198076321	0,1603006	7,47393516	7,78E-14	3,73E-12	
ENSGALG00000009360	505,831372	1,196193521	0,18849047	6,34617507	2,21E-10	7,04E-09	CAPN2

ENSGALG00000014080	86,7875432	1,191385609	0,27640604	4,31027336	1,63E-05	0,00022917	
ENSGALG00000028785	165,798859	1,191015745	0,26472615	4,49904838	6,83E-06	0,0001062	
ENSGALG00000014864	693,502451	1,190480858	0,15988047	7,44606817	9,62E-14	4,59E-12	
ENSGALG00000012927	251,354318	1,187339616	0,23405659	5,07287416	3,92E-07	7,58E-06	
ENSGALG00000007809	1064,24338	1,186972522	0,17157276	6,91818756	4,57E-12	1,77E-10	TSHZ2
ENSGALG00000016455	38193,5361	1,186649639	0,12872943	9,2181693	3,02E-20	2,59E-18	GREB1
ENSGALG00000013149	115,338492	1,183860135	0,26574609	4,45485435	8,40E-06	0,00012698	MOCOS
ENSGALG00000012407	252,485775	1,181422763	0,24199801	4,8819524	1,05E-06	1,90E-05	LRP1B
ENSGALG00000004365	588,746185	1,179727636	0,2121902	5,55976489	2,70E-08	6,32E-07	WDR66
ENSGALG00000011867	1901,86124	1,179474298	0,13477658	8,75132991	2,11E-18	1,52E-16	SERHL2
ENSGALG00000008369	1041,31301	1,177660239	0,16764215	7,02484557	2,14E-12	8,79E-11	ABCC5
ENSGALG00000002294	2609,13169	1,174006641	0,10962142	10,7096464	9,17E-27	1,23E-24	SEMA6A
ENSGALG00000002543	4971,15753	1,169335598	0,11955874	9,78042788	1,37E-22	1,43E-20	CSGALNACT2
ENSGALG00000007608	2014,25657	1,169181539	0,16816744	6,95248465	3,59E-12	1,41E-10	ARHGEF9
ENSGALG00000013892	1213,76955	1,167370699	0,13409168	8,70576514	3,15E-18	2,24E-16	MAP3K5
ENSGALG00000011439	1116,38335	1,165074777	0,15459091	7,53650268	4,83E-14	2,41E-12	
ENSGALG00000009450	2113,57054	1,162765074	0,12246309	9,49482074	2,21E-21	2,10E-19	DUSP10
ENSGALG00000010778	367,923952	1,156146033	0,2139715	5,4032712	6,54E-08	1,43E-06	CHRM3
ENSGALG00000023768	2459,33149	1,155472868	0,1314993	8,78691237	1,54E-18	1,12E-16	
ENSGALG00000004083	1247,88665	1,153422459	0,18198777	6,33791205	2,33E-10	7,41E-09	GRIA1
ENSGALG00000016927	964,07015	1,145535192	0,18286396	6,26441204	3,74E-10	1,16E-08	
ENSGALG00000016258	101,512923	1,141614114	0,26933385	4,23865814	2,25E-05	0,0003031	
ENSGALG00000006988	211,344493	1,141001037	0,25510377	4,47269383	7,72E-06	0,00011783	
ENSGALG00000027554	57,7750225	1,140917568	0,26979126	4,22889002	2,35E-05	0,00031419	
ENSGALG00000010166	921,824931	1,138308624	0,1575227	7,22631474	4,96E-13	2,20E-11	VLDLR
ENSGALG00000022887	1108,58418	1,136537812	0,22465067	5,05913389	4,21E-07	8,11E-06	KBTBD11
ENSGALG00000009556	1809,38722	1,135549159	0,19219403	5,90834769	3,46E-09	9,02E-08	PRICKLE1
ENSGALG00000029090	11253,5016	1,13552965	0,10420101	10,8974918	1,18E-27	1,67E-25	
ENSGALG00000012998	2212,17969	1,132683468	0,17363381	6,52340397	6,87E-11	2,28E-09	WNT5B
ENSGALG00000015355	7944,79086	1,129448636	0,09943877	11,3582319	6,75E-30	1,04E-27	CD47
ENSGALG00000028046	6226,49016	1,129369719	0,09319237	12,1186929	8,41E-34	1,57E-31	INSIG2
ENSGALG00000011690	44,529172	1,125300184	0,27344667	4,11524556	3,87E-05	0,00049258	
ENSGALG00000014866	49930,561	1,123162841	0,1127082	9,96522725	2,16E-23	2,44E-21	
ENSGALG00000004974	170,654697	1,122849026	0,25433468	4,41484832	1,01E-05	0,00014999	PPARG
ENSGALG00000009567	4756,82772	1,119009905	0,16006243	6,99108393	2,73E-12	1,09E-10	TLL1
ENSGALG00000027255	39,1566631	1,118838646	0,26596115	4,20677466	2,59E-05	0,00034362	NRXN3
ENSGALG00000026970	924,805259	1,11847741	0,16590864	6,74152603	1,57E-11	5,65E-10	
ENSGALG00000027778	219,349797	1,117057663	0,24992741	4,46952849	7,84E-06	0,0001193	
ENSGALG00000006376	88,1016959	1,116147137	0,27664315	4,03460964	5,47E-05	0,00067008	TMC3
ENSGALG00000008469	3420,38421	1,115125474	0,13665113	8,16038231	3,34E-16	2,03E-14	SPINT1
ENSGALG00000014687	236,291624	1,113638024	0,23763257	4,68638633	2,78E-06	4,61E-05	EPHA1
ENSGALG00000017044	264,610201	1,113521859	0,21886213	5,08777759	3,62E-07	7,06E-06	TRPC4
ENSGALG00000010692	6211,67753	1,111701613	0,10700281	10,3894623	2,77E-25	3,50E-23	LRP8
ENSGALG00000016470	648,950225	1,110403246	0,25736453	4,3145154	1,60E-05	0,00022558	KCNS3
ENSGALG00000010081	1999,17595	1,110146926	0,15885433	6,98845877	2,78E-12	1,11E-10	SH3D19
ENSGALG00000006337	29,3646416	1,109144959	0,25231504	4,39587332	1,10E-05	0,00016236	
ENSGALG00000026763	669,49741	1,104647316	0,1839101	6,00645275	1,90E-09	5,15E-08	
ENSGALG00000008182	270,211279	1,102402002	0,23087313	4,77492558	1,80E-06	3,11E-05	CHAC2
ENSGALG00000009170	1968,00063	1,101783321	0,11424532	9,64401251	5,21E-22	5,21E-20	NCEH1
ENSGALG00000006797	779,712052	1,10056236	0,16329659	6,73965302	1,59E-11	5,71E-10	ADARB2
ENSGALG00000015980	339,410662	1,100313329	0,24202176	4,54634055	5,46E-06	8,63E-05	
ENSGALG00000012439	1157,51774	1,098736639	0,22006508	4,99278059	5,95E-07	1,12E-05	
ENSGALG00000026518	640,345593	1,097099741	0,17683365	6,20413453	5,50E-10	1,65E-08	RUNDC3A
ENSGALG00000010268	256,63927	1,095957762	0,25692897	4,265606	1,99E-05	0,00027314	
ENSGALG00000028582	726,208031	1,093959706	0,15924578	6,86963091	6,44E-12	2,45E-10	
ENSGALG00000026379	373,049265	1,093396741	0,20587222	5,31104558	1,09E-07	2,29E-06	
ENSGALG00000002714	19270,5199	1,089564948	0,1023794	10,6424241	1,89E-26	2,49E-24	TMEM132C
ENSGALG00000016875	1713,92149	1,086190922	0,14132808	7,685599	1,52E-14	8,00E-13	
ENSGALG00000001434	188,612497	1,08597113	0,2527644	4,29637688	1,74E-05	0,00024177	STOM
ENSGALG00000005691	64,1916299	1,085096229	0,27707745	3,91621988	8,99E-05	0,00105057	
ENSGALG00000005492	5397,76531	1,08468259	0,14476582	7,49267029	6,75E-14	3,28E-12	
ENSGALG00000007113	2485,94591	1,078758406	0,26642197	4,04905949	5,14E-05	0,00063816	

ENSGALG00000006822	6257,91272	1,077915026	0,25174121	4,28183776	1,85E-05	0,00025592	PCDH19
ENSGALG00000013060	290,382045	1,076232694	0,23892972	4,50439019	6,66E-06	0,00010395	ADCY2
ENSGALG00000023581	182,271241	1,073595233	0,25670804	4,18216447	2,89E-05	0,0003802	
ENSGALG00000006705	1115,46368	1,073202663	0,18895057	5,67980637	1,35E-08	3,29E-07	MYOF
ENSGALG00000007056	2832,90477	1,069890641	0,15949999	6,70777866	1,98E-11	7,06E-10	
ENSGALG00000014907	2488,06414	1,069885451	0,14008721	7,63728156	2,22E-14	1,15E-12	DCBLD1
ENSGALG00000006808	8235,96126	1,068278734	0,08180763	13,0584237	5,69E-39	1,34E-36	TSPAN6
ENSGALG00000022871	763,279385	1,067659168	0,16564303	6,44554255	1,15E-10	3,78E-09	
ENSGALG00000003011	132,477548	1,06001077	0,26925957	3,93676173	8,26E-05	0,00097279	TOM1L1
ENSGALG00000028708	5484,0424	1,054408367	0,12654693	8,33215293	7,94E-17	5,07E-15	
ENSGALG00000025773	389,005412	1,052013322	0,20058054	5,24484243	1,56E-07	3,20E-06	
ENSGALG00000001629	48,4307098	1,051770302	0,27008877	3,89416521	9,85E-05	0,00113395	
ENSGALG00000006958	461,662682	1,051053707	0,19372591	5,42546797	5,78E-08	1,28E-06	COL27A1
ENSGALG00000026534	317,277092	1,050613394	0,22283472	4,71476524	2,42E-06	4,08E-05	
ENSGALG00000001020	6102,64046	1,049473012	0,13282357	7,90125574	2,76E-15	1,54E-13	
ENSGALG00000008623	472,488323	1,04510086	0,23119186	4,52049152	6,17E-06	9,70E-05	TOR4A
ENSGALG00000028602	4340,00904	1,04212846	0,26450876	3,93986374	8,15E-05	0,00096378	
ENSGALG00000006310	433,388773	1,04012476	0,19660584	5,2904063	1,22E-07	2,55E-06	
ENSGALG00000010035	1735,92924	1,03790767	0,15536856	6,68029407	2,38E-11	8,43E-10	NR3C2
ENSGALG00000013021	8977,79318	1,037759509	0,12348858	8,40368799	4,33E-17	2,78E-15	
ENSGALG00000014815	1413,97594	1,036175342	0,13546391	7,64908786	2,02E-14	1,05E-12	
ENSGALG00000012594	522,648656	1,03128421	0,20236132	5,09625171	3,46E-07	6,79E-06	NTRK2
ENSGALG00000006472	535,008316	1,030705214	0,20247632	5,09049761	3,57E-07	6,97E-06	
ENSGALG00000017411	67,4262747	1,030664685	0,27683047	3,72308975	0,0001968	0,00206178	RXFP3
ENSGALG00000023355	60,0806602	1,029241933	0,27415306	3,75426024	0,00017385	0,00185084	TMEM154
ENSGALG00000011609	3308,43376	1,027774555	0,27692763	3,71134712	0,00020616	0,00214725	
ENSGALG00000009129	1054,989	1,026339944	0,17522926	5,85712656	4,71E-09	1,21E-07	DLX5
ENSGALG00000004393	1206,90024	1,024579421	0,20985019	4,88243272	1,05E-06	1,89E-05	
ENSGALG00000007646	1017,38466	1,023532832	0,27175014	3,76644827	0,00016559	0,00177191	
ENSGALG00000016841	8687,62321	1,021854986	0,1195056	8,55068686	1,22E-17	8,23E-16	
ENSGALG00000006252	11554,9175	1,021389981	0,14561324	7,01440332	2,31E-12	9,35E-11	LMO4
ENSGALG00000023430	94,0381071	1,019281603	0,25211693	4,04289225	5,28E-05	0,00065195	
ENSGALG00000008491	3560,03676	1,018906247	0,13531729	7,52975657	5,08E-14	2,52E-12	
ENSGALG00000008856	397,953846	1,015823816	0,23484575	4,32549368	1,52E-05	0,00021609	
ENSGALG00000006638	1878,73274	1,014405382	0,11908476	8,51834775	1,62E-17	1,07E-15	GRIK4
ENSGALG00000028294	3072,93213	1,012130398	0,11397222	8,88050124	6,66E-19	4,90E-17	VWA5B2
ENSGALG00000002532	98,8821618	1,010182876	0,27499589	3,67344716	0,0002393	0,00244759	GJA4
ENSGALG00000014961	54,1316457	1,009675076	0,26059966	3,87442974	0,00010687	0,00122202	
ENSGALG00000007069	496,352261	1,009530133	0,21308127	4,73777041	2,16E-06	3,67E-05	
ENSGALG00000006908	12363,8802	1,006453251	0,11636849	8,6488472	5,20E-18	3,64E-16	DLG1
ENSGALG00000015440	150,286833	1,001448719	0,25673749	3,90067193	9,59E-05	0,00110798	
ENSGALG00000001419	1796,40392	0,999950253	0,12013706	8,32341193	8,55E-17	5,43E-15	DAB2IP
ENSGALG00000015208	5846,95619	0,998078994	0,1312595	7,60386116	2,87E-14	1,46E-12	ROR2
ENSGALG00000011623	8193,27422	0,997864905	0,27138614	3,67691917	0,00023607	0,00241849	ADAMTS3
ENSGALG00000010588	53,1286773	0,996815581	0,26205985	3,80377066	0,00014251	0,00155339	
ENSGALG00000011963	622,99738	0,995736113	0,16724263	5,95384154	2,62E-09	6,94E-08	PRDM5
ENSGALG00000008918	750,133591	0,995278864	0,19014125	5,23441849	1,66E-07	3,37E-06	LAMP5
ENSGALG00000013738	4323,62842	0,994196056	0,10739219	9,2576201	2,09E-20	1,82E-18	OPTN
ENSGALG00000011137	93,3711188	0,990105855	0,27694617	3,57508407	0,00035012	0,00339345	
ENSGALG00000021812	73,5735967	0,986192682	0,27617874	3,57084933	0,00035583	0,00343178	
ENSGALG00000006717	1503,28109	0,985550396	0,24782699	3,97676777	6,99E-05	0,00083864	
ENSGALG00000003149	1610,9192	0,985359473	0,14766495	6,67294075	2,51E-11	8,83E-10	
ENSGALG00000004917	473,928512	0,985260253	0,2036331	4,83840905	1,31E-06	2,33E-05	
ENSGALG00000005990	258,942154	0,985024441	0,26677048	3,6924042	0,00022214	0,00229463	
ENSGALG00000005692	405,777143	0,983308202	0,24246612	4,05544582	5,00E-05	0,00062331	
ENSGALG00000020723	739,146919	0,981929794	0,17104516	5,74076334	9,43E-09	2,33E-07	
ENSGALG00000016537	9995,53899	0,979050265	0,13793185	7,09807238	1,27E-12	5,29E-11	SCML2
ENSGALG00000027497	260,124381	0,976598532	0,22923317	4,26028447	2,04E-05	0,00027881	
ENSGALG00000004930	264,560406	0,975284752	0,27532773	3,54226849	0,0003967	0,00376505	HRH1
ENSGALG00000016143	2862,24797	0,975263193	0,16498108	5,91136394	3,39E-09	8,88E-08	
ENSGALG00000010926	130,297678	0,975212806	0,27472538	3,54977322	0,00038556	0,00367048	
ENSGALG00000012808	5197,90929	0,972939427	0,12111181	8,03339831	9,48E-16	5,57E-14	CDYL

ENSGALG00000003739	38757,8816	0,97006222	0,0963384	10,0693205	7,55E-24	8,74E-22	
ENSGALG00000003939	112,384247	0,968631016	0,26679037	3,63068202	0,00028267	0,00282651	
ENSGALG00000007343	72,8603799	0,967183937	0,2759661	3,50472014	0,00045709	0,00421349	
ENSGALG00000016341	139,195007	0,965199253	0,25730295	3,75121721	0,00017598	0,00186552	
ENSGALG00000014343	785,569079	0,963080504	0,16453536	5,85333465	4,82E-09	1,24E-07	
ENSGALG00000002509	14144,0875	0,962423205	0,119871	8,0288245	9,84E-16	5,75E-14	
ENSGALG00000021879	34,5191458	0,950789217	0,2438749	3,89867607	9,67E-05	0,00111509	
ENSGALG00000006614	57,8438472	0,950175613	0,27642148	3,437416	0,00058729	0,00519918	
ENSGALG00000006786	1099,77465	0,950169855	0,14131213	6,72390847	1,77E-11	6,34E-10	
ENSGALG00000016843	6732,69779	0,946578268	0,12267336	7,71624989	1,20E-14	6,38E-13	COL4A2
ENSGALG00000027805	264,08193	0,945988714	0,26256163	3,60292061	0,00031466	0,00309685	
ENSGALG00000017135	1317,87874	0,945725278	0,15347839	6,16194417	7,19E-10	2,11E-08	CRYL1
ENSGALG00000011336	41,6337265	0,944781785	0,2663112	3,54766074	0,00038867	0,00369723	
ENSGALG00000012224	1861,1774	0,94448356	0,15084874	6,26112991	3,82E-10	1,18E-08	
ENSGALG00000008014	268,980372	0,943508119	0,22646821	4,16618353	3,10E-05	0,00040481	CEBPB
ENSGALG00000026892	80,5665735	0,943046494	0,2766565	3,40872711	0,00065267	0,00568143	
ENSGALG00000010707	23,462609	0,94082246	0,24072754	3,90824608	9,30E-05	0,0010798	GPR68
ENSGALG00000015008	34,4613342	0,940194042	0,26427134	3,55768451	0,00037414	0,00358079	ZNF366
ENSGALG00000001405	278,869771	0,939567412	0,21873168	4,29552511	1,74E-05	0,00024223	IRF6
ENSGALG00000010163	10137,7585	0,938977707	0,10980213	8,55154343	1,21E-17	8,21E-16	LGR5
ENSGALG00000004662	3197,84361	0,937959127	0,14071695	6,66557313	2,64E-11	9,24E-10	DISP3
ENSGALG00000010836	1075,45486	0,937907565	0,15481833	6,05811696	1,38E-09	3,83E-08	
ENSGALG00000008206	194,334171	0,934029761	0,24433133	3,82279978	0,00013194	0,00145547	TESC
ENSGALG00000002397	809,586463	0,927215999	0,22531285	4,11523792	3,87E-05	0,00049258	PSPH
ENSGALG00000010412	554,029025	0,925287079	0,19363821	4,77843223	1,77E-06	3,07E-05	WWTR1
ENSGALG00000026370	105,807662	0,924816521	0,26789368	3,45217742	0,00055608	0,00495797	
ENSGALG00000004519	193,098236	0,924449735	0,26575159	3,47862353	0,000504	0,00456847	TRPV2
ENSGALG00000007039	10074,0502	0,92399781	0,125208	7,37970282	1,59E-13	7,38E-12	
ENSGALG00000008778	5061,18074	0,923325024	0,13889594	6,6476025	2,98E-11	1,04E-09	SPRED2
ENSGALG00000010785	431,614767	0,922406238	0,22004896	4,19182269	2,77E-05	0,00036514	ACOT11
ENSGALG00000015164	3009,10926	0,920081875	0,10753234	8,55632742	1,17E-17	7,92E-16	
ENSGALG00000014855	3796,22691	0,916891226	0,15123034	6,06287898	1,34E-09	3,74E-08	
ENSGALG00000016443	4759,48054	0,912806646	0,13882964	6,57501249	4,86E-11	1,65E-09	HPCAL1
ENSGALG00000023193	891,994507	0,912803471	0,15632952	5,83897064	5,25E-09	1,34E-07	
ENSGALG00000007667	897,591606	0,910790115	0,15122323	6,02281894	1,71E-09	4,71E-08	GPR158
ENSGALG00000007416	138,697657	0,91055725	0,26535747	3,43143628	0,00060039	0,00530016	CD3E
ENSGALG00000012533	31364,4878	0,909053879	0,13047228	6,96741015	3,23E-12	1,27E-10	
ENSGALG00000011618	1026,95651	0,908730543	0,18188258	4,99624825	5,85E-07	1,10E-05	ADSSL1
ENSGALG00000015624	28928,123	0,906952704	0,26927951	3,36807168	0,00075696	0,00646335	VCAN
ENSGALG00000001645	772,05714	0,906502233	0,18722125	4,84187682	1,29E-06	2,30E-05	KCNT1
ENSGALG00000011258	41539,9101	0,899386948	0,13199364	6,81386582	9,50E-12	3,51E-10	
ENSGALG00000003401	97,8434771	0,897409124	0,27523071	3,26057042	0,00111188	0,00890653	FBXL22
ENSGALG00000017652	38,6817197	0,894447523	0,26978	3,31547007	0,00091489	0,00757906	TTC9
ENSGALG00000004189	665,542257	0,89261736	0,19559277	4,56365205	5,03E-06	8,01E-05	MAFK
ENSGALG00000025723	106,160903	0,89142204	0,27513226	3,23997649	0,0011954	0,00944015	
ENSGALG00000016308	4475,13284	0,888326042	0,09104509	9,75698981	1,72E-22	1,77E-20	MYC
ENSGALG00000007952	16328,0045	0,887725324	0,11490596	7,72566813	1,11E-14	5,95E-13	AMOT
ENSGALG00000001281	97,6098799	0,886212495	0,27180262	3,26050025	0,00111216	0,00890653	
ENSGALG00000012111	193,797146	0,886059032	0,25134734	3,52523742	0,0004231	0,00396449	TMEM37
ENSGALG00000010148	95,0993319	0,885936489	0,27329578	3,24167639	0,00118829	0,00940681	
ENSGALG00000001276	18245,4455	0,885240877	0,11268934	7,8555867	3,98E-15	2,20E-13	HOXB9
ENSGALG00000015896	1982,50233	0,883690573	0,14767564	5,98399687	2,18E-09	5,84E-08	
ENSGALG00000010090	526,624476	0,882987641	0,22809712	3,87110386	0,00010834	0,0012348	FAM160A1
ENSGALG00000009594	606,263065	0,882488341	0,19693947	4,48101319	7,43E-06	0,00011388	RARRES1
ENSGALG00000005256	9190,22784	0,880582454	0,23610272	3,72965825	0,00019174	0,00201889	EPHA4
ENSGALG00000009179	4215,94774	0,880187457	0,10683081	8,23907883	1,74E-16	1,08E-14	TNFSF10
ENSGALG00000011172	2449,32108	0,879786034	0,13379316	6,57571768	4,84E-11	1,65E-09	
ENSGALG00000027947	77,7692928	0,877628202	0,26244862	3,34400006	0,0008258	0,00697665	GHSR
ENSGALG00000027885	26,4620215	0,876800098	0,23993489	3,65432511	0,00025786	0,00260968	C4orf19
ENSGALG00000014783	8965,42611	0,875888949	0,11142269	7,86095696	3,81E-15	2,12E-13	
ENSGALG00000009405	358,011724	0,875608773	0,20294284	4,31455849	1,60E-05	0,00022558	GRIA2
ENSGALG00000010996	3029,60967	0,875351394	0,11601158	7,54537966	4,51E-14	2,26E-12	IRF2BP2

ENSGALG00000028759	391,741267	0,87268698	0,23549591	3,70574156	0,00021077	0,00219166	LYPD6B
ENSGALG00000008018	469,138933	0,868526004	0,20965704	4,14260361	3,43E-05	0,00044328	SNAI1
ENSGALG00000006008	593,4561	0,867963452	0,20178146	4,30150244	1,70E-05	0,0002371	HOMER2
ENSGALG00000029023	178,138305	0,865902724	0,26156953	3,3104113	0,00093159	0,00768178	DMRTB1
ENSGALG00000016909	3574,55237	0,864393738	0,09752748	8,86307897	7,78E-19	5,69E-17	NDFIP2
ENSGALG00000026582	1114,9679	0,863598751	0,1902411	4,53949614	5,64E-06	8,89E-05	
ENSGALG00000027684	3428,7366	0,863457403	0,14910293	5,79101571	7,00E-09	1,75E-07	
ENSGALG00000014349	1819,66371	0,860574566	0,11955806	7,19796341	6,11E-13	2,65E-11	
ENSGALG00000011613	475,600449	0,857977883	0,20239708	4,23908233	2,24E-05	0,0003031	
ENSGALG00000001617	271,795228	0,855845667	0,23372685	3,6617345	0,00025051	0,0025415	CAMKK1
ENSGALG00000001926	1199,81634	0,853283247	0,15483373	5,51096475	3,57E-08	8,22E-07	HSPB1
ENSGALG00000015180	12680,3183	0,851097646	0,10970107	7,75833511	8,61E-15	4,64E-13	PSAT1
ENSGALG00000009396	1375,97204	0,850250642	0,16438706	5,17224786	2,31E-07	4,59E-06	
ENSGALG00000009172	460,012151	0,849588562	0,22136087	3,83802504	0,00012403	0,001384	OSBPL6
ENSGALG00000016807	8544,75416	0,848101313	0,11909181	7,12140736	1,07E-12	4,55E-11	
ENSGALG00000010291	5393,74102	0,844497871	0,10367256	8,14581883	3,77E-16	2,28E-14	RBPMS
ENSGALG00000000977	7029,01558	0,842188766	0,08563672	9,83443558	8,00E-23	8,48E-21	MAPKAP1
ENSGALG00000009530	719,235997	0,841159622	0,18836636	4,46555117	7,99E-06	0,0001212	PDZRN4
ENSGALG00000004155	211,984568	0,841077447	0,24078857	3,49301238	0,0004776	0,00436403	MYOM3
ENSGALG00000009378	1120,38163	0,840979613	0,13880777	6,05859175	1,37E-09	3,83E-08	PDGFC
ENSGALG00000002335	624,130373	0,840686726	0,18168554	4,62715265	3,71E-06	6,03E-05	DOCK3
ENSGALG00000002623	461,663405	0,839503746	0,18687975	4,49221356	7,05E-06	0,00010872	PCOLCE2
ENSGALG00000005410	3123,4698	0,838361241	0,15163748	5,52872037	3,23E-08	7,46E-07	
ENSGALG00000007932	1262,37356	0,837645303	0,16803809	4,98485361	6,20E-07	1,16E-05	TRAF6
ENSGALG00000015409	14646,8005	0,837042297	0,10986863	7,61857419	2,56E-14	1,32E-12	PROS1
ENSGALG00000012278	12900,8817	0,835859144	0,23517071	3,55426545	0,00037904	0,00361936	
ENSGALG00000010156	256,288262	0,833499217	0,2396958	3,47732084	0,00050645	0,0045874	
ENSGALG00000001220	3532,90233	0,831042878	0,11299613	7,35461342	1,91E-13	8,81E-12	HERPUD1
ENSGALG00000013085	2051,84727	0,830893947	0,12691125	6,54704716	5,87E-11	1,98E-09	PTPRO
ENSGALG00000025770	327,819551	0,830841194	0,24237973	3,42784926	0,00060838	0,00535557	
ENSGALG00000012354	2002,91372	0,829092837	0,15052249	5,50809953	3,63E-08	8,31E-07	
ENSGALG00000000639	543,002187	0,827648848	0,21824285	3,79232975	0,00014924	0,00161912	LRRN2
ENSGALG00000005768	4764,60581	0,825923962	0,11172719	7,39232759	1,44E-13	6,76E-12	ETV5
ENSGALG00000011232	2354,98602	0,825691237	0,13504902	6,11401152	9,72E-10	2,77E-08	
ENSGALG00000017246	1270,40827	0,825125037	0,15896962	5,19045723	2,10E-07	4,19E-06	ME3
ENSGALG00000008883	4099,49868	0,82333394	0,11713657	7,02883754	2,08E-12	8,57E-11	TCF7L2
ENSGALG00000020899	521,609979	0,823141433	0,24736905	3,3275846	0,00087602	0,00731033	
ENSGALG00000003726	904,249522	0,818923475	0,22585036	3,625956	0,00028789	0,00286954	EGFLAM
ENSGALG00000010664	324,510001	0,817672769	0,23994525	3,40774723	0,00065502	0,00569168	KIF26B
ENSGALG00000002054	1352,81303	0,816030183	0,13128078	6,21591508	5,10E-10	1,54E-08	
ENSGALG00000008670	1990,92325	0,815447938	0,13744115	5,9330699	2,97E-09	7,83E-08	
ENSGALG00000013683	16575,6485	0,810200928	0,12018138	6,74148471	1,57E-11	5,65E-10	ARID1B
ENSGALG00000012848	3319,46891	0,809997189	0,15987767	5,06635591	4,06E-07	7,83E-06	
ENSGALG00000014872	561,414638	0,806209646	0,23662254	3,40715487	0,00065644	0,0056984	FGF10
ENSGALG00000009155	822,192391	0,800986669	0,16316786	4,90897338	9,16E-07	1,68E-05	
ENSGALG00000005430	836,618226	0,79963845	0,20651888	3,87198711	0,00010795	0,00123209	ALS2CL
ENSGALG00000012849	4391,56992	0,79670503	0,13188371	6,04096596	1,53E-09	4,24E-08	SLC37A3
ENSGALG00000012277	3279,13345	0,79518636	0,11025611	7,21217515	5,51E-13	2,42E-11	
ENSGALG00000015952	320,695046	0,793177929	0,23917365	3,31632662	0,00091209	0,00756087	
ENSGALG00000004599	1177,35629	0,791092143	0,17593216	4,49657499	6,91E-06	0,00010704	
ENSGALG00000026631	639,083895	0,790472215	0,23279997	3,39549971	0,00068503	0,00592197	HOXA10
ENSGALG00000002443	515,246567	0,789654091	0,18384496	4,29521754	1,75E-05	0,0002423	FBXL16
ENSGALG00000008445	716,590826	0,788973502	0,17118936	4,60877658	4,05E-06	6,52E-05	SLC24A3
ENSGALG00000009718	365,400186	0,788380099	0,1998647	3,94456899	7,99E-05	0,00094788	FAM53B
ENSGALG00000009252	6799,64958	0,7810557	0,11275794	6,92683575	4,30E-12	1,68E-10	
ENSGALG00000026034	890,686008	0,780909637	0,16319128	4,7852411	1,71E-06	2,98E-05	RGS17
ENSGALG00000002598	7375,09654	0,778822215	0,08757756	8,89294263	5,95E-19	4,46E-17	TPST1
ENSGALG00000005310	792,651331	0,773499973	0,20804946	3,71786588	0,00020091	0,0020996	
ENSGALG00000021238	292,286817	0,771321549	0,21541142	3,58069015	0,00034269	0,00333594	
ENSGALG00000026355	316,135871	0,769808548	0,22820508	3,37331911	0,00074268	0,00636312	
ENSGALG00000004158	2408,26029	0,769428918	0,12659815	6,07772646	1,22E-09	3,45E-08	
ENSGALG00000001346	433,934694	0,76776324	0,23273948	3,29880968	0,00097096	0,00794876	

ENSGALG00000013124	3883,6626	0,765332021	0,13324393	5,74384156	9,26E-09	2,29E-07	FHOD3
ENSGALG00000025780	468,175583	0,762758431	0,19965686	3,82034666	0,00013326	0,00146485	ITPRIP
ENSGALG00000023886	345,558396	0,76245624	0,20569517	3,70672893	0,00020995	0,00218495	ZNF804A
ENSGALG00000011376	374,497295	0,758366172	0,20404377	3,71668381	0,00020185	0,00210768	ANKRD9
ENSGALG00000016069	4206,1542	0,758301426	0,09589383	7,90771867	2,62E-15	1,47E-13	
ENSGALG00000001000	105882,406	0,756534693	0,12130242	6,23676501	4,47E-10	1,36E-08	
ENSGALG00000013616	416,825405	0,749712043	0,2111769	3,55016123	0,000385	0,00366786	OPRM1
ENSGALG00000009145	1069,11169	0,74529577	0,2190544	3,40233194	0,00066813	0,00578387	
ENSGALG00000012012	5118,87574	0,743046962	0,14577197	5,09732411	3,44E-07	6,76E-06	
ENSGALG00000011319	11225,2963	0,737544915	0,09161073	8,05085711	8,22E-16	4,85E-14	ADGRL3
ENSGALG00000005512	7764,5793	0,736922168	0,10571311	6,97096281	3,15E-12	1,25E-10	
ENSGALG00000000505	339,344776	0,73643955	0,20375113	3,61440716	0,00030104	0,00298625	KCNH6
ENSGALG00000006138	2327,25759	0,736205494	0,17788661	4,13862223	3,49E-05	0,00045058	
ENSGALG00000003701	2093,50715	0,734267222	0,12693056	5,78479482	7,26E-09	1,81E-07	SGMS1
ENSGALG00000003043	2889,49683	0,734167184	0,12468763	5,88805143	3,91E-09	1,02E-07	
ENSGALG00000015250	1236,64137	0,732130121	0,18294116	4,00199787	6,28E-05	0,00075985	
ENSGALG00000028155	1607,60298	0,730708288	0,1348053	5,42047159	5,94E-08	1,31E-06	
ENSGALG00000003774	6718,46982	0,73043073	0,11984382	6,09485535	1,10E-09	3,11E-08	ATP13A2
ENSGALG00000005653	2602,66741	0,729875771	0,10427934	6,99923686	2,57E-12	1,03E-10	NFKB2
ENSGALG00000007501	443,889264	0,729453053	0,20909338	3,48864733	0,00048547	0,00442944	
ENSGALG00000025887	833,811929	0,729103686	0,19083714	3,8205545	0,00013315	0,00146485	ATF3
ENSGALG00000015184	6562,38853	0,727266294	0,09772755	7,44177369	9,93E-14	4,72E-12	
ENSGALG00000002955	1085,67322	0,725828311	0,20628485	3,51857296	0,00043387	0,00404688	ADAMTSL2
ENSGALG00000028935	4407,81779	0,725128037	0,09141746	7,9320517	2,16E-15	1,22E-13	BCL10
ENSGALG00000009381	14937,4753	0,723727203	0,11606588	6,23548619	4,50E-10	1,37E-08	
ENSGALG00000014441	1267,12755	0,722905404	0,15192936	4,75816803	1,95E-06	3,35E-05	
ENSGALG00000027352	11317,2552	0,722863534	0,10576918	6,83434951	8,24E-12	3,08E-10	
ENSGALG00000001370	1171,81779	0,722550699	0,15690532	4,60501077	4,12E-06	6,62E-05	ADAMTS8
ENSGALG00000007206	17,4582489	0,720355559	0,22314617	3,22817802	0,00124581	0,00980119	
ENSGALG00000015947	765,779345	0,718924866	0,22274381	3,22758633	0,00124839	0,00980672	
ENSGALG00000008163	14921,5324	0,713636421	0,10768275	6,62721234	3,42E-11	1,18E-09	PSME4
ENSGALG00000026727	425,717003	0,713627256	0,19018155	3,75234749	0,00017519	0,00186028	SLC30A10
ENSGALG00000028279	322,93543	0,711492754	0,21583535	3,29646077	0,00097911	0,00800505	SNORD14
ENSGALG00000007712	5151,87784	0,709640176	0,09468723	7,49457113	6,65E-14	3,25E-12	MYO1B
ENSGALG00000005423	1960,17378	0,707902433	0,13324908	5,31262534	1,08E-07	2,28E-06	GRHPR
ENSGALG00000015975	5061,20264	0,705892008	0,11070199	6,3765067	1,81E-10	5,84E-09	
ENSGALG00000002892	626,326751	0,704967282	0,1963291	3,59074262	0,00032974	0,00322241	
ENSGALG00000015310	3979,38646	0,701805821	0,09924244	7,07163008	1,53E-12	6,34E-11	
ENSGALG00000000452	1371,04529	0,701787311	0,16834665	4,16870367	3,06E-05	0,00040083	
ENSGALG00000002198	1124,31872	0,701337674	0,20146263	3,4812296	0,00049912	0,00452753	FHOD1
ENSGALG00000010522	10232,0506	0,699893806	0,1480305	4,72803795	2,27E-06	3,84E-05	
ENSGALG00000027983	1055,55115	0,699268766	0,16725899	4,18075434	2,91E-05	0,00038217	TNK2
ENSGALG00000010204	874,20571	0,699072042	0,16632037	4,20316555	2,63E-05	0,00034841	KCNC2
ENSGALG00000004088	3580,2986	0,695518825	0,10391048	6,69344229	2,18E-11	7,75E-10	FNBP1
ENSGALG00000008680	626,282132	0,694380448	0,18287186	3,79708749	0,00014641	0,00159251	
ENSGALG00000011266	760,864805	0,693047043	0,18382819	3,77008024	0,0001632	0,00174928	PPM1K
ENSGALG00000015721	1214,39078	0,692751518	0,18278823	3,78991313	0,0001507	0,00163213	SVEP1
ENSGALG00000012956	536,621695	0,692712504	0,21091071	3,28438757	0,00102204	0,00828563	TMTC1
ENSGALG00000011025	4159,539	0,69039215	0,09483213	7,28015057	3,33E-13	1,50E-11	
ENSGALG00000012211	4094,58636	0,688791118	0,10047528	6,85532894	7,11E-12	2,69E-10	PDLIM5
ENSGALG00000005065	974,960396	0,685705936	0,15970851	4,29348406	1,76E-05	0,00024393	PLA2G4A
ENSGALG00000016835	5266,33357	0,685225343	0,13174668	5,20108259	1,98E-07	3,97E-06	
ENSGALG00000012696	8253,81984	0,682603698	0,09998162	6,82729216	8,65E-12	3,21E-10	
ENSGALG00000016281	10145,7509	0,681257671	0,08559267	7,95929876	1,73E-15	9,93E-14	DMD
ENSGALG00000023725	1053,58774	0,679129782	0,15467581	4,39066583	1,13E-05	0,00016591	
ENSGALG00000003640	865,821934	0,678918743	0,16063195	4,22654863	2,37E-05	0,00031683	
ENSGALG00000027627	7249,84284	0,677661132	0,0846528	8,00518259	1,19E-15	6,94E-14	
ENSGALG00000027342	11212,2371	0,672830693	0,07880349	8,5380821	1,36E-17	9,08E-16	
ENSGALG00000011550	2837,22447	0,672556317	0,14266182	4,71433983	2,42E-06	4,08E-05	
ENSGALG00000027389	3615,54123	0,672548469	0,11214472	5,99714785	2,01E-09	5,41E-08	RGS7BP
ENSGALG00000002646	9732,02947	0,671803119	0,1449266	4,63547136	3,56E-06	5,80E-05	FAM171B
ENSGALG00000004612	5923,23561	0,671680213	0,09027919	7,44003379	1,01E-13	4,77E-12	MTHFR

ENSGALG00000014106	16074,824	0,667075995	0,09963033	6,69551116	2,15E-11	7,66E-10	PPFIBP1
ENSGALG00000013342	2747,02869	0,666913547	0,1119687	5,95624957	2,58E-09	6,85E-08	GBX2
ENSGALG00000007704	16606,5878	0,666805577	0,12692892	5,25337789	1,49E-07	3,07E-06	
ENSGALG00000023876	3391,40413	0,666027019	0,11746061	5,67021584	1,43E-08	3,46E-07	
ENSGALG00000015887	11962,9806	0,665885713	0,11836362	5,62576338	1,85E-08	4,42E-07	URB1
ENSGALG00000003957	1955,41186	0,665113393	0,12648287	5,25852528	1,45E-07	2,99E-06	
ENSGALG00000006872	8598,0072	0,664035354	0,09325855	7,12036955	1,08E-12	4,56E-11	PISD
ENSGALG00000004437	1051,52128	0,662144004	0,15643348	4,23275119	2,31E-05	0,00031017	LHFPL2
ENSGALG00000014957	23554,8466	0,661960443	0,09128513	7,25156958	4,12E-13	1,84E-11	WDR1
ENSGALG00000002655	5806,05662	0,657486198	0,10761985	6,10933924	1,00E-09	2,84E-08	ITGAV
ENSGALG00000004661	7333,89219	0,654997852	0,09584164	6,83416796	8,25E-12	3,08E-10	
ENSGALG00000004231	624,271591	0,654722209	0,17330476	3,77786627	0,00015818	0,00170573	IFNLR1
ENSGALG00000011770	13005,5067	0,65450966	0,08870671	7,37835601	1,60E-13	7,42E-12	UMPS
ENSGALG00000016803	4124,86158	0,652622211	0,11026512	5,91866387	3,25E-09	8,51E-08	ST6GAL2
ENSGALG00000016719	6369,91266	0,652460747	0,11253545	5,79782427	6,72E-09	1,69E-07	TNFRSF21
ENSGALG00000010186	4216,82392	0,652306301	0,0936837	6,96285777	3,33E-12	1,31E-10	
ENSGALG00000014545	998,185181	0,650360352	0,17599459	3,69534294	0,00021959	0,00227389	
ENSGALG00000005906	1908,90153	0,646437734	0,11565287	5,5894654	2,28E-08	5,39E-07	
ENSGALG00000004042	4037,76912	0,646319325	0,11291506	5,72394258	1,04E-08	2,56E-07	
ENSGALG00000018479	474,539705	0,645095331	0,19673783	3,27895931	0,00104191	0,00843028	
ENSGALG00000012877	2598,2138	0,643827392	0,1203305	5,35049194	8,77E-08	1,87E-06	CREB3L2
ENSGALG00000013031	981,078598	0,641018743	0,1528676	4,19329383	2,75E-05	0,00036355	CECR1
ENSGALG00000027835	4356,75822	0,637590923	0,09597243	6,64348014	3,06E-11	1,06E-09	PCBD1
ENSGALG00000024346	783,051886	0,637233355	0,1537054	4,14580971	3,39E-05	0,00043848	
ENSGALG00000002248	5849,29235	0,635943683	0,08808525	7,21963901	5,21E-13	2,30E-11	
ENSGALG00000026776	1287,36825	0,634230033	0,13457396	4,71287338	2,44E-06	4,10E-05	TIGAR
ENSGALG00000008318	17655,4148	0,633974687	0,08385645	7,56023739	4,02E-14	2,02E-12	SLK
ENSGALG00000015842	5393,93615	0,632020307	0,09677563	6,53077971	6,54E-11	2,19E-09	TIAM1
ENSGALG00000004181	1516,86359	0,628709896	0,12659877	4,96616114	6,83E-07	1,27E-05	
ENSGALG00000014922	13357,9815	0,627329029	0,08060516	7,7827402	7,10E-15	3,88E-13	ROCK1
ENSGALG00000002602	2698,72514	0,625832331	0,15063415	4,15465119	3,26E-05	0,00042362	
ENSGALG00000012726	94562,7089	0,625221924	0,1022285	6,11592611	9,60E-10	2,75E-08	
ENSGALG00000027696	2435,05731	0,622663255	0,13427256	4,6373083	3,53E-06	5,76E-05	
ENSGALG00000008222	3620,89787	0,616961764	0,10986009	5,61588627	1,96E-08	4,66E-07	SLC26A5
ENSGALG00000011435	5310,48391	0,614694006	0,09295677	6,61268659	3,77E-11	1,29E-09	
ENSGALG00000001728	1163,49973	0,610499448	0,14119355	4,32384786	1,53E-05	0,00021746	NACC2
ENSGALG00000026093	847,433551	0,608505556	0,17097819	3,55896599	0,00037232	0,0035661	
ENSGALG00000019397	12768,6069	0,608262914	0,09652905	6,30134598	2,95E-10	9,30E-09	CERK
ENSGALG00000004820	960,081767	0,608049873	0,1426222	4,26336072	2,01E-05	0,0002756	FAM57A
ENSGALG00000026468	6537,04521	0,607177306	0,09886931	6,14121105	8,19E-10	2,38E-08	CHSY1
ENSGALG00000000795	1331,44868	0,605895026	0,13162727	4,60311177	4,16E-06	6,68E-05	MFSD12
ENSGALG00000017199	2734,428	0,605852032	0,15883889	3,81425504	0,00013659	0,00149751	MAML2
ENSGALG00000008903	2249,72466	0,604190258	0,15751307	3,83581031	0,00012515	0,00139302	SSFA2
ENSGALG00000011537	2507,94825	0,603878853	0,15538039	3,88645484	0,00010172	0,00116734	PDE10A
ENSGALG00000006939	25023,5764	0,603844144	0,10825219	5,57812385	2,43E-08	5,73E-07	PFKP
ENSGALG00000007434	2113,20997	0,599865863	0,15496377	3,87100707	0,00010839	0,0012348	RHBDF1
ENSGALG00000011488	1168,68478	0,598872747	0,17053454	3,51173862	0,00044519	0,00412506	CMTM7
ENSGALG00000016834	1257,10709	0,598292773	0,13769793	4,34496565	1,39E-05	0,00019982	MCF2L
ENSGALG00000009988	2544,44093	0,593615724	0,112456	5,27864872	1,30E-07	2,70E-06	ELOVL1
ENSGALG00000011548	2906,3811	0,593504022	0,11422522	5,19591069	2,04E-07	4,08E-06	PPP1R13B
ENSGALG00000009755	3320,85237	0,592776412	0,10287504	5,76210162	8,31E-09	2,06E-07	LRIG3
ENSGALG00000002709	2623,11643	0,59270728	0,12665173	4,67981968	2,87E-06	4,75E-05	MYO1C
ENSGALG00000003642	1312,51148	0,590129639	0,16880923	3,49583753	0,00047258	0,0043244	PDGF-A
ENSGALG00000008615	1132,69776	0,588449543	0,17235789	3,41411434	0,0006399	0,00559753	
ENSGALG00000015096	745,308008	0,586609595	0,15533516	3,77641219	0,0001591	0,00171424	SH3GL2
ENSGALG00000011295	1419,82516	0,585889038	0,13140148	4,45877045	8,24E-06	0,00012483	SOCS2
ENSGALG00000014933	1201,23972	0,584450113	0,16167735	3,61491648	0,00030044	0,00298275	HEXB
ENSGALG00000012979	9491,84596	0,583725131	0,11596876	5,0334689	4,82E-07	9,19E-06	
ENSGALG00000001076	3535,03862	0,580936414	0,09643753	6,02396613	1,70E-09	4,70E-08	LIMK1
ENSGALG00000007242	1537,31241	0,576491214	0,1265805	4,55434468	5,25E-06	8,33E-05	SLITRK4
ENSGALG00000011045	4784,96919	0,574987094	0,09168796	6,27113007	3,58E-10	1,12E-08	
ENSGALG00000020479	3396,9575	0,574556644	0,12483475	4,60253786	4,17E-06	6,68E-05	

ENSGALG00000016276	897,324858	0,574168384	0,14748691	3,89301259	9,90E-05	0,00113831	KHDRBS2
ENSGALG00000012429	2058,77028	0,572869136	0,16484364	3,47522751	0,00051042	0,00461848	BMP4
ENSGALG00000019961	8549,80541	0,57282511	0,10454384	5,47928114	4,27E-08	9,70E-07	SLC18B1
ENSGALG00000009415	20919,8487	0,570748764	0,13417273	4,25383594	2,10E-05	0,00028634	SMOC1
ENSGALG00000011241	1131,08019	0,569997915	0,15948275	3,57404125	0,00035151	0,00340331	RFTN1
ENSGALG00000011708	16164,7225	0,569570289	0,12123193	4,69818723	2,62E-06	4,38E-05	MYLK
ENSGALG00000006340	2676,99994	0,569237942	0,12657684	4,49717295	6,89E-06	0,00010687	
ENSGALG00000009156	4770,93246	0,56553435	0,14428903	3,91945491	8,87E-05	0,00103754	CDAN1
ENSGALG00000012394	13820,7507	0,564570466	0,07862384	7,1806525	6,94E-13	3,00E-11	NID2
ENSGALG00000016350	2492,97331	0,563918458	0,11007286	5,12313797	3,00E-07	5,92E-06	ARHGEF10
ENSGALG00000008293	782,926286	0,562981441	0,15976711	3,52376315	0,00042546	0,00397768	SH3PXD2A
ENSGALG00000006655	826,977917	0,562031156	0,15875615	3,54021654	0,0003998	0,00379131	MAP3K13
ENSGALG00000001664	2551,39665	0,561926589	0,13283895	4,23013411	2,34E-05	0,00031279	PTPN9
ENSGALG00000008621	9550,43432	0,561672993	0,11796857	4,76120892	1,92E-06	3,30E-05	NRP2
ENSGALG00000003122	1351,29404	0,561038511	0,1311515	4,27778952	1,89E-05	0,00026004	ZFAND2A
ENSGALG00000015858	3586,9625	0,559546334	0,10146313	5,51477519	3,49E-08	8,06E-07	DOPEY1
ENSGALG00000013768	1494,29229	0,55942652	0,16037363	3,48827007	0,00048616	0,00442998	PHACTR2
ENSGALG00000015862	11794,0676	0,559415098	0,12796473	4,37163513	1,23E-05	0,00017979	
ENSGALG00000027757	11293,5634	0,559044043	0,10878229	5,13910919	2,76E-07	5,46E-06	ARL4A
ENSGALG00000017462	3715,9454	0,558530219	0,09687426	5,76551735	8,14E-09	2,02E-07	FNIP1
ENSGALG00000000388	11929,068	0,558245728	0,14596937	3,82440333	0,00013109	0,00144858	LIN28A
ENSGALG00000007238	2073,56415	0,558183051	0,16989919	3,28537799	0,00101846	0,00826191	
ENSGALG00000012852	29056,4718	0,557720187	0,10036391	5,55697956	2,74E-08	6,41E-07	MKRN1
ENSGALG00000001163	1519,07571	0,557286192	0,12356604	4,51002717	6,48E-06	0,00010148	NDEL1
ENSGALG00000016480	9465,84299	0,557041155	0,0924977	6,02221656	1,72E-09	4,71E-08	SDC1
ENSGALG00000028291	14773,7601	0,555590355	0,1099194	5,05452513	4,31E-07	8,29E-06	
ENSGALG00000007845	1987,98275	0,553575037	0,11370336	4,86859011	1,12E-06	2,02E-05	REL
ENSGALG00000014084	5460,83517	0,552765103	0,13748262	4,02061798	5,80E-05	0,00070908	FGFR1OP2
ENSGALG00000009433	8016,67263	0,551628222	0,14479495	3,80972014	0,00013912	0,00152391	BAG3
ENSGALG00000000680	7215,7939	0,550999971	0,1233941	4,46536719	7,99E-06	0,0001212	
ENSGALG00000005433	1687,94239	0,550736363	0,11950908	4,60832215	4,06E-06	6,53E-05	
ENSGALG00000002461	9112,46886	0,549280944	0,12891627	4,2607573	2,04E-05	0,00027853	
ENSGALG00000007394	992,390556	0,546419843	0,14202976	3,84722077	0,00011947	0,00134267	
ENSGALG00000015215	5874,51018	0,543882262	0,10412914	5,22315125	1,76E-07	3,55E-06	MAPRE2
ENSGALG00000010164	6967,47145	0,542805965	0,08759539	6,19674117	5,76E-10	1,73E-08	SMARCA2
ENSGALG00000017365	2743,73739	0,541410874	0,12416381	4,36045628	1,30E-05	0,00018835	
ENSGALG00000014860	10032,8437	0,540418516	0,08281121	6,52591008	6,76E-11	2,25E-09	YES1
ENSGALG00000016781	15938,709	0,539823119	0,08390658	6,43362076	1,25E-10	4,08E-09	MAP4K4
ENSGALG00000016883	2972,3538	0,537559856	0,10976916	4,89718485	9,72E-07	1,77E-05	DOCK9
ENSGALG00000011449	1797,12078	0,537091274	0,13239866	4,05662179	4,98E-05	0,00062155	
ENSGALG00000009855	6709,81331	0,536813312	0,11427025	4,69775213	2,63E-06	4,38E-05	TBC1D9
ENSGALG00000017185	9572,81105	0,536156218	0,13194516	4,06347779	4,83E-05	0,00060477	TMEM123
ENSGALG00000013406	658,679579	0,533541915	0,16232604	3,28685351	0,00101314	0,00822408	EOGT
ENSGALG00000026237	8325,49172	0,532296374	0,09561992	5,56679355	2,59E-08	6,08E-07	
ENSGALG00000000669	4577,06628	0,531970586	0,10145026	5,24365914	1,57E-07	3,21E-06	ETV3
ENSGALG00000015792	1821,33594	0,531949092	0,12531887	4,24476438	2,19E-05	0,00029688	
ENSGALG00000000891	3401,91556	0,531658469	0,10937715	4,86078185	1,17E-06	2,10E-05	NSUN5
ENSGALG00000015112	8927,72556	0,528998124	0,10528495	5,02444216	5,05E-07	9,60E-06	
ENSGALG00000002471	3937,07792	0,528654759	0,11018735	4,79778103	1,60E-06	2,82E-05	
ENSGALG00000014834	2740,7337	0,527383014	0,1070106	4,92832478	8,29E-07	1,53E-05	NCOA7
ENSGALG00000027526	2310,64445	0,527340701	0,12420628	4,24568455	2,18E-05	0,00029599	COL9A2
ENSGALG00000017414	7193,90052	0,522128726	0,09330101	5,59617465	2,19E-08	5,19E-07	JUP
ENSGALG00000002759	2898,01396	0,520324255	0,1531807	3,39680035	0,00068179	0,00589798	PEAK1
ENSGALG00000015193	2485,09904	0,518302858	0,13474249	3,84661781	0,00011976	0,00134477	CCDC80
ENSGALG00000002583	881,320118	0,51810067	0,15491432	3,34443367	0,00082451	0,00697347	PIK3CD
ENSGALG00000006002	2311,21963	0,517539205	0,12381422	4,1799657	2,92E-05	0,00038309	WHAMM
ENSGALG00000011162	1743,64653	0,516837911	0,12166248	4,24812899	2,16E-05	0,00029341	CYP46A1
ENSGALG00000000761	2548,35828	0,511636934	0,11935769	4,28658551	1,81E-05	0,00025079	TSKU
ENSGALG00000008811	6319,41069	0,510104856	0,14038413	3,63363628	0,00027945	0,00279777	SLC1A4
ENSGALG00000007016	944,373297	0,509222424	0,14442833	3,52577944	0,00042224	0,00395936	FAM169B
ENSGALG00000003668	1606,5427	0,508101808	0,12448719	4,08155899	4,47E-05	0,00056127	
ENSGALG00000003948	4679,11028	0,503499536	0,09252751	5,44161999	5,28E-08	1,19E-06	ALAS1

ENSGALG00000015458	8275,30923	0,50234495	0,11154476	4,50352792	6,68E-06	0,00010424	ILDR2
ENSGALG00000006569	8235,42763	0,500306434	0,1289293	3,88047121	0,00010425	0,00119425	
ENSGALG00000012449	8575,6897	0,498545559	0,13392532	3,72256398	0,00019721	0,00206435	MBD5
ENSGALG00000004710	3763,43768	0,498075539	0,10011571	4,97499881	6,52E-07	1,22E-05	TMOD3
ENSGALG00000017388	6631,01193	0,49802444	0,09554361	5,21253541	1,86E-07	3,75E-06	
ENSGALG00000004909	2041,96193	0,497587671	0,13255196	3,75390642	0,0001741	0,00185173	
ENSGALG00000005095	2394,73163	0,496064325	0,13816886	3,59027581	0,00033033	0,00322566	SLC25A25
ENSGALG00000003770	2099,85738	0,492649703	0,12147281	4,05563753	5,00E-05	0,00062331	ANXA2
ENSGALG00000001007	23689,0314	0,492162491	0,08170528	6,0236317	1,71E-09	4,70E-08	
ENSGALG00000027925	2899,91937	0,491701601	0,15107329	3,25472237	0,00113503	0,0090607	
ENSGALG00000011054	3850,38645	0,491324799	0,13238005	3,71147149	0,00020606	0,00214725	
ENSGALG00000009503	5216,27727	0,49017284	0,10922838	4,48759608	7,20E-06	0,00011097	RAB3GAP2
ENSGALG00000000936	1155,34155	0,490067343	0,13552456	3,61607777	0,0002991	0,00297412	
ENSGALG00000011539	2399,98235	0,490040841	0,15085476	3,24842803	0,00116045	0,00921652	
ENSGALG00000016768	7017,45831	0,489550679	0,0912379	5,3656504	8,07E-08	1,73E-06	LONRF2
ENSGALG00000016839	2941,70522	0,488322587	0,14416141	3,38733229	0,00070576	0,00607593	
ENSGALG00000001962	47390,8257	0,488180846	0,07927072	6,15840034	7,35E-10	2,15E-08	PTBP1
ENSGALG00000008321	2140,81231	0,487472572	0,11701276	4,16597802	3,10E-05	0,00040481	RNPEPL1
ENSGALG00000026231	4317,60577	0,487086101	0,13624774	3,57500308	0,00035022	0,00339345	ARID3A
ENSGALG00000002316	5849,0452	0,486190455	0,10164247	4,78333983	1,72E-06	3,00E-05	NUBP2
ENSGALG00000005727	11457,1676	0,485528575	0,09017413	5,38434468	7,27E-08	1,58E-06	
ENSGALG00000013661	3653,88906	0,485373842	0,14009384	3,4646338	0,00053095	0,00476108	MICU3
ENSGALG00000006237	12165,443	0,48464383	0,09005848	5,38143497	7,39E-08	1,60E-06	PKN2
ENSGALG00000006121	10037,2715	0,483017989	0,10132916	4,76682134	1,87E-06	3,22E-05	PIK3C2A
ENSGALG00000003835	26017,6944	0,482616746	0,0853296	5,65591273	1,55E-08	3,75E-07	
ENSGALG00000002219	2154,95524	0,481514535	0,11726813	4,10609869	4,02E-05	0,00051051	WBP2
ENSGALG00000014719	2318,70089	0,480107075	0,11701682	4,10288958	4,08E-05	0,00051712	
ENSGALG00000007271	2336,70848	0,480045702	0,10812496	4,43973058	9,01E-06	0,00013558	
ENSGALG00000028128	2623,01722	0,475145623	0,1057506	4,49307716	7,02E-06	0,00010841	
ENSGALG00000002830	1989,1104	0,475072145	0,13713011	3,46438965	0,00053144	0,00476199	NUDT3
ENSGALG00000016283	1439,58733	0,474889919	0,14244125	3,33393539	0,00085627	0,00717416	BEND6
ENSGALG00000014953	9336,02158	0,474792932	0,1011178	4,69544373	2,66E-06	4,43E-05	RWDD1
ENSGALG00000014873	10464,2969	0,474224225	0,08830699	5,37017768	7,87E-08	1,69E-06	GJA1
ENSGALG00000008181	4533,21462	0,473531005	0,11780023	4,01978013	5,83E-05	0,00071091	MADD
ENSGALG00000009532	10930,7149	0,469457498	0,09307351	5,0439434	4,56E-07	8,72E-06	GXYLT1
ENSGALG00000009086	21565,5654	0,468835158	0,07859861	5,96492909	2,45E-09	6,53E-08	EHD3
ENSGALG00000016808	38690,2199	0,46860233	0,08636591	5,42577901	5,77E-08	1,28E-06	RANBP2
ENSGALG00000012033	1874,0337	0,467396297	0,12741413	3,66832386	0,00024415	0,00249111	SGSM3
ENSGALG00000005422	1476,40233	0,465795544	0,13673042	3,40667103	0,0006576	0,00570455	PDLIM1
ENSGALG00000000874	1565,53262	0,465401247	0,12194279	3,8165539	0,00013533	0,00148493	
ENSGALG00000003113	3657,12064	0,464288641	0,09568682	4,85216929	1,22E-06	2,19E-05	RGS14
ENSGALG00000002726	8476,00798	0,464112357	0,12109197	3,83272605	0,00012673	0,00140434	KIF1B
ENSGALG00000025747	1304,00667	0,463404585	0,13117609	3,5326909	0,00041135	0,00388056	NQO2
ENSGALG00000015036	6732,47434	0,461955249	0,11674428	3,95698403	7,59E-05	0,00090425	RIC1
ENSGALG00000016495	2231,85951	0,461211494	0,11447108	4,02906563	5,60E-05	0,00068541	KLHL29
ENSGALG00000000890	12926,8583	0,459656521	0,08135785	5,64981129	1,61E-08	3,87E-07	
ENSGALG00000004144	25688,7242	0,459129046	0,08737901	5,25445453	1,48E-07	3,05E-06	
ENSGALG00000009850	10767,8502	0,458569963	0,088576	5,17713554	2,25E-07	4,48E-06	NOVA1
ENSGALG00000014688	8715,47092	0,458090231	0,11541233	3,96916206	7,21E-05	0,00086337	ZYX
ENSGALG00000013480	4689,04979	0,457972294	0,11338344	4,03914621	5,36E-05	0,00066049	
ENSGALG00000015940	2526,66559	0,457737572	0,10654486	4,2961959	1,74E-05	0,00024177	
ENSGALG00000005134	3403,87437	0,45766228	0,10876996	4,20761648	2,58E-05	0,00034271	ZSWIM8
ENSGALG00000003295	2431,84104	0,455390197	0,14052	3,24075001	0,00119216	0,00942648	
ENSGALG00000011286	5006,91718	0,454714572	0,0910514	4,99404243	5,91E-07	1,12E-05	UBE2E1
ENSGALG00000010361	8260,44964	0,453176333	0,09954173	4,55262667	5,30E-06	8,39E-05	
ENSGALG00000012380	1446,14742	0,453021582	0,1253689	3,61350847	0,00030208	0,00299425	SPOPL
ENSGALG00000002672	3384,73075	0,451332604	0,11773512	3,83345767	0,00012635	0,00140246	
ENSGALG00000008521	14688,0623	0,447794364	0,09286454	4,82201686	1,42E-06	2,51E-05	
ENSGALG00000014371	4193,46245	0,447550868	0,10744176	4,16552078	3,11E-05	0,0004052	LGALS8
ENSGALG00000006947	14379,0645	0,446651103	0,08611381	5,18675352	2,14E-07	4,26E-06	BDH1
ENSGALG00000001596	11592,0471	0,445911724	0,08108031	5,49963016	3,81E-08	8,68E-07	MLLT1
ENSGALG00000016746	1667,79177	0,445777139	0,13374077	3,33314327	0,00085871	0,0071898	

ENSGALG00000006313	1456,17134	0,445448611	0,12850341	3,46643413	0,00052741	0,0047361	
ENSGALG00000003263	4582,86968	0,445240965	0,0980725	4,53991643	5,63E-06	8,89E-05	
ENSGALG00000009300	5731,29585	0,444814977	0,09141833	4,86570901	1,14E-06	2,05E-05	NUMB
ENSGALG00000003862	1841,95984	0,443615862	0,11893475	3,72990962	0,00019155	0,0020186	
ENSGALG00000005505	1365,18076	0,442779592	0,12653606	3,49923634	0,00046659	0,0042759	PIK3CB
ENSGALG00000005785	2692,43001	0,442398124	0,13426574	3,29494421	0,00098441	0,00803597	
ENSGALG00000004169	13236,4427	0,441293938	0,09199916	4,79671711	1,61E-06	2,83E-05	
ENSGALG00000012462	8197,06854	0,439936793	0,10637709	4,13563485	3,54E-05	0,00045554	KIF5C
ENSGALG00000012093	1956,24055	0,439445143	0,11904646	3,69137507	0,00022304	0,00230204	
ENSGALG00000001957	8367,03127	0,438219772	0,11967372	3,66178782	0,00025046	0,0025415	
ENSGALG00000012799	2784,84231	0,438081252	0,12516354	3,5000707	0,00046513	0,00426567	RREB1
ENSGALG00000013861	1114,81045	0,437973226	0,13471835	3,25102872	0,00114988	0,00916171	TNFAIP3
ENSGALG00000014419	9771,32739	0,437804845	0,11330954	3,86379513	0,00011164	0,00126494	
ENSGALG00000026184	2048,29159	0,437296545	0,12629201	3,4625829	0,00053502	0,00478722	
ENSGALG00000010030	8265,21081	0,435973553	0,09014275	4,83647923	1,32E-06	2,35E-05	
ENSGALG00000011464	75594,5083	0,435819807	0,08481234	5,13863704	2,77E-07	5,46E-06	EIF5
ENSGALG00000011643	21083,8275	0,435706974	0,11836787	3,68095649	0,00023236	0,00238441	UTP20
ENSGALG00000011016	2374,56332	0,434866192	0,12967523	3,35350229	0,00079796	0,00676722	PGM1
ENSGALG00000011707	2790,34805	0,43414341	0,11909866	3,64524173	0,00026714	0,00268407	
ENSGALG00000004089	7953,41422	0,433449007	0,08322806	5,20796739	1,91E-07	3,84E-06	
ENSGALG00000014686	1896,32829	0,43250268	0,11603822	3,72724338	0,00019359	0,00203322	FBN2
ENSGALG00000012414	1673,15959	0,431565773	0,12254766	3,52161578	0,00042893	0,00400704	GNPNAT1
ENSGALG00000009534	2593,70172	0,430698068	0,10888319	3,95559732	7,63E-05	0,00090864	
ENSGALG00000001376	6656,04949	0,43069552	0,08629025	4,99124172	6,00E-07	1,13E-05	ARID3B
ENSGALG00000016751	17995,0581	0,429916343	0,11291663	3,80737831	0,00014045	0,00153438	
ENSGALG00000007659	32471,1723	0,429907921	0,0785478	5,47320101	4,42E-08	1,00E-06	HYOU1
ENSGALG00000013134	6778,47765	0,429746745	0,09606485	4,47350681	7,69E-06	0,00011753	
ENSGALG00000010967	19058,4836	0,429437085	0,07951282	5,4008533	6,63E-08	1,45E-06	
ENSGALG00000000243	3939,58343	0,429169147	0,10346666	4,14789781	3,36E-05	0,0004354	RDM1
ENSGALG00000011682	2443,78682	0,428788464	0,10784939	3,97580809	7,01E-05	0,00084122	CNOT4
ENSGALG00000014784	3810,58741	0,427987842	0,11343575	3,77295391	0,00016133	0,00173519	MAST4
ENSGALG00000004638	3143,25207	0,427666392	0,1185082	3,60874933	0,00030768	0,00304007	
ENSGALG00000008128	9640,95815	0,426089907	0,09019502	4,72409566	2,31E-06	3,91E-05	
ENSGALG00000010975	6422,3758	0,425801788	0,09591443	4,43939214	9,02E-06	0,00013563	TMTC2
ENSGALG00000004184	14964,7344	0,42382749	0,07954333	5,32825936	9,92E-08	2,10E-06	SPARC
ENSGALG00000009298	10491,8514	0,42324599	0,12277761	3,44725699	0,00056631	0,00503123	TNIK
ENSGALG00000015033	5942,94152	0,422029877	0,11922305	3,5398347	0,00040038	0,00379131	REV3L
ENSGALG00000008009	4573,71765	0,421760643	0,09376655	4,49798603	6,86E-06	0,0001066	BCAP29
ENSGALG00000001546	5321,72397	0,420857037	0,11791501	3,56915579	0,00035813	0,00345138	ZZEF1
ENSGALG00000009570	4990,17008	0,419915848	0,09759573	4,30260451	1,69E-05	0,00023646	ATP6V1D
ENSGALG00000011246	4677,48231	0,419525625	0,09763	4,29709732	1,73E-05	0,00024132	
ENSGALG00000015942	2654,62871	0,419245968	0,10972783	3,82078074	0,00013303	0,00146485	
ENSGALG00000004531	6608,4459	0,419189684	0,08983065	4,66644405	3,06E-06	5,03E-05	PLOD1
ENSGALG00000012251	1948,91488	0,417627661	0,12070121	3,46001212	0,00054015	0,00482625	
ENSGALG00000012739	5567,17218	0,417000863	0,09837079	4,23907217	2,24E-05	0,0003031	HIVEP1
ENSGALG00000020805	5785,80923	0,416086722	0,09259199	4,49376567	7,00E-06	0,0001082	LRRC8D
ENSGALG00000023806	3007,51007	0,415743003	0,11577931	3,59082288	0,00032964	0,00322241	
ENSGALG00000001443	21339,3728	0,41409085	0,11660287	3,5512922	0,00038334	0,00365771	
ENSGALG00000003181	2537,40381	0,412959133	0,11077844	3,72779349	0,00019316	0,00203049	SURF2
ENSGALG00000005723	2106,65447	0,412949734	0,1250895	3,3012343	0,0009626	0,00789588	
ENSGALG00000007141	2241,98683	0,412800257	0,11510085	3,58642238	0,00033525	0,00327113	LRRK1
ENSGALG00000005660	9409,08057	0,411134823	0,12248124	3,35671682	0,00078874	0,00669812	NF1
ENSGALG00000006863	4226,48137	0,410961417	0,09352764	4,39401049	1,11E-05	0,00016357	
ENSGALG00000005249	4537,35945	0,410952179	0,10151336	4,04825713	5,16E-05	0,00063971	BICD2
ENSGALG00000004644	3446,25125	0,409508513	0,12409834	3,2998709	0,00096729	0,00792395	
ENSGALG00000001194	18631,7262	0,408514612	0,08737937	4,67518369	2,94E-06	4,84E-05	
ENSGALG00000005962	14577,1851	0,407910696	0,09612843	4,24339276	2,20E-05	0,00029838	
ENSGALG00000028637	3638,50777	0,407745497	0,1057417	3,85605195	0,00011523	0,0013033	
ENSGALG00000005796	13049,6305	0,407045166	0,09023433	4,51097882	6,45E-06	0,00010115	XBP1
ENSGALG00000011254	1903,62474	0,406188469	0,11859058	3,42513271	0,0006145	0,00539801	SATB1
ENSGALG00000023451	10229,4738	0,405630741	0,10390284	3,90394266	9,46E-05	0,00109512	TRPM7
ENSGALG00000002399	3646,29569	0,405489945	0,09701983	4,17945444	2,92E-05	0,00038355	

ENSGALG00000012814	2642,59396	0,404889777	0,11881733	3,40766613	0,00065521	0,00569168	PXDC1
ENSGALG00000028267	14506,7903	0,403950335	0,07758347	5,2066546	1,92E-07	3,86E-06	
ENSGALG00000017085	3891,4285	0,403507696	0,11323732	3,56338096	0,00036611	0,00352281	SLC7A1
ENSGALG00000009748	12590,9107	0,403044512	0,10500934	3,83817788	0,00012395	0,001384	ASNS
ENSGALG00000010780	2934,00779	0,401358719	0,11055482	3,63040448	0,00028298	0,00282729	
ENSGALG00000014854	14435,0938	0,401311952	0,08622832	4,65406195	3,25E-06	5,33E-05	
ENSGALG00000011101	5371,2295	0,40109735	0,0891436	4,49945224	6,81E-06	0,00010613	URB2
ENSGALG00000008855	8350,52016	0,400902672	0,10842426	3,69753673	0,0002177	0,00225977	UGP2
ENSGALG00000026850	2047,38224	0,400065314	0,11989409	3,33682262	0,00084742	0,00711913	
ENSGALG00000013830	3806,14371	0,399422	0,11396388	3,50481227	0,00045693	0,00421349	PRELID3A
ENSGALG00000015459	1707,50104	0,399319665	0,11963093	3,3379299	0,00084405	0,00709694	
ENSGALG00000005582	5939,79295	0,398284507	0,09131089	4,36185098	1,29E-05	0,00018737	KLHL18
ENSGALG00000008195	5223,77429	0,394418985	0,08931317	4,41613483	1,00E-05	0,00014928	NPHP1
ENSGALG00000012404	2166,93397	0,394285725	0,11188253	3,52410442	0,00042492	0,00397553	ERO1A
ENSGALG00000012342	4988,06672	0,391855224	0,11601632	3,37758709	0,00073125	0,00626949	SASH1
ENSGALG00000011542	3470,24914	0,391703901	0,11096577	3,52995262	0,00041563	0,00391209	
ENSGALG00000028203	5589,34557	0,39127665	0,10019534	3,90513811	9,42E-05	0,00109174	SPRED1
ENSGALG00000017283	2269,10202	0,390659364	0,1093337	3,57309192	0,00035279	0,00341304	CCND2
ENSGALG00000008676	3130,52664	0,390658638	0,11592196	3,37001398	0,00075164	0,00643113	RRBP1
ENSGALG00000002763	7822,98963	0,38996775	0,09784285	3,98565421	6,73E-05	0,00081175	FRZB
ENSGALG00000012280	4546,76644	0,388208613	0,09700097	4,0021108	6,28E-05	0,00075985	
ENSGALG00000014902	5801,5163	0,387988657	0,11314858	3,42901912	0,00060577	0,00534005	
ENSGALG00000016779	3110,09587	0,387851139	0,10566697	3,67050494	0,00024207	0,00247392	RNF149
ENSGALG00000026822	5680,41299	0,384087371	0,09933111	3,86673784	0,0001103	0,00125318	WDR81
ENSGALG00000008708	8378,00616	0,384060223	0,08161607	4,70569382	2,53E-06	4,24E-05	
ENSGALG00000004200	10300,1977	0,383702067	0,10034391	3,82386986	0,00013137	0,00145044	DPP9
ENSGALG00000019077	12866,6527	0,383662275	0,10944193	3,50562403	0,00045554	0,00420541	
ENSGALG00000008542	22218,9525	0,383345327	0,115816	3,3099513	0,00093312	0,00768428	ADD3
ENSGALG00000015765	12660,3185	0,383191974	0,11316078	3,38626134	0,00070852	0,0060955	
ENSGALG00000001551	13003,1544	0,382951597	0,10019028	3,82224294	0,00013224	0,00145747	CCNL2
ENSGALG00000012608	2396,75353	0,382010791	0,11550101	3,30742386	0,00094158	0,00774886	DAPK1
ENSGALG00000008520	50311,2923	0,381829873	0,07410654	5,15244531	2,57E-07	5,10E-06	EIF4G1
ENSGALG00000000464	4473,94851	0,378042232	0,09693774	3,89984576	9,63E-05	0,00111074	
ENSGALG00000013628	17850,2484	0,377744473	0,0965373	3,91293791	9,12E-05	0,00106213	C6orf62
ENSGALG00000004509	60307,8172	0,377502687	0,10213082	3,69626592	0,00021879	0,00226752	
ENSGALG00000000385	3155,8316	0,377133979	0,11668273	3,23213188	0,0012287	0,00967875	
ENSGALG00000008240	2335,47856	0,376593038	0,112924	3,33492474	0,00085322	0,00715348	CFLAR
ENSGALG00000016457	20077,635	0,37591995	0,08487595	4,42905171	9,46E-06	0,00014162	TRIB2
ENSGALG00000004002	12560,3704	0,375282513	0,09982845	3,75927432	0,00017041	0,00181569	LYPLA2
ENSGALG00000004384	6339,40502	0,37272222	0,09191076	4,05526203	5,01E-05	0,00062331	
ENSGALG00000014213	4846,8151	0,372421493	0,1124273	3,3125538	0,00092448	0,00763324	PRR5
ENSGALG00000017335	8042,45001	0,370836945	0,08378084	4,42627398	9,59E-06	0,00014329	
ENSGALG00000013605	5142,50116	0,369173077	0,09448513	3,90720816	9,34E-05	0,00108344	LRRC16A
ENSGALG00000009428	6757,25752	0,369028406	0,11310884	3,26259556	0,00110397	0,00886364	SUSD6
ENSGALG00000003180	15013,9328	0,367625634	0,08522459	4,31360964	1,61E-05	0,00022599	
ENSGALG00000008414	11352,9971	0,366746111	0,095194	3,85261804	0,00011686	0,00131933	DVL3
ENSGALG00000003748	6555,91118	0,36621302	0,08463061	4,32719328	1,51E-05	0,00021492	ANO5
ENSGALG00000006336	6628,48994	0,365567709	0,10726355	3,4081263	0,00065411	0,00569	
ENSGALG00000005845	8308,86511	0,364474801	0,09382855	3,88447647	0,00010255	0,00117581	SLC7A5
ENSGALG00000011288	4443,75245	0,36304574	0,0964958	3,76229586	0,00016836	0,00179695	DACT2
ENSGALG00000016736	21468,1233	0,362152411	0,10050997	3,60314896	0,00031439	0,00309657	CYFIP1
ENSGALG00000004696	6019,01081	0,360778491	0,09380179	3,84617901	0,00011997	0,00134597	LEO1
ENSGALG00000009562	5205,50613	0,359101036	0,11011889	3,26103035	0,00111008	0,00890653	
ENSGALG00000007145	42543,9665	0,358001987	0,07128079	5,02241869	5,10E-07	9,69E-06	ITGB1
ENSGALG00000023857	2295,50244	0,357227793	0,10931817	3,26778052	0,00108394	0,00871965	
ENSGALG00000010603	12138,5477	0,356862546	0,08271823	4,31419487	1,60E-05	0,00022565	
ENSGALG00000007514	7096,59375	0,355063614	0,08859236	4,00783581	6,13E-05	0,00074492	
ENSGALG00000003563	12215,1985	0,354826794	0,08437076	4,20556587	2,60E-05	0,0003451	YARS
ENSGALG00000009946	16182,3882	0,351940163	0,09459123	3,72064246	0,00019872	0,00207838	HECTD1
ENSGALG00000017112	5413,39298	0,351627272	0,10588126	3,32095857	0,00089709	0,00746122	MTMR6
ENSGALG00000005160	8835,93843	0,351320099	0,09529673	3,68659118	0,00022728	0,00234186	VMP1
ENSGALG00000002954	6375,29438	0,350618821	0,09690427	3,61819794	0,00029666	0,00295222	MBTD1

ENSGALG00000008099	7103,82231	0,350057141	0,08535874	4,10101087	4,11E-05	0,00052028	
ENSGALG00000005678	19719,4298	0,349721909	0,0904842	3,8650054	0,00011109	0,00125982	FLNB
ENSGALG00000014795	5131,9149	0,349663833	0,09082989	3,84965603	0,00011828	0,00133083	TGIF1
ENSGALG00000015013	15895,2683	0,348772486	0,09941333	3,50830713	0,00045097	0,00416629	MSX1
ENSGALG00000011047	7708,25037	0,346858574	0,09143673	3,79342699	0,00014858	0,00161338	LEPROT
ENSGALG00000001749	4850,90171	0,34679817	0,10645528	3,25768863	0,00112324	0,00897799	ACSBG2
ENSGALG00000015667	5950,72909	0,346421134	0,09935991	3,48652816	0,00048933	0,00445169	
ENSGALG00000003427	8942,13088	0,346212827	0,10652898	3,24994036	0,00115429	0,00918514	USP3
ENSGALG00000009540	6305,96328	0,344720194	0,10105941	3,41106481	0,0006471	0,00564079	YAF2
ENSGALG00000006437	3815,5669	0,344612756	0,0964691	3,57226061	0,00035391	0,00342125	
ENSGALG00000001003	4334,6017	0,344161191	0,10407482	3,30686318	0,00094347	0,00775418	SEPN1
ENSGALG00000028726	5513,31502	0,343861226	0,09087882	3,7837332	0,00015449	0,00167032	
ENSGALG00000014139	7333,64409	0,34378877	0,09116278	3,7711527	0,0001625	0,00174327	
ENSGALG00000007234	4391,88237	0,343143822	0,09912131	3,46185737	0,00053646	0,00479671	CLCN5
ENSGALG00000002521	2909,63624	0,341377701	0,10552536	3,23502994	0,0012163	0,00959312	
ENSGALG00000025884	6496,58774	0,339208538	0,0928033	3,65513456	0,00025705	0,00260356	NABP1
ENSGALG00000000546	3505,59123	0,338887883	0,09640887	3,51511115	0,00043957	0,00408392	
ENSGALG00000013677	5281,75302	0,33880019	0,09391468	3,60753163	0,00030912	0,00305196	
ENSGALG00000026349	30008,024	0,337656858	0,07971202	4,23595916	2,28E-05	0,00030611	
ENSGALG00000012202	7460,55254	0,337252452	0,08638462	3,90407991	9,46E-05	0,00109512	
ENSGALG00000010976	5122,33312	0,337203087	0,10038342	3,35915108	0,00078182	0,00665294	CCDC126
ENSGALG00000006417	9187,43681	0,336436699	0,08945097	3,76112978	0,00016915	0,00180381	SEC24A
ENSGALG00000006975	7222,31417	0,33439022	0,08487768	3,93967199	8,16E-05	0,00096378	
ENSGALG00000002362	7456,91772	0,333268709	0,08848366	3,76644362	0,00016559	0,00177191	MANF
ENSGALG00000014370	4742,98825	0,332141176	0,09950672	3,33787686	0,00084421	0,00709694	
ENSGALG00000003767	4420,56627	0,331888441	0,0963142	3,44589313	0,00056918	0,00504594	NKD1
ENSGALG00000006256	7680,29257	0,33180927	0,08270052	4,01217858	6,02E-05	0,00073206	
ENSGALG00000002548	7217,2063	0,330998688	0,08817899	3,75371385	0,00017423	0,00185173	TLN1
ENSGALG00000004691	5665,22107	0,330681288	0,10090233	3,27724134	0,00104827	0,00847626	
ENSGALG00000005139	48915,1737	0,327662098	0,07590851	4,31653954	1,58E-05	0,00022428	CLTC
ENSGALG00000011055	8298,54873	0,326999676	0,09831999	3,32587164	0,00088143	0,0073456	
ENSGALG00000016630	13012,9547	0,325439015	0,08809758	3,6940745	0,00022069	0,00228149	EXTL3
ENSGALG00000011630	13213,6043	0,324450144	0,08177951	3,96737719	7,27E-05	0,00086903	GLI2
ENSGALG00000015049	15025,2928	0,323309666	0,07842419	4,12257561	3,75E-05	0,00047969	
ENSGALG00000004132	40450,1788	0,317911072	0,09276067	3,42721839	0,0006098	0,00536425	
ENSGALG00000009448	18330,278	0,31659087	0,08894466	3,55941395	0,00037168	0,00356548	IFRD1
ENSGALG00000000258	23571,1755	0,314352619	0,08646657	3,63553944	0,0002774	0,00278044	
ENSGALG00000004063	4760,35876	0,313966155	0,0919727	3,41368847	0,0006409	0,00560237	SLC25A14
ENSGALG00000008315	6755,45974	0,312048416	0,08519158	3,66290207	0,00024937	0,00253574	
ENSGALG00000007241	5082,09102	0,311992362	0,09448776	3,30193399	0,00096021	0,00788138	NUBP1
ENSGALG00000009992	11411,8856	0,311546697	0,0854138	3,64749847	0,00026481	0,00266918	ARHGAP5
ENSGALG00000008837	13220,7097	0,311145069	0,0811437	3,83449427	0,00012582	0,0013978	PELI1
ENSGALG00000008265	23891,3346	0,310830618	0,08922654	3,48361172	0,0004947	0,00449394	
ENSGALG00000010084	23564,6118	0,310711961	0,07381728	4,20920342	2,56E-05	0,00034103	EIF4G3
ENSGALG00000011228	6953,04279	0,310544896	0,0872542	3,55908236	0,00037215	0,0035661	WARS
ENSGALG00000004938	5563,15704	0,309369487	0,09539583	3,24300846	0,00118275	0,00936984	
ENSGALG00000017111	14220,273	0,309121441	0,09000983	3,43430744	0,00059407	0,00525175	NUP58
ENSGALG00000006971	10261,5906	0,308524903	0,09239987	3,33901865	0,00084075	0,00708211	
ENSGALG00000003039	8205,74635	0,308092774	0,08797415	3,50208302	0,00046164	0,0042398	SRM
ENSGALG00000000346	4708,45419	0,306247189	0,08995671	3,40438422	0,00066313	0,00574456	
ENSGALG00000002863	20573,4084	0,306238468	0,09158034	3,34393231	0,000826	0,00697665	ERGIC1
ENSGALG00000003830	15302,2463	0,304702804	0,07866157	3,87359158	0,00010724	0,00122512	
ENSGALG00000021041	6232,92756	0,302815674	0,08606635	3,51839787	0,00043416	0,00404688	
ENSGALG00000005397	4086,38441	0,301881405	0,09352409	3,22784652	0,00124726	0,00980639	GGNBP2
ENSGALG00000010349	6576,00357	0,300633215	0,09122991	3,29533608	0,00098304	0,00803191	POLR1C
ENSGALG00000004295	16498,2044	0,300034342	0,08565811	3,50269613	0,00046057	0,00423316	CPD
ENSGALG00000007387	13168,5046	0,298075088	0,08883093	3,35553249	0,00079212	0,0067223	GTF3C1
ENSGALG00000011357	6177,88173	0,296426831	0,09147912	3,24037681	0,00119372	0,00943286	VEZT
ENSGALG00000010536	11637,7722	0,29615271	0,08796769	3,36660761	0,00076099	0,00649324	FAF1
ENSGALG00000011400	11997,2666	0,295706421	0,08251808	3,58353479	0,00033898	0,00330495	USP44
ENSGALG00000012138	28311,992	0,295412949	0,08299351	3,55947062	0,0003716	0,00356548	
ENSGALG00000002985	13749,5981	0,293105421	0,07993628	3,6667381	0,00024566	0,00250449	UTP18

ENSGALG00000003391	11311,3076	0,293097669	0,08152671	3,59511199	0,00032425	0,00317625	EIF2AK1
ENSGALG00000006856	8627,6039	0,290795459	0,08950457	3,2489453	0,00115834	0,00920562	CD151
ENSGALG00000006895	13236,4315	0,290027475	0,08022918	3,61498721	0,00030036	0,00298275	CEP131
ENSGALG00000026077	76281,091	0,289436865	0,08082386	3,58108204	0,00034217	0,00333353	ALDOC
ENSGALG00000004625	14027,7064	0,287002136	0,08764175	3,27471937	0,00105767	0,00854125	ATXN2
ENSGALG00000004098	13227,4006	0,28601688	0,0879168	3,25326778	0,00114086	0,00910141	STK4
ENSGALG00000005464	21720,0724	0,284610322	0,07882026	3,61087753	0,00030516	0,00301783	
ENSGALG00000016910	23116,5508	0,283615388	0,07814404	3,62939229	0,00028409	0,00283596	RBM26
ENSGALG00000015995	13978,5364	0,283340125	0,08204665	3,45340254	0,00055356	0,00494255	PTPRA
ENSGALG00000009325	20595,5323	0,276324484	0,08171116	3,38172254	0,00072033	0,00618434	
ENSGALG00000024094	16575,1537	0,274969008	0,08126295	3,38369463	0,00071517	0,00614658	UBAP2L
ENSGALG00000003339	10161,4212	0,27482717	0,08370391	3,28332526	0,0010259	0,00831154	MTOR
ENSGALG00000010481	11769,112	0,274549325	0,08028743	3,41958023	0,00062718	0,00549396	CMPK1
ENSGALG00000005243	13689,9827	0,272933583	0,07658125	3,56397392	0,00036528	0,00351756	PPP3CB
ENSGALG00000009782	45274,5885	0,272414043	0,08362168	3,25769643	0,0011232	0,00897799	NAA15
ENSGALG00000011596	8125,50614	0,272230864	0,08398232	3,24152578	0,00118892	0,00940681	
ENSGALG00000006935	12087,4137	0,270244925	0,0815817	3,31256806	0,00092444	0,00763324	
ENSGALG00000006564	13301,7317	0,268766276	0,07743018	3,47107908	0,00051837	0,00467506	
ENSGALG00000008348	63887,3158	0,267187928	0,07700129	3,46991508	0,00052062	0,00469198	PDIA3
ENSGALG00000003078	20092,3466	0,264245742	0,07691385	3,43560664	0,00059123	0,00523033	MMD
ENSGALG00000012137	17323,9143	0,256853565	0,07795662	3,29482705	0,00098482	0,00803597	KTN1
ENSGALG00000001364	18433,6359	0,254871955	0,07722743	3,30027759	0,00096589	0,00791766	CSNK1A1
ENSGALG00000010318	32908,0068	0,246411634	0,0714677	3,44787431	0,00056502	0,00502688	TMED10
ENSGALG00000002745	41084,1287	0,246172053	0,07133347	3,45100341	0,00055851	0,00497604	
ENSGALG00000005930	23930,3439	-0,243448299	0,0744484	-3,2700274	0,00107537	0,00867299	PLS3
ENSGALG00000020813	18469,9592	-0,256523625	0,07948115	-3,2274775	0,00124887	0,00980672	
ENSGALG00000026300	10090,9313	-0,257041042	0,0792187	-3,2447017	0,00117574	0,00932021	
ENSGALG00000000812	26499,6103	-0,265852554	0,08212241	-3,2372716	0,00120679	0,00952409	OAZ1
ENSGALG00000029037	7790,04361	-0,270845781	0,08268109	-3,2757889	0,00105367	0,00851447	
ENSGALG00000014756	8148,92025	-0,271609614	0,08352364	-3,2518892	0,00114641	0,00913984	PPWD1
ENSGALG00000002377	301877,116	-0,272471138	0,0772308	-3,5280112	0,00041869	0,00393497	ENO1
ENSGALG00000020003	18619,6916	-0,275409017	0,07553559	-3,6460829	0,00026627	0,0026796	MAP3K4
ENSGALG00000004048	24216,2179	-0,278746659	0,07841685	-3,554678	0,00037844	0,00361645	PTPRS
ENSGALG00000027963	8992,02102	-0,279887477	0,08105728	-3,4529592	0,00055447	0,00494715	COX7C
ENSGALG00000023546	8219,08438	-0,280708865	0,08585195	-3,2696854	0,00107667	0,00867661	
ENSGALG00000012618	8450,7599	-0,282562019	0,08497947	-3,325062	0,00088399	0,00736206	FANCC
ENSGALG00000010127	15511,214	-0,2840371	0,08325403	-3,4116921	0,00064561	0,00563262	GLO1
ENSGALG00000010829	19262,8491	-0,284700815	0,07724408	-3,68573	0,00022805	0,00234786	
ENSGALG00000014646	9607,01137	-0,290131032	0,08710762	-3,3307195	0,00086622	0,007243	SYT11
ENSGALG00000012185	5616,14478	-0,291440425	0,08719363	-3,342451	0,00083042	0,00699981	PLA2G12A
ENSGALG00000011276	26271,7672	-0,293405435	0,08969033	-3,2713161	0,00107048	0,00863913	BTG1
ENSGALG00000012569	7527,80321	-0,295102597	0,09077738	-3,2508383	0,00115065	0,00916201	FBXO7
ENSGALG00000027710	10733,9814	-0,296769112	0,08642619	-3,4337868	0,00059521	0,00525813	
ENSGALG00000001026	8524,74214	-0,297852025	0,08248786	-3,6108589	0,00030518	0,00301783	
ENSGALG00000013837	12275,1858	-0,300523253	0,08475438	-3,5458138	0,0003914	0,00371758	
ENSGALG00000027695	6526,32859	-0,301428682	0,09097649	-3,3132592	0,00092215	0,00762408	PCBP4
ENSGALG00000004769	29935,7205	-0,301514296	0,08604923	-3,5039743	0,00045837	0,0042222	
ENSGALG00000015326	8955,0401	-0,301560094	0,08584014	-3,513043	0,00044301	0,0041079	CLTA
ENSGALG00000013207	7371,39959	-0,304013471	0,09243698	-3,2888729	0,00100589	0,00818655	CTNNAL1
ENSGALG00000006094	12757,9566	-0,306148177	0,08654729	-3,5373513	0,00040416	0,00382136	PHF6
ENSGALG00000012121	14730,7454	-0,306219207	0,07698902	-3,9774401	6,97E-05	0,00083828	RCN1
ENSGALG00000007127	7771,85476	-0,307935097	0,08245774	-3,7344594	0,00018812	0,00198413	
ENSGALG00000009559	5261,85996	-0,308352593	0,09367103	-3,2918672	0,00099525	0,00810516	
ENSGALG00000009474	18920,9808	-0,308530687	0,08226101	-3,7506307	0,00017639	0,0018683	
ENSGALG00000002040	12321,1382	-0,309590748	0,08819991	-3,5101026	0,00044793	0,00414132	
ENSGALG00000026981	16367,9889	-0,309740111	0,09419663	-3,2882293	0,0010082	0,00819996	NHSL1
ENSGALG00000006277	4802,8632	-0,310824597	0,09438854	-3,2930334	0,00099113	0,00807697	
ENSGALG00000012546	32458,4091	-0,312592383	0,09413799	-3,3205762	0,00089832	0,00746649	MCM5
ENSGALG00000003474	3939,39547	-0,313391117	0,09428082	-3,3240177	0,00088731	0,00738477	MED20
ENSGALG00000026437	4338,56055	-0,314002706	0,09459663	-3,3193857	0,00090216	0,00748844	
ENSGALG00000015712	7609,04078	-0,314599398	0,08949622	-3,5152257	0,00043938	0,00408392	SLBP
ENSGALG00000007131	72027,5204	-0,31501454	0,08157254	-3,8617718	0,00011257	0,00127431	

ENSGALG00000025764	4071,26079	-0,316576106	0,096448	-3,28235	0,00102946	0,00833494	FKBP10
ENSGALG00000007668	10342,6342	-0,317701794	0,08996064	-3,5315644	0,00041311	0,00389125	BMP7
ENSGALG00000001506	7912,15144	-0,318173703	0,08732074	-3,6437356	0,00026871	0,0026955	
ENSGALG00000006483	6631,82379	-0,318521408	0,08620504	-3,6949278	0,00021995	0,00227572	
ENSGALG00000023131	4044,89831	-0,321300472	0,09495836	-3,3835932	0,00071544	0,00614658	PDXP
ENSGALG00000013784	12402,0503	-0,32170776	0,08351065	-3,8522964	0,00011702	0,00131988	CDT1
ENSGALG00000021021	5260,37371	-0,322180937	0,09166421	-3,5147954	0,00044009	0,00408392	ARL4C
ENSGALG00000001475	99646,137	-0,322303202	0,09596675	-3,3584881	0,0007837	0,00666371	
ENSGALG00000016870	4880,45934	-0,322919481	0,09246763	-3,4922437	0,00047898	0,00437341	TMTC4
ENSGALG00000020528	5056,48494	-0,323800436	0,09904607	-3,2691901	0,00107856	0,00868191	INIP
ENSGALG00000007965	19359,9019	-0,323830546	0,09621896	-3,3655586	0,00076389	0,00650475	HN1
ENSGALG00000006780	12874,7901	-0,325989737	0,10037051	-3,2478639	0,00116275	0,00922895	
ENSGALG00000009362	9549,85526	-0,327914914	0,09727937	-3,3708577	0,00074935	0,00641585	
ENSGALG00000004621	10575,1137	-0,328680536	0,09230808	-3,5606907	0,00036988	0,00355364	PREX1
ENSGALG00000010758	4794,60081	-0,329891138	0,08975382	-3,6755107	0,00023737	0,00242988	
ENSGALG00000028470	4548,02852	-0,330867645	0,09287432	-3,5625312	0,0003673	0,00353152	
ENSGALG00000021365	6497,94756	-0,33098251	0,0871271	-3,7988467	0,00014537	0,00158263	DCTN3
ENSGALG00000017015	5143,28194	-0,331426117	0,09201475	-3,6018804	0,00031592	0,00310683	RNASEH2B
ENSGALG00000007254	4308,53571	-0,33313089	0,09410502	-3,5399905	0,00040014	0,00379131	ARHGAP12
ENSGALG00000003429	14328,0151	-0,333616807	0,0996214	-3,3488469	0,00081149	0,00687711	PBX1
ENSGALG00000025818	7103,10421	-0,334464872	0,08352702	-4,0042717	6,22E-05	0,00075404	
ENSGALG00000002225	3236,66516	-0,336939904	0,09933028	-3,3921168	0,00069355	0,00597493	RFFL
ENSGALG00000008677	87282,2545	-0,33704479	0,09556181	-3,5269822	0,00042033	0,00394733	VIM
ENSGALG00000001567	14603,7017	-0,337139369	0,08829903	-3,8181548	0,00013445	0,00147663	
ENSGALG00000008438	5574,45982	-0,337474785	0,10321767	-3,2695448	0,00107721	0,00867661	SLC35C1
ENSGALG00000010745	57730,9692	-0,33788407	0,10090108	-3,3486665	0,00081201	0,00687711	HMGB2
ENSGALG00000005739	5611,79649	-0,338102535	0,09729228	-3,4751219	0,00051062	0,00461848	SCD
ENSGALG00000014496	5642,31406	-0,339160901	0,09495091	-3,5719606	0,00035432	0,00342253	Prom1
ENSGALG00000006539	5064,49097	-0,340143008	0,09970044	-3,41165	0,00064571	0,00563262	C21orf2
ENSGALG00000003930	5156,97026	-0,340892203	0,10217013	-3,3365153	0,00084836	0,00712222	APBA2
ENSGALG00000004581	6531,73175	-0,341091866	0,08790547	-3,8802119	0,00010437	0,00119443	NDST1
ENSGALG00000008525	5352,16855	-0,34141534	0,09154577	-3,7294496	0,0001919	0,00201889	ABHD12
ENSGALG00000010533	8438,68556	-0,341487952	0,10389076	-3,2869907	0,00101264	0,00822408	ELAVL4
ENSGALG00000010892	4569,599	-0,341632417	0,09812504	-3,481603	0,00049842	0,0045245	
ENSGALG00000016417	9617,73728	-0,343209424	0,08745497	-3,9244129	8,69E-05	0,00101926	
ENSGALG00000000684	3976,23685	-0,343510226	0,09330701	-3,681505	0,00023186	0,00238124	KLHDC8A
ENSGALG00000010444	2882,2778	-0,345177441	0,10587293	-3,2602994	0,00111295	0,00890713	
ENSGALG00000000231	17219,2246	-0,345221534	0,08265368	-4,1767231	2,96E-05	0,00038737	
ENSGALG00000010170	3503,64077	-0,34648621	0,09982039	-3,4710967	0,00051834	0,00467506	INTU
ENSGALG00000027648	7668,3666	-0,347098945	0,08376328	-4,1438078	3,42E-05	0,00044142	
ENSGALG00000008758	4589,06242	-0,348320506	0,0918692	-3,791483	0,00014975	0,00162325	
ENSGALG00000010674	11516,7147	-0,348433039	0,07775009	-4,4814487	7,41E-06	0,00011379	DCTD
ENSGALG00000020956	5772,71418	-0,348683982	0,0891113	-3,9129044	9,12E-05	0,00106213	
ENSGALG00000016034	14272,6354	-0,349640251	0,08038023	-4,3498288	1,36E-05	0,00019567	ATRN
ENSGALG00000005961	8289,71197	-0,349909098	0,10219515	-3,4239306	0,00061722	0,00541814	dAK
ENSGALG00000001733	12421,7814	-0,350339549	0,08564084	-4,0907999	4,30E-05	0,00054208	LRIG2
ENSGALG00000014262	6153,36709	-0,35183883	0,09238387	-3,8084442	0,00013984	0,00152929	APBB2
ENSGALG00000014970	12546,5116	-0,352181226	0,08624907	-4,0833047	4,44E-05	0,00055763	FSTL1
ENSGALG00000009670	3220,19002	-0,352936897	0,10843055	-3,254958	0,00113409	0,00905897	SGCE
ENSGALG00000001329	7348,8892	-0,35438055	0,08566964	-4,1365943	3,52E-05	0,00045411	EPB41
ENSGALG00000001604	6686,31029	-0,355709643	0,09818594	-3,6228163	0,00029141	0,00290229	
ENSGALG00000000921	4280,88802	-0,356222635	0,09720598	-3,6646163	0,00024771	0,00252329	
ENSGALG00000013672	6274,94537	-0,356442405	0,09450669	-3,7716103	0,0001622	0,00174157	TSHZ1
ENSGALG00000006634	43306,474	-0,358696121	0,07111068	-5,0441945	4,55E-07	8,72E-06	
ENSGALG00000022891	4638,29282	-0,359408325	0,09123912	-3,9391911	8,18E-05	0,00096481	
ENSGALG00000006887	5925,3009	-0,35954959	0,08731049	-4,1180572	3,82E-05	0,00048789	FAM174B
ENSGALG00000017329	3279,3596	-0,359975454	0,1052627	-3,4197818	0,00062671	0,00549374	FCHSD2
ENSGALG00000009650	11565,6296	-0,362142597	0,07848435	-4,6142011	3,95E-06	6,39E-05	
ENSGALG00000007128	21899,2708	-0,362594465	0,07501963	-4,8333277	1,34E-06	2,38E-05	SH3BGRL
ENSGALG00000004954	30674,7809	-0,365189206	0,0801208	-4,5579828	5,16E-06	8,21E-05	
ENSGALG00000012395	9821,77442	-0,367131435	0,08321753	-4,4117079	1,03E-05	0,00015182	PLEKHG1
ENSGALG00000014749	4798,93922	-0,36753325	0,09967341	-3,6873752	0,00022658	0,00233659	EPHB6

ENSGALG00000011078	7107,44613	-0,368557879	0,09522589	-3,8703537	0,00010868	0,00123699	
ENSGALG00000021340	9694,86728	-0,368750238	0,08310139	-4,4373533	9,11E-06	0,0001366	CA9
ENSGALG00000016390	3066,76348	-0,369515643	0,106671	-3,4640683	0,00053207	0,00476427	
ENSGALG00000015444	4115,05377	-0,370185453	0,10012196	-3,6973451	0,00021787	0,00225977	
ENSGALG00000012130	2125,48893	-0,373712161	0,11059162	-3,3792088	0,00072695	0,00623689	DNAJC24
ENSGALG00000014033	4259,25139	-0,375904723	0,10689108	-3,516708	0,00043693	0,00406667	
ENSGALG00000021653	5151,69547	-0,375983807	0,10509679	-3,5775006	0,0003469	0,00337293	IFT22
ENSGALG00000002940	2775,52872	-0,376363633	0,10520586	-3,5774017	0,00034703	0,00337293	
ENSGALG00000006886	5794,91901	-0,376920608	0,10869717	-3,4676213	0,00052509	0,00471861	DACH2
ENSGALG00000022603	14106,8838	-0,378152541	0,07662552	-4,9350734	8,01E-07	1,48E-05	
ENSGALG00000026081	3245,15307	-0,379250879	0,10385941	-3,6515795	0,00026063	0,00263136	RBM38
ENSGALG00000015469	2385,73691	-0,382238702	0,11262796	-3,393817	0,00068926	0,00595026	
ENSGALG00000014094	3273,18619	-0,382585394	0,11483546	-3,3315964	0,00086349	0,00722505	STK38L
ENSGALG00000006702	2276,02955	-0,38370743	0,11242363	-3,4130498	0,0006424	0,0056116	MFGE8
ENSGALG00000027472	3563,95993	-0,383715502	0,11493616	-3,3385097	0,00084229	0,00709032	TMEM38B
ENSGALG00000026313	6897,17922	-0,38698684	0,11169382	-3,4647111	0,0005308	0,00476108	RND3
ENSGALG00000024047	8317,97675	-0,387381439	0,09008061	-4,3003863	1,71E-05	0,00023803	MYCL
ENSGALG00000002249	11964,7275	-0,388441944	0,08418985	-4,6138809	3,95E-06	6,39E-05	AGO1
ENSGALG00000002056	4578,49374	-0,389501294	0,10629829	-3,6642291	0,00024808	0,00252505	TANGO2
ENSGALG00000014662	2243,29	-0,390476627	0,1184381	-3,2968836	0,00097764	0,00799823	RBP5
ENSGALG00000015663	9711,19793	-0,390574583	0,09832214	-3,9723971	7,12E-05	0,00085254	HSDL2
ENSGALG00000016148	7859,64044	-0,391482924	0,08589693	-4,557589	5,17E-06	8,21E-05	C2CD2
ENSGALG00000006606	1856,635	-0,392475054	0,11686619	-3,3583285	0,00078415	0,00666371	
ENSGALG00000002599	2253,34485	-0,392633405	0,11309679	-3,4716581	0,00051725	0,00467172	ARHGEF39
ENSGALG00000015702	9863,18717	-0,39306292	0,08831382	-4,4507519	8,56E-06	0,00012928	
ENSGALG00000005214	1715,18636	-0,394482457	0,11892754	-3,3169983	0,0009099	0,00754772	
ENSGALG00000003135	8376,23704	-0,395749835	0,09573043	-4,1340026	3,56E-05	0,00045832	ANK3
ENSGALG00000014753	5047,55497	-0,396666478	0,09075263	-4,3708537	1,24E-05	0,00018023	CENPK
ENSGALG00000003457	2509,63115	-0,397746039	0,12188792	-3,2632112	0,00110157	0,00885009	CNP
ENSGALG00000009354	2023,12355	-0,398534666	0,12361626	-3,2239664	0,00126428	0,00992152	
ENSGALG00000013067	7763,34164	-0,398595631	0,09019442	-4,4192935	9,90E-06	0,00014729	
ENSGALG00000026203	4347,01953	-0,401070002	0,09158837	-4,3790496	1,19E-05	0,00017419	FAM174A
ENSGALG00000008425	3562,16922	-0,404601902	0,10623889	-3,8084161	0,00013986	0,00152929	
ENSGALG00000012936	10559,4624	-0,405432395	0,11906246	-3,4052077	0,00066114	0,00573123	
ENSGALG00000028567	2180,82431	-0,405726705	0,11524199	-3,52065	0,00043049	0,00401866	MYL9
ENSGALG00000012796	9183,72656	-0,406079242	0,08854404	-4,5861839	4,51E-06	7,22E-05	KIAA1549
ENSGALG00000014648	2054,34955	-0,406366184	0,11901606	-3,4143811	0,00063927	0,00559597	CETN3
ENSGALG00000016497	4692,83115	-0,407107351	0,09447725	-4,3090517	1,64E-05	0,00022992	UBXN2A
ENSGALG00000012361	12035,1023	-0,409904876	0,11706425	-3,5015377	0,00046258	0,00424536	NIN
ENSGALG00000015109	6799,6685	-0,409909583	0,09218338	-4,4466758	8,72E-06	0,00013143	
ENSGALG00000015386	2068,30084	-0,41009329	0,11802543	-3,4746181	0,00051158	0,00462382	RNF170
ENSGALG00000026870	1831,07326	-0,410373931	0,12408835	-3,3071109	0,00094264	0,00775242	
ENSGALG00000006158	8974,72855	-0,410915864	0,08502916	-4,8326464	1,35E-06	2,38E-05	ACVR2B
ENSGALG00000016152	6212,20219	-0,412571324	0,0962395	-4,286923	1,81E-05	0,00025069	
ENSGALG00000015713	7773,18487	-0,41471478	0,09908023	-4,1856459	2,84E-05	0,00037482	FAM53A
ENSGALG00000012082	1953,58525	-0,41500134	0,1210386	-3,4286694	0,00060655	0,00534317	FAIM
ENSGALG00000011778	2303,47072	-0,415058611	0,12790911	-3,2449496	0,00117471	0,00931801	
ENSGALG00000013370	2831,43254	-0,415383566	0,10536475	-3,942339	8,07E-05	0,00095493	
ENSGALG00000014004	7899,02977	-0,415462509	0,09272364	-4,4806538	7,44E-06	0,00011394	
ENSGALG00000007304	5003,8857	-0,415655575	0,09074301	-4,5805797	4,64E-06	7,41E-05	
ENSGALG00000008152	3837,90866	-0,415829645	0,10117889	-4,1098461	3,96E-05	0,0005028	
ENSGALG00000002831	3577,75168	-0,41600984	0,10574326	-3,9341499	8,35E-05	0,0009825	SH3PXD2B
ENSGALG00000011238	44015,5054	-0,417063936	0,07670305	-5,4373842	5,41E-08	1,21E-06	WLS
ENSGALG00000027897	2595,29874	-0,417252062	0,11571548	-3,6058448	0,00031114	0,00306943	DTX1
ENSGALG00000006172	1892,0827	-0,41758713	0,11598879	-3,600237	0,00031793	0,00312163	ABCC8
ENSGALG00000011558	4354,80067	-0,418200744	0,11988928	-3,4882246	0,00048624	0,00442998	
ENSGALG00000021616	42268,4535	-0,419663237	0,0886351	-4,7347298	2,19E-06	3,72E-05	
ENSGALG00000008873	22196,3678	-0,419829297	0,09887727	-4,2459637	2,18E-05	0,00029594	POLE3
ENSGALG00000013002	2566,67672	-0,420522444	0,10532109	-3,9927659	6,53E-05	0,00078854	DAP1
ENSGALG00000000713	13614,4975	-0,421676085	0,08952353	-4,7102262	2,47E-06	4,15E-05	ZFHX3
ENSGALG00000002484	4308,16241	-0,423017499	0,1016587	-4,1611539	3,17E-05	0,00041259	CRYM
ENSGALG00000026085	4035,50855	-0,424635504	0,0949709	-4,4712169	7,78E-06	0,0001185	

ENSGALG00000005237	13631,0034	-0,426583983	0,07933189	-5,3772069	7,57E-08	1,64E-06	FGD3
ENSGALG00000000966	3098,3696	-0,42899963	0,11454974	-3,7450949	0,00018033	0,00190837	
ENSGALG00000003742	3986,83364	-0,429013967	0,10607378	-4,0444865	5,24E-05	0,00064817	CPAMD8
ENSGALG00000014903	2320,30966	-0,429814699	0,12033773	-3,5717369	0,00035462	0,00342281	ITGA2
ENSGALG00000010346	4336,77039	-0,430819834	0,09585982	-4,4942692	6,98E-06	0,00010807	TGFB3
ENSGALG00000003602	3515,61032	-0,430901727	0,11141807	-3,8674312	0,00010999	0,00125076	
ENSGALG00000005330	1520,22623	-0,431988153	0,12387209	-3,4873727	0,00048779	0,00444089	SNCAIP
ENSGALG00000010158	10233,5261	-0,433584905	0,07981872	-5,4321205	5,57E-08	1,25E-06	KANK1
ENSGALG00000016328	3476,01841	-0,434072431	0,12020068	-3,6112311	0,00030475	0,00301783	
ENSGALG00000026113	3962,92871	-0,434078932	0,09966992	-4,355165	1,33E-05	0,00019229	
ENSGALG00000017334	2609,2748	-0,435842486	0,12736757	-3,4219266	0,00062179	0,00545441	
ENSGALG00000009212	1797,44087	-0,438467006	0,1197104	-3,6627312	0,00024954	0,00253574	MND1
ENSGALG00000017321	1548,21324	-0,438859423	0,12937108	-3,3922529	0,0006932	0,00597493	PLEKHB1
ENSGALG00000000362	6059,70576	-0,440564998	0,08939063	-4,9285366	8,28E-07	1,53E-05	
ENSGALG00000015368	4121,06739	-0,441174377	0,09460983	-4,6630922	3,11E-06	5,11E-05	TMEM175
ENSGALG00000016200	8447,45569	-0,442373266	0,1201603	-3,6815259	0,00023184	0,00238124	
ENSGALG00000021285	5699,38195	-0,442446231	0,11153099	-3,967025	7,28E-05	0,00086948	
ENSGALG00000006950	4791,25164	-0,44318092	0,09921158	-4,467028	7,93E-06	0,00012055	LIMK2
ENSGALG00000009516	4808,75075	-0,444515061	0,09185634	-4,8392421	1,30E-06	2,32E-05	
ENSGALG00000013754	4623,32074	-0,444800112	0,10725118	-4,1472749	3,36E-05	0,00043614	
ENSGALG00000012158	18186,212	-0,44560696	0,08214388	-5,4247128	5,80E-08	1,29E-06	
ENSGALG00000008700	14557,9866	-0,447098587	0,09405696	-4,7534873	2,00E-06	3,42E-05	PDCD4
ENSGALG00000024120	15628,5446	-0,448054591	0,0899222	-4,9826915	6,27E-07	1,18E-05	
ENSGALG00000029174	2839,91825	-0,450699904	0,12705495	-3,5472832	0,00038923	0,00369972	
ENSGALG00000010971	1510,70666	-0,45100601	0,13003884	-3,4682407	0,00052388	0,00471113	CSRNP3
ENSGALG00000019987	7452,0282	-0,451061537	0,11200004	-4,0273338	5,64E-05	0,0006898	LRP11
ENSGALG00000000970	6965,54674	-0,451525139	0,09506462	-4,7496653	2,04E-06	3,48E-05	
ENSGALG00000008136	1284,16167	-0,451819148	0,13252856	-3,409221	0,00065149	0,0056751	C2orf69
ENSGALG00000007839	50053,4791	-0,453579178	0,09430846	-4,8095279	1,51E-06	2,66E-05	NCAM1
ENSGALG00000000628	9521,22366	-0,454003443	0,10552649	-4,3022698	1,69E-05	0,00023655	
ENSGALG00000025915	4915,79448	-0,454200002	0,12936551	-3,5109822	0,00044645	0,00413069	ARRDC3
ENSGALG00000015537	3136,68818	-0,459457432	0,12608507	-3,6440272	0,00026841	0,00269461	MAN2B2
ENSGALG00000006036	5865,93911	-0,461900797	0,11016505	-4,1928072	2,76E-05	0,00036394	
ENSGALG00000010705	6215,71804	-0,462215381	0,14311354	-3,2297111	0,00123915	0,00975492	ZBTB18
ENSGALG00000022926	4103,57352	-0,463163963	0,11247396	-4,117966	3,82E-05	0,00048789	GPR162
ENSGALG00000006982	2095,88213	-0,463202789	0,13888123	-3,335244	0,00085225	0,00715006	
ENSGALG00000009064	6911,57971	-0,463588885	0,0867529	-5,3437857	9,10E-08	1,93E-06	FZD1
ENSGALG00000000939	13136,3898	-0,464735788	0,11044249	-4,2079439	2,58E-05	0,00034257	
ENSGALG00000003595	2171,0558	-0,465858104	0,11751231	-3,9643346	7,36E-05	0,00087851	SARM1
ENSGALG00000017119	2363,66079	-0,467363832	0,11628448	-4,0191421	5,84E-05	0,00071214	TNFRSF19
ENSGALG00000004598	1493,7651	-0,468100334	0,1412582	-3,3137923	0,0009204	0,00761459	CUX2
ENSGALG00000009475	10491,2397	-0,469402079	0,09789746	-4,7948339	1,63E-06	2,85E-05	
ENSGALG00000006885	5604,36422	-0,469804206	0,10737161	-4,3754975	1,21E-05	0,00017684	
ENSGALG00000010422	4706,76498	-0,470203966	0,12428621	-3,7832354	0,0001548	0,00167222	
ENSGALG00000007710	2351,67196	-0,471105348	0,10711222	-4,3982409	1,09E-05	0,00016079	
ENSGALG00000027373	14650,434	-0,47216803	0,10227634	-4,616591	3,90E-06	6,33E-05	
ENSGALG00000001774	34212,4119	-0,47258081	0,07718637	-6,1225938	9,21E-10	2,65E-08	NEO1
ENSGALG00000015107	1553,32125	-0,4734568	0,1315676	-3,598582	0,00031996	0,00313663	PIP5K1B
ENSGALG00000012658	1659,97017	-0,475116315	0,12664668	-3,7515101	0,00017577	0,00186492	TCP11L2
ENSGALG00000015660	2274,63097	-0,475673447	0,12210103	-3,8957367	9,79E-05	0,00112766	SNX30
ENSGALG00000012586	1975,98196	-0,476656889	0,11252641	-4,2359557	2,28E-05	0,00030611	GKAP1
ENSGALG00000000316	1513,96938	-0,476826949	0,12199447	-3,9085948	9,28E-05	0,00107924	
ENSGALG00000024481	3771,0681	-0,479725051	0,11484828	-4,177033	2,95E-05	0,00038725	
ENSGALG00000003782	9195,14164	-0,482389155	0,1220329	-3,9529435	7,72E-05	0,00091791	
ENSGALG00000014537	1887,30146	-0,482456357	0,13681311	-3,5263898	0,00042127	0,00395321	BMF
ENSGALG00000029011	28886,6858	-0,483499319	0,07402631	-6,5314526	6,51E-11	2,18E-09	SLC35F1
ENSGALG00000015037	2876,08049	-0,483915865	0,10209562	-4,7398299	2,14E-06	3,64E-05	EVC
ENSGALG00000010316	6055,57077	-0,484012085	0,12348301	-3,9196654	8,87E-05	0,00103754	FRAS1
ENSGALG00000016518	2706,67897	-0,485066531	0,11148666	-4,3508932	1,36E-05	0,00019517	PHKA2
ENSGALG00000010818	5671,54414	-0,485799462	0,10196839	-4,7642161	1,90E-06	3,26E-05	PLPP3
ENSGALG00000009291	1052,37449	-0,489358793	0,13781708	-3,5507847	0,00038408	0,00366198	EPHX1
ENSGALG00000001966	2082,4586	-0,490465312	0,14000341	-3,5032383	0,00045964	0,00423064	

ENSGALG00000005806	2070,07142	-0,492123395	0,11517395	-4,2728706	1,93E-05	0,00026497	KIF24
ENSGALG00000016244	1547,30105	-0,492320823	0,12815771	-3,8415233	0,00012227	0,00136808	
ENSGALG00000001816	2143,75098	-0,495397442	0,135894	-3,6454696	0,0002669	0,00268384	MAGI3
ENSGALG00000020626	1193,83839	-0,496278462	0,14167036	-3,5030507	0,00045996	0,00423064	
ENSGALG00000013722	3288,15489	-0,496572265	0,12655366	-3,9238082	8,72E-05	0,00102087	SEMA3B
ENSGALG00000016289	12122,6528	-0,49658952	0,10671074	-4,653604	3,26E-06	5,33E-05	DST
ENSGALG00000011444	5389,01007	-0,497224463	0,11523481	-4,3148806	1,60E-05	0,00022558	MFHAS1
ENSGALG00000009207	2177,62925	-0,499904522	0,13219125	-3,7816762	0,00015578	0,00168128	TRIM2
ENSGALG00000012017	6540,80506	-0,499993655	0,08604614	-5,8107619	6,22E-09	1,57E-07	DAAM1
ENSGALG00000005680	1094,2433	-0,500648312	0,13778586	-3,6335245	0,00027958	0,00279777	
ENSGALG00000003099	3627,50167	-0,502434401	0,11543971	-4,3523531	1,35E-05	0,00019432	PCTP
ENSGALG00000003539	4597,6882	-0,503534691	0,0959582	-5,2474378	1,54E-07	3,16E-06	KIAA1522
ENSGALG00000026505	1253,87743	-0,503934058	0,14609798	-3,4492883	0,00056207	0,00500419	
ENSGALG00000008060	1648,89018	-0,504019022	0,12955696	-3,8903277	0,00010011	0,00114992	PRKAR2B
ENSGALG00000006859	2008,47317	-0,505092886	0,12844032	-3,9325104	8,41E-05	0,0009883	
ENSGALG00000010811	6795,76845	-0,506356689	0,1227478	-4,1251794	3,70E-05	0,00047479	LGMN
ENSGALG00000012124	3819,62883	-0,507526075	0,09613084	-5,2795346	1,30E-07	2,69E-06	PELI2
ENSGALG00000015786	1271,75753	-0,508635434	0,14394592	-3,5335176	0,00041007	0,00387136	REEP1
ENSGALG00000011083	1036,15097	-0,509062257	0,15598828	-3,2634648	0,00110059	0,00884785	
ENSGALG00000004338	30363,8557	-0,509560052	0,07503149	-6,791283	1,11E-11	4,07E-10	
ENSGALG00000002777	11306,9083	-0,509947463	0,08235673	-6,1919342	5,94E-10	1,77E-08	
ENSGALG00000005107	11201,627	-0,51021951	0,10373469	-4,9185041	8,72E-07	1,60E-05	
ENSGALG00000015152	10783,9679	-0,512951325	0,14885454	-3,4459905	0,00056897	0,00504594	BOC
ENSGALG00000025977	2371,64375	-0,513209442	0,10745001	-4,7762626	1,79E-06	3,10E-05	GADD45A
ENSGALG00000028635	2843,21769	-0,514353516	0,11810903	-4,3549042	1,33E-05	0,00019229	
ENSGALG00000008728	786,945495	-0,517913117	0,15751486	-3,2880271	0,00100892	0,00820052	PTER
ENSGALG00000000912	1664,29263	-0,519425471	0,11917413	-4,3585423	1,31E-05	0,00018956	
ENSGALG00000010835	74050,5322	-0,52138496	0,08594348	-6,0666028	1,31E-09	3,68E-08	DTX4
ENSGALG00000022713	3872,09413	-0,525946923	0,12443951	-4,2265269	2,37E-05	0,00031683	
ENSGALG00000027714	1668,20814	-0,526177638	0,12300172	-4,2778071	1,89E-05	0,00026004	ATG10
ENSGALG00000005246	13679,395	-0,527306483	0,1075472	-4,903024	9,44E-07	1,73E-05	
ENSGALG00000014069	15161,3096	-0,529241353	0,09850777	-5,3725847	7,76E-08	1,68E-06	TP53I11
ENSGALG00000015628	929,886545	-0,529404749	0,14733434	-3,5932203	0,00032662	0,0031969	MSANTD1
ENSGALG00000013182	11579,6316	-0,530382571	0,10696981	-4,9582454	7,11E-07	1,32E-05	
ENSGALG00000010982	4044,72703	-0,53129631	0,13536613	-3,9248836	8,68E-05	0,00101918	
ENSGALG00000015847	1982,57576	-0,531317329	0,11374907	-4,6709596	3,00E-06	4,93E-05	PRSS35
ENSGALG00000003601	11958,9068	-0,532351713	0,09422206	-5,6499692	1,60E-08	3,87E-07	ATP2B4
ENSGALG00000006480	1476,70794	-0,534658874	0,12638611	-4,230361	2,33E-05	0,00031279	TCF7
ENSGALG00000003185	3385,21843	-0,535546504	0,12368984	-4,3297532	1,49E-05	0,00021268	SCPEP1
ENSGALG00000011518	813,593359	-0,535965737	0,1518756	-3,5289785	0,00041717	0,00392357	POMGNT2
ENSGALG00000027002	29714,3795	-0,536298125	0,10571913	-5,0728579	3,92E-07	7,58E-06	
ENSGALG00000003648	1181,0691	-0,536868872	0,14374328	-3,7349146	0,00018778	0,00198222	SVIP
ENSGALG00000013211	1995,32702	-0,539056737	0,14233958	-3,7871176	0,0001524	0,00164916	Sial-T2
ENSGALG00000016576	12672,1252	-0,539357045	0,13054957	-4,1314348	3,61E-05	0,00046252	OFD1
ENSGALG00000013155	2327,87007	-0,539440335	0,12203608	-4,4203349	9,85E-06	0,00014675	
ENSGALG00000028015	4297,84833	-0,539854796	0,12735891	-4,238846	2,25E-05	0,0003031	
ENSGALG00000000449	5699,15535	-0,541504758	0,1320378	-4,1011343	4,11E-05	0,00052028	
ENSGALG00000011298	3487,55527	-0,543014986	0,11354366	-4,7824332	1,73E-06	3,01E-05	RARB
ENSGALG00000004270	3394,83221	-0,544775501	0,15590131	-3,4943614	0,0004752	0,00434521	ALDH1A2
ENSGALG00000007269	2258,18619	-0,545360128	0,11824854	-4,6119821	3,99E-06	6,43E-05	GABRA3
ENSGALG00000016551	3348,17917	-0,548003277	0,12821461	-4,2741096	1,92E-05	0,00026408	AP1S2
ENSGALG00000029102	8856,23983	-0,549378242	0,09139118	-6,0112829	1,84E-09	5,02E-08	PXYLP1
ENSGALG00000008433	2467,54155	-0,549541331	0,11423742	-4,8105196	1,51E-06	2,65E-05	
ENSGALG00000007245	695,960091	-0,550277699	0,16045688	-3,4294429	0,00060482	0,00533548	
ENSGALG00000013964	1040,16713	-0,55034782	0,13837128	-3,977327	6,97E-05	0,00083828	SPATA18
ENSGALG00000002552	868,094238	-0,551384108	0,15097485	-3,6521586	0,00026005	0,00262813	
ENSGALG00000012219	2204,64004	-0,554221866	0,12302026	-4,5051268	6,63E-06	0,00010372	UNC5C
ENSGALG00000004323	3093,09427	-0,5545441	0,12637337	-4,3881405	1,14E-05	0,00016765	RASAL2
ENSGALG00000015987	4635,85249	-0,554680878	0,12141534	-4,5684578	4,91E-06	7,84E-05	
ENSGALG00000008871	21521,8352	-0,555142332	0,09290105	-5,9756301	2,29E-09	6,13E-08	
ENSGALG00000004593	2415,84953	-0,555306466	0,163609	-3,3941071	0,00068853	0,00594806	SULF2
ENSGALG00000026757	7722,36602	-0,557063112	0,08452448	-6,5905534	4,38E-11	1,50E-09	

ENSGALG00000014242	1501,10358	-0,557782152	0,14125221	-3,9488383	7,85E-05	0,0009329	
ENSGALG00000012710	1128,84374	-0,557841328	0,15170545	-3,6771344	0,00023587	0,00241843	NCS1
ENSGALG00000004775	914,832175	-0,558344864	0,14503927	-3,8496118	0,00011831	0,00133083	
ENSGALG00000015593	2880,67783	-0,558901424	0,11770789	-4,7482072	2,05E-06	3,50E-05	EPHA7
ENSGALG00000004537	5159,7803	-0,559387674	0,08988409	-6,2234338	4,86E-10	1,48E-08	
ENSGALG00000017247	4207,7876	-0,562403241	0,09675844	-5,8124463	6,16E-09	1,56E-07	
ENSGALG00000000692	955,152266	-0,563167267	0,16964073	-3,3197645	0,00090093	0,00748326	CDK18
ENSGALG00000009799	18742,1488	-0,563286437	0,12204958	-4,6152265	3,93E-06	6,36E-05	
ENSGALG00000002375	32718,3264	-0,563646666	0,12800002	-4,403489	1,07E-05	0,00015732	NOTCH1
ENSGALG00000012487	1377,92439	-0,566700383	0,13846686	-4,0926789	4,26E-05	0,00053825	
ENSGALG00000028795	2245,71249	-0,566961474	0,16346113	-3,468479	0,00052341	0,00471035	
ENSGALG00000006583	5537,14066	-0,567576615	0,11179655	-5,0768704	3,84E-07	7,44E-06	LSP1
ENSGALG00000007007	719,343847	-0,57059825	0,16187065	-3,525026	0,00042344	0,00396469	
ENSGALG00000004741	4792,68775	-0,573167729	0,0909464	-6,3022583	2,93E-10	9,27E-09	EPHB2
ENSGALG00000004256	10783,2788	-0,575998874	0,08476767	-6,7950305	1,08E-11	3,97E-10	
ENSGALG00000004888	7031,61915	-0,578281859	0,0937448	-6,1686821	6,89E-10	2,03E-08	RAI1
ENSGALG00000004400	2735,32523	-0,578988451	0,11404307	-5,0769278	3,84E-07	7,44E-06	
ENSGALG00000013357	3130,31216	-0,580498969	0,15257333	-3,804721	0,00014196	0,00154959	
ENSGALG00000027173	1590,92759	-0,581363396	0,13148887	-4,4213887	9,81E-06	0,00014639	TMEM159
ENSGALG00000014485	4340,50749	-0,582732951	0,10497253	-5,5512902	2,84E-08	6,61E-07	LDB2
ENSGALG00000008034	2213,75476	-0,582849514	0,15579351	-3,7411669	0,00018317	0,00193681	CASKIN2
ENSGALG00000005396	1307,23993	-0,582889644	0,13145181	-4,4342459	9,24E-06	0,00013842	
ENSGALG00000011848	4663,28267	-0,584806197	0,10489037	-5,575404	2,47E-08	5,81E-07	
ENSGALG00000010940	1275,78523	-0,585237912	0,15386088	-3,8036823	0,00014256	0,00155339	ACSS3
ENSGALG00000003404	1593,24426	-0,585273519	0,17593109	-3,3267203	0,00087875	0,00732815	
ENSGALG00000002707	872,059957	-0,585668905	0,17399105	-3,3660864	0,00076243	0,00649674	CHRNB2
ENSGALG00000011657	4589,29906	-0,589843047	0,0912792	-6,4619656	1,03E-10	3,41E-09	EAF2
ENSGALG00000012748	903,483649	-0,592402199	0,17585259	-3,368743	0,00075512	0,00645203	ELOVL2
ENSGALG00000023279	941,141249	-0,593060121	0,14801399	-4,0067841	6,16E-05	0,00074752	EPN3
ENSGALG00000008961	48076,4841	-0,593936394	0,13546511	-4,3844233	1,16E-05	0,00017034	
ENSGALG00000006409	15248,7839	-0,594856883	0,11200977	-5,310759	1,09E-07	2,29E-06	
ENSGALG00000001916	3626,02385	-0,595193183	0,09718325	-6,124442	9,10E-10	2,63E-08	SLIT3
ENSGALG00000020554	873,190798	-0,595564073	0,16838642	-3,5368889	0,00040487	0,00382516	
ENSGALG00000015903	1387,24274	-0,597526236	0,13866253	-4,309212	1,64E-05	0,00022992	
ENSGALG00000002116	700,369856	-0,598531501	0,16175305	-3,7002796	0,00021536	0,00223751	TEN1
ENSGALG00000005036	1758,38822	-0,599441683	0,16374911	-3,6607325	0,0002515	0,0025494	SH2D3C
ENSGALG00000026180	3833,45288	-0,59981586	0,14976744	-4,0049816	6,20E-05	0,00075251	
ENSGALG00000012346	1154,67986	-0,599840638	0,13375751	-4,4845379	7,31E-06	0,00011243	
ENSGALG00000000927	2190,2949	-0,600422638	0,14772586	-4,0644382	4,81E-05	0,00060289	ANGPTL2
ENSGALG00000000644	7209,00394	-0,60054163	0,12270703	-4,8941093	9,88E-07	1,80E-05	
ENSGALG00000011350	541,285072	-0,601290146	0,17331192	-3,4694103	0,0005216	0,00469742	NEGR1
ENSGALG00000015661	1332,78697	-0,601504233	0,14287821	-4,2099089	2,55E-05	0,00034033	
ENSGALG00000010960	1489,43056	-0,603326229	0,15158134	-3,9802144	6,89E-05	0,00082975	GALNT3
ENSGALG00000007993	1260,65804	-0,603453342	0,13715554	-4,3997738	1,08E-05	0,00015985	DCX
ENSGALG00000003973	3817,71731	-0,603622901	0,09870926	-6,1151598	9,65E-10	2,75E-08	TLCD1
ENSGALG00000006942	9609,38746	-0,606230537	0,07966615	-7,6096378	2,75E-14	1,40E-12	
ENSGALG00000012697	695,072503	-0,6064194	0,18664528	-3,2490476	0,00115792	0,00920562	SLC41A2
ENSGALG00000026941	1374,3273	-0,606678305	0,14040651	-4,3208702	1,55E-05	0,00022017	
ENSGALG00000012712	1815,03517	-0,607252424	0,12916444	-4,70139	2,58E-06	4,32E-05	RBM24
ENSGALG00000015774	1065,02106	-0,60746392	0,15041346	-4,0386273	5,38E-05	0,0006613	CYYR1
ENSGALG00000015542	9498,31771	-0,607580543	0,12993075	-4,6761873	2,92E-06	4,82E-05	PLPPR1
ENSGALG00000009537	490,58222	-0,607598218	0,18032255	-3,3695076	0,00075303	0,00643855	PPM1L
ENSGALG00000000623	23029,3222	-0,609505879	0,08219995	-7,4149182	1,22E-13	5,72E-12	
ENSGALG00000003375	5633,57059	-0,611230825	0,08908432	-6,861262	6,83E-12	2,59E-10	
ENSGALG00000014627	944,560693	-0,611235613	0,15029669	-4,0668602	4,77E-05	0,00059726	
ENSGALG00000016251	5263,27603	-0,611728855	0,14715775	-4,1569598	3,23E-05	0,0004198	
ENSGALG00000002974	1437,50977	-0,611833543	0,15307455	-3,9969645	6,42E-05	0,00077544	SPHKAP
ENSGALG00000027240	7653,65786	-0,611869645	0,14969349	-4,0874834	4,36E-05	0,00054823	TSPAN13
ENSGALG00000002155	2491,16451	-0,612943427	0,15977684	-3,8362469	0,00012493	0,00139281	
ENSGALG00000008367	12046,292	-0,614602858	0,10352854	-5,9365551	2,91E-09	7,68E-08	MDK
ENSGALG00000003163	4932,62175	-0,61460754	0,11759786	-5,2263499	1,73E-07	3,51E-06	GNAO1
ENSGALG00000010551	740,499781	-0,614933753	0,17383652	-3,5374256	0,00040405	0,00382136	CDC42EP3

ENSGALG00000027183	953,592441	-0,617367034	0,15820971	-3,9022069	9,53E-05	0,00110199	
ENSGALG00000029070	932,579973	-0,617616574	0,16266457	-3,7968722	0,00014653	0,00159251	FAM181A
ENSGALG00000011798	1064,51312	-0,617821943	0,17164092	-3,5995026	0,00031883	0,003128	HEBP1
ENSGALG00000002652	11400,8043	-0,621511478	0,12728765	-4,8827319	1,05E-06	1,89E-05	
ENSGALG00000013505	3208,27244	-0,621565495	0,14984432	-4,1480752	3,35E-05	0,0004354	
ENSGALG00000003582	10849,7231	-0,622442826	0,14351033	-4,3372686	1,44E-05	0,00020648	SLC1A3
ENSGALG00000008555	1679,68251	-0,624562442	0,15429616	-4,0478159	5,17E-05	0,00064028	MXI1
ENSGALG00000007076	2388,80102	-0,624816157	0,15466095	-4,0399089	5,35E-05	0,000659	CCNY
ENSGALG00000016391	3109,4295	-0,625110592	0,10416619	-6,0010894	1,96E-09	5,30E-08	CNKSR2
ENSGALG00000027377	1563,60517	-0,626128444	0,12409736	-5,0454616	4,52E-07	8,68E-06	
ENSGALG00000008774	10001,1995	-0,627375234	0,09280246	-6,7603298	1,38E-11	4,99E-10	
ENSGALG00000000584	815,161004	-0,628246913	0,15550344	-4,0400836	5,34E-05	0,000659	
ENSGALG00000005403	6166,93158	-0,628470531	0,10727764	-5,8583552	4,67E-09	1,21E-07	DHRS11
ENSGALG00000007819	1168,66551	-0,629150395	0,15548421	-4,0463942	5,20E-05	0,00064355	PDZRN3
ENSGALG00000015799	696,846524	-0,630241657	0,16107768	-3,9126566	9,13E-05	0,00106223	ELAVL2
ENSGALG00000015729	1257,59964	-0,630253804	0,13571537	-4,6439383	3,42E-06	5,58E-05	LPAR1
ENSGALG00000007892	2832,91388	-0,631636449	0,10556148	-5,9835882	2,18E-09	5,85E-08	SPAG6
ENSGALG00000007748	2144,94025	-0,63248212	0,11245141	-5,6244928	1,86E-08	4,44E-07	SLC16A2
ENSGALG00000000040	635,110846	-0,633128633	0,18365469	-3,4473862	0,00056604	0,00503123	
ENSGALG00000002897	1585,0892	-0,633730718	0,13210035	-4,7973432	1,61E-06	2,82E-05	SLC16A14
ENSGALG00000010320	1455,65862	-0,636363776	0,13429986	-4,7383799	2,15E-06	3,66E-05	PAWR
ENSGALG00000006152	1112,15045	-0,63795304	0,16197274	-3,9386446	8,19E-05	0,0009661	
ENSGALG00000002437	563,695653	-0,638363537	0,17402082	-3,668317	0,00024415	0,00249111	FAM69B
ENSGALG00000010269	920,840765	-0,639329258	0,17097965	-3,7392125	0,0001846	0,00195028	KCNAB1
ENSGALG00000001153	10543,8081	-0,64025058	0,09353164	-6,8452831	7,63E-12	2,88E-10	AUTS2
ENSGALG00000006826	5812,76792	-0,641184602	0,13004063	-4,9306482	8,20E-07	1,51E-05	KLHL25
ENSGALG00000028543	1233,57546	-0,64250805	0,17971356	-3,5751785	0,00034999	0,00339345	FGFR4
ENSGALG00000026460	5792,76927	-0,643922736	0,09174473	-7,018635	2,24E-12	9,13E-11	
ENSGALG00000012577	1370,05679	-0,644333952	0,12936964	-4,9805656	6,34E-07	1,19E-05	FRMD3
ENSGALG00000001516	2392,04392	-0,648414359	0,13283038	-4,8815216	1,05E-06	1,90E-05	
ENSGALG00000015241	2158,94253	-0,649652118	0,12094277	-5,3715665	7,81E-08	1,68E-06	
ENSGALG00000017036	2072,67923	-0,649834759	0,14869137	-4,3703596	1,24E-05	0,00018042	LHFP
ENSGALG00000000750	998,349389	-0,65019981	0,16954656	-3,8349336	0,0001256	0,00139654	
ENSGALG00000014508	1028,74107	-0,651699626	0,19490886	-3,3436121	0,00082695	0,00698	CD38
ENSGALG00000006490	2371,83217	-0,651963707	0,12330582	-5,2873719	1,24E-07	2,59E-06	SCN3B
ENSGALG00000002125	658,342298	-0,653074319	0,17067212	-3,826485	0,00012999	0,00143893	TMOD1
ENSGALG00000028043	7943,94691	-0,65417089	0,08543968	-7,6565229	1,91E-14	1,00E-12	
ENSGALG00000002840	4314,5965	-0,659899714	0,10957661	-6,0222678	1,72E-09	4,71E-08	GRM4
ENSGALG00000005886	3052,74002	-0,660958059	0,12281861	-5,3815792	7,38E-08	1,60E-06	WSCD1
ENSGALG00000029072	3177,58522	-0,662820707	0,15123881	-4,3826099	1,17E-05	0,00017156	NTN3
ENSGALG00000009439	566,603521	-0,662995235	0,19541313	-3,3927875	0,00069185	0,00596854	GPR85
ENSGALG00000002712	9163,53899	-0,663245926	0,11304776	-5,8669534	4,44E-09	1,15E-07	
ENSGALG00000005628	1530,5988	-0,664092027	0,18442306	-3,6009165	0,0003171	0,00311592	COL9A3
ENSGALG00000005657	1315,51328	-0,664586696	0,19971819	-3,3276222	0,00087591	0,00731033	CRHR2
ENSGALG00000011592	769,411267	-0,666316729	0,16592744	-4,0157116	5,93E-05	0,00072188	MRAS
ENSGALG00000007732	416,813973	-0,671216647	0,19001649	-3,5324126	0,00041179	0,00388171	RTN4R
ENSGALG00000011019	3781,75966	-0,672110507	0,11796056	-5,697756	1,21E-08	2,97E-07	ROR1
ENSGALG00000016100	441,759488	-0,672712737	0,2029277	-3,3150365	0,00091631	0,0075858	
ENSGALG00000010775	2089,69413	-0,672804554	0,12893781	-5,2180549	1,81E-07	3,65E-06	
ENSGALG00000010119	1066,14976	-0,672877793	0,15931928	-4,2234548	2,41E-05	0,00032084	CDKL2
ENSGALG00000026055	2148,9591	-0,673106345	0,1590473	-4,2321142	2,32E-05	0,00031072	PALM
ENSGALG00000004309	779,594502	-0,673847887	0,18081676	-3,7266892	0,00019401	0,00203427	GDAP1L1
ENSGALG00000005774	3489,02871	-0,674007323	0,11106476	-6,0685974	1,29E-09	3,64E-08	
ENSGALG00000017106	466,641421	-0,674065701	0,1927802	-3,4965505	0,00047132	0,00431602	ATP8A2
ENSGALG00000016945	2967,84555	-0,674331354	0,14913858	-4,5215085	6,14E-06	9,66E-05	LECT1
ENSGALG00000012575	8218,93791	-0,674390391	0,10186247	-6,6205974	3,58E-11	1,23E-09	TLE1
ENSGALG00000026704	817,227006	-0,675659204	0,17070288	-3,9581008	7,55E-05	0,00090089	GPX8
ENSGALG00000007719	1302,68364	-0,67764292	0,20270636	-3,3429781	0,00082884	0,00699124	
ENSGALG00000006555	2117,21497	-0,681158051	0,13379771	-5,0909546	3,56E-07	6,96E-06	
ENSGALG00000016575	9168,66303	-0,686420093	0,1297794	-5,2891299	1,23E-07	2,57E-06	GPM6B
ENSGALG00000000151	1937,7168	-0,687891713	0,11424333	-6,0212854	1,73E-09	4,73E-08	ADAMTS19
ENSGALG00000001727	463,318695	-0,689838866	0,18509677	-3,7269093	0,00019384	0,0020342	LZTS1

ENSGALG00000005426	877,605348	-0,690145659	0,15508752	-4,45004	8,59E-06	0,00012955	FREM1
ENSGALG00000003550	652,802151	-0,692363086	0,17977735	-3,8512254	0,00011753	0,00132447	ARHGAP40
ENSGALG00000023073	20456,9956	-0,692447843	0,08514089	-8,1329649	4,19E-16	2,52E-14	
ENSGALG00000007364	1353,3018	-0,692880741	0,18051262	-3,8384061	0,00012384	0,001384	TOB1
ENSGALG00000009626	8049,48001	-0,694120539	0,10537026	-6,5874428	4,47E-11	1,53E-09	THBS1
ENSGALG00000012620	28578,6606	-0,694383357	0,081059	-8,566394	1,07E-17	7,34E-16	PTCH1
ENSGALG00000003580	1352,19249	-0,694931491	0,15746973	-4,4131115	1,02E-05	0,00015102	MMP2
ENSGALG00000027793	607,089392	-0,696507844	0,18113408	-3,8452612	0,00012042	0,00134981	SCN9A
ENSGALG00000010903	2596,79645	-0,69779548	0,11340379	-6,1531935	7,59E-10	2,22E-08	
ENSGALG00000002818	12036,7414	-0,699438203	0,11243999	-6,2205469	4,95E-10	1,50E-08	
ENSGALG00000003589	5291,70331	-0,699453771	0,09356739	-7,4754011	7,70E-14	3,70E-12	VTN
ENSGALG00000018624	844,593067	-0,700079474	0,18303859	-3,8247644	0,0001309	0,00144774	
ENSGALG00000006882	2236,27031	-0,700351611	0,11195432	-6,255691	3,96E-10	1,22E-08	ST8SIA2
ENSGALG00000002952	1456,76104	-0,700899465	0,13291552	-5,2732701	1,34E-07	2,78E-06	NRG4
ENSGALG00000012226	644,458201	-0,707828644	0,18746681	-3,7757545	0,00015952	0,00171729	
ENSGALG00000012775	643,488801	-0,709215839	0,20574547	-3,4470545	0,00056673	0,00503143	TFAP2A
ENSGALG00000004676	1415,38407	-0,710261932	0,14188675	-5,0058368	5,56E-07	1,05E-05	
ENSGALG00000029140	9887,31246	-0,710672238	0,11105305	-6,3993944	1,56E-10	5,08E-09	CRABP2
ENSGALG00000017405	1348,63296	-0,713912912	0,17423648	-4,0973791	4,18E-05	0,00052797	NPR3
ENSGALG00000015926	932,376679	-0,714450321	0,16657966	-4,2889409	1,80E-05	0,00024869	
ENSGALG00000019719	1763,03906	-0,714724163	0,18211375	-3,924603	8,69E-05	0,00101926	KRT19
ENSGALG00000025919	2203,75306	-0,717706563	0,17787583	-4,0348739	5,46E-05	0,00066999	
ENSGALG00000024486	1640,6708	-0,717823268	0,14268972	-5,0306586	4,89E-07	9,31E-06	
ENSGALG00000002016	716,489518	-0,719060037	0,164965	-4,3588642	1,31E-05	0,0001895	PRKCD
ENSGALG00000000674	402,036081	-0,719912044	0,20464736	-3,5178175	0,00043511	0,00405273	MYO1D
ENSGALG00000005608	3947,85993	-0,721159959	0,11459976	-6,2928576	3,12E-10	9,80E-09	SLC9A3R2
ENSGALG00000027198	9040,88356	-0,721260873	0,09723673	-7,4175766	1,19E-13	5,63E-12	TMEM47
ENSGALG00000011823	11238,0507	-0,721999514	0,10012518	-7,2109681	5,56E-13	2,43E-11	FAT4
ENSGALG00000003473	17669,5597	-0,722769839	0,13594288	-5,3167173	1,06E-07	2,23E-06	SFRP1
ENSGALG00000009248	4501,54609	-0,722897324	0,13466182	-5,3682427	7,95E-08	1,71E-06	HSPA12A
ENSGALG00000002749	1465,51039	-0,723137568	0,13433788	-5,3829757	7,33E-08	1,59E-06	
ENSGALG00000001449	3438,32151	-0,723393814	0,10318556	-7,0106112	2,37E-12	9,57E-11	STRA6
ENSGALG00000006792	350,301605	-0,726229187	0,22053524	-3,2930301	0,00099114	0,00807697	VSTM2L
ENSGALG00000011712	1179,53872	-0,727241211	0,14005331	-5,1926027	2,07E-07	4,14E-06	
ENSGALG00000012094	284,066214	-0,727903351	0,22026176	-3,3047196	0,00095071	0,00780859	SLC35F4
ENSGALG00000003894	796,557275	-0,728609826	0,15200269	-4,7934008	1,64E-06	2,86E-05	
ENSGALG00000027907	6182,31359	-0,731289025	0,08932741	-8,1866136	2,69E-16	1,66E-14	NR2F1
ENSGALG00000029004	2171,38417	-0,731300396	0,17746098	-4,120908	3,77E-05	0,00048269	
ENSGALG00000015597	952,530813	-0,731477034	0,1532441	-4,7732801	1,81E-06	3,13E-05	
ENSGALG00000015673	6092,107	-0,738858521	0,12050003	-6,1316045	8,70E-10	2,52E-08	
ENSGALG00000026858	6446,94204	-0,741127309	0,11137264	-6,6544825	2,84E-11	9,91E-10	BTG2
ENSGALG00000009020	13288,8425	-0,742019908	0,08130962	-9,125856	7,12E-20	5,90E-18	JAG1
ENSGALG00000005319	564,975279	-0,742306515	0,19352205	-3,8357723	0,00012517	0,00139302	CDH8
ENSGALG00000006211	4538,06281	-0,742344985	0,0986063	-7,5283727	5,14E-14	2,54E-12	BCL2L1
ENSGALG00000009536	396,027671	-0,742471798	0,20594764	-3,6051484	0,00031197	0,00307525	
ENSGALG00000000044	1571,14006	-0,742898149	0,12135747	-6,1215694	9,27E-10	2,66E-08	WFIKKN1
ENSGALG00000026119	1205,64066	-0,744514312	0,18875953	-3,9442475	8,01E-05	0,00094826	
ENSGALG00000002645	1167,98665	-0,746278475	0,19471396	-3,8326911	0,00012675	0,00140434	
ENSGALG00000011655	1680,80637	-0,746810997	0,13582015	-5,4985288	3,83E-08	8,71E-07	IQCB1
ENSGALG00000015075	456,601258	-0,751299073	0,202365	-3,7125939	0,00020515	0,00214026	
ENSGALG00000012440	13707,6458	-0,752102714	0,10042226	-7,4894023	6,92E-14	3,35E-12	
ENSGALG00000004370	1173,97528	-0,752418484	0,16274623	-4,6232621	3,78E-06	6,14E-05	MAP2K6
ENSGALG00000009048	718,531943	-0,75410293	0,17428775	-4,3267695	1,51E-05	0,00021509	ABLIM1
ENSGALG00000026179	302,231909	-0,755339606	0,23357232	-3,2338576	0,0012213	0,00962652	FAM163B
ENSGALG00000015448	3790,52276	-0,755416575	0,16376879	-4,6127018	3,97E-06	6,42E-05	
ENSGALG00000005626	527,341226	-0,756044449	0,20533084	-3,6820793	0,00023134	0,00237979	TPST2
ENSGALG00000011182	9658,13673	-0,756680805	0,13954289	-5,4225681	5,87E-08	1,30E-06	DLL1
ENSGALG00000000690	8376,38333	-0,756893418	0,08326065	-9,0906497	9,84E-20	8,05E-18	GDPD4
ENSGALG00000015768	2395,79768	-0,757420183	0,12945293	-5,8509311	4,89E-09	1,25E-07	
ENSGALG00000006793	352,610169	-0,758190034	0,20784148	-3,6479245	0,00026437	0,00266691	
ENSGALG00000009959	1998,29964	-0,759714189	0,12312472	-6,1702815	6,82E-10	2,02E-08	
ENSGALG00000006715	4235,48084	-0,760061887	0,0985729	-7,7106575	1,25E-14	6,63E-13	OAF

ENSGALG00000003434	13153,9326	-0,761134193	0,10176159	-7,4795823	7,46E-14	3,60E-12	C17orf75
ENSGALG00000003837	1171,25005	-0,761557993	0,14071896	-5,4119075	6,24E-08	1,37E-06	ADCY7
ENSGALG00000002909	7457,53402	-0,762624106	0,09533386	-7,999509	1,25E-15	7,23E-14	
ENSGALG00000003193	8812,21661	-0,764114517	0,14621053	-5,2261251	1,73E-07	3,51E-06	CRABP-I
ENSGALG00000002742	2459,13281	-0,7644405	0,11429203	-6,6884845	2,25E-11	7,99E-10	TMEM132B
ENSGALG00000015297	1133,52891	-0,764510224	0,13956384	-5,477853	4,31E-08	9,76E-07	FOXO3
ENSGALG00000016543	3109,19527	-0,766325509	0,11889026	-6,4456544	1,15E-10	3,78E-09	NHS
ENSGALG00000006192	500,889627	-0,767192565	0,19007181	-4,0363301	5,43E-05	0,0006665	
ENSGALG00000015399	380,128404	-0,76931559	0,20396936	-3,7717213	0,00016213	0,00174157	FUT10
ENSGALG00000011742	234,220676	-0,769619243	0,23242839	-3,3112102	0,00092893	0,00766493	ART4
ENSGALG00000009732	5423,65869	-0,771536411	0,14233295	-5,420645	5,94E-08	1,31E-06	PCDH18
ENSGALG00000013194	2884,73548	-0,772416578	0,1120601	-6,8928778	5,47E-12	2,10E-10	
ENSGALG00000004331	987,414427	-0,775822966	0,14440744	-5,3724583	7,77E-08	1,68E-06	
ENSGALG00000012595	1196,34853	-0,775902432	0,16489518	-4,7054283	2,53E-06	4,24E-05	AGTPBP1
ENSGALG00000007623	3044,29553	-0,778310378	0,11922769	-6,5279333	6,67E-11	2,22E-09	CACNA1G
ENSGALG00000014853	603,727095	-0,778595081	0,1836687	-4,2391277	2,24E-05	0,0003031	CLVS2
ENSGALG00000026042	740,41298	-0,780763307	0,23587999	-3,3100024	0,00093295	0,00768428	
ENSGALG00000006341	275,593591	-0,786972369	0,23377109	-3,366423	0,0007615	0,00649324	
ENSGALG00000012191	7238,91303	-0,787037882	0,10851003	-7,2531352	4,07E-13	1,83E-11	
ENSGALG00000021593	580,680268	-0,787646676	0,19140603	-4,1150567	3,87E-05	0,00049258	
ENSGALG00000004028	313,965547	-0,787784238	0,22915888	-3,4377208	0,00058663	0,00519701	MATN4
ENSGALG00000010854	1552,15616	-0,790623838	0,14163311	-5,5821962	2,38E-08	5,61E-07	HDAC9
ENSGALG00000011487	7037,53698	-0,797547344	0,08867908	-8,9936355	2,39E-19	1,85E-17	
ENSGALG00000015476	710,998877	-0,797713032	0,19508358	-4,0890835	4,33E-05	0,00054556	
ENSGALG00000005892	22205,1699	-0,805793553	0,08367702	-9,6298065	5,98E-22	5,94E-20	
ENSGALG00000012652	512,848116	-0,806982417	0,18892568	-4,2714278	1,94E-05	0,0002664	
ENSGALG00000016223	637,815955	-0,80722926	0,16999597	-4,7485201	2,05E-06	3,50E-05	
ENSGALG00000004844	6351,78047	-0,807613149	0,13771449	-5,8644021	4,51E-09	1,17E-07	
ENSGALG00000007929	5051,2907	-0,807992906	0,1300334	-6,2137338	5,17E-10	1,56E-08	DIXDC1
ENSGALG00000002228	3137,43096	-0,808972118	0,16544265	-4,8897435	1,01E-06	1,83E-05	
ENSGALG00000004986	20927,5453	-0,810825031	0,09474777	-8,5577211	1,15E-17	7,87E-16	CERCAM
ENSGALG00000027498	3868,25329	-0,813503322	0,13291213	-6,1206102	9,32E-10	2,67E-08	FOXB1
ENSGALG00000004472	11062,9838	-0,814406115	0,10696645	-7,6136592	2,66E-14	1,37E-12	ASTN1
ENSGALG00000010055	752,36105	-0,814715149	0,17382196	-4,6870669	2,77E-06	4,60E-05	DAAM2
ENSGALG00000017374	378,439025	-0,814717413	0,2081861	-3,9134092	9,10E-05	0,00106189	
ENSGALG00000005903	1351,52	-0,81577863	0,16378148	-4,9808968	6,33E-07	1,19E-05	NRIP3
ENSGALG00000009514	9638,41366	-0,816414808	0,09378509	-8,7051667	3,17E-18	2,24E-16	NRCAM
ENSGALG00000013297	1134,91265	-0,820011934	0,15144755	-5,4144946	6,15E-08	1,35E-06	BBOX1
ENSGALG00000012092	334,595506	-0,821365542	0,23392101	-3,5112944	0,00044593	0,0041289	ESYT3
ENSGALG00000017332	676,273003	-0,827504109	0,22658295	-3,6521022	0,0002601	0,00262813	KLHL35
ENSGALG00000027300	4224,617	-0,83359345	0,12039523	-6,9238082	4,40E-12	1,70E-10	
ENSGALG00000026388	192,213198	-0,83565579	0,23979309	-3,4849036	0,00049231	0,00447555	
ENSGALG00000011206	1241,95142	-0,836880661	0,13265101	-6,30889	2,81E-10	8,90E-09	KITLG
ENSGALG00000002911	3538,18445	-0,840005591	0,1545939	-5,4336271	5,52E-08	1,24E-06	MOXD1
ENSGALG00000012715	336,22161	-0,842621875	0,2238429	-3,7643449	0,00016699	0,00178533	CAP2
ENSGALG00000022967	514,043952	-0,84515578	0,20455601	-4,1316596	3,60E-05	0,00046252	
ENSGALG00000011117	340,372899	-0,850337952	0,2419193	-3,5149653	0,00043981	0,00408392	AGT
ENSGALG00000003424	1326,91239	-0,851626485	0,15683659	-5,4300242	5,63E-08	1,26E-06	LMX1A
ENSGALG00000015084	885,260142	-0,852219609	0,20558018	-4,1454367	3,39E-05	0,00043874	IGSF11
ENSGALG00000023359	636,48694	-0,852612547	0,1928659	-4,4207533	9,84E-06	0,00014665	BTBD17
ENSGALG00000006873	2180,73864	-0,854385087	0,11802019	-7,2393131	4,51E-13	2,01E-11	PHRF1
ENSGALG00000017195	132,512378	-0,858697871	0,2579367	-3,3291031	0,00087126	0,0072803	PGR
ENSGALG00000009052	2878,13312	-0,861077306	0,16724187	-5,1486945	2,62E-07	5,19E-06	LBH
ENSGALG00000026192	3872,6926	-0,861152656	0,11660797	-7,3850243	1,52E-13	7,11E-12	ST8SIA4
ENSGALG00000026214	847,888186	-0,867136808	0,18566435	-4,6704539	3,01E-06	4,94E-05	LRTM2
ENSGALG00000028946	302,158738	-0,867278013	0,21980251	-3,9457147	7,96E-05	0,00094425	
ENSGALG00000009478	940,161846	-0,868396515	0,21374409	-4,0627861	4,85E-05	0,00060596	
ENSGALG00000006662	604,284175	-0,86909427	0,21853019	-3,9769986	6,98E-05	0,00083863	
ENSGALG00000027484	107,369685	-0,870548896	0,26698629	-3,2606502	0,00111157	0,00890653	C20orf85
ENSGALG00000021031	6097,64928	-0,871546666	0,10804236	-8,0667127	7,22E-16	4,30E-14	ACKR3
ENSGALG00000008297	5581,87806	-0,875877387	0,0888238	-9,8608413	6,15E-23	6,64E-21	SEMA4B
ENSGALG00000011709	491,428075	-0,876476185	0,23285575	-3,7640307	0,0001672	0,00178605	

ENSGALG00000003345	838,196489	-0,878084006	0,15479837	-5,6724371	1,41E-08	3,43E-07	
ENSGALG00000023738	720,292835	-0,882263773	0,18476554	-4,7750451	1,80E-06	3,11E-05	
ENSGALG00000007441	1451,19846	-0,88468627	0,1740619	-5,0825959	3,72E-07	7,24E-06	
ENSGALG00000027501	978,829878	-0,88631831	0,14664686	-6,0438954	1,50E-09	4,17E-08	ANPEP
ENSGALG00000004527	511,661724	-0,887058508	0,20448368	-4,3380405	1,44E-05	0,00020599	
ENSGALG00000006235	1550,13485	-0,888605951	0,18068598	-4,9179574	8,75E-07	1,61E-05	
ENSGALG00000003426	2655,93043	-0,891859274	0,1255624	-7,1029166	1,22E-12	5,14E-11	RHBDL3
ENSGALG00000028371	2318,19897	-0,893107554	0,18274531	-4,8871708	1,02E-06	1,85E-05	
ENSGALG00000012006	1652,91118	-0,89358513	0,13601021	-6,5699857	5,03E-11	1,70E-09	RTN1
ENSGALG00000028931	2326,56897	-0,894112314	0,12608288	-7,091465	1,33E-12	5,53E-11	
ENSGALG00000001129	1687,21268	-0,899923251	0,15466843	-5,818403	5,94E-09	1,51E-07	
ENSGALG00000000678	793,449754	-0,899943985	0,22197436	-4,0542701	5,03E-05	0,00062534	CITED4
ENSGALG00000015186	836,758425	-0,900331794	0,15959516	-5,6413479	1,69E-08	4,04E-07	
ENSGALG00000006245	281,138415	-0,900397348	0,25179693	-3,5758869	0,00034904	0,00338989	
ENSGALG00000029057	576,095316	-0,907467539	0,18536984	-4,8954433	9,81E-07	1,79E-05	
ENSGALG00000009603	326,307151	-0,909672796	0,25065366	-3,6292021	0,0002843	0,00283596	
ENSGALG00000017074	110,235407	-0,912794343	0,27273176	-3,3468575	0,00081733	0,00691746	
ENSGALG00000028234	573,481229	-0,912907561	0,22530534	-4,0518682	5,08E-05	0,00063117	
ENSGALG00000015967	195,046703	-0,91657646	0,23708171	-3,8660784	0,0001106	0,00125543	LRRTM1
ENSGALG00000004530	1049,1338	-0,916608336	0,21450742	-4,2730846	1,93E-05	0,00026497	MMD2
ENSGALG00000028790	633,225912	-0,919355636	0,19021109	-4,833344	1,34E-06	2,38E-05	DNASE2B
ENSGALG00000020688	639,432719	-0,921010154	0,21245986	-4,3349843	1,46E-05	0,00020792	
ENSGALG00000020376	2296,58151	-0,925271568	0,10950523	-8,449565	2,92E-17	1,90E-15	
ENSGALG00000007078	118,265561	-0,925877896	0,27021704	-3,426423	0,00061159	0,00537621	
ENSGALG00000005539	516,027592	-0,927364348	0,19120632	-4,8500715	1,23E-06	2,21E-05	
ENSGALG00000006346	1363,78378	-0,927790987	0,17515018	-5,2971169	1,18E-07	2,47E-06	CXCL14
ENSGALG00000027154	1132,69645	-0,928050292	0,17313065	-5,3604045	8,30E-08	1,77E-06	
ENSGALG00000029106	1760,23678	-0,929278314	0,13137058	-7,0737171	1,51E-12	6,27E-11	
ENSGALG00000012747	3481,16686	-0,930889427	0,16898793	-5,508615	3,62E-08	8,30E-07	NEDD9
ENSGALG00000020575	478,814354	-0,930944308	0,20604338	-4,5181957	6,24E-06	9,79E-05	
ENSGALG00000028745	260,770335	-0,940548991	0,23003741	-4,0886784	4,34E-05	0,00054596	
ENSGALG00000009237	245,620907	-0,942598283	0,22912432	-4,1139163	3,89E-05	0,00049452	
ENSGALG00000009042	1781,95784	-0,948736093	0,13944347	-6,8037326	1,02E-11	3,75E-10	ISM1
ENSGALG00000026622	538,54299	-0,952490076	0,18017774	-5,2863915	1,25E-07	2,60E-06	
ENSGALG00000004849	4449,83906	-0,955100006	0,11584173	-8,2448699	1,65E-16	1,03E-14	WSCD2
ENSGALG00000000280	585,892203	-0,956447554	0,17580335	-5,4404398	5,31E-08	1,20E-06	EFNA5
ENSGALG00000027514	44,3005488	-0,958727122	0,26964144	-3,555563	0,00037717	0,00360705	
ENSGALG00000011200	585,083893	-0,962880109	0,21843281	-4,4081295	1,04E-05	0,00015417	THBS2
ENSGALG00000001063	2024,22024	-0,96616322	0,18451124	-5,2363381	1,64E-07	3,34E-06	
ENSGALG00000006507	1219,79935	-0,967854239	0,13223106	-7,3194168	2,49E-13	1,13E-11	CLMP
ENSGALG00000004790	24425,8053	-0,971232729	0,24050391	-4,0383241	5,38E-05	0,00066151	
ENSGALG00000028005	1938,07994	-0,972644101	0,16792696	-5,7920664	6,95E-09	1,74E-07	GADD45G
ENSGALG00000016459	278,752189	-0,980053688	0,21673389	-4,5219218	6,13E-06	9,65E-05	FAM84A
ENSGALG00000016222	2409,35151	-0,982900573	0,14501715	-6,7778229	1,22E-11	4,45E-10	NDP
ENSGALG00000026153	543,41516	-0,984548638	0,18483355	-5,326677	1,00E-07	2,11E-06	
ENSGALG00000014829	780,481119	-0,984611419	0,22187254	-4,4377345	9,09E-06	0,00013652	
ENSGALG00000026560	1741,85122	-0,986320846	0,13866695	-7,1128761	1,14E-12	4,80E-11	
ENSGALG00000007419	131,80991	-0,986369449	0,27049343	-3,6465561	0,00026578	0,00267683	MASP1
ENSGALG00000017021	594,509791	-0,994338044	0,18535281	-5,3645694	8,11E-08	1,74E-06	ATP7B
ENSGALG00000001795	808,510482	-0,995172186	0,18954952	-5,2501962	1,52E-07	3,12E-06	
ENSGALG00000002832	341,099375	-0,995334878	0,21073325	-4,7231981	2,32E-06	3,92E-05	IFI35
ENSGALG00000002098	280,73409	-0,997392471	0,25960887	-3,8419045	0,00012208	0,00136718	GRIK3
ENSGALG00000006563	4758,12134	-1,001967625	0,10653632	-9,4049393	5,21E-21	4,82E-19	SEMA3D
ENSGALG00000019280	829,693531	-1,005672086	0,15079747	-6,669025	2,58E-11	9,05E-10	
ENSGALG00000020876	273,366838	-1,006719844	0,26113425	-3,8551811	0,00011564	0,00130677	
ENSGALG00000003554	4815,21553	-1,008700457	0,1286997	-7,8376287	4,59E-15	2,53E-13	OPTC
ENSGALG00000016570	243,048597	-1,009438758	0,22517634	-4,4828811	7,36E-06	0,00011317	
ENSGALG00000010500	3048,93457	-1,016915026	0,1141321	-8,9099825	5,10E-19	3,85E-17	NKX6-2
ENSGALG00000020615	425,649069	-1,022483514	0,20534443	-4,9793584	6,38E-07	1,19E-05	STK32C
ENSGALG00000027891	12053,1726	-1,030686173	0,08559178	-12,041883	2,14E-33	3,77E-31	
ENSGALG00000005981	1473,77881	-1,035267465	0,18464036	-5,6069401	2,06E-08	4,90E-07	GDF2
ENSGALG00000003551	1571,2827	-1,036323807	0,13255448	-7,8180974	5,36E-15	2,94E-13	

ENSGALG00000016810	5739,43629	-1,036681311	0,12690987	-8,1686423	3,12E-16	1,90E-14	
ENSGALG00000002298	1870,01778	-1,039424987	0,15003565	-6,9278535	4,27E-12	1,67E-10	COL8A2
ENSGALG00000020788	2719,7038	-1,041075867	0,11340313	-9,1803095	4,30E-20	3,66E-18	
ENSGALG00000008279	45522,988	-1,052545903	0,11115526	-9,469151	2,82E-21	2,65E-19	
ENSGALG00000008455	5120,18929	-1,05482755	0,15577951	-6,7712854	1,28E-11	4,64E-10	
ENSGALG00000027821	3043,02821	-1,05591502	0,14824678	-7,1226844	1,06E-12	4,52E-11	
ENSGALG00000013363	1658,14825	-1,059850407	0,15135845	-7,0022548	2,52E-12	1,01E-10	
ENSGALG00000006576	474,441333	-1,060241233	0,24449814	-4,336398	1,45E-05	0,00020682	GRM3
ENSGALG00000009601	8847,52543	-1,061491773	0,12977069	-8,1797498	2,84E-16	1,75E-14	NELL2
ENSGALG00000003588	1399,08969	-1,081475528	0,13003924	-8,3165325	9,06E-17	5,72E-15	
ENSGALG00000009638	352,651719	-1,085140036	0,22094352	-4,9113911	9,04E-07	1,66E-05	ANO6
ENSGALG00000007908	401,240258	-1,09305059	0,19841137	-5,5090119	3,61E-08	8,30E-07	
ENSGALG00000007781	567,69339	-1,095474125	0,20462468	-5,3535776	8,62E-08	1,84E-06	GAL3ST1
ENSGALG00000013948	833,750503	-1,099632212	0,18641525	-5,8988318	3,66E-09	9,54E-08	RASL11B
ENSGALG00000028682	140,240123	-1,101427497	0,25860917	-4,2590428	2,05E-05	0,00028006	CBLN2
ENSGALG00000008440	326,768063	-1,108083257	0,21643685	-5,1196608	3,06E-07	6,02E-06	CHST1
ENSGALG00000007211	263,73208	-1,108214376	0,2365099	-4,6856998	2,79E-06	4,62E-05	CDH22
ENSGALG00000016636	272,318125	-1,110176301	0,23729514	-4,6784621	2,89E-06	4,78E-05	
ENSGALG00000028506	1366,3389	-1,110787668	0,13126336	-8,4622829	2,62E-17	1,71E-15	NHSL2
ENSGALG00000023904	802,719075	-1,115042621	0,15879335	-7,0219729	2,19E-12	8,94E-11	FIBIN
ENSGALG00000005985	8172,66492	-1,116443935	0,11421091	-9,7752824	1,44E-22	1,49E-20	GDF10
ENSGALG00000016511	275,151393	-1,119703281	0,22403272	-4,9979453	5,79E-07	1,10E-05	ADGRG2
ENSGALG00000012123	16654,8584	-1,123025117	0,07863736	-14,281063	2,87E-46	8,16E-44	PAX6
ENSGALG00000002028	1441,95498	-1,145532965	0,19074043	-6,0057167	1,90E-09	5,16E-08	GPRC5B
ENSGALG00000003965	6511,37534	-1,147515866	0,15881452	-7,2255096	4,99E-13	2,21E-11	DBX1
ENSGALG00000004448	445,618082	-1,158751146	0,22989436	-5,0403635	4,65E-07	8,87E-06	BRINP2
ENSGALG00000014699	1041,3098	-1,163328429	0,21482752	-5,4151741	6,12E-08	1,35E-06	MEGF10
ENSGALG00000013900	1854,85305	-1,17030528	0,14712084	-7,9547211	1,80E-15	1,03E-13	MAP7
ENSGALG00000027633	302,366053	-1,199702733	0,21814739	-5,4995053	3,81E-08	8,68E-07	RASSF9
ENSGALG00000001264	15314,0129	-1,21609258	0,1243485	-9,7797129	1,38E-22	1,43E-20	PLXNA2
ENSGALG00000008215	2557,20905	-1,222668874	0,12813223	-9,5422426	1,40E-21	1,36E-19	
ENSGALG00000006210	10737,2281	-1,225951378	0,09874124	-12,415799	2,15E-35	4,40E-33	
ENSGALG00000005967	601,737643	-1,230002366	0,21719963	-5,663004	1,49E-08	3,61E-07	
ENSGALG00000023114	289,478138	-1,251095176	0,23426953	-5,3404092	9,27E-08	1,97E-06	TAL2
ENSGALG00000026395	5473,90727	-1,254721779	0,10114284	-12,405443	2,44E-35	4,93E-33	TRABD2A
ENSGALG00000014258	1644,91976	-1,256488908	0,16302387	-7,7073924	1,28E-14	6,78E-13	LIMCH1
ENSGALG00000023577	185,179234	-1,257090663	0,26329484	-4,77446	1,80E-06	3,11E-05	
ENSGALG00000016411	2414,195	-1,262862187	0,12175871	-10,371843	3,33E-25	4,12E-23	
ENSGALG00000016428	17978,0384	-1,276203339	0,12837408	-9,9412849	2,75E-23	3,07E-21	
ENSGALG00000029012	451,347482	-1,299502297	0,18638978	-6,971961	3,13E-12	1,24E-10	
ENSGALG00000007945	609,326872	-1,299576949	0,18158766	-7,156747	8,26E-13	3,54E-11	CRYAB
ENSGALG00000009604	7273,89514	-1,349943691	0,11617707	-11,619708	3,27E-31	5,32E-29	DBX2
ENSGALG00000009948	2089,1825	-1,364538663	0,11221803	-12,15971	5,09E-34	9,80E-32	HHIP
ENSGALG00000012184	1097,0824	-1,376965225	0,21041222	-6,5441314	5,98E-11	2,01E-09	
ENSGALG00000005413	755,657663	-1,418701803	0,22290236	-6,3646784	1,96E-10	6,28E-09	
ENSGALG00000005975	1102,4413	-1,45581134	0,21067685	-6,9101628	4,84E-12	1,86E-10	
ENSGALG00000011205	1465,86067	-1,462754207	0,13057425	-11,20247	3,97E-29	5,98E-27	SMOC2
