# Supplementary material for: HOXB8 Counteracts MAPK/ERK Oncogenic Signaling in a Chicken Embryo Model of Neoplasia
Source: Int J Mol Sci. 2021 Aug 18;22(16):8911. doi: 10.3390/ijms22168911 (PMC8396257; doi:10.3390/ijms22168911)
Supplement: Supplementary file 1 [file ijms-22-08911-s001.zip › ijms-1277141 Table S3.pdf]

Table S3: Commonly differentially regulated genes between MEK1+HOXB8 vs MEK1ca and pCIG vs MEK1ca (False Discovery Rate (padj) <= 0.01). Genes that are inversely regulated are highlighted.

ID	pCIG vs MEK1ca, Log2FC	MEK1ca+HOXB8 vs MEK1ca, Log2FC	Symbol
ENSGALG00000016783	6,569509059	-5,301446621	IL1R1
ENSGALG00000026862	4,954899999	-0,764882325	CLDN1
ENSGALG00000013624	4,748606334	-3,6799039	FAM65B
ENSGALG00000007701	4,453082303	-2,906313035	B3GNT7
ENSGALG00000023406	4,424329106	-1,774894708	
ENSGALG00000004221	4,334100391	-5,338632777	IL22RA1
ENSGALG00000016431	4,191515764	-4,37340061	PAX5
ENSGALG00000006421	4,186231336	-3,742853665	SLC16A12
ENSGALG00000014994	4,160137851	-5,043766073	CRHBP
ENSGALG00000016145	3,924548833	-2,754137035	RIPK4
ENSGALG00000016906	3,743251462	-1,870006943	SPRY2
ENSGALG00000020359	3,572883143	-3,821393345	SIX6
ENSGALG00000023379	3,494464883	-3,949811074	F2RL2
ENSGALG00000010435	3,471549614	-2,340611141	RASGRP3
ENSGALG00000007656	3,445291545	-2,823200316	
ENSGALG00000005519	3,391598976	-1,767947956	TLL2
ENSGALG00000026663	3,38430006	-2,478656591	
ENSGALG00000002562	3,3452383	-3,12939254	ARAP3
ENSGALG00000008905	3,342984543	-2,456637668	HABP2
ENSGALG00000016784	3,229015878	-2,607549769	IL1RL2
ENSGALG00000008581	3,209755496	-2,566439645	DUSP5
ENSGALG00000011099	3,19842177	-2,760215905	FAP
ENSGALG00000003456	3,130034846	-3,934712358	CA12
ENSGALG00000015358	3,121559149	-3,063209848	MYH15
ENSGALG00000011940	2,983749675	-1,496267605	CCK
ENSGALG00000007963	2,963911322	-1,84239576	NFATC2
ENSGALG00000023521	2,942428455	-1,651708778	ADRA2A
ENSGALG00000007171	2,91119837	-1,518198388	CIITA
ENSGALG00000005000	2,843605103	-2,158647655	COMTD1
ENSGALG00000000600	2,828234018	-2,27272638	PTPN7
ENSGALG00000012018	2,812280515	-1,435600968	UGT8
ENSGALG00000000177	2,765911854	-1,083701806	KLF3
ENSGALG00000007346	2,763402401	-2,361764382	KIAA1462
ENSGALG00000001621	2,731716978	-1,495017381	CLDN5
ENSGALG00000010224	2,688243072	-2,486198557	GLIPR1L
ENSGALG00000009704	2,653508455	-2,934403789	CHST15
ENSGALG00000005209	2,631256548	-2,791291062	RP5-877J2.1
ENSGALG00000014984	2,599579307	-2,931476354	F2RL1
ENSGALG00000022962	2,592879602	-3,362304357	OVST
ENSGALG00000002183	2,571301428	-2,438499895	PRR16
ENSGALG00000015388	2,539489467	-1,507384513	PRDM1
ENSGALG00000016140	2,527534269	-2,998033733	FAM3B
ENSGALG00000013102	2,517151592	-1,724325032	COBL
ENSGALG00000005855	2,515154305	-1,488520056	CA5B
ENSGALG00000002816	2,498779812	-2,170634895	STK10
ENSGALG00000026258	2,487122645	-0,909500707	
ENSGALG00000003126	2,475370264	-1,615974863	ERBB4
ENSGALG00000002540	2,44573463	-2,132167559	RGS2
ENSGALG00000015826	2,399148661	-2,484029553	HTR1E
ENSGALG00000007538	2,386772965	-3,275953369	IRG1L
ENSGALG00000019054	2,38528849	-1,427749976	NAALAD2
ENSGALG00000026366	2,348234721	-1,956428809	TTN
ENSGALG00000004306	2,335846949	-1,830041848	SYT12
ENSGALG00000009393	2,335695464	-0,782303357	CAV1
ENSGALG00000007657	2,332413704	-2,412172788	CDX4
ENSGALG00000017091	2,329587636	-2,363903034	FLT1
ENSGALG00000011562	2,318757284	-2,817896047	PARK2
ENSGALG00000012051	2,272220282	-2,878544854	STAC

ENSGALG00000011207	2,267210819	-1,434035643	DUSP6
ENSGALG00000010036	2,253052375	-0,717787007	FOSL2
ENSGALG00000000949	2,249487158	-1,013784064	HBEGF
ENSGALG00000004376	2,243095428	-2,323750078	KCNJ2
ENSGALG00000014159	2,234266769	-1,81424576	PACSIN2
ENSGALG00000017244	2,226234853	-2,189436741	PRSS23
ENSGALG00000007786	2,190066734	-1,895122662	DOK5
ENSGALG00000017242	2,189728881	-1,579394776	FZD4
ENSGALG00000005061	2,1754071	-2,114741441	PPM1E
ENSGALG00000003437	2,148196892	-1,896224168	ADAM8
ENSGALG00000005499	2,068323361	-1,531899327	IL17RD
ENSGALG00000006995	2,052973523	-1,759714805	APOD
ENSGALG00000004594	2,028493881	-0,774792529	CD74
ENSGALG00000006112	2,027013529	-2,692857061	SCN5A
ENSGALG00000025941	1,970690155	-2,032822244	RGS20
ENSGALG00000012732	1,970234077	-2,298252682	PHACTR1
ENSGALG00000017179	1,969058193	-1,548130217	PDGFD
ENSGALG00000007940	1,939184933	-2,542363859	RAG2
ENSGALG00000011833	1,93191024	-0,971207285	SPATA5
ENSGALG00000003323	1,918109692	-1,591926379	OSGIN1
ENSGALG00000012635	1,911001153	-1,755097361	BTBD11
ENSGALG00000022490	1,907440028	-0,638026939	ARPC1B
ENSGALG00000008461	1,900360149	0,900341356	HGF
ENSGALG00000007415	1,899486225	0,938743099	SH3RF2
ENSGALG00000000544	1,895252672	1,877518925	
ENSGALG00000016785	1,892879929	-2,405348378	IL1RL1
ENSGALG00000005498	1,873960213	-1,707286081	
ENSGALG00000008353	1,848707427	-1,679225856	MAGI2
ENSGALG00000010597	1,8345583	-0,973101915	CRIM1
ENSGALG00000012106	1,828065766	-2,236831516	SCTR
ENSGALG00000000141	1,818659764	-1,223136562	BLB1
ENSGALG00000026262	1,788029436	-1,4922838	PMP22
ENSGALG00000010468	1,7857319	-1,6309453	ISM2
ENSGALG00000003706	1,784490953	-1,390323026	DUSP1
ENSGALG00000011581	1,763989645	-1,059360643	KIF26A
ENSGALG00000023241	1,749836088	-0,938819916	
ENSGALG00000027521	1,741041239	-0,803725098	ANKRD33B
ENSGALG00000012623	1,738546704	-2,482707417	CFC1B
ENSGALG00000029175	1,731471147	-1,685259625	EFNB2
ENSGALG00000001560	1,726930021	-2,555527127	KCNU1
ENSGALG00000024083	1,719739442	-2,175833689	C8orf4
ENSGALG00000005069	1,702946767	0,669583836	PTGS2
ENSGALG00000005689	1,702270541	-2,271345118	PAX2
ENSGALG00000009693	1,692012083	-1,908196694	CPXM2
ENSGALG00000015332	1,689640626	-2,044938557	CPLX1
ENSGALG00000006084	1,684603389	-1,531133293	WNT8A
ENSGALG00000028250	1,684255106	-2,884651456	ANKRD22
ENSGALG00000010258	1,674285408	-1,528410178	LTBP2
ENSGALG00000008281	1,659542176	-1,009962725	NEURL1
ENSGALG00000013033	1,649436663	-2,410715686	CMBL
ENSGALG00000009740	1,648366239	-1,063900656	RASGRP1
ENSGALG00000010910	1,643397512	-0,801671392	RAPGEF5
ENSGALG00000016602	1,632391617	-1,339565689	ARHGAP6
ENSGALG00000026989	1,621656649	-0,47543634	
ENSGALG00000016377	1,612690665	-1,984049771	PXDN
ENSGALG00000027218	1,609222298	1,190798556	
ENSGALG00000008118	1,603231134	-2,340544818	RFTN2
ENSGALG00000009282	1,600131022	-1,769054515	ELMSAN1
ENSGALG00000027707	1,597314136	-1,082314729	ESM1
ENSGALG00000005347	1,58404156	-2,102469906	ADAMTS18
ENSGALG00000001383	1,582310757	-1,543511015	HTR4
ENSGALG00000008890	1,553993643	-2,449553325	ADGRL4

ENSGALG00000017714	1,553191397	-1,052113718	FHAD1
ENSGALG00000011148	1,54566024	-1,754606037	HHIPL1
ENSGALG00000011962	1,540068054	-0,494326456	NDNF
ENSGALG00000008875	1,539400497	-1,156831558	PLCB1
ENSGALG00000001189	1,519846307	0,851541498	NCF1
ENSGALG00000016754	1,508945346	-1,034769759	MGAT4A
ENSGALG00000006997	1,507910301	-1,684979264	BRINP1
ENSGALG00000019426	1,491625247	-2,280006749	TMEM71
ENSGALG00000011236	1,484958654	-1,070413941	BEGAIN
ENSGALG00000002594	1,484249601	-3,661637709	TFPI
ENSGALG00000014930	1,479606742	-1,696897635	ENC1
ENSGALG00000010683	1,466981653	-0,919156764	S1PR3
ENSGALG00000007660	1,456310727	-1,228207524	CHIC1
ENSGALG00000028130	1,44989129	1,140619832	KIAA1644
ENSGALG00000015865	1,445378264	-0,393610955	FAM46A
ENSGALG00000009667	1,442248321	-1,219318734	MFAP3L
ENSGALG00000005442	1,437319869	-1,327990169	PALMD
ENSGALG00000007771	1,43708626	-1,353742442	FAM210B
ENSGALG00000026790	1,433302155	-0,903869706	DMRT2
ENSGALG00000027210	1,422887936	-1,142415301	
ENSGALG00000007218	1,41095016	-1,732428983	MB21D2
ENSGALG00000012702	1,407925441	-0,483830731	JARID2
ENSGALG00000009546	1,402581457	-0,832251183	HTRA1
ENSGALG00000011468	1,39801296	-1,424650779	IGFBP5
ENSGALG00000015665	1,394518864	-1,188036426	SH3BP2
ENSGALG00000011680	1,389021273	-0,709602993	EPHA5
ENSGALG00000011419	1,382981881	-0,529859697	DUSP4
ENSGALG00000014852	1,380781721	-1,119019515	
ENSGALG00000010049	1,375590195	-0,6748978	PTPRB
ENSGALG00000006401	1,372021166	-1,299022844	OSBPL5
ENSGALG00000002326	1,369220123	-0,593338649	
ENSGALG00000007846	1,3613354	-0,863598394	
ENSGALG00000011120	1,356774191	-0,88227849	SLC4A10
ENSGALG00000004569	1,348974691	-0,82424563	UNC5B
ENSGALG00000012911	1,345449733	-0,929872331	SYT10
ENSGALG00000015028	1,342064123	-0,946739996	RLN1
ENSGALG00000005476	1,338834069	-1,752170936	PTH1R
ENSGALG00000000107	1,334266505	1,227291307	TRIM7.1
ENSGALG00000006258	1,330333906	-0,778635815	SPOCK1
ENSGALG00000015089	1,327665029	-1,867848339	GAP43
ENSGALG00000011110	1,322132253	-1,431359085	DPP4
ENSGALG00000008893	1,318238187	-2,444595373	SYT4
ENSGALG00000005805	1,313014625	-0,954718819	PLCD1
ENSGALG00000010311	1,29508602	-0,865329105	NAV3
ENSGALG00000002993	1,293663972	-1,397301236	CA10
ENSGALG00000015256	1,284509548	-1,397562325	PAM
ENSGALG00000015908	1,276423655	-0,970529728	COL12A1
ENSGALG00000014727	1,275557809	-2,02826974	PDE4D
ENSGALG00000016817	1,2720896	-0,658902643	RASA3
ENSGALG00000007253	1,27114813	1,036667095	IL1RAP
ENSGALG00000009920	1,271106358	-1,80046145	COCH
ENSGALG00000002632	1,269452785	-1,220611399	CALCRL
ENSGALG00000016102	1,263113327	-1,630912312	CYP26B1
ENSGALG00000001608	1,262511696	-2,081100043	UNC5D
ENSGALG00000016998	1,256730923	-1,177970615	
ENSGALG00000011831	1,24831638	-0,347478626	ANKRD50
ENSGALG00000028047	1,243898501	-1,369008166	RHOV
ENSGALG00000010010	1,237630354	-0,869227214	RHOQ
ENSGALG00000009947	1,236965236	-1,358616298	PLEKHH2
ENSGALG00000014983	1,23637443	-1,39278689	F2R
ENSGALG00000005619	1,235271948	-1,189698364	F3
ENSGALG00000015653	1,234972794	-0,655959247	JPH1

ENSGALG00000003114	1,234804889	-1,398035927	NOG
ENSGALG00000019716	1,234170903	-1,618712644	KRT20
ENSGALG00000019261	1,230099163	-1,655584971	ARHGAP8
ENSGALG00000028821	1,225133338	-1,46102831	GLIPR2
ENSGALG00000008999	1,220108565	0,583129446	AFAP1L2
ENSGALG00000014823	1,217853522	-1,422401118	C6orf58
ENSGALG00000000307	1,215832976	-0,894951246	CERS4
ENSGALG00000007268	1,214609385	-1,168892699	CADPS
ENSGALG00000000721	1,214440274	-0,47173717	SLC41A1
ENSGALG00000026557	1,203253046	-1,412974627	PGF
ENSGALG00000002555	1,202502668	-0,892902927	RET
ENSGALG00000027133	1,199491369	-1,405319409	CD83
ENSGALG00000009360	1,196193521	-0,890830846	CAPN2
ENSGALG00000014864	1,190480858	-1,150010609	SMPDL3A
ENSGALG00000012927	1,187339616	-2,419967236	CDH10
ENSGALG00000016455	1,186649639	-2,034647249	GREB1
ENSGALG00000011867	1,179474298	-1,242989678	SERHL2
ENSGALG00000002294	1,174006641	-0,482168758	SEMA6A
ENSGALG00000002543	1,169335598	-0,840502021	CSGALNACT2
ENSGALG00000013892	1,167370699	-1,122648268	MAP3K5
ENSGALG00000011439	1,165074777	-1,116853678	RBMS3
ENSGALG00000009450	1,162765074	-1,016064164	DUSP10
ENSGALG00000023768	1,155472868	0,58136874	EPHA2
ENSGALG00000016927	1,145535192	-0,715628819	KLF5
ENSGALG00000006988	1,141001037	0,944077449	RNF213
ENSGALG00000012998	1,132683468	-0,849149352	WNT5B
ENSGALG00000028046	1,129369719	-1,098357134	INSIG2
ENSGALG00000014866	1,123162841	-1,312013425	FABP7
ENSGALG00000009567	1,119009905	-1,113206952	TLL1
ENSGALG00000026970	1,11847741	-0,785311616	
ENSGALG00000006376	1,116147137	-1,138122247	TMC3
ENSGALG00000008469	1,115125474	-0,83875822	SPINT1
ENSGALG00000010692	1,111701613	-0,996294098	LRP8
ENSGALG00000010081	1,110146926	-0,563235953	SH3D19
ENSGALG00000006337	1,109144959	-1,128489411	K123
ENSGALG00000026763	1,104647316	-1,177313255	
ENSGALG00000009170	1,101783321	-1,581054934	NCEH1
ENSGALG00000006797	1,10056236	-2,093089949	ADARB2
ENSGALG00000015980	1,100313329	-0,841274637	
ENSGALG00000012439	1,098736639	-1,028638845	IGFBP3
ENSGALG00000026518	1,097099741	-0,773283921	RUNDC3A
ENSGALG00000028582	1,093959706	0,832821984	LMO2
ENSGALG00000002714	1,089564948	-1,839377527	TMEM132C
ENSGALG00000016875	1,086190922	-1,825016077	CLYBL
ENSGALG00000005492	1,08468259	-0,609713298	NBEAL2
ENSGALG00000007113	1,078758406	-1,77145981	TNC
ENSGALG00000006822	1,077915026	-2,117548167	PCDH19
ENSGALG00000023581	1,073595233	-0,876769713	
ENSGALG00000007056	1,069890641	-0,638790551	KLF6
ENSGALG00000014907	1,069885451	-0,80019622	DCBLD1
ENSGALG00000006808	1,068278734	-0,948868926	TSPAN6
ENSGALG00000022871	1,067659168	-1,176605905	HHLA2
ENSGALG00000028708	1,054408367	-0,938854547	
ENSGALG00000025773	1,052013322	-1,506718569	
ENSGALG00000001020	1,049473012	-0,780112538	KIFC3
ENSGALG00000028602	1,04212846	-0,881510014	
ENSGALG00000012594	1,03128421	-2,283939186	NTRK2
ENSGALG00000017411	1,030664685	-1,17758282	RXFP3
ENSGALG00000011609	1,027774555	-1,61436812	ABTB2
ENSGALG00000009129	1,026339944	-0,878296092	DLX5
ENSGALG00000004393	1,024579421	-0,962441655	
ENSGALG00000007646	1,023532832	-1,999886692	

ENSGALG00000016841	1,021854986	-0,576008454	COL4A1
ENSGALG00000006252	1,021389981	-0,49037706	LMO4
ENSGALG00000008491	1,018906247	-1,354708919	XIAP
ENSGALG00000006638	1,014405382	-0,469621749	GRIK4
ENSGALG00000028294	1,012130398	-0,727698928	VWA5B2
ENSGALG00000002532	1,010182876	-0,997428466	GJA4
ENSGALG00000007069	1,009530133	-0,738101541	ADAMTS17
ENSGALG00000006908	1,006453251	-0,789724754	DLG1
ENSGALG00000015440	1,001448719	-0,996123586	RCSD1
ENSGALG00000001419	0,999950253	-0,758492411	DAB2IP
ENSGALG00000015208	0,998078994	-0,778489641	ROR2
ENSGALG00000008918	0,995278864	-1,685987772	LAMP5
ENSGALG00000003149	0,985359473	-0,749443102	ITPR3
ENSGALG00000020723	0,981929794	-0,913543779	NOG2
ENSGALG00000016537	0,979050265	-0,739933749	SCML2
ENSGALG00000027497	0,976598532	-0,973179721	CFD
ENSGALG00000004930	0,975284752	-1,782009807	HRH1
ENSGALG00000016143	0,975263193	0,607480677	RHPN1
ENSGALG00000010926	0,975212806	-1,152009571	SPP1
ENSGALG00000012808	0,972939427	-0,789210521	CDYL
ENSGALG00000003739	0,97006222	-1,237172502	SALL1
ENSGALG00000014343	0,963080504	-1,412639292	ARAP2
ENSGALG00000002509	0,962423205	-1,185807324	COL5A2
ENSGALG00000021879	0,950789217	-1,34129965	
ENSGALG00000006614	0,950175613	2,721769719	TECTA
ENSGALG00000016843	0,946578268	-0,926939677	COL4A2
ENSGALG00000017135	0,945725278	-1,071209509	CRYL1
ENSGALG00000012224	0,94448356	-1,20528636	PDE1C
ENSGALG00000008014	0,943508119	-1,126068911	CEBPB
ENSGALG00000026892	0,943046494	-1,6734567	
ENSGALG00000010163	0,938977707	-0,998496131	LGR5
ENSGALG00000004662	0,937959127	-0,792400882	DISP3
ENSGALG00000010836	0,937907565	-0,688393097	AHR
ENSGALG00000002397	0,927215999	-1,291814757	PSPH
ENSGALG00000010412	0,925287079	-0,756929566	WWTR1
ENSGALG00000007039	0,92399781	-0,823801989	IGF1R
ENSGALG00000008778	0,923325024	-0,51039033	SPRED2
ENSGALG00000010785	0,922406238	-0,818155142	ACOT11
ENSGALG00000015164	0,920081875	-0,982829392	PCSK5
ENSGALG00000014855	0,916891226	-0,627700184	GHR
ENSGALG00000007667	0,910790115	-0,757882718	GPR158
ENSGALG00000012533	0,909053879	-0,619977689	MYH9
ENSGALG00000011618	0,908730543	-0,609057453	ADSSL1
ENSGALG00000015624	0,906952704	-0,97910183	VCAN
ENSGALG00000001645	0,906502233	-1,608468631	KCNT1
ENSGALG00000011258	0,899386948	-0,946927699	ATP2B1
ENSGALG00000016308	0,888326042	-0,690314419	MYC
ENSGALG00000007952	0,887725324	-0,932963194	AMOT
ENSGALG00000001281	0,886212495	1,101845994	PHOSPHO1
ENSGALG00000010148	0,885936489	-1,379685752	PTPRR
ENSGALG00000001276	0,885240877	-1,144775826	HOXB9
ENSGALG00000015896	0,883690573	-1,023057651	MMP16
ENSGALG00000005256	0,880582454	-0,63393871	EPHA4
ENSGALG00000009179	0,880187457	-1,119360692	TNFSF10
ENSGALG00000011172	0,879786034	-1,121518184	MYO10L
ENSGALG00000027885	0,876800098	-1,151437329	C4orf19
ENSGALG00000014783	0,875888949	-0,572122303	PTPRK
ENSGALG00000009405	0,875608773	-1,391691484	GRIA2
ENSGALG00000006008	0,867963452	0,882382705	HOMER2
ENSGALG00000029023	0,865902724	-1,242039059	DMRTB1
ENSGALG00000016909	0,864393738	-0,488526858	NDFIP2
ENSGALG00000026582	0,863598751	-0,997100015	SOX18

ENSGALG00000001617	0,855845667	1,898502237	CAMKK1
ENSGALG00000015180	0,851097646	-0,540189844	PSAT1
ENSGALG00000016807	0,848101313	-0,574724846	LIMS1
ENSGALG00000000977	0,842188766	-1,032464943	MAPKAP1
ENSGALG00000009530	0,841159622	-1,411853584	PDZRN4
ENSGALG00000004155	0,841077447	-1,147734088	MYOM3
ENSGALG00000015409	0,837042297	-0,599227418	PROS1
ENSGALG00000012278	0,835859144	-2,383840075	EMCN
ENSGALG00000010156	0,833499217	-1,025083823	DOCK8
ENSGALG00000001220	0,831042878	-0,818155857	HERPUD1
ENSGALG00000013085	0,830893947	-0,535478159	PTPRO
ENSGALG00000025770	0,830841194	-1,089960517	
ENSGALG00000012354	0,829092837	-0,521764337	VOPP1
ENSGALG00000000639	0,827648848	-0,79001541	LRRN2
ENSGALG00000011232	0,825691237	-0,469477808	STK11IP
ENSGALG00000020899	0,823141433	-0,585391733	
ENSGALG00000010664	0,817672769	0,852579615	KIF26B
ENSGALG00000008670	0,815447938	-0,486758564	C1orf52
ENSGALG00000013683	0,810200928	-0,780228434	ARID1B
ENSGALG00000012848	0,809997189	-0,711139877	KDM7A
ENSGALG00000014872	0,806209646	-2,137267919	FGF10
ENSGALG00000005430	0,79963845	-0,72235333	ALS2CL
ENSGALG00000012849	0,79670503	-0,57279907	SLC37A3
ENSGALG00000002443	0,789654091	-1,353430658	FBXL16
ENSGALG00000009252	0,7810557	-0,788959505	PLD1
ENSGALG00000002598	0,778822215	-0,82047281	TPST1
ENSGALG00000004158	0,769428918	-0,467330867	GPT2
ENSGALG00000001346	0,76776324	-1,362109597	
ENSGALG00000013124	0,765332021	-1,026819624	FHOD3
ENSGALG00000011376	0,758366172	-0,870171475	ANKRD9
ENSGALG00000001000	0,756534693	-0,924720639	HSPA5
ENSGALG00000012012	0,743046962	-0,895703636	CMIP
ENSGALG00000011319	0,737544915	-1,42017882	LPHN3
ENSGALG00000005512	0,736922168	-0,667653797	KIAA1671
ENSGALG00000006138	0,736205494	-1,026936244	HELZ2
ENSGALG00000003043	0,734167184	-0,395518744	BBS2
ENSGALG00000005653	0,729875771	-0,515161656	NFKB2
ENSGALG00000007501	0,729453053	-0,702573548	P2RX3
ENSGALG00000015184	0,727266294	-0,431383947	TLE4
ENSGALG00000009381	0,723727203	-0,972226453	SIPA1L1
ENSGALG00000001370	0,722550699	-1,073396904	ADAMTS8
ENSGALG00000015947	0,718924866	-2,438977585	CDH17
ENSGALG00000008163	0,713636421	-0,439350797	PSME4
ENSGALG00000015975	0,705892008	-1,056406602	DPY19L4
ENSGALG00000015310	0,701805821	-0,587354563	SOBP
ENSGALG00000002198	0,701337674	-0,485078535	FHOD1
ENSGALG00000010522	0,699893806	-0,747786716	ALDH18A1
ENSGALG00000010204	0,699072042	-0,660708753	KCNC2
ENSGALG00000012956	0,692712504	1,372804533	TMTC1
ENSGALG00000016835	0,685225343	-0,539714594	ATP11A
ENSGALG00000012696	0,682603698	-0,443665071	RANBP9
ENSGALG00000016281	0,681257671	-0,569360911	DMD
ENSGALG00000003640	0,678918743	1,071788322	FAM20C
ENSGALG00000027342	0,672830693	-0,968330352	METRN
ENSGALG00000027389	0,672548469	-0,766788012	RGS7BP
ENSGALG00000002646	0,671803119	-0,515104787	FAM171B
ENSGALG00000004612	0,671680213	-0,579433401	MTHFR
ENSGALG00000007704	0,666805577	-0,445645488	NPM3
ENSGALG00000023876	0,666027019	0,598475769	PRKCQ
ENSGALG00000015887	0,665885713	-0,776552733	URB1
ENSGALG00000003957	0,665113393	-1,565814231	APOH
ENSGALG00000006872	0,664035354	-0,823650254	PISD

ENSGALG00000014957	0,661960443	-0,81285735	WDR1
ENSGALG00000002655	0,657486198	-0,468798244	ITGAV
ENSGALG00000004231	0,654722209	-0,911405982	IFNLR1
ENSGALG00000011770	0,65450966	-0,525890593	UMPS
ENSGALG00000016803	0,652622211	-0,451555414	ST6GAL2
ENSGALG00000013031	0,641018743	-0,574014118	CECR1
ENSGALG00000027835	0,637590923	-0,430117051	PCBD1
ENSGALG00000024346	0,637233355	0,525612903	FAM46B
ENSGALG00000008318	0,633974687	-0,756789619	SLK
ENSGALG00000004181	0,628709896	-0,425938272	GAA
ENSGALG00000014922	0,627329029	-0,504425665	ROCK1
ENSGALG00000012726	0,625221924	-0,941672551	HSP90B1
ENSGALG00000011435	0,614694006	0,35897775	ELK3
ENSGALG00000019397	0,608262914	-0,975439675	CERK
ENSGALG00000026468	0,607177306	-0,514962132	CHSY1
ENSGALG00000000795	0,605895026	-0,495778483	MFSD12
ENSGALG00000006939	0,603844144	-0,728311968	PFKP
ENSGALG00000016834	0,598292773	-0,698351437	MCF2L
ENSGALG00000011548	0,593504022	-0,434202614	PPP1R13B
ENSGALG00000002709	0,59270728	-0,511853662	MYO1C
ENSGALG00000008615	0,588449543	-1,233824562	LTK
ENSGALG00000011295	0,585889038	-1,007421144	SOCS2
ENSGALG00000012979	0,583725131	-0,528975634	TRIO
ENSGALG00000007242	0,576491214	-0,724284111	SLITRK4
ENSGALG00000011045	0,574987094	-0,799075807	AFF1
ENSGALG00000020479	0,574556644	-0,731996794	RP11-872D17.8
ENSGALG00000012429	0,572869136	1,680823476	BMP4
ENSGALG00000019961	0,57282511	-0,7532835	SLC18B1
ENSGALG00000009415	0,570748764	-0,939656238	SMOC1
ENSGALG00000011708	0,569570289	-1,833902608	MYLK
ENSGALG00000009156	0,56553435	-0,682556058	CDAN1
ENSGALG00000012394	0,564570466	-0,855781498	NID2
ENSGALG00000008293	0,562981441	-0,66560113	SH3PXD2A
ENSGALG00000008621	0,561672993	-0,76612038	NRP2
ENSGALG00000003122	0,561038511	0,68839827	ZFAND2A
ENSGALG00000015862	0,559415098	-0,756350316	CA2
ENSGALG00000027757	0,559044043	-1,076478944	ARL4A
ENSGALG00000017462	0,558530219	-0,763892042	FNIP1
ENSGALG00000000388	0,558245728	-1,555950834	LIN28A
ENSGALG00000007238	0,558183051	0,466556765	PCSK6
ENSGALG00000012852	0,557720187	-0,42431457	MKRN1
ENSGALG00000016480	0,557041155	-0,647091328	SDC1
ENSGALG00000028291	0,555590355	-0,672824764	BCAR1
ENSGALG00000009433	0,551628222	0,528259009	BAG3
ENSGALG00000000680	0,550999971	-1,411570576	NUAK2
ENSGALG00000002461	0,549280944	-0,808165073	MMP17
ENSGALG00000015215	0,543882262	-0,397390834	MAPRE2
ENSGALG00000017365	0,541410874	-0,712049377	REEP2
ENSGALG00000014860	0,540418516	-0,625239897	YES1
ENSGALG00000016781	0,539823119	-0,504088725	MAP4K4
ENSGALG00000016883	0,537559856	-0,555002491	DOCK9
ENSGALG00000011449	0,537091274	-0,594721435	
ENSGALG00000009855	0,536813312	-0,567662024	TBC1D9
ENSGALG00000000891	0,531658469	-0,533422492	NSUN5
ENSGALG00000015112	0,528998124	-1,013051509	ZNF521
ENSGALG00000006002	0,517539205	-0,422978689	WHDC1
ENSGALG00000011162	0,516837911	-0,640538648	CYP46A1
ENSGALG00000000761	0,511636934	-0,649687901	TSKU
ENSGALG00000008811	0,510104856	-0,931412058	SLC1A4
ENSGALG00000003668	0,508101808	-0,85921128	PITPNC1
ENSGALG00000015458	0,50234495	-0,453606012	ILDR2
ENSGALG00000017388	0,49802444	-0,619582917	YY1

ENSGALG00000009503	0,49017284	-0,547842813	RAB3GAP2
ENSGALG00000000936	0,490067343	-1,193444435	LMX1B
ENSGALG00000016768	0,489550679	-0,54750013	LONRF2
ENSGALG00000001962	0,488180846	-0,330389801	PTBP1
ENSGALG00000002316	0,486190455	-0,700708149	NUBP2
ENSGALG00000005727	0,485528575	-0,548590838	SLC7A3
ENSGALG00000013661	0,485373842	-0,40778269	MICU3
ENSGALG00000006237	0,48464383	-0,616678741	PKN2
ENSGALG00000006121	0,483017989	-0,468808823	PIK3C2A
ENSGALG00000003835	0,482616746	-0,552761335	ATP2A2
ENSGALG00000028128	0,475145623	0,378077792	HOXD8
ENSGALG00000014953	0,474792932	-0,429472364	RWDD1
ENSGALG00000014873	0,474224225	-0,6514166	GJA1
ENSGALG00000009532	0,469457498	-1,242374232	GXYLT1
ENSGALG00000016808	0,46860233	-0,615970468	RANBP2
ENSGALG00000002726	0,464112357	-0,708755608	KIF1B
ENSGALG00000004144	0,459129046	-0,588131949	DDX50
ENSGALG00000009850	0,458569963	-0,63459607	NOVA1
ENSGALG00000014688	0,458090231	-0,396059354	ZYX
ENSGALG00000013480	0,457972294	-0,615680556	MTHFD2
ENSGALG00000015940	0,457737572	-0,972506334	TMEM67
ENSGALG00000005134	0,45766228	-0,483437706	ZSWIM8
ENSGALG00000003295	0,455390197	-1,063705711	ADAMTS12
ENSGALG00000008521	0,447794364	-0,395949661	TP53BP1
ENSGALG00000006947	0,446651103	0,64187784	BDH1
ENSGALG00000001596	0,445911724	-0,383722949	MLLT1
ENSGALG00000009300	0,444814977	-0,413699825	NUMB
ENSGALG00000005785	0,442398124	-0,791416097	UBAP2
ENSGALG00000004169	0,441293938	-0,613558741	
ENSGALG00000001957	0,438219772	-0,541703645	ITCH
ENSGALG00000013861	0,437973226	-0,913164571	TNFAIP3
ENSGALG00000014419	0,437804845	-0,730417334	SLIT2
ENSGALG00000011464	0,435819807	-0,582311331	EIF5
ENSGALG00000011643	0,435706974	-0,816325927	UTP20
ENSGALG00000011707	0,43414341	-0,516018775	UBA5
ENSGALG00000014686	0,43250268	-0,691021625	FBN2
ENSGALG00000009534	0,430698068	-0,408065762	PLEKHA1
ENSGALG00000001376	0,43069552	0,45012042	ARID3B
ENSGALG00000007659	0,429907921	-0,45246564	HYOU1
ENSGALG00000014784	0,427987842	-0,520975246	MAST4
ENSGALG00000004638	0,427666392	-0,48870476	HEPH
ENSGALG00000010975	0,425801788	-0,624280901	TMTC2
ENSGALG00000011246	0,419525625	-0,471179653	CHPF
ENSGALG00000004531	0,419189684	-0,458962037	PLOD1
ENSGALG00000020805	0,416086722	-0,719835862	LRRC8D
ENSGALG00000005723	0,412949734	-0,890647194	RP11-96O20.4
ENSGALG00000007141	0,412800257	-0,70907976	LRRK1
ENSGALG00000005796	0,407045166	-0,664735985	XBP1
ENSGALG00000023451	0,405630741	-0,638494594	TRPM7
ENSGALG00000012814	0,404889777	-0,898069575	PXDC1
ENSGALG00000028267	0,403950335	-0,473386218	GOLPH3
ENSGALG00000017085	0,403507696	-0,511832688	SLC7A1
ENSGALG00000009748	0,403044512	-0,762363604	ASNS
ENSGALG00000011101	0,40109735	-0,47005983	URB2
ENSGALG00000008195	0,394418985	-0,378910892	NPHP1
ENSGALG00000002763	0,38996775	-0,423193935	FRZB
ENSGALG00000014902	0,387988657	-0,650325285	RP1-179P9.3
ENSGALG00000008708	0,384060223	-0,423296325	ESF1
ENSGALG00000004200	0,383702067	-0,347970886	DPP9
ENSGALG00000019077	0,383662275	-1,030078317	
ENSGALG00000015765	0,383191974	-0,587270267	POLR1A
ENSGALG00000000464	0,378042232	0,587838297	RHOBTB2

ENSGALG00000008240	0,376593038	-0,670953542	CFLAR
ENSGALG00000014213	0,372421493	-0,596053951	PRR5
ENSGALG00000009428	0,369028406	-0,792261942	SUSD6
ENSGALG00000003180	0,367625634	-0,573999724	TSR1
ENSGALG00000006336	0,365567709	-0,452052929	PAXIP1
ENSGALG00000005845	0,364474801	-0,655210424	SLC7A5
ENSGALG00000004696	0,360778491	-0,381141883	LEO1
ENSGALG00000009562	0,359101036	-0,714412903	PLEK2
ENSGALG00000007145	0,358001987	-0,440432711	ITGB1
ENSGALG00000010603	0,356862546	-0,604746743	CEBPZ
ENSGALG00000003563	0,354826794	-0,364389392	YARS
ENSGALG00000005160	0,351320099	-0,454934871	VMP1
ENSGALG00000002954	0,350618821	-0,457794297	MBTD1
ENSGALG00000001749	0,34679817	-0,88875485	ACSBG2
ENSGALG00000003427	0,346212827	-0,813323099	USP3
ENSGALG00000009540	0,344720194	-0,9229711	YAF2
ENSGALG00000006437	0,344612756	-0,682364759	NOM1
ENSGALG00000001003	0,344161191	-0,600081977	SEPN1
ENSGALG00000007234	0,343143822	-0,505755971	CLCN5
ENSGALG00000025884	0,339208538	-0,543082298	NABP1
ENSGALG00000026349	0,337656858	-0,736634172	HSD17B12
ENSGALG00000012202	0,337252452	-0,341191282	KBTBD2
ENSGALG00000010976	0,337203087	-0,434636867	CCDC126
ENSGALG00000006975	0,33439022	-0,659116429	TBC1D22A
ENSGALG00000003767	0,331888441	-0,636798593	NKD1
ENSGALG00000006256	0,33180927	-0,600800071	MAP7D3
ENSGALG00000015049	0,323309666	-0,441268065	AMD1
ENSGALG00000004132	0,317911072	-0,797075856	UHRF1
ENSGALG00000009448	0,31659087	-0,43880669	IFRD1
ENSGALG00000000258	0,314352619	-0,649481888	HEATR1
ENSGALG00000009992	0,311546697	-0,521017168	ARHGAP5
ENSGALG00000008837	0,311145069	-0,471745405	PELI1
ENSGALG00000008265	0,310830618	-0,619236444	PDCD11
ENSGALG00000011228	0,310544896	-0,565699287	WARS
ENSGALG00000017111	0,309121441	-0,428517877	NUP58
ENSGALG00000006971	0,308524903	-0,549657911	CDK5RAP2
ENSGALG00000003039	0,308092774	-0,372512284	SRM
ENSGALG00000000346	0,306247189	-0,429865287	ZNF606
ENSGALG00000002863	0,306238468	-0,447764784	ERGIC1
ENSGALG00000010349	0,300633215	-0,358393493	POLR1C
ENSGALG00000007387	0,298075088	-0,330955786	GTF3C1
ENSGALG00000011357	0,296426831	-0,387039211	VEZT
ENSGALG00000010536	0,29615271	-0,646034394	FAF1
ENSGALG00000011400	0,295706421	-0,550294288	USP44
ENSGALG00000012138	0,295412949	-0,402996238	sept-07
ENSGALG00000002985	0,293105421	-0,378678888	UTP18
ENSGALG00000003391	0,293097669	-0,827807721	EIF2AK1
ENSGALG00000006856	0,290795459	-0,537151634	CD151
ENSGALG00000006895	0,290027475	-0,543915173	CEP131
ENSGALG00000016910	0,283615388	-0,572230903	RBM26
ENSGALG00000009325	0,276324484	-0,363294006	OLA1
ENSGALG00000003339	0,27482717	-0,366040447	MTOR
ENSGALG00000010481	0,274549325	-0,330642085	CMPK1
ENSGALG00000005243	0,272933583	-0,597738338	PPP3CB
ENSGALG00000009782	0,272414043	-0,507579083	NAA15
ENSGALG00000011596	0,272230864	-0,519655791	PTPN4
ENSGALG00000006935	0,270244925	-0,506724591	BTAF1
ENSGALG00000003078	0,264245742	-0,483195985	MMD
ENSGALG00000026300	-0,257041042	0,345400821	
ENSGALG00000020003	-0,275409017	-0,59891041	MAP3K4
ENSGALG00000027963	-0,279887477	0,451771641	COX7C
ENSGALG00000027710	-0,296769112	0,421872733	DAG1

ENSGALG00000015326	-0,301560094	0,524363909	CLTA
ENSGALG00000006094	-0,306148177	-0,454736184	PHF6
ENSGALG00000012121	-0,306219207	-0,541445831	RCN1
ENSGALG00000012546	-0,312592383	0,318249627	MCM5
ENSGALG00000026437	-0,314002706	0,703536871	
ENSGALG00000025764	-0,316576106	0,675250138	FKBP10
ENSGALG00000006483	-0,318521408	-0,399267586	AMOTL2
ENSGALG00000013784	-0,32170776	0,339967639	CDT1
ENSGALG00000016870	-0,322919481	0,454001604	TMTC4
ENSGALG00000020528	-0,323800436	0,424819785	INIP
ENSGALG00000004621	-0,328680536	-0,512109985	PREX1
ENSGALG00000028470	-0,330867645	0,39443368	GSR
ENSGALG00000021365	-0,33098251	0,432965219	DCTN3
ENSGALG00000025818	-0,334464872	0,571475138	KCNK5
ENSGALG00000004581	-0,341091866	0,466855623	NDST1
ENSGALG00000000684	-0,343510226	-0,552570682	KLHDC8A
ENSGALG00000008758	-0,348320506	-0,696392948	B3GNT5
ENSGALG00000010674	-0,348433039	-0,399166032	DCTD
ENSGALG00000016034	-0,349640251	-0,4051791	ATRN
ENSGALG00000014970	-0,352181226	0,397421157	FSTL1
ENSGALG00000000921	-0,356222635	0,454646053	SLC9A1
ENSGALG00000017329	-0,359975454	0,44555284	FCHSD2
ENSGALG00000026081	-0,379250879	0,588777417	RBM38
ENSGALG00000015469	-0,382238702	1,115704157	IGSF3
ENSGALG00000006702	-0,38370743	0,471728545	MFGE8
ENSGALG00000024047	-0,387381439	-0,380223912	MYCL
ENSGALG00000006606	-0,392475054	1,610976489	KIAA1324L
ENSGALG00000003457	-0,397746039	0,662964808	CNP
ENSGALG00000013067	-0,398595631	0,494753671	AKR1B1
ENSGALG00000028567	-0,405726705	0,515072598	MYL9
ENSGALG00000026870	-0,410373931	0,966911139	TMEM200C
ENSGALG00000016152	-0,412571324	0,558712272	LY6E
ENSGALG00000011778	-0,415058611	0,494085012	ITGB5
ENSGALG00000011238	-0,417063936	0,316616268	WLS
ENSGALG00000013002	-0,420522444	0,734684533	DAP
ENSGALG00000005237	-0,426583983	-0,342909039	FGD3
ENSGALG00000005330	-0,431988153	1,328511787	SNCAIP
ENSGALG00000016328	-0,434072431	1,146952103	GSTA
ENSGALG00000026113	-0,434078932	0,523056553	CARHSP1
ENSGALG00000029174	-0,450699904	1,589321167	
ENSGALG00000006036	-0,461900797	0,644947298	
ENSGALG00000022926	-0,463163963	0,576442566	GPR162
ENSGALG00000017119	-0,467363832	-0,420708881	TNFRSF19
ENSGALG00000006885	-0,469804206	0,574664846	HRAS
ENSGALG00000010422	-0,470203966	0,646628908	FEZ1
ENSGALG00000007710	-0,471105348	-0,637223247	
ENSGALG00000027373	-0,47216803	-0,413207621	
ENSGALG00000015107	-0,4734568	0,542439326	PIP5K1B
ENSGALG00000012658	-0,475116315	1,214376265	TCP11L2
ENSGALG00000015660	-0,475673447	0,831570906	SNX30
ENSGALG00000000316	-0,476826949	0,549437781	PKNOX2
ENSGALG00000029011	-0,483499319	-0,444941401	SLC35F1
ENSGALG00000010316	-0,484012085	0,662660688	FRAS1
ENSGALG00000001966	-0,490465312	0,545673399	RP11-106M3.5
ENSGALG00000001816	-0,495397442	0,414935997	MAGI3
ENSGALG00000009207	-0,499904522	-0,518081559	TRIM2
ENSGALG00000026505	-0,503934058	0,837091894	RDH10
ENSGALG00000002777	-0,509947463	0,524514209	ACAA2
ENSGALG00000025977	-0,513209442	0,502103804	GADD45A
ENSGALG00000008728	-0,517913117	0,625207283	PTER
ENSGALG00000022713	-0,525946923	0,539793648	FAM181B
ENSGALG00000005246	-0,527306483	-0,7272373	PAX3

ENSGALG00000010982	-0,53129631	0,55035484	NID1
ENSGALG00000006480	-0,534658874	0,810690895	TCF7
ENSGALG00000016551	-0,548003277	1,321564075	AC025048.1
ENSGALG00000008433	-0,549541331	0,732487	FZD2
ENSGALG00000013964	-0,55034782	0,602864404	SPATA18
ENSGALG00000004323	-0,5545441	0,7637615	RASAL2
ENSGALG00000015987	-0,554680878	0,445391941	TP53INP1
ENSGALG00000008871	-0,555142332	-0,492812674	LPHN2
ENSGALG00000004593	-0,555306466	0,839985137	SULF2
ENSGALG00000014242	-0,557782152	0,583190799	BEND4
ENSGALG00000012710	-0,557841328	0,660750331	NCS1
ENSGALG00000004537	-0,559387674	0,454616982	EFNB1
ENSGALG00000028795	-0,566961474	1,118039364	CADM3
ENSGALG00000004741	-0,573167729	0,509578858	EPHB2
ENSGALG00000004256	-0,575998874	-0,396589984	DHRS3
ENSGALG00000003404	-0,585273519	1,24748878	TFEB
ENSGALG00000002707	-0,585668905	0,58133458	CHRNB2
ENSGALG00000008961	-0,593936394	-0,780468343	PTPRZ1
ENSGALG00000006409	-0,594856883	-0,436770571	PODXL
ENSGALG00000026180	-0,59981586	0,591162305	MSX2
ENSGALG00000012712	-0,607252424	0,742584862	RBM24
ENSGALG00000016251	-0,611728855	1,000647408	TSPAN7
ENSGALG00000002974	-0,611833543	-0,529999669	SPHKAP
ENSGALG00000008367	-0,614602858	0,522250337	MDK
ENSGALG00000010551	-0,614933753	0,911984879	CDC42EP3
ENSGALG00000002652	-0,621511478	-0,509052755	FZD10
ENSGALG00000003582	-0,622442826	-1,218636528	SLC1A3
ENSGALG00000007076	-0,624816157	0,617550884	CCNY
ENSGALG00000016391	-0,625110592	-0,569812042	CNKSR2
ENSGALG00000027377	-0,626128444	0,469161575	NKAIN3
ENSGALG00000015729	-0,630253804	0,747405326	LPAR1
ENSGALG00000007748	-0,63248212	0,758936347	SLC16A2
ENSGALG00000000040	-0,633128633	0,669045681	FAM195A
ENSGALG00000010320	-0,636363776	0,704383823	PAWR
ENSGALG00000006152	-0,63795304	0,657637784	
ENSGALG00000002437	-0,638363537	0,993124174	FAM69B
ENSGALG00000010269	-0,639329258	0,886301871	KCNAB1
ENSGALG00000006826	-0,641184602	-0,530661584	KLHL25
ENSGALG00000028543	-0,64250805	0,614319385	FGFR4
ENSGALG00000012577	-0,644333952	0,956309243	FRMD3
ENSGALG00000015241	-0,649652118	-0,655397927	SNAI2
ENSGALG00000017036	-0,649834759	1,353726483	LHFP
ENSGALG00000009439	-0,662995235	0,751125582	GPR85
ENSGALG00000005628	-0,664092027	0,601333554	COL9A3
ENSGALG00000005657	-0,664586696	0,780089243	CRHR2
ENSGALG00000007732	-0,671216647	1,306274335	RTN4R
ENSGALG00000010119	-0,672877793	0,900097678	CDKL2
ENSGALG00000004309	-0,673847887	0,913060877	GDAP1L1
ENSGALG00000005774	-0,674007323	-0,555555373	NKAIN4
ENSGALG00000017106	-0,674065701	1,082115941	ATP8A2
ENSGALG00000016945	-0,674331354	0,953551228	LECT1
ENSGALG00000006555	-0,681158051	-0,729109536	IGF2
ENSGALG00000001727	-0,689838866	1,445847057	LZTS1
ENSGALG00000023073	-0,692447843	0,353608475	AKAP12
ENSGALG00000009626	-0,694120539	-0,375704266	THBS1
ENSGALG00000003580	-0,694931491	1,284312011	MMP2
ENSGALG00000002818	-0,699438203	0,595523293	VAT1
ENSGALG00000003589	-0,699453771	0,652278144	VTN
ENSGALG00000006882	-0,700351611	0,976472658	ST8SIA2
ENSGALG00000004676	-0,710261932	0,910530695	BTG3
ENSGALG00000015926	-0,714450321	0,934566237	RUNX1T1
ENSGALG00000025919	-0,717706563	0,857090747	BAMBI

ENSGALG00000002016	-0,719060037	1,156233761	PRKCD
ENSGALG00000000674	-0,719912044	0,67836471	MYO1D
ENSGALG00000005608	-0,721159959	0,44662731	SLC9A3R2
ENSGALG00000002749	-0,723137568	0,687223145	RP11-565P22.6
ENSGALG00000001449	-0,723393814	0,740070536	STRA6
ENSGALG00000006792	-0,726229187	1,109047707	VSTM2L
ENSGALG00000009020	-0,742019908	-0,446477137	JAG1
ENSGALG00000009536	-0,742471798	0,851130737	
ENSGALG00000015448	-0,755416575	-0,533672096	TOX
ENSGALG00000005626	-0,756044449	0,92496767	TPST2
ENSGALG00000000690	-0,756893418	-0,657633862	GDPD4
ENSGALG00000015768	-0,757420183	0,855142896	ANKRD6
ENSGALG00000006793	-0,758190034	0,898163749	NTRK3
ENSGALG00000002742	-0,7644405	0,847416181	TMEM132B
ENSGALG00000015297	-0,764510224	0,81224071	FOXO3
ENSGALG00000016543	-0,766325509	0,654951402	NHS
ENSGALG00000006192	-0,767192565	0,958606154	USH1C
ENSGALG00000007623	-0,778310378	0,788173273	CACNA1G
ENSGALG00000026042	-0,780763307	0,987626868	SHISA3
ENSGALG00000012191	-0,787037882	0,72968259	LGR4
ENSGALG00000012652	-0,806982417	1,438231617	KCNG2
ENSGALG00000016223	-0,80722926	1,013131958	MAOB
ENSGALG00000004844	-0,807613149	0,581688828	SEMA6D
ENSGALG00000004986	-0,810825031	-0,430376609	CERCAM
ENSGALG00000004472	-0,814406115	0,672299012	ASTN1
ENSGALG00000017374	-0,814717413	1,149174538	SLITRK3
ENSGALG00000009514	-0,816414808	-0,779501664	NRCAM
ENSGALG00000012715	-0,842621875	0,978917656	CAP2
ENSGALG00000015084	-0,852219609	1,480304896	IGSF11
ENSGALG00000006873	-0,854385087	0,565449117	RASSF7
ENSGALG00000009052	-0,861077306	0,987910707	LBH
ENSGALG00000026192	-0,861152656	-0,473341595	ST8SIA4
ENSGALG00000028946	-0,867278013	1,217039733	CSRP2
ENSGALG00000008297	-0,875877387	0,379216929	SEMA4B
ENSGALG00000011709	-0,876476185	0,918333355	TMEM108
ENSGALG00000003345	-0,878084006	1,211546204	PLCD4
ENSGALG00000023738	-0,882263773	0,704601132	
ENSGALG00000027501	-0,88631831	1,108782799	ANPEP
ENSGALG00000003426	-0,891859274	-0,587203746	RHBDL3
ENSGALG00000001129	-0,899923251	0,871868135	KIRREL3
ENSGALG00000015186	-0,900331794	1,345677898	KLHL14
ENSGALG00000006245	-0,900397348	1,589094556	KLF15
ENSGALG00000009603	-0,909672796	0,844955114	
ENSGALG00000028234	-0,912907561	1,03829388	SYNDIG1
ENSGALG00000015967	-0,91657646	0,936813576	LRRTM1
ENSGALG00000020688	-0,921010154	0,678030575	CYP4B1L
ENSGALG00000020376	-0,925271568	0,781084057	
ENSGALG00000007078	-0,925877896	1,270778013	ENTPD1
ENSGALG00000005539	-0,927364348	0,77704495	ITM2C
ENSGALG00000006346	-0,927790987	0,756405715	CXCL14
ENSGALG00000020575	-0,930944308	0,690014438	ERMN
ENSGALG00000009237	-0,942598283	1,124183808	TLR2A
ENSGALG00000009042	-0,948736093	0,719468223	ISM1
ENSGALG00000004849	-0,955100006	0,553445556	WSCD2
ENSGALG00000011200	-0,962880109	1,609360966	THBS2
ENSGALG00000006507	-0,967854239	0,793097237	CLMP
ENSGALG00000026153	-0,984548638	0,755983234	FOXO6
ENSGALG00000001795	-0,995172186	1,261084777	GFRA2
ENSGALG00000002832	-0,995334878	1,610990532	IFI35
ENSGALG00000019280	-1,005672086	1,199070585	LMO3
ENSGALG00000010500	-1,016915026	0,828235743	NKX6-2
ENSGALG00000027891	-1,030686173	0,504324315	NREP

ENSGALG00000020788	-1,041075867	-0,986044977	CCDC141
ENSGALG00000008455	-1,05482755	0,695894902	SEMA3C
ENSGALG00000009601	-1,061491773	0,878730197	NELL2
ENSGALG00000003588	-1,081475528	0,911216657	
ENSGALG00000009638	-1,085140036	0,988233263	ANO6
ENSGALG00000007908	-1,09305059	1,477721596	EFEMP1
ENSGALG00000013948	-1,099632212	0,760675086	RASL11B
ENSGALG00000028682	-1,101427497	0,990483134	
ENSGALG00000008440	-1,108083257	1,543692993	CHST1
ENSGALG00000007211	-1,108214376	1,44654086	CDH22
ENSGALG00000028506	-1,110787668	0,637581477	
ENSGALG00000002028	-1,145532965	0,675138088	GPRC5B
ENSGALG00000004448	-1,158751146	1,504790559	BRINP2
ENSGALG00000005967	-1,230002366	1,545091384	LMO1
ENSGALG00000014258	-1,256488908	1,040436704	LIMCH1
ENSGALG00000023577	-1,257090663	2,257832359	TBX2
ENSGALG00000016411	-1,262862187	0,657856223	COL14A1
ENSGALG00000009604	-1,349943691	0,581479374	DBX2
ENSGALG00000011205	-1,462754207	0,836341019	SMOC2
