# Supplementary material for: HOXB8 Counteracts MAPK/ERK Oncogenic Signaling in a Chicken Embryo Model of Neoplasia
Source: Int J Mol Sci. 2021 Aug 18;22(16):8911. doi: 10.3390/ijms22168911 (PMC8396257; doi:10.3390/ijms22168911)
Supplement: Supplementary file 1 [file ijms-22-08911-s001.zip › ijms-1277141 Table S4.pdf]

Table S4: MEK1ca+HOXB8 versus MEK1ca differentially regulated genes (False Discovery Rate (padj) <= 0.05).

ID	baseMean	log2FoldChange	lfcSE	stat	pvalue	padj	Symbol
ENSGALG00000026853	1756,7605	5,751530736	0,1962667	29,304673	9,04E-189	6,16E-185	FGF3
ENSGALG00000011433	901,86118	4,353428748	0,1979937	21,987719	3,77E-107	1,28E-103	VIL1
ENSGALG00000009778	267,92271	4,29765073	0,2973351	14,453898	2,37E-47	9,77E-45	HCLS1
ENSGALG00000011582	175,17021	4,101846269	0,3182796	12,887557	5,29E-38	1,53E-35	SLC17A8
ENSGALG00000027742	312,3427	3,96517198	0,2663087	14,889379	3,86E-50	1,81E-47	KLHL33
ENSGALG00000028044	194,51466	3,90733608	0,3041023	12,848755	8,74E-38	2,48E-35	ADMP
ENSGALG00000002108	813,37463	3,799830272	0,1965883	19,328872	3,07E-83	5,23E-80	EVPL
ENSGALG00000021395	138,40693	3,681673719	0,3287873	11,197736	4,18E-29	6,70E-27	
ENSGALG00000001080	589,09501	3,620989798	0,2231106	16,229571	3,12E-59	2,02E-56	NR5A1
ENSGALG00000005292	233,99511	3,593969871	0,2819274	12,747854	3,20E-37	8,90E-35	KLHL40
ENSGALG00000028376	1521,8358	3,47510163	0,1982878	17,525546	9,15E-69	7,78E-66	FGF19
ENSGALG00000023843	120,84193	3,393126537	0,3307759	10,258084	1,09E-24	1,35E-22	FRMD7
ENSGALG00000006471	279,29896	3,309144864	0,2889251	11,453298	2,26E-30	4,04E-28	SLCO2A1
ENSGALG00000028304	118,7931	3,251722149	0,3347604	9,7135804	2,64E-22	2,58E-20	
ENSGALG00000004526	421,69106	3,217263436	0,2437389	13,199628	8,82E-40	2,93E-37	TNR
ENSGALG00000008069	109,41167	3,182915984	0,3413839	9,3235675	1,12E-20	9,51E-19	CYP2J6L3
ENSGALG00000004134	133,43428	3,164728278	0,3267271	9,6861515	3,45E-22	3,31E-20	GLRA1
ENSGALG00000012254	87,731989	3,162704218	0,3459847	9,1411679	6,18E-20	4,98E-18	KCNJ4
ENSGALG00000006944	469,14467	3,116432937	0,2398381	12,993902	1,32E-38	4,01E-36	
ENSGALG00000028612	103,60478	3,070056483	0,3464928	8,8603749	7,97E-19	5,81E-17	MYH1G
ENSGALG00000005285	100,23743	2,996752626	0,3402533	8,8074179	1,28E-18	8,94E-17	TBX4
ENSGALG00000007335	107,29888	2,985281892	0,3405577	8,7658615	1,85E-18	1,28E-16	KBTBD13
ENSGALG00000001256	266,94499	2,963677659	0,2979331	9,9474608	2,59E-23	2,71E-21	NLRC5
ENSGALG00000016685	93,447456	2,945393761	0,3383685	8,7046936	3,18E-18	2,13E-16	ASMT
ENSGALG00000016721	160,3282	2,940591405	0,3042022	9,6665674	4,18E-22	3,93E-20	PLCXD1
ENSGALG00000026167	171,61409	2,925839411	0,3177833	9,2070258	3,35E-20	2,78E-18	PIK3R5
ENSGALG00000002926	76,22895	2,900739748	0,3500461	8,2867354	1,16E-16	6,86E-15	DBH
ENSGALG00000004700	97,840654	2,88173675	0,3343593	8,6186822	6,77E-18	4,45E-16	NCF2
ENSGALG00000016722	73,43112	2,829128945	0,3527315	8,0206314	1,05E-15	5,52E-14	RGNL
ENSGALG00000012830	227,95073	2,760094911	0,2851044	9,6809972	3,63E-22	3,44E-20	IRF4
ENSGALG00000027247	97,436329	2,75413056	0,3327446	8,2770098	1,26E-16	7,41E-15	EOMES
ENSGALG00000002823	276,03395	2,746001119	0,2565217	10,70475	9,67E-27	1,39E-24	
ENSGALG00000006614	540,92661	2,721769719	0,2601033	10,464189	1,26E-25	1,70E-23	TECTA
ENSGALG00000006744	446,95073	2,709854582	0,2639156	10,267881	9,83E-25	1,23E-22	MUC2
ENSGALG00000020485	352,82533	2,707785881	0,2864956	9,4514033	3,34E-21	2,90E-19	
ENSGALG00000015322	401,95748	2,694878773	0,2468716	10,916117	9,65E-28	1,43E-25	IMPG2
ENSGALG00000025886	234,93273	2,681244585	0,2769653	9,6807963	3,64E-22	3,44E-20	SUSD3
ENSGALG00000013922	145,69265	2,651462599	0,3144764	8,4313551	3,42E-17	2,11E-15	ZP1
ENSGALG00000026590	256,39028	2,649598672	0,2655285	9,9785844	1,89E-23	2,01E-21	KLF13
ENSGALG00000016325	669,81495	2,632246576	0,2229691	11,805432	3,66E-32	7,66E-30	GSTA3
ENSGALG00000026627	138,55984	2,620280418	0,325291	8,0551894	7,94E-16	4,29E-14	
ENSGALG00000001780	98,3971	2,613843166	0,3358627	7,7824758	7,11E-15	3,32E-13	PROC
ENSGALG00000002987	118,45636	2,607861451	0,3206775	8,1323505	4,21E-16	2,34E-14	SERPINF2
ENSGALG00000027059	200,39104	2,606173672	0,2890015	9,0178542	1,92E-19	1,48E-17	
ENSGALG00000023821	140,66943	2,605691323	0,3149662	8,2729246	1,31E-16	7,64E-15	DHX58
ENSGALG00000006032	296,77177	2,599165832	0,2722516	9,5469253	1,34E-21	1,19E-19	IL13RA1
ENSGALG00000002960	103,93249	2,574242623	0,3355369	7,6720094	1,69E-14	7,76E-13	HMGCS2
ENSGALG00000010841	110,20084	2,5587632	0,3321477	7,7036919	1,32E-14	6,10E-13	
ENSGALG00000002829	649,13345	2,553310959	0,2049577	12,457744	1,27E-35	3,20E-33	SCARF1
ENSGALG00000021198	61,503229	2,552636185	0,3511007	7,2703825	3,58E-13	1,39E-11	
ENSGALG00000015141	58,962649	2,548558749	0,3520748	7,2386848	4,53E-13	1,74E-11	GDA
ENSGALG00000005122	338,26914	2,528381619	0,242755	10,415364	2,11E-25	2,79E-23	MYO1H
ENSGALG00000010145	278,24749	2,508391386	0,3014197	8,3219221	8,65E-17	5,15E-15	MDGA1
ENSGALG00000014372	67,978413	2,495880293	0,3492839	7,1457073	8,95E-13	3,29E-11	SLC34A2
ENSGALG00000029144	72,328613	2,46980754	0,3488262	7,0803381	1,44E-12	5,17E-11	
ENSGALG00000021399	89,329511	2,464358792	0,336861	7,3156547	2,56E-13	1,01E-11	ABCA8
ENSGALG00000001111	752,30516	2,451755117	0,2113059	11,602872	3,98E-31	7,64E-29	DNAH9
ENSGALG00000012985	261,61267	2,44205884	0,2658681	9,185228	4,11E-20	3,39E-18	SLC6A13
ENSGALG00000008105	47,87761	2,404806479	0,3539579	6,7940461	1,09E-11	3,44E-10	CYP2J6L2

ENSGALG00000012812	176,06145	2,39422427	0,2959701	8,089412	6,00E-16	3,29E-14	SVOPL
ENSGALG00000002638	117,91406	2,388124012	0,3283139	7,2739052	3,49E-13	1,35E-11	SRRL
ENSGALG00000002318	63,409663	2,376044719	0,3494777	6,7988449	1,05E-11	3,33E-10	LVRN
ENSGALG00000002015	214,32321	2,374226325	0,2864093	8,2896265	1,14E-16	6,72E-15	SNCG
ENSGALG00000026256	199,83767	2,357924834	0,3332057	7,0764832	1,48E-12	5,30E-11	
ENSGALG00000007395	402,67751	2,356587415	0,2261671	10,419672	2,02E-25	2,69E-23	ABCC2
ENSGALG00000002046	463,72169	2,341658671	0,2422889	9,6647372	4,26E-22	3,97E-20	SYT15
ENSGALG00000007340	53,630639	2,338672246	0,3529687	6,6257213	3,46E-11	1,01E-09	TMPRSS5
ENSGALG00000023934	52,57595	2,337998666	0,3538351	6,6075938	3,91E-11	1,12E-09	
ENSGALG00000028341	324,38787	2,327426751	0,2360199	9,8611477	6,13E-23	6,23E-21	MADCAM1
ENSGALG00000002260	279,41384	2,326075861	0,254913	9,1249794	7,17E-20	5,71E-18	CISH
ENSGALG00000006402	199,28187	2,315345967	0,2776938	8,3377677	7,57E-17	4,56E-15	NRROS
ENSGALG00000008709	98,437291	2,291854243	0,3268395	7,0121701	2,35E-12	8,17E-11	MCOLN3
ENSGALG00000013006	357,98483	2,275189997	0,2579484	8,8203283	1,14E-18	8,05E-17	ROPN1L
ENSGALG00000006992	182,22358	2,270300547	0,2984455	7,6070847	2,80E-14	1,27E-12	MMP9
ENSGALG00000011113	527,00037	2,26759493	0,2713629	8,356319	6,47E-17	3,93E-15	SGIP1
ENSGALG00000023577	321,7197	2,257832359	0,2669922	8,4565486	2,75E-17	1,74E-15	TBX2
ENSGALG00000016852	56,030921	2,256531595	0,3516922	6,416211	1,40E-10	3,74E-09	TNFSF13B
ENSGALG00000027617	219,45804	2,255993416	0,2851339	7,9120503	2,53E-15	1,25E-13	RP11-385J1.3
ENSGALG00000003677	113,60782	2,250263529	0,3449597	6,5232654	6,88E-11	1,92E-09	NTNG2
ENSGALG00000023742	249,60413	2,24719921	0,2820489	7,9674098	1,62E-15	8,26E-14	
ENSGALG00000001589	111,25767	2,246033051	0,3441573	6,526182	6,75E-11	1,89E-09	INPP5D
ENSGALG00000016369	952,12228	2,242110682	0,1770401	12,664423	9,31E-37	2,54E-34	FBXO32
ENSGALG00000027703	404,86881	2,238234147	0,2535546	8,8274262	1,07E-18	7,64E-17	
ENSGALG00000003389	248,60083	2,230534118	0,2705236	8,2452465	1,65E-16	9,51E-15	IFI30
ENSGALG00000015244	123,74828	2,228224412	0,3204639	6,9531211	3,57E-12	1,20E-10	TBX19
ENSGALG00000001857	255,91037	2,203449137	0,2595043	8,4909939	2,05E-17	1,31E-15	FAM132A
ENSGALG00000019861	47,604267	2,196772492	0,3539452	6,2065321	5,42E-10	1,33E-08	
ENSGALG00000008514	959,21197	2,19329695	0,1769465	12,395254	2,77E-35	6,86E-33	DLL4
ENSGALG00000021714	88,405928	2,185372855	0,3343395	6,5363894	6,30E-11	1,78E-09	PADI1
ENSGALG00000009499	136,98531	2,185164296	0,3152046	6,932526	4,13E-12	1,37E-10	NPY5R
ENSGALG00000026467	67,174686	2,184133106	0,3474873	6,2855052	3,27E-10	8,22E-09	MESP1
ENSGALG00000027532	181,56516	2,181261094	0,3353331	6,5047583	7,78E-11	2,16E-09	COLEC11
ENSGALG00000002817	43,721989	2,174112777	0,3531294	6,1567025	7,43E-10	1,79E-08	CPLX4
ENSGALG00000028339	572,22079	2,143746023	0,2535634	8,4544787	2,80E-17	1,76E-15	AMIGO3
ENSGALG00000003548	717,97931	2,131798574	0,1846233	11,546747	7,67E-31	1,43E-28	BAIAP2L1
ENSGALG00000029168	574,12283	2,125452005	0,2633801	8,069905	7,04E-16	3,85E-14	TMEM27
ENSGALG00000005062	695,37715	2,112402161	0,1891794	11,166133	5,97E-29	9,35E-27	FOXN4
ENSGALG00000001879	730,68274	2,106018407	0,1838916	11,4525	2,28E-30	4,04E-28	GRID1
ENSGALG00000028551	2508,945	2,105237568	0,1772334	11,878334	1,53E-32	3,32E-30	
ENSGALG00000001696	267,69848	2,102610472	0,2564004	8,2004945	2,39E-16	1,36E-14	SAG
ENSGALG00000008275	48,571485	2,098791425	0,3539549	5,9295439	3,04E-09	6,67E-08	MESP2
ENSGALG00000021567	284,86115	2,081191963	0,246615	8,4390326	3,20E-17	1,99E-15	EPHA8
ENSGALG00000001874	67,244987	2,080511409	0,3538919	5,8789453	4,13E-09	8,88E-08	
ENSGALG00000028654	92,92864	2,074935686	0,3457662	6,0009788	1,96E-09	4,45E-08	
ENSGALG00000006260	56,956461	2,062296513	0,3527945	5,8456027	5,05E-09	1,06E-07	KLHL30
ENSGALG00000007570	256,24389	2,055518081	0,2571315	7,9940356	1,31E-15	6,73E-14	KLF8
ENSGALG00000019527	38,897028	2,052781357	0,3521947	5,8285416	5,59E-09	1,16E-07	SLC6A19
ENSGALG00000000656	95,76214	2,036711968	0,3414768	5,964423	2,46E-09	5,51E-08	TMEM81
ENSGALG00000007104	125,61776	2,032418742	0,3200563	6,3501909	2,15E-10	5,59E-09	LRRC15
ENSGALG00000026541	175,99093	2,028653787	0,2976967	6,8144995	9,46E-12	3,00E-10	
ENSGALG00000014203	138,12328	2,025939839	0,3005882	6,7399175	1,58E-11	4,87E-10	PARVG
ENSGALG00000020195	143,5922	2,021370929	0,3033718	6,6630159	2,68E-11	7,92E-10	DMP1
ENSGALG00000012204	102,97928	2,019423041	0,32821	6,1528374	7,61E-10	1,83E-08	FAM20CL
ENSGALG00000005797	87,794264	2,014484064	0,3341899	6,0279615	1,66E-09	3,83E-08	COL20A1
ENSGALG00000000437	37,093294	2,013731373	0,3518291	5,7236071	1,04E-08	2,04E-07	
ENSGALG00000002931	53,692699	2,013598707	0,3534025	5,6977488	1,21E-08	2,37E-07	REG4
ENSGALG00000006273	107,39879	2,011386223	0,3229782	6,2276225	4,74E-10	1,16E-08	MYLK2
ENSGALG00000000722	46,564331	2,006522785	0,3537549	5,6720703	1,41E-08	2,72E-07	
ENSGALG00000028147	229,82466	2,001096858	0,2664367	7,5105891	5,89E-14	2,49E-12	CTSK
ENSGALG00000004248	56,901253	1,998749091	0,352065	5,6772168	1,37E-08	2,65E-07	TACR2
ENSGALG00000003075	93,078833	1,997443704	0,3325728	6,0060344	1,90E-09	4,34E-08	SLC34A1

ENSGALG00000001571	57,11691	1,994083312	0,35177	5,6687129	1,44E-08	2,77E-07	MYO1F
ENSGALG00000010458	184,3782	1,990178775	0,2802188	7,1022313	1,23E-12	4,45E-11	NOXRED1
ENSGALG00000011889	230,92302	1,988400853	0,2676563	7,4289338	1,09E-13	4,48E-12	RP11-47I22.4
ENSGALG00000010924	209,96874	1,986898996	0,2874232	6,9127999	4,75E-12	1,56E-10	NFIA
ENSGALG00000015846	313,73062	1,982793598	0,2465855	8,0409991	8,91E-16	4,78E-14	SNAP91
ENSGALG00000006835	112,62877	1,974805269	0,3171624	6,2264797	4,77E-10	1,17E-08	TNNC2
ENSGALG00000013253	157,86205	1,972349113	0,3198156	6,1671455	6,95E-10	1,68E-08	INSRR
ENSGALG00000009302	367,16268	1,971912015	0,2879884	6,8471929	7,53E-12	2,42E-10	EMX2
ENSGALG00000004414	101,95784	1,959564216	0,3212322	6,1001479	1,06E-09	2,50E-08	LUZP2
ENSGALG00000009489	127,74049	1,956908773	0,320476	6,1062578	1,02E-09	2,42E-08	FSTL5
ENSGALG00000005822	147,05646	1,951526015	0,3027027	6,447005	1,14E-10	3,07E-09	KCNQ2
ENSGALG00000027147	228,05825	1,950896881	0,2801413	6,9639743	3,31E-12	1,13E-10	SUB1L1
ENSGALG00000021869	645,94199	1,950468731	0,2435545	8,0083473	1,16E-15	6,04E-14	PODN
ENSGALG00000028975	532,93063	1,945491073	0,2058434	9,4513148	3,35E-21	2,90E-19	FRMPD3
ENSGALG00000007666	61,970313	1,945055405	0,3514319	5,5346576	3,12E-08	5,56E-07	KCNIP2
ENSGALG00000021656	37,511821	1,943986445	0,3507973	5,5416231	3,00E-08	5,37E-07	PTPN22
ENSGALG00000008188	164,94281	1,939910796	0,2907086	6,6730415	2,51E-11	7,46E-10	TRIM36
ENSGALG00000010362	56,130567	1,930559856	0,3538155	5,456402	4,86E-08	8,33E-07	SUCNR1
ENSGALG00000000560	58,758167	1,925570368	0,353335	5,4497022	5,05E-08	8,61E-07	PI16
ENSGALG00000015021	44,681844	1,923323022	0,3539578	5,4337643	5,52E-08	9,36E-07	TMEM174
ENSGALG00000015551	1931,1282	1,921428632	0,1720637	11,16696	5,92E-29	9,35E-27	GRIN3A
ENSGALG00000010867	80,266993	1,919026004	0,3407804	5,6312691	1,79E-08	3,37E-07	TACSTD2
ENSGALG00000001004	271,27145	1,914258624	0,2596069	7,3736827	1,66E-13	6,69E-12	MPO
ENSGALG00000011347	48,911798	1,909599526	0,3539061	5,3957801	6,82E-08	1,14E-06	IHH
ENSGALG00000006413	117,16579	1,90767902	0,3112895	6,1283111	8,88E-10	2,11E-08	CEMIP
ENSGALG00000003836	138,24842	1,907173269	0,3450544	5,5271675	3,25E-08	5,78E-07	RBM44
ENSGALG00000007345	111,97256	1,905774604	0,3245679	5,8717287	4,31E-09	9,20E-08	TMEM114
ENSGALG00000008701	97,136553	1,905747917	0,3400514	5,6042938	2,09E-08	3,87E-07	XDH
ENSGALG00000010853	484,76878	1,904256394	0,2172677	8,7645617	1,88E-18	1,29E-16	C8B
ENSGALG00000000221	51,676802	1,900065815	0,35396	5,3680237	7,96E-08	1,31E-06	PROM2
ENSGALG00000001617	1078,3293	1,898502237	0,2123138	8,9419639	3,82E-19	2,86E-17	CAMKK1
ENSGALG00000019290	78,123837	1,892925943	0,3496984	5,4130245	6,20E-08	1,04E-06	SLC6A12
ENSGALG00000009265	88,174325	1,891791729	0,3459146	5,4689565	4,53E-08	7,80E-07	KCNK18
ENSGALG00000003923	2335,3387	1,889033194	0,1503705	12,562527	3,39E-36	8,88E-34	COL6A3
ENSGALG00000008995	104,54637	1,882652598	0,3282959	5,7346211	9,77E-09	1,93E-07	MAMDC4
ENSGALG00000000544	347,72624	1,877518925	0,2328757	8,0623238	7,49E-16	4,08E-14	
ENSGALG00000007706	86,703491	1,877381913	0,3347682	5,6080061	2,05E-08	3,82E-07	FGF8
ENSGALG00000015347	73,173206	1,876981559	0,3445279	5,4479816	5,09E-08	8,66E-07	ZPLD1
ENSGALG00000009078	124,06611	1,868595491	0,3338294	5,5974556	2,18E-08	4,01E-07	GALNT14
ENSGALG00000000899	303,59428	1,867380532	0,252928	7,3830526	1,55E-13	6,29E-12	PSD2
ENSGALG00000023459	62,523363	1,863475253	0,3506341	5,314586	1,07E-07	1,71E-06	MC1R
ENSGALG00000013953	153,51081	1,853929495	0,316158	5,8639338	4,52E-09	9,60E-08	TACR1
ENSGALG00000020316	66,987938	1,852228829	0,3437054	5,3890016	7,09E-08	1,17E-06	IL13RA2
ENSGALG00000014770	4689,6836	1,850573228	0,1917177	9,6525964	4,79E-22	4,41E-20	EPB41L3
ENSGALG00000015406	32,824277	1,850052547	0,3495416	5,2927962	1,20E-07	1,90E-06	MOS
ENSGALG00000007047	78,987356	1,844593336	0,3380656	5,4563178	4,86E-08	8,33E-07	GAL
ENSGALG00000011421	155,78727	1,840566258	0,2941943	6,2562953	3,94E-10	9,81E-09	HAL
ENSGALG00000003751	52,948026	1,835104614	0,3538037	5,186787	2,14E-07	3,22E-06	ASAH2
ENSGALG00000021693	69,312923	1,830329106	0,3430516	5,3354333	9,53E-08	1,54E-06	
ENSGALG00000005634	92,232146	1,826798031	0,3274114	5,5795183	2,41E-08	4,40E-07	CRYBA4
ENSGALG00000005644	54,639892	1,819377424	0,3504577	5,1914323	2,09E-07	3,15E-06	MYO1G
ENSGALG00000029006	89,819678	1,803532169	0,3296621	5,4708508	4,48E-08	7,73E-07	SYPL1
ENSGALG00000010126	116,19587	1,799221778	0,3298899	5,4540074	4,92E-08	8,42E-07	BEST4
ENSGALG00000019361	44,228613	1,792121861	0,3538597	5,0644988	4,09E-07	5,78E-06	FAM19A2
ENSGALG00000020982	53,872261	1,789263062	0,3513204	5,092966	3,53E-07	5,05E-06	BPIFB3
ENSGALG00000004841	236,70913	1,788099107	0,2763588	6,4702084	9,79E-11	2,66E-09	APLNR
ENSGALG00000008845	211,0907	1,784489653	0,2697983	6,6141623	3,74E-11	1,08E-09	HAO1
ENSGALG00000023354	87,321322	1,782015396	0,3447223	5,1694224	2,35E-07	3,49E-06	FSCN2
ENSGALG00000010364	75,712359	1,777216343	0,3496501	5,0828426	3,72E-07	5,29E-06	AADACL2
ENSGALG00000027090	122,23539	1,772101833	0,3238631	5,4717629	4,46E-08	7,70E-07	
ENSGALG00000005043	168,0345	1,771501032	0,2886982	6,13617	8,45E-10	2,02E-08	ACACB
ENSGALG00000005747	39,000743	1,767653479	0,3536042	4,998961	5,76E-07	7,89E-06	

ENSGALG00000014252	107,13553	1,765576737	0,346339	5,0978281	3,44E-07	4,94E-06	
ENSGALG00000006007	80,52031	1,763247242	0,3431655	5,1381839	2,77E-07	4,06E-06	SAMD10
ENSGALG00000000630	44,986023	1,762139369	0,352772	4,9951231	5,88E-07	8,02E-06	HAS3
ENSGALG00000013971	623,6268	1,754983652	0,2217786	7,9132227	2,51E-15	1,24E-13	SGK1
ENSGALG00000025804	57,829823	1,753854275	0,3486801	5,0299803	4,91E-07	6,84E-06	CLCN2
ENSGALG00000016233	282,39076	1,751740847	0,2591609	6,7592786	1,39E-11	4,28E-10	TG
ENSGALG00000000299	146,62252	1,750555222	0,3173752	5,5157274	3,47E-08	6,13E-07	PKP1
ENSGALG00000015154	107,54458	1,749162012	0,3192627	5,4787551	4,28E-08	7,43E-07	TRPM6
ENSGALG00000028801	326,04496	1,749152851	0,2317152	7,548717	4,40E-14	1,90E-12	YPEL2
ENSGALG00000003103	147,01296	1,745350116	0,2934725	5,9472356	2,73E-09	6,09E-08	MST1R
ENSGALG00000010408	48,693762	1,74244975	0,3525174	4,9428754	7,70E-07	1,02E-05	OPN4-1
ENSGALG00000016580	156,41685	1,740943756	0,3137577	5,5486878	2,88E-08	5,20E-07	SCARA5
ENSGALG00000020779	287,43405	1,737619419	0,2447032	7,1009277	1,24E-12	4,48E-11	
ENSGALG00000015544	64,576519	1,719200832	0,3511965	4,895268	9,82E-07	1,28E-05	ALDOB
ENSGALG00000015305	121,8353	1,715527315	0,3092461	5,547451	2,90E-08	5,21E-07	NR2E1
ENSGALG00000008926	1594,7541	1,713961304	0,1563378	10,963191	5,74E-28	8,69E-26	CADPS2
ENSGALG00000002957	403,40008	1,713056332	0,2361807	7,2531612	4,07E-13	1,57E-11	SLC12A3
ENSGALG00000017212	76,54735	1,710911672	0,3424068	4,9967228	5,83E-07	7,97E-06	GPR83
ENSGALG00000004827	60,764569	1,70800582	0,3466264	4,9275126	8,33E-07	1,10E-05	PAK6
ENSGALG00000003242	570,22011	1,707949225	0,2101315	8,1280036	4,36E-16	2,42E-14	STAR
ENSGALG00000001912	146,49438	1,699528733	0,3012842	5,6409491	1,69E-08	3,21E-07	EPHA10
ENSGALG00000003003	42,669769	1,694427776	0,3520315	4,8132841	1,48E-06	1,86E-05	CCL20
ENSGALG00000016261	158,70666	1,693178751	0,3145024	5,3836747	7,30E-08	1,20E-06	CYBB
ENSGALG00000011007	1150,329	1,688733669	0,1907765	8,8518972	8,60E-19	6,20E-17	OSBPL3
ENSGALG00000015117	149,51437	1,688648354	0,2954854	5,7148283	1,10E-08	2,15E-07	DRD3
ENSGALG00000012429	6013,8279	1,680823476	0,1520104	11,05729	2,02E-28	3,09E-26	BMP4
ENSGALG00000000458	427,66514	1,679795969	0,2174489	7,7250163	1,12E-14	5,20E-13	TMPRSS9
ENSGALG00000011622	28,65156	1,67904306	0,3460309	4,8522925	1,22E-06	1,56E-05	MAS1
ENSGALG00000016572	77,200694	1,678816771	0,3434432	4,8881939	1,02E-06	1,32E-05	C3H8ORF80
ENSGALG00000001084	353,56487	1,677708871	0,2418689	6,9364397	4,02E-12	1,34E-10	ATP8B3
ENSGALG00000026473	1522,1511	1,674638021	0,2112768	7,9262761	2,26E-15	1,13E-13	CHST11
ENSGALG00000008178	111,71167	1,67186128	0,3225826	5,1827379	2,19E-07	3,28E-06	CYP2J2L5
ENSGALG00000005140	240,21489	1,671709253	0,257977	6,48007	9,17E-11	2,52E-09	RRAD
ENSGALG00000001234	40,862653	1,668621796	0,3539185	4,7147069	2,42E-06	2,87E-05	CETP
ENSGALG00000006514	1126,0055	1,665636549	0,1751467	9,5099504	1,91E-21	1,69E-19	FSTL4
ENSGALG00000004936	57,472904	1,658043102	0,3494689	4,744466	2,09E-06	2,51E-05	GLRA4
ENSGALG00000028174	599,32727	1,656109672	0,2408103	6,877239	6,10E-12	1,98E-10	SDPR
ENSGALG00000011882	446,5677	1,655648124	0,2116439	7,8228016	5,17E-15	2,44E-13	CDCP1
ENSGALG00000020143	148,7819	1,654526791	0,3218985	5,1399029	2,75E-07	4,02E-06	GABRG1
ENSGALG00000027314	27,97681	1,648583002	0,3452618	4,7748783	1,80E-06	2,19E-05	SAXO1
ENSGALG00000019019	89,671454	1,647447024	0,3439031	4,7904393	1,66E-06	2,05E-05	NTRK1
ENSGALG00000026161	123,20926	1,647025641	0,3125278	5,2700132	1,36E-07	2,13E-06	
ENSGALG00000024298	32,030697	1,646495761	0,3488596	4,7196511	2,36E-06	2,81E-05	ADAMTSL5
ENSGALG00000004394	167,65918	1,640660466	0,2837407	5,7822521	7,37E-09	1,49E-07	PCBP3
ENSGALG00000016262	396,40337	1,640510999	0,2287869	7,1704758	7,47E-13	2,77E-11	XK
ENSGALG00000015905	84,004407	1,632452898	0,3358609	4,860503	1,17E-06	1,51E-05	SLC7A7
ENSGALG00000005293	115,92754	1,631397185	0,3272119	4,9857512	6,17E-07	8,36E-06	GNG13
ENSGALG00000025946	137,58478	1,630278266	0,3118772	5,2273088	1,72E-07	2,64E-06	MOB3C
ENSGALG00000011806	33,35332	1,625032877	0,3475057	4,6762767	2,92E-06	3,39E-05	MUC13
ENSGALG00000010721	516,48932	1,61713725	0,1982525	8,1569561	3,44E-16	1,92E-14	GLIS1
ENSGALG00000008133	122,89128	1,61551597	0,3252043	4,9676957	6,78E-07	9,10E-06	AS3MT
ENSGALG00000000820	127,58617	1,614544959	0,3185699	5,068102	4,02E-07	5,68E-06	HTR1D
ENSGALG00000002832	501,68162	1,610990532	0,2127694	7,5715338	3,69E-14	1,62E-12	IFI35
ENSGALG00000006606	3620,8517	1,610976489	0,1224776	13,153237	1,63E-39	5,29E-37	KIAA1324L
ENSGALG00000011200	907,82877	1,609360966	0,246667	6,5244282	6,83E-11	1,91E-09	THBS2
ENSGALG00000007980	258,34488	1,607363167	0,2746243	5,8529528	4,83E-09	1,02E-07	KCNG1
ENSGALG00000001062	680,57905	1,606326071	0,191836	8,3734359	5,60E-17	3,43E-15	GGT7
ENSGALG00000003106	661,98127	1,604428011	0,2222828	7,217957	5,28E-13	2,01E-11	TOX2
ENSGALG00000007815	333,86581	1,603027021	0,2797129	5,7309725	9,99E-09	1,96E-07	ANKK1
ENSGALG00000015366	80,07277	1,602600172	0,3432645	4,6687031	3,03E-06	3,52E-05	
ENSGALG00000005725	91,108572	1,601523904	0,352027	4,5494347	5,38E-06	5,90E-05	CSF1R
ENSGALG00000022644	26,874228	1,595235245	0,3437941	4,6400892	3,48E-06	3,98E-05	CCDC36

ENSGALG00000007759	856,86635	1,594985498	0,1989057	8,0188035	1,07E-15	5,57E-14	TMEFF2
ENSGALG00000008830	781,04938	1,591806155	0,2114024	7,5297432	5,08E-14	2,17E-12	BMP2
ENSGALG00000006412	53,058714	1,591456503	0,3527925	4,5110267	6,45E-06	6,90E-05	VGLL1
ENSGALG00000029174	5348,1501	1,589321167	0,1290901	12,311717	7,83E-35	1,87E-32	
ENSGALG00000006245	402,97003	1,589094556	0,227751	6,9773328	3,01E-12	1,04E-10	KLF15
ENSGALG00000026733	77,5006	1,588089103	0,3406644	4,6617403	3,14E-06	3,62E-05	FFAR4
ENSGALG00000016138	93,251278	1,581494384	0,3296626	4,7973118	1,61E-06	2,00E-05	DSCAM
ENSGALG00000016498	94,791705	1,580375213	0,3294934	4,7963786	1,62E-06	2,01E-05	MFSD2B
ENSGALG00000011343	80,565753	1,579403764	0,3348001	4,7174527	2,39E-06	2,83E-05	SLC23A3
ENSGALG00000000733	1063,131	1,578595816	0,1987694	7,941845	1,99E-15	1,00E-13	MYO7A
ENSGALG00000028175	72,934835	1,575180638	0,35396	4,4501653	8,58E-06	9,01E-05	GJA9
ENSGALG00000015293	333,00466	1,574970296	0,2444462	6,4430147	1,17E-10	3,15E-09	TMEM45A
ENSGALG00000012115	228,29439	1,572479232	0,2734467	5,7505884	8,89E-09	1,77E-07	
ENSGALG00000004692	57,025385	1,559793633	0,3537015	4,4099148	1,03E-05	0,0001064	FDX1L
ENSGALG00000005958	558,63577	1,558583818	0,2329918	6,6894354	2,24E-11	6,70E-10	TRIM66
ENSGALG00000000826	97,953466	1,551558986	0,3236865	4,7933999	1,64E-06	2,03E-05	MAPK13
ENSGALG00000007690	2070,9973	1,54950396	0,1755538	8,8263752	1,08E-18	7,67E-17	TFAP2C
ENSGALG00000006964	225,19101	1,546629935	0,2930271	5,2781128	1,31E-07	2,05E-06	SDR42E2
ENSGALG00000005967	735,63667	1,545091384	0,2216792	6,9699438	3,17E-12	1,09E-10	LMO1
ENSGALG00000008440	429,09527	1,543692993	0,21647	7,1312088	9,95E-13	3,64E-11	CHST1
ENSGALG00000001882	38,709827	1,540579881	0,352114	4,3752309	1,21E-05	0,0001224	TNFRSF18
ENSGALG00000015373	27,601769	1,538791481	0,3445115	4,4665892	7,95E-06	8,37E-05	PDE6B
ENSGALG00000011577	399,56293	1,531134988	0,217286	7,0466337	1,83E-12	6,48E-11	CNTNAP5
ENSGALG00000025774	5724,9696	1,527733684	0,1270402	12,025592	2,61E-33	5,82E-31	HOXB2
ENSGALG00000005473	107,68664	1,527501434	0,3207303	4,7625734	1,91E-06	2,31E-05	
ENSGALG00000000479	244,69562	1,527264642	0,2552088	5,984374	2,17E-09	4,90E-08	PADI3
ENSGALG00000011071	944,33324	1,527069671	0,169472	9,0107512	2,05E-19	1,57E-17	CLMN
ENSGALG00000020459	25,515422	1,526419374	0,3416733	4,4674817	7,91E-06	8,34E-05	
ENSGALG00000014267	99,335217	1,525149591	0,3197285	4,7701403	1,84E-06	2,24E-05	RBM47
ENSGALG00000007803	165,80729	1,522432144	0,2956669	5,1491467	2,62E-07	3,86E-06	
ENSGALG00000001569	88,715373	1,518412601	0,3422278	4,4368471	9,13E-06	9,51E-05	RHOC
ENSGALG00000020745	195,27404	1,515524715	0,2743093	5,5248748	3,30E-08	5,85E-07	RHO
ENSGALG00000011227	778,97981	1,515331444	0,1818025	8,3350423	7,75E-17	4,65E-15	CDS1
ENSGALG00000007594	126,92297	1,512071087	0,3020168	5,0065791	5,54E-07	7,63E-06	ASB12
ENSGALG00000002314	85,131488	1,511687395	0,3333097	4,5353841	5,75E-06	6,23E-05	CDHR1
ENSGALG00000002353	221,89831	1,511078773	0,2623724	5,7592906	8,45E-09	1,69E-07	C1orf216
ENSGALG00000024397	46,37702	1,510326295	0,3539532	4,2670226	1,98E-05	0,000186	
ENSGALG00000023866	254,33653	1,510055693	0,2802587	5,3880776	7,12E-08	1,18E-06	ALPI
ENSGALG00000026089	41,811297	1,509966763	0,3504419	4,3087509	1,64E-05	0,0001581	SLC26A6
ENSGALG00000028386	982,7252	1,507238343	0,1699771	8,8673018	7,49E-19	5,49E-17	SPEG
ENSGALG00000006479	34,346853	1,50588664	0,3481979	4,3248009	1,53E-05	0,0001484	CCM2L
ENSGALG00000005297	304,6597	1,505251197	0,2455725	6,1295583	8,81E-10	2,10E-08	HHATL
ENSGALG00000004448	547,11485	1,504790559	0,2323026	6,4777179	9,31E-11	2,55E-09	BRINP2
ENSGALG00000026983	590,10355	1,50209184	0,2137064	7,0287654	2,08E-12	7,31E-11	CHRD
ENSGALG00000024264	40,681213	1,501303133	0,349559	4,2948485	1,75E-05	0,0001668	CTD-2207O23.12
ENSGALG00000028347	76,972548	1,501152953	0,3426288	4,381281	1,18E-05	0,0001192	SDCBP2
ENSGALG00000016556	93,532731	1,500831481	0,3253057	4,6136038	3,96E-06	4,47E-05	GVIN1
ENSGALG00000008424	31,555997	1,500581439	0,3495073	4,2934189	1,76E-05	0,0001675	CALML3
ENSGALG00000016287	253,69324	1,497802744	0,250661	5,9754121	2,30E-09	5,17E-08	NR0B1
ENSGALG00000028069	276,23402	1,497716991	0,2556436	5,858613	4,67E-09	9,87E-08	WNT16
ENSGALG00000015410	210,47767	1,497100835	0,2682786	5,5803964	2,40E-08	4,38E-07	BVES
ENSGALG00000014074	676,43326	1,49566297	0,191936	7,7925091	6,57E-15	3,08E-13	CPEB4
ENSGALG00000003713	62,007135	1,494159857	0,3495106	4,2750063	1,91E-05	0,0001803	CLCNKB
ENSGALG00000007409	87,08015	1,493981037	0,3289914	4,5410949	5,60E-06	6,09E-05	SCN4B
ENSGALG00000028823	172,11814	1,491796906	0,2989787	4,9896427	6,05E-07	8,21E-06	C9orf172
ENSGALG00000026585	53,571101	1,487448109	0,3507492	4,240774	2,23E-05	0,0002057	FAM19A3
ENSGALG00000027548	35,763933	1,48608799	0,3490327	4,2577329	2,07E-05	0,0001927	PROK1
ENSGALG00000028770	169,21444	1,483894729	0,3097948	4,7899285	1,67E-06	2,06E-05	INHBB
ENSGALG00000015237	27,293769	1,481694783	0,3444717	4,3013545	1,70E-05	0,0001626	PPIL6
ENSGALG00000015084	1284,7693	1,480304896	0,1853415	7,9869029	1,38E-15	7,11E-14	IGSF11
ENSGALG00000027357	83,363535	1,478068348	0,3342619	4,4218869	9,78E-06	0,000101	PTGES
ENSGALG00000007908	523,87573	1,477721596	0,2119438	6,9722343	3,12E-12	1,08E-10	EFEMP1

ENSGALG00000008888	216,09563	1,475198435	0,2663438	5,5386996	3,05E-08	5,45E-07	IL1RAPL2
ENSGALG00000000984	31,631294	1,47391651	0,3504148	4,2062051	2,60E-05	0,0002346	AGPAT1L
ENSGALG00000007862	431,76183	1,47265082	0,2334046	6,3094329	2,80E-10	7,11E-09	CNTN3
ENSGALG00000016538	1671,2767	1,472170836	0,1444523	10,191396	2,17E-24	2,59E-22	RAI2
ENSGALG00000002442	183,2009	1,464263461	0,2851722	5,1346638	2,83E-07	4,12E-06	ANKS4B
ENSGALG00000010107	1860,6289	1,462273804	0,1417872	10,313158	6,14E-25	7,75E-23	FHDC1
ENSGALG00000026677	510,78911	1,461550873	0,2721661	5,3700699	7,87E-08	1,29E-06	F10
ENSGALG00000000703	748,40789	1,459568013	0,2288692	6,3773027	1,80E-10	4,77E-09	SLC45A3
ENSGALG00000026069	33,975719	1,459502097	0,3469009	4,207259	2,58E-05	0,0002339	
ENSGALG00000001319	188,5387	1,458388464	0,2745911	5,3111274	1,09E-07	1,73E-06	CAMK1G
ENSGALG00000014297	511,06078	1,457150717	0,2085114	6,9883507	2,78E-12	9,64E-11	IRF7
ENSGALG00000004565	123,01022	1,456247274	0,3389164	4,2967747	1,73E-05	0,0001657	PHYHD1
ENSGALG00000010024	105,10355	1,453185654	0,3195715	4,5472947	5,43E-06	5,94E-05	
ENSGALG00000014941	729,89146	1,451697765	0,1864124	7,7875602	6,83E-15	3,20E-13	STXBP5L
ENSGALG00000018878	40,669529	1,44967545	0,3538085	4,0973448	4,18E-05	0,000353	
ENSGALG00000015128	44,346554	1,44940013	0,3539598	4,0948156	4,23E-05	0,0003559	AQP4
ENSGALG00000006751	459,34864	1,449311054	0,2141744	6,7669659	1,32E-11	4,09E-10	THY1
ENSGALG00000017041	876,05201	1,44806939	0,2013684	7,1911442	6,43E-13	2,41E-11	FREM2
ENSGALG00000007211	323,24032	1,44654086	0,2459257	5,8820245	4,05E-09	8,74E-08	CDH22
ENSGALG00000001727	728,89366	1,445847057	0,1802658	8,0206403	1,05E-15	5,52E-14	LZTS1
ENSGALG00000006239	29,398702	1,443346989	0,3491735	4,1336095	3,57E-05	0,0003093	
ENSGALG00000023395	151,59782	1,441830671	0,2999274	4,807265	1,53E-06	1,91E-05	PLIN1
ENSGALG00000013640	91,822966	1,440450208	0,3243745	4,4407017	8,97E-06	9,38E-05	MBP
ENSGALG00000014962	26,464506	1,440003001	0,3431954	4,1958697	2,72E-05	0,0002436	FAM26F
ENSGALG00000008267	52,964686	1,439464257	0,3533811	4,0734046	4,63E-05	0,0003844	IL5RA
ENSGALG00000011202	37,140825	1,438867662	0,3522352	4,0849627	4,41E-05	0,0003675	COLQ
ENSGALG00000012652	778,18023	1,438231617	0,2169215	6,6301938	3,35E-11	9,79E-10	KCNG2
ENSGALG00000001710	36,697213	1,437606263	0,3472289	4,1402264	3,47E-05	0,0003013	
ENSGALG00000014695	237,14245	1,436054042	0,2846492	5,0449961	4,54E-07	6,35E-06	CLCN1
ENSGALG00000024368	80,858185	1,435967902	0,3383077	4,2445619	2,19E-05	0,0002029	AMH
ENSGALG00000003678	3218,3918	1,429238494	0,1247553	11,456331	2,19E-30	3,97E-28	GLUL
ENSGALG00000012313	84,383689	1,428353126	0,34636	4,1238977	3,73E-05	0,0003194	H1F0
ENSGALG00000000914	24,818166	1,42659313	0,3378824	4,2221587	2,42E-05	0,0002211	
ENSGALG00000006565	175,49957	1,424795357	0,3261375	4,3686951	1,25E-05	0,0001254	GGT1
ENSGALG00000011888	40,65783	1,420709802	0,353895	4,0144953	5,96E-05	0,0004785	TGM4
ENSGALG00000010213	97,780516	1,420151903	0,3251042	4,3682976	1,25E-05	0,0001255	VSX2
ENSGALG00000008901	121,60703	1,417468594	0,3085387	4,594136	4,35E-06	4,87E-05	PTGFR
ENSGALG00000009596	108,17405	1,416089625	0,3214369	4,4054986	1,06E-05	0,0001081	TMEM117
ENSGALG00000014412	49,062969	1,415817045	0,3518535	4,0238822	5,72E-05	0,0004628	CSTA
ENSGALG00000000589	81,385408	1,415474788	0,3386211	4,1801144	2,91E-05	0,0002588	BTN1A1
ENSGALG00000023834	58,026469	1,413727582	0,350805	4,0299523	5,58E-05	0,0004527	
ENSGALG00000018998	220,94684	1,412120959	0,2810636	5,0242041	5,06E-07	7,03E-06	UNC5A
ENSGALG00000013081	81,619637	1,411618895	0,3443628	4,0992204	4,15E-05	0,0003508	VWC2
ENSGALG00000027520	176,34242	1,409426755	0,294554	4,7849521	1,71E-06	2,10E-05	
ENSGALG00000020423	91,673069	1,409349068	0,344453	4,0915566	4,28E-05	0,0003598	NKX2-1
ENSGALG00000026957	482,71907	1,407844107	0,20929	6,726761	1,73E-11	5,28E-10	SEMA4G
ENSGALG00000010486	221,46033	1,406315246	0,2602657	5,4033833	6,54E-08	1,10E-06	JAKMIP3
ENSGALG00000028936	318,09117	1,405167114	0,2341016	6,0023824	1,94E-09	4,43E-08	
ENSGALG00000002637	48,952453	1,404353552	0,3533799	3,9740621	7,07E-05	0,0005557	RBP7
ENSGALG00000026948	265,89811	1,40406892	0,2839426	4,9449037	7,62E-07	1,01E-05	ADD2
ENSGALG00000016782	106,88039	1,400781577	0,3284889	4,2643192	2,01E-05	0,000188	IL1R2
ENSGALG00000015918	313,69588	1,399804901	0,2380455	5,8804082	4,09E-09	8,82E-08	NECAB1
ENSGALG00000011594	42,210813	1,39976735	0,3539056	3,9552003	7,65E-05	0,0005929	NR1H4
ENSGALG00000009390	538,73431	1,398258988	0,2055508	6,8024979	1,03E-11	3,26E-10	MET
ENSGALG00000004252	60,119703	1,3977567	0,3472961	4,0246834	5,71E-05	0,0004615	
ENSGALG00000019864	58,892366	1,392548079	0,3487729	3,9927067	6,53E-05	0,0005177	
ENSGALG00000029083	179,60714	1,390642479	0,2814687	4,9406649	7,79E-07	1,03E-05	NXPH2
ENSGALG00000012875	68,467203	1,389711553	0,3440597	4,0391582	5,36E-05	0,0004376	WEE2
ENSGALG00000023689	74,977508	1,388126911	0,3405714	4,0758757	4,58E-05	0,0003808	ASS1
ENSGALG00000026536	52,51215	1,387507758	0,3536902	3,9229466	8,75E-05	0,0006706	IP6K3
ENSGALG00000014857	696,58457	1,387229623	0,1828391	7,587161	3,27E-14	1,45E-12	SEPP1
ENSGALG00000002319	151,6038	1,386034084	0,2938554	4,7167213	2,40E-06	2,84E-05	TEKT2

ENSGALG00000015776	28,48922	1,382386109	0,3437254	4,0217745	5,78E-05	0,0004664	GABRR2
ENSGALG00000007636	33,478978	1,381489214	0,3508105	3,9379927	8,22E-05	0,0006327	PCK1
ENSGALG00000007014	150,69691	1,378668928	0,2898641	4,7562591	1,97E-06	2,38E-05	PYROXD2
ENSGALG00000027973	59,731595	1,378158223	0,3525539	3,909071	9,27E-05	0,0007038	CATH1
ENSGALG00000017099	99,501102	1,377440865	0,3228202	4,266898	1,98E-05	0,000186	RASL11A
ENSGALG00000005493	91,862545	1,375327372	0,3276333	4,1977643	2,70E-05	0,0002424	ITGB1BP2
ENSGALG00000006522	45,353339	1,373172	0,3533367	3,8862984	0,0001018	0,0007623	HCK
ENSGALG00000012956	1426,5049	1,372804533	0,1863192	7,3680244	1,73E-13	6,93E-12	TMTC1
ENSGALG00000002105	44,541184	1,371897103	0,3536727	3,8790025	0,0001049	0,0007838	FOXI1
ENSGALG00000016584	157,58752	1,371284929	0,2995639	4,5776048	4,70E-06	5,25E-05	EGFL6
ENSGALG00000000730	181,14132	1,369179792	0,2801686	4,8869848	1,02E-06	1,33E-05	CACNA1S
ENSGALG00000009151	104,26311	1,368816021	0,3214356	4,2584453	2,06E-05	0,0001922	FCN2
ENSGALG00000027420	141,8792	1,367365407	0,2942071	4,6476294	3,36E-06	3,86E-05	POPDC3
ENSGALG00000012971	180,16128	1,365874431	0,2750242	4,9663794	6,82E-07	9,15E-06	CCDC170
ENSGALG00000005852	42,716737	1,364112477	0,3537079	3,8566077	0,000115	0,0008438	SRMS
ENSGALG00000020458	27,129951	1,361629377	0,3397843	4,0073347	6,14E-05	0,0004906	PLA2G4E
ENSGALG00000013221	46,584811	1,36089575	0,3526259	3,8593185	0,0001137	0,0008363	ARHGEF33
ENSGALG00000016155	67,519598	1,359832396	0,3425225	3,9700528	7,19E-05	0,0005636	COL19A1
ENSGALG00000004840	833,06506	1,358453061	0,1689566	8,0402477	8,97E-16	4,78E-14	RPH3A
ENSGALG00000014913	89,586553	1,358196979	0,3332154	4,0760324	4,58E-05	0,0003808	ROS1
ENSGALG00000028822	179,37935	1,357789969	0,2792664	4,861989	1,16E-06	1,50E-05	RNF152
ENSGALG00000001932	150,41219	1,357009459	0,310456	4,3710208	1,24E-05	0,0001245	SRRM3
ENSGALG00000006104	74,225594	1,356707559	0,3368845	4,027219	5,64E-05	0,0004571	ADAMTSL3
ENSGALG00000013914	741,46351	1,355434386	0,2231994	6,0727508	1,26E-09	2,95E-08	SERINC4
ENSGALG00000001863	95,573016	1,354146203	0,3495798	3,8736396	0,0001072	0,0007966	VTG2
ENSGALG00000024064	33,370003	1,353791502	0,3529207	3,835965	0,0001251	0,0009082	BMP8B
ENSGALG00000017036	3151,4224	1,353726483	0,1434983	9,4337437	3,96E-21	3,41E-19	LHFP
ENSGALG00000016342	64,688956	1,353678127	0,3480736	3,8890574	0,0001006	0,0007541	MYOM2
ENSGALG00000028162	25,784822	1,353451729	0,3407708	3,9717367	7,14E-05	0,0005606	
ENSGALG00000025857	83,462878	1,352908608	0,3353198	4,0346816	5,47E-05	0,0004451	
ENSGALG00000005898	30,859755	1,351366295	0,3483263	3,8795984	0,0001046	0,0007823	DND1
ENSGALG00000026817	190,25856	1,350039834	0,3072699	4,3936618	1,11E-05	0,0001138	
ENSGALG00000011259	318,18554	1,346953088	0,237873	5,6624895	1,49E-08	2,86E-07	RPE65
ENSGALG00000015186	1129,1529	1,345677898	0,1669938	8,0582487	7,74E-16	4,20E-14	KLHL14
ENSGALG00000027568	132,01086	1,344832291	0,3108069	4,3269066	1,51E-05	0,0001475	
ENSGALG00000003671	47,547146	1,341551712	0,3538875	3,7908993	0,0001501	0,0010672	
ENSGALG00000007106	977,16123	1,340640592	0,2071983	6,4703268	9,78E-11	2,66E-09	DNAH17
ENSGALG00000002466	67,363583	1,337320513	0,3459423	3,8657324	0,0001108	0,0008178	SLC2A5
ENSGALG00000008283	62,956159	1,336619904	0,3480958	3,8398045	0,0001231	0,0008951	CYP2J2L6
ENSGALG00000009058	854,57904	1,336327117	0,176152	7,5862168	3,29E-14	1,46E-12	ENTPD2
ENSGALG00000014523	712,2168	1,335395904	0,1808886	7,3824225	1,55E-13	6,30E-12	MATN2
ENSGALG00000027624	90,7092	1,332865286	0,324427	4,1083672	3,98E-05	0,0003397	
ENSGALG00000020380	160,77545	1,332253662	0,2975792	4,476972	7,57E-06	8,01E-05	
ENSGALG00000014117	36,1409	1,330198207	0,3537602	3,7601691	0,0001698	0,0011923	AVP
ENSGALG00000005330	2519,7711	1,328511787	0,1342416	9,8964213	4,31E-23	4,45E-21	SNCAIP
ENSGALG00000013481	89,334053	1,327640637	0,3315342	4,0045364	6,21E-05	0,0004953	IKZF3
ENSGALG00000008434	453,34748	1,327566577	0,2234773	5,9404989	2,84E-09	6,30E-08	SORCS3
ENSGALG00000006134	56,091168	1,326807835	0,348696	3,8050556	0,0001418	0,0010148	HOGA1
ENSGALG00000004903	45,997304	1,326402261	0,3537178	3,7498888	0,0001769	0,0012333	CHST8
ENSGALG00000015309	102,61836	1,321942011	0,3301857	4,0036322	6,24E-05	0,0004969	SCML4
ENSGALG00000016551	5221,0551	1,321564075	0,1177455	11,223902	3,11E-29	5,11E-27	AC025048.1
ENSGALG00000013302	789,25879	1,321288665	0,1757375	7,5185339	5,54E-14	2,35E-12	SYT7
ENSGALG00000009315	90,474554	1,318764229	0,3335429	3,9538073	7,69E-05	0,000596	PAPLN
ENSGALG00000027575	153,60071	1,316495304	0,2966273	4,4382135	9,07E-06	9,46E-05	RAMP3
ENSGALG00000003050	83,782773	1,314582894	0,3351464	3,9224135	8,77E-05	0,0006717	ADRA2B
ENSGALG00000002792	26,932942	1,31306542	0,34508	3,8051042	0,0001417	0,0010148	SPDEF
ENSGALG00000016368	37,747118	1,308737431	0,3539388	3,6976373	0,0002176	0,0014711	KLHL38
ENSGALG00000002389	532,14602	1,308458099	0,1976049	6,6215867	3,55E-11	1,03E-09	ITGB4
ENSGALG00000000164	213,8267	1,307977843	0,2786458	4,6940518	2,68E-06	3,15E-05	MYBPH
ENSGALG00000007732	613,17499	1,306274335	0,1852621	7,0509534	1,78E-12	6,32E-11	RTN4R
ENSGALG00000013838	384,54265	1,305966304	0,2158246	6,0510536	1,44E-09	3,34E-08	
ENSGALG00000003316	80,926571	1,303670454	0,3335441	3,9085405	9,29E-05	0,0007043	FMO3

ENSGALG00000005802	409,59537	1,302158184	0,2262217	5,7561153	8,61E-09	1,72E-07	FLT4
ENSGALG00000016109	151,6721	1,299400996	0,288909	4,4976137	6,87E-06	7,31E-05	KCNV1
ENSGALG00000009700	477,10962	1,299326338	0,2326682	5,5844594	2,34E-08	4,28E-07	PDK4
ENSGALG00000006045	136,04518	1,297721233	0,3015022	4,3041847	1,68E-05	0,0001609	SH3GL3
ENSGALG00000006165	67,169846	1,296804529	0,3443385	3,7660745	0,0001658	0,0011656	WDFY4
ENSGALG00000002234	407,0013	1,296274176	0,217295	5,9655032	2,44E-09	5,48E-08	UNC13D
ENSGALG00000002051	21,039089	1,294841211	0,3323973	3,895462	9,80E-05	0,0007377	DNASE1
ENSGALG00000008022	294,02931	1,293915137	0,239607	5,4001556	6,66E-08	1,11E-06	TAPBP
ENSGALG00000000775	136,7775	1,291374316	0,3199881	4,0356942	5,44E-05	0,0004436	CTSS
ENSGALG00000010540	264,70457	1,289706596	0,2432527	5,3019215	1,15E-07	1,81E-06	TTC39A
ENSGALG00000005790	146,07742	1,289660896	0,3149604	4,0946765	4,23E-05	0,0003559	DEPTOR
ENSGALG00000004859	354,90162	1,288425333	0,2477818	5,1998389	1,99E-07	3,02E-06	ZNFX1
ENSGALG00000003118	754,37839	1,286216079	0,1877588	6,8503656	7,37E-12	2,38E-10	GPR124
ENSGALG00000006779	194,18454	1,285794374	0,2904687	4,4266201	9,57E-06	9,92E-05	PDZD3
ENSGALG00000012362	940,67168	1,285048698	0,1725255	7,4484556	9,44E-14	3,88E-12	THSD7B
ENSGALG00000004044	637,18108	1,285008651	0,1978538	6,4947372	8,32E-11	2,30E-09	CSRP3
ENSGALG00000003580	1991,2953	1,284312011	0,1942227	6,6125738	3,78E-11	1,09E-09	MMP2
ENSGALG00000002855	1183,6448	1,278753967	0,1621147	7,8879566	3,07E-15	1,50E-13	SARDH
ENSGALG00000016564	80,407544	1,275019755	0,3413328	3,7354158	0,0001874	0,0012926	PTK2B
ENSGALG00000001028	434,61935	1,272121671	0,2282446	5,5735011	2,50E-08	4,54E-07	RNF43
ENSGALG00000007078	141,27907	1,270778013	0,3066415	4,1441819	3,41E-05	0,0002963	ENTPD1
ENSGALG00000009689	798,33378	1,269454305	0,1789844	7,0925427	1,32E-12	4,74E-11	PON2
ENSGALG00000012740	435,21639	1,269186578	0,2159823	5,8763447	4,19E-09	8,99E-08	ADAM12
ENSGALG00000000909	237,54325	1,268775969	0,2625461	4,832584	1,35E-06	1,70E-05	GFAP
ENSGALG00000016967	712,9034	1,267335006	0,2153024	5,8863033	3,95E-09	8,55E-08	ENOX1
ENSGALG00000019797	403,10583	1,266041829	0,2858329	4,429307	9,45E-06	9,81E-05	
ENSGALG00000007562	30,687084	1,263491411	0,3496081	3,6140222	0,0003015	0,00195	FGF4
ENSGALG00000008953	507,74233	1,263384491	0,2035775	6,2059148	5,44E-10	1,33E-08	AASS
ENSGALG00000001795	978,86532	1,261084777	0,1873221	6,7321726	1,67E-11	5,13E-10	GFRA2
ENSGALG00000012978	566,09757	1,261073108	0,1899643	6,6384742	3,17E-11	9,30E-10	CDYL2
ENSGALG00000006725	183,57715	1,259943343	0,3248929	3,8780263	0,0001053	0,0007865	ACAN
ENSGALG00000014398	578,99454	1,259804122	0,2168754	5,8088844	6,29E-09	1,29E-07	PPARGC1A
ENSGALG00000015598	190,82086	1,259472248	0,277866	4,5326611	5,82E-06	6,30E-05	RASGRF2
ENSGALG00000002647	518,74694	1,258418808	0,2451254	5,1337755	2,84E-07	4,13E-06	PLS1
ENSGALG00000003490	147,80461	1,25835771	0,2972254	4,2336817	2,30E-05	0,0002112	ALDH3B1
ENSGALG00000005458	78,515702	1,258264066	0,3397845	3,7031237	0,000213	0,0014432	RPL3L
ENSGALG00000005843	363,33688	1,25767053	0,2724846	4,6155656	3,92E-06	4,43E-05	EEF1A2
ENSGALG00000006809	89,492709	1,25736391	0,3250463	3,8682605	0,0001096	0,0008106	POU2AF1
ENSGALG00000014014	690,53146	1,255177749	0,1779605	7,0531265	1,75E-12	6,24E-11	LAMA2
ENSGALG00000001348	34,354329	1,253857774	0,350669	3,5756163	0,0003494	0,0022157	TEKT3
ENSGALG00000021855	271,81428	1,251787198	0,252542	4,9567492	7,17E-07	9,57E-06	CCDC39
ENSGALG00000007193	236,11491	1,251769992	0,2743537	4,5626132	5,05E-06	5,59E-05	HRASLS
ENSGALG00000018557	36,565332	1,251460959	0,3537306	3,537893	0,0004033	0,0025029	SOD3
ENSGALG00000025896	117,28876	1,251281202	0,3211612	3,8961159	9,77E-05	0,0007361	PLPPR4
ENSGALG00000012547	219,75607	1,25117298	0,2908698	4,3014887	1,70E-05	0,0001626	ACVR1C
ENSGALG00000013356	85,223066	1,250931573	0,339616	3,6833703	0,0002302	0,001543	IKBKE
ENSGALG00000003404	2332,0677	1,24748878	0,1390419	8,972036	2,91E-19	2,21E-17	TFEB
ENSGALG00000001206	49,037007	1,247009104	0,3530765	3,5318378	0,0004127	0,0025563	
ENSGALG00000028496	22,12018	1,24376929	0,3359139	3,7026432	0,0002134	0,0014453	
ENSGALG00000007065	27,838516	1,239888336	0,3478406	3,5645304	0,0003645	0,0022955	GJD4
ENSGALG00000015345	537,38522	1,237723953	0,2000428	6,187296	6,12E-10	1,49E-08	
ENSGALG00000028448	68,098086	1,236909029	0,3465147	3,5695715	0,0003576	0,002257	SH2D1B
ENSGALG00000001564	906,15479	1,234344815	0,196986	6,2661565	3,70E-10	9,23E-09	ATP2A3
ENSGALG00000023036	562,73194	1,234170444	0,2058267	5,9961639	2,02E-09	4,57E-08	FRMPD1
ENSGALG00000010927	22,212585	1,233723332	0,3353328	3,6791008	0,0002341	0,0015644	MEPE
ENSGALG00000028681	168,61801	1,232657979	0,2905515	4,2424766	2,21E-05	0,0002043	
ENSGALG00000015573	43,775019	1,232214926	0,3535094	3,4856638	0,0004909	0,0029548	FHL5
ENSGALG00000005298	800,81856	1,231219861	0,1718969	7,1625504	7,92E-13	2,92E-11	ST6GALNAC6
ENSGALG00000016112	539,87209	1,228482119	0,2093942	5,8668384	4,44E-09	9,46E-08	NOV
ENSGALG00000000107	237,72923	1,227291307	0,277099	4,4290711	9,46E-06	9,81E-05	TRIM7.1
ENSGALG00000002086	83,650156	1,225708617	0,3336205	3,6739612	0,0002388	0,0015908	RASL10B
ENSGALG00000002667	699,19963	1,225466735	0,1768396	6,9298206	4,21E-12	1,40E-10	OLFML2B

ENSGALG00000013431	35,449073	1,223926576	0,3535105	3,4622067	0,0005358	0,0031756	RAX2
ENSGALG00000028031	210,36093	1,22342747	0,2837755	4,3112512	1,62E-05	0,0001564	C1QL1
ENSGALG00000012019	150,4401	1,222353012	0,2981526	4,0997566	4,14E-05	0,0003505	ARSJ
ENSGALG00000000048	677,12707	1,221920851	0,1990761	6,1379597	8,36E-10	2,00E-08	
ENSGALG00000028946	391,05664	1,217039733	0,2510887	4,8470509	1,25E-06	1,59E-05	CSRP2
ENSGALG00000014407	118,55781	1,216198172	0,3199077	3,801716	0,0001437	0,0010259	CASR
ENSGALG00000012658	2563,9636	1,214376265	0,1523893	7,9689058	1,60E-15	8,19E-14	TCP11L2
ENSGALG00000028402	33,811586	1,211737057	0,3495685	3,466379	0,0005275	0,0031377	
ENSGALG00000003345	1065,5168	1,211546204	0,160937	7,5280764	5,15E-14	2,19E-12	PLCD4
ENSGALG00000005083	47,868272	1,210240985	0,3512641	3,4453881	0,0005702	0,0033455	SLC5A10
ENSGALG00000003803	110,85757	1,208011558	0,3397797	3,555279	0,0003776	0,0023603	DAB2
ENSGALG00000026578	55,333978	1,20748664	0,3538872	3,4120664	0,0006447	0,0037053	
ENSGALG00000014126	18,992333	1,207239433	0,3279747	3,6808919	0,0002324	0,0015558	ENDOUL
ENSGALG00000015764	5967,7523	1,20416113	0,1908692	6,3088274	2,81E-10	7,13E-09	FABP5
ENSGALG00000015528	170,23305	1,204136401	0,3006436	4,0051957	6,20E-05	0,0004943	JAKMIP1
ENSGALG00000002355	234,00639	1,203134529	0,2548917	4,7201799	2,36E-06	2,80E-05	RBPMS2
ENSGALG00000004641	146,28084	1,20257315	0,2965454	4,0552753	5,01E-05	0,0004122	MYO5C
ENSGALG00000026903	111,36196	1,200671289	0,3283141	3,6570806	0,0002551	0,0016869	CALB2
ENSGALG00000004745	1802,3575	1,199713581	0,141877	8,4560121	2,77E-17	1,74E-15	NATD1
ENSGALG00000013890	33,560749	1,1996045	0,3520141	3,4078307	0,0006548	0,0037491	MC5R
ENSGALG00000019280	990,72458	1,199070585	0,1733998	6,915063	4,68E-12	1,54E-10	LMO3
ENSGALG00000004066	55,61946	1,199060923	0,3539016	3,3881191	0,0007037	0,0039939	HTR6
ENSGALG00000002082	57,1028	1,198569946	0,3484813	3,4394093	0,000583	0,0034066	AMPD1
ENSGALG00000013090	44,714809	1,197838739	0,3538143	3,3855007	0,0007105	0,0040265	LOXL4
ENSGALG00000014696	222,59602	1,197793931	0,2597544	4,6112558	4,00E-06	4,51E-05	ZBTB7C
ENSGALG00000006922	19,384458	1,194740346	0,3271281	3,6522095	0,00026	0,0017142	KCNJ14
ENSGALG00000011489	4656,6813	1,193784841	0,1216657	9,8120096	1,00E-22	9,93E-21	
ENSGALG00000016590	18,890623	1,192009768	0,3270811	3,6443865	0,000268	0,001757	TLR7
ENSGALG00000002407	144,98036	1,191296249	0,3041418	3,9169112	8,97E-05	0,0006837	HYDIN
ENSGALG00000012890	273,96599	1,191168201	0,2640739	4,5107378	6,46E-06	6,90E-05	DGKI
ENSGALG00000027218	239,44089	1,190798556	0,2757781	4,317959	1,57E-05	0,0001524	
ENSGALG00000017040	33,080589	1,190245244	0,3523261	3,3782489	0,0007295	0,0041177	
ENSGALG00000008362	175,02358	1,190011885	0,2754196	4,3207232	1,56E-05	0,000151	SSTR4
ENSGALG00000004521	401,35326	1,187877806	0,2197321	5,4060281	6,44E-08	1,08E-06	GPX3
ENSGALG00000006074	272,24078	1,186152058	0,28359	4,1826293	2,88E-05	0,0002563	SOX6
ENSGALG00000003842	39,781188	1,185854219	0,3538378	3,3514066	0,000804	0,0044662	GHRH
ENSGALG00000021882	58,697437	1,185518376	0,3468224	3,4182285	0,0006303	0,0036347	
ENSGALG00000002744	252,46116	1,184999187	0,2518537	4,705109	2,54E-06	3,00E-05	PCDH15
ENSGALG00000006768	37,969199	1,18499895	0,3536582	3,3506896	0,0008061	0,0044728	
ENSGALG00000014812	711,71818	1,183271769	0,1870163	6,3271055	2,50E-10	6,39E-09	SIDT1
ENSGALG00000017331	630,73395	1,182854332	0,188652	6,2700317	3,61E-10	9,05E-09	GDPD5
ENSGALG00000005547	144,89722	1,180323703	0,2989787	3,9478524	7,89E-05	0,0006093	PIK3AP1
ENSGALG00000017291	662,46181	1,180080414	0,209044	5,6451284	1,65E-08	3,14E-07	FAR2
ENSGALG00000006308	28,078472	1,179461013	0,3370947	3,4989008	0,0004672	0,0028338	
ENSGALG00000013112	320,80404	1,179282262	0,2330597	5,0600015	4,19E-07	5,90E-06	RERGL
ENSGALG00000007333	325,51856	1,178440858	0,2584425	4,5597804	5,12E-06	5,65E-05	UBAP1L
ENSGALG00000001021	191,27134	1,17756894	0,2838137	4,1490906	3,34E-05	0,0002911	MYOCD
ENSGALG00000003821	322,04402	1,177115475	0,2347575	5,0141754	5,33E-07	7,38E-06	RAMP1
ENSGALG00000003164	243,86317	1,17697714	0,2590714	4,5430608	5,54E-06	6,04E-05	DNM3
ENSGALG00000016651	946,90096	1,174964335	0,1848632	6,35586	2,07E-10	5,41E-09	TDH
ENSGALG00000002435	70,191489	1,174947125	0,34691	3,3868933	0,0007069	0,0040085	DUOX2
ENSGALG00000001331	1782,6492	1,174179888	0,1437468	8,16839	3,13E-16	1,75E-14	ST14
ENSGALG00000009114	216,59264	1,170782693	0,2608026	4,4891525	7,15E-06	7,60E-05	DGAT2
ENSGALG00000028580	59,186277	1,170297276	0,3494653	3,3488229	0,0008116	0,0044934	C15orf48
ENSGALG00000006099	3185,4261	1,169037408	0,1372144	8,5197869	1,60E-17	1,03E-15	ZFPM1
ENSGALG00000014261	23192,424	1,166708788	0,1480767	7,8790822	3,30E-15	1,59E-13	UCHL1
ENSGALG00000006627	44,723529	1,164076921	0,352923	3,2983882	0,0009724	0,0052103	UPB1
ENSGALG00000012668	622,84546	1,1628752	0,2448035	4,7502395	2,03E-06	2,44E-05	DCDC2
ENSGALG00000011446	368,84385	1,161895934	0,2215158	5,2452066	1,56E-07	2,42E-06	TNFAIP2
ENSGALG00000005630	38,097087	1,161740711	0,3428362	3,3886172	0,0007025	0,0039883	CRYBB1
ENSGALG00000023879	439,21059	1,160823609	0,2116765	5,4839511	4,16E-08	7,24E-07	
ENSGALG00000007596	99,216367	1,160341621	0,3237884	3,5836422	0,0003388	0,0021608	

ENSGALG00000005721	236,12764	1,159323873	0,251452	4,6105181	4,02E-06	4,52E-05	DGKG
ENSGALG00000013911	546,72587	1,159209839	0,2024827	5,7249821	1,03E-08	2,03E-07	ZC3HAV1
ENSGALG00000011418	18,242274	1,158885244	0,3251653	3,5639875	0,0003653	0,002297	CCR6
ENSGALG00000028489	20,536962	1,158414063	0,3317704	3,4916132	0,0004801	0,0029001	
ENSGALG00000001031	327,10314	1,156740289	0,2440319	4,7401197	2,14E-06	2,56E-05	HSF5
ENSGALG00000002016	953,70998	1,156233761	0,1649483	7,0096752	2,39E-12	8,30E-11	PRKCD
ENSGALG00000001095	18,255475	1,156162616	0,3249866	3,5575704	0,0003743	0,0023463	CCDC183
ENSGALG00000013980	288,26492	1,155390063	0,2983628	3,8724333	0,0001078	0,0007995	SLC2A12
ENSGALG00000026241	639,21895	1,154849081	0,2302822	5,0149306	5,31E-07	7,36E-06	GRIN3B
ENSGALG00000006208	100,69013	1,154488936	0,3219519	3,5859053	0,0003359	0,0021441	OTOG
ENSGALG00000009641	18352,412	1,153845642	0,1458991	7,9085187	2,60E-15	1,28E-13	COL1A2
ENSGALG00000012288	52,934002	1,152071153	0,3501204	3,2905002	0,0010001	0,0053293	SLC16A8
ENSGALG00000015932	523,70552	1,152042382	0,2240419	5,1420853	2,72E-07	3,98E-06	KCNQ5
ENSGALG00000003789	56,043715	1,151405842	0,3500328	3,2894227	0,0010039	0,0053476	FAM78A
ENSGALG00000010365	313,07869	1,151056068	0,2620814	4,39198	1,12E-05	0,0001143	ESRRB
ENSGALG00000017691	74,633315	1,149429932	0,3427848	3,3532112	0,0007988	0,0044427	
ENSGALG00000017374	478,61541	1,149174538	0,2222568	5,1704808	2,33E-07	3,48E-06	SLITRK3
ENSGALG00000028710	21,101173	1,148265888	0,3321095	3,4574919	0,0005452	0,0032233	
ENSGALG00000012374	515,33143	1,147327656	0,1949041	5,8866278	3,94E-09	8,55E-08	FRMD6
ENSGALG00000016328	5259,3238	1,146952103	0,1404012	8,1691038	3,11E-16	1,75E-14	GSTA
ENSGALG00000025721	19,243146	1,146921853	0,324147	3,5382766	0,0004027	0,0025004	
ENSGALG00000012365	9658,5394	1,146353139	0,1101377	10,408358	2,27E-25	2,97E-23	PYGL
ENSGALG00000005540	392,97544	1,145405784	0,219512	5,2179637	1,81E-07	2,76E-06	MICAL2
ENSGALG00000028702	48,721731	1,145044679	0,3522221	3,2509165	0,0011503	0,0060053	EVX1
ENSGALG00000022819	93,418767	1,14482978	0,3365037	3,4021308	0,0006686	0,0038249	PCP4
ENSGALG00000001968	55,570176	1,14394352	0,3522778	3,2472771	0,0011651	0,006064	SYT6
ENSGALG00000012505	1173,094	1,142096363	0,156235	7,3101186	2,67E-13	1,05E-11	LRFN5
ENSGALG00000015758	1198,984	1,141437782	0,170779	6,6837117	2,33E-11	6,96E-10	PAG1
ENSGALG00000003876	41,544916	1,140755556	0,3447861	3,3085895	0,0009377	0,0050721	TIMD4
ENSGALG00000009791	992,57388	1,140657464	0,2126878	5,3630614	8,18E-08	1,34E-06	PROX1
ENSGALG00000028130	1683,1758	1,140619832	0,1589917	7,1740827	7,28E-13	2,72E-11	KIAA1644
ENSGALG00000010673	22,777798	1,139845847	0,3403007	3,3495255	0,0008095	0,0044839	SLC1A7
ENSGALG00000006436	58,776846	1,138634151	0,3530266	3,2253491	0,0012582	0,0064643	AQP12
ENSGALG00000000620	143,16144	1,137872957	0,293674	3,8746119	0,0001068	0,0007954	FABP3
ENSGALG00000013818	524,97865	1,136933045	0,2108978	5,3909206	7,01E-08	1,17E-06	CITED2
ENSGALG00000007236	26,585317	1,136243459	0,3440899	3,30217	0,0009594	0,005167	PLA2G1BL
ENSGALG00000023643	36,522348	1,136155947	0,3539078	3,2103166	0,0013259	0,0067509	
ENSGALG00000007129	549,58573	1,134513156	0,2759889	4,1107204	3,94E-05	0,0003371	ALDH1A3
ENSGALG00000001442	382,78952	1,131898366	0,2156493	5,248791	1,53E-07	2,37E-06	OPCML
ENSGALG00000000217	558,86585	1,131400306	0,2196843	5,1501182	2,60E-07	3,84E-06	PPFIA4
ENSGALG00000000911	18,802113	1,131377385	0,3232235	3,5002948	0,0004647	0,002821	IL19
ENSGALG00000003028	252,86338	1,131167716	0,2536539	4,4594925	8,22E-06	8,64E-05	ADAMTS13
ENSGALG00000027626	26,263554	1,129092221	0,3472331	3,251684	0,0011472	0,0059985	
ENSGALG00000008063	112,83661	1,129089778	0,3199799	3,5286266	0,0004177	0,0025816	NOX5
ENSGALG00000016592	75,509326	1,127895523	0,3458232	3,2614806	0,0011083	0,0058217	PRPS2
ENSGALG00000021529	183,02929	1,127864143	0,273066	4,1303714	3,62E-05	0,0003123	GP1BB
ENSGALG00000006503	1322,5302	1,127315674	0,1739831	6,4794533	9,21E-11	2,52E-09	VDAC1
ENSGALG00000026333	170,5148	1,126889713	0,280963	4,0108121	6,05E-05	0,0004843	GNGT2
ENSGALG00000015348	3709,6553	1,126202791	0,1601094	7,03396	2,01E-12	7,08E-11	ALCAM
ENSGALG00000027872	562,41556	1,125834202	0,2065498	5,4506672	5,02E-08	8,57E-07	
ENSGALG00000007964	712,00528	1,125506336	0,1939073	5,8043524	6,46E-09	1,32E-07	GATSL3
ENSGALG00000008940	122,07312	1,125469701	0,3044547	3,6966742	0,0002184	0,0014752	SPTBN5
ENSGALG00000012252	31,066473	1,124245419	0,3463855	3,2456478	0,0011718	0,0060895	
ENSGALG00000009237	283,64685	1,124183808	0,2526532	4,4495132	8,61E-06	9,03E-05	TLR2A
ENSGALG00000008629	362,4306	1,122527446	0,2874644	3,9049269	9,43E-05	0,0007125	
ENSGALG00000004649	115,31891	1,122312882	0,3390875	3,3098029	0,0009336	0,0050602	TRPV1
ENSGALG00000016821	91,586414	1,121951764	0,3275569	3,4252114	0,0006143	0,0035683	TMEM255B
ENSGALG00000000994	36,33755	1,120119947	0,3533528	3,1699761	0,0015245	0,0075368	RAB42
ENSGALG00000009943	1812,2354	1,119992507	0,1420982	7,8818219	3,23E-15	1,56E-13	FAM117A
ENSGALG00000028795	3146,8331	1,118039364	0,160426	6,9691886	3,19E-12	1,09E-10	CADM3
ENSGALG00000026758	74,554613	1,11741437	0,3353836	3,33175	0,000863	0,0047277	
ENSGALG00000001204	1443,1255	1,117076992	0,1466887	7,6152892	2,63E-14	1,19E-12	ENKD1

ENSGALG00000012055	30,631938	1,115864912	0,3495485	3,1923038	0,0014114	0,0070989	TGFBR2L
ENSGALG00000015469	3626,2078	1,115704157	0,1321025	8,4457457	3,02E-17	1,89E-15	IGSF3
ENSGALG00000009963	76,780039	1,114244364	0,3375856	3,3006277	0,0009647	0,0051852	LYZ
ENSGALG00000011414	712,8987	1,112714675	0,1796621	6,1933742	5,89E-10	1,44E-08	AMDHD1
ENSGALG00000015454	87,061362	1,111985108	0,3376828	3,2929875	0,0009913	0,0052948	CA8
ENSGALG00000010233	149,38519	1,111330421	0,289789	3,8349641	0,0001256	0,0009114	SYNDIG1L
ENSGALG00000024411	95,571509	1,110994905	0,322591	3,4439741	0,0005732	0,0033582	C2orf82
ENSGALG00000003016	111,9759	1,109545556	0,3100714	3,5783554	0,0003458	0,0021977	MASP2
ENSGALG00000006792	447,85614	1,109047707	0,21888	5,0669216	4,04E-07	5,71E-06	VSTM2L
ENSGALG00000009823	207,86307	1,108978484	0,2670183	4,1531933	3,28E-05	0,0002863	PTPRE
ENSGALG00000027501	1178,2304	1,108782799	0,1555553	7,1279016	1,02E-12	3,72E-11	ANPEP
ENSGALG00000004017	335,24395	1,108435951	0,2275944	4,8702245	1,11E-06	1,44E-05	TOR1AIP2
ENSGALG00000025912	62,633971	1,108425226	0,3437693	3,2243285	0,0012627	0,00648	
ENSGALG00000000158	209,10615	1,107475494	0,2643096	4,1900691	2,79E-05	0,0002495	DMA
ENSGALG00000002610	604,59063	1,105862871	0,189678	5,8302128	5,54E-09	1,15E-07	
ENSGALG00000023621	702,55001	1,105773477	0,197195	5,6075141	2,05E-08	3,82E-07	SFXN3
ENSGALG00000009574	24,975698	1,105678443	0,3438055	3,216	0,0012999	0,0066483	IL12A
ENSGALG00000006000	170,90987	1,105166021	0,292418	3,7794044	0,0001572	0,0011107	ADAMTS2
ENSGALG00000001011	623,47251	1,104831537	0,2206424	5,00734	5,52E-07	7,61E-06	CNGB1
ENSGALG00000011473	347,60043	1,104703027	0,2275411	4,8549595	1,20E-06	1,54E-05	RPS6KA2
ENSGALG00000026834	233,70852	1,10373431	0,2598301	4,2479073	2,16E-05	0,0002001	PPAP2C
ENSGALG00000006003	58,995483	1,10329195	0,3504724	3,1480138	0,0016438	0,0080223	C10orf10
ENSGALG00000001281	270,65777	1,101845994	0,2489516	4,4259451	9,60E-06	9,94E-05	PHOSPHO1
ENSGALG00000006407	926,88526	1,100961832	0,1782262	6,1773296	6,52E-10	1,58E-08	
ENSGALG00000011003	180,05065	1,099169303	0,2856147	3,8484334	0,0001189	0,0008669	SLC35F3
ENSGALG00000000168	20,237218	1,099055711	0,3363625	3,2674737	0,0010851	0,0057152	ADORA1
ENSGALG00000014798	1444,6552	1,098754437	0,1853247	5,9288075	3,05E-09	6,69E-08	RP11-296A16.1
ENSGALG00000015076	118,81874	1,094512369	0,3047536	3,5914667	0,0003288	0,0021068	
ENSGALG00000002579	438,86248	1,09231668	0,2407976	4,5362436	5,73E-06	6,21E-05	RIMBP2
ENSGALG00000027102	27,931361	1,090659115	0,3485566	3,129073	0,0017536	0,0084616	
ENSGALG00000007740	159,04973	1,089704597	0,297943	3,6574259	0,0002548	0,0016854	
ENSGALG00000004972	200,69424	1,089536909	0,3028577	3,5975215	0,0003213	0,0020613	ALKBH5
ENSGALG00000004538	131,20236	1,088596778	0,3003085	3,6249277	0,000289	0,001882	TNN
ENSGALG00000005470	915,92774	1,087631823	0,1770332	6,1436603	8,06E-10	1,93E-08	LPPR5
ENSGALG00000006189	81,047665	1,08750661	0,3396623	3,2017288	0,0013661	0,0069166	CRYGN
ENSGALG00000001716	219,08602	1,084481493	0,276627	3,9203751	8,84E-05	0,0006759	C15orf59
ENSGALG00000017106	609,78404	1,082115941	0,1927819	5,6131603	1,99E-08	3,72E-07	ATP8A2
ENSGALG00000014264	24,119483	1,081789708	0,3421863	3,1614055	0,0015701	0,0077368	NSUN7
ENSGALG00000001470	804,49858	1,081645785	0,1818986	5,9464233	2,74E-09	6,10E-08	STOML1
ENSGALG00000023636	80,78387	1,079731402	0,3357478	3,2159003	0,0013004	0,0066483	REM1
ENSGALG00000021303	70,179062	1,078464744	0,3387078	3,1840559	0,0014523	0,0072578	NPY7R
ENSGALG00000011353	167,02407	1,077984355	0,2948088	3,6565536	0,0002556	0,0016887	TNNI3K
ENSGALG00000027354	35,225101	1,077448801	0,3534209	3,048628	0,0022989	0,0105741	MPND
ENSGALG00000026651	469,2115	1,076820994	0,2627359	4,0984919	4,16E-05	0,0003517	SCARA3
ENSGALG00000005031	222,86675	1,076697936	0,2761103	3,8995209	9,64E-05	0,000727	DRP2
ENSGALG00000005076	658,30851	1,075685444	0,2013532	5,3422809	9,18E-08	1,49E-06	GUCY1B4
ENSGALG00000013180	23,783592	1,075255786	0,3379303	3,1818859	0,0014632	0,0072892	SLC6A18
ENSGALG00000004043	1743,1221	1,074636101	0,1810984	5,9339914	2,96E-09	6,52E-08	NBL1
ENSGALG00000008822	37,095962	1,073866227	0,3512806	3,0570039	0,0022356	0,0103284	
ENSGALG00000000420	275,78606	1,072751202	0,2433406	4,4084345	1,04E-05	0,000107	KLHDC7A
ENSGALG00000021559	51,236888	1,072736007	0,3500464	3,0645537	0,00218	0,0101332	CD14
ENSGALG00000003640	1881,5268	1,071788322	0,1472843	7,2770018	3,41E-13	1,33E-11	FAM20C
ENSGALG00000009355	164,05056	1,070935457	0,2815733	3,8033985	0,0001427	0,0010204	CLDN11
ENSGALG00000026748	19,49545	1,069944148	0,3278792	3,2632271	0,0011015	0,0057881	MYH1A
ENSGALG00000020492	36,098876	1,069561713	0,3538691	3,0224786	0,0025071	0,0113329	MUC6
ENSGALG00000004406	128,12145	1,066534863	0,3115962	3,4228111	0,0006198	0,0035892	LRRC43
ENSGALG00000006370	31,168172	1,066397762	0,3455845	3,0857802	0,0020302	0,0095644	TULP1
ENSGALG00000012377	68,881814	1,065846022	0,3384914	3,148813	0,0016394	0,0080143	HNMT
ENSGALG00000006344	48,374655	1,06541292	0,3514839	3,0311857	0,002436	0,0110921	GSTT1
ENSGALG00000010752	133,53254	1,063752645	0,3170147	3,3555312	0,0007921	0,0044138	CDCP2
ENSGALG00000021049	25,160532	1,063749147	0,3349924	3,175443	0,0014961	0,0074177	FNDC11
ENSGALG00000006374	154,22163	1,06372742	0,3461141	3,0733436	0,0021167	0,0099138	TBX6

ENSGALG00000007780	1769,7678	1,063721106	0,1641554	6,4799655	9,17E-11	2,52E-09	EIF4E3
ENSGALG00000004149	50,119553	1,06296288	0,353007	3,0111663	0,0026025	0,01169	KCNK15
ENSGALG00000021542	348,90792	1,061917275	0,2447028	4,3396212	1,43E-05	0,000141	SNCB
ENSGALG00000002055	5059,126	1,061032342	0,1289624	8,2274527	1,91E-16	1,10E-14	HES4
ENSGALG00000028742	318,00325	1,06018982	0,2447624	4,3315054	1,48E-05	0,0001453	
ENSGALG00000001040	379,37277	1,060173693	0,2265161	4,6803463	2,86E-06	3,34E-05	MEGF6
ENSGALG00000008734	1277,1497	1,060092794	0,1784849	5,9393992	2,86E-09	6,33E-08	PCSK2
ENSGALG00000025771	418,4548	1,059868939	0,2130592	4,9745277	6,54E-07	8,82E-06	PLCD3
ENSGALG00000017311	141,94595	1,059578058	0,2931335	3,6146605	0,0003007	0,0019465	P4HA3
ENSGALG00000026017	67,59259	1,059475908	0,3510549	3,0179783	0,0025447	0,0114759	DRGX
ENSGALG00000002554	211,29924	1,058725863	0,2726542	3,8830359	0,0001032	0,0007721	TSPAN2
ENSGALG00000011898	1941,7841	1,056447793	0,1400944	7,5409707	4,66E-14	2,00E-12	FAM109B
ENSGALG00000012545	20,395626	1,051934631	0,3331292	3,1577373	0,00159	0,0078066	CYTIP
ENSGALG00000016620	37,122352	1,049979269	0,351653	2,9858389	0,002828	0,0125541	PNOC
ENSGALG00000010497	795,79291	1,048579632	0,1787889	5,8649021	4,49E-09	9,56E-08	SLC29A4
ENSGALG00000000503	90,521684	1,0483641	0,3288307	3,1881578	0,0014318	0,0071802	PADI2
ENSGALG00000007959	24,992818	1,04699816	0,3339903	3,134816	0,0017196	0,0083253	SLC26A3
ENSGALG00000012045	94,161025	1,046487546	0,3346828	3,1268044	0,0017672	0,0085078	SLC12A8
ENSGALG00000009340	441,50856	1,044750001	0,2505951	4,1690755	3,06E-05	0,0002702	TRIM63
ENSGALG00000007617	161,13247	1,044420437	0,3032396	3,4442081	0,0005727	0,0033568	ANO1
ENSGALG00000006854	35,896481	1,044373279	0,3538993	2,9510467	0,003167	0,0137583	NEURL2
ENSGALG00000007837	153,44217	1,044277791	0,317273	3,2914167	0,0009968	0,0053161	KAZALD1
ENSGALG00000001252	75,259479	1,044188086	0,3423423	3,0501291	0,0022874	0,0105321	CREB3L3
ENSGALG00000009588	47,706596	1,044149084	0,3525375	2,9618103	0,0030584	0,0133846	
ENSGALG00000016020	82,686873	1,041815502	0,331022	3,1472698	0,001648	0,0080365	CLIC6
ENSGALG00000016608	1071,038	1,041630113	0,1614881	6,4501978	1,12E-10	3,01E-09	ADCY3
ENSGALG00000014258	1564,4528	1,040436704	0,1704968	6,1023832	1,04E-09	2,47E-08	LIMCH1
ENSGALG00000027096	1851,9247	1,040113834	0,1377861	7,5487579	4,39E-14	1,90E-12	LGALSL
ENSGALG00000024369	197,40751	1,038738473	0,2651565	3,917454	8,95E-05	0,0006826	
ENSGALG00000028234	617,7355	1,03829388	0,1910987	5,4332854	5,53E-08	9,37E-07	SYNDIG1
ENSGALG00000007219	1265,6281	1,038287687	0,1548697	6,7042673	2,02E-11	6,12E-10	FGF12
ENSGALG00000007253	1268,2995	1,036667095	0,1580585	6,5587573	5,43E-11	1,54E-09	IL1RAP
ENSGALG00000005460	763,14713	1,036332157	0,1841326	5,6281837	1,82E-08	3,43E-07	SLCO4A1
ENSGALG00000005951	50,333342	1,035951706	0,3500228	2,9596694	0,0030797	0,0134575	VPS29L
ENSGALG00000026714	2323,4921	1,035206481	0,1567075	6,6059781	3,95E-11	1,13E-09	SHISA2
ENSGALG00000024122	36,985836	1,03453695	0,3344358	3,0933796	0,0019789	0,0093558	TNFRSF13B
ENSGALG00000023779	766,12884	1,033222085	0,198913	5,1943408	2,05E-07	3,11E-06	TMEM82
ENSGALG00000028318	1679,8373	1,033139386	0,1849882	5,584894	2,34E-08	4,28E-07	CDKN1A
ENSGALG00000005114	245,74674	1,032666035	0,2545785	4,0563754	4,98E-05	0,0004108	CES1
ENSGALG00000028628	176,65893	1,032210406	0,27931	3,6955723	0,0002194	0,0014809	LAPTM4B
ENSGALG00000011875	520,49686	1,032103241	0,2093207	4,9307268	8,19E-07	1,08E-05	TRPC3
ENSGALG00000001304	107,91968	1,031984172	0,3164648	3,2609758	0,0011103	0,0058298	TMEM52
ENSGALG00000002459	72,761835	1,030692731	0,3400622	3,0308944	0,0024383	0,0110991	
ENSGALG00000004860	237,1229	1,03038994	0,2518626	4,0910789	4,29E-05	0,00036	RASD1
ENSGALG00000010566	1230,6364	1,029946325	0,1643356	6,2673336	3,67E-10	9,18E-09	NPNT
ENSGALG00000012791	100,57094	1,028853172	0,3257471	3,1584418	0,0015861	0,0077906	TBXAS1
ENSGALG00000024024	333,88236	1,028182094	0,2283532	4,5025956	6,71E-06	7,16E-05	TGFA
ENSGALG00000009739	1255,1864	1,027863615	0,1833266	5,6067343	2,06E-08	3,83E-07	AMIGO2
ENSGALG00000010078	138,04602	1,027109659	0,2951454	3,4800125	0,0005014	0,0030072	GLP1R
ENSGALG00000003349	284,81496	1,025286613	0,2360846	4,3428788	1,41E-05	0,0001392	PLEKHA2
ENSGALG00000009722	2700,6877	1,024969081	0,1274848	8,0399312	8,99E-16	4,78E-14	DYNC1I1
ENSGALG00000013200	141,95986	1,024952745	0,3004034	3,4119214	0,0006451	0,0037057	GABBR2
ENSGALG00000029169	143,48628	1,024627176	0,2979791	3,4385878	0,0005848	0,003414	HHEX
ENSGALG00000026420	49,217095	1,024145559	0,3514259	2,9142577	0,0035654	0,0152027	CSF3
ENSGALG00000013934	77,966051	1,022547067	0,3347704	3,0544729	0,0022546	0,0103983	FAM196A
ENSGALG00000002370	25,819164	1,022537057	0,3456971	2,957899	0,0030974	0,0135165	SH2D4B
ENSGALG00000028153	28,525881	1,022401628	0,3430351	2,9804582	0,0028782	0,012727	FOXE1
ENSGALG00000012470	43,321678	1,020606107	0,3536746	2,8857204	0,0039052	0,0163839	LYPD6
ENSGALG00000026070	69,472965	1,019730322	0,3407891	2,9922619	0,0027692	0,0123558	
ENSGALG00000025906	86,105418	1,019071961	0,3299575	3,088495	0,0020117	0,0094905	
ENSGALG00000012564	122,7387	1,018929434	0,3020158	3,3737619	0,0007415	0,0041682	
ENSGALG00000009686	819,70472	1,017336386	0,2030924	5,0092304	5,46E-07	7,54E-06	PPP1R9A

ENSGALG00000001698	101,57773	1,017252154	0,3196233	3,1826597	0,0014593	0,0072777	GABRA1
ENSGALG00000002991	1547,8463	1,016425478	0,1518876	6,6919594	2,20E-11	6,60E-10	ARID5B
ENSGALG00000001191	726,03044	1,014738695	0,1760749	5,7631095	8,26E-09	1,66E-07	MEOX1
ENSGALG00000009066	83,156898	1,013658719	0,3282182	3,0883687	0,0020126	0,0094913	KCND2
ENSGALG00000003571	35,860323	1,013482972	0,3515721	2,882717	0,0039426	0,0164962	KIF12
ENSGALG00000024381	18,525889	1,013267738	0,3321669	3,050478	0,0022848	0,0105234	LARP6L
ENSGALG00000016223	759,19748	1,013131958	0,1825727	5,5491961	2,87E-08	5,19E-07	MAOB
ENSGALG00000012307	29,303894	1,012697653	0,3486352	2,904749	0,0036755	0,015599	GALR3
ENSGALG00000010203	639,91361	1,012100046	0,1964193	5,1527526	2,57E-07	3,79E-06	
ENSGALG00000017362	22,179359	1,011846131	0,3421939	2,9569383	0,0031071	0,0135504	
ENSGALG00000008874	19,446543	1,010524458	0,323522	3,1235106	0,0017871	0,0085793	SLC13A1
ENSGALG00000022718	221,71371	1,010197798	0,2618613	3,8577596	0,0001144	0,0008403	FUT4
ENSGALG00000014455	15,37556	1,009369671	0,3151604	3,2027176	0,0013614	0,0068955	LPAR5
ENSGALG00000008080	251,32912	1,008867324	0,2511963	4,01625	5,91E-05	0,0004755	C1QTNF4
ENSGALG00000021655	77,574154	1,008428495	0,3474014	2,902776	0,0036987	0,015683	BCL2L15
ENSGALG00000001951	315,95633	1,007959576	0,2412905	4,1773687	2,95E-05	0,0002612	FAM102B
ENSGALG00000025876	51,163718	1,007886002	0,3524372	2,8597604	0,0042396	0,0175022	THPO
ENSGALG00000027712	289,19222	1,006836187	0,246269	4,0883601	4,34E-05	0,0003633	
ENSGALG00000005094	32,433793	1,005780474	0,350919	2,8661325	0,0041552	0,0172007	GRAP
ENSGALG00000028186	897,73377	1,005437673	0,1860189	5,4050306	6,48E-08	1,09E-06	RERG
ENSGALG00000003816	91,378339	1,005135037	0,3271198	3,0726821	0,0021214	0,009926	NT5C1A
ENSGALG00000004653	66,385404	1,003752617	0,3500053	2,8678215	0,0041331	0,0171352	CDH23
ENSGALG00000007713	153,5491	1,002069085	0,297438	3,3690019	0,0007544	0,0042333	CD300A
ENSGALG00000026584	85,189769	1,001767633	0,3522109	2,844227	0,0044519	0,0181912	
ENSGALG00000016251	6835,3703	1,000647408	0,1546994	6,4683351	9,91E-11	2,69E-09	TSPAN7
ENSGALG00000028962	99,714152	1,000589641	0,3302214	3,030057	0,0024451	0,0111225	
ENSGALG00000023953	637,17536	0,998916427	0,1866162	5,3527844	8,66E-08	1,41E-06	
ENSGALG00000005887	163,6325	0,998359123	0,3075104	3,2465868	0,001168	0,0060741	PXDNL
ENSGALG00000009511	29,357161	0,996402413	0,3503011	2,8444172	0,0044493	0,0181858	SI
ENSGALG00000012480	541,53262	0,99597856	0,2064035	4,8253958	1,40E-06	1,76E-05	NMI
ENSGALG00000014681	398,45148	0,995892967	0,2311425	4,3085667	1,64E-05	0,0001581	PCSK1
ENSGALG00000000172	20,017715	0,995836198	0,3363958	2,9603109	0,0030733	0,013437	MYOG
ENSGALG00000017316	218,98095	0,995598244	0,3012626	3,3047519	0,0009506	0,0051298	UCP3
ENSGALG00000007045	32,468926	0,994891941	0,3526069	2,8215327	0,0047795	0,0193208	GPR123
ENSGALG00000005467	75,167039	0,994871662	0,3408309	2,9189596	0,003512	0,0150035	HSD17B2
ENSGALG00000001453	365,61787	0,994068241	0,2454962	4,0492198	5,14E-05	0,0004207	FAM110D
ENSGALG00000002437	722,53208	0,993124174	0,1885536	5,2670664	1,39E-07	2,17E-06	FAM69B
ENSGALG00000012613	121,97261	0,992227725	0,3163097	3,1368866	0,0017075	0,0082733	FBP1
ENSGALG00000015094	84,375027	0,9904996	0,3373138	2,9364333	0,0033201	0,0142958	ZBTB20
ENSGALG00000028682	130,82591	0,990483134	0,3063312	3,2333734	0,0012234	0,006314	
ENSGALG00000013183	389,46671	0,988585254	0,2164446	4,5673816	4,94E-06	5,48E-05	TERT
ENSGALG00000002766	25,140518	0,98837641	0,3444585	2,8693625	0,004113	0,0170883	MLKL
ENSGALG00000009638	342,69847	0,988233263	0,2260661	4,3714351	1,23E-05	0,0001244	ANO6
ENSGALG00000016224	196,09798	0,988198095	0,2772614	3,5641386	0,0003651	0,0022968	MAOA
ENSGALG00000009052	3269,1548	0,987910707	0,1624129	6,0827127	1,18E-09	2,78E-08	LBH
ENSGALG00000026042	849,9138	0,987626868	0,2371785	4,1640662	3,13E-05	0,0002751	SHISA3
ENSGALG00000012536	763,86305	0,987580114	0,2116261	4,6666263	3,06E-06	3,54E-05	GALNT13
ENSGALG00000016599	16,001403	0,987073109	0,3131543	3,1520339	0,0016214	0,0079321	MFAP4
ENSGALG00000008638	22,301546	0,987044947	0,3280366	3,008948	0,0026215	0,011768	
ENSGALG00000009463	2838,7047	0,986691121	0,1403118	7,0321343	2,03E-12	7,16E-11	DCAF5
ENSGALG00000006812	66,925337	0,985329432	0,3466414	2,8425032	0,0044761	0,0182625	TTC36
ENSGALG00000001764	93,207252	0,985279854	0,3248167	3,0333416	0,0024186	0,0110206	HCN4
ENSGALG00000005030	1142,7214	0,984706358	0,1778942	5,5353473	3,11E-08	5,55E-07	DOCK10
ENSGALG00000009488	49,450774	0,98346472	0,3539444	2,7785852	0,0054596	0,0216083	LRRN3
ENSGALG00000027429	108,06142	0,983232181	0,3123358	3,1479968	0,0016439	0,0080223	HSD17B1
ENSGALG00000011203	97,875883	0,983177315	0,3203781	3,0688031	0,0021492	0,0100141	HPSE
ENSGALG00000005419	103,38309	0,983150481	0,3221858	3,0515019	0,002277	0,0104911	ERC2
ENSGALG00000015172	24,235396	0,982809248	0,3441667	2,85562	0,0042953	0,0176945	
ENSGALG00000023847	205,80734	0,981888346	0,2682741	3,6600198	0,0002522	0,0016692	
ENSGALG00000012552	632,63312	0,981819434	0,1820243	5,3938935	6,89E-08	1,15E-06	TOM1
ENSGALG00000027739	223,97962	0,981670308	0,2808826	3,4949492	0,0004742	0,0028704	CPNE9
ENSGALG00000000394	39,052306	0,981204777	0,3535388	2,7753805	0,0055137	0,0217718	SLC6A17

ENSGALG00000005883	37,607236	0,980233834	0,3534383	2,7734226	0,005547	0,0218779	BRICD5
ENSGALG00000012715	380,22403	0,978917656	0,2453996	3,9890758	6,63E-05	0,0005244	CAP2
ENSGALG00000012709	399,32692	0,978132572	0,259146	3,774446	0,0001604	0,0011301	ATXN1
ENSGALG00000027489	17,903941	0,978014836	0,3301784	2,9620804	0,0030557	0,0133772	
ENSGALG00000026754	127,90019	0,977513049	0,3209323	3,0458545	0,0023202	0,0106577	
ENSGALG00000028301	585,85613	0,976931086	0,2053037	4,7584672	1,95E-06	2,36E-05	TRIM47
ENSGALG00000006882	2766,7066	0,976472658	0,130813	7,4646479	8,35E-14	3,47E-12	ST8SIA2
ENSGALG00000026027	52,610545	0,976263156	0,3534975	2,7617256	0,0057497	0,0224496	CLIC3
ENSGALG00000024059	15,053333	0,976199065	0,3125125	3,1237123	0,0017858	0,0085765	PGA
ENSGALG00000010797	412,00234	0,975073157	0,2100324	4,6424888	3,44E-06	3,95E-05	SOSTDC1
ENSGALG00000000902	273,8179	0,974915117	0,2442269	3,9918423	6,56E-05	0,0005187	KDF1
ENSGALG00000004591	574,2787	0,974905454	0,1969693	4,949529	7,44E-07	9,89E-06	SERPINC1
ENSGALG00000016715	46,103475	0,974213716	0,3521064	2,7668161	0,0056607	0,0221976	MEP1A
ENSGALG00000016944	4957,4542	0,973864805	0,3289279	2,9607247	0,0030692	0,0134233	PCDH8
ENSGALG00000018875	14,740412	0,973272591	0,3123576	3,1158922	0,0018339	0,0087609	ACSM4
ENSGALG00000004721	51,579363	0,972599162	0,3523855	2,7600432	0,0057794	0,0225397	KCNJ12
ENSGALG00000003886	1023,8773	0,971801068	0,1773944	5,4781962	4,30E-08	7,44E-07	SGCD
ENSGALG00000003069	21,65475	0,971652009	0,3390802	2,8655523	0,0041628	0,017227	TNFSF4
ENSGALG00000023869	23,260449	0,971412526	0,3437175	2,8261945	0,0047105	0,0190805	
ENSGALG00000019231	26,295329	0,970164482	0,3464496	2,8003049	0,0051054	0,0203963	CD200R1
ENSGALG00000023439	59,847743	0,969553596	0,3512997	2,7599043	0,0057818	0,0225428	
ENSGALG00000019325	54,610886	0,96951089	0,3507875	2,7638126	0,005713	0,0223258	
ENSGALG00000005844	1501,3882	0,969445519	0,1528693	6,3416622	2,27E-10	5,87E-09	CASKIN1
ENSGALG00000016820	787,30983	0,968970008	0,1783249	5,4337332	5,52E-08	9,36E-07	GAS6
ENSGALG00000027287	736,98606	0,968636988	0,1893148	5,11654	3,11E-07	4,50E-06	HK3
ENSGALG00000003170	1633,7315	0,967045053	0,1741681	5,552367	2,82E-08	5,10E-07	DNAH10
ENSGALG00000026870	2578,9703	0,966911139	0,1632907	5,9214111	3,19E-09	6,99E-08	TMEM200C
ENSGALG00000027511	3914,707	0,966828969	0,1525889	6,3361698	2,36E-10	6,06E-09	SP5
ENSGALG00000021275	22,663889	0,966605206	0,3386389	2,8543832	0,004312	0,0177474	LTC4S
ENSGALG00000004734	21,747155	0,966247375	0,3385892	2,8537457	0,0043207	0,0177582	
ENSGALG00000015418	42,123895	0,966243505	0,353786	2,7311521	0,0063113	0,0242874	EPHA6
ENSGALG00000001209	151,27678	0,963839438	0,2885245	3,3405805	0,000836	0,0046009	TP53INP2
ENSGALG00000008932	24,822651	0,962818941	0,3469054	2,7754514	0,0055125	0,0217718	ENTPD8
ENSGALG00000012681	687,54966	0,96179459	0,1933738	4,9737578	6,57E-07	8,84E-06	MBOAT1
ENSGALG00000026914	303,14643	0,960831377	0,2341299	4,1038388	4,06E-05	0,0003453	DOK3
ENSGALG00000016597	546,5809	0,96044418	0,2115615	4,5397871	5,63E-06	6,12E-05	STMN4
ENSGALG00000006911	92,463176	0,960163411	0,3331134	2,8823917	0,0039467	0,0165082	TMC5
ENSGALG00000028041	172,71276	0,96010795	0,292817	3,2788668	0,0010422	0,0055242	DKK2
ENSGALG00000008317	155,9685	0,958791686	0,2982524	3,2146994	0,0013058	0,0066662	SDSL
ENSGALG00000006192	588,22456	0,958606154	0,1986953	4,824504	1,40E-06	1,77E-05	USH1C
ENSGALG00000012285	88,550664	0,958415036	0,3255253	2,9442104	0,0032378	0,0140066	BAIAP2L2
ENSGALG00000017274	44,802442	0,957455073	0,3539461	2,7050872	0,0068286	0,0258936	CD9
ENSGALG00000009896	1725,9341	0,956710434	0,138947	6,8854343	5,76E-12	1,88E-10	TRERF1
ENSGALG00000008936	346,61065	0,956545148	0,2393518	3,9963977	6,43E-05	0,0005109	ENTPD8L
ENSGALG00000012577	1716,8746	0,956309243	0,1378913	6,9352415	4,06E-12	1,35E-10	FRMD3
ENSGALG00000006375	24,972242	0,956253302	0,3445218	2,7755957	0,0055101	0,0217718	TM4SF19
ENSGALG00000012271	67,173186	0,955959348	0,3478063	2,7485396	0,0059861	0,0232197	MTTP
ENSGALG00000023271	71,780111	0,955084764	0,3439036	2,7771873	0,0054832	0,0216826	
ENSGALG00000016945	3627,6803	0,953551228	0,1270066	7,5078871	6,01E-14	2,53E-12	LECT1
ENSGALG00000003209	25,968303	0,953438931	0,3318207	2,8733556	0,0040614	0,0169048	NODAL
ENSGALG00000014843	607,09399	0,953307805	0,2111335	4,5151905	6,33E-06	6,80E-05	TPD52L1
ENSGALG00000029150	2513,115	0,953288378	0,1486082	6,414778	1,41E-10	3,76E-09	AK1
ENSGALG00000005118	32,326575	0,952825585	0,3518222	2,7082585	0,0067637	0,0257069	
ENSGALG00000000295	158,36113	0,951838119	0,2874721	3,3110626	0,0009294	0,0050415	IGFN1
ENSGALG00000002008	770,02151	0,951550674	0,2636914	3,6085762	0,0003079	0,0019866	RNF157
ENSGALG00000009216	17,250683	0,951287914	0,3202238	2,970697	0,0029712	0,0130664	
ENSGALG00000005472	26,534994	0,951255745	0,3483401	2,7308243	0,0063176	0,024288	NAT
ENSGALG00000017926	117,34516	0,950771836	0,3049521	3,1177741	0,0018222	0,0087174	
ENSGALG00000009123	138,33508	0,94996493	0,2943779	3,227025	0,0012508	0,006434	
ENSGALG00000017068	94,861308	0,947287623	0,3314816	2,8577385	0,0042667	0,0175875	KL
ENSGALG00000021696	69,405082	0,947251279	0,3378068	2,8041212	0,0050454	0,0202098	TRIM50
ENSGALG00000026793	20,910942	0,946537074	0,3217404	2,9419277	0,0032618	0,0140891	CYP2C8

ENSGALG00000011046	918,66681	0,946061534	0,2245408	4,2133163	2,52E-05	0,0002287	SNX10
ENSGALG00000004792	282,6657	0,945718297	0,2609466	3,6241839	0,0002899	0,0018856	PLCB2
ENSGALG00000001091	62,437953	0,945610586	0,3444618	2,7451826	0,0060477	0,0234186	C1orf116
ENSGALG00000005857	90,998254	0,944585535	0,322613	2,9279211	0,0034124	0,0146283	ALOX5
ENSGALG00000006988	545,9632	0,944077449	0,2380928	3,9651665	7,33E-05	0,0005736	RNF213
ENSGALG00000009002	180,8252	0,943061124	0,2745333	3,4351427	0,0005922	0,0034533	C7ORF58
ENSGALG00000003370	6860,8975	0,941055859	0,1158875	8,120424	4,65E-16	2,56E-14	KIAA1191
ENSGALG00000015183	541,00251	0,940741895	0,2469295	3,8097593	0,0001391	0,0009994	
ENSGALG00000010977	629,76264	0,94045823	0,1853813	5,0731011	3,91E-07	5,55E-06	FAM221A
ENSGALG00000017169	2527,4044	0,94013642	0,1550564	6,0631902	1,33E-09	3,11E-08	ELMOD1
ENSGALG00000006351	260,93159	0,940035694	0,256606	3,6633421	0,0002489	0,0016526	FAS
ENSGALG00000016699	24,25844	0,939513809	0,3453851	2,7201919	0,0065244	0,0249662	OPN5L2
ENSGALG00000007415	599,16556	0,938743099	0,2370594	3,9599488	7,50E-05	0,0005829	SH3RF2
ENSGALG00000002678	150,0779	0,938682309	0,293615	3,1969839	0,0013887	0,007008	CSPG4
ENSGALG00000003832	1422,7636	0,938218604	0,1475404	6,3590641	2,03E-10	5,31E-09	TEX264
ENSGALG00000009725	763,67798	0,938023358	0,224257	4,182805	2,88E-05	0,0002563	SCG5
ENSGALG00000015967	204,24999	0,936813576	0,2634911	3,5553898	0,0003774	0,0023603	LRRTM1
ENSGALG00000003435	45,697878	0,936462652	0,3537379	2,6473351	0,0081129	0,0297327	PGC
ENSGALG00000002473	480,3177	0,93617222	0,2026166	4,6204127	3,83E-06	4,35E-05	GPR157
ENSGALG00000015926	1110,0554	0,934566237	0,1598776	5,84551	5,05E-09	1,06E-07	RUNX1T1
ENSGALG00000019312	16,25864	0,934023396	0,3177972	2,939055	0,0032921	0,0141844	
ENSGALG00000001398	801,87151	0,933503925	0,1756569	5,3143584	1,07E-07	1,71E-06	
ENSGALG00000010337	96,826556	0,93241733	0,319691	2,9166205	0,0035385	0,0151022	GPR149
ENSGALG00000028292	453,15086	0,931661788	0,2502644	3,7227094	0,0001971	0,0013471	FAM19A4
ENSGALG00000008735	158,65028	0,931477748	0,2951381	3,1560747	0,0015991	0,0078456	BFSP1
ENSGALG00000010139	381,67431	0,931031468	0,2175137	4,2803349	1,87E-05	0,0001763	SSTR1
ENSGALG00000002843	586,86661	0,930974563	0,1932115	4,8184228	1,45E-06	1,82E-05	RILP
ENSGALG00000001694	115,671	0,929649369	0,3250641	2,8598955	0,0042378	0,0175001	FER1L4
ENSGALG00000001287	117,66472	0,929543301	0,3091097	3,0071632	0,002637	0,0118295	ABI3
ENSGALG00000023626	1834,269	0,929476271	0,1654852	5,6166736	1,95E-08	3,65E-07	NTN1
ENSGALG00000002083	14,444586	0,928533788	0,3085021	3,0098139	0,0026141	0,0117383	PLIN3
ENSGALG00000027053	71,270094	0,927920334	0,3515108	2,6398059	0,0082954	0,0302791	C18orf42
ENSGALG00000028091	182,30387	0,927849672	0,2899177	3,2003896	0,0013724	0,0069433	MYF5
ENSGALG00000025801	28,789593	0,927018359	0,349612	2,6515632	0,008012	0,0294645	
ENSGALG00000000866	124,56244	0,926965653	0,3251101	2,8512364	0,004355	0,0178646	SELENBP1
ENSGALG00000006388	73,461217	0,926517942	0,3489684	2,6550197	0,0079304	0,029237	IL16
ENSGALG00000000919	14,595204	0,925422799	0,3081645	3,0030152	0,0026732	0,0119683	PIGR
ENSGALG00000023517	259,64579	0,925400404	0,2568731	3,602558	0,0003151	0,0020246	AGPAT2
ENSGALG00000005626	609,64517	0,92496767	0,2159097	4,2840488	1,84E-05	0,0001735	TPST2
ENSGALG00000001035	102,60165	0,924878686	0,3465453	2,6688536	0,0076111	0,028305	NR1D1
ENSGALG00000020233	23,832939	0,924086016	0,3386279	2,7289127	0,0063544	0,0243978	
ENSGALG00000000311	558,40847	0,92295118	0,2153587	4,2856468	1,82E-05	0,0001725	DOCK5
ENSGALG00000014988	88,836245	0,920904288	0,3383615	2,7216578	0,0064955	0,0248884	POPDC2
ENSGALG00000015207	1291,1294	0,92039826	0,1472006	6,2526786	4,03E-10	1,00E-08	F5
ENSGALG00000010417	18,919028	0,920255863	0,3320234	2,77166	0,0055771	0,0219585	VDHAP
ENSGALG00000010288	20,644022	0,918819724	0,3351049	2,7418866	0,0061087	0,0236079	
ENSGALG00000023936	805,04939	0,918539777	0,2012641	4,5638532	5,02E-06	5,56E-05	
ENSGALG00000009509	42,581938	0,91836029	0,3539579	2,5945464	0,0094716	0,0336435	CALCR
ENSGALG00000011709	513,36272	0,918333355	0,2317844	3,9620158	7,43E-05	0,0005792	TMEM108
ENSGALG00000027622	398,87687	0,916634803	0,2658647	3,4477494	0,0005653	0,0033245	ADAM11
ENSGALG00000015795	539,93954	0,91628777	0,2153216	4,2554383	2,09E-05	0,0001946	ADAMTS5
ENSGALG00000005120	2531,22	0,916224882	0,1549653	5,9124511	3,37E-09	7,37E-08	FBLN2
ENSGALG00000010736	93,908737	0,914854903	0,3255588	2,8101064	0,0049525	0,0199374	DIO1
ENSGALG00000013071	1399,4771	0,914850391	0,17423	5,2508198	1,51E-07	2,35E-06	CALD1
ENSGALG00000010382	346,13588	0,914779385	0,2280121	4,0119779	6,02E-05	0,0004822	LRRC73
ENSGALG00000004309	938,33548	0,913060877	0,1855348	4,9212369	8,60E-07	1,13E-05	GDAP1L1
ENSGALG00000028622	281,67527	0,912974481	0,2559896	3,566452	0,0003618	0,0022808	PEAR1
ENSGALG00000017214	131,47371	0,912890352	0,3103783	2,9412185	0,0032692	0,0141048	HEPHL1
ENSGALG00000016013	42,116835	0,912662466	0,3534877	2,5818789	0,0098264	0,0347137	HTR7L
ENSGALG00000026687	69,587609	0,912634606	0,347276	2,6279803	0,0085893	0,0311435	PLCD4
ENSGALG00000005331	677,48087	0,912514017	0,1798939	5,0725131	3,93E-07	5,56E-06	GJC2
ENSGALG00000003529	116,76012	0,912498344	0,3196723	2,8544806	0,0043107	0,0177474	SYNC

ENSGALG00000027462	1468,6722	0,912376278	0,1432394	6,369589	1,90E-10	4,98E-09	LEPREL4
ENSGALG00000015789	559,92994	0,912066241	0,2123869	4,2943618	1,75E-05	0,000167	CNR1
ENSGALG00000026563	13,690632	0,912028647	0,3072132	2,9687161	0,0029905	0,0131255	HAPLN4
ENSGALG00000010551	919,31695	0,911984879	0,1832286	4,9773072	6,45E-07	8,70E-06	CDC42EP3
ENSGALG00000012298	550,04797	0,911513817	0,2224948	4,0967868	4,19E-05	0,0003536	SLC39A8
ENSGALG00000003588	1398,6038	0,911216657	0,1584852	5,7495378	8,95E-09	1,78E-07	
ENSGALG00000005814	212,00295	0,910795359	0,271855	3,3502982	0,0008072	0,0044752	
ENSGALG00000002581	512,90179	0,910753526	0,1951976	4,6658018	3,07E-06	3,56E-05	HTATIP2
ENSGALG00000004676	1678,5831	0,910530695	0,1433097	6,3535879	2,10E-10	5,48E-09	BTG3
ENSGALG00000008727	92,320134	0,910292988	0,3286188	2,7700573	0,0056046	0,022035	
ENSGALG00000023195	260,80986	0,909179266	0,2471713	3,6783362	0,0002348	0,0015683	DLX6
ENSGALG00000027013	1552,1008	0,908413942	0,3505249	2,5915816	0,0095536	0,0338377	HOX1
ENSGALG00000015725	901,98347	0,907979483	0,1701411	5,3366278	9,47E-08	1,53E-06	FGFRL1
ENSGALG00000007114	305,81852	0,907573399	0,2767971	3,2788398	0,0010423	0,0055242	APOA1
ENSGALG00000010635	38,448493	0,906970906	0,3515336	2,5800407	0,0098789	0,0348087	
ENSGALG00000011213	25,560047	0,906880966	0,3462592	2,619081	0,0088167	0,0317985	DEGS2
ENSGALG00000019635	32,562895	0,90677452	0,3521651	2,5748562	0,0100282	0,0352345	PP2D1
ENSGALG00000006202	116,81237	0,906421176	0,31598	2,8686028	0,0041229	0,0171086	SCNN1B
ENSGALG00000003018	309,19026	0,906093174	0,2488521	3,6410907	0,0002715	0,0017771	SCARB1
ENSGALG00000016076	120,93641	0,906052944	0,3046837	2,9737494	0,0029419	0,0129706	DPYS
ENSGALG00000015495	24,536119	0,904690337	0,3465615	2,6104756	0,0090416	0,0324294	POU1F1
ENSGALG00000013886	841,1366	0,904236576	0,1804703	5,0104453	5,43E-07	7,51E-06	LDLRAD4
ENSGALG00000005795	32,624744	0,904049889	0,343537	2,6315941	0,0084985	0,0308718	CYP2H1
ENSGALG00000021442	473,2158	0,903637173	0,225822	4,0015457	6,29E-05	0,000501	RLTPR
ENSGALG00000005014	159,55971	0,903243848	0,2894313	3,1207539	0,0018039	0,0086448	AIPL1
ENSGALG00000002963	189,40667	0,902926335	0,2699116	3,3452673	0,000822	0,0045354	PID1
ENSGALG00000000836	482,38155	0,902335572	0,2085245	4,3272398	1,51E-05	0,0001475	RFX5
ENSGALG00000011124	436,07123	0,902068067	0,2097494	4,3006934	1,70E-05	0,000163	ARHGAP24
ENSGALG00000010827	4357,4834	0,901311467	0,1146698	7,8600622	3,84E-15	1,84E-13	ITPK1
ENSGALG00000006756	144,39	0,900987054	0,2996417	3,0068817	0,0026394	0,0118366	BPI
ENSGALG00000006740	150,08447	0,900492229	0,3014742	2,9869628	0,0028176	0,0125162	
ENSGALG00000008461	387,65672	0,900341356	0,2481083	3,6288245	0,0002847	0,0018574	HGF
ENSGALG00000016761	58,000294	0,90015721	0,3467952	2,5956447	0,0094414	0,0335537	LYG2
ENSGALG00000010119	1290,7566	0,900097678	0,1879287	4,78957	1,67E-06	2,06E-05	CDKL2
ENSGALG00000020905	199,73795	0,899806675	0,2823411	3,1869491	0,0014378	0,007205	KSR2
ENSGALG00000009737	97,351691	0,899099989	0,342334	2,6263823	0,0086298	0,0312485	TAC1
ENSGALG00000006793	399,96964	0,898163749	0,2122528	4,2315762	2,32E-05	0,0002129	NTRK3
ENSGALG00000008632	774,98804	0,897411708	0,1727746	5,1941169	2,06E-07	3,11E-06	ACSS1
ENSGALG00000001896	633,5555	0,897398544	0,1829807	4,9043349	9,37E-07	1,23E-05	NTNG1
ENSGALG00000006083	883,4583	0,896818662	0,1847643	4,8538537	1,21E-06	1,55E-05	
ENSGALG00000023772	19,974194	0,896459603	0,3370772	2,6595087	0,0078255	0,02896	HSPB7
ENSGALG00000005853	55,709692	0,89635133	0,3538655	2,5330285	0,0113082	0,0387543	HTR2C
ENSGALG00000007052	507,96535	0,895651499	0,203478	4,4017107	1,07E-05	0,0001099	PXK
ENSGALG00000015977	788,48732	0,89563673	0,2070525	4,3256514	1,52E-05	0,0001481	SMOX
ENSGALG00000005349	125,03741	0,895405672	0,3020632	2,9642993	0,0030337	0,0132897	MSLN
ENSGALG00000005085	110,02771	0,895178522	0,3179722	2,8152725	0,0048736	0,0196662	DRD4
ENSGALG00000008262	171,78981	0,894529697	0,2811864	3,1812692	0,0014663	0,0072994	RASGRF1
ENSGALG00000000374	1105,8431	0,894239416	0,1877046	4,7640791	1,90E-06	2,30E-05	RNPEP
ENSGALG00000017204	419,66256	0,891856915	0,2224702	4,0088824	6,10E-05	0,000488	SESN3
ENSGALG00000002854	440,26532	0,890941536	0,2420509	3,6808025	0,0002325	0,0015558	PDZD2
ENSGALG00000008599	20,776579	0,889603293	0,3339725	2,6637025	0,0077286	0,0286811	
ENSGALG00000005203	773,68467	0,889220424	0,2133641	4,1676197	3,08E-05	0,0002718	OLFM3
ENSGALG00000027603	933,53527	0,889190298	0,1666499	5,335677	9,52E-08	1,54E-06	ZHX3
ENSGALG00000003932	494,66101	0,888085177	0,2291723	3,8751853	0,0001065	0,000794	SDC4
ENSGALG00000007388	193,87974	0,887520344	0,3042223	2,9173416	0,0035303	0,0150721	SRRM4
ENSGALG00000013265	26,768688	0,887507398	0,3491977	2,5415617	0,0110358	0,0380484	GYS2
ENSGALG00000001917	36,330226	0,88709327	0,3537861	2,5074281	0,0121613	0,0410137	SSC4D
ENSGALG00000021666	75,893072	0,886771767	0,3536689	2,5073499	0,012164	0,0410137	TMEM240
ENSGALG00000010269	1110,1934	0,886301871	0,1651233	5,3675161	7,98E-08	1,31E-06	KCNAB1
ENSGALG00000004093	635,62125	0,885654514	0,2097536	4,2223558	2,42E-05	0,0002211	TBX22
ENSGALG00000009256	32,540437	0,884987978	0,3436981	2,5748992	0,0100269	0,0352345	LEFTY2
ENSGALG00000004729	91,741024	0,88490464	0,3219745	2,7483686	0,0059893	0,0232252	SLC7A10

ENSGALG00000008722	81,303501	0,884773994	0,3362693	2,6311471	0,0085097	0,030896	MCOLN2
ENSGALG00000015788	630,45937	0,884593035	0,2015792	4,3883151	1,14E-05	0,000116	CHMP4C
ENSGALG00000004970	368,25658	0,884420332	0,2518256	3,5120356	0,0004447	0,0027174	SVOP
ENSGALG00000026152	376,28373	0,884418189	0,2320545	3,8112524	0,0001383	0,0009939	GBP
ENSGALG00000002252	31,22735	0,883701655	0,3473707	2,5439732	0,0109599	0,0378538	
ENSGALG00000010862	27,986731	0,883307692	0,3482432	2,5364677	0,0111977	0,0384768	FERD3L
ENSGALG00000004854	90,659457	0,882798305	0,3265019	2,7038079	0,006855	0,0259757	
ENSGALG00000025733	660,61374	0,882587352	0,1955092	4,5143008	6,35E-06	6,83E-05	
ENSGALG00000006008	1270,6534	0,882382705	0,1743852	5,0599632	4,19E-07	5,90E-06	HOMER2
ENSGALG00000027027	1969,0809	0,882215122	0,163127	5,4081483	6,37E-08	1,07E-06	FBXL7
ENSGALG00000011235	63,312192	0,882157118	0,3457444	2,5514721	0,0107269	0,0372093	GALNT15
ENSGALG00000003270	387,81088	0,881994372	0,2135989	4,1292087	3,64E-05	0,0003135	DRD1
ENSGALG00000020160	119,73819	0,881263086	0,3087394	2,8543913	0,0043119	0,0177474	ADH6
ENSGALG00000011234	31,35804	0,880790396	0,3529516	2,4954988	0,012578	0,0422005	INHA
ENSGALG00000001519	27,987195	0,879960975	0,3507261	2,5089692	0,0121084	0,0408565	NEU2
ENSGALG00000009580	19,504731	0,879798414	0,32149	2,7366276	0,0062073	0,0239207	IQCJ
ENSGALG00000016986	175,40028	0,879609126	0,2982944	2,9487952	0,0031902	0,0138545	LCP1
ENSGALG00000008349	209,56657	0,879571782	0,2609378	3,3708107	0,0007495	0,0042079	LMCD1
ENSGALG00000009601	8743,3872	0,878730197	0,1376458	6,3839954	1,73E-10	4,59E-09	NELL2
ENSGALG00000008121	42,835354	0,876566467	0,3539561	2,4764833	0,0132684	0,0439535	CYP17A1
ENSGALG00000008364	36,778976	0,876075442	0,3534635	2,4785458	0,0131919	0,0437898	
ENSGALG00000023415	29,787572	0,875042862	0,3509632	2,4932611	0,0126576	0,0424047	HMX3
ENSGALG00000027920	386,75217	0,874011674	0,2284444	3,8259279	0,0001303	0,0009425	CPLX2
ENSGALG00000026059	270,41633	0,873870501	0,2397099	3,6455337	0,0002668	0,0017508	TWIST1
ENSGALG00000004788	68,83418	0,873507388	0,3452821	2,5298369	0,0114116	0,0389981	TNFAIP8L3
ENSGALG00000011219	83,302927	0,873388378	0,3403042	2,5664933	0,0102733	0,0359195	GCGR
ENSGALG00000016017	590,76905	0,873175292	0,2154495	4,0528075	5,06E-05	0,000416	SLC4A11
ENSGALG00000005974	256,91419	0,872920311	0,2521004	3,4625904	0,000535	0,0031739	COL6A1
ENSGALG00000012422	27,355404	0,872315124	0,3483618	2,5040496	0,0122781	0,0413266	SH3BP1
ENSGALG00000026939	41,153772	0,872255445	0,3538073	2,4653407	0,0136883	0,044964	
ENSGALG00000009640	27,779879	0,872186333	0,3505999	2,487697	0,0128573	0,0429155	CHRM5
ENSGALG00000001840	243,72168	0,872138912	0,2890387	3,0173773	0,0025497	0,0114911	TEK
ENSGALG00000001129	1785,0406	0,871868135	0,1530444	5,69683	1,22E-08	2,38E-07	KIRREL3
ENSGALG00000028928	43,951223	0,871728708	0,3534532	2,4663203	0,0136509	0,0448931	LCAT
ENSGALG00000001532	54,980298	0,870029294	0,3496522	2,4882708	0,0128366	0,0428667	WNT2B
ENSGALG00000009867	212,19054	0,869748432	0,2675059	3,2513241	0,0011487	0,0059997	WIF1
ENSGALG00000027364	6291,5882	0,867638443	0,1162491	7,4636145	8,42E-14	3,48E-12	HEY1
ENSGALG00000026246	29,222799	0,867468639	0,3517182	2,4663739	0,0136489	0,0448931	GIF
ENSGALG00000020876	244,82737	0,867421463	0,2948139	2,942268	0,0032582	0,0140826	AOX2
ENSGALG00000011624	479,03518	0,867273579	0,2018671	4,2962601	1,74E-05	0,000166	PNLDC1
ENSGALG00000016384	268,59226	0,867172927	0,2685681	3,2288758	0,0012428	0,0064011	MYT1L
ENSGALG00000013515	39,808741	0,866546513	0,3539601	2,4481471	0,0143593	0,0467149	RP11-287D1.3
ENSGALG00000000225	112,7664	0,866321273	0,3372745	2,5685943	0,0102112	0,0357668	MGAT4C
ENSGALG00000011354	21,346176	0,866050474	0,3402355	2,5454444	0,0109139	0,0377138	CRYBA2
ENSGALG00000027965	101,43615	0,865747962	0,3205874	2,700505	0,0069234	0,0261695	OPRL1
ENSGALG00000026172	493,17496	0,865691508	0,2111797	4,0993117	4,14E-05	0,0003508	CYBA
ENSGALG00000016115	186,82276	0,864642259	0,2830702	3,0545156	0,0022542	0,0103983	SAMD12
ENSGALG00000027380	43,976414	0,862762922	0,3539532	2,4375054	0,014789	0,0478271	
ENSGALG00000002206	95,268458	0,862737257	0,3285875	2,6255938	0,0086498	0,031296	SNX22
ENSGALG00000019804	21,501251	0,862231301	0,3371774	2,5572037	0,0105517	0,0367422	
ENSGALG00000003895	788,02112	0,861640305	0,2589823	3,3270233	0,0008778	0,0047996	PRDM12
ENSGALG00000012072	201,72423	0,861021311	0,2753299	3,1272347	0,0017646	0,0084984	PARP14
ENSGALG00000007838	40,646821	0,860354034	0,3539557	2,4306829	0,0150704	0,0485568	NLRC3
ENSGALG00000000953	1344,7125	0,860062109	0,1616375	5,3209304	1,03E-07	1,66E-06	TEAD3
ENSGALG00000009902	340,49684	0,859561208	0,2490936	3,4507553	0,000559	0,0032934	CFAP57
ENSGALG00000005655	299,93592	0,858231064	0,2557112	3,3562518	0,0007901	0,0044067	TSPEAR
ENSGALG00000017072	1099,8408	0,857400203	0,1810022	4,7369608	2,17E-06	2,59E-05	N4BP2L1
ENSGALG00000025919	2494,4885	0,857090747	0,142817	6,0013216	1,96E-09	4,45E-08	BAMBI
ENSGALG00000016473	44,778577	0,856849135	0,3459792	2,4765913	0,0132644	0,0439509	OSR1
ENSGALG00000017228	49,296654	0,856739492	0,3532728	2,4251498	0,0153021	0,0491478	MTNR1B
ENSGALG00000016755	172,26247	0,856689481	0,2873424	2,9814236	0,0028691	0,0126954	KIAA1211L
ENSGALG00000007376	87,397516	0,856687427	0,3445883	2,4861189	0,0129145	0,0430009	CCDC60

ENSGALG00000015768	2710,7019	0,855142896	0,1297486	6,5907703	4,38E-11	1,25E-09	ANKRD6
ENSGALG00000026896	77,09018	0,854217305	0,3380109	2,527189	0,011498	0,0392048	
ENSGALG00000012515	43,853432	0,854026842	0,3512742	2,4312255	0,0150478	0,0485029	TPPP
ENSGALG00000010664	712,1771	0,852579615	0,2288179	3,7260174	0,0001945	0,0013328	KIF26B
ENSGALG00000001189	452,19238	0,851541498	0,2063764	4,126158	3,69E-05	0,0003169	NCF1
ENSGALG00000009536	446,62772	0,851130737	0,2275785	3,7399437	0,0001841	0,0012727	
ENSGALG00000003612	268,62308	0,850682606	0,2669792	3,1863255	0,0014409	0,0072179	RNLS
ENSGALG00000023172	1255,7616	0,849498162	0,1537527	5,5250928	3,29E-08	5,85E-07	
ENSGALG00000004511	28,137324	0,848004478	0,350316	2,4206844	0,0154913	0,0496386	MTTPL
ENSGALG00000026671	2116,5692	0,847898773	0,1425868	5,9465465	2,74E-09	6,10E-08	
ENSGALG00000002742	2787,3387	0,847416181	0,1449021	5,8481963	4,97E-09	1,04E-07	TMEM132B
ENSGALG00000015670	217,38931	0,846232332	0,2802832	3,0192043	0,0025344	0,0114409	HNF4G
ENSGALG00000005791	520,60885	0,84592391	0,2039846	4,146998	3,37E-05	0,0002934	JPH3
ENSGALG00000009603	307,91481	0,844955114	0,2387657	3,5388467	0,0004019	0,0024962	
ENSGALG00000006379	1670,5263	0,844378171	0,1827468	4,6204804	3,83E-06	4,35E-05	SHH
ENSGALG00000005895	481,78824	0,844174027	0,2049844	4,1182354	3,82E-05	0,0003271	FYTTD1L
ENSGALG00000026103	166,74522	0,842618716	0,3162093	2,6647499	0,0077046	0,0286293	CACNG3
ENSGALG00000016677	26,013707	0,842209203	0,3459083	2,4347759	0,014901	0,0481322	IL17F
ENSGALG00000003952	60,952026	0,841003956	0,3435108	2,4482605	0,0143548	0,0467114	RBPJL
ENSGALG00000007043	22,708865	0,840648204	0,3395839	2,4755244	0,0133041	0,0440503	
ENSGALG00000007775	18,86724	0,840390709	0,3327596	2,525519	0,0115528	0,0393522	USH1G
ENSGALG00000000731	28,360648	0,840347147	0,3458767	2,4296148	0,0151149	0,0486392	THEMIS2
ENSGALG00000004593	2963,7081	0,839985137	0,1559067	5,3877418	7,13E-08	1,18E-06	SULF2
ENSGALG00000010857	11562,252	0,839106931	0,1136809	7,381249	1,57E-13	6,34E-12	DAB1
ENSGALG00000004946	111,2048	0,83835973	0,3100886	2,7036139	0,006859	0,0259771	COL4A4
ENSGALG00000010762	102,40709	0,837861719	0,3182395	2,6328025	0,0084684	0,0307881	OPN3
ENSGALG00000005204	101,3211	0,837525509	0,330602	2,5333346	0,0112983	0,0387374	GSTT1L
ENSGALG00000026505	1579,9758	0,837091894	0,1526832	5,48254	4,19E-08	7,29E-07	RDH10
ENSGALG00000003354	276,83001	0,837083394	0,2477788	3,3783489	0,0007292	0,0041177	KCNH4
ENSGALG00000011205	1172,7422	0,836341019	0,2011925	4,1569189	3,23E-05	0,0002826	SMOC2
ENSGALG00000005161	2738,7124	0,836184904	0,1385814	6,0338884	1,60E-09	3,70E-08	PHF2
ENSGALG00000006014	961,40069	0,836085862	0,1594344	5,2440759	1,57E-07	2,43E-06	PRKCB
ENSGALG00000007770	193,51785	0,835610241	0,2684966	3,1121814	0,0018571	0,00885	FADS6
ENSGALG00000020515	13,200692	0,834854635	0,2996969	2,7856631	0,0053418	0,0211799	
ENSGALG00000010044	118,46337	0,834748583	0,304154	2,7444935	0,0060604	0,0234611	KCNMB4
ENSGALG00000001071	194,63937	0,834483858	0,2807285	2,9725654	0,0029532	0,0130039	ELN
ENSGALG00000026098	20,591793	0,833498696	0,3202723	2,6024691	0,0092555	0,0330238	IL8L2
ENSGALG00000028582	1565,1403	0,832821984	0,1507685	5,5238471	3,32E-08	5,87E-07	LMO2
ENSGALG00000005481	1783,1099	0,83266607	0,1448108	5,7500286	8,92E-09	1,77E-07	ARHGEF3
ENSGALG00000015660	2889,8193	0,831570906	0,1228249	6,7703746	1,28E-11	4,00E-10	SNX30
ENSGALG00000028125	921,39565	0,831550629	0,1803628	4,6104334	4,02E-06	4,52E-05	CACNG4
ENSGALG00000011883	233,92984	0,831245154	0,2791409	2,9778696	0,0029026	0,0128183	CLEC3B
ENSGALG00000027201	25,956081	0,831158121	0,3370736	2,4658058	0,0136705	0,0449359	
ENSGALG00000011808	15,036213	0,830943596	0,3107832	2,6737086	0,0075018	0,0279749	CCR9
ENSGALG00000002771	197,13737	0,83021733	0,2733379	3,0373302	0,0023868	0,01091	AL928654.7
ENSGALG00000025776	414,67908	0,83014723	0,214629	3,8678243	0,0001098	0,0008117	EMID1
ENSGALG00000027903	10650,644	0,829170411	0,110533	7,501567	6,31E-14	2,64E-12	HOXB5
ENSGALG00000010500	3026,4125	0,828235743	0,1300266	6,3697426	1,89E-10	4,98E-09	NKX6-2
ENSGALG00000024487	160,03616	0,827463833	0,3258506	2,5393961	0,0111044	0,0382267	C8orf22
ENSGALG00000020002	21,954447	0,826702298	0,3346434	2,4703975	0,0134963	0,0445036	SLC22A3
ENSGALG00000007015	336,01016	0,825119263	0,2296496	3,5929492	0,000327	0,0020958	CD40
ENSGALG00000015720	662,9716	0,824601233	0,2077728	3,9687648	7,22E-05	0,000566	CHODL
ENSGALG00000011744	1298,602	0,824149013	0,1729843	4,7642989	1,90E-06	2,30E-05	KALRN
ENSGALG00000015575	115,532	0,823970797	0,3263661	2,5246826	0,0115803	0,0394262	HTRA3
ENSGALG00000017155	286,34498	0,823340146	0,2794831	2,9459393	0,0032198	0,0139435	C11orf87
ENSGALG00000016558	512,36729	0,822973317	0,2009905	4,0945888	4,23E-05	0,0003559	FIGF
ENSGALG00000002383	12,21302	0,822929288	0,2987538	2,75454	0,0058775	0,0228764	CD72AG
ENSGALG00000006833	142,93183	0,822822635	0,2953281	2,7861304	0,0053341	0,0211609	ITIH2
ENSGALG00000005662	148,91563	0,822087333	0,2869197	2,865217	0,0041672	0,01724	AMPD3
ENSGALG00000001688	9554,3654	0,822044682	0,1265194	6,497383	8,17E-11	2,27E-09	sept-05
ENSGALG00000015941	330,15649	0,819153887	0,2336045	3,5065841	0,0004539	0,0027625	AP000295.9
ENSGALG00000019768	3223,3224	0,819142269	0,1222158	6,7024262	2,05E-11	6,19E-10	ACSF2

ENSGALG00000003360	122,83324	0,818748241	0,302344	2,7080025	0,006769	0,0257069	ARL10
ENSGALG00000026276	151,57437	0,818604795	0,2887563	2,834933	0,0045835	0,0186339	TCF15
ENSGALG00000003014	83,658638	0,818530331	0,3298981	2,4811612	0,0130955	0,0435185	CHRNA3
ENSGALG00000005175	201,26271	0,818212824	0,2767102	2,9569306	0,0031072	0,0135504	BAAT
ENSGALG00000017089	188,71558	0,817752266	0,2686445	3,0439946	0,0023346	0,0107165	SLC46A3
ENSGALG00000016774	140,17126	0,817235755	0,294664	2,7734498	0,0055465	0,0218779	NPAS2
ENSGALG00000010698	1578,3511	0,816916741	0,1404823	5,8150867	6,06E-09	1,25E-07	RPS6KA5
ENSGALG00000012628	1115,1061	0,816609796	0,1882236	4,3385098	1,43E-05	0,0001415	HABP4
ENSGALG00000027830	138,16537	0,816343449	0,2952222	2,7651828	0,0056891	0,0222705	CACNG2
ENSGALG00000027534	844,14704	0,815389343	0,1740275	4,6854054	2,79E-06	3,27E-05	GM2A
ENSGALG00000023601	110,08849	0,814252411	0,3257765	2,4994207	0,0124397	0,0418084	
ENSGALG00000010397	282,5332	0,813527008	0,2534717	3,2095382	0,0013295	0,0067667	GRID2
ENSGALG00000027338	409,81098	0,813282814	0,2198892	3,6986034	0,0002168	0,0014663	
ENSGALG00000015297	1260,5465	0,81224071	0,1674371	4,8510192	1,23E-06	1,57E-05	FOXO3
ENSGALG00000000954	405,07056	0,812029159	0,2420413	3,3549193	0,0007939	0,0044207	SMPD2
ENSGALG00000028570	89,253148	0,811507626	0,3263252	2,4868067	0,0128895	0,0429494	C6orf132
ENSGALG00000006480	1820,4374	0,810690895	0,1479509	5,4794584	4,27E-08	7,41E-07	TCF7
ENSGALG00000011861	430,59758	0,810665245	0,2125716	3,8136096	0,000137	0,0009866	SYT16
ENSGALG00000011615	12,005704	0,809627409	0,2973783	2,7225505	0,006478	0,0248312	SLC22A2
ENSGALG00000009680	349,5215	0,809243098	0,2208092	3,6648968	0,0002474	0,0016447	PAQR7
ENSGALG00000001605	101,47062	0,807232306	0,3186097	2,5336084	0,0112895	0,0387248	PPM1J
ENSGALG00000015051	777,65205	0,807158582	0,1825122	4,4224914	9,76E-06	0,0001009	CDK19
ENSGALG00000013051	1092,6973	0,806546944	0,1922229	4,1958947	2,72E-05	0,0002436	SEMA5A
ENSGALG00000024090	246,87805	0,806200799	0,263668	3,0576358	0,0022309	0,0103145	UNC93B1
ENSGALG00000005147	486,92842	0,80606613	0,2005832	4,0186116	5,85E-05	0,0004713	AMY1A
ENSGALG00000013600	13,810453	0,805946686	0,307319	2,6225087	0,0087285	0,0315473	
ENSGALG00000004260	114,05372	0,805411915	0,3114119	2,5863235	0,0097006	0,0343137	PKP3
ENSGALG00000008980	97,942464	0,805130642	0,3170949	2,5390838	0,0111143	0,0382415	VWA2
ENSGALG00000017016	265,33781	0,804980828	0,247719	3,2495724	0,0011558	0,0060245	FAM124A
ENSGALG00000015608	746,79897	0,802634493	0,1733654	4,629727	3,66E-06	4,17E-05	ADRA2C
ENSGALG00000011394	463,3825	0,801850887	0,2051329	3,9089335	9,27E-05	0,0007038	CYP2J2L3
ENSGALG00000003261	114,4772	0,800174677	0,3173455	2,5214623	0,0116868	0,0397492	PTRF
ENSGALG00000007186	16,312433	0,798835823	0,3250305	2,457726	0,013982	0,0457372	C9orf91
ENSGALG00000007565	677,7029	0,798729286	0,2213224	3,6088951	0,0003075	0,0019851	PPP2R2B
ENSGALG00000027043	126,47192	0,798613446	0,3175009	2,515311	0,0118927	0,0403187	KCNH2
ENSGALG00000012073	470,07631	0,797159786	0,2039204	3,9091719	9,26E-05	0,0007038	SFRP4
ENSGALG00000008408	1323,0351	0,796075417	0,1521355	5,2326742	1,67E-07	2,57E-06	GAB3
ENSGALG00000014122	10545,592	0,795943411	0,1349861	5,8964838	3,71E-09	8,08E-08	CYB5R3
ENSGALG00000000865	119,7276	0,795679669	0,3113005	2,5559862	0,0105887	0,0368427	
ENSGALG00000014313	191,48762	0,795282607	0,2722671	2,9209643	0,0034895	0,0149206	
ENSGALG00000013489	630,05884	0,794845244	0,1872111	4,2457157	2,18E-05	0,000202	CCDC82
ENSGALG00000015233	5666,2368	0,794464087	0,1575131	5,0437964	4,56E-07	6,39E-06	ATP1B1
ENSGALG00000006507	1217,4322	0,793097237	0,1534206	5,169432	2,35E-07	3,49E-06	CLMP
ENSGALG00000017267	12,565544	0,792427514	0,2952723	2,6837179	0,0072809	0,0272932	THRSP
ENSGALG00000026605	1028,743	0,792369586	0,1581582	5,0099806	5,44E-07	7,52E-06	BAG2
ENSGALG00000007720	244,44298	0,792327844	0,2555746	3,1001818	0,001934	0,009178	AIFM3
ENSGALG00000006319	831,26714	0,791303644	0,1737644	4,5538886	5,27E-06	5,80E-05	TGFBI
ENSGALG00000028576	735,47031	0,790565069	0,2313869	3,4166365	0,000634	0,0036529	PAX1
ENSGALG00000010708	2290,4638	0,790532368	0,1502852	5,2602149	1,44E-07	2,24E-06	ICA1
ENSGALG00000021589	1830,7464	0,790216265	0,177386	4,4547837	8,40E-06	8,83E-05	RTN4RL1
ENSGALG00000027458	914,19579	0,790135397	0,1634873	4,8330066	1,34E-06	1,70E-05	
ENSGALG00000014300	310,95646	0,790002156	0,2504565	3,1542493	0,0016091	0,0078863	KLB
ENSGALG00000013862	6295,0756	0,789845326	0,1439094	5,4884886	4,05E-08	7,08E-07	OLIG3
ENSGALG00000026899	678,26051	0,789824953	0,18654	4,2340774	2,29E-05	0,000211	CHSY3
ENSGALG00000020522	2265,9648	0,78974705	0,152638	5,1739888	2,29E-07	3,42E-06	MYRF
ENSGALG00000009039	1670,7449	0,789311851	0,1468245	5,375886	7,62E-08	1,25E-06	NPDC1
ENSGALG00000003933	4489,5312	0,788744436	0,1311022	6,0162586	1,78E-09	4,11E-08	QSOX1
ENSGALG00000013642	1029,7112	0,788560165	0,1619937	4,8678436	1,13E-06	1,46E-05	KAZN
ENSGALG00000006453	2069,6286	0,788464487	0,1533354	5,1420896	2,72E-07	3,98E-06	TF
ENSGALG00000007623	3327,2226	0,788173273	0,1396726	5,6430046	1,67E-08	3,18E-07	CACNA1G
ENSGALG00000024097	498,63238	0,787003033	0,253676	3,102395	0,0019196	0,0091192	AGBL2
ENSGALG00000011913	446,23727	0,78594155	0,2729807	2,8791099	0,003988	0,0166514	sept-03

ENSGALG00000005849	1033,3817	0,784505411	0,1637307	4,7914382	1,66E-06	2,05E-05	PPDPF
ENSGALG00000000076	922,87992	0,7843985	0,1622181	4,8354571	1,33E-06	1,68E-05	NRBP2
ENSGALG00000016507	201,43925	0,784023754	0,2659211	2,9483321	0,0031949	0,0138709	FNDC4
ENSGALG00000012116	675,67694	0,783832372	0,1875924	4,1783795	2,94E-05	0,0002606	EEPD1
ENSGALG00000006508	175,86669	0,783784604	0,2797588	2,8016444	0,0050843	0,0203416	FGF13
ENSGALG00000011635	890,9248	0,783625313	0,1778317	4,4065551	1,05E-05	0,0001078	TFCP2L1
ENSGALG00000000313	165,09493	0,78307364	0,2881217	2,7178574	0,0065706	0,0251007	TNNI1
ENSGALG00000003900	14,168362	0,782866932	0,3173181	2,467136	0,0136199	0,0448342	CACNG1
ENSGALG00000027788	2064,8116	0,782858625	0,1847884	4,2365144	2,27E-05	0,0002093	CPE
ENSGALG00000009866	599,48537	0,782677583	0,2085508	3,7529357	0,0001748	0,0012203	CAMK1D
ENSGALG00000023069	225,10329	0,782582264	0,2592407	3,0187478	0,0025382	0,0114506	MYRIP
ENSGALG00000020376	2337,0409	0,781084057	0,129917	6,0121758	1,83E-09	4,20E-08	
ENSGALG00000024447	255,3388	0,780783451	0,2581275	3,0247981	0,002488	0,0112763	NMB
ENSGALG00000007357	1727,6304	0,780757803	0,1788255	4,3660311	1,27E-05	0,0001264	BCL6
ENSGALG00000021240	149,64373	0,780471598	0,2990547	2,6097954	0,0090596	0,0324549	
ENSGALG00000009407	420,64448	0,780277328	0,2147109	3,6340831	0,000279	0,0018234	
ENSGALG00000021620	400,19342	0,780112352	0,215822	3,6146106	0,0003008	0,0019465	TMEM59L
ENSGALG00000005657	1485,5044	0,780089243	0,2099353	3,7158564	0,0002025	0,0013786	CRHR2
ENSGALG00000017327	228,75567	0,779838018	0,2748161	2,8376719	0,0045444	0,0184913	P2RY6
ENSGALG00000017065	605,99073	0,779643959	0,2212955	3,5230893	0,0004265	0,0026302	STARD13
ENSGALG00000015379	3926,961	0,779239634	0,1720773	4,5284285	5,94E-06	6,41E-05	TAGLN3
ENSGALG00000010898	121,86089	0,778406503	0,3067735	2,5373979	0,011168	0,0383873	BMP3
ENSGALG00000001735	332,00127	0,778269785	0,3162815	2,4606869	0,0138671	0,0454285	LHX4
ENSGALG00000005418	474,48986	0,777790922	0,2764455	2,8135412	0,0048999	0,019755	FRRS1
ENSGALG00000015615	136,43246	0,777196481	0,3023569	2,5704607	0,0101563	0,0355929	DOK7
ENSGALG00000005539	507,50867	0,77704495	0,1983441	3,9176614	8,94E-05	0,0006824	ITM2C
ENSGALG00000009612	1284,262	0,776962988	0,1567277	4,9574072	7,14E-07	9,55E-06	TGFB2
ENSGALG00000005421	527,43362	0,776882668	0,193304	4,0189685	5,85E-05	0,0004709	TEX2L
ENSGALG00000017086	244,06613	0,776712467	0,2590522	2,9982852	0,002715	0,0121396	MTUS2
ENSGALG00000026326	2889,7968	0,77526969	0,1465876	5,2887801	1,23E-07	1,94E-06	WBP1L
ENSGALG00000003747	210,55966	0,773681017	0,268223	2,8844693	0,0039207	0,0164299	OSMR
ENSGALG00000002708	2271,2308	0,772455755	0,1693238	4,5620036	5,07E-06	5,60E-05	LINGO1
ENSGALG00000011986	13,457929	0,772453397	0,3038739	2,5420199	0,0110214	0,0380183	FABP2
ENSGALG00000004183	169,51264	0,772146407	0,2847172	2,7119762	0,0066883	0,0255075	FAM20A
ENSGALG00000013404	465,07043	0,770980594	0,2045333	3,7694617	0,0001636	0,0011517	AK8
ENSGALG00000002283	1172,584	0,770174106	0,1718025	4,482905	7,36E-06	7,80E-05	MAPKAPK3
ENSGALG00000017208	521,56067	0,769802089	0,1959747	3,9280679	8,56E-05	0,0006572	AMOTL1
ENSGALG00000013350	163,60204	0,766573209	0,2847601	2,6919969	0,0071026	0,0267426	MTMR11
ENSGALG00000027592	1174,3515	0,765780883	0,1667631	4,5920291	4,39E-06	4,91E-05	ERN1
ENSGALG00000005779	2301,589	0,765244932	0,1290039	5,9319529	2,99E-09	6,58E-08	FBXO31
ENSGALG00000006900	414,72698	0,764663976	0,2420639	3,158934	0,0015835	0,0077802	ST6GALNAC2L
ENSGALG00000004323	3702,9555	0,7637615	0,1520557	5,0229053	5,09E-07	7,07E-06	RASAL2
ENSGALG00000007744	1224,4032	0,76351713	0,1645392	4,6403371	3,48E-06	3,98E-05	CASS4
ENSGALG00000000897	268,90562	0,763363816	0,249632	3,0579566	0,0022285	0,0103145	LHFPL5
ENSGALG00000004491	190,54596	0,761794314	0,2733192	2,7871964	0,0053166	0,0211161	DMGDH
ENSGALG00000002069	610,16885	0,761037525	0,2096031	3,6308501	0,0002825	0,0018438	FAM214B
ENSGALG00000013948	736,87288	0,760675086	0,1727814	4,402528	1,07E-05	0,0001095	RASL11B
ENSGALG00000006642	162,08607	0,760653303	0,2972229	2,5592016	0,0104913	0,0365597	ADORA2A
ENSGALG00000015115	155,99801	0,760473682	0,2860137	2,6588715	0,0078403	0,028999	MAMDC2
ENSGALG00000002804	155,62632	0,759679339	0,2858837	2,6573024	0,0078769	0,0290949	RND2
ENSGALG00000007956	2266,7266	0,759404394	0,1455052	5,2190893	1,80E-07	2,75E-06	PLXDC2
ENSGALG00000007748	2468,6975	0,758936347	0,126557	5,9967954	2,01E-09	4,56E-08	SLC16A2
ENSGALG00000004170	385,32134	0,75875998	0,2343812	3,23729	0,0012067	0,0062493	ADA
ENSGALG00000015688	1188,5305	0,758515207	0,1730216	4,3839346	1,17E-05	0,0001181	MXD4
ENSGALG00000006576	399,34369	0,758241661	0,2615129	2,8994424	0,0037383	0,015831	GRM3
ENSGALG00000001933	120,41471	0,757286355	0,3102287	2,4410587	0,0146443	0,0474719	CORO2A
ENSGALG00000003115	505,20482	0,756684773	0,2022143	3,7419946	0,0001826	0,0012662	COL4A3
ENSGALG00000007889	769,26198	0,756666335	0,1846622	4,0975693	4,18E-05	0,0003529	CCDC85A
ENSGALG00000006346	1339,3203	0,756405715	0,1832878	4,1268742	3,68E-05	0,0003163	CXCL14
ENSGALG00000026153	524,42092	0,755983234	0,2314691	3,2660225	0,0010907	0,0057424	FOXO6
ENSGALG00000026091	117,64664	0,755485898	0,3112696	2,4271108	0,0152196	0,0489092	
ENSGALG00000002958	2112,9504	0,755313619	0,167448	4,510735	6,46E-06	6,90E-05	DNER

ENSGALG00000001311	1301,9009	0,752137925	0,1518518	4,9531061	7,30E-07	9,72E-06	ARHGAP44
ENSGALG00000027905	3422,0143	0,751625166	0,132987	5,6518692	1,59E-08	3,03E-07	CSRNP1
ENSGALG00000009439	631,53261	0,751125582	0,2024985	3,7092895	0,0002078	0,0014128	GPR85
ENSGALG00000006795	336,85781	0,75042189	0,2480756	3,0249723	0,0024866	0,0112763	ABCG4
ENSGALG00000029047	339,90745	0,749178885	0,226666	3,30521	0,0009491	0,0051255	TMEM204
ENSGALG00000015729	1439,0922	0,747405326	0,14976	4,9906871	6,02E-07	8,18E-06	LPAR1
ENSGALG00000019030	11,696679	0,747320322	0,2902826	2,5744581	0,0100397	0,035266	
ENSGALG00000006203	182,30527	0,747208785	0,2854721	2,6174493	0,008859	0,0319171	
ENSGALG00000001969	1252,395	0,746864271	0,1550045	4,8183402	1,45E-06	1,82E-05	LPPR3
ENSGALG00000006050	280,4499	0,74642112	0,2472463	3,0189372	0,0025366	0,0114472	CYP2R1
ENSGALG00000004420	2554,1446	0,745430944	0,1535099	4,8559134	1,20E-06	1,54E-05	SDK1
ENSGALG00000018733	341,10493	0,744919582	0,2235871	3,3316754	0,0008632	0,0047277	
ENSGALG00000023828	228,11537	0,744788155	0,2585381	2,8807671	0,0039671	0,0165833	LDHD
ENSGALG00000007849	195,07279	0,744715772	0,2780479	2,6783724	0,0073981	0,0276565	CD44
ENSGALG00000005063	630,43033	0,74466275	0,1956389	3,8063128	0,0001411	0,0010113	ST6GALNAC4
ENSGALG00000017050	201,15206	0,743800542	0,2755212	2,6996126	0,006942	0,0262325	SMAD9
ENSGALG00000026919	497,57567	0,743491927	0,2001741	3,7142261	0,0002038	0,0013869	
ENSGALG00000025937	424,74226	0,743354667	0,2192559	3,3903524	0,000698	0,0039714	FXYD6
ENSGALG00000012712	2097,8069	0,742584862	0,1498005	4,9571602	7,15E-07	9,56E-06	RBM24
ENSGALG00000015994	1103,9909	0,741778145	0,1550114	4,7853126	1,71E-06	2,10E-05	MAVS
ENSGALG00000016622	136,85244	0,741316835	0,3044477	2,4349564	0,0148936	0,0481221	STS
ENSGALG00000001449	3789,5374	0,740070536	0,1451567	5,0984252	3,42E-07	4,93E-06	STRA6
ENSGALG00000006467	165,38559	0,739825587	0,2835935	2,6087534	0,0090873	0,0325074	XKR7
ENSGALG00000003022	149,11768	0,738879702	0,2881931	2,5638358	0,0103522	0,0361678	
ENSGALG00000026449	3878,5798	0,737442369	0,1336353	5,5183189	3,42E-08	6,04E-07	
ENSGALG00000011524	227,70396	0,737317525	0,2799	2,6342174	0,0084331	0,0306998	PPEF2
ENSGALG00000002539	465,9328	0,737264559	0,2112723	3,4896412	0,0004837	0,002919	KCNN2
ENSGALG00000022001	220,24358	0,737013016	0,2592948	2,8423753	0,0044779	0,0182643	CAMKV
ENSGALG00000016749	204,45908	0,736312128	0,2623793	2,8062894	0,0050116	0,0200861	CNGA3
ENSGALG00000008339	615,63573	0,736061451	0,1948925	3,776756	0,0001589	0,0011208	GRM7
ENSGALG00000013002	3217,4534	0,734684533	0,1469698	4,9988824	5,77E-07	7,89E-06	DAP
ENSGALG00000019087	2799,7159	0,734240753	0,1295389	5,6681115	1,44E-08	2,78E-07	RFXAP
ENSGALG00000011059	526,88294	0,733913318	0,20212	3,6310777	0,0002822	0,001843	HOX1B
ENSGALG00000019291	131,18453	0,733807509	0,2877581	2,5500843	0,0107697	0,0373103	
ENSGALG00000010326	286,24182	0,733495449	0,2372268	3,0919588	0,0019884	0,0093882	FLVCR2
ENSGALG00000008433	2910,7777	0,732487	0,1303149	5,6209017	1,90E-08	3,57E-07	FZD2
ENSGALG00000008076	5047,2961	0,731953468	0,1274268	5,7441112	9,24E-09	1,83E-07	TMEM164
ENSGALG00000006897	1904,4846	0,73176707	0,1370838	5,3381014	9,39E-08	1,52E-06	
ENSGALG00000025916	1511,635	0,730226914	0,1971166	3,7045423	0,0002118	0,0014359	EMILIN3
ENSGALG00000002651	2712,1143	0,730025866	0,128861	5,6652212	1,47E-08	2,82E-07	ST3GAL2
ENSGALG00000012191	7671,5827	0,72968259	0,1256369	5,8078663	6,33E-09	1,30E-07	LGR4
ENSGALG00000002451	1225,1572	0,728956851	0,1533766	4,7527269	2,01E-06	2,41E-05	KCNT2
ENSGALG00000019317	6925,165	0,728083244	0,1200043	6,0671438	1,30E-09	3,05E-08	JOSD1
ENSGALG00000001561	154,80244	0,72739532	0,2918515	2,4923472	0,0126902	0,0424526	MXRA8
ENSGALG00000013925	194,37903	0,727322252	0,27832	2,6132593	0,0089683	0,0321919	KIT
ENSGALG00000000400	352,09264	0,727285624	0,2194005	3,3148775	0,0009168	0,0049811	KCNC4
ENSGALG00000013143	266,7402	0,72680287	0,2471882	2,9402819	0,0032791	0,0141417	PDE3A
ENSGALG00000014910	3784,5308	0,72588259	0,1623113	4,4721633	7,74E-06	8,18E-05	COLEC12
ENSGALG00000023083	215,10004	0,725643274	0,2997631	2,4207225	0,0154897	0,0496386	KIAA1958
ENSGALG00000004875	3335,2739	0,724796191	0,1381226	5,2474846	1,54E-07	2,39E-06	PEMT
ENSGALG00000015123	798,45006	0,722653298	0,1722336	4,1957734	2,72E-05	0,0002436	ZDHHC23
ENSGALG00000026864	531,42097	0,721498621	0,203036	3,5535495	0,0003801	0,0023748	ZCCHC24
ENSGALG00000011426	2063,2289	0,721230387	0,1613379	4,4703085	7,81E-06	8,24E-05	RP11-514O12.4
ENSGALG00000000723	8396,3268	0,72079059	0,1405098	5,1298237	2,90E-07	4,21E-06	STX12
ENSGALG00000008914	183,84781	0,720697485	0,2822547	2,5533588	0,010669	0,0370556	NRAP
ENSGALG00000002558	389,45186	0,720642154	0,2290837	3,1457591	0,0016566	0,0080666	ASL1
ENSGALG00000001459	1037,3333	0,719793089	0,1787902	4,0259094	5,68E-05	0,0004594	TNNC1
ENSGALG00000007981	2067,6116	0,719546729	0,1428586	5,0367766	4,73E-07	6,61E-06	CLN6
ENSGALG00000009042	1726,6169	0,719468223	0,1493757	4,8165026	1,46E-06	1,83E-05	ISM1
ENSGALG00000017296	332,74685	0,719276435	0,2726134	2,6384485	0,0083286	0,0303762	KLHL42
ENSGALG00000003855	2094,2466	0,719100031	0,1368455	5,2548333	1,48E-07	2,31E-06	SRC
ENSGALG00000017031	283,53237	0,718524914	0,2888562	2,4874835	0,012865	0,0429181	TPTE2

ENSGALG00000015403	10307,491	0,718054416	0,1317872	5,4485916	5,08E-08	8,65E-07	EPHA3
ENSGALG00000007383	515,08856	0,717575013	0,2017592	3,5565912	0,0003757	0,0023529	HSPB8
ENSGALG00000012792	5661,7981	0,717520705	0,1251036	5,7354139	9,73E-09	1,92E-07	HIPK2
ENSGALG00000013583	619,65104	0,717484093	0,2169479	3,3071723	0,0009424	0,0050958	FAM114A1
ENSGALG00000024012	315,05149	0,716701131	0,2440127	2,937147	0,0033125	0,0142674	TDRKH
ENSGALG00000023607	192,83095	0,714467677	0,2785112	2,5653106	0,0103083	0,0360237	ASCL2
ENSGALG00000004812	420,51393	0,714148656	0,2276151	3,1375277	0,0017038	0,0082611	FAM129A
ENSGALG00000003340	440,9962	0,714011033	0,2132487	3,3482552	0,0008132	0,0044953	GBGT1
ENSGALG00000001233	1022,3151	0,713516886	0,1751304	4,0742031	4,62E-05	0,0003833	RHCE
ENSGALG00000026230	11,282047	0,713226116	0,2861696	2,49232	0,0126912	0,0424526	
ENSGALG00000007244	345,61048	0,711922899	0,2206692	3,2261997	0,0012545	0,0064477	SIRT4
ENSGALG00000009628	330,24566	0,711918795	0,2307632	3,085062	0,0020351	0,0095809	
ENSGALG00000003282	5419,4179	0,711820753	0,1111661	6,4032191	1,52E-10	4,05E-09	STAT5B
ENSGALG00000007196	4102,6967	0,710478048	0,1280928	5,5465879	2,91E-08	5,23E-07	STXBP1
ENSGALG00000003805	6589,8039	0,710406685	0,1297333	5,4759004	4,35E-08	7,53E-07	PRKG1
ENSGALG00000001130	1241,2514	0,709264893	0,1624051	4,3672563	1,26E-05	0,0001259	WBSCR17
ENSGALG00000020794	10,814613	0,707249449	0,285562	2,4766932	0,0132606	0,0439509	
ENSGALG00000002038	769,11793	0,706334316	0,1755894	4,0226475	5,75E-05	0,000465	DENND2C
ENSGALG00000007618	10,281175	0,705775117	0,2855705	2,4714563	0,0134564	0,0444413	BTG4
ENSGALG00000028443	5716,4355	0,705249503	0,169055	4,1717175	3,02E-05	0,0002673	CSPG5
ENSGALG00000023738	705,32888	0,704601132	0,2001565	3,5202511	0,0004311	0,0026513	
ENSGALG00000010320	1626,0227	0,704383823	0,138468	5,0869789	3,64E-07	5,19E-06	PAWR
ENSGALG00000028685	205,34816	0,704382223	0,2842057	2,4784238	0,0131964	0,0437898	
ENSGALG00000001640	312,33982	0,704326284	0,2358711	2,9860645	0,0028259	0,0125489	RNF122
ENSGALG00000000599	300,39451	0,703788264	0,2449165	2,8735847	0,0040584	0,0168977	mars-02
ENSGALG00000026437	5573,4884	0,703536871	0,1156883	6,0813142	1,19E-09	2,80E-08	
ENSGALG00000004735	2180,5981	0,703327732	0,1488288	4,7257505	2,29E-06	2,73E-05	MAP2K3
ENSGALG00000026973	1340,8175	0,702510997	0,1463553	4,8000384	1,59E-06	1,97E-05	KIF3C
ENSGALG00000026873	606,99857	0,701267796	0,1971895	3,5563142	0,0003761	0,0023543	RSPO2
ENSGALG00000017269	189,52991	0,70061916	0,2816451	2,4875958	0,012861	0,0429172	USP35
ENSGALG00000023266	1017,6993	0,69953934	0,177551	3,9399339	8,15E-05	0,0006287	INPP5J
ENSGALG00000027058	1118,3528	0,699526976	0,185719	3,7665882	0,0001655	0,001164	EMP2
ENSGALG00000028018	221,75017	0,69904366	0,2851436	2,4515496	0,0142243	0,0463753	RAB38
ENSGALG00000020497	375,96471	0,698148301	0,21726	3,2134229	0,0013116	0,0066923	IFITM10
ENSGALG00000008902	238,20892	0,6980432	0,2801236	2,4919118	0,0127058	0,0424825	GIPC2
ENSGALG00000004647	2581,3028	0,697164089	0,1609537	4,3314572	1,48E-05	0,0001453	C5H15ORF57
ENSGALG00000015181	1358,5983	0,696928823	0,1980876	3,518286	0,0004343	0,0026634	GAREM1
ENSGALG00000016299	188,53968	0,696461514	0,2699353	2,5801055	0,009877	0,0348087	FAM83B
ENSGALG00000028945	1325,8832	0,696399862	0,1972938	3,5297611	0,0004159	0,0025729	LGI2
ENSGALG00000007349	388,2147	0,696333265	0,2142206	3,2505423	0,0011519	0,0060109	RASL12
ENSGALG00000008455	4612,7445	0,695894902	0,1608753	4,3256789	1,52E-05	0,0001481	SEMA3C
ENSGALG00000010094	8338,3892	0,695566561	0,1102371	6,3097346	2,80E-10	7,11E-09	B4GALT2
ENSGALG00000008250	259,75115	0,695347261	0,2674448	2,5999651	0,0093233	0,0332035	TBX3
ENSGALG00000010331	1143,0104	0,693784562	0,1997471	3,4733156	0,0005141	0,0030671	MME
ENSGALG00000010406	684,71072	0,693540735	0,1775952	3,9051762	9,42E-05	0,0007122	TMEM63C
ENSGALG00000001889	934,57066	0,693259058	0,1726852	4,0145828	5,96E-05	0,0004785	CARD9
ENSGALG00000008651	2483,2004	0,693028969	0,1577851	4,3922326	1,12E-05	0,0001142	APMAP
ENSGALG00000005103	1726,7826	0,692645693	0,1383584	5,0061716	5,55E-07	7,63E-06	GDPD1
ENSGALG00000023188	975,83818	0,692540652	0,1600052	4,3282373	1,50E-05	0,0001471	NANP
ENSGALG00000001697	10,047458	0,69224725	0,2838775	2,4385418	0,0147467	0,0477354	ITIH3
ENSGALG00000002192	251,47548	0,692054061	0,2782223	2,4874138	0,0128676	0,0429181	PTPRC
ENSGALG00000003636	432,59045	0,690941222	0,2090885	3,3045388	0,0009513	0,0051317	FBLIM1
ENSGALG00000020575	447,18026	0,690014438	0,2210762	3,1211618	0,0018014	0,0086359	ERMN
ENSGALG00000023655	726,63007	0,689103212	0,1781324	3,8684889	0,0001095	0,0008103	
ENSGALG00000019436	255,33631	0,68869647	0,2721433	2,5306391	0,0113855	0,0389481	MAL2
ENSGALG00000011356	243,58893	0,688404159	0,2584311	2,6637818	0,0077268	0,0286811	
ENSGALG00000003122	2359,0002	0,68839827	0,1635612	4,2088128	2,57E-05	0,0002327	ZFAND2A
ENSGALG00000026361	265,71557	0,687466561	0,2597715	2,6464277	0,0081347	0,0297885	SLITRK5
ENSGALG00000002749	1561,2531	0,687223145	0,1398964	4,9123728	9,00E-07	1,18E-05	RP11-565P22.6
ENSGALG00000001342	285,4866	0,686942007	0,2446203	2,8081975	0,004982	0,0200146	HCN2
ENSGALG00000016844	1336,9691	0,685906484	0,1561619	4,3922785	1,12E-05	0,0001142	RAB20
ENSGALG00000016123	816,29325	0,685692574	0,2096562	3,2705578	0,0010734	0,0056686	TRPS1

ENSGALG00000002204	670,70196	0,684953864	0,196026	3,4941984	0,0004755	0,0028759	SLC7A4
ENSGALG00000009444	276,54744	0,684544481	0,2525059	2,7110044	0,006708	0,0255466	HHIPL2
ENSGALG00000014559	1808,9059	0,684328525	0,1507966	4,5380898	5,68E-06	6,17E-05	MEF2D
ENSGALG00000016570	213,7613	0,684262758	0,2599004	2,6327884	0,0084687	0,0307881	
ENSGALG00000009075	1175,3212	0,683878573	0,1540118	4,44043	8,98E-06	9,38E-05	CD99L2
ENSGALG00000010770	1614,3878	0,683740122	0,1643769	4,159587	3,19E-05	0,0002801	SCIN
ENSGALG00000026269	172,06551	0,683345796	0,2809077	2,432635	0,0149894	0,0483604	TAP1
ENSGALG00000009523	834,57418	0,681144754	0,1860744	3,6606041	0,0002516	0,0016663	CNTN1
ENSGALG00000007251	3608,0883	0,681060329	0,121444	5,6080193	2,05E-08	3,82E-07	PCYT2
ENSGALG00000004341	261,34286	0,680949124	0,262149	2,5975655	0,0093887	0,0333927	
ENSGALG00000019702	11617,301	0,678512546	0,132661	5,1146335	3,14E-07	4,54E-06	RP4-613B23.5
ENSGALG00000000674	418,54848	0,67836471	0,2130129	3,1846173	0,0014495	0,00725	MYO1D
ENSGALG00000020688	589,72193	0,678030575	0,2132646	3,1792929	0,0014763	0,0073359	CYP4B1L
ENSGALG00000026986	1312,7721	0,67793682	0,1686465	4,0198699	5,82E-05	0,0004694	KIAA1324
ENSGALG00000004240	977,7105	0,676367039	0,1738198	3,8911964	9,98E-05	0,0007483	ORAI1
ENSGALG00000025764	5164,9467	0,675250138	0,1225181	5,5114325	3,56E-08	6,27E-07	FKBP10
ENSGALG00000002028	1196,673	0,675138088	0,1985373	3,4005596	0,0006725	0,0038438	GPRC5B
ENSGALG00000015425	550,05237	0,674783987	0,2346154	2,8761286	0,0040259	0,0167724	LPL
ENSGALG00000012583	448,70952	0,674342421	0,2079713	3,242479	0,0011849	0,0061506	IDNK
ENSGALG00000016471	262,57308	0,674081782	0,2520085	2,6748379	0,0074765	0,0278962	NT5C1B
ENSGALG00000021345	271,10964	0,673475888	0,2582429	2,6079164	0,0091095	0,0325699	B3GNT9
ENSGALG00000016399	1323,2505	0,673456474	0,1609458	4,1843685	2,86E-05	0,000255	SNTB1
ENSGALG00000004472	11283,073	0,672299012	0,1222317	5,5002025	3,79E-08	6,65E-07	ASTN1
ENSGALG00000005041	1267,2443	0,67175632	0,1650486	4,0700516	4,70E-05	0,0003893	TMEM35
ENSGALG00000011896	1990,3879	0,671754963	0,1334515	5,0337007	4,81E-07	6,71E-06	SIDT2
ENSGALG00000007038	2302,528	0,670533206	0,1352237	4,9586965	7,10E-07	9,50E-06	ASTN2
ENSGALG00000005069	1045,9472	0,669583836	0,1580685	4,2360364	2,27E-05	0,0002096	PTGS2
ENSGALG00000006700	425,17548	0,669478691	0,2185126	3,0637986	0,0021855	0,0101553	PROSER2
ENSGALG00000000040	692,57517	0,669045681	0,1867751	3,5820926	0,0003409	0,0021706	FAM195A
ENSGALG00000000920	727,43745	0,668851002	0,1978996	3,3797499	0,0007255	0,0041021	CGN
ENSGALG00000016415	1383,1065	0,668618298	0,1866966	3,581309	0,0003419	0,0021744	MAP7D2
ENSGALG00000002551	236,59495	0,667886193	0,2539141	2,6303623	0,0085294	0,0309591	TTC18
ENSGALG00000016617	4902,5535	0,667497538	0,1287787	5,1832934	2,18E-07	3,27E-06	NCOA1
ENSGALG00000008970	735,02263	0,666872911	0,1806417	3,6916889	0,0002228	0,0015	NRK
ENSGALG00000006449	910,14644	0,666661271	0,1961576	3,3985997	0,0006773	0,0038698	GRIP2
ENSGALG00000003736	312,42816	0,665332911	0,230427	2,8873912	0,0038845	0,0163183	ABCB9
ENSGALG00000007324	392,14542	0,66463294	0,2296182	2,8945136	0,0037975	0,0160169	TP63
ENSGALG00000003457	3064,2612	0,662964808	0,1431321	4,6318371	3,62E-06	4,14E-05	CNP
ENSGALG00000013766	661,355	0,662853472	0,1976254	3,35409	0,0007963	0,0044322	DSEL
ENSGALG00000008135	345,6592	0,662771473	0,2243246	2,9545197	0,0031316	0,0136305	SATB2
ENSGALG00000010316	7099,3915	0,662660688	0,1194525	5,5474833	2,90E-08	5,21E-07	FRAS1
ENSGALG00000016800	213,64071	0,662047446	0,2628994	2,5182536	0,0117938	0,0400432	C2orf40
ENSGALG00000006775	290,55417	0,661623797	0,267005	2,4779452	0,0132141	0,0438272	TGM2
ENSGALG00000002775	509,11695	0,661278251	0,2056466	3,2156052	0,0013017	0,0066526	FA2H
ENSGALG00000012710	1279,2972	0,660750331	0,1586684	4,1643484	3,12E-05	0,000275	NCS1
ENSGALG00000023708	346,0463	0,660417389	0,226761	2,9123934	0,0035867	0,0152699	UBXN10
ENSGALG00000018815	410,19043	0,659828915	0,2173041	3,0364305	0,002394	0,0109266	PCGF2
ENSGALG00000006236	481,20913	0,65916962	0,2166125	3,0430824	0,0023417	0,0107419	TPH1
ENSGALG00000016411	1960,7449	0,657856223	0,1571317	4,1866556	2,83E-05	0,0002527	COL14A1
ENSGALG00000006152	1208,2078	0,657637784	0,1593989	4,125736	3,70E-05	0,0003172	
ENSGALG00000025698	1218,1933	0,657354664	0,227163	2,8937581	0,0038066	0,0160455	
ENSGALG00000018967	1911,6852	0,656123394	0,1849185	3,5481766	0,0003879	0,0024149	LYN
ENSGALG00000016543	3236,9117	0,654951402	0,1701332	3,8496396	0,0001183	0,000864	NHS
ENSGALG00000010732	284,29764	0,654838117	0,2441236	2,6824038	0,0073095	0,027378	GALNTL6
ENSGALG00000009473	257,7395	0,654742804	0,2591149	2,5268436	0,0115093	0,0392335	EFCAB1
ENSGALG00000012092	321,27125	0,654654529	0,2674895	2,4474025	0,014389	0,046778	ESYT3
ENSGALG00000012023	986,86948	0,654468757	0,1614892	4,0527095	5,06E-05	0,000416	DACT1
ENSGALG00000003845	2106,1901	0,653837192	0,1371393	4,7676855	1,86E-06	2,26E-05	MKNK2
ENSGALG00000006676	521,68608	0,652541968	0,2289326	2,8503676	0,0043669	0,0179081	RLBP1
ENSGALG00000003589	5654,7984	0,652278144	0,1207315	5,4027172	6,56E-08	1,10E-06	VTN
ENSGALG00000001426	1341,2118	0,651807691	0,1484727	4,3900856	1,13E-05	0,0001152	MECR
ENSGALG00000002403	2862,6989	0,651643976	0,1530067	4,2589243	2,05E-05	0,0001919	ULK1

ENSGALG00000001956	2538,005	0,650817237	0,1761305	3,6950845	0,0002198	0,001483	OLFML3
ENSGALG00000014225	320,24798	0,65009587	0,2560628	2,5388138	0,0111229	0,0382613	KCTD8
ENSGALG00000008120	6095,8052	0,649872522	0,1262195	5,1487478	2,62E-07	3,86E-06	TLE3
ENSGALG00000024104	1479,6002	0,64969951	0,1504929	4,3171453	1,58E-05	0,0001528	
ENSGALG00000005145	354,77662	0,64926713	0,2184796	2,9717518	0,0029611	0,0130342	SS18L1
ENSGALG00000024488	3112,8373	0,649250333	0,1855727	3,4986306	0,0004677	0,0028349	CD99
ENSGALG00000008763	548,60866	0,64905793	0,2030908	3,1959002	0,001394	0,0070213	SSX2IP
ENSGALG00000007090	354,84899	0,648812032	0,2646101	2,4519552	0,0142082	0,0463564	CPN1
ENSGALG00000008780	291,63944	0,648139707	0,2519098	2,5729041	0,0100849	0,0354064	CTBS
ENSGALG00000007848	1247,6356	0,647083546	0,1839624	3,5174772	0,0004357	0,0026671	PTS
ENSGALG00000010422	5513,2698	0,646628908	0,1246783	5,1863789	2,14E-07	3,22E-06	FEZ1
ENSGALG00000010719	1138,2128	0,646215963	0,1598376	4,0429522	5,28E-05	0,0004319	NEIL3
ENSGALG00000006036	6926,7297	0,644947298	0,1418361	4,547131	5,44E-06	5,94E-05	
ENSGALG00000003655	1118,2527	0,643488267	0,1848793	3,4805866	0,0005003	0,0030021	GAS2
ENSGALG00000000402	743,97016	0,642116084	0,180432	3,5587699	0,0003726	0,0023388	LOXL2
ENSGALG00000006947	23413,053	0,64187784	0,1108573	5,7901257	7,03E-09	1,43E-07	BDH1
ENSGALG00000003568	441,24282	0,640936632	0,2094653	3,0598708	0,0022143	0,0102684	PPP1R16B
ENSGALG00000011335	567,87108	0,64086738	0,205863	3,113077	0,0018515	0,0088263	NHEJ1
ENSGALG00000008479	572,78152	0,640276298	0,2217437	2,8874605	0,0038837	0,0163183	PLEKHM3
ENSGALG00000026159	2624,117	0,639768169	0,1366904	4,6804192	2,86E-06	3,34E-05	PDLIM7
ENSGALG00000004074	504,98036	0,63919781	0,2089342	3,0593254	0,0022184	0,0102792	KCNS1
ENSGALG00000026295	3751,1713	0,638993968	0,1256122	5,0870391	3,64E-07	5,19E-06	CDR2L
ENSGALG00000000935	1038,8243	0,638135684	0,1951826	3,2694289	0,0010776	0,0056891	TUFT1
ENSGALG00000009478	877,18337	0,637933371	0,2202594	2,8962816	0,0037761	0,0159566	BCHE
ENSGALG00000028506	1185,606	0,637581477	0,166917	3,8197523	0,0001336	0,0009649	
ENSGALG00000003527	1639,1106	0,636215476	0,1470878	4,3254142	1,52E-05	0,0001482	ARRDC2
ENSGALG00000028756	1081,2256	0,636133999	0,1663302	3,8245244	0,000131	0,0009474	SLC4A2
ENSGALG00000004585	1609,3421	0,634633954	0,1652199	3,841147	0,0001225	0,0008907	SLC29A3
ENSGALG00000020726	246,91021	0,63266925	0,2559148	2,4721867	0,0134289	0,0443774	PDZD7
ENSGALG00000008057	2849,9643	0,63255389	0,1469667	4,3040624	1,68E-05	0,0001609	LLGL2
ENSGALG00000011053	10065,657	0,630484727	0,107695	5,8543543	4,79E-09	1,01E-07	HOXA2
ENSGALG00000008151	5862,8164	0,629127593	0,1186504	5,3023621	1,14E-07	1,81E-06	WSB2
ENSGALG00000027658	594,25398	0,628365542	0,1906089	3,2966228	0,0009785	0,005237	TESK1
ENSGALG00000002664	1165,5467	0,627944771	0,1531652	4,0997864	4,14E-05	0,0003505	ANXA11
ENSGALG00000029000	1142,4251	0,627547484	0,158422	3,9612397	7,46E-05	0,0005808	HYAL3
ENSGALG00000028204	8948,3531	0,626531339	0,1218206	5,1430676	2,70E-07	3,97E-06	GPX1
ENSGALG00000001100	796,40837	0,626475621	0,1802096	3,4763714	0,0005082	0,0030393	ST3GAL4
ENSGALG00000002313	2718,417	0,626374122	0,1346423	4,6521335	3,29E-06	3,79E-05	SLC38A7
ENSGALG00000016745	455,542	0,625941712	0,2478958	2,5250189	0,0115692	0,0393984	GABRB3
ENSGALG00000026762	1809,4015	0,625670937	0,1367941	4,5738152	4,79E-06	5,34E-05	WBSCR27
ENSGALG00000008728	894,54762	0,625207283	0,1707065	3,6624697	0,0002498	0,0016558	PTER
ENSGALG00000011856	1033,6518	0,624422163	0,1575632	3,9629959	7,40E-05	0,0005778	C7orf55
ENSGALG00000007267	4451,8628	0,623851268	0,1170233	5,3309986	9,77E-08	1,57E-06	MVD
ENSGALG00000014890	1413,3331	0,620763059	0,1462687	4,2439922	2,20E-05	0,0002031	TNFRSF1A
ENSGALG00000000426	554,75724	0,620684473	0,1991349	3,1169053	0,0018276	0,0087369	IGSF21
ENSGALG00000006114	909,8822	0,61972927	0,1710742	3,6225768	0,0002917	0,0018965	
ENSGALG00000016595	534,98984	0,618865991	0,2042	3,0306858	0,00244	0,0111031	TRIM35
ENSGALG00000011900	1365,0771	0,61861917	0,1798125	3,440356	0,0005809	0,0033976	NAGA
ENSGALG00000007076	2564,3514	0,617550884	0,1437626	4,2956288	1,74E-05	0,0001663	CCNY
ENSGALG00000016934	244,09661	0,616949085	0,2531836	2,4367659	0,0148193	0,0479038	PCDH20
ENSGALG00000014561	290,72868	0,616417234	0,2372422	2,5982612	0,0093697	0,0333426	PTPN6
ENSGALG00000007647	329,01065	0,615818159	0,2520071	2,443654	0,0145394	0,0471767	MYO3A
ENSGALG00000007381	1220,4425	0,615500074	0,1553201	3,9627846	7,41E-05	0,000578	SERPING1
ENSGALG00000028543	1301,4945	0,614319385	0,1647996	3,7276752	0,0001933	0,0013256	FGFR4
ENSGALG00000015734	903,95178	0,61429478	0,1983985	3,0962669	0,0019597	0,0092839	ZC2HC1A
ENSGALG00000016728	5650,4017	0,613362339	0,115156	5,3263591	1,00E-07	1,61E-06	RP2
ENSGALG00000023881	330,14663	0,612305207	0,2457805	2,4912681	0,0127288	0,0425491	PLXDC1
ENSGALG00000016092	954,71804	0,611645343	0,1643983	3,720508	0,0001988	0,0013569	RAB11FIP5
ENSGALG00000011975	2830,8254	0,61116717	0,1227563	4,9787019	6,40E-07	8,65E-06	FBXL2
ENSGALG00000014699	806,27033	0,610005378	0,2334147	2,6133976	0,0089647	0,0321873	MEGF10
ENSGALG00000012495	822,21166	0,608707981	0,2014447	3,0217131	0,0025135	0,0113541	Q9DEH4_CHICK
ENSGALG00000016143	5447,091	0,607480677	0,1713253	3,5457739	0,0003915	0,0024359	RHPN1

ENSGALG00000004103	351,09074	0,607102605	0,2488829	2,4393098	0,0147153	0,0476455	FAM81A
ENSGALG00000008512	665,49717	0,606896507	0,1931945	3,141375	0,0016816	0,0081679	GRIA3
ENSGALG00000008272	1496,8242	0,6063144	0,1570984	3,8594554	0,0001136	0,0008363	TMED3
ENSGALG00000012934	686,11794	0,606004522	0,2092753	2,8957294	0,0037828	0,0159748	BICD1
ENSGALG00000016071	698,48269	0,605474788	0,1980237	3,0575882	0,0022313	0,0103145	CTHRC1
ENSGALG00000015414	1285,1197	0,605396806	0,1804212	3,3554643	0,0007923	0,0044138	NSUN3
ENSGALG00000002930	5292,4924	0,603605896	0,1376656	4,3845818	1,16E-05	0,0001178	STX7
ENSGALG00000005303	729,47439	0,603106981	0,1827363	3,3004217	0,0009654	0,005187	CCDC13
ENSGALG00000006742	8579,7495	0,60288418	0,1075096	5,6077227	2,05E-08	3,82E-07	NECTIN1
ENSGALG00000013964	1158,3447	0,602864404	0,163189	3,6942711	0,0002205	0,0014863	SPATA18
ENSGALG00000015075	452,11233	0,602809512	0,2158206	2,7931043	0,0052205	0,0207767	
ENSGALG00000000608	482,47931	0,60269407	0,1990068	3,0285101	0,0024576	0,0111684	CDH1
ENSGALG00000000739	566,84692	0,602546305	0,188602	3,1948037	0,0013993	0,0070455	XKR8
ENSGALG00000009047	3574,1219	0,601534497	0,1392573	4,3195906	1,56E-05	0,0001517	YPEL5
ENSGALG00000005628	1589,6542	0,601333554	0,1810639	3,3211128	0,0008966	0,0048849	COL9A3
ENSGALG00000029021	4810,2675	0,600451025	0,1243158	4,830046	1,37E-06	1,72E-05	CACNA2D2
ENSGALG00000023435	807,96294	0,600270173	0,1899681	3,1598469	0,0015785	0,0077671	GATM
ENSGALG00000028023	391,94362	0,598578703	0,2158662	2,7729159	0,0055556	0,0219007	C14ORF166B
ENSGALG00000023876	5804,1712	0,598475769	0,1258285	4,7562796	1,97E-06	2,38E-05	PRKCQ
ENSGALG00000016330	507,71021	0,598164421	0,2008484	2,9781891	0,0028996	0,0128091	ANGPT2
ENSGALG00000008475	1732,9827	0,598095279	0,145561	4,1088979	3,98E-05	0,0003394	ARRDC1
ENSGALG00000013167	14635,204	0,597899753	0,1179107	5,0707829	3,96E-07	5,61E-06	SDHA
ENSGALG00000007801	1966,8829	0,597201094	0,1460776	4,0882463	4,35E-05	0,0003633	CORO7
ENSGALG00000027450	1958,1012	0,596865465	0,1360214	4,3880272	1,14E-05	0,000116	
ENSGALG00000005839	281,11607	0,595553706	0,2359627	2,5239319	0,011605	0,0394908	ARID3C
ENSGALG00000002818	12498,951	0,595523293	0,1252741	4,7537617	2,00E-06	2,41E-05	VAT1
ENSGALG00000015399	371,45534	0,595444245	0,2198201	2,7087794	0,0067531	0,0256826	FUT10
ENSGALG00000008285	2205,3966	0,594659875	0,1335709	4,4520161	8,51E-06	8,94E-05	NAPEPLD
ENSGALG00000026756	608,70147	0,593058479	0,1859613	3,1891498	0,0014269	0,0071635	
ENSGALG00000022661	267,36767	0,593055191	0,2440949	2,429609	0,0151151	0,0486392	
ENSGALG00000011145	3513,1587	0,5922185	0,1195159	4,9551454	7,23E-07	9,63E-06	TRIL
ENSGALG00000026180	4122,3044	0,591162305	0,1425421	4,1472816	3,36E-05	0,0002933	MSX2
ENSGALG00000005999	5083,9087	0,591144605	0,1218787	4,8502694	1,23E-06	1,57E-05	ABTB1
ENSGALG00000004596	540,83556	0,59090413	0,1964513	3,0078909	0,0026307	0,0118051	AR
ENSGALG00000013436	9895,6737	0,590008237	0,1280495	4,6076575	4,07E-06	4,58E-05	TET3
ENSGALG00000013742	6102,599	0,589905166	0,1472857	4,0051753	6,20E-05	0,0004943	EZR
ENSGALG00000025745	2020,2184	0,589609597	0,1655708	3,5610734	0,0003693	0,0023216	ARHGAP27
ENSGALG00000026081	3861,3662	0,588777417	0,1223416	4,8125694	1,49E-06	1,86E-05	RBM38
ENSGALG00000013882	324,83067	0,587886739	0,2342948	2,5091749	0,0121014	0,0408428	MXD1
ENSGALG00000000464	6973,1204	0,587838297	0,1100043	5,3437778	9,10E-08	1,48E-06	RHOBTB2
ENSGALG00000004900	814,58968	0,587812156	0,240363	2,4455181	0,0144644	0,0469671	FAM101B
ENSGALG00000005102	2254,4611	0,58732124	0,1676607	3,503035	0,00046	0,0027934	CDH4
ENSGALG00000016594	1080,5684	0,58674351	0,1703086	3,4451783	0,0005707	0,0033462	FRMPD4
ENSGALG00000010934	813,60176	0,586239182	0,1706421	3,4354907	0,0005915	0,0034503	INADL
ENSGALG00000025822	1186,6239	0,586217768	0,1900116	3,0851675	0,0020344	0,0095808	CYP1B1
ENSGALG00000017558	835,37967	0,585776262	0,1728652	3,3886308	0,0007024	0,0039883	
ENSGALG00000000407	552,43307	0,58498369	0,2033571	2,876632	0,0040194	0,0167507	
ENSGALG00000002169	5307,0492	0,58477373	0,1393007	4,1979236	2,69E-05	0,0002424	PLEKHB2
ENSGALG00000016108	377,17299	0,58441259	0,2150799	2,7171887	0,0065839	0,0251303	SYBU
ENSGALG00000025738	2313,9355	0,584187633	0,1378228	4,2386855	2,25E-05	0,0002075	RHOU
ENSGALG00000028942	551,36062	0,584183813	0,2020534	2,8912352	0,0038373	0,0161649	ZBTB25
ENSGALG00000015938	2888,9851	0,583396297	0,1447547	4,0302413	5,57E-05	0,0004524	IFNAR2
ENSGALG00000001574	3769,6924	0,583342794	0,1296384	4,499767	6,80E-06	7,24E-05	MLLT6
ENSGALG00000014242	1647,3047	0,583190799	0,1556527	3,7467429	0,0001791	0,0012457	BEND4
ENSGALG00000008999	3276,8472	0,583129446	0,1551657	3,7581073	0,0001712	0,001199	AFAP1L2
ENSGALG00000010706	6618,9625	0,582943162	0,1129494	5,161101	2,46E-07	3,64E-06	TENM3
ENSGALG00000015952	611,25698	0,582757747	0,2345109	2,4849925	0,0129554	0,0431161	B3GAT2
ENSGALG00000004844	6201,28	0,581688828	0,1476751	3,9389777	8,18E-05	0,0006305	SEMA6D
ENSGALG00000009604	5450,2438	0,581479374	0,1323792	4,392529	1,12E-05	0,0001142	DBX2
ENSGALG00000001446	6585,1604	0,581431635	0,1501068	3,8734532	0,0001073	0,0007966	GSN
ENSGALG00000023768	4728,0275	0,58136874	0,1164642	4,9918245	5,98E-07	8,14E-06	EPHA2
ENSGALG00000002707	937,59842	0,58133458	0,1802757	3,2246966	0,0012611	0,0064766	CHRNB2

ENSGALG00000029020	1577,3992	0,581217481	0,1719893	3,3793821	0,0007265	0,0041059	PPAPDC1A
ENSGALG00000028478	1030,9127	0,580829913	0,1824278	3,1838898	0,0014531	0,0072578	CELF3
ENSGALG00000007531	904,34237	0,57752222	0,1731776	3,3348561	0,0008534	0,0046815	ZFYVE27
ENSGALG00000006054	759,82179	0,576882549	0,1820676	3,1685082	0,0015322	0,0075694	CALCA
ENSGALG00000022926	4686,6657	0,576442566	0,1261533	4,569381	4,89E-06	5,45E-05	GPR162
ENSGALG00000013185	3965,0445	0,575558846	0,1485635	3,8741602	0,000107	0,0007965	PITPNM1
ENSGALG00000000869	855,33376	0,574690811	0,1735124	3,3121031	0,000926	0,0050248	ZDHHC18
ENSGALG00000006885	6383,5426	0,574664846	0,1264083	4,5461019	5,46E-06	5,96E-05	HRAS
ENSGALG00000017150	518,06641	0,574401936	0,1974777	2,9086925	0,0036294	0,0154277	ARHGAP20
ENSGALG00000015110	438,79213	0,573807714	0,2198442	2,6100658	0,0090525	0,0324512	FAM189A2
ENSGALG00000015136	2417,8066	0,573614313	0,1609296	3,5643815	0,0003647	0,0022957	ILDR1
ENSGALG00000015744	3529,1332	0,573305804	0,1255432	4,5666009	4,96E-06	5,50E-05	RP11-26J3.4
ENSGALG00000001489	8334,3262	0,572451499	0,129456	4,4219762	9,78E-06	0,000101	SSU72
ENSGALG00000014911	1071,9721	0,572338387	0,1572272	3,6402005	0,0002724	0,0017824	ARL15
ENSGALG00000015351	2719,2778	0,572206068	0,1704231	3,357561	0,0007863	0,0043931	CBLB
ENSGALG00000025818	8546,9056	0,571475138	0,109953	5,1974483	2,02E-07	3,06E-06	KCNK5
ENSGALG00000005549	1034,7545	0,569387138	0,1561531	3,6463379	0,000266	0,0017479	CUEDC1
ENSGALG00000024153	1062,2502	0,56872295	0,170972	3,326409	0,0008797	0,0048083	NLRX1
ENSGALG00000000186	787,09326	0,568415238	0,1771285	3,2090555	0,0013317	0,006773	MTCH1
ENSGALG00000001146	1103,9797	0,568231367	0,1686686	3,3689227	0,0007546	0,0042333	UBXN6
ENSGALG00000026471	418,38308	0,568003242	0,2176763	2,6093938	0,0090703	0,0324722	CRIP2
ENSGALG00000009403	580,12466	0,567910527	0,2061904	2,7543016	0,0058818	0,02288	GRK5
ENSGALG00000012788	1309,5248	0,567748724	0,1613363	3,5190387	0,0004331	0,0026598	PARP12
ENSGALG00000026703	524,51341	0,567716959	0,1939681	2,926857	0,0034241	0,0146738	SLC2A4RG
ENSGALG00000027275	1081,3036	0,567504466	0,2139281	2,6527819	0,0079831	0,0293917	PPP1R3B
ENSGALG00000008682	380,85967	0,566238069	0,2325764	2,4346325	0,0149069	0,0481399	SLC25A43
ENSGALG00000009880	899,08782	0,565731325	0,2142452	2,6405788	0,0082765	0,0302345	INPP4B
ENSGALG00000006873	2079,2766	0,565449117	0,1310751	4,3139312	1,60E-05	0,0001546	RASSF7
ENSGALG00000007358	319,01574	0,565211229	0,2295584	2,462168	0,01381	0,0452631	CILP
ENSGALG00000007093	1910,9478	0,563647311	0,1531316	3,6808026	0,0002325	0,0015558	CYTH1
ENSGALG00000002778	10757,974	0,563240809	0,1099995	5,1203932	3,05E-07	4,42E-06	C6orf106
ENSGALG00000028605	2035,8396	0,561167945	0,1578097	3,5559782	0,0003766	0,0023562	CRELD1
ENSGALG00000001478	436,63763	0,560376671	0,2038738	2,7486445	0,0059842	0,0232189	
ENSGALG00000002681	1896,5953	0,559637304	0,1492264	3,750257	0,0001767	0,0012328	HSD17B7
ENSGALG00000008416	491,34771	0,559354461	0,2224807	2,5141709	0,0119313	0,040409	CFAP61
ENSGALG00000016152	7208,3385	0,558712272	0,1259225	4,4369523	9,12E-06	9,51E-05	LY6E
ENSGALG00000021079	356,52842	0,558275751	0,2206598	2,5300293	0,0114053	0,0389865	RGS19
ENSGALG00000010307	1309,92	0,558082008	0,160225	3,4831137	0,0004956	0,0029792	ACYP1
ENSGALG00000007997	607,00115	0,557958539	0,1934786	2,883826	0,0039288	0,0164585	ACCS
ENSGALG00000011604	1003,2775	0,557581352	0,1841576	3,0277409	0,0024639	0,0111932	SLC4A4
ENSGALG00000010614	5022,3744	0,557460992	0,1255639	4,4396588	9,01E-06	9,41E-05	SLC25A4
ENSGALG00000001682	1516,3508	0,557286636	0,150105	3,712645	0,0002051	0,0013949	FBXL20
ENSGALG00000001238	3616,0144	0,556401234	0,1285764	4,3273971	1,51E-05	0,0001475	STX8
ENSGALG00000002286	73998,022	0,555324016	0,1110271	5,0016995	5,68E-07	7,79E-06	H3F3C
ENSGALG00000025787	2442,8908	0,554206894	0,1300356	4,2619625	2,03E-05	0,0001895	CDK5RAP3
ENSGALG00000004849	4017,8273	0,553445556	0,1159402	4,7735421	1,81E-06	2,21E-05	WSCD2
ENSGALG00000013929	1086,8441	0,552883419	0,2064792	2,6776709	0,0074136	0,0277069	PDGFRA
ENSGALG00000001766	1421,2057	0,552758547	0,1737186	3,1819197	0,001463	0,0072892	RNF165
ENSGALG00000005377	707,88046	0,551995057	0,1867603	2,9556343	0,0031203	0,0135944	WWOX
ENSGALG00000023025	647,83568	0,551042888	0,1904818	2,8928903	0,0038171	0,0160849	ZBTB42
ENSGALG00000010982	4422,5508	0,55035484	0,1393577	3,9492248	7,84E-05	0,0006062	NID1
ENSGALG00000000316	1699,5534	0,549437781	0,1376171	3,9925111	6,54E-05	0,0005177	PKNOX2
ENSGALG00000027194	1293,5716	0,548094206	0,1517129	3,6127065	0,000303	0,001959	PMVK
ENSGALG00000016166	3894,3455	0,547970517	0,1393864	3,9313064	8,45E-05	0,0006489	SLC45A4
ENSGALG00000006663	9717,8185	0,547489923	0,1171093	4,6750332	2,94E-06	3,41E-05	ARHGEF12
ENSGALG00000009092	736,78214	0,546285921	0,1858557	2,9393012	0,0032895	0,0141776	GTF2A1L
ENSGALG00000001966	2313,195	0,545673399	0,1456394	3,7467418	0,0001791	0,0012457	RP11-106M3.5
ENSGALG00000001864	1163,993	0,545343287	0,1686132	3,2342865	0,0012195	0,006301	C9orf72
ENSGALG00000003201	647,35709	0,545148693	0,199247	2,7360447	0,0062183	0,0239563	RP11-5A19.5
ENSGALG00000006598	2327,6663	0,544504824	0,1375901	3,9574412	7,58E-05	0,0005879	SORL1
ENSGALG00000004272	762,39975	0,543009602	0,1799766	3,017113	0,0025519	0,0114973	ASB1
ENSGALG00000015107	1737,1601	0,542439326	0,135653	3,9987266	6,37E-05	0,0005065	PIP5K1B

ENSGALG00000008860	2251,9145	0,541261109	0,1571898	3,4433603	0,0005745	0,0033644	LONRF3
ENSGALG00000004438	432,7024	0,540948656	0,2158033	2,5066749	0,0121873	0,0410717	ARSB
ENSGALG00000005315	1118,2152	0,540760838	0,1598616	3,3826823	0,0007178	0,0040636	RPUSD1
ENSGALG00000004033	1001,8846	0,540669975	0,1647104	3,2825495	0,0010287	0,0054647	GALNT10
ENSGALG00000016176	404,69822	0,540238641	0,2176628	2,4819979	0,0130648	0,0434589	BAI3
ENSGALG00000022713	4245,7427	0,539793648	0,1498998	3,6010297	0,000317	0,0020356	FAM181B
ENSGALG00000025800	976,78159	0,538353026	0,1626794	3,3092876	0,0009353	0,0050615	
ENSGALG00000028513	5510,2985	0,53711532	0,1123093	4,7824632	1,73E-06	2,12E-05	TMUB1
ENSGALG00000026466	809,72654	0,537047161	0,1854082	2,8965663	0,0037727	0,015947	
ENSGALG00000015437	3953,661	0,536831128	0,1328102	4,0420929	5,30E-05	0,0004329	MPC2
ENSGALG00000024049	660,99489	0,536773384	0,1891289	2,8381347	0,0045378	0,0184744	RAB33A
ENSGALG00000029027	627,86616	0,53597017	0,182674	2,9340251	0,003346	0,0143844	
ENSGALG00000007389	2084,8413	0,53455468	0,1327207	4,0276668	5,63E-05	0,0004565	ANKRD40
ENSGALG00000021313	982,25761	0,534412472	0,180088	2,9675073	0,0030023	0,0131729	PDGFRB
ENSGALG00000016037	768,10735	0,532870331	0,1779289	2,9948499	0,0027458	0,0122611	SPAG1
ENSGALG00000009805	563,53778	0,53211751	0,1982743	2,6837437	0,0072803	0,0272932	PPM1H
ENSGALG00000026039	442,77163	0,531809088	0,2097706	2,5351933	0,0112385	0,0385811	
ENSGALG00000016167	2827,4394	0,530005093	0,1278741	4,1447408	3,40E-05	0,0002958	DENND3
ENSGALG00000027498	3720,6236	0,529700401	0,1865582	2,8393312	0,0045208	0,0184174	FOXB1
ENSGALG00000023189	809,88073	0,529094048	0,1674238	3,1602079	0,0015766	0,0077603	UMAD1
ENSGALG00000009433	12972,3	0,528259009	0,1367266	3,8636145	0,0001117	0,0008235	BAG3
ENSGALG00000011350	565,5219	0,52709173	0,1912783	2,7556272	0,005858	0,0228071	NEGR1
ENSGALG00000015372	6118,1225	0,526646329	0,1098598	4,7938042	1,64E-06	2,03E-05	ATP5I
ENSGALG00000024346	1302,1617	0,525612903	0,1486838	3,5351054	0,0004076	0,0025272	FAM46B
ENSGALG00000013839	706,87354	0,524875259	0,1772725	2,9608384	0,003068	0,0134226	
ENSGALG00000015306	3107,5418	0,524586766	0,1467809	3,5739432	0,0003516	0,0022279	OSTM1
ENSGALG00000026545	5534,2449	0,524540794	0,126285	4,1536273	3,27E-05	0,000286	
ENSGALG00000002777	12410,374	0,524514209	0,1049816	4,996249	5,85E-07	7,98E-06	ACAA2
ENSGALG00000015326	10708,875	0,524363909	0,1179079	4,4472344	8,70E-06	9,12E-05	CLTA
ENSGALG00000014846	1408,263	0,524315684	0,1475731	3,5529214	0,000381	0,0023794	OXCT1
ENSGALG00000011945	1101,0533	0,524157211	0,1675307	3,1287225	0,0017557	0,0084616	SCRN2
ENSGALG00000011349	1290,6252	0,523508946	0,2028294	2,5810314	0,0098506	0,0347594	MNR2
ENSGALG00000006901	2767,4012	0,523326668	0,1242839	4,2107361	2,55E-05	0,0002309	AATK
ENSGALG00000026113	4499,0711	0,523056553	0,1465805	3,5683905	0,0003592	0,0022661	CARHSP1
ENSGALG00000000433	145504,42	0,523011809	0,1027427	5,0905032	3,57E-07	5,11E-06	TUBA4A
ENSGALG00000016886	6214,4284	0,522545779	0,1102341	4,7403267	2,13E-06	2,56E-05	FARP1
ENSGALG00000014321	925,70397	0,522295082	0,1727289	3,0237853	0,0024963	0,0113033	SMIM14
ENSGALG00000008367	12605,915	0,522250337	0,1198719	4,3567381	1,32E-05	0,0001314	MDK
ENSGALG00000023812	573,55559	0,520869219	0,1910734	2,7260163	0,0064104	0,024599	CCNI2
ENSGALG00000008911	1167,8871	0,520705327	0,1637362	3,1801469	0,001472	0,0073224	RNF208
ENSGALG00000023338	5372,4298	0,520605081	0,1279451	4,0689734	4,72E-05	0,0003908	CBX2
ENSGALG00000016266	844,50002	0,519693116	0,1718383	3,0243148	0,002492	0,0112906	PRRG1
ENSGALG00000027069	549,10808	0,519660528	0,2040614	2,5465893	0,0108781	0,037619	TSC22D3
ENSGALG00000001944	1567,5072	0,519218499	0,1502243	3,4562878	0,0005477	0,0032349	TBC1D2
ENSGALG00000000059	125389,82	0,519152064	0,1205209	4,307569	1,65E-05	0,0001587	TUBB3
ENSGALG00000004775	978,22061	0,519049003	0,1721556	3,0149984	0,0025698	0,0115624	COLGALT2
ENSGALG00000005346	2351,8167	0,518505546	0,1295966	4,0009207	6,31E-05	0,0005021	PPIC
ENSGALG00000026560	1526,6551	0,518030334	0,1900719	2,7254446	0,0064215	0,0246347	PRDM8
ENSGALG00000028033	2016,6076	0,517359519	0,1337773	3,8673194	0,00011	0,0008129	PURA
ENSGALG00000026891	1109,4806	0,516642503	0,1835376	2,814914	0,004879	0,0196824	NINJ1
ENSGALG00000013024	1376,2985	0,516485359	0,1557483	3,3161536	0,0009127	0,0049604	IL17RA
ENSGALG00000028567	2485,7677	0,515072598	0,1278731	4,027999	5,63E-05	0,0004562	MYL9
ENSGALG00000001998	1765,5757	0,514536405	0,138305	3,7203024	0,000199	0,0013569	R3HDM4
ENSGALG00000027073	784,17281	0,514122556	0,1915064	2,684623	0,0072612	0,0272551	AAMDC
ENSGALG00000012089	5360,5907	0,513995299	0,1209653	4,2491144	2,15E-05	0,0001992	C14orf37
ENSGALG00000013708	5397,0941	0,513478256	0,1315099	3,9044846	9,44E-05	0,0007134	CYB5A
ENSGALG00000029159	2974,1732	0,51305649	0,129165	3,9721007	7,12E-05	0,00056	APBB1
ENSGALG00000005901	2799,2351	0,510684045	0,1324352	3,8561047	0,0001152	0,0008451	PCMTD2
ENSGALG00000023786	1008,229	0,510469642	0,1622026	3,1471121	0,0016489	0,008038	PLEKHH3
ENSGALG00000017053	1433,2665	0,50974214	0,1465107	3,4792149	0,0005029	0,0030135	SPG20
ENSGALG00000004741	5108,3909	0,509578858	0,1446763	3,5221998	0,000428	0,0026378	EPHB2
ENSGALG00000012644	1843,5171	0,509005651	0,1372404	3,7088609	0,0002082	0,0014145	FOXD3_CHICK

ENSGALG00000012697	713,61608	0,508988728	0,1895408	2,6853778	0,0072448	0,0272029	SLC41A2
ENSGALG00000016014	587,48188	0,508625141	0,2007004	2,534251	0,0112688	0,0386655	RCAN1
ENSGALG00000029036	2011,0671	0,506773529	0,1625524	3,1176018	0,0018233	0,0087194	ZNF687
ENSGALG00000016708	1004,6127	0,506256389	0,1774268	2,8533257	0,0043264	0,0177744	ENPP4
ENSGALG00000011324	77589,574	0,50624037	0,1039388	4,8705612	1,11E-06	1,44E-05	TUBA4B
ENSGALG00000015597	932,24281	0,505967585	0,1755067	2,8828969	0,0039404	0,0164962	CPZ
ENSGALG00000005757	2287,1727	0,505955901	0,148039	3,4177214	0,0006315	0,0036399	IRF8
ENSGALG00000011942	693,1373	0,505923034	0,1765129	2,8662097	0,0041542	0,0172007	
ENSGALG00000003175	433,6143	0,505815122	0,2067003	2,4470941	0,0144013	0,0467957	STKLD1
ENSGALG00000012670	2710,2763	0,505329169	0,1395627	3,6208026	0,0002937	0,0019059	NRSN1
ENSGALG00000028145	4237,9607	0,505320547	0,1554994	3,2496615	0,0011554	0,0060245	
ENSGALG00000027891	10377,445	0,504324315	0,1084665	4,649585	3,33E-06	3,83E-05	NREP
ENSGALG00000024295	3217,4381	0,504249612	0,1324209	3,8079298	0,0001401	0,0010058	
ENSGALG00000008631	3955,1009	0,503418102	0,1318317	3,8186431	0,0001342	0,0009682	TYRO3
ENSGALG00000015026	714,61563	0,503288049	0,182056	2,7644677	0,0057016	0,0223002	TRAF3IP2
ENSGALG00000005811	1644,0505	0,503226947	0,1577089	3,1908596	0,0014185	0,0071292	COL7A1
ENSGALG00000006802	2159,6578	0,502556738	0,1878266	2,6756417	0,0074586	0,0278522	LAMB2
ENSGALG00000025977	2572,6109	0,502103804	0,1296104	3,8739483	0,0001071	0,0007966	GADD45A
ENSGALG00000023554	790,06032	0,500814372	0,1776938	2,8184128	0,0048262	0,0194807	PIGW
ENSGALG00000024398	21096,229	0,500531072	0,1162284	4,3064435	1,66E-05	0,0001594	RPS28
ENSGALG00000004851	3943,1572	0,500191453	0,1444617	3,4624504	0,0005353	0,0031741	MED9
ENSGALG00000008341	3732,2674	0,499807137	0,1274369	3,9219961	8,78E-05	0,0006725	FURIN
ENSGALG00000012487	1459,0736	0,499098415	0,1787383	2,7923417	0,0052328	0,0208196	MPST
ENSGALG00000011326	2703,9221	0,4983963	0,1426307	3,4943128	0,0004753	0,0028759	STK16
ENSGALG00000000842	2625,6895	0,49824929	0,1497155	3,3279751	0,0008748	0,0047852	NDUFA2
ENSGALG00000011181	876,58708	0,497942212	0,1756687	2,834553	0,004589	0,0186403	FKBP14
ENSGALG00000018819	651,0396	0,497763645	0,1880595	2,6468407	0,0081248	0,0297602	TBCEL
ENSGALG00000007370	4047,939	0,4969378	0,1207586	4,115134	3,87E-05	0,0003312	CLP1
ENSGALG00000012947	2224,1495	0,495800698	0,1377049	3,6004578	0,0003177	0,0020391	CAPRIN2
ENSGALG00000013067	8815,6345	0,494753671	0,1163549	4,2521102	2,12E-05	0,0001972	AKR1B1
ENSGALG00000007441	1294,7426	0,494713822	0,1878271	2,633879	0,0084416	0,030714	MAP6
ENSGALG00000000527	6471,7809	0,494652161	0,1195904	4,1362185	3,53E-05	0,0003062	SH3BGRL3
ENSGALG00000012540	1788,9218	0,494557337	0,1380803	3,5816639	0,0003414	0,0021731	RBFOX2
ENSGALG00000011778	2592,1932	0,494085012	0,1468186	3,3652744	0,0007647	0,0042862	ITGB5
ENSGALG00000009090	635,87673	0,493735019	0,1952486	2,5287506	0,0114469	0,0390797	UAP1L1
ENSGALG00000001242	570,55086	0,493511493	0,1867093	2,6432083	0,0082124	0,030041	
ENSGALG00000027824	1654,6946	0,493225579	0,1467768	3,360379	0,0007784	0,0043539	MRPL55
ENSGALG00000005897	4766,7142	0,493064333	0,1262096	3,9067089	9,36E-05	0,0007093	HMCES
ENSGALG00000012816	2261,4061	0,492973169	0,1565978	3,1480205	0,0016438	0,0080223	SLC22A23
ENSGALG00000005071	6694,0161	0,492854493	0,1137172	4,3340352	1,46E-05	0,000144	MPP1
ENSGALG00000000336	3779,8129	0,492268059	0,1394428	3,5302496	0,0004152	0,0025705	PPP1R12B
ENSGALG00000004351	891,83433	0,49204731	0,1689419	2,9125234	0,0035852	0,0152683	
ENSGALG00000004902	4281,0079	0,491426093	0,1175409	4,1808935	2,90E-05	0,0002581	TOM1L2
ENSGALG00000026195	759,73378	0,491395512	0,1717565	2,8610013	0,0042231	0,017455	GALT
ENSGALG00000011894	1400,2218	0,491394089	0,1454582	3,3782503	0,0007295	0,0041177	CYP2D6
ENSGALG00000017076	6428,3314	0,490620681	0,1467776	3,3426126	0,0008299	0,0045704	B3GLCT
ENSGALG00000008409	5336,4487	0,490103959	0,1431132	3,4245884	0,0006157	0,0035734	GSTO1
ENSGALG00000005751	599,07782	0,487576383	0,1863158	2,6169354	0,0088723	0,0319489	RAB26
ENSGALG00000005299	758,00774	0,487367317	0,1823876	2,6721512	0,0075367	0,028082	ANXA7
ENSGALG00000010230	677,39962	0,486896744	0,1774474	2,7438932	0,0060715	0,0234907	TESK2
ENSGALG00000011531	2214,443	0,486736013	0,1514726	3,2133593	0,0013119	0,0066923	APAF1
ENSGALG00000006915	1392,1391	0,485309324	0,1555538	3,1198817	0,0018092	0,0086661	BAIAP2
ENSGALG00000011798	1079,8245	0,484782273	0,1756184	2,7604306	0,0057725	0,0225205	HEBP1
ENSGALG00000017419	644,62948	0,484006649	0,1950269	2,4817427	0,0130742	0,0434688	HPS1
ENSGALG00000000947	2256,1097	0,482847185	0,1494215	3,2314447	0,0012317	0,0063519	FKBP5
ENSGALG00000027173	1663,1472	0,482690141	0,1744895	2,7662985	0,0056697	0,0222137	TMEM159
ENSGALG00000008604	829,00435	0,482676253	0,1667486	2,8946345	0,003796	0,0160157	TMEM255A
ENSGALG00000014581	2314,88	0,482286067	0,1382204	3,4892527	0,0004844	0,0029219	MEF2BNB
ENSGALG00000011738	6621,7844	0,482221251	0,1182329	4,0785695	4,53E-05	0,0003771	ARHGDIB
ENSGALG00000027688	6057,2057	0,481915396	0,1181641	4,0783585	4,54E-05	0,0003772	NDUFB4
ENSGALG00000004476	498,99618	0,48187718	0,1958501	2,4604391	0,0138767	0,045449	SEMA3G
ENSGALG00000000625	1809,062	0,481753107	0,1576512	3,0558166	0,0022445	0,0103589	MAPT

ENSGALG00000015774	1090,9886	0,481727224	0,159212	3,0256962	0,0024806	0,0112579	CYYR1
ENSGALG00000001763	1095,0503	0,481398992	0,1634996	2,9443437	0,0032364	0,0140062	CZH18ORF25
ENSGALG00000005713	4508,0892	0,480930735	0,1143118	4,2071834	2,59E-05	0,0002339	NCKIPSD
ENSGALG00000022838	522,00396	0,480740174	0,1959359	2,4535587	0,014145	0,0461834	GAREM2
ENSGALG00000003405	5095,9892	0,480657067	0,1124415	4,2747319	1,91E-05	0,0001804	BSDC1
ENSGALG00000002033	6624,4207	0,480537464	0,1111006	4,3252479	1,52E-05	0,0001482	NDUFB6
ENSGALG00000001531	815,50813	0,480440457	0,1690567	2,8418887	0,0044847	0,0182867	FN3K
ENSGALG00000029135	7403,5283	0,479980361	0,1296505	3,7021087	0,0002138	0,0014476	YEATS4
ENSGALG00000011436	3916,7711	0,477975314	0,1192676	4,0075871	6,13E-05	0,0004904	TMBIM1
ENSGALG00000003466	4818,2759	0,477695903	0,1342072	3,5593901	0,0003717	0,0023355	AKR1B1L
ENSGALG00000007661	1313,7584	0,477627101	0,1756565	2,7190975	0,006546	0,0250419	MYCBPAP
ENSGALG00000029123	831,21662	0,477624117	0,1743995	2,7386782	0,0061687	0,0238124	RBFOX3
ENSGALG00000001606	1726,3623	0,477443775	0,1469062	3,2499912	0,0011541	0,0060203	INPP5B
ENSGALG00000000785	1523,6714	0,476932316	0,1468983	3,2466828	0,0011676	0,0060741	HYAL1
ENSGALG00000028573	1369,3549	0,476874115	0,1566656	3,0438978	0,0023353	0,0107165	SAYSD1
ENSGALG00000014765	2914,6817	0,476665637	0,1245755	3,8263205	0,0001301	0,0009415	TRIM23
ENSGALG00000016994	1571,3108	0,474313428	0,1588828	2,9853031	0,002833	0,012572	NUDT15
ENSGALG00000000943	4846,5059	0,473716041	0,112782	4,2002794	2,67E-05	0,0002402	WDTC1
ENSGALG00000006588	1949,8657	0,473632802	0,1386756	3,4154005	0,0006369	0,0036653	LRRC75B
ENSGALG00000012704	1775,4396	0,473322212	0,1740897	2,7188406	0,0065511	0,0250543	MYLIP
ENSGALG00000002615	1139,5869	0,472751564	0,1639644	2,883258	0,0039358	0,0164831	DEF6
ENSGALG00000023157	3625,8738	0,472554642	0,1422354	3,3223424	0,0008927	0,0048731	REEP5
ENSGALG00000001849	1893,9454	0,47187756	0,1796068	2,6272817	0,008607	0,0311909	BSN
ENSGALG00000006702	2572,8058	0,471728545	0,1314835	3,5877394	0,0003336	0,0021305	MFGE8
ENSGALG00000003359	6429,5457	0,469661247	0,1200374	3,9126251	9,13E-05	0,0006948	RAB5C
ENSGALG00000027377	1588,8633	0,469161575	0,1429069	3,2829883	0,0010271	0,0054584	NKAIN3
ENSGALG00000013511	6097,6262	0,469095656	0,1205264	3,892056	9,94E-05	0,000746	ANKRA2
ENSGALG00000014963	978,12348	0,468684934	0,1590044	2,9476227	0,0032023	0,0138939	DSE
ENSGALG00000016988	780,62352	0,468570179	0,173966	2,6934591	0,0070715	0,026633	KIAA0226L
ENSGALG00000003348	1706,407	0,468370054	0,138122	3,3909876	0,0006964	0,0039639	HIGD2A
ENSGALG00000027993	718,86063	0,467989481	0,184762	2,5329319	0,0113113	0,0387543	MXD3
ENSGALG00000028342	3684,9232	0,4672884	0,1405267	3,3252632	0,0008834	0,0048262	CHRAC1
ENSGALG00000004581	7502,7339	0,466855623	0,1169353	3,9924255	6,54E-05	0,0005177	NDST1
ENSGALG00000013795	1427,0027	0,466841888	0,1691704	2,759595	0,0057873	0,0225577	HIVEP2
ENSGALG00000007238	3294,4647	0,466556765	0,1396865	3,3400276	0,0008377	0,0046082	PCSK6
ENSGALG00000001556	1997,4095	0,466292104	0,1362595	3,4220882	0,0006214	0,0035957	ROMO1
ENSGALG00000006942	9942,2696	0,465540173	0,155783	2,988388	0,0028045	0,0124702	PIK3IP1
ENSGALG00000009655	1343,4112	0,465196113	0,1463089	3,1795467	0,0014751	0,0073322	AADAT
ENSGALG00000005074	969,33864	0,464823262	0,1663096	2,7949274	0,0051911	0,020684	FAM102A
ENSGALG00000002485	4126,4279	0,464623616	0,1275886	3,6415767	0,000271	0,0017746	PHKG1
ENSGALG00000011474	2398,5188	0,463345487	0,1386209	3,3425371	0,0008302	0,0045704	OSBPL10
ENSGALG00000013698	1914,3877	0,462883571	0,1399784	3,3068211	0,0009436	0,0051001	EFHD2
ENSGALG00000007582	9089,3986	0,462276159	0,111854	4,1328546	3,58E-05	0,0003098	DPYSL3
ENSGALG00000003976	1632,2576	0,461042882	0,1442377	3,1964111	0,0013915	0,0070167	BCORL1
ENSGALG00000024391	9415,8207	0,460947942	0,1147209	4,0179932	5,87E-05	0,0004723	CACTIN
ENSGALG00000027418	872,62308	0,460841146	0,1699387	2,7118078	0,0066917	0,0255122	HINT2
ENSGALG00000003406	1016,1622	0,460645128	0,185859	2,4784659	0,0131949	0,0437898	RXRG
ENSGALG00000014818	715,41879	0,460609845	0,1805705	2,5508589	0,0107458	0,0372559	TRIM45
ENSGALG00000024034	3571,4505	0,460548751	0,1231211	3,740617	0,0001836	0,0012713	PI4KB
ENSGALG00000014226	4001,8851	0,460133597	0,1394922	3,2986321	0,0009716	0,0052095	KIAA0930
ENSGALG00000022980	7902,7397	0,459059074	0,1123607	4,0855838	4,40E-05	0,0003668	CDKN1B
ENSGALG00000001728	1860,8126	0,458348865	0,1498189	3,0593533	0,0022182	0,0102792	NACC2
ENSGALG00000014324	2616,4144	0,458208088	0,1484479	3,0866594	0,0020242	0,0095394	SLC2A3
ENSGALG00000026871	2483,7701	0,457953468	0,1821233	2,5145249	0,0119193	0,0403885	SC5D
ENSGALG00000015972	3109,9302	0,45786464	0,1525893	3,0006332	0,0026942	0,0120504	IFNGR2
ENSGALG00000001881	2571,7226	0,456046301	0,1474962	3,0919195	0,0019887	0,0093882	DNLZ
ENSGALG00000001779	903,85433	0,455324974	0,1721096	2,6455517	0,0081558	0,0298497	KIAA0895L
ENSGALG00000000921	4858,4012	0,454646053	0,1160077	3,9191038	8,89E-05	0,0006791	SLC9A1
ENSGALG00000004537	5387,8208	0,454616982	0,1154772	3,9368562	8,26E-05	0,0006354	EFNB1
ENSGALG00000016870	5615,9964	0,454001604	0,1190199	3,8145028	0,0001365	0,000984	TMTC4
ENSGALG00000011655	1599,7524	0,452530841	0,1507993	3,0008809	0,002692	0,0120445	IQCB1
ENSGALG00000011592	753,887	0,452054353	0,1756234	2,5739982	0,0100531	0,0353038	MRAS

ENSGALG00000027963	10511,195	0,451771641	0,1053151	4,2897137	1,79E-05	0,0001697	COX7C
ENSGALG00000012110	938,42508	0,451377511	0,1665857	2,7095817	0,0067368	0,0256277	KIAA0895
ENSGALG00000009054	1742,5243	0,451368563	0,1444522	3,1246911	0,0017799	0,008551	TMEM141
ENSGALG00000017351	2392,614	0,45115395	0,1297902	3,4760237	0,0005089	0,0030416	STIM1
ENSGALG00000007286	2211,1348	0,450759437	0,1433837	3,1437292	0,0016681	0,0081111	SIRT7
ENSGALG00000004623	967,41624	0,450139696	0,1793951	2,5092091	0,0121002	0,0408428	AGTRAP
ENSGALG00000001376	9938,3188	0,45012042	0,1054385	4,2690347	1,96E-05	0,0001846	ARID3B
ENSGALG00000017126	2105,4507	0,449836372	0,1445661	3,111632	0,0018606	0,0088634	ZDHHC20
ENSGALG00000004353	9460,5472	0,448918917	0,1126661	3,9845069	6,76E-05	0,0005331	TBCA
ENSGALG00000015780	22777,794	0,447991765	0,1105939	4,0507816	5,10E-05	0,0004182	PNRC1
ENSGALG00000026200	2407,7782	0,447943252	0,1654706	2,7070867	0,0067877	0,0257707	FAM134B
ENSGALG00000012218	3810,3697	0,44776649	0,1283039	3,4898898	0,0004832	0,0029175	CBY1
ENSGALG00000008274	1660,0276	0,446767639	0,1420336	3,1455061	0,001658	0,0080707	CRBN
ENSGALG00000005608	3810,2468	0,44662731	0,1300871	3,4332951	0,0005963	0,0034739	SLC9A3R2
ENSGALG00000007296	1317,9251	0,446130433	0,1486148	3,0019248	0,0026828	0,0120073	MAFG
ENSGALG00000016545	1336,374	0,445723593	0,1531647	2,910094	0,0036132	0,0153682	REPS2
ENSGALG00000017329	3706,9673	0,44555284	0,1363133	3,2685934	0,0010808	0,0057037	FCHSD2
ENSGALG00000016210	979,19029	0,445450514	0,1608438	2,7694608	0,0056149	0,0220587	ST3GAL1
ENSGALG00000015987	4807,1926	0,445391941	0,1245569	3,5758102	0,0003491	0,0022157	TP53INP1
ENSGALG00000008681	1024,5364	0,444903231	0,167862	2,65041	0,0080394	0,029535	NSMF
ENSGALG00000000067	2534,6314	0,444870546	0,1286038	3,4592336	0,0005417	0,0032081	SPR
ENSGALG00000011921	3797,7786	0,444716768	0,1414811	3,1432948	0,0016706	0,0081203	DHRS7
ENSGALG00000000233	2504,2434	0,444272958	0,1492937	2,9758312	0,002922	0,0128913	SP2
ENSGALG00000015344	1754,4897	0,442789405	0,1532928	2,88852	0,0038706	0,0162799	NXPE3
ENSGALG00000027337	6395,1608	0,441351335	0,1148234	3,8437395	0,0001212	0,0008818	CLDND1
ENSGALG00000004907	2377,2693	0,441232207	0,1337197	3,2996803	0,000968	0,0051966	KCTD15
ENSGALG00000002626	1744,7946	0,440405851	0,1788023	2,4630889	0,0137746	0,0451855	RXRA
ENSGALG00000017096	2040,4546	0,440278375	0,1337931	3,29074	0,0009992	0,0053268	LNX2
ENSGALG00000012043	13548,036	0,439717314	0,1042663	4,2172536	2,47E-05	0,0002255	PDCD6IP
ENSGALG00000012265	3656,8809	0,439419657	0,1289098	3,4087374	0,0006526	0,0037398	MICALL1
ENSGALG00000015988	2456,5859	0,439214581	0,1576704	2,785651	0,005342	0,0211799	PLEKHF2
ENSGALG00000002986	2686,672	0,439006087	0,125125	3,5085396	0,0004506	0,0027448	WNK4
ENSGALG00000010811	7190,0128	0,438424216	0,1472899	2,9766068	0,0029146	0,0128629	LGMN
ENSGALG00000005820	8527,7428	0,438136271	0,1128485	3,8825187	0,0001034	0,0007734	RNF130
ENSGALG00000012913	1327,8126	0,437865562	0,1766936	2,478107	0,0132082	0,043818	PKP2
ENSGALG00000006204	1464,0386	0,437842966	0,1451037	3,0174484	0,0025491	0,0114911	CHST13
ENSGALG00000012136	1595,9568	0,437388472	0,1538246	2,8434236	0,0044632	0,0182316	MPPED2
ENSGALG00000026116	1987,2825	0,437155594	0,135619	3,2234086	0,0012667	0,006496	MAPRE3
ENSGALG00000013091	2461,6299	0,436802578	0,1334788	3,272449	0,0010662	0,0056418	EPS8
ENSGALG00000008444	16562,743	0,436671101	0,1126641	3,8758677	0,0001062	0,0007926	TUBAL3
ENSGALG00000012821	60743,332	0,436526282	0,1102259	3,9602861	7,49E-05	0,0005824	TUBB2B
ENSGALG00000016322	1469,7377	0,435481617	0,165226	2,6356732	0,0083971	0,030593	
ENSGALG00000006399	2888,1679	0,435019992	0,1286562	3,38126	0,0007215	0,0040828	MESDC1
ENSGALG00000001289	1028,449	0,434897808	0,1641592	2,6492441	0,0080672	0,0296038	SHC2
ENSGALG00000010134	2529,1958	0,434710794	0,126122	3,4467496	0,0005674	0,003334	ZFAND3
ENSGALG00000000270	895,80796	0,434589704	0,1737942	2,500599	0,0123983	0,0416902	THRA
ENSGALG00000016059	2071,2967	0,434358409	0,1376843	3,1547427	0,0016064	0,0078758	ETS2
ENSGALG00000000049	1247,3305	0,433824072	0,1496385	2,8991468	0,0037418	0,015841	CSDC2
ENSGALG00000006655	1295,5112	0,433042079	0,15427	2,8070402	0,0049999	0,0200672	MAP3K13
ENSGALG00000021365	7390,78	0,432965219	0,1172033	3,6941392	0,0002206	0,0014864	DCTN3
ENSGALG00000003447	2587,3652	0,432921053	0,1381602	3,1334707	0,0017275	0,0083553	FRS3
ENSGALG00000015607	3625,3575	0,432451982	0,1684544	2,5671749	0,0102531	0,035895	NCOA2
ENSGALG00000004725	7054,5494	0,431307897	0,129893	3,3204852	0,0008986	0,0048938	RP11-162P23.2
ENSGALG00000014410	1635,3072	0,430076039	0,1754863	2,450767	0,0142552	0,0464477	CSTB
ENSGALG00000029157	4821,9983	0,430051945	0,1512627	2,8430801	0,004468	0,0182458	MBNL3
ENSGALG00000000551	4770,6021	0,428841133	0,1175738	3,6474204	0,0002649	0,0017414	C6orf89
ENSGALG00000014804	1784,8867	0,427778224	0,1683627	2,5408135	0,0110595	0,0381106	THBS4
ENSGALG00000005295	1837,9552	0,4276113	0,1611793	2,653017	0,0079776	0,029382	BCAS3
ENSGALG00000023612	4284,8687	0,427551312	0,1222371	3,4977227	0,0004692	0,0028433	LZTS2
ENSGALG00000010838	5580,7171	0,427079258	0,1310404	3,2591413	0,0011175	0,0058586	ERO1LB
ENSGALG00000016605	1894,8074	0,426964808	0,1498455	2,8493661	0,0043806	0,0179484	EFR3B
ENSGALG00000007630	1371,8459	0,426278895	0,1475832	2,8883966	0,0038721	0,0162813	CDIP1

ENSGALG00000026022	1208,8026	0,425857109	0,1749797	2,4337512	0,0149433	0,0482458	CREG2
ENSGALG00000027822	849,07694	0,425453865	0,1646142	2,5845511	0,0097506	0,0344727	
ENSGALG00000015304	5360,7582	0,425237788	0,1168587	3,6389042	0,0002738	0,0017905	SNX3
ENSGALG00000020528	5739,52	0,424819785	0,1109172	3,8300637	0,0001281	0,0009293	INIP
ENSGALG00000014569	787,05235	0,42466715	0,1733979	2,4490901	0,0143218	0,0466262	PNPLA2
ENSGALG00000015603	3809,7497	0,424497436	0,1338532	3,171366	0,0015172	0,0075035	ZCCHC9
ENSGALG00000021688	803,0907	0,424387775	0,170819	2,4844292	0,0129759	0,0431738	CD320
ENSGALG00000001975	1467,2899	0,42427092	0,1628237	2,6057072	0,0091685	0,0327635	TMEM120A
ENSGALG00000026692	7703,2554	0,424103985	0,1361891	3,1140823	0,0018452	0,0088024	NRAS
ENSGALG00000013194	2699,5463	0,423818786	0,1417822	2,9892249	0,0027969	0,0124483	IRX1
ENSGALG00000015903	1395,6597	0,423426358	0,1458209	2,9037432	0,0036873	0,0156395	FILIP1
ENSGALG00000012009	3507,1232	0,422977808	0,1235894	3,4224446	0,0006206	0,0035925	JKAMP
ENSGALG00000009107	1525,7253	0,422108392	0,149748	2,8187922	0,0048205	0,0194634	NRXN1
ENSGALG00000008257	1616,4159	0,421896886	0,1646167	2,5629047	0,0103801	0,036237	MERTK
ENSGALG00000027710	12308,995	0,421872733	0,1075864	3,9212473	8,81E-05	0,0006742	DAG1
ENSGALG00000000436	3616,9217	0,421563067	0,1280766	3,2914916	0,0009966	0,0053161	RABIF
ENSGALG00000008613	2544,3778	0,420884082	0,1331062	3,1620172	0,0015668	0,007729	NDUFA1
ENSGALG00000028302	1437,8585	0,420702301	0,1488301	2,826728	0,0047026	0,0190554	
ENSGALG00000012131	1906,2436	0,419815141	0,1493221	2,8114727	0,0049315	0,0198588	ELOVL6
ENSGALG00000009595	1381,8135	0,419771594	0,1544649	2,7175851	0,006576	0,0251107	TWF1
ENSGALG00000007619	4223,0444	0,419444023	0,1439729	2,9133548	0,0035757	0,015242	UBALD1
ENSGALG00000007025	838,27153	0,41933553	0,165861	2,5282343	0,0114638	0,0391176	CPNE8
ENSGALG00000007054	5550,7308	0,419262331	0,1227261	3,4162453	0,0006349	0,0036566	TBC1D16
ENSGALG00000017675	6606,9674	0,417757976	0,11207	3,7276537	0,0001933	0,0013256	NDUFS8
ENSGALG00000008818	5178,1883	0,417517144	0,1293144	3,2286974	0,0012436	0,0064011	IDH1
ENSGALG00000004038	1559,4978	0,417388311	0,1404734	2,9712977	0,0029654	0,0130493	ADAL
ENSGALG00000026394	6922,0955	0,417110916	0,1102725	3,782546	0,0001552	0,0010991	CORO1B
ENSGALG00000002840	4242,1731	0,415750464	0,1518147	2,7385381	0,0061713	0,0238158	GRM4
ENSGALG00000007220	48744,176	0,415111704	0,1048964	3,9573492	7,58E-05	0,0005879	FTH1
ENSGALG00000001816	2247,2353	0,414935997	0,1327278	3,1262169	0,0017707	0,0085128	MAGI3
ENSGALG00000028812	2137,7927	0,414162814	0,1436705	2,8827261	0,0039425	0,0164962	CREBL2
ENSGALG00000015352	2437,8785	0,413775598	0,1391873	2,9727977	0,002951	0,0130039	GAK
ENSGALG00000026802	39202,809	0,413746284	0,1146157	3,6098563	0,0003064	0,0019787	TUBB2A
ENSGALG00000015915	2924,5989	0,413737551	0,1308763	3,1612868	0,0015707	0,0077372	TMEM64
ENSGALG00000020626	1251,1665	0,41305269	0,1546192	2,6714191	0,0075531	0,0281203	C3ORF55
ENSGALG00000012426	3567,3724	0,412512543	0,125055	3,2986494	0,0009715	0,0052095	DDHD1
ENSGALG00000004918	890,67566	0,411822203	0,1629138	2,5278534	0,0114762	0,0391454	RAB9B
ENSGALG00000001201	7498,136	0,411468635	0,10775	3,8187341	0,0001341	0,0009682	GFOD2
ENSGALG00000024081	3055,1077	0,410810718	0,129763	3,1658532	0,0015463	0,0076361	
ENSGALG00000004873	2191,1914	0,410670756	0,1393688	2,9466478	0,0032124	0,0139255	PHACTR3
ENSGALG00000017405	1260,4536	0,410522272	0,1629615	2,5191367	0,0117643	0,0399927	NPR3
ENSGALG00000003750	7887,3706	0,409049332	0,1094937	3,7358254	0,0001871	0,0012918	PLCG1
ENSGALG00000016828	4406,9031	0,407053917	0,1467975	2,7728942	0,005556	0,0219007	GRTP1
ENSGALG00000000478	3159,4613	0,406777826	0,1532189	2,6548799	0,0079337	0,0292412	TANC2
ENSGALG00000015004	7430,1607	0,406416416	0,1365123	2,9771421	0,0029095	0,0128446	RIOK3
ENSGALG00000011488	1838,711	0,405575212	0,1659995	2,443232	0,0145564	0,0472207	CMTM7
ENSGALG00000008597	7184,8669	0,404910294	0,1198878	3,37741	0,0007317	0,0041252	MRPL2
ENSGALG00000006505	3147,8767	0,40439474	0,1221546	3,310517	0,0009312	0,0050493	ZFAND6
ENSGALG00000014981	1640,2876	0,404127478	0,1524452	2,6509683	0,0080261	0,0295009	COX17
ENSGALG00000008143	14658,924	0,402789538	0,1063945	3,7858132	0,0001532	0,0010858	UACA
ENSGALG00000003924	3485,2604	0,402077112	0,1262253	3,1853918	0,0014456	0,0072359	ACBD6
ENSGALG00000027768	2765,0207	0,401592495	0,1379224	2,9117282	0,0035944	0,0152976	CTDSP1
ENSGALG00000028931	2027,2809	0,401212621	0,1470133	2,7290911	0,0063509	0,0243915	IGFBP4
ENSGALG00000008508	3096,6728	0,401158456	0,1203234	3,3340018	0,0008561	0,004694	PYGB
ENSGALG00000010704	15057,888	0,401049242	0,1064765	3,7665529	0,0001655	0,001164	GLCCI1
ENSGALG00000003371	6971,3556	0,400787786	0,1174349	3,4128513	0,0006429	0,0036962	ELL
ENSGALG00000003575	5543,8738	0,400668854	0,1362202	2,9413311	0,003268	0,0141048	DNTTIP1
ENSGALG00000026316	1590,1424	0,400010985	0,1398143	2,8610159	0,0042229	0,017455	TMEM136
ENSGALG00000001473	2324,7953	0,399774883	0,1339201	2,9851752	0,0028342	0,0125732	MIB2
ENSGALG00000000253	2196,4708	0,399568995	0,1345932	2,9687154	0,0029905	0,0131255	SLC25A46
ENSGALG00000001096	5395,305	0,399476875	0,1210243	3,3007994	0,0009641	0,0051841	AC005943.2
ENSGALG00000002492	1100,2646	0,398681223	0,1527657	2,6097562	0,0090607	0,0324549	H6PD

ENSGALG00000016191	3940,4435	0,398485008	0,1411098	2,8239358	0,0047438	0,0191937	PKNOX1
ENSGALG00000000972	1494,0966	0,398341588	0,1611855	2,4713244	0,0134614	0,0444414	PBX3
ENSGALG00000015961	15590,16	0,398310373	0,130734	3,0467236	0,0023135	0,0106334	SUCLG1
ENSGALG00000003412	4305,4895	0,398169298	0,118859	3,34993	0,0008083	0,0044792	RALGDS
ENSGALG00000016549	1882,0348	0,397970824	0,1562578	2,5468866	0,0108689	0,037606	CTPS2
ENSGALG00000004176	2961,1415	0,397874037	0,1416462	2,8089278	0,0049707	0,0199869	ATOX1
ENSGALG00000014970	13935,559	0,397421157	0,1074971	3,6970395	0,0002181	0,0014739	FSTL1
ENSGALG00000003422	9411,1693	0,397187449	0,1102449	3,6027755	0,0003148	0,0020238	TRIM3
ENSGALG00000017262	1782,0171	0,39670681	0,1365862	2,9044423	0,0036791	0,0156094	NARS2
ENSGALG00000006736	1555,2322	0,396360498	0,1559292	2,5419267	0,0110243	0,0380183	AEN
ENSGALG00000006889	2493,8401	0,396136143	0,1473921	2,6876356	0,007196	0,0270495	TMC7
ENSGALG00000014233	13243,506	0,394951392	0,1481986	2,6650135	0,0076985	0,0286146	FBLN1
ENSGALG00000005046	2539,1359	0,394497591	0,1359729	2,9012952	0,0037162	0,0157524	SHMT1
ENSGALG00000001572	4170,7383	0,394480266	0,1178093	3,3484648	0,0008126	0,0044938	COMMD4
ENSGALG00000028470	5093,1917	0,39443368	0,1241813	3,1762725	0,0014918	0,007402	GSR
ENSGALG00000005675	3085,819	0,394073439	0,1342631	2,9350829	0,0033346	0,014349	PTTG1IP
ENSGALG00000021047	9190,8891	0,393972396	0,122897	3,2057124	0,0013473	0,0068343	KIFAP3
ENSGALG00000008039	2411,3069	0,392392533	0,1551271	2,5294903	0,0114228	0,0390268	MFSD13A
ENSGALG00000008050	1623,9365	0,391124824	0,1411515	2,7709581	0,0055892	0,0219805	HBP1
ENSGALG00000013348	2256,8618	0,390928939	0,128295	3,0471095	0,0023105	0,0106241	OTUD7B
ENSGALG00000010490	10248,566	0,389494511	0,1117875	3,4842389	0,0004935	0,002968	DPYSL4
ENSGALG00000013000	7034,8497	0,388948819	0,1248778	3,1146355	0,0018417	0,008789	ADIPOR2
ENSGALG00000026095	1420,7135	0,388637494	0,1454616	2,6717534	0,0075456	0,0281076	CDC26
ENSGALG00000000955	5256,5616	0,388613492	0,1145617	3,3921753	0,0006934	0,0039501	TMEM222
ENSGALG00000011035	1343,9931	0,388589204	0,1474226	2,6358867	0,0083918	0,0305819	AK4
ENSGALG00000008386	1936,8714	0,388150893	0,1387904	2,7966699	0,0051632	0,0205937	KIZ
ENSGALG00000015311	2435,4616	0,387705603	0,1261741	3,0727833	0,0021207	0,009926	RNF38
ENSGALG00000014496	6279,2131	0,385917845	0,134402	2,8713694	0,004087	0,0169958	PROM1
ENSGALG00000004011	4239,1769	0,385892717	0,1224058	3,1525685	0,0016184	0,0079204	CAMKK2
ENSGALG00000005337	6236,1037	0,385644449	0,1096926	3,5156828	0,0004386	0,0026828	SNX2
ENSGALG00000004086	6417,7852	0,385423377	0,1245819	3,0937355	0,0019765	0,0093504	CHMP1B
ENSGALG00000026102	19802,423	0,385336727	0,121726	3,1656082	0,0015476	0,0076398	CHTOP
ENSGALG00000015898	1319,6724	0,384642657	0,1580612	2,4335051	0,0149534	0,0482633	MYO6
ENSGALG00000016639	3515,2869	0,384196385	0,1227968	3,1287174	0,0017557	0,0084616	KIF13B
ENSGALG00000026188	5641,8964	0,384179427	0,1152566	3,3332525	0,0008584	0,0047048	TMEM8A
ENSGALG00000008736	6339,8487	0,384117634	0,1174006	3,271854	0,0010684	0,0056493	PNPLA7
ENSGALG00000020005	6706,2183	0,383841955	0,130019	2,9521995	0,0031552	0,0137158	SFT2D1
ENSGALG00000014808	2216,3488	0,383214364	0,1540134	2,488189	0,0128395	0,0428667	USF3
ENSGALG00000001536	1337,5687	0,382405736	0,149925	2,5506477	0,0107523	0,0372615	ARHGAP39
ENSGALG00000028635	2927,7161	0,382285105	0,1328162	2,8783008	0,0039982	0,0166777	C9ORF58
ENSGALG00000000456	4419,0221	0,381123553	0,1256788	3,0325206	0,0024252	0,0110469	MRPS21
ENSGALG00000011325	5273,0258	0,380378399	0,1366737	2,7831135	0,005384	0,0213339	NDUFA12
ENSGALG00000008297	4909,6132	0,379216929	0,1148855	3,3008262	0,000964	0,0051841	SEMA4B
ENSGALG00000001166	2588,454	0,378691011	0,1266846	2,9892423	0,0027967	0,0124483	GATSL2
ENSGALG00000007205	14498,189	0,37857906	0,1049589	3,6069259	0,0003098	0,0019961	COX6A1
ENSGALG00000015958	3885,4269	0,378120956	0,1313335	2,8790889	0,0039883	0,0166514	EIF2AK3
ENSGALG00000028128	3864,6629	0,378077792	0,1172702	3,2239889	0,0012642	0,0064852	HOXD8
ENSGALG00000015436	20607,243	0,377556464	0,1126811	3,3506644	0,0008062	0,0044728	SDCBP
ENSGALG00000002053	9601,2632	0,376194926	0,1115908	3,3711997	0,0007484	0,0042037	POR
ENSGALG00000027416	1624,7836	0,375994995	0,1449326	2,5942749	0,0094791	0,0336613	ROMO1L
ENSGALG00000002959	3279,2685	0,375394519	0,1495524	2,5101204	0,012069	0,0407741	NRBF2
ENSGALG00000009948	1430,5348	0,375138393	0,1485226	2,5258008	0,0115435	0,0393305	HHIP
ENSGALG00000023180	1278,6895	0,374920803	0,1483185	2,527809	0,0114777	0,0391454	
ENSGALG00000004191	5196,6988	0,374652449	0,1167651	3,2086007	0,0013338	0,0067774	RIC8A
ENSGALG00000009959	1836,2989	0,374451686	0,1402648	2,6696054	0,007594	0,0282649	AC025263.3
ENSGALG00000003717	5316,7007	0,373543889	0,1130969	3,3028656	0,000957	0,0051565	MYO9B
ENSGALG00000004324	3684,0767	0,373249102	0,1339589	2,7862949	0,0053314	0,0211564	SETD1B
ENSGALG00000008438	6164,0602	0,373204358	0,1216092	3,0688816	0,0021486	0,0100141	SLC35C1
ENSGALG00000001032	4578,5026	0,373005757	0,1227121	3,039681	0,0023683	0,010842	STUB1
ENSGALG00000026291	5117,1597	0,37266418	0,1170472	3,1838789	0,0014532	0,0072578	CDH3
ENSGALG00000026421	20179,815	0,372144283	0,1169454	3,1822057	0,0014616	0,0072865	PSMB2
ENSGALG00000002990	14374,489	0,371847834	0,1302887	2,8540301	0,0043168	0,017756	PRELID1

ENSGALG00000002605	7879,732	0,371275034	0,1234991	3,006297	0,0026445	0,0118554	MRPL17
ENSGALG00000009073	2474,3956	0,370739827	0,1479952	2,5050805	0,0122424	0,0412267	PSEN2
ENSGALG00000004357	2772,1991	0,370669228	0,1314113	2,8206804	0,0047922	0,019355	ANXA6
ENSGALG00000016850	6183,7618	0,369402589	0,1221012	3,0253801	0,0024832	0,0112659	ANKRD10
ENSGALG00000015543	2204,9702	0,369304606	0,1292142	2,8580804	0,0042621	0,0175766	MRPL50
ENSGALG00000003185	3436,5941	0,368988195	0,1514261	2,4367543	0,0148197	0,0479038	SCPEP1
ENSGALG00000015537	3286,5424	0,368709498	0,1440858	2,5589581	0,0104986	0,036576	MAN2B2
ENSGALG00000006857	3516,2609	0,36847671	0,1191186	3,0933594	0,001979	0,0093558	SPATA20
ENSGALG00000026716	2067,7158	0,368315116	0,1476133	2,4951349	0,0125909	0,042223	CELF5
ENSGALG00000000747	4334,9927	0,36652476	0,1212386	3,0231688	0,0025014	0,0113184	CHD5
ENSGALG00000028249	8201,3974	0,366515679	0,1075014	3,409405	0,000651	0,0037354	MCL1
ENSGALG00000006149	3876,2934	0,366158451	0,1322855	2,7679406	0,0056412	0,0221403	MORN4
ENSGALG00000012952	17818,525	0,365044211	0,1286631	2,8372099	0,004551	0,0185125	BASP1
ENSGALG00000013848	3142,0736	0,364984572	0,1264318	2,8868109	0,0038917	0,0163434	MVK
ENSGALG00000010621	3304,8585	0,364976716	0,1292617	2,8235486	0,0047495	0,0192112	DPY30
ENSGALG00000015663	10480,988	0,363842291	0,1220245	2,9817148	0,0028664	0,0126913	HSDL2
ENSGALG00000001597	12155,403	0,36336647	0,135585	2,6799894	0,0073624	0,027546	PSMB3
ENSGALG00000013876	3474,876	0,361738997	0,1264822	2,8599989	0,0042364	0,0174997	FAM136A
ENSGALG00000021692	6544,9246	0,361385734	0,1165284	3,1012667	0,0019269	0,0091476	AC024592.12
ENSGALG00000016333	1977,9922	0,361282215	0,1426527	2,5326003	0,011322	0,0387796	MTSS1
ENSGALG00000005704	10751,887	0,361085404	0,1196869	3,0169164	0,0025536	0,0115009	RP11-411B6.6
ENSGALG00000006630	3807,9216	0,360923749	0,1347522	2,6784253	0,0073969	0,0276565	COMMD7
ENSGALG00000014589	16537,437	0,360345797	0,1177437	3,0604243	0,0022102	0,0102529	
ENSGALG00000007589	8938,4163	0,360072922	0,1085811	3,3161649	0,0009126	0,0049604	DEDD
ENSGALG00000011435	8069,4783	0,35897775	0,1123046	3,1964666	0,0013912	0,0070167	ELK3
ENSGALG00000010171	1427,5927	0,358902977	0,1475799	2,4319235	0,0150189	0,048421	NFKBIE
ENSGALG00000001330	11236,984	0,358718136	0,1146464	3,128908	0,0017546	0,0084616	ATP5G1
ENSGALG00000006143	4041,5216	0,358630949	0,1180875	3,0369922	0,0023895	0,0109172	RP11-548K23.11
ENSGALG00000002159	5312,4581	0,35820654	0,1168583	3,0653076	0,0021745	0,0101146	ACOX1
ENSGALG00000005478	5105,9851	0,35705086	0,1183988	3,015662	0,0025642	0,011541	SNX7
ENSGALG00000026531	11386,746	0,356957338	0,1206613	2,9583418	0,003093	0,0135058	CHST14
ENSGALG00000026517	6681,3961	0,355711572	0,1112537	3,1973011	0,0013872	0,0070029	RFC5
ENSGALG00000011176	5734,5779	0,354690121	0,1402295	2,5293538	0,0114273	0,0390322	SCRN1
ENSGALG00000003096	2680,9001	0,354610608	0,1443928	2,455874	0,0140542	0,045942	CBFA2T2
ENSGALG00000001425	2462,9176	0,354258416	0,1359645	2,6055206	0,0091735	0,0327727	SNX19
ENSGALG00000023073	19350,483	0,353608475	0,1057356	3,3442711	0,000825	0,004546	AKAP12
ENSGALG00000013036	6599,1108	0,353370495	0,1362318	2,5938909	0,0094897	0,0336858	ATP6V1E1
ENSGALG00000014891	4747,3286	0,353275878	0,1368587	2,5813185	0,0098424	0,034752	PELO
ENSGALG00000026600	7491,3546	0,352554713	0,1374236	2,5654603	0,0103039	0,0360174	
ENSGALG00000026565	5799,2794	0,352044819	0,1113845	3,1606258	0,0015743	0,007752	ENSA
ENSGALG00000007396	5182,898	0,351138505	0,1196447	2,9348426	0,0033372	0,0143556	TAOK3
ENSGALG00000012229	22179,4	0,350504617	0,1021787	3,430311	0,0006029	0,0035093	RPS29
ENSGALG00000007305	2042,815	0,350349865	0,1310633	2,6731358	0,0075146	0,0280151	NME1
ENSGALG00000000461	6717,843	0,348147161	0,1224667	2,8427896	0,0044721	0,0182515	MRC2
ENSGALG00000016241	5823,7161	0,347015145	0,1359999	2,5515842	0,0107234	0,0372068	ATP6AP2
ENSGALG00000001381	43057,795	0,34689365	0,1163519	2,9814171	0,0028692	0,0126954	ACTG1
ENSGALG00000010347	11028,081	0,346318448	0,1080262	3,2058747	0,0013465	0,0068343	CCNG2
ENSGALG00000005135	11151,897	0,345569481	0,1285407	2,688405	0,0071794	0,0270058	SERPINE2
ENSGALG00000026300	11402,689	0,345400821	0,1081778	3,1928993	0,0014085	0,0070895	
ENSGALG00000020922	2968,9637	0,345299483	0,1246827	2,769425	0,0056155	0,0220587	C1orf21
ENSGALG00000023447	10483,165	0,344836567	0,1046431	3,2953598	0,000983	0,0052585	RP11-343C2.11
ENSGALG00000013563	2902,2623	0,344320197	0,1287254	2,6748425	0,0074764	0,0278962	CCDC102A
ENSGALG00000002484	4545,8313	0,343583814	0,1190666	2,8856437	0,0039061	0,0163839	CRYM
ENSGALG00000003215	4360,1328	0,343135123	0,1166654	2,9411903	0,0032695	0,0141048	PEF1
ENSGALG00000016665	8909,5125	0,342839663	0,1328584	2,5804883	0,0098661	0,0347816	FDFT1
ENSGALG00000026203	4631,9513	0,341949081	0,1195864	2,8594311	0,004244	0,0175151	FAM174A
ENSGALG00000012586	2037,1791	0,341920905	0,1360582	2,5130482	0,0119693	0,0405176	GKAP1
ENSGALG00000003293	12978,607	0,341652813	0,1149964	2,9709875	0,0029684	0,0130583	MBTPS1
ENSGALG00000012770	2998,9246	0,340485203	0,1279524	2,6610294	0,0077902	0,028853	MAK
ENSGALG00000013784	13631,601	0,339967639	0,1079829	3,1483476	0,001642	0,0080213	CDT1
ENSGALG00000006837	2354,847	0,339965168	0,1390009	2,4457766	0,0144541	0,0469446	TSPAN4
ENSGALG00000006463	6778,1634	0,33979504	0,1341936	2,5321248	0,0113374	0,0388225	TMA7

ENSGALG00000000833	15942,313	0,339708342	0,1136284	2,9896436	0,002793	0,0124394	IK
ENSGALG00000015998	9692,1223	0,339150714	0,1058855	3,2029939	0,0013601	0,0068915	PTDSS1
ENSGALG00000006056	5221,8645	0,338801705	0,1296	2,6142105	0,0089434	0,0321363	CHCHD6
ENSGALG00000027609	5715,7167	0,338322071	0,1138079	2,9727469	0,0029515	0,0130039	RAB40C
ENSGALG00000024261	6604,4448	0,336614329	0,1086574	3,0979431	0,0019487	0,0092412	NHP2
ENSGALG00000001476	3822,0948	0,335505795	0,1349976	2,485273	0,0129452	0,0430927	RAP1A
ENSGALG00000001329	7967,7759	0,33542437	0,1280369	2,6197481	0,0087995	0,0317532	EPB41
ENSGALG00000012093	2809,4561	0,335251509	0,1300305	2,5782533	0,0099301	0,0349712	ELMO1
ENSGALG00000002157	23751,205	0,335162629	0,1132861	2,958549	0,0030909	0,013501	RPS17
ENSGALG00000011476	10900,501	0,334810497	0,1121934	2,9842264	0,002843	0,0125999	sept-11
ENSGALG00000012277	5198,3118	0,33449799	0,122213	2,7370074	0,0062001	0,0239052	MAFF
ENSGALG00000024033	5551,4467	0,334154631	0,1192958	2,8010587	0,0050935	0,0203689	PHLDB1
ENSGALG00000012312	5618,5193	0,333752483	0,1164047	2,8671731	0,0041416	0,017158	GCAT
ENSGALG00000007519	2801,5468	0,333421083	0,1296588	2,5715271	0,0101251	0,0355292	ACBD5
ENSGALG00000009234	2846,7839	0,332951253	0,1239542	2,6860825	0,0072295	0,0271531	RP11-20I23.3
ENSGALG00000006546	6021,0715	0,332225604	0,1201276	2,765605	0,0056817	0,0222545	CD81
ENSGALG00000003934	3446,0691	0,332093035	0,1296938	2,5605934	0,0104494	0,0364416	WIPF2
ENSGALG00000013529	4334,8333	0,331833816	0,1204748	2,7543838	0,0058803	0,02288	NUP210L
ENSGALG00000014236	4088,5792	0,331720827	0,1185166	2,7989389	0,0051271	0,0204647	ATP8A1
ENSGALG00000001761	3252,5626	0,33061823	0,1263585	2,6165091	0,0088834	0,0319713	HAUS1
ENSGALG00000000905	28204,583	0,330518761	0,1072649	3,0813327	0,0020608	0,0096783	PSMB4
ENSGALG00000000055	4657,6107	0,330211238	0,1218412	2,7101775	0,0067247	0,0256032	LAMTOR3
ENSGALG00000002967	2956,1697	0,329951632	0,1271164	2,5956654	0,0094408	0,0335537	ZNF365
ENSGALG00000008457	3707,8992	0,329256556	0,1280479	2,5713549	0,0101301	0,0355377	RIN2
ENSGALG00000015844	18562,8	0,329214954	0,1087925	3,0260806	0,0024775	0,0112473	SOD1
ENSGALG00000000629	2184,8298	0,329174801	0,1291076	2,5496155	0,0107842	0,0373494	
ENSGALG00000010109	8235,1722	0,328812909	0,1134692	2,8978173	0,0037577	0,0158984	RNF220
ENSGALG00000029052	4904,723	0,328574855	0,1134688	2,8957295	0,0037828	0,0159748	CERS2L1
ENSGALG00000003331	9717,1633	0,328559948	0,1145187	2,8690497	0,0041171	0,0171	FAF2
ENSGALG00000002919	3330,9646	0,328149978	0,1337566	2,4533361	0,0141538	0,0462009	MON1A
ENSGALG00000007272	8940,9306	0,327852784	0,1056422	3,1034251	0,0019129	0,0090938	RNF166
ENSGALG00000001604	7206,5279	0,327620067	0,1148758	2,8519514	0,0043452	0,0178353	
ENSGALG00000010441	3362,4409	0,327510367	0,1300487	2,5183664	0,0117901	0,0400403	GYG1
ENSGALG00000008966	9784,0345	0,327459196	0,1078303	3,0368019	0,002391	0,0109204	FBXW5
ENSGALG00000006439	2469,3771	0,325297261	0,1285361	2,5307849	0,0113808	0,0389417	ARHGEF6
ENSGALG00000004000	3131,3983	0,325159334	0,1227269	2,6494547	0,0080622	0,0295947	FLOT2
ENSGALG00000008925	2847,1933	0,32505073	0,1271866	2,555699	0,0105975	0,0368637	TMEM203
ENSGALG00000006161	2625,1209	0,324498322	0,1340451	2,4208145	0,0154858	0,0496386	CHMP1A
ENSGALG00000004899	7704,948	0,323966145	0,1311985	2,469283	0,0135384	0,0446201	PEPD
ENSGALG00000013039	15959,11	0,323619	0,1177645	2,7480188	0,0059957	0,0232434	BID
ENSGALG00000014555	6185,863	0,32349139	0,119778	2,7007582	0,0069182	0,0261637	C12orf57
ENSGALG00000015259	6306,083	0,322565394	0,1124418	2,8687313	0,0041212	0,0171086	RB1CC1
ENSGALG00000015828	4399,085	0,322397999	0,1225293	2,6311901	0,0085086	0,030896	BACH1
ENSGALG00000010985	3705,9823	0,321700662	0,1306157	2,462956	0,0137797	0,0451855	ATG4C
ENSGALG00000028499	5696,2423	0,321645303	0,1148381	2,8008589	0,0050967	0,0203732	FAM63A
ENSGALG00000013738	7162,7044	0,321523306	0,1318588	2,4383909	0,0147528	0,0477354	OPTN
ENSGALG00000025781	5021,0191	0,321200342	0,1135034	2,8298746	0,0046566	0,0188803	CERS2L2
ENSGALG00000028937	3780,2165	0,321152786	0,1241263	2,5873067	0,0096729	0,0342248	TIMM13
ENSGALG00000008443	36760,133	0,321038212	0,1010416	3,1772886	0,0014866	0,0073788	ARHGDIA
ENSGALG00000003513	5800,0504	0,320426255	0,1140656	2,8091408	0,0049674	0,0199796	KLHL20
ENSGALG00000014682	2661,1178	0,320335943	0,1298503	2,4669635	0,0136264	0,044845	CAST
ENSGALG00000005701	4780,9727	0,319914204	0,1181292	2,708173	0,0067655	0,0257069	IP6K2
ENSGALG00000016172	54051,231	0,319415143	0,1086593	2,9396034	0,0032863	0,0141682	RPLP1
ENSGALG00000012843	3927,8364	0,319142472	0,131197	2,4325431	0,0149932	0,0483612	GMDS
ENSGALG00000015664	24898,273	0,319058165	0,1003222	3,1803344	0,0014711	0,0073203	ADD1
ENSGALG00000012546	35449,688	0,318249627	0,099977	3,1832278	0,0014564	0,0072688	MCM5
ENSGALG00000011192	4976,6178	0,317451368	0,1192137	2,6628766	0,0077476	0,0287264	PHF10
ENSGALG00000002595	4724,8428	0,316793207	0,1219683	2,5973403	0,0093949	0,0334059	
ENSGALG00000011238	46142,463	0,316616268	0,1021796	3,0986245	0,0019442	0,0092232	WLS
ENSGALG00000013242	4048,7621	0,316427862	0,1207163	2,621252	0,0087607	0,0316302	MRPL24
ENSGALG00000002891	7363,8293	0,315737267	0,1107929	2,8497977	0,0043747	0,0179348	ACADL
ENSGALG00000023308	5464,7909	0,314498813	0,1190501	2,6417343	0,0082483	0,0301558	NPB

ENSGALG00000020078	34717,171	0,313921783	0,0988798	3,174781	0,0014995	0,007432	
ENSGALG00000028600	142500,09	0,313870865	0,0985585	3,1846146	0,0014495	0,00725	
ENSGALG00000011106	5264,4735	0,312738357	0,1159112	2,698085	0,006974	0,0263167	GALNT2
ENSGALG00000006880	22127,188	0,310525591	0,1109413	2,7990071	0,005126	0,0204647	SLC38A10
ENSGALG00000011958	2755,4733	0,30674184	0,1239537	2,4746489	0,0133367	0,0441477	TEF
ENSGALG00000008586	62912,793	0,306372777	0,1047411	2,9250486	0,003444	0,0147454	TUBB4B
ENSGALG00000003691	21366,038	0,306291187	0,1008897	3,0359009	0,0023982	0,0109384	RNF145
ENSGALG00000014190	4272,095	0,305976217	0,1248086	2,4515639	0,0142237	0,0463753	PNPLA3
ENSGALG00000003428	8918,8411	0,30596567	0,1074998	2,8461972	0,0044245	0,0181062	PIK3R2
ENSGALG00000010997	7028,5315	0,305349661	0,119889	2,5469371	0,0108673	0,037606	DFNA5
ENSGALG00000001024	11719,39	0,304587459	0,1095728	2,779773	0,0054397	0,0215357	TPRG1L
ENSGALG00000004898	4936,2049	0,304436049	0,1215502	2,5046118	0,0122586	0,0412712	
ENSGALG00000015273	5611,3118	0,304040992	0,1190879	2,5530798	0,0106775	0,0370663	TBC1D23
ENSGALG00000000606	4262,8792	0,302883764	0,1188851	2,5477019	0,0108435	0,0375373	ARL8A
ENSGALG00000002712	8556,0623	0,302868054	0,1070979	2,8279558	0,0046846	0,0189881	LIPG
ENSGALG00000014118	12062,787	0,302595823	0,1052318	2,8755167	0,0040337	0,0167998	MRPS26
ENSGALG00000000094	5913,4414	0,30200193	0,1190933	2,5358428	0,0112177	0,0385193	ADIPOR1
ENSGALG00000000970	7143,9739	0,300853659	0,1087984	2,7652385	0,0056881	0,0222705	CHEK1
ENSGALG00000006876	3198,9684	0,300817961	0,1240621	2,4247362	0,0153195	0,0491923	CTSA
ENSGALG00000015211	3091,6006	0,300524293	0,1216505	2,4703913	0,0134965	0,0445036	DTNA
ENSGALG00000004156	5252,3729	0,300005854	0,114727	2,6149543	0,0089239	0,0320918	KIF1BP
ENSGALG00000003973	3666,163	0,297790777	0,1193872	2,4943284	0,0126196	0,0422982	TLCD1
ENSGALG00000027838	4595,5664	0,29706177	0,1173947	2,5304523	0,0113916	0,0389591	HMG20B
ENSGALG00000002279	6077,2003	0,297008819	0,1136905	2,6124335	0,00899	0,0322612	CTD-2116N17.1
ENSGALG00000017249	12265,651	0,295667982	0,1084027	2,7274952	0,0063817	0,0244959	PCF11
ENSGALG00000009570	6991,744	0,29562858	0,1160829	2,5467029	0,0108746	0,0376163	ATP6V1D
ENSGALG00000001913	13013,108	0,29356182	0,1209326	2,4274828	0,015204	0,048879	DYNLRB1
ENSGALG00000000944	3984,6591	0,29296383	0,1166894	2,5106287	0,0120516	0,0407356	SUPT4H1
ENSGALG00000014408	16252,764	0,292920829	0,1032929	2,8358262	0,0045707	0,0185874	LLGL1
ENSGALG00000023975	11745,641	0,291584025	0,1045871	2,7879551	0,0053042	0,0210728	SLC25A39
ENSGALG00000028356	9980,9657	0,288424278	0,1104192	2,6120845	0,0089992	0,0322857	LMAN2
ENSGALG00000006904	13921,155	0,287723415	0,1047262	2,7473884	0,0060072	0,0232815	RNH1
ENSGALG00000008865	4561,3827	0,286783741	0,1175361	2,4399621	0,0146888	0,0475709	HDHD3
ENSGALG00000013341	5963,8498	0,285851588	0,116546	2,4526925	0,0141791	0,0462726	AGAP3
ENSGALG00000007250	25520,219	0,285601509	0,1086627	2,6283308	0,0085805	0,0311198	SNRPB
ENSGALG00000021242	6224,5764	0,284935514	0,1141595	2,4959426	0,0125623	0,0421581	PHPT1
ENSGALG00000004410	6738,2931	0,28304622	0,1082646	2,6143925	0,0089386	0,0321276	ANAPC16
ENSGALG00000006073	7325,56	0,281547021	0,1095092	2,5709901	0,0101408	0,0355602	NDUFAB1
ENSGALG00000009940	12012,29	0,281084158	0,1035397	2,7147474	0,0066326	0,0253092	SLC35E3
ENSGALG00000014800	8363,7154	0,280973212	0,1136279	2,4727492	0,0134078	0,0443399	ATP6V1A
ENSGALG00000014467	5734,7231	0,280861046	0,1140326	2,462989	0,0137784	0,0451855	COPS7A
ENSGALG00000006917	6209,9134	0,280478545	0,1125356	2,4923538	0,01269	0,0424526	CHKA
ENSGALG00000001026	9235,9335	0,279930511	0,1055048	2,65325	0,0079721	0,0293748	EPB41L1
ENSGALG00000007260	8065,1993	0,279432916	0,1094636	2,5527474	0,0106877	0,0370923	ZEB1
ENSGALG00000014004	8156,8334	0,27938572	0,1132363	2,4672797	0,0136144	0,0448342	EPB41L2
ENSGALG00000002546	9375,5325	0,275701764	0,1107787	2,4887613	0,0128189	0,0428188	COL5A1
ENSGALG00000005138	8773,7935	0,274876887	0,1065601	2,5795489	0,0098929	0,0348493	KCTD10
ENSGALG00000003625	7388,448	0,273140927	0,1127098	2,4233993	0,015376	0,0493388	NDUFS5
ENSGALG00000006611	7163,1018	0,272646564	0,1103641	2,4704288	0,0134951	0,0445036	KIF7
ENSGALG00000011916	19570,61	0,271939056	0,112004	2,4279397	0,0151849	0,048829	SREBF2
ENSGALG00000008149	20254,48	0,269265878	0,1016307	2,6494538	0,0080622	0,0295947	EWSR1
ENSGALG00000004639	22048,421	0,266827236	0,1006911	2,6499598	0,0080501	0,0295664	GNAI2
ENSGALG00000008609	46235,247	0,259800517	0,1027436	2,5286292	0,0114509	0,0390835	PTK7
ENSGALG00000015617	50870,723	0,25500812	0,1021977	2,4952431	0,0125871	0,0422205	RPS23
ENSGALG00000017299	53500,096	0,254780179	0,1021944	2,4930939	0,0126635	0,0424143	RPS11
ENSGALG00000011951	179564,91	0,254345179	0,1001273	2,5402178	0,0110783	0,0381466	RPSA
ENSGALG00000008812	67809,432	-0,244632027	0,100856	-2,425557	0,0152849	0,0491044	CCT4
ENSGALG00000008038	49840,281	-0,24989979	0,1024864	-2,438371	0,0147536	0,0477354	SF3B1
ENSGALG00000010027	20404,953	-0,251221533	0,1016636	-2,471106	0,0134696	0,0444515	CFL2
ENSGALG00000009228	73654,992	-0,251350219	0,0988688	-2,54226	0,0110138	0,0380109	PARP1
ENSGALG00000001053	21507,706	-0,251351424	0,101079	-2,486682	0,0128941	0,0429539	SRPRA
ENSGALG00000009309	84581,571	-0,253473054	0,1003203	-2,526638	0,011516	0,0392467	EIF5A2

ENSGALG00000028484	18420,667	-0,257706043	0,100811	-2,556328	0,0105783	0,036816	
ENSGALG00000029167	20948,612	-0,258969024	0,102918	-2,516265	0,0118606	0,0402497	THOP1
ENSGALG00000010842	26982,926	-0,259509696	0,1041078	-2,492703	0,0126775	0,0424402	UBR7
ENSGALG00000008944	11174,776	-0,259905745	0,1056639	-2,459741	0,0139037	0,0455156	FBXO11
ENSGALG00000004040	10377,603	-0,259935536	0,107237	-2,423935	0,0153534	0,0492777	MFAP3
ENSGALG00000015706	7834,195	-0,260107652	0,1070306	-2,430217	0,0150898	0,0485921	LETM1
ENSGALG00000008962	22921,574	-0,260994621	0,1007267	-2,591116	0,0095665	0,0338747	MSH2
ENSGALG00000004965	10149,59	-0,261538698	0,1058067	-2,471854	0,0134414	0,044408	SLC27A4
ENSGALG00000002569	85602,751	-0,262007268	0,1060299	-2,47107	0,013471	0,0444515	RAN
ENSGALG00000015596	26927,118	-0,262154887	0,1027805	-2,550629	0,0107529	0,0372615	MAP3K7
ENSGALG00000015821	61741,915	-0,262392566	0,1001176	-2,620842	0,0087713	0,0316599	CCT8
ENSGALG00000002488	22309,721	-0,262864461	0,100189	-2,623685	0,0086984	0,0314552	PAIP2
ENSGALG00000008180	11420,002	-0,263317561	0,1088104	-2,419968	0,0155219	0,0497248	SPG11
ENSGALG00000008500	8629,7439	-0,263668812	0,1056933	-2,494659	0,0126078	0,0422692	RBSN
ENSGALG00000012405	16329,496	-0,26384951	0,1069456	-2,467137	0,0136198	0,0448342	EZH2
ENSGALG00000001350	6586,6747	-0,264940499	0,1094289	-2,421119	0,0154728	0,0496142	RSPRY1
ENSGALG00000001515	15194,726	-0,265107192	0,1041998	-2,54422	0,0109522	0,0378366	ATAD3A
ENSGALG00000016042	8800,9648	-0,265273818	0,1060345	-2,501769	0,0123574	0,0415732	CHAF1B
ENSGALG00000000581	14682,192	-0,265993327	0,1094668	-2,4299	0,015103	0,0486232	DDX42
ENSGALG00000014330	13035,527	-0,266560386	0,1080798	-2,466329	0,0136506	0,0448931	NECAP1
ENSGALG00000011504	43957,582	-0,266735415	0,0983735	-2,711456	0,0066988	0,0255261	TMPO
ENSGALG00000016226	14374,267	-0,266804523	0,10248	-2,603479	0,0092283	0,0329512	CASK
ENSGALG00000009781	15826,657	-0,267258812	0,1074538	-2,487198	0,0128754	0,0429337	DOCK1
ENSGALG00000009941	12598,143	-0,268249202	0,1049288	-2,556488	0,0105735	0,0368085	SLC35B1
ENSGALG00000017118	10000,178	-0,268386575	0,1059335	-2,533537	0,0112918	0,0387248	MIPEP
ENSGALG00000011553	8178,0483	-0,268405007	0,1061309	-2,528999	0,0114389	0,0390619	UTP3
ENSGALG00000016417	8419,6417	-0,268534668	0,1066163	-2,518702	0,0117788	0,0400122	DSCC1
ENSGALG00000016894	12688,39	-0,268921468	0,1105656	-2,432233	0,015006	0,0483911	DNAJC3
ENSGALG00000005587	297190,08	-0,269468112	0,0978414	-2,754133	0,0058848	0,0228853	EIF4G2
ENSGALG00000026111	15113,012	-0,269770034	0,1052633	-2,562812	0,0103828	0,0362374	SAAL1
ENSGALG00000006113	19999,679	-0,272100604	0,1073343	-2,535075	0,0112423	0,0385844	ZNF326
ENSGALG00000028900	8664,7981	-0,272551766	0,1086758	-2,507935	0,0121439	0,0409661	
ENSGALG00000010284	17075,328	-0,272708241	0,1031857	-2,642887	0,0082202	0,0300614	GMPS
ENSGALG00000016953	7971,8105	-0,272857363	0,1064399	-2,563488	0,0103626	0,0361948	NAA16
ENSGALG00000012402	11643,369	-0,273460141	0,1041025	-2,626836	0,0086183	0,0312235	CUL1
ENSGALG00000005870	10634,139	-0,27364395	0,1062314	-2,575923	0,0099973	0,0351622	MCM10
ENSGALG00000014331	49106,781	-0,273875014	0,1046006	-2,618292	0,0088371	0,0318637	FKBP4
ENSGALG00000005654	13436,846	-0,27412231	0,1031825	-2,656674	0,0078916	0,0291333	PITPNB
ENSGALG00000000907	34606,894	-0,274397863	0,1051305	-2,610069	0,0090524	0,0324512	KARS
ENSGALG00000006974	21987,582	-0,275795791	0,1053463	-2,617992	0,0088449	0,0318788	DDX27
ENSGALG00000014439	23731,455	-0,275802443	0,1013775	-2,72055	0,0065173	0,024948	NCAPD2
ENSGALG00000012244	14183,266	-0,27619833	0,1043027	-2,648045	0,0080959	0,0296783	KLHDC2
ENSGALG00000011130	11036,561	-0,276443798	0,1084288	-2,549541	0,0107865	0,0373494	COPS4
ENSGALG00000007491	11685,784	-0,276864687	0,1090804	-2,538171	0,0111433	0,0383219	VWA9
ENSGALG00000000856	7768,1765	-0,277333067	0,1078018	-2,57262	0,0100932	0,0354264	EIF2D
ENSGALG00000017128	6812,3227	-0,278120188	0,1105176	-2,516523	0,0118519	0,0402303	SKA3
ENSGALG00000002179	5553,9071	-0,278228201	0,1141171	-2,438094	0,0147649	0,0477607	RP11-552F3.12
ENSGALG00000008394	17588,746	-0,278483321	0,1028235	-2,708363	0,0067616	0,0257069	THUMPD3
ENSGALG00000007712	6385,5881	-0,27852982	0,1114303	-2,499589	0,0124338	0,0417989	MYO1B
ENSGALG00000003532	126353,26	-0,278703141	0,1005665	-2,771332	0,0055828	0,0219743	DDX5
ENSGALG00000009774	8409,19	-0,279620047	0,1118848	-2,499177	0,0124482	0,0418268	ELF2
ENSGALG00000014425	11773,73	-0,280179605	0,1054976	-2,655791	0,0079123	0,0291781	NCAPG
ENSGALG00000016340	46321,016	-0,280763167	0,1135687	-2,472187	0,0134289	0,0443774	EIF2S3L
ENSGALG00000012798	32319,553	-0,280818753	0,1118437	-2,510814	0,0120453	0,0407243	SSR1
ENSGALG00000016447	53614,037	-0,281195284	0,1001808	-2,806878	0,0050024	0,0200672	PDIA6
ENSGALG00000023682	6598,1149	-0,28149273	0,1105533	-2,546216	0,0108898	0,0376475	GTF2H3
ENSGALG00000026390	10622,058	-0,281499002	0,1082118	-2,60137	0,0092852	0,0330938	BAHD1
ENSGALG00000011762	7368,3608	-0,281679323	0,1102252	-2,55549	0,0106038	0,03687	UBA6
ENSGALG00000000726	16204,608	-0,284722338	0,1131864	-2,515517	0,0118858	0,0403051	ELAVL1
ENSGALG00000003248	40887,68	-0,284935192	0,0989747	-2,878868	0,003991	0,0166579	SUB1
ENSGALG00000011171	7789,5801	-0,285220301	0,1097597	-2,59859	0,0093608	0,0333194	TBP
ENSGALG00000015285	36051,228	-0,286253589	0,1140443	-2,510021	0,0120724	0,0407754	TOMM70

ENSGALG00000014773	11518,187	-0,287175691	0,1109758	-2,587732	0,009661	0,0342004	ERBB2IP
ENSGALG00000008086	20675,956	-0,2873629	0,1034267	-2,778421	0,0054624	0,021613	ACTR1A
ENSGALG00000026761	29796,866	-0,287544813	0,1163484	-2,471412	0,0134581	0,0444413	LIN28B
ENSGALG00000007101	5189,3303	-0,287687091	0,1145161	-2,512197	0,0119982	0,0406054	ZPR1
ENSGALG00000000823	24312,395	-0,287692196	0,1169353	-2,460268	0,0138833	0,0454597	BAZ1B
ENSGALG00000001219	4265,8107	-0,287809408	0,1164176	-2,472216	0,0134278	0,0443774	PIGU
ENSGALG00000010925	7979,9749	-0,288036245	0,1081614	-2,663022	0,0077442	0,0287218	FAM126A
ENSGALG00000005302	10087,37	-0,288116362	0,105057	-2,742476	0,0060978	0,0235755	SASS6
ENSGALG00000016238	8227,2702	-0,288804451	0,1172255	-2,463666	0,0137524	0,0451396	MED14
ENSGALG00000001277	6953,8461	-0,28888441	0,1159131	-2,492249	0,0126937	0,0424526	STAG1
ENSGALG00000003086	11675,474	-0,289022537	0,1042361	-2,772768	0,0055582	0,0219029	EXOSC10
ENSGALG00000010829	17151,788	-0,289288545	0,1076078	-2,68836	0,0071804	0,0270058	CDCA7
ENSGALG00000014570	6323,6704	-0,289387372	0,1174534	-2,463847	0,0137455	0,0451277	LPCAT3
ENSGALG00000010318	35384,519	-0,289579204	0,1116994	-2,592487	0,0095285	0,0337839	TMED10
ENSGALG00000015804	7311,6424	-0,289600409	0,1113075	-2,601805	0,0092734	0,0330778	LTN1
ENSGALG00000003355	8990,1272	-0,289601526	0,1161151	-2,49409	0,0126281	0,0423163	NOP16
ENSGALG00000008095	17234,199	-0,289644341	0,113924	-2,542434	0,0110083	0,0380016	KIF23
ENSGALG00000005769	9850,6081	-0,289680709	0,1121661	-2,582606	0,0098057	0,0346586	WEE1
ENSGALG00000010167	23334,849	-0,289818191	0,1045631	-2,771707	0,0055763	0,0219585	PNN
ENSGALG00000003194	7179,1586	-0,290381921	0,1074323	-2,702929	0,0068731	0,0260208	ATP6V0A2
ENSGALG00000011304	6781,2258	-0,290613007	0,1160055	-2,505165	0,0122394	0,0412267	NGLY1
ENSGALG00000005858	11910,812	-0,290886069	0,1040132	-2,796626	0,0051639	0,0205937	DR1
ENSGALG00000006706	5131,464	-0,290887314	0,1158194	-2,511558	0,0120199	0,0406486	USP6NL
ENSGALG00000013369	5721,3826	-0,290969648	0,1118474	-2,601488	0,009282	0,0330933	NCLN
ENSGALG00000001443	24266,064	-0,291025391	0,1058054	-2,750573	0,0059491	0,0231222	PTPRU
ENSGALG00000014854	16295,817	-0,291513975	0,1167727	-2,496423	0,0125453	0,0421114	MYL12A
ENSGALG00000006934	7206,0363	-0,291528022	0,1149418	-2,536309	0,0112028	0,0384778	PATZ1
ENSGALG00000007254	3754,7945	-0,291530428	0,1205128	-2,419082	0,0155597	0,0498226	ARHGAP12
ENSGALG00000006884	8596,5657	-0,291624168	0,113545	-2,568359	0,0102181	0,0357819	mars-05
ENSGALG00000015120	9551,3619	-0,291739099	0,116855	-2,49659	0,0125394	0,042102	QTRT2
ENSGALG00000001438	13509,485	-0,292098905	0,1078016	-2,709598	0,0067365	0,0256277	WDR77
ENSGALG00000015483	16683,55	-0,292201948	0,1099352	-2,657946	0,0078618	0,029063	PNISR
ENSGALG00000002341	9767,8137	-0,292212762	0,1068419	-2,735002	0,006238	0,0240255	AP3S1
ENSGALG00000001733	10762,095	-0,292618206	0,1060051	-2,760415	0,0057728	0,0225205	LRIG2
ENSGALG00000015933	13856,149	-0,292842928	0,1142604	-2,562944	0,0103789	0,036237	PAXBP1
ENSGALG00000007516	13969,274	-0,293041961	0,1052871	-2,783265	0,0053815	0,0213301	RP11-449H3.3
ENSGALG00000002136	3591,7129	-0,293341559	0,1202128	-2,440185	0,0146797	0,0475641	AP3B2
ENSGALG00000016106	14612,023	-0,293596343	0,1087124	-2,700671	0,00692	0,0261637	NUDCD1
ENSGALG00000013424	3938,6768	-0,293681861	0,1183319	-2,481848	0,0130703	0,0434666	TULP3
ENSGALG00000008529	7562,5902	-0,293829308	0,1095259	-2,682737	0,0073022	0,0273583	FASTKD2
ENSGALG00000001175	5051,078	-0,294210197	0,1151299	-2,555462	0,0106047	0,03687	C4BPA
ENSGALG00000000890	14838,887	-0,29430607	0,1074987	-2,737765	0,0061858	0,0238651	SRPK1
ENSGALG00000001503	12542,871	-0,294569539	0,1129054	-2,608993	0,0090809	0,0325017	TBCD
ENSGALG00000005927	7896,7145	-0,295114532	0,1102438	-2,676927	0,0074301	0,0277608	ST5
ENSGALG00000016006	15900,752	-0,295193317	0,10687	-2,762171	0,0057418	0,0224255	ITSN1
ENSGALG00000011392	3156,1633	-0,295432379	0,1200153	-2,461624	0,013831	0,045321	MSH4
ENSGALG00000001301	23007,802	-0,295608227	0,1064113	-2,777977	0,0054699	0,0216363	CLTCL1
ENSGALG00000003262	4105,2449	-0,296160558	0,1196838	-2,474525	0,0133413	0,0441513	VAMP4
ENSGALG00000002134	7387,1514	-0,296314687	0,1196419	-2,47668	0,0132611	0,0439509	THUMPD1
ENSGALG00000016270	5777,0167	-0,296663159	0,109934	-2,698557	0,0069641	0,0262954	EFR3A
ENSGALG00000002670	23872,138	-0,297199956	0,1006311	-2,953362	0,0031433	0,0136686	ZC3H15
ENSGALG00000008094	100007,59	-0,297620991	0,1010893	-2,94414	0,0032385	0,0140066	HSPD1
ENSGALG00000010787	8044,4352	-0,297642358	0,1129413	-2,635371	0,0084045	0,030612	TTC4
ENSGALG00000016203	5413,6009	-0,297788719	0,1132631	-2,629177	0,0085592	0,0310507	KHDRBS3
ENSGALG00000017134	4848,4656	-0,298805127	0,1142181	-2,616093	0,0088942	0,0320019	IFT88
ENSGALG00000023703	6517,2936	-0,298876557	0,1228202	-2,433448	0,0149558	0,0482633	GIT1
ENSGALG00000006646	12074,844	-0,299111546	0,108755	-2,750324	0,0059536	0,0231332	ABCC1
ENSGALG00000008325	37371,278	-0,299659865	0,1093847	-2,739505	0,0061532	0,0237661	CKAP5
ENSGALG00000011445	6835,5845	-0,299863462	0,1227479	-2,442921	0,0145689	0,0472501	AAMP
ENSGALG00000013382	5584,8468	-0,299878268	0,1168395	-2,566582	0,0102706	0,0359195	SCO1
ENSGALG00000012561	14201,494	-0,299998657	0,1061635	-2,825817	0,004716	0,0190927	PKP4
ENSGALG00000010120	15856,807	-0,300036521	0,1036618	-2,894379	0,0037991	0,0160188	KIF2C

ENSGALG00000010818	4623,916	-0,300127816	0,1207061	-2,486435	0,012903	0,0429732	PLPP3
ENSGALG00000010619	3449,0695	-0,300249899	0,1235308	-2,430566	0,0150752	0,0485568	CC2D1B
ENSGALG00000001964	6474,3266	-0,300692826	0,114042	-2,636685	0,0083721	0,0305182	INPP5E
ENSGALG00000014370	5230,6919	-0,300941728	0,1119644	-2,687833	0,0071917	0,027041	SEL1L3
ENSGALG00000010275	8613,432	-0,301228493	0,1133946	-2,656462	0,0078965	0,0291438	FCF1
ENSGALG00000015611	5412,5223	-0,301903565	0,1149449	-2,626507	0,0086266	0,0312454	CASP8AP2
ENSGALG00000007351	8585,3707	-0,301983	0,1078117	-2,801023	0,0050941	0,0203689	MTPAP
ENSGALG00000014248	12329,033	-0,30224682	0,1037621	-2,912883	0,0035811	0,0152603	TMEM33
ENSGALG00000011301	5632,1725	-0,302449789	0,1176772	-2,570164	0,010165	0,0356142	LRRC40
ENSGALG00000005786	13742,557	-0,302474038	0,1235613	-2,447967	0,0143665	0,046727	DNTTIP2
ENSGALG00000016872	5074,8717	-0,30261365	0,1222955	-2,474446	0,0133443	0,0441513	PCCA
ENSGALG00000009990	5003,3757	-0,302651446	0,112748	-2,684317	0,0072678	0,0272594	SRBD1
ENSGALG00000005054	41673,988	-0,303560897	0,1093589	-2,775822	0,0055062	0,0217675	DKC1
ENSGALG00000005525	43269,502	-0,303682785	0,1060484	-2,863624	0,0041882	0,0173217	SRSF1
ENSGALG00000013573	4217,1955	-0,303884724	0,1221979	-2,486825	0,0128889	0,0429494	ALG2
ENSGALG00000020454	8238,8869	-0,304067404	0,1241364	-2,449462	0,014307	0,0465892	C5H14ORF169
ENSGALG00000001726	4862,9398	-0,304268782	0,1143657	-2,660491	0,0078027	0,0288914	NUDCD2
ENSGALG00000007102	6521,5758	-0,304336302	0,1232551	-2,469158	0,0135432	0,0446249	MLEC
ENSGALG00000000627	7600,7154	-0,305117543	0,1084461	-2,81354	0,0048999	0,019755	DNAJC11
ENSGALG00000026838	4227,5246	-0,305207931	0,1214042	-2,513982	0,0119377	0,0404206	MDFIC
ENSGALG00000003052	7714,0905	-0,30523634	0,1156088	-2,640252	0,0082844	0,0302474	AGFG1
ENSGALG00000006418	20583,346	-0,305254291	0,1100474	-2,773843	0,0055398	0,0218623	PDPK1
ENSGALG00000015013	17595,102	-0,305329384	0,1094445	-2,789809	0,0052739	0,0209647	MSX1
ENSGALG00000006488	10099,124	-0,305368261	0,111059	-2,749605	0,0059667	0,0231774	RYK
ENSGALG00000009856	9272,2302	-0,305500873	0,1237141	-2,46941	0,0135336	0,044615	GNS
ENSGALG00000001007	27355,205	-0,305959563	0,1072059	-2,853942	0,004318	0,017756	MMP15
ENSGALG00000015465	4968,7172	-0,305990751	0,1148791	-2,66359	0,0077312	0,0286811	ASCC3
ENSGALG00000001267	9421,3215	-0,306647481	0,1081863	-2,83444	0,0045906	0,0186403	MAP2K2
ENSGALG00000013064	12429,63	-0,306789618	0,1032068	-2,972572	0,0029532	0,0130039	NSUN2
ENSGALG00000010042	7954,106	-0,30721043	0,1081752	-2,839935	0,0045123	0,0183881	CNOT2
ENSGALG00000007307	30782,066	-0,307584455	0,1030681	-2,984284	0,0028424	0,0125999	USP7
ENSGALG00000015331	7117,5258	-0,308171718	0,1237599	-2,490077	0,0127715	0,0426815	GNE
ENSGALG00000012109	33178,661	-0,308358257	0,1122444	-2,747204	0,0060106	0,023288	EIF3M
ENSGALG00000017073	7452,5062	-0,308364987	0,1160232	-2,657786	0,0078656	0,0290689	BRCA2
ENSGALG00000006445	15242,685	-0,308553523	0,1035516	-2,979708	0,0028852	0,012754	ARNT2
ENSGALG00000003373	12183,365	-0,309311012	0,1178882	-2,623766	0,0086964	0,0314552	BRIX1
ENSGALG00000000990	39372,44	-0,309348416	0,12727	-2,430646	0,0150719	0,0485568	SRSF6
ENSGALG00000012936	8859,5776	-0,309500805	0,1175868	-2,632105	0,0084858	0,0308336	KIAA1551
ENSGALG00000020164	4566,1787	-0,31008137	0,1239655	-2,501353	0,012372	0,0416118	GAR1
ENSGALG00000001504	8296,3292	-0,31053349	0,1111444	-2,793964	0,0052066	0,0207336	DDX20
ENSGALG00000010961	89733,227	-0,310634298	0,100957	-3,076896	0,0020917	0,0098032	IGF2BP3
ENSGALG00000008211	5098,0335	-0,311240889	0,1149304	-2,708081	0,0067673	0,0257069	ACP2
ENSGALG00000003815	34063,921	-0,31144656	0,1009753	-3,084385	0,0020397	0,0095994	RPN2
ENSGALG00000007905	37631,106	-0,311643664	0,1126417	-2,766681	0,005663	0,0222004	LAMB1
ENSGALG00000016267	13457,673	-0,312264026	0,115716	-2,698538	0,0069645	0,0262954	PHF3
ENSGALG00000010344	30762,544	-0,312514856	0,1018116	-3,069541	0,0021439	0,0100027	DHX36
ENSGALG00000001848	13173,32	-0,312572754	0,1053696	-2,96644	0,0030127	0,0132102	UBE2J2
ENSGALG00000002106	4264,2615	-0,312942638	0,1236027	-2,531844	0,0113465	0,0388341	CLCC1
ENSGALG00000013462	2483,9812	-0,3130983	0,1273973	-2,457652	0,0139849	0,0457372	STX17
ENSGALG00000008714	4292,5321	-0,313343892	0,1254822	-2,497119	0,0125207	0,04206	ABI2
ENSGALG00000011797	18609,134	-0,313395866	0,1178867	-2,65845	0,0078501	0,0290274	MTHFD1
ENSGALG00000007089	17474,95	-0,313684689	0,1041053	-3,013149	0,0025855	0,0116254	KDELR2
ENSGALG00000005647	9129,0785	-0,313707238	0,1081392	-2,900958	0,0037202	0,0157595	ABCD3
ENSGALG00000016736	23809,889	-0,314343681	0,1027519	-3,05925	0,0022189	0,0102792	CYFIP1
ENSGALG00000001973	14919,644	-0,314418391	0,103095	-3,049793	0,00229	0,0105372	GNL2
ENSGALG00000007410	10084,856	-0,314498411	0,1237962	-2,540453	0,0110709	0,0381306	IGDCC4
ENSGALG00000012827	3045,2609	-0,314591513	0,12464	-2,524001	0,0116028	0,0394908	RIPK1
ENSGALG00000016072	4693,3951	-0,314891598	0,1172976	-2,684553	0,0072627	0,0272551	SLC25A32
ENSGALG00000003419	3186,2022	-0,314999563	0,1269361	-2,481561	0,0130808	0,0434804	MPHOSPH9
ENSGALG00000016893	6244,0698	-0,315224782	0,1174264	-2,684447	0,007265	0,0272563	UGGT2
ENSGALG00000006356	8494,9234	-0,315726233	0,1090464	-2,895338	0,0037875	0,0159897	MKLN1
ENSGALG00000013174	3418,9295	-0,315789129	0,1225684	-2,576431	0,0099826	0,0351244	RECQL

ENSGALG00000003948	5405,0125	-0,315917308	0,1189483	-2,65592	0,0079092	0,0291748	ALAS1
ENSGALG00000010115	2409,6668	-0,316246717	0,1295797	-2,440558	0,0146646	0,0475264	SCLT1
ENSGALG00000009305	39225,132	-0,31625936	0,1073859	-2,945072	0,0032288	0,0139777	LBR
ENSGALG00000014378	6758,1006	-0,3165666	0,1112178	-2,846367	0,0044221	0,018102	ANAPC4
ENSGALG00000025996	45890,797	-0,316767046	0,1146038	-2,76402	0,0057094	0,0223244	MKI67
ENSGALG00000009438	13859,101	-0,316803968	0,1103871	-2,869936	0,0041056	0,0170626	SLC39A9
ENSGALG00000002158	4001,6826	-0,31684048	0,1201134	-2,637844	0,0083435	0,0304223	GOLGA3
ENSGALG00000004375	15903,904	-0,317187722	0,1029166	-3,081989	0,0020562	0,0096636	VPS35
ENSGALG00000005518	5338,317	-0,317368673	0,1224356	-2,592127	0,0095385	0,0338105	ADRBK2
ENSGALG00000005567	4701,7613	-0,317423466	0,1173805	-2,704227	0,0068464	0,0259502	
ENSGALG00000016056	26843,51	-0,317775278	0,1063633	-2,98764	0,0028114	0,0124926	AZIN1
ENSGALG00000003753	2677,8795	-0,317987251	0,1281857	-2,480677	0,0131133	0,0435671	CYLD
ENSGALG00000028935	5415,5214	-0,318166766	0,1109111	-2,868666	0,0041221	0,0171086	BCL10
ENSGALG00000016243	13413,478	-0,318335491	0,1120682	-2,840551	0,0045036	0,0183581	PHF20L1
ENSGALG00000006525	5626,0083	-0,318337533	0,1236191	-2,575147	0,0100197	0,035223	NCAPG2
ENSGALG00000008045	3399,549	-0,318998276	0,1242709	-2,566959	0,0102595	0,0359082	SLC9A8
ENSGALG00000010748	12682,351	-0,319116199	0,1031995	-3,092226	0,0019866	0,0093851	EXO1
ENSGALG00000006861	3243,9283	-0,319421528	0,1239662	-2,576682	0,0099754	0,0351124	COQ7
ENSGALG00000006400	5333,2312	-0,319435266	0,1204467	-2,652088	0,0079996	0,0294363	LMBR1
ENSGALG00000004283	4377,1001	-0,319446156	0,1200243	-2,661512	0,007779	0,0288274	GCOM1
ENSGALG00000009854	6064,675	-0,319466559	0,1106787	-2,886432	0,0038964	0,016358	NEK2
ENSGALG00000003294	7834,3924	-0,31979622	0,1079493	-2,962467	0,0030518	0,0133647	ATAD5
ENSGALG00000010312	8288,8613	-0,319830197	0,1182081	-2,705655	0,006817	0,0258605	PLCH1
ENSGALG00000000847	5954,7792	-0,320140743	0,1166802	-2,743746	0,0060743	0,0234946	GARNL3
ENSGALG00000015983	15093,436	-0,320176555	0,1272555	-2,516013	0,0118691	0,0402685	GART
ENSGALG00000012160	7671,7979	-0,320265774	0,1148045	-2,789663	0,0052763	0,0209681	KIF18A
ENSGALG00000005139	53456,399	-0,320485908	0,1129243	-2,838059	0,0045389	0,0184744	CLTC
ENSGALG00000011568	15058,649	-0,320702144	0,1071903	-2,991896	0,0027725	0,0123601	GRSF1
ENSGALG00000010677	4086,7271	-0,320970598	0,1263152	-2,54103	0,0110526	0,0380966	NRDE2
ENSGALG00000005367	6338,6685	-0,320977764	0,116103	-2,764594	0,0056994	0,022298	MRPS22
ENSGALG00000004197	7479,7717	-0,321070935	0,1089802	-2,94614	0,0032177	0,0139429	SUPV3L1
ENSGALG00000005668	17427,249	-0,321274618	0,102142	-3,145373	0,0016588	0,0080714	WSB1
ENSGALG00000001817	20809,427	-0,321828859	0,1040048	-3,094366	0,0019723	0,0093338	CTCF
ENSGALG00000000235	25074,326	-0,321994098	0,1026312	-3,13739	0,0017046	0,008262	PPP2R2A
ENSGALG00000016496	7694,8086	-0,322028168	0,1163281	-2,768276	0,0056354	0,0221302	ATAD2B
ENSGALG00000007021	10708,327	-0,322236639	0,122335	-2,634052	0,0084373	0,0307066	PCNT
ENSGALG00000010178	15181,256	-0,322485727	0,103283	-3,122351	0,0017941	0,0086102	PUM3
ENSGALG00000012415	30254,104	-0,322526711	0,1060731	-3,040608	0,002361	0,010816	PDIA4
ENSGALG00000010629	6077,087	-0,322714728	0,1236984	-2,608883	0,0090838	0,0325036	ZCCHC11
ENSGALG00000015420	5618,1384	-0,322913162	0,1200773	-2,689211	0,0071621	0,0269519	MINA
ENSGALG00000016312	24555,619	-0,323062498	0,1115176	-2,896963	0,0037679	0,0159318	POLA1
ENSGALG00000027524	18598,963	-0,323112776	0,1191737	-2,711276	0,0067025	0,0255328	TNKS1BP1
ENSGALG00000004905	15958,906	-0,323232118	0,1254595	-2,576385	0,0099839	0,0351244	MYEF2
ENSGALG00000026825	4552,9877	-0,323431285	0,117936	-2,742431	0,0060986	0,0235755	CENPL
ENSGALG00000005758	19288,831	-0,323694502	0,1027713	-3,149659	0,0016346	0,007994	SPPL2A
ENSGALG00000012795	9238,0512	-0,32399695	0,115695	-2,80044	0,0051033	0,0203938	RIOK1
ENSGALG00000006477	10679,273	-0,324083678	0,1040257	-3,11542	0,0018368	0,0087718	DNAJB6
ENSGALG00000001225	6585,9552	-0,324115689	0,1122468	-2,887527	0,0038828	0,0163183	RABGAP1
ENSGALG00000002248	6999,8386	-0,324167266	0,1095291	-2,959644	0,0030799	0,0134575	NAB1
ENSGALG00000004468	4954,5803	-0,324300015	0,1240229	-2,61484	0,0089269	0,0320941	RMDN3
ENSGALG00000001774	27937,325	-0,324482091	0,1102622	-2,942822	0,0032524	0,0140619	NEO1
ENSGALG00000014740	9788,9965	-0,324585149	0,1200564	-2,703606	0,0068592	0,0259771	IPO11
ENSGALG00000002222	13238,295	-0,324594377	0,102741	-3,159348	0,0015812	0,0077775	NOC2L
ENSGALG00000001691	7796,647	-0,324650287	0,1188991	-2,73047	0,0063244	0,0243034	CPSF3L
ENSGALG00000016630	14201,696	-0,324650816	0,1156607	-2,806924	0,0050017	0,0200672	EXTL3
ENSGALG00000008963	9612,6755	-0,324767208	0,1203182	-2,699236	0,0069499	0,0262549	DBF4
ENSGALG00000016383	4080,3919	-0,324853698	0,1238239	-2,623513	0,0087028	0,0314627	TBC1D31
ENSGALG00000000814	43324,255	-0,325022823	0,0992306	-3,275429	0,001055	0,0055891	HNRNPR
ENSGALG00000014575	10529,411	-0,325103377	0,1055361	-3,080496	0,0020666	0,0096988	SEPHS1
ENSGALG00000010228	4674,7787	-0,325552019	0,1143948	-2,845863	0,0044291	0,0181143	TOE1
ENSGALG00000017098	6011,3801	-0,325598928	0,1104524	-2,947867	0,0031997	0,0138873	GTF3A
ENSGALG00000013626	6229,0015	-0,325639127	0,1131349	-2,878325	0,0039979	0,0166777	GMNN

ENSGALG00000011251	4075,2982	-0,326088701	0,1204829	-2,706514	0,0067994	0,025808	TBC1D5
ENSGALG00000004780	17002,116	-0,326184617	0,1040438	-3,135071	0,0017181	0,0083217	NAA25
ENSGALG00000006512	270063,95	-0,326293391	0,1225651	-2,662205	0,0077631	0,028776	HSPA8
ENSGALG00000017200	4010,3886	-0,326558761	0,134864	-2,421394	0,0154611	0,0495885	MTMR2
ENSGALG00000003303	8501,0081	-0,326575058	0,1195862	-2,730875	0,0063166	0,024288	IDH3A
ENSGALG00000012986	12589,688	-0,326734663	0,1169574	-2,793621	0,0052122	0,0207496	KDM5A
ENSGALG00000011091	12877,91	-0,326829517	0,1036675	-3,152671	0,0016178	0,0079204	NUP133
ENSGALG00000010185	21490,623	-0,326946921	0,1096738	-2,981085	0,0028723	0,0127051	HSPA4L
ENSGALG00000004263	8041,9782	-0,32716572	0,1124664	-2,909008	0,0036258	0,0154169	MYDGF
ENSGALG00000001386	15479,004	-0,32719689	0,1250442	-2,61665	0,0088797	0,0319666	PI4KA
ENSGALG00000015118	7147,4862	-0,327224219	0,1258447	-2,600222	0,0093163	0,0331874	SMC5
ENSGALG00000004691	6193,4106	-0,327404992	0,1145398	-2,85844	0,0042573	0,0175646	NLE1
ENSGALG00000001262	15946,322	-0,32743844	0,1065971	-3,07174	0,0021282	0,0099471	CLIC4
ENSGALG00000003624	5974,0185	-0,32749199	0,132372	-2,474028	0,0133599	0,0441923	KLHL10
ENSGALG00000021128	30791,423	-0,32759269	0,1148555	-2,852217	0,0043415	0,0178311	FSCN1
ENSGALG00000004161	24484,078	-0,327725215	0,1031907	-3,175919	0,0014936	0,0074083	CCNB2
ENSGALG00000012192	7395,3976	-0,328017116	0,1315753	-2,492999	0,0126669	0,0424152	NT5C3A
ENSGALG00000015367	2777,8919	-0,328321788	0,124308	-2,641195	0,0082614	0,0301957	PVRL3
ENSGALG00000009944	17813,767	-0,328405469	0,1120225	-2,931602	0,0033722	0,0144834	MYST2
ENSGALG00000006829	14523,345	-0,328581471	0,1344576	-2,443756	0,0145352	0,0471746	SMG1
ENSGALG00000014286	15937,559	-0,329230926	0,1063546	-3,095595	0,0019642	0,0093017	PDS5A
ENSGALG00000015334	2718,7701	-0,329290061	0,1296871	-2,539112	0,0111134	0,0382415	ZBTB11
ENSGALG00000027091	4014,8809	-0,329390279	0,1266481	-2,60083	0,0092998	0,0331373	GPR27
ENSGALG00000013962	7389,8352	-0,329646116	0,1086383	-3,034345	0,0024106	0,0109913	HBS1L
ENSGALG00000007603	19301,424	-0,329941075	0,1204714	-2,738749	0,0061673	0,0238124	RRP12
ENSGALG00000002062	16195,779	-0,329944458	0,105102	-3,139279	0,0016936	0,0082195	SRP68
ENSGALG00000001962	54240,973	-0,330389801	0,103799	-3,182976	0,0014577	0,0072725	PTBP1
ENSGALG00000001283	38879,748	-0,330483919	0,1158085	-2,853711	0,0043212	0,0177582	SRRM1
ENSGALG00000010576	13964,899	-0,330632554	0,1026092	-3,22225	0,0012719	0,0065198	GTF2A1
ENSGALG00000010481	12612,575	-0,330642085	0,1059113	-3,121879	0,001797	0,008621	CMPK1
ENSGALG00000028637	4067,4699	-0,330733659	0,135202	-2,446219	0,0144363	0,0468982	PTPN23
ENSGALG00000010341	71633,337	-0,33081711	0,1033707	-3,2003	0,0013728	0,0069433	XPO5
ENSGALG00000015540	16673,279	-0,330835071	0,1205533	-2,744306	0,0060639	0,0234679	RAD23B
ENSGALG00000007387	14223,805	-0,330955786	0,1065147	-3,107138	0,0018891	0,0089867	GTF3C1
ENSGALG00000021683	8310,9409	-0,330974467	0,1292911	-2,559917	0,0104697	0,0364939	RER1
ENSGALG00000003547	13605,07	-0,331189406	0,1062178	-3,118021	0,0018207	0,0087131	SKP2
ENSGALG00000002889	12683,471	-0,331591263	0,1090874	-3,039685	0,0023683	0,010842	ASPSCR1
ENSGALG00000001788	3234,7706	-0,331738591	0,1195268	-2,775432	0,0055128	0,0217718	ADPGK
ENSGALG00000014445	19258,2	-0,331954776	0,1074559	-3,08922	0,0020068	0,0094707	NOP2
ENSGALG00000011904	3328,8747	-0,332013213	0,1288249	-2,577244	0,0099592	0,0350644	TRMT5
ENSGALG00000007590	10666,564	-0,332146464	0,1081338	-3,071625	0,002129	0,0099471	MTMR8
ENSGALG00000004806	7000,4013	-0,332607085	0,1094342	-3,039334	0,002371	0,0108489	EDEM3
ENSGALG00000004108	32339,09	-0,332634184	0,1003676	-3,314158	0,0009192	0,004992	CCAR1
ENSGALG00000015813	10809,928	-0,332684119	0,1173697	-2,834497	0,0045898	0,0186403	USP16
ENSGALG00000010967	21405,06	-0,332907911	0,1160936	-2,867581	0,0041362	0,017143	DOCK7
ENSGALG00000007528	28367,48	-0,33311917	0,1060953	-3,139811	0,0016906	0,0082087	TCERG1
ENSGALG00000016766	4441,5098	-0,333177403	0,118864	-2,803014	0,0050627	0,0202733	REV1
ENSGALG00000006051	6105,2991	-0,333204336	0,1106146	-3,0123	0,0025928	0,0116542	CDC7
ENSGALG00000005177	21314,802	-0,333333735	0,1014573	-3,285458	0,0010182	0,0054171	FAM120A
ENSGALG00000001976	9937,8689	-0,333600989	0,1086579	-3,070194	0,0021392	0,0099866	TRIM33
ENSGALG00000011704	13971,193	-0,333952521	0,1117683	-2,987901	0,002809	0,012486	PTPLB
ENSGALG00000010282	7159,3971	-0,334004467	0,1089729	-3,065022	0,0021765	0,0101207	TMEM63B
ENSGALG00000026315	3315,4842	-0,334081105	0,119333	-2,799571	0,0051171	0,0204367	RAB34
ENSGALG00000015505	6307,2082	-0,334198908	0,1151962	-2,901129	0,0037182	0,0157558	YTHDF3
ENSGALG00000009332	24425,521	-0,334253024	0,1022919	-3,26764	0,0010845	0,0057141	RBM25
ENSGALG00000005042	3847,7663	-0,334275581	0,1289111	-2,593071	0,0095123	0,0337354	IQSEC1
ENSGALG00000003169	2023,2915	-0,334437759	0,1334861	-2,505413	0,0122309	0,0412084	CBFB
ENSGALG00000012130	1792,2693	-0,334703976	0,1357589	-2,46543	0,0136849	0,044964	DNAJC24
ENSGALG00000004325	13667,765	-0,334704266	0,1162502	-2,879172	0,0039872	0,0166514	P4HA1
ENSGALG00000004088	4344,9033	-0,335048738	0,1281962	-2,613561	0,0089604	0,0321873	FNBP1
ENSGALG00000009500	5544,5023	-0,33525036	0,1244385	-2,694104	0,0070578	0,0265889	VPS50
ENSGALG00000010733	14379,472	-0,335293656	0,1230465	-2,724935	0,0064314	0,0246658	CEP170

ENSGALG00000015858	4190,1025	-0,335665691	0,1163166	-2,885795	0,0039043	0,0163839	DOPEY1
ENSGALG00000004523	6662,5854	-0,335874075	0,1147961	-2,925833	0,0034354	0,0147175	MFN2
ENSGALG00000011533	4440,3034	-0,335898283	0,1139943	-2,946625	0,0032126	0,0139255	XRCC3
ENSGALG00000014298	14009,413	-0,335955725	0,1061838	-3,163909	0,0015567	0,0076817	RFC1
ENSGALG00000028471	3553,6714	-0,335976223	0,1281632	-2,621473	0,0087551	0,0316265	CNPY3
ENSGALG00000014822	2960,1308	-0,336894676	0,1318948	-2,554268	0,0106411	0,0369778	CTC1
ENSGALG00000003231	28849,937	-0,336908133	0,1031629	-3,265788	0,0010916	0,0057449	KIF20A
ENSGALG00000018800	6576,7401	-0,337277222	0,1183588	-2,849617	0,0043772	0,0179396	POP1
ENSGALG00000016898	2197,9018	-0,33731956	0,1297596	-2,599573	0,009334	0,0332327	TGDS
ENSGALG00000006617	7019,2341	-0,337429872	0,117642	-2,868278	0,0041271	0,0171209	BCCIP
ENSGALG00000009494	6742,6053	-0,337457352	0,1118976	-3,015769	0,0025633	0,0115407	THAP5
ENSGALG00000009410	4067,5004	-0,337671951	0,1159276	-2,912783	0,0035822	0,0152603	PEX1
ENSGALG00000009097	3815,7028	-0,338568689	0,1171869	-2,889135	0,003863	0,0162676	SNAP23
ENSGALG00000009464	6021,3203	-0,338614502	0,1119841	-3,023773	0,0024964	0,0113033	GOLIM4
ENSGALG00000014327	7067,9269	-0,338948318	0,1081812	-3,133152	0,0017294	0,0083614	ARID4B
ENSGALG00000002302	11281,327	-0,339317581	0,1087619	-3,119821	0,0018096	0,0086661	YEATS2
ENSGALG00000020569	5039,2721	-0,339770586	0,1193898	-2,845892	0,0044287	0,0181143	DIMT1
ENSGALG00000012617	6367,5718	-0,339828609	0,1237855	-2,745303	0,0060455	0,0234167	TGFBR1
ENSGALG00000000408	12091,853	-0,339880371	0,121973	-2,786521	0,0053277	0,0211518	LSM7
ENSGALG00000013403	2742,2413	-0,340019904	0,1302877	-2,609761	0,0090605	0,0324549	MSANTD3
ENSGALG00000004089	8925,1812	-0,340215935	0,1167229	-2,914732	0,0035599	0,0151891	PRDM2
ENSGALG00000005966	13999,743	-0,340533213	0,1101239	-3,092273	0,0019863	0,0093851	SEC61A1
ENSGALG00000016255	2201,4032	-0,340652705	0,131986	-2,580977	0,0098521	0,0347594	RPGR
ENSGALG00000006601	4208,8165	-0,340895714	0,1177608	-2,894815	0,0037938	0,0160114	MTMR14
ENSGALG00000012202	8130,087	-0,341191282	0,1080608	-3,157402	0,0015918	0,0078127	KBTBD2
ENSGALG00000004759	3321,8502	-0,341574811	0,1390989	-2,455626	0,0140639	0,0459627	DHRS7B
ENSGALG00000007989	28672,589	-0,341827011	0,1092587	-3,128603	0,0017564	0,0084619	ADNP
ENSGALG00000003263	5162,4302	-0,341906085	0,1243755	-2,748982	0,0059781	0,0232054	SFXN1
ENSGALG00000010361	9329,7103	-0,342127455	0,1233327	-2,774021	0,0055368	0,0218567	MBNL1
ENSGALG00000004515	10691,241	-0,342166688	0,1169935	-2,924664	0,0034483	0,014759	KNTC1
ENSGALG00000010541	14976,655	-0,3427229	0,1277901	-2,681921	0,0073201	0,02741	BIRC6
ENSGALG00000004628	2015,8556	-0,342763414	0,1400684	-2,447115	0,0144005	0,0467957	CTNS
ENSGALG00000005237	11277,397	-0,342909039	0,1056363	-3,24613	0,0011699	0,0060815	FGD3
ENSGALG00000005420	7995,347	-0,343276467	0,1261306	-2,721596	0,0064967	0,0248884	AATF
ENSGALG00000002648	3882,3909	-0,343349251	0,1203014	-2,854076	0,0043162	0,017756	FUK
ENSGALG00000016314	22102,711	-0,343364339	0,1069912	-3,209276	0,0013307	0,0067704	ELOVL5
ENSGALG00000006455	4218,2466	-0,343579981	0,1158475	-2,965794	0,003019	0,0132318	SMPD4
ENSGALG00000012239	9046,4232	-0,343596083	0,1058983	-3,244586	0,0011762	0,0061076	METAP1
ENSGALG00000004624	11632,549	-0,34416889	0,1347829	-2,553506	0,0106645	0,0370494	MYO5A
ENSGALG00000003365	1822,7969	-0,344340057	0,1360837	-2,530355	0,0113947	0,0389601	RAD1
ENSGALG00000002527	4444,3372	-0,344545241	0,1227619	-2,806613	0,0050065	0,0200778	TMEM201
ENSGALG00000009813	1922,5946	-0,344705696	0,1334833	-2,582388	0,0098119	0,0346715	B0LHV3_CHICK
ENSGALG00000013053	3803,4898	-0,345339591	0,1218833	-2,833364	0,0046061	0,0186977	MTRR
ENSGALG00000001494	9000,8803	-0,345374318	0,1246336	-2,771118	0,0055864	0,021976	NISCH
ENSGALG00000011884	7925,1907	-0,345461445	0,1150003	-3,004005	0,0026645	0,0119412	EXOSC9
ENSGALG00000001236	5792,0595	-0,345759524	0,1103803	-3,132439	0,0017336	0,0083777	ZBTB26
ENSGALG00000005261	15832,957	-0,345768981	0,1275962	-2,70987	0,006731	0,0256198	RP11-403P17.5
ENSGALG00000012837	7840,9496	-0,345878883	0,1096612	-3,154068	0,0016101	0,0078884	EXOC2
ENSGALG00000006307	2423,8788	-0,346032557	0,1338086	-2,586026	0,009709	0,0343344	NSMCE1
ENSGALG00000003434	9368,6549	-0,346127339	0,1194628	-2,897364	0,0037631	0,0159164	C17orf75
ENSGALG00000006153	2844,7772	-0,346474519	0,1286035	-2,69413	0,0070573	0,0265889	EXOG
ENSGALG00000006475	6864,1942	-0,347016477	0,1079405	-3,214886	0,001305	0,0066643	KIAA0430
ENSGALG00000002276	1478,127	-0,34721896	0,1416798	-2,45073	0,0142567	0,0464477	EME2
ENSGALG00000015553	10445,976	-0,347410904	0,1127	-3,082616	0,0020519	0,0096499	ARFGEF1
ENSGALG00000011831	9083,6449	-0,347478626	0,1108523	-3,13461	0,0017208	0,0083259	ANKRD50
ENSGALG00000004269	64393,653	-0,347526191	0,103785	-3,348521	0,0008124	0,0044938	EIF3B
ENSGALG00000004548	15606,046	-0,347529204	0,1215975	-2,85803	0,0042628	0,0175766	RP11-101E3.5
ENSGALG00000005477	8038,2726	-0,347589933	0,1308189	-2,657031	0,0078832	0,0291104	MPHOSPH6
ENSGALG00000004200	11378,582	-0,347970886	0,1075116	-3,236588	0,0012097	0,0062623	DPP9
ENSGALG00000012364	4848,5693	-0,348109095	0,1264902	-2,752064	0,0059221	0,0230238	PPIL4
ENSGALG00000016887	64639,106	-0,348222743	0,0978749	-3,557836	0,0003739	0,002345	IPO5
ENSGALG00000013649	18631,187	-0,348311839	0,1020152	-3,414312	0,0006394	0,003678	CNOT7

ENSGALG00000017241	2897,41	-0,348538577	0,1300034	-2,680995	0,0073404	0,0274784	TMEM135
ENSGALG00000016931	5043,2864	-0,348656857	0,1247665	-2,794476	0,0051984	0,0207069	BORA
ENSGALG00000002469	8118,1027	-0,348923543	0,1164279	-2,996907	0,0027273	0,0121867	CDC73
ENSGALG00000004168	7087,525	-0,349365069	0,1171636	-2,981857	0,0028651	0,0126896	FEM1A
ENSGALG00000027444	3608,9781	-0,349517124	0,1245455	-2,806341	0,0050108	0,0200861	
ENSGALG00000017139	10651,91	-0,349554954	0,107047	-3,265433	0,001093	0,0057499	ZMYM2
ENSGALG00000002719	17659,446	-0,349571816	0,1018084	-3,433625	0,0005956	0,0034712	CSNK1D
ENSGALG00000008645	8917,9646	-0,349922505	0,1156037	-3,026915	0,0024706	0,0112201	NKRF
ENSGALG00000004042	4817,4388	-0,349988244	0,1306697	-2,67842	0,007397	0,0276565	NUFIP2
ENSGALG00000009633	4605,1827	-0,350007911	0,1141085	-3,067326	0,0021598	0,0100534	KATNBL1
ENSGALG00000007500	4924,5265	-0,350118918	0,1432067	-2,44485	0,0144912	0,047043	DENND4A
ENSGALG00000009249	5232,7054	-0,350123017	0,1287908	-2,71854	0,0065571	0,0250618	UBR1
ENSGALG00000008454	36878,682	-0,351028215	0,1038189	-3,38116	0,0007218	0,0040828	NOP58
ENSGALG00000016727	9641,1293	-0,351861521	0,1225244	-2,871766	0,0040818	0,0169796	JADE3
ENSGALG00000001023	2133,9525	-0,352229585	0,1347749	-2,613466	0,0089629	0,0321873	BZRAP1
ENSGALG00000017026	9366,3877	-0,352330056	0,1086684	-3,24225	0,0011859	0,0061532	VPS36
ENSGALG00000007861	11084,886	-0,352469383	0,1082462	-3,256183	0,0011292	0,0059177	MAGT1
ENSGALG00000005569	11340,438	-0,352585966	0,10935	-3,224379	0,0012625	0,00648	USP47
ENSGALG00000009283	5220,1674	-0,352608684	0,1245251	-2,831627	0,0046312	0,0187848	KIAA1715
ENSGALG00000006612	7533,7656	-0,353107966	0,1223847	-2,885229	0,0039113	0,0164004	ASXL1
ENSGALG00000009913	62516,864	-0,353547892	0,0988051	-3,578237	0,0003459	0,0021977	SMARCA5
ENSGALG00000011620	9603,3836	-0,353615473	0,1055478	-3,350286	0,0008073	0,0044752	AKT1
ENSGALG00000010177	12046,319	-0,353917428	0,1080741	-3,274766	0,0010575	0,0056001	ZFC3H1
ENSGALG00000015043	11145,365	-0,35411601	0,1099631	-3,220318	0,0012805	0,006559	RPF2
ENSGALG00000011368	2716,7046	-0,354344153	0,1315134	-2,694357	0,0070525	0,0265834	ZNF839
ENSGALG00000015024	3631,7153	-0,355056011	0,138118	-2,570671	0,0101502	0,0355805	RCL1
ENSGALG00000013863	3597,6259	-0,35519316	0,1205708	-2,94593	0,0032199	0,0139435	PSMG2
ENSGALG00000009380	1987,1722	-0,355339175	0,1456281	-2,440045	0,0146854	0,0475709	ST7
ENSGALG00000008036	5782,6274	-0,355406874	0,1116416	-3,183463	0,0014552	0,0072656	TMEM209
ENSGALG00000014383	9301,5506	-0,355665092	0,1163485	-3,056896	0,0022364	0,0103287	PI4K2B
ENSGALG00000010805	16069,419	-0,355813089	0,1260649	-2,822459	0,0047657	0,0192708	USP24
ENSGALG00000021031	4102,9147	-0,355958904	0,1319256	-2,698179	0,006972	0,0263165	ACKR3
ENSGALG00000005125	7959,3271	-0,356020767	0,1067406	-3,335383	0,0008518	0,0046764	LSM14B
ENSGALG00000012717	4761,9391	-0,356301296	0,1163989	-3,061036	0,0022057	0,0102355	NFYB
ENSGALG00000006123	7411,1798	-0,356657256	0,13028	-2,73762	0,0061885	0,0238689	RTEL1
ENSGALG00000006254	6379,7603	-0,357023226	0,1173769	-3,041681	0,0023526	0,0107811	ERCC6
ENSGALG00000001811	14773,974	-0,357025162	0,1141916	-3,126546	0,0017687	0,0085114	RP4-614O4.11
ENSGALG00000003441	4610,7331	-0,357140672	0,1182742	-3,0196	0,0025311	0,0114298	GTF3C5
ENSGALG00000008957	22456,525	-0,357191503	0,1156693	-3,088042	0,0020148	0,0094985	MSH6
ENSGALG00000012782	4940,896	-0,35754473	0,1182911	-3,022584	0,0025063	0,0113327	BLOC1S5
ENSGALG00000013657	2568,382	-0,35785969	0,136842	-2,615131	0,0089193	0,0320837	ZNF236
ENSGALG00000008218	3293,8403	-0,357888042	0,1386724	-2,580816	0,0098567	0,0347614	DDB2
ENSGALG00000009330	6639,4947	-0,358006963	0,1142065	-3,134734	0,0017201	0,0083253	DEGS1
ENSGALG00000010763	6453,8983	-0,358059956	0,1126815	-3,177629	0,0014848	0,0073728	TRIP11
ENSGALG00000008507	23518,146	-0,358201653	0,1023769	-3,498854	0,0004673	0,0028338	THOC2
ENSGALG00000009839	30979,576	-0,358257804	0,1012165	-3,539518	0,0004009	0,002491	PPIG
ENSGALG00000010349	7048,1543	-0,358393493	0,1129044	-3,174309	0,0015019	0,0074414	POLR1C
ENSGALG00000007721	11622,16	-0,358457512	0,1098179	-3,264109	0,0010981	0,0057724	ARHGAP21
ENSGALG00000012414	1870,7753	-0,358664924	0,1359051	-2,639084	0,0083131	0,0303275	GNPNAT1
ENSGALG00000016419	4650,1008	-0,358846606	0,1220043	-2,941261	0,0032688	0,0141048	ASAP2
ENSGALG00000002399	4037,3816	-0,359044332	0,1192358	-3,011213	0,0026021	0,01169	GTF2H2
ENSGALG00000014795	5578,5367	-0,359083857	0,1276747	-2,812491	0,0049159	0,0198137	TGIF1
ENSGALG00000005694	30867,661	-0,359162398	0,1021823	-3,514918	0,0004399	0,0026893	GARS
ENSGALG00000016598	5686,2877	-0,359376196	0,1202475	-2,988638	0,0028022	0,0124641	MSL3
ENSGALG00000008254	17757,266	-0,35964551	0,1079509	-3,331565	0,0008636	0,0047277	ANAPC1
ENSGALG00000007683	31803,136	-0,359759566	0,1106419	-3,251567	0,0011477	0,0059985	
ENSGALG00000005374	3276,9339	-0,359759753	0,1426205	-2,522497	0,0116525	0,0396423	MYO19
ENSGALG00000004670	7571,3318	-0,360055666	0,109156	-3,298544	0,0009719	0,0052095	ATRIP
ENSGALG00000024039	12181,866	-0,36099515	0,1049573	-3,439449	0,0005829	0,0034066	UTP14A
ENSGALG00000004379	20460,522	-0,361272684	0,1033693	-3,494972	0,0004741	0,0028704	BCL7A
ENSGALG00000000463	2471,2656	-0,361708293	0,131949	-2,741273	0,0061202	0,0236453	DDX25
ENSGALG00000006955	3003,9828	-0,36242424	0,1397632	-2,59313	0,0095107	0,0337354	CCNJ

ENSGALG00000012763	12297,883	-0,362574608	0,1084037	-3,344671	0,0008238	0,0045427	GNPTAB
ENSGALG00000001942	6053,3632	-0,362623052	0,1158608	-3,129817	0,0017492	0,0084479	SPDL1
ENSGALG00000012638	9149,4343	-0,362691803	0,1227328	-2,955134	0,0031253	0,0136121	CRY1
ENSGALG00000012916	8111,1057	-0,362729901	0,1091942	-3,321879	0,0008941	0,0048792	DROSHA
ENSGALG00000002139	98917,168	-0,362887161	0,1016046	-3,571563	0,0003549	0,0022423	CSDE1
ENSGALG00000009325	21860,643	-0,363294006	0,1110082	-3,272678	0,0010653	0,0056394	OLA1
ENSGALG00000015993	4919,2212	-0,363761464	0,123068	-2,955776	0,0031188	0,0135925	MTERF3
ENSGALG00000015363	3478,9826	-0,363849789	0,1186073	-3,067684	0,0021572	0,0100482	HJURP
ENSGALG00000003826	8394,886	-0,363869123	0,1071172	-3,396925	0,0006815	0,0038903	RP11-478C19.2
ENSGALG00000001790	14734,645	-0,363943316	0,1104661	-3,294616	0,0009856	0,0052683	RP11-318A15.7
ENSGALG00000004553	9745,0828	-0,363967467	0,1049477	-3,468085	0,0005242	0,0031206	SGPL1
ENSGALG00000002471	4521,5673	-0,363984132	0,1261711	-2,884846	0,0039161	0,0164153	ZNF592
ENSGALG00000001663	10259,573	-0,364202029	0,1099047	-3,3138	0,0009204	0,0049964	MAK16
ENSGALG00000007199	3668,9433	-0,36428019	0,1273389	-2,860714	0,0042269	0,0174655	TRIAP1
ENSGALG00000010900	12043,923	-0,364291872	0,1128172	-3,229045	0,001242	0,0064006	SPC25
ENSGALG00000010618	2510,6274	-0,364367021	0,127904	-2,848755	0,0043891	0,0179775	SLC30A6
ENSGALG00000003563	13287,766	-0,364389392	0,1101047	-3,309482	0,0009347	0,0050615	YARS
ENSGALG00000000638	12789,677	-0,364411808	0,1251634	-2,911488	0,0035971	0,0153046	UTP4
ENSGALG00000010006	4182,5012	-0,364534028	0,1219632	-2,988885	0,0028	0,0124581	SLC10A7
ENSGALG00000002137	15220,893	-0,364569255	0,1047688	-3,479749	0,0005019	0,0030089	PGAM5
ENSGALG00000004485	1975,7552	-0,364730971	0,1340663	-2,720526	0,0065178	0,024948	DOLPP1
ENSGALG00000004467	5426,4673	-0,365351749	0,1191675	-3,065867	0,0021704	0,0100991	CLIP1
ENSGALG00000001126	13435,921	-0,365650894	0,1039475	-3,51765	0,0004354	0,0026666	CHAF1A
ENSGALG00000003674	4753,1822	-0,365887371	0,1502656	-2,434938	0,0148943	0,0481221	DHTKD1
ENSGALG00000015874	13228,099	-0,365980992	0,1079617	-3,389915	0,0006991	0,0039761	TTK
ENSGALG00000003339	10767,061	-0,366040447	0,1177244	-3,109301	0,0018753	0,0089243	MTOR
ENSGALG00000010400	7431,8392	-0,366106792	0,1070902	-3,418676	0,0006293	0,0036317	SMARCAD1
ENSGALG00000012205	2497,2514	-0,366370844	0,1293829	-2,831679	0,0046304	0,0187848	AVL9
ENSGALG00000014867	5553,4309	-0,366420731	0,1173966	-3,12122	0,001801	0,0086359	PAIP1
ENSGALG00000006193	20925,466	-0,366551091	0,1152835	-3,179563	0,001475	0,0073322	TXNRD3
ENSGALG00000012807	3029,2492	-0,366699033	0,1239604	-2,958195	0,0030945	0,0135079	RPP40
ENSGALG00000009587	5817,192	-0,366858159	0,1201432	-3,053508	0,0022618	0,0104283	GPHN
ENSGALG00000011242	2355,7326	-0,367017354	0,1308827	-2,80417	0,0050446	0,0202098	OBSL1
ENSGALG00000005353	4596,566	-0,367159893	0,1195448	-3,071317	0,0021312	0,009954	FAR1
ENSGALG00000001872	9491,7801	-0,367383699	0,1243406	-2,954657	0,0031302	0,0136288	SDF4
ENSGALG00000015683	11433,193	-0,367498221	0,1101651	-3,335886	0,0008503	0,0046699	GNG10
ENSGALG00000003171	20304,055	-0,367614474	0,1019213	-3,606847	0,0003099	0,0019961	IREB2
ENSGALG00000028828	22676,693	-0,367651199	0,1028075	-3,576112	0,0003487	0,0022146	EIF4E
ENSGALG00000009637	4483,6182	-0,367846509	0,1376888	-2,671578	0,0075495	0,0281146	EMC7
ENSGALG00000013100	3042,9761	-0,368006107	0,1414611	-2,601465	0,0092826	0,0330933	GRB10
ENSGALG00000015009	1981,8828	-0,368164733	0,1354772	-2,71754	0,0065769	0,0251107	ZBTB49
ENSGALG00000024159	24561,804	-0,368265263	0,1065192	-3,457267	0,0005457	0,0032246	
ENSGALG00000003881	9972,6997	-0,368271023	0,1180506	-3,119604	0,0018109	0,0086695	ERCC6L
ENSGALG00000015957	2851,4747	-0,368440661	0,1383223	-2,663638	0,0077301	0,0286811	RNF151
ENSGALG00000001619	9403,9871	-0,368532524	0,1142023	-3,227015	0,0012509	0,006434	ATG16L1
ENSGALG00000004115	26356,095	-0,369026879	0,1077431	-3,425063	0,0006147	0,0035687	DNAJA2
ENSGALG00000012266	5845,0519	-0,369135559	0,1123441	-3,285757	0,0010171	0,0054135	LRRFIP2
ENSGALG00000015355	10608,533	-0,369224786	0,1221453	-3,022833	0,0025042	0,0113271	CD47
ENSGALG00000008526	11139,841	-0,369313202	0,1088311	-3,393452	0,0006902	0,003935	BRD1
ENSGALG00000001618	4391,5637	-0,369471319	0,1261006	-2,929973	0,0033899	0,0145504	OGFOD3
ENSGALG00000001485	7897,8046	-0,369653264	0,1079491	-3,424331	0,0006163	0,0035753	TOP3B
ENSGALG00000012263	9464,4086	-0,36996383	0,1064406	-3,475778	0,0005094	0,0030417	NEMF
ENSGALG00000007509	10510,151	-0,37005709	0,1074005	-3,44558	0,0005698	0,0033455	GNAS
ENSGALG00000008807	7903,608	-0,370129883	0,107044	-3,457735	0,0005447	0,0032218	DCUN1D1
ENSGALG00000009547	3006,9462	-0,370182446	0,1224834	-3,022307	0,0025086	0,0113355	PPHLN1
ENSGALG00000014582	21606,21	-0,370326496	0,1092612	-3,389368	0,0007005	0,0039824	CADM1
ENSGALG00000010052	1789,2611	-0,370466621	0,138855	-2,668012	0,0076302	0,0283683	PPP2R3C
ENSGALG00000010296	6483,9405	-0,370634079	0,1153594	-3,212864	0,0013142	0,0067014	MRPS18A
ENSGALG00000011215	4111,7689	-0,370718656	0,1158847	-3,199029	0,0013789	0,0069662	POC1B
ENSGALG00000004254	3591,4431	-0,370755667	0,1184788	-3,1293	0,0017522	0,0084598	TMEM120B
ENSGALG00000015649	13211,542	-0,370890117	0,1067035	-3,475894	0,0005092	0,0030417	NOP14
ENSGALG00000008557	15377,869	-0,371010327	0,1075723	-3,44894	0,0005628	0,0033142	ARL5B

ENSGALG00000004224	5462,5737	-0,371126705	0,1408849	-2,634254	0,0084322	0,0306998	MAD1L1
ENSGALG00000008795	3264,8024	-0,371277159	0,1212162	-3,062934	0,0021918	0,0101812	GPAM
ENSGALG00000006586	3512,8975	-0,371428946	0,1391432	-2,6694	0,0075987	0,0282744	PLAGL2
ENSGALG00000016614	8922,873	-0,371522735	0,1401003	-2,651835	0,0080056	0,0294504	SHROOM2
ENSGALG00000015847	1545,0428	-0,371535052	0,1402786	-2,64855	0,0080838	0,02965	PRSS35
ENSGALG00000009817	1241,5525	-0,371617458	0,1484632	-2,503095	0,0123113	0,041428	ZNF770
ENSGALG00000005935	6194,9397	-0,371684146	0,110711	-3,357247	0,0007872	0,0043945	EVI5
ENSGALG00000009807	6561,2068	-0,371941676	0,1238286	-3,003683	0,0026673	0,011946	FLVCR1
ENSGALG00000004834	12710,539	-0,372460145	0,1085125	-3,432418	0,0005982	0,0034837	COPS3
ENSGALG00000016578	7057,3465	-0,372483757	0,11837	-3,146774	0,0016508	0,0080444	
ENSGALG00000003039	8776,92	-0,372512284	0,1093684	-3,406031	0,0006591	0,0037723	SRM
ENSGALG00000008787	2268,5743	-0,372527529	0,1464722	-2,543332	0,0109801	0,0379137	CEP68
ENSGALG00000015705	13014,296	-0,372615903	0,1210163	-3,079056	0,0020766	0,0097424	WHSC1
ENSGALG00000015800	3887,9053	-0,37263738	0,128126	-2,908367	0,0036332	0,0154341	ORC3
ENSGALG00000011713	7918,9457	-0,372947939	0,1156001	-3,226189	0,0012545	0,0064477	CDV3
ENSGALG00000001513	6151,0051	-0,373009041	0,1249789	-2,984577	0,0028397	0,0125937	CNTRL
ENSGALG00000023536	26304,92	-0,373188455	0,1263177	-2,954363	0,0031332	0,0136331	FZD7
ENSGALG00000000412	5015,4199	-0,373361237	0,1191944	-3,132372	0,001734	0,0083777	CCDC15
ENSGALG00000003942	9873,2639	-0,373719716	0,1078722	-3,464467	0,0005313	0,0031559	LONP2
ENSGALG00000016236	30922,987	-0,374432078	0,1135095	-3,298685	0,0009714	0,0052095	USP9X
ENSGALG00000005618	12381,341	-0,374714579	0,1064892	-3,518803	0,0004335	0,002661	CTR9
ENSGALG00000010744	5078,2186	-0,375014992	0,1175988	-3,188937	0,001428	0,0071662	GALNT7
ENSGALG00000012689	4208,649	-0,375087396	0,1416897	-2,647245	0,0081151	0,0297327	KIF13A
ENSGALG00000001939	11757,482	-0,375413327	0,1227756	-3,057719	0,0022303	0,0103145	HIPK1
ENSGALG00000009626	5852,8685	-0,375704266	0,1201786	-3,126215	0,0017707	0,0085128	THBS1
ENSGALG00000021251	12519,129	-0,375765974	0,1228993	-3,057511	0,0022318	0,0103145	DDX6
ENSGALG00000003325	1577,6189	-0,375811622	0,1416562	-2,652984	0,0079784	0,029382	KMT5A
ENSGALG00000013034	8837,0774	-0,375844048	0,1530686	-2,455396	0,0140729	0,045981	CECR2
ENSGALG00000009677	24959,786	-0,376298569	0,10418	-3,612004	0,0003038	0,0019633	SSB
ENSGALG00000003776	26231,392	-0,376541512	0,1408091	-2,674127	0,0074924	0,0279477	PRRC2B
ENSGALG00000004867	10358,587	-0,376546151	0,1068635	-3,523619	0,0004257	0,0026261	CEBPG
ENSGALG00000000085	18552,019	-0,37666785	0,1073497	-3,508793	0,0004501	0,0027434	GFPT1
ENSGALG00000007929	3448,5443	-0,376720223	0,1459515	-2,581133	0,0098477	0,0347594	DIXDC1
ENSGALG00000011281	6523,2043	-0,37690737	0,1178655	-3,197776	0,0013849	0,0069939	SGOL1
ENSGALG00000003740	3014,38	-0,377276945	0,1362236	-2,769542	0,0056135	0,0220587	IKBKB
ENSGALG00000016337	3166,2527	-0,377573201	0,1356955	-2,782504	0,0053941	0,0213616	TATDN1
ENSGALG00000012251	2154,1949	-0,377607005	0,1483053	-2,546147	0,0108919	0,0376475	KDELR3
ENSGALG00000008247	7600,6846	-0,377692943	0,109538	-3,448054	0,0005646	0,0033222	ARFGAP2
ENSGALG00000005006	11070,754	-0,377708377	0,1075988	-3,510339	0,0004475	0,0027311	UBA2
ENSGALG00000000978	4782,6943	-0,377763607	0,1179131	-3,203745	0,0013565	0,0068761	CEP104
ENSGALG00000013146	5329,2902	-0,377879987	0,1326893	-2,847857	0,0044015	0,0180229	PLXNB2
ENSGALG00000006558	1973,6885	-0,378031821	0,1426055	-2,650891	0,008028	0,0295009	WDR60
ENSGALG00000005468	6023,6864	-0,378125454	0,1402851	-2,695408	0,0070303	0,026507	SYNRG
ENSGALG00000009093	6655,7671	-0,378156036	0,1279163	-2,956277	0,0031138	0,0135748	PNO1
ENSGALG00000002985	14604,542	-0,378678888	0,1056758	-3,583404	0,0003391	0,0021617	UTP18
ENSGALG00000016200	6807,9958	-0,378720539	0,1505954	-2,514821	0,0119093	0,0403647	SIK1
ENSGALG00000016851	8342,7517	-0,378897954	0,1201029	-3,154778	0,0016062	0,0078758	ARHGEF7
ENSGALG00000008195	5727,1148	-0,378910892	0,1131533	-3,348651	0,0008121	0,0044938	NPHP1
ENSGALG00000003326	2369,3349	-0,379089886	0,1276328	-2,97016	0,0029764	0,0130808	AMACR
ENSGALG00000011015	2757,9302	-0,379357377	0,1543896	-2,457143	0,0140047	0,045791	NTPCR
ENSGALG00000014085	1445,5651	-0,379926489	0,1417435	-2,68038	0,0073539	0,0275214	TM7SF3
ENSGALG00000006011	4210,2643	-0,380179632	0,1512099	-2,51425	0,0119286	0,040409	KIAA0753
ENSGALG00000024047	6900,307	-0,380223912	0,115759	-3,284616	0,0010212	0,0054312	MYCL
ENSGALG00000003322	2664,8777	-0,380407199	0,1320573	-2,880623	0,0039689	0,0165858	TBC1D2B
ENSGALG00000007036	8649,5744	-0,380541953	0,1060007	-3,589994	0,0003307	0,0021167	PITRM1
ENSGALG00000005573	47811,321	-0,380592325	0,1004918	-3,787297	0,0001523	0,0010811	NOLC1
ENSGALG00000008187	22960,701	-0,380821067	0,1023642	-3,720256	0,000199	0,0013569	EIF3J
ENSGALG00000000864	4682,0682	-0,380939683	0,115984	-3,284416	0,0010219	0,0054329	FBXW2
ENSGALG00000004696	6529,7846	-0,381141883	0,109965	-3,46603	0,0005282	0,0031404	LEO1
ENSGALG00000011330	86092,558	-0,381207584	0,1173583	-3,248237	0,0011612	0,0060506	DYNC1H1
ENSGALG00000000543	2560,0751	-0,381260503	0,1306196	-2,91886	0,0035131	0,0150036	TNFRSF9
ENSGALG00000005776	1699,2203	-0,38126111	0,1437588	-2,65209	0,0079995	0,0294363	TECR

ENSGALG00000007354	7140,2177	-0,381532857	0,1451021	-2,629409	0,0085533	0,0310378	CIT
ENSGALG00000011484	5458,5585	-0,381581605	0,1495733	-2,551135	0,0107373	0,0372359	CMTM8
ENSGALG00000012953	7564,6193	-0,381744469	0,1101496	-3,465692	0,0005289	0,003143	IPO8
ENSGALG00000007559	4279,2164	-0,381904484	0,1214524	-3,144479	0,0016638	0,0080933	PDSS1
ENSGALG00000017333	5175,4799	-0,382465165	0,1273235	-3,003886	0,0026656	0,0119419	LCMT2
ENSGALG00000010680	1744,2067	-0,38261968	0,1387981	-2,756664	0,0058394	0,0227414	TTC7B
ENSGALG00000015226	1410,5634	-0,382624366	0,1475175	-2,593756	0,0094934	0,0336858	SPIDR
ENSGALG00000008587	12026,073	-0,382953864	0,1040771	-3,679521	0,0002337	0,0015626	NDUFAF1
ENSGALG00000008493	1237,0726	-0,38325533	0,1561662	-2,45415	0,0141218	0,0461297	METTL21A
ENSGALG00000005301	7189,628	-0,383471192	0,1309696	-2,92794	0,0034122	0,0146283	CYB5R2
ENSGALG00000027355	3798,5712	-0,383473155	0,1422345	-2,696064	0,0070164	0,0264622	TPBG
ENSGALG00000001596	12885,592	-0,383722949	0,1090252	-3,51958	0,0004322	0,0026566	MLLT1
ENSGALG00000000054	13558,561	-0,383859531	0,1440809	-2,664195	0,0077173	0,0286687	OAT
ENSGALG00000004839	5677,6109	-0,383869776	0,1125796	-3,409763	0,0006502	0,003732	GPATCH1
ENSGALG00000002346	7982,3843	-0,383971209	0,1121749	-3,422968	0,0006194	0,0035886	CLSPN
ENSGALG00000007556	1766,8481	-0,384355829	0,1442884	-2,663803	0,0077263	0,0286811	ORAOV1
ENSGALG00000016040	7227,3379	-0,384424184	0,1139331	-3,374123	0,0007405	0,0041657	MORC3
ENSGALG00000011881	17920,009	-0,384788496	0,101237	-3,800869	0,0001442	0,0010289	CCNA2
ENSGALG00000016751	19933,902	-0,385163412	0,1317503	-2,923435	0,0034619	0,0148127	TMEM131
ENSGALG00000017105	3524,6327	-0,385190009	0,1190655	-3,235111	0,001216	0,0062852	RNF6
ENSGALG00000015010	9588,3759	-0,38521292	0,1153698	-3,338939	0,000841	0,0046244	NSG1
ENSGALG00000010061	5963,3093	-0,385385873	0,159264	-2,419792	0,0155294	0,0497371	LRBA
ENSGALG00000008542	24254,362	-0,385688801	0,137665	-2,801647	0,0050842	0,0203416	ADD3
ENSGALG00000015040	4593,8104	-0,38608617	0,1172115	-3,293928	0,000988	0,0052792	SLC16A10
ENSGALG00000001876	1186,4565	-0,386144808	0,1524506	-2,532918	0,0113118	0,0387543	RASA4
ENSGALG00000013061	7957,2214	-0,38617661	0,1127911	-3,423821	0,0006175	0,0035805	PAPD7
ENSGALG00000011357	6554,7154	-0,387039211	0,1110437	-3,485467	0,0004913	0,0029557	VEZT
ENSGALG00000006259	19676,996	-0,387217866	0,1021071	-3,792272	0,0001493	0,0010619	KIRREL
ENSGALG00000027774	12094,125	-0,387231267	0,1136139	-3,40831	0,0006537	0,0037441	ALMS1
ENSGALG00000002143	8359,2157	-0,387272727	0,1268467	-3,053076	0,0022651	0,0104398	ANKLE2
ENSGALG00000008280	7969,0775	-0,387606949	0,111093	-3,48903	0,0004848	0,002923	PMPCB
ENSGALG00000012724	10174,958	-0,387874274	0,1052864	-3,683992	0,0002296	0,001541	PAK1IP1
ENSGALG00000015247	7003,5436	-0,387982807	0,1080781	-3,589838	0,0003309	0,002117	GPR161
ENSGALG00000027262	21153,65	-0,388074983	0,1013462	-3,829203	0,0001286	0,0009315	CAB39
ENSGALG00000005575	18548,779	-0,388395592	0,1080882	-3,593321	0,0003265	0,0020938	AKAP1
ENSGALG00000010609	3709,2893	-0,388677075	0,1243181	-3,126472	0,0017692	0,0085114	NDUFAF7
ENSGALG00000005012	6065,171	-0,388993034	0,1215819	-3,199432	0,001377	0,006959	ISY1
ENSGALG00000005904	32126,543	-0,389017981	0,1104816	-3,521111	0,0004297	0,0026439	TMED5
ENSGALG00000011103	30281,636	-0,389199345	0,1020702	-3,813057	0,0001373	0,0009872	PAPOLA
ENSGALG00000007788	4541,3087	-0,389226557	0,117185	-3,321472	0,0008954	0,0048844	ABCB7
ENSGALG00000001073	7165,4084	-0,389421011	0,147194	-2,645631	0,0081539	0,0298497	NR6A1
ENSGALG00000003166	4550,9206	-0,38962163	0,1147713	-3,394765	0,0006869	0,0039178	REXO4
ENSGALG00000002129	6971,2134	-0,390332447	0,1113848	-3,504359	0,0004577	0,0027845	WDR47
ENSGALG00000027696	2856,8564	-0,390616277	0,1467859	-2,661129	0,0077879	0,0288524	
ENSGALG00000007485	13617,236	-0,391583137	0,1247391	-3,139217	0,001694	0,0082195	TFRC
ENSGALG00000008983	6819,4343	-0,391807233	0,114877	-3,410666	0,000648	0,0037213	GNB4
ENSGALG00000009364	15815,387	-0,391850394	0,1069886	-3,662544	0,0002497	0,0016558	PRKCI
ENSGALG00000015396	993,36818	-0,391937266	0,1599566	-2,450272	0,0142748	0,0464956	CD200
ENSGALG00000028035	5338,9185	-0,391942986	0,1230917	-3,184154	0,0014518	0,0072578	FBXO22
ENSGALG00000025858	3504,3476	-0,392172869	0,1322344	-2,96574	0,0030196	0,0132318	LUZP1
ENSGALG00000017064	15165,119	-0,392266132	0,1075067	-3,648759	0,0002635	0,001734	RFC3
ENSGALG00000013410	8555,6915	-0,392331865	0,1073488	-3,654738	0,0002574	0,0016999	UBA3
ENSGALG00000003277	6528,6806	-0,393093094	0,1163756	-3,377797	0,0007307	0,0041228	SUZ12
ENSGALG00000007312	1591,4187	-0,393109181	0,1537105	-2,557464	0,0105438	0,0367241	GNPDA1
ENSGALG00000002682	5643,0549	-0,393160876	0,1228268	-3,200938	0,0013698	0,0069331	BRD3
ENSGALG00000004481	5167,7274	-0,39336646	0,121645	-3,233724	0,0012219	0,0063086	EDA
ENSGALG00000015865	4560,3514	-0,393610955	0,1160346	-3,392187	0,0006934	0,0039501	FAM46A
ENSGALG00000009569	11508,232	-0,393918558	0,1069286	-3,683939	0,0002297	0,001541	TLK1
ENSGALG00000009201	11168,289	-0,39393497	0,1118548	-3,521842	0,0004286	0,0026399	CCNF
ENSGALG00000028133	7664,2653	-0,394703045	0,1165319	-3,387082	0,0007064	0,0040074	UBE2V2
ENSGALG00000028883	9007,8407	-0,394818784	0,1358341	-2,906626	0,0036535	0,0155154	NOL11
ENSGALG00000028898	2507,0991	-0,395098922	0,1286111	-3,072045	0,002126	0,0099434	GLTPD1

ENSGALG00000014166	1449,8688	-0,395137478	0,1437153	-2,749445	0,0059696	0,0231821	TSPO
ENSGALG00000016467	8711,5563	-0,395365049	0,1194699	-3,309327	0,0009352	0,0050615	SMC6
ENSGALG00000003043	3489,7523	-0,395518744	0,1229266	-3,217519	0,001293	0,0066184	BBS2
ENSGALG00000017024	1501,8199	-0,395600818	0,1535883	-2,575722	0,0100031	0,0351736	NEK3
ENSGALG00000008738	1562,619	-0,395613631	0,1395655	-2,83461	0,0045882	0,0186403	
ENSGALG00000002076	11878,124	-0,395918706	0,1140373	-3,471836	0,0005169	0,0030827	DGCR8
ENSGALG00000008521	16274,178	-0,395949661	0,1276174	-3,10263	0,0019181	0,0091151	TP53BP1
ENSGALG00000014688	9710,7864	-0,396059354	0,1249828	-3,16891	0,0015301	0,0075617	ZYX
ENSGALG00000003273	1825,9769	-0,396166645	0,1597575	-2,4798	0,0131456	0,0436637	HSDL1
ENSGALG00000004256	8236,2208	-0,396589984	0,1140041	-3,478736	0,0005038	0,003015	DHRS3
ENSGALG00000010643	4501,8566	-0,396627508	0,117516	-3,375094	0,0007379	0,004154	ZYG11B
ENSGALG00000012759	9679,7293	-0,396656849	0,1130301	-3,509303	0,0004493	0,0027403	NUP37
ENSGALG00000004435	2804,793	-0,396797105	0,1243897	-3,189952	0,001423	0,0071489	RAB27A
ENSGALG00000009608	5107,6787	-0,396825966	0,1273293	-3,116534	0,0018299	0,0087449	UBR3
ENSGALG00000009838	16688,775	-0,397053502	0,1016373	-3,906571	9,36E-05	0,0007093	AQR
ENSGALG00000009533	7127,7941	-0,397081701	0,1281809	-3,097822	0,0019495	0,0092417	NMD3
ENSGALG00000006563	2968,8404	-0,397183147	0,1427397	-2,78257	0,005393	0,0213616	SEMA3D
ENSGALG00000015435	3490,2323	-0,397319834	0,1216986	-3,264786	0,0010955	0,0057608	DCAF6
ENSGALG00000010886	2623,1957	-0,397321501	0,1327902	-2,992101	0,0027706	0,0123558	PAQR3
ENSGALG00000015215	6704,2917	-0,397390834	0,1204862	-3,298228	0,000973	0,0052112	MAPRE2
ENSGALG00000013776	3256,8373	-0,397455529	0,1277422	-3,111389	0,0018621	0,0088676	CEP135
ENSGALG00000002675	3557,3289	-0,397531672	0,1252523	-3,173848	0,0015043	0,0074478	SCUBE3
ENSGALG00000009322	5962,2024	-0,397973054	0,1135762	-3,504017	0,0004583	0,0027868	NVL
ENSGALG00000001576	13166,504	-0,398019961	0,106825	-3,725905	0,0001946	0,0013328	HIRA
ENSGALG00000003675	2269,9539	-0,398220954	0,1307081	-3,046644	0,0023141	0,0106334	PRKAR1B
ENSGALG00000010256	2730,229	-0,398269176	0,1444457	-2,757224	0,0058294	0,0227155	TIPARP
ENSGALG00000000838	3236,5634	-0,398388838	0,1429731	-2,786459	0,0053287	0,0211518	THAP12
ENSGALG00000008889	7462,9462	-0,398667809	0,107895	-3,694959	0,0002199	0,001483	TTC14
ENSGALG00000010674	9662,4596	-0,399166032	0,1113474	-3,58487	0,0003372	0,0021516	DCTD
ENSGALG00000011186	1570,9959	-0,399261707	0,1516141	-2,633408	0,0084533	0,0307484	ERMARD
ENSGALG00000006483	5623,947	-0,399267586	0,1210637	-3,297995	0,0009738	0,0052135	AMOTL2
ENSGALG00000004368	3768,914	-0,39990946	0,1253746	-3,189717	0,0014241	0,0071521	GNA12
ENSGALG00000001377	9703,5691	-0,400093128	0,1073106	-3,728364	0,0001927	0,0013239	POLRMT
ENSGALG00000023725	1256,3767	-0,400126756	0,1588481	-2,518927	0,0117713	0,0399966	IER5
ENSGALG00000020251	2658,2357	-0,40015479	0,1247153	-3,208547	0,0013341	0,0067774	FNIP2
ENSGALG00000007663	14617,833	-0,400171791	0,1147124	-3,488479	0,0004858	0,0029278	KDM3B
ENSGALG00000016437	3164,1402	-0,40025509	0,1368097	-2,925634	0,0034375	0,0147223	TAF1B
ENSGALG00000008289	17537,453	-0,401142997	0,1117051	-3,59109	0,0003293	0,0021088	RBM19
ENSGALG00000000332	20852,835	-0,401294712	0,1053884	-3,80777	0,0001402	0,0010059	FTSJ3
ENSGALG00000016747	15836,06	-0,401466819	0,10405	-3,858403	0,0001141	0,0008386	UBE3A
ENSGALG00000011542	3771,8431	-0,401482328	0,1540301	-2,606518	0,0091468	0,0326946	METTL6
ENSGALG00000001094	14665,623	-0,40156915	0,1240977	-3,235912	0,0012125	0,0062731	ADGRD2
ENSGALG00000011493	4936,2961	-0,401631833	0,1189649	-3,376053	0,0007353	0,0041422	TMEM169
ENSGALG00000010304	4399,9164	-0,401743756	0,1198974	-3,350729	0,000806	0,0044728	MLH3
ENSGALG00000001422	12037,379	-0,402405365	0,1260368	-3,192761	0,0014092	0,0070902	RRP9
ENSGALG00000005516	8167,2279	-0,402406965	0,1079699	-3,72703	0,0001937	0,0013282	HEATR6
ENSGALG00000010903	1862,2125	-0,40247318	0,14557	-2,764808	0,0056956	0,0222898	RASGEF1B
ENSGALG00000012138	29874,231	-0,402996238	0,1040941	-3,871462	0,0001082	0,0008023	sept-07
ENSGALG00000011379	3502,2993	-0,403264726	0,1218523	-3,309456	0,0009348	0,0050615	SLC44A5
ENSGALG00000004486	7663,2736	-0,403537703	0,1189928	-3,391278	0,0006957	0,0039614	ZCCHC8
ENSGALG00000011505	19437,736	-0,403647787	0,1041549	-3,875456	0,0001064	0,0007935	MARK3
ENSGALG00000007610	8753,2545	-0,403710794	0,1080893	-3,734976	0,0001877	0,0012942	CDC23
ENSGALG00000003219	2817,8802	-0,403748967	0,1250219	-3,229426	0,0012404	0,0063945	RP11-77K12.7
ENSGALG00000002602	3162,965	-0,403772578	0,1577228	-2,560015	0,0104668	0,036493	CSMD2
ENSGALG00000012919	4205,6363	-0,40384181	0,1377531	-2,931636	0,0033718	0,0144834	YARS2
ENSGALG00000015033	6515,5396	-0,403855123	0,1645709	-2,453988	0,0141282	0,0461393	REV3L
ENSGALG00000003033	4208,0329	-0,4040823	0,1239451	-3,260173	0,0011134	0,0058396	STXBP4
ENSGALG00000009887	7499,0415	-0,404219345	0,1116137	-3,621592	0,0002928	0,0019019	DIEXF
ENSGALG00000016070	5235,9258	-0,404284672	0,1139162	-3,548966	0,0003867	0,0024088	WRB
ENSGALG00000028679	2739,4279	-0,404433944	0,1560396	-2,591867	0,0095457	0,0338272	RRP8
ENSGALG00000005558	18603,537	-0,404537586	0,1021007	-3,962142	7,43E-05	0,0005792	TBL3
ENSGALG00000003358	9790,289	-0,404595333	0,1105122	-3,661092	0,0002511	0,0016639	UBIAD1

ENSGALG00000010428	7926,1945	-0,404704607	0,1082049	-3,74017	0,0001839	0,0012722	FAM98A
ENSGALG00000012509	6088,1178	-0,40489721	0,1172775	-3,452472	0,0005555	0,0032739	ARL5A
ENSGALG00000016034	11941,122	-0,4051791	0,11639	-3,481218	0,0004991	0,0029977	ATRN
ENSGALG00000016397	10814,151	-0,405217096	0,1464803	-2,766359	0,0056686	0,0222137	SOX11
ENSGALG00000009015	5602,2699	-0,40522305	0,1351963	-2,997294	0,0027239	0,0121752	PIGK
ENSGALG00000011405	5639,0186	-0,405273633	0,1171817	-3,458507	0,0005432	0,0032154	SLC4A7
ENSGALG00000002042	9110,0202	-0,405517273	0,1100944	-3,683359	0,0002302	0,001543	KNOP1
ENSGALG00000015313	1727,1874	-0,405609451	0,1673222	-2,424122	0,0153455	0,049264	PDSS2
ENSGALG00000011118	5098,9857	-0,405615226	0,1135418	-3,572385	0,0003537	0,0022401	MIER1
ENSGALG00000012756	1600,2653	-0,405783919	0,1444512	-2,809142	0,0049674	0,0199796	PARPBP
ENSGALG00000004300	11426,337	-0,405827457	0,1082534	-3,748866	0,0001776	0,0012377	PTDSS2
ENSGALG00000002107	25522,201	-0,406162719	0,1018979	-3,985977	6,72E-05	0,0005304	RANBP1
ENSGALG00000011571	3230,4791	-0,406488263	0,1210977	-3,356697	0,0007888	0,0044014	AGPAT4
ENSGALG00000015439	3241,7503	-0,406514431	0,1539648	-2,640308	0,0082831	0,0302474	SLC44A1
ENSGALG00000009800	11257,087	-0,406824322	0,137832	-2,951596	0,0031614	0,0137382	SETD7
ENSGALG00000014404	9644,7108	-0,407214942	0,1124218	-3,622207	0,0002921	0,0018983	GPR125
ENSGALG00000004001	29564,347	-0,407260268	0,1220375	-3,337173	0,0008464	0,0046501	LARP1
ENSGALG00000014165	2563,4692	-0,407468645	0,1414422	-2,880813	0,0039665	0,0165833	MCAT
ENSGALG00000000275	5245,7551	-0,407767805	0,1196134	-3,409049	0,0006519	0,0037377	KCTD9
ENSGALG00000013661	4102,8132	-0,40778269	0,1293022	-3,153718	0,001612	0,007895	MICU3
ENSGALG00000009534	2852,2534	-0,408065762	0,1282621	-3,1815	0,0014651	0,0072963	PLEKHA1
ENSGALG00000013715	2414,831	-0,408079285	0,1364191	-2,991364	0,0027773	0,0123776	DLC1
ENSGALG00000003143	15625,053	-0,408123735	0,1067545	-3,823012	0,0001318	0,0009527	ZC3H7A
ENSGALG00000001616	13431,828	-0,408530697	0,1073575	-3,805328	0,0001416	0,0010148	CDC45
ENSGALG00000003995	3806,3629	-0,408610387	0,1221472	-3,34523	0,0008221	0,0045354	ERAL1
ENSGALG00000028536	1522,6438	-0,40868801	0,1548439	-2,639355	0,0083064	0,0303114	MPEG1
ENSGALG00000011100	3743,986	-0,408960809	0,1243724	-3,288197	0,0010083	0,0053689	HIBADH
ENSGALG00000008523	12531,511	-0,409027447	0,1050057	-3,895289	9,81E-05	0,0007378	XPNPEP1
ENSGALG00000012607	5903,9235	-0,409282973	0,1276687	-3,20582	0,0013468	0,0068343	ZCCHC6
ENSGALG00000009483	9325,7747	-0,409303762	0,1092985	-3,744826	0,0001805	0,0012537	MARK1
ENSGALG00000008658	2008,7076	-0,409694565	0,1367947	-2,994959	0,0027448	0,0122607	ZNHIT6
ENSGALG00000013858	2841,1323	-0,410414198	0,1233001	-3,328579	0,0008729	0,0047767	CEP76
ENSGALG00000002673	4173,3236	-0,41052388	0,114577	-3,582951	0,0003397	0,0021645	GRPEL2
ENSGALG00000016345	4179,8127	-0,410542245	0,1255035	-3,271162	0,0010711	0,0056609	KLHL15
ENSGALG00000006404	23302,915	-0,411265326	0,1022948	-4,020395	5,81E-05	0,0004686	DDX46
ENSGALG00000002830	2218,8919	-0,411355669	0,1470401	-2,797575	0,0051488	0,0205453	NUDT3
ENSGALG00000011662	25762,115	-0,412100057	0,1171723	-3,517043	0,0004364	0,0026703	NUP205
ENSGALG00000006157	6346,9334	-0,412112134	0,1105255	-3,728661	0,0001925	0,0013231	DDX26B
ENSGALG00000015944	3056,9164	-0,412472548	0,1405652	-2,934387	0,0033421	0,0143722	RIMS1
ENSGALG00000000780	20148,91	-0,413093901	0,1091845	-3,783448	0,0001547	0,0010956	SMPD3
ENSGALG00000010734	12330,712	-0,413140268	0,115155	-3,587689	0,0003336	0,0021305	PPP4R3A
ENSGALG00000027373	11590,467	-0,413207621	0,1275875	-3,238621	0,0012011	0,0062229	
ENSGALG00000014811	9624,081	-0,413279775	0,1068315	-3,868519	0,0001095	0,0008103	SMCHD1
ENSGALG00000000860	3991,9401	-0,413426982	0,141168	-2,928617	0,0034047	0,0146048	SLC7A6
ENSGALG00000014464	1467,51	-0,413666995	0,1677373	-2,466159	0,0136571	0,0449024	MTR
ENSGALG00000027864	1064,9642	-0,413669777	0,1682884	-2,458101	0,0139674	0,045702	NFKBIA
ENSGALG00000009300	6311,4725	-0,413699825	0,1174909	-3,521122	0,0004297	0,0026439	NUMB
ENSGALG00000014978	5258,7361	-0,41400107	0,1181716	-3,503389	0,0004594	0,0027915	IQGAP2
ENSGALG00000004947	6549,1555	-0,414737624	0,1229203	-3,374037	0,0007407	0,0041657	POLR3A
ENSGALG00000001613	28424,625	-0,414922322	0,1050352	-3,950318	7,80E-05	0,0006041	GNL3
ENSGALG00000013943	3049,8006	-0,414996341	0,1265367	-3,279652	0,0010394	0,0055126	FIP1L1
ENSGALG00000012241	2635,2581	-0,415005332	0,1298425	-3,196222	0,0013924	0,0070187	KLHDC1
ENSGALG00000014708	1768,2325	-0,415209722	0,1395692	-2,974938	0,0029305	0,0129247	GSTK1
ENSGALG00000027593	7745,809	-0,415214085	0,1233358	-3,366535	0,0007612	0,0042684	ZNF292
ENSGALG00000000800	3898,7504	-0,415275292	0,1200235	-3,459951	0,0005403	0,003201	PRMT7
ENSGALG00000027666	1353,3857	-0,415292257	0,1696173	-2,448407	0,014349	0,0467036	TMEM80
ENSGALG00000012608	2590,1311	-0,415354931	0,1398222	-2,970593	0,0029723	0,0130666	DAPK1
ENSGALG00000009634	10679,835	-0,415471196	0,1072605	-3,873478	0,0001073	0,0007966	GFM1
ENSGALG00000012030	9331,9586	-0,415761326	0,140767	-2,953543	0,0031415	0,013665	QSER1
ENSGALG00000016972	5736,7284	-0,415815188	0,1184924	-3,509213	0,0004494	0,0027403	NUFIP1
ENSGALG00000026470	13532,223	-0,41655555	0,1120502	-3,71758	0,0002011	0,0013699	BOD1L1
ENSGALG00000010996	3758,0298	-0,416979888	0,1506983	-2,766985	0,0056577	0,0221925	IRF2BP2

ENSGALG00000003737	3492,3176	-0,417242803	0,1329577	-3,138163	0,0017001	0,0082461	TOX3
ENSGALG00000006818	7817,9637	-0,417614565	0,1586281	-2,632664	0,0084718	0,0307911	KMT2A
ENSGALG00000014386	4214,1295	-0,417751127	0,1215158	-3,437832	0,0005864	0,0034221	SEPSECS
ENSGALG00000014013	11695,533	-0,418753457	0,1054585	-3,970791	7,16E-05	0,0005625	ARHGAP18
ENSGALG00000015132	49891,397	-0,418923213	0,1007384	-4,158527	3,20E-05	0,000281	CDH2
ENSGALG00000004121	4190,9905	-0,419252057	0,1222891	-3,428369	0,0006072	0,003533	LPAR4
ENSGALG00000013681	3809,2674	-0,419314087	0,1641248	-2,55485	0,0106234	0,0369255	ZNF407
ENSGALG00000009961	27537,675	-0,419402034	0,1063815	-3,942434	8,07E-05	0,0006229	ABCE1
ENSGALG00000010201	4830,0616	-0,41947829	0,1360243	-3,083848	0,0020434	0,0096134	ABHD18
ENSGALG00000006526	6573,3308	-0,419710882	0,1536968	-2,730772	0,0063186	0,024288	PLXNB1
ENSGALG00000004716	1607,3564	-0,41985683	0,1470278	-2,855629	0,0042952	0,0176945	ZC4H2
ENSGALG00000019228	2351,715	-0,419863222	0,1356378	-3,095474	0,001965	0,0093023	SFT2D2
ENSGALG00000007227	17010,789	-0,420130207	0,125082	-3,358839	0,0007827	0,0043764	MSI1
ENSGALG00000000975	4091,6956	-0,420207875	0,1199453	-3,50333	0,0004595	0,0027915	
ENSGALG00000003985	6692,4044	-0,42022626	0,1152659	-3,645711	0,0002667	0,0017505	TARSL2
ENSGALG00000007736	8209,2388	-0,420323444	0,1102306	-3,813127	0,0001372	0,0009872	ZWILCH
ENSGALG00000014827	2639,1918	-0,420567115	0,1657257	-2,537731	0,0111574	0,0383605	KIAA0408
ENSGALG00000012188	1607,0265	-0,420636341	0,1572383	-2,675152	0,0074695	0,0278853	BBS9
ENSGALG00000008989	27837,925	-0,420708851	0,1092049	-3,852471	0,0001169	0,0008555	WDR43
ENSGALG00000017119	1864,3003	-0,420708881	0,1365688	-3,080563	0,0020661	0,0096988	TNFRSF19
ENSGALG00000023650	764,17678	-0,420788842	0,1697354	-2,479087	0,0131719	0,0437404	
ENSGALG00000010630	14099,6	-0,420914921	0,1126811	-3,735454	0,0001874	0,0012926	AHCTF1
ENSGALG00000004310	3386,8687	-0,4211147	0,1211839	-3,475005	0,0005108	0,0030492	TTYH3
ENSGALG00000010671	83381,23	-0,421121888	0,1031007	-4,084569	4,42E-05	0,0003679	HNRNPU
ENSGALG00000008466	2872,7321	-0,421406429	0,1332906	-3,16156	0,0015693	0,0077355	FAM117B
ENSGALG00000027311	10812,972	-0,421551178	0,1501476	-2,807579	0,0049915	0,0200413	NGFR
ENSGALG00000014937	3129,4872	-0,421603891	0,1255788	-3,357285	0,0007871	0,0043945	KPNA5
ENSGALG00000018803	3405,6338	-0,421885923	0,1263968	-3,33779	0,0008445	0,0046417	TMEM198
ENSGALG00000011965	3023,1564	-0,422009879	0,1389961	-3,036128	0,0023964	0,0109339	ZC3H7B
ENSGALG00000015692	1953,5689	-0,422243248	0,1336531	-3,159247	0,0015818	0,0077775	POLN
ENSGALG00000012579	13996,553	-0,422359298	0,1452155	-2,908501	0,0036317	0,0154323	BAZ2B
ENSGALG00000013607	7250,9812	-0,422561681	0,11201	-3,772535	0,0001616	0,0011382	FBXO5
ENSGALG00000016276	1028,3831	-0,422833677	0,1624303	-2,60317	0,0092366	0,0329724	KHDRBS2
ENSGALG00000015589	4158,3548	-0,422922749	0,1597028	-2,648186	0,0080925	0,029674	MSH3
ENSGALG00000006002	2601,7776	-0,422978689	0,1302324	-3,247876	0,0011627	0,0060536	WHDC1
ENSGALG00000006652	5529,9226	-0,423060365	0,1599744	-2,644551	0,0081799	0,0299301	DIP2C
ENSGALG00000027334	5378,2134	-0,423190484	0,1394998	-3,033628	0,0024163	0,0110138	
ENSGALG00000002763	8455,8856	-0,423193935	0,11583	-3,653579	0,0002586	0,0017067	FRZB
ENSGALG00000000962	2645,4224	-0,423212428	0,1264405	-3,347126	0,0008165	0,00451	PUS3
ENSGALG00000008708	9031,5502	-0,423296325	0,1063024	-3,982003	6,83E-05	0,0005384	ESF1
ENSGALG00000004297	1889,6269	-0,42334794	0,1444708	-2,930335	0,003386	0,014538	DNAH1
ENSGALG00000014568	6675,2521	-0,423460012	0,1137337	-3,723259	0,0001967	0,0013449	RP3-461F17.3
ENSGALG00000012216	1240,2281	-0,423752018	0,1593085	-2,659946	0,0078153	0,0289303	BMPR1B
ENSGALG00000001850	22801,494	-0,423798382	0,1070003	-3,960721	7,47E-05	0,0005817	RARS
ENSGALG00000016701	2038,8685	-0,423807866	0,1319527	-3,211817	0,001319	0,0067183	
ENSGALG00000003288	22428,698	-0,423843105	0,1239826	-3,418569	0,0006295	0,0036317	TARS
ENSGALG00000009177	7539,3588	-0,423896895	0,110067	-3,851261	0,0001175	0,0008592	FMR1
ENSGALG00000012852	33011,495	-0,42431457	0,1189985	-3,565714	0,0003629	0,0022862	MKRN1
ENSGALG00000002288	13379,292	-0,424413539	0,1057585	-4,013044	5,99E-05	0,0004809	LIG3
ENSGALG00000000644	5332,9382	-0,424448701	0,1720142	-2,467522	0,0136052	0,0448185	NFASC
ENSGALG00000011177	6032,2306	-0,424449663	0,1147226	-3,699791	0,0002158	0,0014601	CEP290
ENSGALG00000005945	41976,029	-0,425016747	0,1048948	-4,051839	5,08E-05	0,0004173	CANX
ENSGALG00000002119	6930,5852	-0,425364527	0,1209038	-3,518206	0,0004345	0,0026634	C16orf62
ENSGALG00000000033	3918,6949	-0,425866952	0,1266041	-3,363768	0,0007689	0,0043061	NFU1
ENSGALG00000004181	1761,4875	-0,425938272	0,1375166	-3,09736	0,0019525	0,0092529	GAA
ENSGALG00000016911	5511,4642	-0,426013919	0,1155064	-3,688227	0,0002258	0,0015183	RNF219
ENSGALG00000001229	3132,3073	-0,426291988	0,1628978	-2,616929	0,0088725	0,0319489	SKI
ENSGALG00000005205	5076,6373	-0,426364463	0,1335815	-3,191793	0,0014139	0,0071088	RNFT1
ENSGALG00000016955	4576,7081	-0,426960619	0,1306354	-3,268337	0,0010818	0,0057067	VWA8
ENSGALG00000008616	22133,522	-0,427012968	0,114208	-3,738907	0,0001848	0,0012773	GTF2H4
ENSGALG00000001181	1351,4114	-0,427049071	0,1574822	-2,711729	0,0066933	0,0255122	KCNJ5
ENSGALG00000004104	16560,152	-0,427862285	0,1288312	-3,321108	0,0008966	0,0048849	INTS1

ENSGALG00000002262	28425,435	-0,428265663	0,1046681	-4,091655	4,28E-05	0,0003598	ETF1
ENSGALG00000009145	1321,8829	-0,428291708	0,1635071	-2,619407	0,0088083	0,0317766	STARD9
ENSGALG00000012068	1109,9265	-0,428298706	0,1525081	-2,808367	0,0049793	0,0200099	ZGRF1
ENSGALG00000005164	1906,2497	-0,428309171	0,1642287	-2,608005	0,0091072	0,0325699	MTG2
ENSGALG00000004776	7278,2903	-0,428490556	0,1147583	-3,733852	0,0001886	0,0012986	AP4E1
ENSGALG00000012227	10485,474	-0,428492425	0,1091678	-3,92508	8,67E-05	0,000665	TSPAN5
ENSGALG00000017111	14958,655	-0,428517877	0,1112294	-3,852559	0,0001169	0,0008555	NUP58
ENSGALG00000008844	4547,7203	-0,428880605	0,1351184	-3,17411	0,001503	0,0074438	SLC31A1
ENSGALG00000023571	6639,2137	-0,428973189	0,1232423	-3,480731	0,0005	0,0030018	TRIM59
ENSGALG00000014953	10323,626	-0,429472364	0,1343816	-3,195916	0,0013939	0,0070213	RWDD1
ENSGALG00000016454	3850,1908	-0,429504414	0,1220362	-3,519484	0,0004324	0,0026566	E2F6
ENSGALG00000000346	4943,5232	-0,429865287	0,1169676	-3,675082	0,0002378	0,0015854	ZNF606
ENSGALG00000004133	28687,584	-0,430003398	0,1149324	-3,741359	0,000183	0,0012682	SRSF10
ENSGALG00000006730	17262,845	-0,430049755	0,1029557	-4,177038	2,95E-05	0,0002614	PM5
ENSGALG00000027835	5051,5612	-0,430117051	0,1151335	-3,735813	0,0001871	0,0012918	PCBD1
ENSGALG00000001501	5273,6643	-0,430190725	0,1139513	-3,775216	0,0001599	0,0011272	MAPK1
ENSGALG00000006500	13286,475	-0,430344251	0,1186807	-3,626068	0,0002878	0,0018755	NDE1
ENSGALG00000004986	14224,048	-0,430376609	0,1189456	-3,618265	0,0002966	0,0019238	CERCAM
ENSGALG00000004756	2781,9182	-0,43117271	0,1246958	-3,457796	0,0005446	0,0032218	CEP89
ENSGALG00000015184	7792,5962	-0,431383947	0,1228874	-3,510399	0,0004474	0,0027311	TLE4
ENSGALG00000007705	251805,66	-0,431935045	0,0988918	-4,367752	1,26E-05	0,0001257	NCL
ENSGALG00000011555	11516,332	-0,431962973	0,1142249	-3,781689	0,0001558	0,0011017	QKI
ENSGALG00000014222	26624,515	-0,431998929	0,0998402	-4,326904	1,51E-05	0,0001475	NUP50
ENSGALG00000011013	3992,5007	-0,43257266	0,1184225	-3,65279	0,0002594	0,0017112	PCNX2
ENSGALG00000015635	5954,0554	-0,432598021	0,1516977	-2,851712	0,0043485	0,0178433	HTT
ENSGALG00000008413	12232,739	-0,432788919	0,1151054	-3,759937	0,00017	0,0011928	PPP4R1
ENSGALG00000007481	6576,2725	-0,43296248	0,1091555	-3,966473	7,29E-05	0,0005708	HACD3
ENSGALG00000006378	6236,1912	-0,43312035	0,1311772	-3,301795	0,0009607	0,0051719	LIPA
ENSGALG00000013920	7232,4329	-0,433479079	0,1210408	-3,581264	0,0003419	0,0021744	SUV39H2
ENSGALG00000002877	3840,7613	-0,433897954	0,1361261	-3,187471	0,0014352	0,0071946	LRRC45
ENSGALG00000011548	3329,6306	-0,434202614	0,128113	-3,389215	0,0007009	0,003983	PPP1R13B
ENSGALG00000016279	1166,2207	-0,434455337	0,1634837	-2,657485	0,0078726	0,029087	RAB23
ENSGALG00000010976	5423,3364	-0,434636867	0,1246154	-3,487825	0,000487	0,0029336	CCDC126
ENSGALG00000009930	17651,917	-0,434701609	0,1010801	-4,300564	1,70E-05	0,000163	STRN3
ENSGALG00000006221	21172,656	-0,434757063	0,1020087	-4,261961	2,03E-05	0,0001895	KIF1A
ENSGALG00000028462	7479,587	-0,434862527	0,1269541	-3,425353	0,000614	0,0035679	USP36
ENSGALG00000008355	3052,8201	-0,434887931	0,1335972	-3,255217	0,0011331	0,0059333	CASP8
ENSGALG00000011543	9465,1404	-0,435034048	0,1235278	-3,521751	0,0004287	0,0026399	SDAD1
ENSGALG00000009519	11233,674	-0,43521067	0,1059425	-4,107988	3,99E-05	0,0003401	ARG2
ENSGALG00000009598	50606,56	-0,435652779	0,1207717	-3,607242	0,0003095	0,0019954	EPRS
ENSGALG00000006409	11343,942	-0,436770571	0,1258794	-3,469755	0,0005209	0,0031026	PODXL
ENSGALG00000028675	1287,5833	-0,436847257	0,1749597	-2,496845	0,0125304	0,0420822	
ENSGALG00000001717	40955,931	-0,437024576	0,1153453	-3,788837	0,0001514	0,0010755	SLC16A1
ENSGALG00000012680	2735,4526	-0,437301833	0,1231056	-3,552249	0,000382	0,0023844	E2F3
ENSGALG00000026093	981,75895	-0,437495765	0,1708286	-2,561021	0,0104365	0,0364061	PTPRN
ENSGALG00000011475	4517,3627	-0,437819648	0,1241287	-3,527143	0,0004201	0,0025938	SMARCAL1
ENSGALG00000014375	10299,481	-0,437825336	0,1079876	-4,054405	5,03E-05	0,0004135	
ENSGALG00000017038	6592,4492	-0,437833021	0,1366122	-3,204934	0,0013509	0,0068503	PROSER1
ENSGALG00000012642	14397,962	-0,437930988	0,1176937	-3,720938	0,0001985	0,0013557	RIC8B
ENSGALG00000008796	7379,0553	-0,438293488	0,1157441	-3,786746	0,0001526	0,0010829	RPF1
ENSGALG00000007999	6686,6947	-0,438325223	0,1112507	-3,939978	8,15E-05	0,0006287	MRPS7
ENSGALG00000009448	19270,632	-0,43880669	0,1070346	-4,099673	4,14E-05	0,0003505	IFRD1
ENSGALG00000008163	17633,406	-0,439350797	0,1262915	-3,478863	0,0005035	0,0030149	PSME4
ENSGALG00000004522	17438,777	-0,439397212	0,1129665	-3,889624	0,0001004	0,0007527	
ENSGALG00000005854	8969,9116	-0,439557852	0,1134785	-3,87349	0,0001073	0,0007966	CLUH
ENSGALG00000014870	12682,587	-0,439767118	0,1169979	-3,75876	0,0001708	0,0011965	HSF2
ENSGALG00000009731	11305,313	-0,439893222	0,106114	-4,145476	3,39E-05	0,000295	ARHGAP11A
ENSGALG00000006427	15012,164	-0,439960428	0,10348	-4,251647	2,12E-05	0,0001975	CARS
ENSGALG00000012497	3402,6563	-0,440007974	0,1212931	-3,627643	0,000286	0,001865	SLC12A7
ENSGALG00000022802	18215,83	-0,44018485	0,113024	-3,894614	9,84E-05	0,0007387	PWP2
ENSGALG00000000430	10996,849	-0,440297246	0,1104513	-3,986348	6,71E-05	0,0005299	SPPL2B
ENSGALG00000007145	45254,09	-0,440432711	0,0991199	-4,443434	8,85E-06	9,27E-05	ITGB1

ENSGALG00000002732	1764,9109	-0,440734535	0,1504889	-2,928684	0,003404	0,0146048	AKAP7
ENSGALG00000003071	6465,8929	-0,441229907	0,1183654	-3,727693	0,0001932	0,0013256	OGFOD1
ENSGALG00000015049	15802,948	-0,441268065	0,1109947	-3,975577	7,02E-05	0,0005529	AMD1
ENSGALG00000008988	11231,556	-0,442984401	0,1126159	-3,933587	8,37E-05	0,0006437	ZZZ3
ENSGALG00000006403	20700,703	-0,442987653	0,1077974	-4,109445	3,97E-05	0,0003388	MESDC2
ENSGALG00000010957	23588,305	-0,443064247	0,1020626	-4,341102	1,42E-05	0,0001402	USP1
ENSGALG00000004820	1103,092	-0,443483479	0,1622612	-2,733145	0,0062733	0,0241546	FAM57A
ENSGALG00000020879	1971,7439	-0,443502872	0,137058	-3,235877	0,0012127	0,0062731	SLC30A7
ENSGALG00000012696	9654,978	-0,443665071	0,1168806	-3,795883	0,0001471	0,0010476	RANBP9
ENSGALG00000012000	7157,9161	-0,444031788	0,1096112	-4,05097	5,10E-05	0,0004182	METTL14
ENSGALG00000005206	7037,81	-0,444288079	0,1130121	-3,931331	8,45E-05	0,0006489	ACSL3
ENSGALG00000003861	8032,1428	-0,444434747	0,1081924	-4,107819	3,99E-05	0,0003401	HERC4
ENSGALG00000005241	3696,959	-0,444802963	0,1260848	-3,527809	0,000419	0,0025884	DYNC1LI2
ENSGALG00000029011	22651,885	-0,444941401	0,1003035	-4,435953	9,17E-06	9,53E-05	SLC35F1
ENSGALG00000026971	4341,3764	-0,445130127	0,1193093	-3,730892	0,0001908	0,0013127	RRAGD
ENSGALG00000026753	1244,1641	-0,445460857	0,1753388	-2,540573	0,0110671	0,0381272	FSBP
ENSGALG00000007704	19378,595	-0,445645488	0,1426064	-3,125003	0,001778	0,008545	NPM3
ENSGALG00000010786	1210,4793	-0,44600197	0,1746834	-2,553203	0,0106737	0,0370627	DGKB
ENSGALG00000003400	9822,1857	-0,446090625	0,1073373	-4,155972	3,24E-05	0,0002836	AIMP2
ENSGALG00000009020	9304,4549	-0,446477137	0,1140153	-3,91594	9,01E-05	0,0006861	JAG1
ENSGALG00000000619	1429,9309	-0,446602215	0,1730494	-2,580779	0,0098578	0,0347614	ANGPTL4
ENSGALG00000013131	1864,5844	-0,447051346	0,1333424	-3,352657	0,0008004	0,0044479	C18orf21
ENSGALG00000002536	10005,2	-0,447408092	0,1148794	-3,894588	9,84E-05	0,0007387	STX2
ENSGALG00000002863	21477,092	-0,447764784	0,1264663	-3,540586	0,0003992	0,002482	ERGIC1
ENSGALG00000009645	4180,3141	-0,447866483	0,1704432	-2,627659	0,0085975	0,0311647	ESRRG
ENSGALG00000007437	4980,3271	-0,447866955	0,1129748	-3,96431	7,36E-05	0,0005753	DPP8
ENSGALG00000012914	8462,3227	-0,447972492	0,15259	-2,935792	0,003327	0,0143208	PRKDC
ENSGALG00000010612	2716,3259	-0,44797699	0,144448	-3,101302	0,0019267	0,0091476	PRKD3
ENSGALG00000013568	1779,8432	-0,448058074	0,1709275	-2,621333	0,0087587	0,0316302	NR4A3
ENSGALG00000016385	1906,4631	-0,448510168	0,1379255	-3,251829	0,0011467	0,0059985	TSSC1
ENSGALG00000000424	6758,6507	-0,448587625	0,1462417	-3,067441	0,002159	0,0100529	CMTM3
ENSGALG00000006656	4293,7935	-0,448668509	0,1620852	-2,768103	0,0056384	0,0221356	FRMD4A
ENSGALG00000011642	18424,345	-0,448893503	0,1458916	-3,076897	0,0020917	0,0098032	ANKRD17
ENSGALG00000001845	10040,892	-0,449444654	0,1070784	-4,197342	2,70E-05	0,0002425	PHTF1
ENSGALG00000012568	2033,2155	-0,449796718	0,1384814	-3,248067	0,0011619	0,0060519	TIMP3
ENSGALG00000006324	5540,5991	-0,450353768	0,1447725	-3,110768	0,001866	0,0088831	PFKFB3
ENSGALG00000012598	6595,5132	-0,450640697	0,1225301	-3,677795	0,0002353	0,0015709	NAA35
ENSGALG00000002519	3426,1294	-0,450983038	0,1241584	-3,632319	0,0002809	0,0018351	SLC25A33
ENSGALG00000007652	11015,319	-0,45121126	0,1103319	-4,089582	4,32E-05	0,0003619	SUCLG2
ENSGALG00000005959	3317,973	-0,451515508	0,1209724	-3,732383	0,0001897	0,0013056	GLMN
ENSGALG00000016803	4775,6071	-0,451555414	0,1329918	-3,395363	0,0006854	0,0039109	ST6GAL2
ENSGALG00000002663	8380,3175	-0,451591009	0,1168807	-3,863693	0,0001117	0,0008235	ATR
ENSGALG00000007075	11514,123	-0,451627608	0,1269002	-3,558919	0,0003724	0,0023386	ATP13A3
ENSGALG00000004049	11640,773	-0,45169714	0,107806	-4,189907	2,79E-05	0,0002495	ITFG1
ENSGALG00000013579	24869,718	-0,451701092	0,1629664	-2,771743	0,0055757	0,0219585	FAT1
ENSGALG00000013233	9834,1286	-0,4517759	0,1141463	-3,957866	7,56E-05	0,0005873	PPP1R9B
ENSGALG00000006336	7051,0846	-0,452052929	0,1247016	-3,625078	0,0002889	0,0018818	PAXIP1
ENSGALG00000007659	35189,399	-0,45246564	0,1027053	-4,405474	1,06E-05	0,0001081	HYOU1
ENSGALG00000008088	3813,1419	-0,452924609	0,1170567	-3,869275	0,0001092	0,0008088	ACSL4
ENSGALG00000004291	6511,6259	-0,453004371	0,1491475	-3,037292	0,0023871	0,01091	VPS13D
ENSGALG00000008848	8858,4601	-0,453382128	0,1083977	-4,182581	2,88E-05	0,0002563	SOX2
ENSGALG00000009835	14808,429	-0,453500739	0,1117239	-4,059121	4,93E-05	0,0004065	XPOT
ENSGALG00000023480	1643,2534	-0,453589407	0,1642924	-2,760867	0,0057648	0,0225023	FBXO39
ENSGALG00000015458	9174,0129	-0,453606012	0,1307234	-3,469967	0,0005205	0,0031015	ILDR2
ENSGALG00000005716	3689,9521	-0,453921562	0,1547747	-2,932789	0,0033593	0,0144372	SBF2
ENSGALG00000004971	14464,929	-0,454228028	0,152979	-2,969218	0,0029856	0,0131129	URM1
ENSGALG00000005840	6869,8394	-0,454270613	0,1158533	-3,921086	8,82E-05	0,0006743	DENND5A
ENSGALG00000016074	1091,7628	-0,454409142	0,1579184	-2,877493	0,0040085	0,0167153	RIMS2
ENSGALG00000006094	10694,318	-0,454736184	0,1116856	-4,071574	4,67E-05	0,000387	PHF6
ENSGALG00000004713	12581,373	-0,454819325	0,1044249	-4,355468	1,33E-05	0,0001321	LAS1L
ENSGALG00000005160	9338,8368	-0,454934871	0,1255105	-3,624675	0,0002893	0,001883	VMP1
ENSGALG00000021883	1337,969	-0,455471836	0,1467354	-3,104035	0,001909	0,0090783	

ENSGALG00000005489	5406,3342	-0,455551792	0,1294829	-3,518238	0,0004344	0,0026634	DDX52
ENSGALG00000016196	5946,1092	-0,455745009	0,1321926	-3,447582	0,0005656	0,0033251	CBS
ENSGALG00000011191	11563,883	-0,455971006	0,1072313	-4,252219	2,12E-05	0,0001972	EML1
ENSGALG00000008839	9872,7485	-0,456849267	0,1286669	-3,550635	0,0003843	0,0023968	ATP11B
ENSGALG00000008415	6540,8044	-0,456904989	0,1186805	-3,849874	0,0001182	0,0008637	ALS2
ENSGALG00000000884	7194,478	-0,456928071	0,1125409	-4,060106	4,91E-05	0,000405	CXXC5
ENSGALG00000021592	966,74998	-0,457097931	0,1634651	-2,796303	0,0051691	0,0206083	TLCD2
ENSGALG00000002338	29369,786	-0,457290151	0,1429103	-3,199841	0,001375	0,0069517	ASPM
ENSGALG00000001179	4301,5313	-0,45735712	0,1154017	-3,963175	7,40E-05	0,0005777	ARMC8
ENSGALG00000023193	1113,686	-0,457481355	0,1527665	-2,994644	0,0027477	0,0122653	SOCS1B
ENSGALG00000002954	6734,124	-0,457794297	0,1205807	-3,79658	0,0001467	0,0010458	MBTD1
ENSGALG00000008497	15581,475	-0,457962816	0,1156674	-3,959307	7,52E-05	0,0005841	SLC38A2
ENSGALG00000007777	3900,1787	-0,45814234	0,1287478	-3,558449	0,0003731	0,0023406	SLC39A10
ENSGALG00000026322	5686,9342	-0,458344915	0,1182055	-3,877526	0,0001055	0,0007877	ARMT1
ENSGALG00000013647	1849,9954	-0,458404091	0,1358218	-3,375041	0,000738	0,004154	TFB1M
ENSGALG00000026639	1529,3689	-0,458562471	0,139721	-3,281988	0,0010308	0,0054735	SMCO4
ENSGALG00000004531	7125,0235	-0,458962037	0,1117417	-4,107348	4,00E-05	0,0003406	PLOD1
ENSGALG00000007842	5019,27	-0,459124954	0,1174579	-3,908847	9,27E-05	0,0007038	PDHX
ENSGALG00000016930	13372,763	-0,459461549	0,1064549	-4,316019	1,59E-05	0,0001534	DIS3
ENSGALG00000008705	14944,012	-0,4597037	0,1096313	-4,193181	2,75E-05	0,0002462	TRABD
ENSGALG00000015773	9016,2735	-0,459782065	0,1064627	-4,318717	1,57E-05	0,0001521	PTCD3
ENSGALG00000008482	22772,22	-0,459855147	0,1105258	-4,160612	3,17E-05	0,000279	STAG2
ENSGALG00000013406	738,83887	-0,460037256	0,1721459	-2,672369	0,0075318	0,0280715	EOGT
ENSGALG00000010411	14781,22	-0,460452211	0,1181671	-3,896619	9,75E-05	0,000735	RNF13
ENSGALG00000002501	3377,4292	-0,46100497	0,1227582	-3,755392	0,0001731	0,0012109	RP11-196G11.1
ENSGALG00000006281	5170,2004	-0,461106682	0,1148963	-4,013241	5,99E-05	0,0004808	GTF2H1
ENSGALG00000006078	2849,4853	-0,461126659	0,1296477	-3,556767	0,0003754	0,0023524	PALB2
ENSGALG00000016216	6550,317	-0,461519219	0,1642494	-2,809869	0,0049562	0,0199462	NDRG1
ENSGALG00000013893	1508,8689	-0,461829974	0,1439203	-3,208929	0,0013323	0,0067735	MNS1
ENSGALG00000015633	9547,4016	-0,462141421	0,1057703	-4,369294	1,25E-05	0,0001252	TERF1
ENSGALG00000016980	3122,8167	-0,462357479	0,1258227	-3,674674	0,0002382	0,0015871	COG3
ENSGALG00000013136	2283,1481	-0,462408191	0,131854	-3,506971	0,0004532	0,0027598	AEBP2
ENSGALG00000000687	3686,837	-0,462411333	0,1264523	-3,656806	0,0002554	0,0016878	KIAA0556
ENSGALG00000013306	1859,3469	-0,463147904	0,1696975	-2,729256	0,0063477	0,0243862	DAGLA
ENSGALG00000016445	7095,5905	-0,463408352	0,1140354	-4,063725	4,83E-05	0,000399	NOL10
ENSGALG00000009899	35232,794	-0,46345514	0,1085537	-4,269364	1,96E-05	0,0001844	CAND1
ENSGALG00000009974	634,62537	-0,463813865	0,1807038	-2,566708	0,0102669	0,0359195	MMAA
ENSGALG00000011794	10368,91	-0,463824908	0,1147491	-4,042078	5,30E-05	0,0004329	DDX47
ENSGALG00000007057	3861,2618	-0,46473909	0,1208896	-3,844327	0,0001209	0,0008806	LRRC28
ENSGALG00000010234	635,90171	-0,464990416	0,1851223	-2,511801	0,0120117	0,0406308	MMACHC
ENSGALG00000012655	11727,465	-0,4651119	0,1097394	-4,23833	2,25E-05	0,0002077	POLR3B
ENSGALG00000011630	13721,826	-0,465130144	0,1774121	-2,62175	0,008748	0,0316092	GLI2
ENSGALG00000015138	14137,444	-0,465157078	0,1241857	-3,745659	0,0001799	0,0012504	TMEM2
ENSGALG00000009507	1700,6819	-0,465226456	0,1787644	-2,602456	0,0092559	0,0330238	PLEKHH1
ENSGALG00000011677	985,1206	-0,465468876	0,1657897	-2,807586	0,0049914	0,0200413	HSPBAP1
ENSGALG00000002942	14672,633	-0,465828236	0,1343592	-3,467036	0,0005262	0,0031314	JMJD1C
ENSGALG00000014217	2291,1249	-0,46602463	0,1389942	-3,352834	0,0007999	0,0044469	GUF1
ENSGALG00000010054	3266,3331	-0,466429533	0,1270851	-3,670215	0,0002423	0,0016127	RP11-561B11.2
ENSGALG00000004158	2874,2398	-0,467330867	0,1229214	-3,801867	0,0001436	0,0010258	GPT2
ENSGALG00000028849	26828,63	-0,467375912	0,1168899	-3,99843	6,38E-05	0,0005068	SCAF11
ENSGALG00000011309	5857,3376	-0,467663025	0,1226263	-3,813725	0,0001369	0,0009866	PLXNC1
ENSGALG00000013728	12921,155	-0,467853342	0,1080614	-4,329513	1,49E-05	0,0001465	PPAT
ENSGALG00000013765	8392,681	-0,468026469	0,1155365	-4,050895	5,10E-05	0,0004182	RP3-468K18.7
ENSGALG00000002282	2493,1225	-0,468163907	0,1304601	-3,588561	0,0003325	0,0021254	TMEM200A
ENSGALG00000000895	27062,291	-0,468166268	0,1101353	-4,250829	2,13E-05	0,0001979	NUDC
ENSGALG00000017309	912,73532	-0,468669212	0,1805876	-2,595246	0,0094523	0,0335839	RNF169
ENSGALG00000002655	6693,9795	-0,468798244	0,1325586	-3,536536	0,0004054	0,0025147	ITGAV
ENSGALG00000006121	11011,198	-0,468808823	0,116573	-4,021589	5,78E-05	0,0004665	PIK3C2A
ENSGALG00000011232	2855,7622	-0,469477808	0,1239226	-3,788475	0,0001516	0,0010765	STK11IP
ENSGALG00000006638	2375,8984	-0,469621749	0,1392735	-3,37194	0,0007464	0,0041941	GRIK4
ENSGALG00000009954	1375,372	-0,469791805	0,1450112	-3,239694	0,0011966	0,0062039	DYNC2LI1
ENSGALG00000007058	9479,7297	-0,469857249	0,1077974	-4,358706	1,31E-05	0,0001303	LSG1

ENSGALG00000011101	5741,1035	-0,47005983	0,1114966	-4,215912	2,49E-05	0,0002267	URB2
ENSGALG00000001424	896,527	-0,470247	0,1654153	-2,842827	0,0044715	0,0182515	YDJC
ENSGALG00000010434	945,24487	-0,470849898	0,1876053	-2,509791	0,0120803	0,0407919	CP
ENSGALG00000000843	7211,6886	-0,471124949	0,1430863	-3,292592	0,0009927	0,0053002	DOT1L
ENSGALG00000011246	5026,827	-0,471179653	0,1165719	-4,041966	5,30E-05	0,0004329	CHPF
ENSGALG00000007564	1255,8688	-0,471313446	0,1490623	-3,161856	0,0015677	0,0077305	
ENSGALG00000011537	2857,0805	-0,471339277	0,1817158	-2,593827	0,0094914	0,0336858	PDE10A
ENSGALG00000016836	6379,6617	-0,471449962	0,1187504	-3,970091	7,18E-05	0,0005636	TUBGCP3
ENSGALG00000002439	1784,311	-0,471657703	0,1533679	-3,075335	0,0021027	0,0098513	BDP1
ENSGALG00000000721	4145,8062	-0,47173717	0,1427965	-3,303562	0,0009546	0,0051475	SLC41A1
ENSGALG00000008837	13730,848	-0,471745405	0,1080446	-4,366209	1,26E-05	0,0001264	PELI1
ENSGALG00000026412	3599,375	-0,471881516	0,1379941	-3,419578	0,0006272	0,0036213	
ENSGALG00000016195	2664,1039	-0,472549861	0,1405226	-3,362804	0,0007716	0,0043194	COL22A1
ENSGALG00000004228	2475,7819	-0,472593441	0,1261151	-3,747317	0,0001787	0,0012448	TRAF3IP1
ENSGALG00000012647	9993,6453	-0,472701385	0,125933	-3,753594	0,0001743	0,0012177	RFX4
ENSGALG00000001627	4772,9003	-0,472962819	0,1154681	-4,096047	4,20E-05	0,0003545	CPNE1
ENSGALG00000026192	2515,1802	-0,473341595	0,1470736	-3,218399	0,0012891	0,0066005	ST8SIA4
ENSGALG00000028267	15487,152	-0,473386218	0,114594	-4,130988	3,61E-05	0,0003117	GOLPH3
ENSGALG00000006296	11149,265	-0,47366212	0,1093498	-4,331622	1,48E-05	0,0001453	SON
ENSGALG00000014903	1822,8102	-0,474039661	0,1650108	-2,87278	0,0040688	0,0169304	ITGA2
ENSGALG00000004055	3994,9616	-0,474043343	0,1194933	-3,967113	7,27E-05	0,0005696	C7orf50
ENSGALG00000027678	2192,33	-0,474178987	0,1641393	-2,888881	0,0038662	0,0162692	SLC35G1
ENSGALG00000027440	2934,027	-0,474361212	0,127321	-3,725711	0,0001948	0,0013332	COX20
ENSGALG00000004889	8112,9525	-0,474641509	0,1093142	-4,341993	1,41E-05	0,0001397	SLC2A1
ENSGALG00000005211	6813,7998	-0,474686708	0,1155357	-4,108572	3,98E-05	0,0003396	NAE1
ENSGALG00000026034	1070,3788	-0,47474594	0,1567205	-3,029253	0,0024516	0,0111484	RGS17
ENSGALG00000010182	7219,0566	-0,474781283	0,1123939	-4,224264	2,40E-05	0,0002195	SLC29A1
ENSGALG00000015925	3585,1162	-0,475263897	0,1282784	-3,704941	0,0002114	0,0014344	MB21D1
ENSGALG00000026989	16347,278	-0,47543634	0,1239145	-3,83681	0,0001246	0,0009056	
ENSGALG00000006698	967,37858	-0,475588456	0,1653212	-2,876753	0,0040179	0,0167494	ABCC6
ENSGALG00000002783	1895,8368	-0,475653855	0,1505722	-3,158976	0,0015832	0,0077802	SLC43A2
ENSGALG00000016409	21993,092	-0,475709899	0,1119047	-4,251027	2,13E-05	0,0001979	KIDINS220
ENSGALG00000004957	935,39904	-0,476296229	0,1744085	-2,730924	0,0063157	0,024288	RHBDD1
ENSGALG00000008686	2315,3907	-0,476496754	0,1292591	-3,686368	0,0002275	0,0015287	TRDMT1
ENSGALG00000006906	7509,2168	-0,477103418	0,1079356	-4,420261	9,86E-06	0,0001016	CHM
ENSGALG00000009841	1397,5061	-0,477667905	0,1552355	-3,077053	0,0020906	0,0098032	FASTKD1
ENSGALG00000011342	3208,6738	-0,478233256	0,1425056	-3,355892	0,0007911	0,0044106	FGD6
ENSGALG00000002045	30343,402	-0,478318973	0,0998188	-4,791874	1,65E-06	2,05E-05	EIF2S2
ENSGALG00000002460	17010	-0,480154381	0,105142	-4,566722	4,95E-06	5,50E-05	WDR75
ENSGALG00000002862	5434,2601	-0,480739106	0,1110641	-4,328483	1,50E-05	0,0001471	RRN3
ENSGALG00000012484	32855,96	-0,4809575	0,1211628	-3,969513	7,20E-05	0,0005645	RIF1
ENSGALG00000010884	11252,816	-0,481115067	0,1148894	-4,187635	2,82E-05	0,0002518	DDX24
ENSGALG00000015901	15729,886	-0,481643321	0,1054274	-4,568482	4,91E-06	5,46E-05	SENP6
ENSGALG00000023516	18924,681	-0,48179177	0,1052391	-4,578068	4,69E-06	5,25E-05	PHRF1
ENSGALG00000002294	3404,1182	-0,482168758	0,1282462	-3,759711	0,0001701	0,0011932	SEMA6A
ENSGALG00000007948	1369,1179	-0,482500979	0,1814408	-2,659276	0,0078309	0,0289722	LRRC4C
ENSGALG00000001612	1053,9675	-0,482944134	0,1589083	-3,039137	0,0023726	0,0108507	HEXDC
ENSGALG00000003078	20488,019	-0,483195985	0,106301	-4,545546	5,48E-06	5,97E-05	MMD
ENSGALG00000008765	5344,7574	-0,483352119	0,1278807	-3,779711	0,000157	0,0011099	C1D
ENSGALG00000005134	3683,9209	-0,483437706	0,1457126	-3,317748	0,0009075	0,0049361	ZSWIM8
ENSGALG00000000569	10192,548	-0,483580181	0,1749621	-2,763914	0,0057113	0,0223253	SDC3
ENSGALG00000009967	51604,136	-0,483675694	0,1030754	-4,692447	2,70E-06	3,17E-05	LRPPRC
ENSGALG00000012702	17854,641	-0,483830731	0,1194258	-4,051309	5,09E-05	0,000418	JARID2
ENSGALG00000015830	24016,122	-0,484314141	0,1030076	-4,70173	2,58E-06	3,04E-05	SYNCRIP
ENSGALG00000004887	17300,511	-0,484420703	0,1068743	-4,532622	5,83E-06	6,30E-05	SART3
ENSGALG00000002781	17608,172	-0,484707648	0,1272601	-3,808796	0,0001396	0,0010028	BRCA1
ENSGALG00000001950	14346,845	-0,484721287	0,11288	-4,294128	1,75E-05	0,0001671	PMPCA
ENSGALG00000001777	2307,3102	-0,484938734	0,1469529	-3,299961	0,000967	0,0051935	MFSD11
ENSGALG00000002198	1337,3607	-0,485078535	0,1489905	-3,255768	0,0011309	0,0059241	FHOD1
ENSGALG00000004416	1683,2285	-0,485858255	0,141996	-3,421634	0,0006225	0,0035994	TTC19
ENSGALG00000012258	6299,7876	-0,485912726	0,1153331	-4,213123	2,52E-05	0,0002288	GOLGA4
ENSGALG00000013745	3956,926	-0,486562498	0,1295758	-3,755041	0,0001733	0,0012113	RTTN

ENSGALG00000008862	12336,53	-0,486712741	0,1063335	-4,57723	4,71E-06	5,26E-05	DNAJC10
ENSGALG00000008670	2394,2538	-0,486758564	0,1345509	-3,617654	0,0002973	0,0019265	C1orf52
ENSGALG00000009157	10495,932	-0,48696748	0,111678	-4,36046	1,30E-05	0,0001295	ECT2
ENSGALG00000002906	23481,039	-0,487686151	0,1087191	-4,485745	7,27E-06	7,71E-05	TARDBP
ENSGALG00000008155	684,45488	-0,487898949	0,1882495	-2,591767	0,0095484	0,0338282	KCTD18
ENSGALG00000005198	7882,4671	-0,488262054	0,1163467	-4,196612	2,71E-05	0,0002432	RPS6KB1
ENSGALG00000004750	7577,7666	-0,488295007	0,1092829	-4,468172	7,89E-06	8,32E-05	NOL8
ENSGALG00000010570	52092,719	-0,488515596	0,1005153	-4,860111	1,17E-06	1,51E-05	BTF3L4
ENSGALG00000016909	4324,6991	-0,488526858	0,1162572	-4,202121	2,64E-05	0,0002386	NDFIP2
ENSGALG00000004638	3370,7614	-0,48870476	0,1414816	-3,454194	0,0005519	0,0032587	HEPH
ENSGALG00000001036	9684,7005	-0,488799588	0,1105545	-4,421345	9,81E-06	0,0001012	ELAC2
ENSGALG00000009981	1405,745	-0,49003231	0,1465298	-3,34425	0,0008251	0,004546	PREPL
ENSGALG00000010638	13156,313	-0,49033693	0,1208756	-4,056541	4,98E-05	0,0004107	CASP3
ENSGALG00000006252	14647,749	-0,49037706	0,1393987	-3,517803	0,0004351	0,0026662	LMO4
ENSGALG00000000272	5267,8402	-0,490419343	0,1354436	-3,620838	0,0002936	0,0019059	CDCA2
ENSGALG00000008553	5356,6273	-0,490452503	0,1346372	-3,642771	0,0002697	0,0017672	INO80
ENSGALG00000010013	1321,1558	-0,490665695	0,1680223	-2,920241	0,0034976	0,0149466	EDNRA
ENSGALG00000000367	793,50452	-0,492073862	0,2010375	-2,447672	0,0143783	0,0467543	ASIC2
ENSGALG00000016404	5806,1629	-0,492286998	0,1192985	-4,126516	3,68E-05	0,0003166	MTBP
ENSGALG00000010335	1726,889	-0,492481433	0,1437148	-3,426798	0,0006107	0,0035505	PIK3R3
ENSGALG00000002141	14007,058	-0,492600599	0,1062141	-4,637807	3,52E-06	4,03E-05	UGGT1
ENSGALG00000008871	16063,59	-0,492812674	0,1349508	-3,651796	0,0002604	0,0017161	LPHN2
ENSGALG00000015059	7575,55	-0,49315511	0,1131114	-4,359906	1,30E-05	0,0001297	
ENSGALG00000005652	5954,5779	-0,493244478	0,113265	-4,354781	1,33E-05	0,0001323	DIDO1
ENSGALG00000014218	876,2756	-0,493463947	0,1862713	-2,649168	0,008069	0,0296038	PHF21B
ENSGALG00000023576	3299,2992	-0,493564294	0,1207816	-4,086419	4,38E-05	0,0003657	PIM3
ENSGALG00000008798	4574,7649	-0,493864419	0,1182978	-4,174757	2,98E-05	0,0002639	ZMYND19
ENSGALG00000020742	13114,145	-0,493871262	0,1393963	-3,54293	0,0003957	0,0024612	PPRC1
ENSGALG00000011962	7036,6211	-0,494326456	0,1348846	-3,664809	0,0002475	0,0016447	NDNF
ENSGALG00000015478	5554,5683	-0,494614804	0,1302844	-3,796423	0,0001468	0,0010459	USP45
ENSGALG00000007326	7076,3591	-0,494698301	0,1094474	-4,519964	6,19E-06	6,66E-05	PMM2
ENSGALG00000005683	5447,0767	-0,494902817	0,117901	-4,197614	2,70E-05	0,0002424	ARHGAP29
ENSGALG00000010960	1065,9668	-0,495730596	0,183425	-2,702634	0,0068792	0,0260314	GALNT3
ENSGALG00000000795	1504,9365	-0,495778483	0,1425043	-3,479042	0,0005032	0,0030142	MFSD12
ENSGALG00000004273	11441,37	-0,495936282	0,1511882	-3,280258	0,0010371	0,005505	RALGPS2
ENSGALG00000014998	1987,961	-0,495971981	0,1542046	-3,216325	0,0012984	0,0066435	CABLES1
ENSGALG00000015684	4408,6888	-0,496145153	0,1139834	-4,352783	1,34E-05	0,0001334	DNAJC25
ENSGALG00000004570	5026,7973	-0,496270556	0,1351131	-3,673	0,0002397	0,001596	NCOA3
ENSGALG00000013257	123007,22	-0,496798558	0,1026267	-4,840832	1,29E-06	1,64E-05	LDHB
ENSGALG00000027635	9027,1813	-0,49702612	0,1189634	-4,177976	2,94E-05	0,0002609	NME4
ENSGALG00000005583	1029,2928	-0,497171251	0,1674424	-2,969208	0,0029857	0,0131129	ALG14
ENSGALG00000003596	25796,912	-0,497856616	0,162082	-3,071634	0,0021289	0,0099471	TRRAP
ENSGALG00000010624	9648,3759	-0,498764877	0,1086145	-4,592066	4,39E-06	4,91E-05	MEMO1
ENSGALG00000026865	1245,9917	-0,49909443	0,1619603	-3,081585	0,002059	0,0096734	XRCC2
ENSGALG00000007691	3206,9135	-0,499115113	0,1294529	-3,855572	0,0001155	0,0008465	ARMC9
ENSGALG00000007155	2000,5013	-0,499370494	0,1491539	-3,348022	0,0008139	0,0044973	RMI2
ENSGALG00000003874	8943,2552	-0,499635075	0,1097884	-4,550893	5,34E-06	5,87E-05	HEATR3
ENSGALG00000005695	3266,3031	-0,500082186	0,1314863	-3,803302	0,0001428	0,0010204	CHEK2
ENSGALG00000025792	3188,1064	-0,500292681	0,1218494	-4,105829	4,03E-05	0,0003426	FRRS1L
ENSGALG00000011264	1925,3077	-0,500518992	0,1479576	-3,382854	0,0007174	0,0040628	SULF1
ENSGALG00000016758	1465,7896	-0,5005603	0,1465611	-3,415369	0,000637	0,0036653	LIPT1
ENSGALG00000010716	5501,7296	-0,50085931	0,1188557	-4,214011	2,51E-05	0,0002282	AGA
ENSGALG00000009269	3597,5456	-0,501724842	0,1302073	-3,853278	0,0001165	0,000854	RP5-1021I20.4
ENSGALG00000011857	24467,231	-0,502240381	0,105172	-4,775418	1,79E-06	2,19E-05	LUC7L2
ENSGALG00000003719	6864,0435	-0,502933526	0,135236	-3,718932	0,0002001	0,0013633	CHD9
ENSGALG00000024035	1950,9384	-0,503656541	0,1918019	-2,625921	0,0086415	0,0312743	EPS8L2
ENSGALG00000006524	12378,285	-0,503792354	0,1077015	-4,677671	2,90E-06	3,38E-05	NOC3L
ENSGALG00000016781	17546,507	-0,504088725	0,1248435	-4,037765	5,40E-05	0,0004399	MAP4K4
ENSGALG00000014922	15076,997	-0,504425665	0,118689	-4,249979	2,14E-05	0,0001986	ROCK1
ENSGALG00000004690	52340,48	-0,504461763	0,1007635	-5,006396	5,55E-07	7,63E-06	MAPK6
ENSGALG00000002728	6868,7758	-0,504726866	0,1233642	-4,091357	4,29E-05	0,0003598	SLC16A3
ENSGALG00000011877	4219,7935	-0,504776099	0,1219876	-4,137928	3,50E-05	0,0003041	LARS2

ENSGALG00000005533	4875,7726	-0,504976403	0,1222357	-4,131171	3,61E-05	0,0003116	ZMYM3
ENSGALG00000013209	5984,6391	-0,505418476	0,1115613	-4,530409	5,89E-06	6,36E-05	SLC39A6
ENSGALG00000015252	4947,9442	-0,505578034	0,1423502	-3,551649	0,0003828	0,0023887	ST3GAL6
ENSGALG00000007234	4564,3675	-0,505755971	0,117953	-4,287774	1,80E-05	0,000171	CLCN5
ENSGALG00000010034	2578,0952	-0,50624459	0,1322184	-3,828852	0,0001287	0,0009324	
ENSGALG00000009556	2369,7227	-0,506316289	0,2035819	-2,487039	0,0128811	0,0429423	PRICKLE1
ENSGALG00000003540	2816,5136	-0,506324474	0,1426366	-3,549751	0,0003856	0,0024027	CEP95
ENSGALG00000005992	1391,4533	-0,506625581	0,1532979	-3,304844	0,0009503	0,0051298	PDE8A
ENSGALG00000000271	3107,1088	-0,506687982	0,1663878	-3,045222	0,0023251	0,0106766	C2CD2L
ENSGALG00000006935	12264,183	-0,506724591	0,1094246	-4,630812	3,64E-06	4,15E-05	BTAF1
ENSGALG00000019867	6166,9755	-0,506841982	0,1119268	-4,528335	5,95E-06	6,41E-05	WDCP
ENSGALG00000008420	3322,248	-0,507225725	0,1221905	-4,151106	3,31E-05	0,0002888	CAND2
ENSGALG00000012020	2468,0174	-0,507245855	0,184003	-2,756726	0,0058383	0,0227414	KIAA1549L
ENSGALG00000009782	45976,854	-0,507579083	0,1062698	-4,776324	1,79E-06	2,18E-05	NAA15
ENSGALG00000004118	1623,6627	-0,508229667	0,1686061	-3,014301	0,0025757	0,0115852	
ENSGALG00000002424	2020,6569	-0,508419582	0,160293	-3,171813	0,0015149	0,0074947	RECQL5
ENSGALG00000002652	8163,3179	-0,509052755	0,1365252	-3,728635	0,0001925	0,0013231	FZD10
ENSGALG00000005509	6460,5427	-0,509428544	0,1427708	-3,568157	0,0003595	0,0022671	DPYD
ENSGALG00000004990	1688,4348	-0,509590179	0,1471207	-3,463756	0,0005327	0,0031615	C10orf11
ENSGALG00000002505	458,85789	-0,509844408	0,2099041	-2,42894	0,015143	0,0487175	
ENSGALG00000010735	16013,937	-0,510060882	0,1046006	-4,876271	1,08E-06	1,40E-05	NDC1
ENSGALG00000003628	14552,598	-0,510073233	0,1414317	-3,6065	0,0003104	0,0019979	TLN2
ENSGALG00000015787	3935,8112	-0,510253054	0,1410404	-3,617779	0,0002971	0,0019265	RNGTT
ENSGALG00000008778	6210,2201	-0,51039033	0,1491907	-3,421059	0,0006238	0,0036047	SPRED2
ENSGALG00000012135	48183,581	-0,511735314	0,1079748	-4,739395	2,14E-06	2,56E-05	ATF4
ENSGALG00000017085	4116,9494	-0,511832688	0,125074	-4,092239	4,27E-05	0,0003593	SLC7A1
ENSGALG00000002709	2938,4248	-0,511853662	0,1402453	-3,649703	0,0002625	0,0017285	MYO1C
ENSGALG00000004621	8629,8848	-0,512109985	0,1230296	-4,162493	3,15E-05	0,0002769	PREX1
ENSGALG00000005278	9525,2437	-0,512823817	0,1583471	-3,238605	0,0012012	0,0062229	CDH11
ENSGALG00000014791	22071,934	-0,513191503	0,1237594	-4,146686	3,37E-05	0,0002936	WDR3
ENSGALG00000009825	16041,569	-0,51401966	0,1105113	-4,651284	3,30E-06	3,80E-05	SRGAP1
ENSGALG00000001441	10679	-0,514329303	0,1052351	-4,88743	1,02E-06	1,33E-05	UBE2G1
ENSGALG00000026468	7333,857	-0,514962132	0,1096475	-4,696525	2,65E-06	3,11E-05	CHSY1
ENSGALG00000002646	11173,638	-0,515104787	0,1477459	-3,486422	0,0004895	0,0029478	FAM171B
ENSGALG00000005653	3018,7755	-0,515161656	0,1231259	-4,184022	2,86E-05	0,0002552	NFKB2
ENSGALG00000011707	2975,9589	-0,516018775	0,1353146	-3,813473	0,000137	0,0009866	UBA5
ENSGALG00000003566	2456,1483	-0,516245691	0,1356954	-3,804444	0,0001421	0,0010168	SMURF2
ENSGALG00000003047	12574,653	-0,516252465	0,132068	-3,908991	9,27E-05	0,0007038	SUGP2
ENSGALG00000009784	1512,522	-0,516604655	0,1915407	-2,697101	0,0069946	0,0263872	PTPN14
ENSGALG00000006830	714,61116	-0,51666647	0,1768872	-2,920882	0,0034904	0,0149206	CHID1
ENSGALG00000007527	4564,3635	-0,516973735	0,1183481	-4,368248	1,25E-05	0,0001255	ADARB1
ENSGALG00000012550	2418,3256	-0,517745286	0,1390423	-3,723653	0,0001964	0,0013434	HMOX1
ENSGALG00000009207	1636,7589	-0,518081559	0,1511771	-3,426985	0,0006103	0,0035496	TRIM2
ENSGALG00000012207	4193,9067	-0,518404651	0,1155257	-4,487352	7,21E-06	7,66E-05	CCNT2
ENSGALG00000009451	10542,681	-0,518444547	0,1054026	-4,918707	8,71E-07	1,14E-05	PPID
ENSGALG00000010189	6061,8235	-0,518843565	0,1108188	-4,681909	2,84E-06	3,32E-05	RP11-407N17.3
ENSGALG00000011596	8210,1595	-0,519655791	0,1247256	-4,166392	3,09E-05	0,0002731	PTPN4
ENSGALG00000006622	2389,7433	-0,520113735	0,1331747	-3,9055	9,40E-05	0,0007116	FRA10AC1
ENSGALG00000014784	4045,4061	-0,520975246	0,1405154	-3,707602	0,0002092	0,0014201	MAST4
ENSGALG00000009992	11680,473	-0,521017168	0,1135606	-4,588008	4,47E-06	5,01E-05	ARHGAP5
ENSGALG00000015889	13285,71	-0,52134607	0,1484689	-3,511482	0,0004456	0,0027219	PHIP
ENSGALG00000007752	6790,4614	-0,521363763	0,1083691	-4,811	1,50E-06	1,88E-05	COPS7B
ENSGALG00000005850	15672,107	-0,521743975	0,1094822	-4,765559	1,88E-06	2,29E-05	FNBP1L
ENSGALG00000012354	2395,6585	-0,521764337	0,1590822	-3,27984	0,0010387	0,005511	VOPP1
ENSGALG00000015314	12934,444	-0,521997148	0,1092952	-4,77603	1,79E-06	2,19E-05	BEND3
ENSGALG00000001109	1011,8383	-0,522206198	0,1701303	-3,069449	0,0021445	0,0100027	GTSF1
ENSGALG00000004397	3452,7405	-0,523035736	0,1275508	-4,100607	4,12E-05	0,0003498	MLXIP
ENSGALG00000012592	3142,2738	-0,523397807	0,1406662	-3,720849	0,0001986	0,0013557	RMI1
ENSGALG00000006811	8467,8095	-0,523451296	0,1844356	-2,838125	0,0045379	0,0184744	ZIC1
ENSGALG00000002487	10163,363	-0,523537895	0,1313011	-3,987308	6,68E-05	0,0005281	SFSWAP
ENSGALG00000003260	9438,4125	-0,523953501	0,1105482	-4,739594	2,14E-06	2,56E-05	UTP6
ENSGALG00000012720	24474,404	-0,524679114	0,1115414	-4,703897	2,55E-06	3,01E-05	NUP153

ENSGALG00000005425	38874,994	-0,524696803	0,1183507	-4,433407	9,28E-06	9,63E-05	OGT
ENSGALG00000010861	2392,2986	-0,524736464	0,1328216	-3,950687	7,79E-05	0,0006035	OMA1
ENSGALG00000004489	1483,1995	-0,524803254	0,1455768	-3,604991	0,0003122	0,0020076	
ENSGALG00000011770	14710,919	-0,525890593	0,1188458	-4,424984	9,64E-06	9,98E-05	UMPS
ENSGALG00000002850	6570,4074	-0,526275555	0,1347444	-3,905733	9,39E-05	0,0007113	RASA2
ENSGALG00000023562	1492,3921	-0,526424205	0,1608921	-3,271908	0,0010682	0,0056493	TEX9
ENSGALG00000015836	3297,8542	-0,526441345	0,132466	-3,974163	7,06E-05	0,0005557	CEP162
ENSGALG00000014426	14003,05	-0,526522361	0,1207296	-4,361172	1,29E-05	0,0001291	KPNA1
ENSGALG00000009172	566,03742	-0,527120334	0,2081817	-2,532021	0,0113407	0,0388242	OSBPL6
ENSGALG00000011982	5512,8717	-0,527666168	0,1403594	-3,759392	0,0001703	0,0011941	PDE5A
ENSGALG00000011149	879,0742	-0,527681437	0,1626198	-3,244879	0,001175	0,0061037	PLA2R1
ENSGALG00000008096	6070,9895	-0,528190931	0,1247684	-4,233371	2,30E-05	0,0002114	SUFU
ENSGALG00000007140	3712,2899	-0,528407251	0,1869729	-2,826116	0,0047116	0,0190805	NRP1
ENSGALG00000008847	3493,1884	-0,528756478	0,1263169	-4,185952	2,84E-05	0,0002534	VPS54
ENSGALG00000013177	2545,0183	-0,528877548	0,1412318	-3,744749	0,0001806	0,0012537	BCAT1
ENSGALG00000017392	1036,5696	-0,528892969	0,1585879	-3,335014	0,000853	0,0046807	SPDYA
ENSGALG00000012979	10526,261	-0,528975634	0,1480989	-3,571772	0,0003546	0,0022422	TRIO
ENSGALG00000000208	3421,1766	-0,529383362	0,1352035	-3,915457	9,02E-05	0,0006871	MCC
ENSGALG00000004474	1133,2959	-0,529777367	0,1534298	-3,452897	0,0005546	0,0032709	PLEKHF1
ENSGALG00000013203	7478,1862	-0,52983334	0,1163226	-4,554862	5,24E-06	5,78E-05	ELP2
ENSGALG00000011419	3053,0195	-0,529859697	0,123662	-4,284741	1,83E-05	0,0001731	DUSP4
ENSGALG00000010159	4422,5959	-0,529906457	0,122307	-4,332595	1,47E-05	0,0001449	SLC20A2
ENSGALG00000002974	1015,7762	-0,529999669	0,168327	-3,148631	0,0016404	0,0080164	SPHKAP
ENSGALG00000002624	31172,832	-0,530323004	0,1123984	-4,718243	2,38E-06	2,82E-05	BMS1
ENSGALG00000006826	4092,1467	-0,530661584	0,1454636	-3,648071	0,0002642	0,0017378	KLHL25
ENSGALG00000002253	1425,4871	-0,530784268	0,1456031	-3,64542	0,000267	0,0017508	HEMK1
ENSGALG00000011569	5815,2909	-0,530885029	0,12043	-4,408246	1,04E-05	0,000107	SCYL2
ENSGALG00000003944	1246,484	-0,531232705	0,1600895	-3,318348	0,0009055	0,0049275	FAM189A1
ENSGALG00000004347	6495,1605	-0,533321296	0,1103628	-4,832435	1,35E-06	1,70E-05	NEDD4
ENSGALG00000006710	714,0564	-0,533378082	0,1783936	-2,989895	0,0027907	0,0124332	RAB36
ENSGALG00000000891	3716,0687	-0,533422492	0,1230689	-4,33434	1,46E-05	0,000144	NSUN5
ENSGALG00000015818	5198,5394	-0,53353462	0,1266077	-4,214076	2,51E-05	0,0002282	E2F5
ENSGALG00000015448	2486,2853	-0,533672096	0,1639578	-3,254936	0,0011342	0,0059369	TOX
ENSGALG00000008312	1844,0991	-0,534378374	0,1384766	-3,858978	0,0001139	0,0008371	GSAP
ENSGALG00000004506	11897,642	-0,534405871	0,1101239	-4,852769	1,22E-06	1,55E-05	URI1
ENSGALG00000013085	2436,8268	-0,535478159	0,1366821	-3,917689	8,94E-05	0,0006824	PTPRO
ENSGALG00000008098	5171,8512	-0,536763264	0,1152605	-4,656959	3,21E-06	3,70E-05	NAMPT
ENSGALG00000016024	730,05733	-0,537143611	0,1775788	-3,024818	0,0024878	0,0112763	SETD4
ENSGALG00000006856	8733,4208	-0,537151634	0,1162633	-4,62013	3,83E-06	4,35E-05	CD151
ENSGALG00000011024	13428,049	-0,537183599	0,1048649	-5,122625	3,01E-07	4,37E-06	CACHD1
ENSGALG00000010564	3014,7883	-0,537373895	0,1233997	-4,354741	1,33E-05	0,0001323	TBCK
ENSGALG00000015360	9022,7064	-0,537653684	0,1055635	-5,093178	3,52E-07	5,05E-06	KIAA1524
ENSGALG00000026253	1652,9426	-0,5379573	0,1506081	-3,571902	0,0003544	0,0022422	TMEM144
ENSGALG00000003705	3639,3367	-0,538250704	0,1231851	-4,369445	1,25E-05	0,0001252	VPS13C
ENSGALG00000012468	13779,95	-0,538390713	0,1366618	-3,939585	8,16E-05	0,0006293	PRPF39
ENSGALG00000016440	2000,29	-0,538612864	0,1606177	-3,353384	0,0007983	0,0044417	KLF11
ENSGALG00000016835	6008,9103	-0,539714594	0,1468334	-3,675694	0,0002372	0,0015823	ATP11A
ENSGALG00000009476	2384,795	-0,539812489	0,1852255	-2,914353	0,0035643	0,0152027	CDK6
ENSGALG00000002873	15190,766	-0,539979366	0,10839	-4,981821	6,30E-07	8,52E-06	RSL1D1
ENSGALG00000015701	9337,5788	-0,540112853	0,1299709	-4,155644	3,24E-05	0,0002838	CXADR
ENSGALG00000015180	15097,101	-0,540189844	0,1129211	-4,78378	1,72E-06	2,11E-05	PSAT1
ENSGALG00000001389	3052,2811	-0,540945617	0,1260291	-4,292227	1,77E-05	0,0001682	TTLL11
ENSGALG00000029022	1420,5998	-0,540984923	0,2146447	-2,520374	0,011723	0,0398623	NPAS3
ENSGALG00000015491	4074,1339	-0,541085652	0,1219675	-4,436312	9,15E-06	9,53E-05	GPR89B
ENSGALG00000012121	12053,922	-0,541445831	0,1045431	-5,179165	2,23E-07	3,34E-06	RCN1
ENSGALG00000023089	788,71726	-0,541614618	0,1982755	-2,731627	0,0063022	0,0242593	SSBP2
ENSGALG00000013438	21891,879	-0,541667727	0,1120475	-4,834271	1,34E-06	1,69E-05	TEX10
ENSGALG00000001957	8870,632	-0,541703645	0,1341035	-4,039444	5,36E-05	0,0004373	ITCH
ENSGALG00000016460	6190,6626	-0,541890883	0,14842	-3,651065	0,0002612	0,0017202	NBAS
ENSGALG00000014573	17671,905	-0,541989088	0,1103445	-4,911792	9,02E-07	1,18E-05	NAT10
ENSGALG00000025884	6669,8373	-0,543082298	0,1134242	-4,788063	1,68E-06	2,07E-05	NABP1
ENSGALG00000007835	1738,059	-0,543517994	0,1483373	-3,664068	0,0002482	0,0016487	PUS10

ENSGALG00000006895	13372,527	-0,543915173	0,1058888	-5,136663	2,80E-07	4,09E-06	CEP131
ENSGALG00000004364	7914,9764	-0,544307466	0,1156323	-4,707225	2,51E-06	2,97E-05	ABR
ENSGALG00000014769	613,98427	-0,544618003	0,20267	-2,687216	0,007205	0,0270761	NLN
ENSGALG00000026452	7763,7201	-0,545277299	0,1319298	-4,133086	3,58E-05	0,0003098	MURC
ENSGALG00000008623	615,02509	-0,545289509	0,1967721	-2,771173	0,0055855	0,021976	TOR4A
ENSGALG00000012223	1554,6199	-0,545359079	0,1399232	-3,89756	9,72E-05	0,0007325	ZRANB3
ENSGALG00000004879	617,17052	-0,545838008	0,1927673	-2,831591	0,0046317	0,0187848	SLC6A11
ENSGALG00000004744	525,6252	-0,546332008	0,1957015	-2,791659	0,0052439	0,0208575	DISP2
ENSGALG00000003440	1101,0238	-0,546469024	0,1603017	-3,409004	0,000652	0,0037377	
ENSGALG00000011351	157820,47	-0,547374715	0,1580098	-3,464182	0,0005318	0,0031579	HSP90AA1
ENSGALG00000016768	7527,2287	-0,54750013	0,11987	-4,56745	4,94E-06	5,48E-05	LONRF2
ENSGALG00000012894	568,24499	-0,54779071	0,2155009	-2,541941	0,0110239	0,0380183	CHRM2
ENSGALG00000009503	5606,8039	-0,547842813	0,1186519	-4,617227	3,89E-06	4,41E-05	RAB3GAP2
ENSGALG00000015746	3303,5119	-0,547863301	0,1403611	-3,903241	9,49E-05	0,0007163	JAM2
ENSGALG00000003937	13547,502	-0,548529686	0,130015	-4,218972	2,45E-05	0,0002241	GEMIN5
ENSGALG00000005727	12274,551	-0,548590838	0,1154072	-4,753525	2,00E-06	2,41E-05	SLC7A3
ENSGALG00000017083	1252,4876	-0,54907192	0,1596795	-3,438588	0,0005848	0,003414	KATNAL1
ENSGALG00000006971	10409,679	-0,549657911	0,1186418	-4,63292	3,61E-06	4,12E-05	CDK5RAP2
ENSGALG00000003026	1987,5595	-0,550107166	0,1687281	-3,260317	0,0011129	0,0058389	MKL2
ENSGALG00000029127	622,20536	-0,550279038	0,2169523	-2,536406	0,0111997	0,0384768	
ENSGALG00000011400	12123,593	-0,550294288	0,103122	-5,336343	9,48E-08	1,53E-06	USP44
ENSGALG00000028501	7019,464	-0,550742687	0,1179763	-4,668248	3,04E-06	3,52E-05	DCK
ENSGALG00000013503	838,79916	-0,551514976	0,1874586	-2,942063	0,0032603	0,0140874	FAM149A
ENSGALG00000007097	2035,7636	-0,551520351	0,1696313	-3,25129	0,0011488	0,0059997	RPS6KA6
ENSGALG00000017006	4626,7368	-0,551986159	0,1179626	-4,679334	2,88E-06	3,35E-05	SETDB2
ENSGALG00000004071	3249,4429	-0,552437495	0,123172	-4,485092	7,29E-06	7,73E-05	NETO2
ENSGALG00000000684	3181,0431	-0,552570682	0,1435454	-3,849448	0,0001184	0,0008642	KLHDC8A
ENSGALG00000003835	27823,208	-0,552761335	0,1061578	-5,20698	1,92E-07	2,92E-06	ATP2A2
ENSGALG00000011052	797,4232	-0,553217179	0,2034892	-2,718657	0,0065548	0,0250612	SLC38A11
ENSGALG00000010309	346,20571	-0,554430553	0,2211705	-2,506801	0,0121829	0,0410672	ZC2HC1C
ENSGALG00000016883	3231,0954	-0,555002491	0,1279098	-4,339014	1,43E-05	0,0001413	DOCK9
ENSGALG00000004813	26912,189	-0,555221474	0,117319	-4,732578	2,22E-06	2,65E-05	IARS
ENSGALG00000005774	2418,9054	-0,555555373	0,133361	-4,165801	3,10E-05	0,0002736	NKAIN4
ENSGALG00000013635	10619,161	-0,555662229	0,1221599	-4,548648	5,40E-06	5,91E-05	SCAF8
ENSGALG00000016468	6793,0911	-0,555855719	0,108947	-5,102077	3,36E-07	4,84E-06	GEN1
ENSGALG00000026379	480,26073	-0,557143449	0,1981326	-2,811973	0,0049239	0,0198356	OPRD1
ENSGALG00000013430	2775,6809	-0,557212283	0,1237629	-4,502256	6,72E-06	7,16E-05	
ENSGALG00000021487	6790,2413	-0,55890103	0,1279072	-4,369583	1,24E-05	0,0001252	
ENSGALG00000016231	123947,5	-0,55907395	0,1098338	-5,09018	3,58E-07	5,11E-06	DDX3X
ENSGALG00000019178	3990,1334	-0,559313492	0,1249859	-4,475011	7,64E-06	8,08E-05	PRDM15
ENSGALG00000010022	5530,328	-0,559381952	0,1189802	-4,701473	2,58E-06	3,04E-05	PRMT9
ENSGALG00000011692	9047,4774	-0,559417827	0,1160828	-4,819127	1,44E-06	1,81E-05	CDCA4
ENSGALG00000017021	332,60369	-0,55959631	0,2270786	-2,464329	0,013727	0,045078	ATP7B
ENSGALG00000000447	760,9204	-0,559669779	0,171256	-3,268029	0,001083	0,0057107	KCNA3
ENSGALG00000011541	6975,1988	-0,559795067	0,1404905	-3,984576	6,76E-05	0,0005331	BIN1
ENSGALG00000003510	5135,2375	-0,562160497	0,117815	-4,771553	1,83E-06	2,23E-05	UCK2
ENSGALG00000012508	4886,2726	-0,562203872	0,114843	-4,895414	9,81E-07	1,28E-05	TRIP13
ENSGALG00000027231	9354,1487	-0,562988244	0,1537161	-3,66252	0,0002497	0,0016558	NRARP
ENSGALG00000016897	2920,7661	-0,563110321	0,1234746	-4,560535	5,10E-06	5,64E-05	GPR180
ENSGALG00000010081	2535,9184	-0,563235953	0,1656002	-3,401179	0,000671	0,0038367	SH3D19
ENSGALG00000003074	3831,5986	-0,563796907	0,1286586	-4,382116	1,18E-05	0,0001188	HOMER3
ENSGALG00000012256	15406,505	-0,563809375	0,163288	-3,452852	0,0005547	0,0032709	UTRN
ENSGALG00000026438	2075,6069	-0,563826313	0,1305634	-4,318409	1,57E-05	0,0001522	
ENSGALG00000008132	7657,4934	-0,564518166	0,1163041	-4,853812	1,21E-06	1,55E-05	PUS7
ENSGALG00000013641	530,0064	-0,565215365	0,2025498	-2,790501	0,0052627	0,0209261	MTMR7
ENSGALG00000026583	481,9172	-0,565402739	0,219019	-2,581523	0,0098365	0,0347405	
ENSGALG00000022808	24156,793	-0,565497585	0,1052406	-5,373379	7,73E-08	1,27E-06	RRP1B
ENSGALG00000011228	7018,8875	-0,565699287	0,1248478	-4,53111	5,87E-06	6,35E-05	WARS
ENSGALG00000002623	550,49256	-0,566538314	0,1922408	-2,947024	0,0032085	0,0139164	PCOLCE2
ENSGALG00000014940	2607,3053	-0,566659105	0,1584762	-3,575674	0,0003493	0,0022157	ZUFSP
ENSGALG00000016917	1018,5023	-0,567495374	0,1648915	-3,441629	0,0005782	0,0033831	CLN5
ENSGALG00000015431	2610,4581	-0,567537408	0,1279963	-4,434014	9,25E-06	9,61E-05	UBXN2B

ENSGALG00000009855	7273,6535	-0,567662024	0,1223057	-4,641336	3,46E-06	3,97E-05	TBC1D9
ENSGALG00000017079	12990,655	-0,56791639	0,124822	-4,549812	5,37E-06	5,89E-05	USPL1
ENSGALG00000000195	1824,66	-0,568283448	0,1600789	-3,55002	0,0003852	0,0024013	SLC23A2
ENSGALG00000003906	12475,038	-0,568387401	0,109475	-5,191937	2,08E-07	3,14E-06	MPHOSPH10
ENSGALG00000016281	11421,229	-0,569360911	0,1081146	-5,266272	1,39E-07	2,17E-06	DMD
ENSGALG00000016391	2197,6068	-0,569812042	0,1354173	-4,207823	2,58E-05	0,0002336	CNKSR2
ENSGALG00000015142	3202,1058	-0,570285644	0,1334226	-4,27428	1,92E-05	0,0001807	DSG2
ENSGALG00000009714	1150,5594	-0,570305895	0,1555825	-3,665616	0,0002467	0,0016411	CBR4
ENSGALG00000014783	10636,1	-0,572122303	0,1261051	-4,53687	5,71E-06	6,20E-05	PTPRK
ENSGALG00000016910	23108,199	-0,572230903	0,1116391	-5,125723	2,96E-07	4,30E-06	RBM26
ENSGALG00000012849	5113,4695	-0,57279907	0,1488538	-3,848064	0,0001191	0,0008677	SLC37A3
ENSGALG00000001363	21664,352	-0,573517858	0,1098878	-5,219123	1,80E-07	2,75E-06	MYBBP1A
ENSGALG00000003180	15412,658	-0,573999724	0,1081856	-5,305695	1,12E-07	1,78E-06	TSR1
ENSGALG00000013031	1094,9404	-0,574014118	0,173605	-3,306438	0,0009449	0,0051051	CECR1
ENSGALG00000010035	2145,4649	-0,574430705	0,1965144	-2,923098	0,0034657	0,0148241	NR3C2
ENSGALG00000016807	10081,673	-0,574724846	0,1138634	-5,047493	4,48E-07	6,28E-06	LIMS1
ENSGALG00000016841	10674,737	-0,576008454	0,1276781	-4,511411	6,44E-06	6,90E-05	COL4A1
ENSGALG00000020057	1737,427	-0,576621955	0,1365122	-4,223958	2,40E-05	0,0002196	PIGF
ENSGALG00000007992	6680,6643	-0,5769061	0,1093351	-5,276494	1,32E-07	2,06E-06	GGA3
ENSGALG00000003591	1041,8529	-0,577610116	0,1793022	-3,221433	0,0012755	0,006536	FTO
ENSGALG00000014939	2493,3187	-0,579384653	0,1743511	-3,323091	0,0008903	0,004862	GFM2
ENSGALG00000004612	6633,1212	-0,579433401	0,1094771	-5,292734	1,21E-07	1,90E-06	MTHFR
ENSGALG00000007008	8124,263	-0,579649516	0,117537	-4,931634	8,15E-07	1,08E-05	POLR3E
ENSGALG00000004140	1503,5608	-0,579940212	0,1679604	-3,452839	0,0005547	0,0032709	SH3BP4
ENSGALG00000015629	16787,85	-0,581629702	0,1013849	-5,736848	9,65E-09	1,91E-07	TRAM1
ENSGALG00000010202	1508,6027	-0,581722546	0,1688428	-3,445349	0,0005703	0,0033455	ENTPD5
ENSGALG00000006713	2292,8924	-0,58181715	0,1450076	-4,012322	6,01E-05	0,0004821	CELF2
ENSGALG00000011464	79055,793	-0,582311331	0,1058239	-5,502646	3,74E-08	6,56E-07	EIF5
ENSGALG00000017130	3473,8029	-0,582570563	0,1291633	-4,510341	6,47E-06	6,91E-05	LATS2
ENSGALG00000008288	656,98572	-0,582969807	0,1906368	-3,058013	0,0022281	0,0103145	ARMC10
ENSGALG00000015000	15510,253	-0,584898632	0,104418	-5,601509	2,12E-08	3,93E-07	LYAR
ENSGALG00000020899	644,11569	-0,585391733	0,1791404	-3,267782	0,0010839	0,0057134	
ENSGALG00000027517	979,67929	-0,585667855	0,1639516	-3,5722	0,000354	0,0022407	gga-mir-3064
ENSGALG00000007677	1856,8714	-0,585818002	0,1452003	-4,03455	5,47E-05	0,0004451	THNSL1
ENSGALG00000003426	1636,1649	-0,587203746	0,1565835	-3,750099	0,0001768	0,0012329	RHBDL3
ENSGALG00000015765	12998,254	-0,587270267	0,1455632	-4,034469	5,47E-05	0,0004451	POLR1A
ENSGALG00000015310	4463,7435	-0,587354563	0,1636253	-3,589632	0,0003311	0,0021177	SOBP
ENSGALG00000008263	616,84886	-0,587476931	0,2027246	-2,897907	0,0037566	0,0158984	CNTN4
ENSGALG00000004144	27007,859	-0,588131949	0,109754	-5,358638	8,39E-08	1,37E-06	DDX50
ENSGALG00000012946	11580,052	-0,588849778	0,1146976	-5,133933	2,84E-07	4,13E-06	FAM60A
ENSGALG00000016187	8513,8325	-0,588903063	0,112954	-5,213653	1,85E-07	2,82E-06	WDR4
ENSGALG00000000284	7651,6591	-0,589684267	0,1227001	-4,8059	1,54E-06	1,92E-05	HOXB4
ENSGALG00000025668	302,68906	-0,590006716	0,2416858	-2,441214	0,014638	0,0474627	
ENSGALG00000015556	4802,1991	-0,591646833	0,1279382	-4,624475	3,76E-06	4,27E-05	AFAP1
ENSGALG00000012147	18627,042	-0,591734529	0,112588	-5,255749	1,47E-07	2,30E-06	DDX18
ENSGALG00000008376	8424,8255	-0,592197559	0,1214407	-4,876433	1,08E-06	1,40E-05	EIF2B5
ENSGALG00000005803	3271,7652	-0,592808251	0,1582098	-3,746976	0,000179	0,0012457	ZNRF3
ENSGALG00000002326	890,06973	-0,593338649	0,1837643	-3,228802	0,0012431	0,0064011	
ENSGALG00000028863	286,98407	-0,594135525	0,2453357	-2,421725	0,015447	0,049555	
ENSGALG00000011449	1934,5212	-0,594721435	0,1428135	-4,164321	3,12E-05	0,000275	
ENSGALG00000007536	1357,1535	-0,59551277	0,156731	-3,799585	0,0001449	0,0010337	
ENSGALG00000007048	584,57505	-0,595618933	0,2088367	-2,85208	0,0043434	0,0178334	SYNM
ENSGALG00000005692	514,92687	-0,595884023	0,2256362	-2,640906	0,0082685	0,0302134	CRISPLD2
ENSGALG00000014213	4954,5042	-0,596053951	0,129599	-4,599216	4,24E-06	4,76E-05	PRR5
ENSGALG00000009458	5058,5683	-0,596085417	0,1247109	-4,779738	1,76E-06	2,15E-05	
ENSGALG00000005171	1357,2271	-0,597406416	0,14458	-4,132013	3,60E-05	0,0003107	AP1S3
ENSGALG00000005243	13547,504	-0,597738338	0,1028436	-5,812113	6,17E-09	1,27E-07	PPP3CB
ENSGALG00000020438	1854,2018	-0,597817303	0,1402495	-4,262527	2,02E-05	0,0001893	
ENSGALG00000027159	3433,7581	-0,598718251	0,1676492	-3,571256	0,0003553	0,0022435	CNKSR3
ENSGALG00000014615	13865,336	-0,598774766	0,1450012	-4,129446	3,64E-05	0,0003134	LAMA1
ENSGALG00000011210	4763,2532	-0,598839459	0,1129355	-5,302492	1,14E-07	1,81E-06	FAM175A
ENSGALG00000020003	15165,061	-0,59891041	0,1111234	-5,389599	7,06E-08	1,17E-06	MAP3K4

ENSGALG00000015409	17031,867	-0,599227418	0,1473812	-4,065834	4,79E-05	0,0003956	PROS1
ENSGALG00000011710	2961,6307	-0,599591029	0,1881948	-3,186013	0,0014425	0,007223	BTBD6
ENSGALG00000009437	2415,945	-0,59959234	0,2125339	-2,821161	0,004785	0,0193375	MECOM
ENSGALG00000001003	4385,7574	-0,600081977	0,1214895	-4,939372	7,84E-07	1,04E-05	SEPN1
ENSGALG00000006256	7733,3335	-0,600800071	0,1126462	-5,333512	9,63E-08	1,55E-06	MAP7D3
ENSGALG00000003110	375,89024	-0,601903021	0,2441478	-2,465322	0,013689	0,044964	
ENSGALG00000011280	7822,1495	-0,603497491	0,1227685	-4,915737	8,84E-07	1,16E-05	EEA1
ENSGALG00000010603	12309,752	-0,604746743	0,1082631	-5,585897	2,32E-08	4,26E-07	CEBPZ
ENSGALG00000005807	96623,567	-0,604834873	0,1046771	-5,778103	7,55E-09	1,52E-07	IPO7
ENSGALG00000007030	1042,0347	-0,605544059	0,1602843	-3,777937	0,0001581	0,0011161	
ENSGALG00000010623	6764,8612	-0,606701235	0,111882	-5,422691	5,87E-08	9,92E-07	ORC1
ENSGALG00000006147	7732,277	-0,607224855	0,1099367	-5,523405	3,32E-08	5,88E-07	NUCB2
ENSGALG00000009348	14314,788	-0,607303929	0,150627	-4,031839	5,53E-05	0,0004496	AKAP9
ENSGALG00000011522	2524,7796	-0,607850232	0,1342636	-4,527291	5,97E-06	6,44E-05	TRMT61A
ENSGALG00000012407	335,57386	-0,607964974	0,2234167	-2,721215	0,0065042	0,02491	LRP1B
ENSGALG00000010538	11960,175	-0,60879058	0,1100325	-5,532827	3,15E-08	5,61E-07	TTC27
ENSGALG00000011618	1230,2077	-0,609057453	0,1895822	-3,21263	0,0013153	0,0067043	ADSSL1
ENSGALG00000015689	13523,347	-0,609077567	0,14383	-4,234705	2,29E-05	0,0002105	KIAA0368
ENSGALG00000005492	6703,9725	-0,609713298	0,1420841	-4,291213	1,78E-05	0,0001688	NBEAL2
ENSGALG00000009698	6354,4127	-0,610790424	0,1589015	-3,843831	0,0001211	0,0008818	SLC38A1
ENSGALG00000016302	7538,4329	-0,61104581	0,1423529	-4,292472	1,77E-05	0,0001681	ASAP1
ENSGALG00000000769	395,02523	-0,611277614	0,2259769	-2,705045	0,0068295	0,0258936	RAB7B
ENSGALG00000000438	3178,9721	-0,61235395	0,1445774	-4,235475	2,28E-05	0,0002099	ERMP1
ENSGALG00000010607	463,53756	-0,613528032	0,2050464	-2,992142	0,0027703	0,0123558	SPATA7
ENSGALG00000004169	13748,93	-0,613558741	0,1070672	-5,730594	1,00E-08	1,97E-07	
ENSGALG00000013760	3088,0215	-0,614432788	0,1210707	-5,07499	3,88E-07	5,50E-06	GFPT2
ENSGALG00000016574	1715,0637	-0,614768808	0,142039	-4,328168	1,50E-05	0,0001471	GEMIN8
ENSGALG00000007703	1248,9929	-0,614958252	0,1535298	-4,005464	6,19E-05	0,0004943	
ENSGALG00000013480	4896,2242	-0,615680556	0,1242349	-4,955777	7,20E-07	9,61E-06	MTHFD2
ENSGALG00000016808	40422,889	-0,615970468	0,1230783	-5,004705	5,59E-07	7,68E-06	RANBP2
ENSGALG00000006237	12767,815	-0,616678741	0,1272849	-4,844871	1,27E-06	1,61E-05	PKN2
ENSGALG00000016951	1808,556	-0,617422601	0,1357822	-4,547156	5,44E-06	5,94E-05	MTRF1
ENSGALG00000007631	328,24576	-0,617881783	0,2272908	-2,718464	0,0065586	0,0250618	MEGF11
ENSGALG00000028279	367,51739	-0,618647245	0,2374707	-2,605152	0,0091834	0,0327994	
ENSGALG00000008265	23793,57	-0,619236444	0,1097973	-5,639813	1,70E-08	3,22E-07	PDCD11
ENSGALG00000017388	6991,3984	-0,619582917	0,1159517	-5,343459	9,12E-08	1,48E-06	YY1
ENSGALG00000005815	11029,748	-0,619938317	0,1099084	-5,640501	1,70E-08	3,22E-07	TMEM41B
ENSGALG00000012533	37098,602	-0,619977689	0,143535	-4,31935	1,56E-05	0,0001518	MYH9
ENSGALG00000001192	22737,825	-0,620466491	0,1546499	-4,012072	6,02E-05	0,0004822	ARHGAP32
ENSGALG00000011058	317,84832	-0,620682204	0,234088	-2,651491	0,0080137	0,0294645	LEPR
ENSGALG00000016653	1523,2789	-0,621295808	0,1474254	-4,214308	2,51E-05	0,0002282	FAM167A
ENSGALG00000012447	5839,1863	-0,62156312	0,1278297	-4,862432	1,16E-06	1,49E-05	TNS3
ENSGALG00000005319	350,2389	-0,621682491	0,2536246	-2,451192	0,0142384	0,0464103	CDH8
ENSGALG00000008539	7630,1458	-0,622137491	0,1211519	-5,135185	2,82E-07	4,11E-06	ALG12
ENSGALG00000023419	9218,5287	-0,622730836	0,1310534	-4,751733	2,02E-06	2,42E-05	HOXD4
ENSGALG00000015271	532,65573	-0,623845538	0,2308973	-2,701831	0,0068959	0,0260871	FILIP1L
ENSGALG00000000234	2924,3601	-0,623968223	0,1484686	-4,202696	2,64E-05	0,0002381	EPB41L4A
ENSGALG00000005438	938,76871	-0,624083264	0,1616022	-3,861849	0,0001125	0,0008286	PARVA
ENSGALG00000010975	6618,9998	-0,624280901	0,1157101	-5,395213	6,84E-08	1,14E-06	TMTC2
ENSGALG00000005504	1081,6574	-0,624552568	0,1731409	-3,607192	0,0003095	0,0019954	HNF1B
ENSGALG00000006609	9375,2554	-0,624834648	0,1373671	-4,548649	5,40E-06	5,91E-05	EPHB1
ENSGALG00000014860	10686,581	-0,625239897	0,107074	-5,839324	5,24E-09	1,09E-07	YES1
ENSGALG00000017159	5806,6713	-0,626252438	0,142901	-4,382421	1,17E-05	0,0001187	ATM
ENSGALG00000004046	1117,4696	-0,627070394	0,169937	-3,690016	0,0002242	0,0015092	LARP6
ENSGALG00000014855	4504,1501	-0,627700184	0,1611556	-3,894995	9,82E-05	0,0007383	GHR
ENSGALG00000010702	11951,045	-0,628390454	0,1083577	-5,799225	6,66E-09	1,36E-07	ADSS
ENSGALG00000015472	37144,636	-0,629536006	0,1475373	-4,266963	1,98E-05	0,000186	CHD7
ENSGALG00000012966	5358,7576	-0,62976328	0,1160975	-5,424435	5,81E-08	9,83E-07	ZBTB2
ENSGALG00000005450	5500,7304	-0,632586778	0,1208381	-5,234993	1,65E-07	2,54E-06	CCDC66
ENSGALG00000005256	11273,337	-0,63393871	0,1641492	-3,861966	0,0001125	0,0008286	EPHA4
ENSGALG00000014337	1369,7017	-0,634337238	0,1462286	-4,337984	1,44E-05	0,0001418	NWD2
ENSGALG00000009850	11171,857	-0,63459607	0,108041	-5,87366	4,26E-09	9,11E-08	NOVA1

ENSGALG00000004937	10776,497	-0,634904509	0,1095156	-5,797386	6,74E-09	1,37E-07	VGLL4
ENSGALG00000017368	7609,7868	-0,635358308	0,1095099	-5,801832	6,56E-09	1,34E-07	TRNT1
ENSGALG00000007392	3781,9294	-0,635704603	0,1408897	-4,512074	6,42E-06	6,88E-05	ADAMTS9
ENSGALG00000003767	4407,983	-0,636798593	0,1177151	-5,409657	6,31E-08	1,06E-06	NKD1
ENSGALG00000007710	1737,5833	-0,637223247	0,1525396	-4,177427	2,95E-05	0,0002612	
ENSGALG00000012813	15846,526	-0,637913408	0,1220106	-5,228345	1,71E-07	2,63E-06	PRPF4B
ENSGALG00000005521	7067,2749	-0,637995859	0,1317048	-4,844135	1,27E-06	1,62E-05	PER2
ENSGALG00000022490	838,24845	-0,638026939	0,2048258	-3,114973	0,0018396	0,008782	ARPC1B
ENSGALG00000023451	10451,614	-0,638494594	0,1052822	-6,064604	1,32E-09	3,09E-08	TRPM7
ENSGALG00000007056	3476,5095	-0,638790551	0,1810296	-3,528652	0,0004177	0,0025816	KLF6
ENSGALG00000003891	23052,983	-0,638963678	0,1001377	-6,380853	1,76E-10	4,67E-09	ZNF423
ENSGALG00000008304	1332,9115	-0,639371492	0,2087659	-3,062623	0,0021941	0,0101883	OBFC1
ENSGALG00000012662	3593,3785	-0,639523486	0,1240921	-5,153618	2,56E-07	3,78E-06	NUAK1
ENSGALG00000014138	4048,403	-0,640267154	0,1406798	-4,551239	5,33E-06	5,86E-05	NFXL1
ENSGALG00000011162	1836,7364	-0,640538648	0,1515623	-4,226239	2,38E-05	0,0002177	CYP46A1
ENSGALG00000021238	337,09341	-0,640934901	0,2252878	-2,84496	0,0044417	0,0181603	CYP2W1
ENSGALG00000009240	3768,4908	-0,641416186	0,1167026	-5,49616	3,88E-08	6,79E-07	NFE2L2
ENSGALG00000007352	21915,639	-0,641667347	0,1092352	-5,874181	4,25E-09	9,10E-08	SPAG9
ENSGALG00000009445	3221,2949	-0,642383978	0,1226543	-5,237353	1,63E-07	2,51E-06	INPP5F
ENSGALG00000010642	1388,2464	-0,643267076	0,1679801	-3,829424	0,0001284	0,0009312	IRF2
ENSGALG00000027704	758,7446	-0,643979597	0,2120706	-3,036628	0,0023924	0,0109231	
ENSGALG00000005321	14326,683	-0,645095033	0,1462871	-4,409787	1,03E-05	0,0001064	LAMA5
ENSGALG00000005024	4274,063	-0,645541534	0,1373988	-4,698306	2,62E-06	3,09E-05	EFCC1
ENSGALG00000026181	1587,8742	-0,645581251	0,1576569	-4,094848	4,22E-05	0,0003559	STRIP2
ENSGALG00000010536	11449,034	-0,646034394	0,1106982	-5,835997	5,35E-09	1,11E-07	FAF1
ENSGALG00000016480	10063,583	-0,647091328	0,1222876	-5,291552	1,21E-07	1,91E-06	SDC1
ENSGALG00000000258	23273,697	-0,649481888	0,1229147	-5,284004	1,26E-07	1,98E-06	HEATR1
ENSGALG00000000761	2678,9011	-0,649687901	0,1320076	-4,921596	8,58E-07	1,13E-05	TSKU
ENSGALG00000014902	5870,3239	-0,650325285	0,1293417	-5,027965	4,96E-07	6,90E-06	RP1-179P9.3
ENSGALG00000012847	300,13784	-0,651077536	0,259355	-2,510372	0,0120604	0,0407551	
ENSGALG00000006864	655,13638	-0,651213169	0,1997356	-3,260377	0,0011126	0,0058389	COL24A1
ENSGALG00000014873	10860,594	-0,6514166	0,1060541	-6,142302	8,13E-10	1,95E-08	GJA1
ENSGALG00000012232	769,02109	-0,652989979	0,197777	-3,301647	0,0009612	0,0051726	DNAAF2
ENSGALG00000003154	3509,1767	-0,653496705	0,122188	-5,348291	8,88E-08	1,45E-06	TXNDC11
ENSGALG00000005845	8323,5942	-0,655210424	0,124744	-5,252442	1,50E-07	2,33E-06	SLC7A5
ENSGALG00000015241	1466,0595	-0,655397927	0,141762	-4,623228	3,78E-06	4,29E-05	SNAI2
ENSGALG00000015653	733,01948	-0,655959247	0,2027357	-3,235538	0,0012141	0,0062782	JPH1
ENSGALG00000016681	817,92363	-0,656368052	0,1696389	-3,869207	0,0001092	0,0008088	DHRSX
ENSGALG00000000690	5466,0012	-0,657633862	0,1415168	-4,647039	3,37E-06	3,87E-05	GDPD4
ENSGALG00000016817	6729,2541	-0,658902643	0,1108429	-5,944473	2,77E-09	6,16E-08	RASA3
ENSGALG00000006975	7158,0796	-0,659116429	0,117355	-5,616432	1,95E-08	3,65E-07	TBC1D22A
ENSGALG00000006840	2751,6848	-0,659559733	0,1711898	-3,852798	0,0001168	0,0008552	AKAP13
ENSGALG00000006681	619,27869	-0,660452265	0,2149441	-3,07267	0,0021215	0,009926	BRSK2
ENSGALG00000010204	972,3601	-0,660708753	0,1680654	-3,93126	8,45E-05	0,0006489	KCNC2
ENSGALG00000008551	7057,1759	-0,661341847	0,1249566	-5,292574	1,21E-07	1,90E-06	CRELD2
ENSGALG00000007870	12818,558	-0,66197661	0,1166502	-5,674884	1,39E-08	2,68E-07	SMAD3
ENSGALG00000001405	334,6643	-0,663151012	0,2350856	-2,820891	0,004789	0,019348	IRF6
ENSGALG00000005796	13227,627	-0,664735985	0,1063503	-6,25044	4,09E-10	1,01E-08	XBP1
ENSGALG00000008293	829,33594	-0,66560113	0,2005473	-3,318924	0,0009036	0,0049193	SH3PXD2A
ENSGALG00000016246	560,38544	-0,667393695	0,2438472	-2,736934	0,0062015	0,0239052	KCNQ3
ENSGALG00000009915	13723,099	-0,66741216	0,1151588	-5,79558	6,81E-09	1,39E-07	EML4
ENSGALG00000005512	8645,0111	-0,667653797	0,1184896	-5,634703	1,75E-08	3,31E-07	KIAA1671
ENSGALG00000008418	2809,2517	-0,668553187	0,1227062	-5,448405	5,08E-08	8,65E-07	GYLTL1B
ENSGALG00000011139	4725,8286	-0,66929694	0,1340022	-4,99467	5,89E-07	8,03E-06	CCDC85C
ENSGALG00000011686	12938,733	-0,670528172	0,1470322	-4,560417	5,11E-06	5,64E-05	SEMA5B
ENSGALG00000007278	350,13209	-0,670809216	0,2308902	-2,905317	0,0036688	0,0155756	GRIN2A
ENSGALG00000008240	2340,2977	-0,670953542	0,1295685	-5,178368	2,24E-07	3,35E-06	CFLAR
ENSGALG00000006080	30589,303	-0,672288685	0,1018389	-6,601491	4,07E-11	1,17E-09	GPC4
ENSGALG00000028291	15611,072	-0,672824764	0,1341958	-5,013756	5,34E-07	7,39E-06	BCAR1
ENSGALG00000004715	1867,9414	-0,672888292	0,1525035	-4,41228	1,02E-05	0,0001053	ZC3H12B
ENSGALG00000015912	3388,0589	-0,674238434	0,1460392	-4,616833	3,90E-06	4,41E-05	NBN
ENSGALG00000015708	22628,562	-0,674375402	0,1784895	-3,778235	0,0001579	0,0011154	FGFR3

ENSGALG00000008684	70609,009	-0,674536207	0,1288068	-5,236804	1,63E-07	2,52E-06	EIF4A2
ENSGALG00000010049	749,198	-0,6748978	0,2082509	-3,240792	0,001192	0,0061824	PTPRB
ENSGALG00000003249	7468,8212	-0,675105103	0,1116673	-6,045684	1,49E-09	3,45E-08	CWF19L1
ENSGALG00000006035	6729,7716	-0,676290578	0,1083357	-6,242548	4,30E-10	1,06E-08	PDE3B
ENSGALG00000004494	4756,9197	-0,676838081	0,11958	-5,66013	1,51E-08	2,89E-07	CCNE1
ENSGALG00000016451	7945,9007	-0,679900325	0,139439	-4,87597	1,08E-06	1,40E-05	ROCK2
ENSGALG00000025674	227,33639	-0,681326883	0,2807179	-2,427088	0,0152206	0,0489092	
ENSGALG00000006437	3771,9905	-0,682364759	0,1217534	-5,60448	2,09E-08	3,87E-07	NOM1
ENSGALG00000009156	5048,9849	-0,682556058	0,1676146	-4,072176	4,66E-05	0,0003862	CDAN1
ENSGALG00000014274	3304,6353	-0,683648491	0,1394936	-4,900932	9,54E-07	1,25E-05	RIMKLB
ENSGALG00000012390	2074,4615	-0,684899295	0,1473975	-4,646615	3,37E-06	3,87E-05	PPP1R14C
ENSGALG00000009152	8451,9252	-0,685418448	0,1346381	-5,09082	3,57E-07	5,10E-06	ATRNL1
ENSGALG00000004631	4926,2821	-0,686947368	0,1966631	-3,493016	0,0004776	0,0028862	DRAXIN
ENSGALG00000010836	1260,2339	-0,688393097	0,166876	-4,125178	3,70E-05	0,0003178	AHR
ENSGALG00000005990	327,32624	-0,688776212	0,2796142	-2,463309	0,0137661	0,0451737	MGLL
ENSGALG00000003726	1048,3673	-0,689550024	0,2280538	-3,023628	0,0024976	0,0113049	EGFLAM
ENSGALG00000015879	11968,416	-0,689623134	0,1480044	-4,659477	3,17E-06	3,66E-05	HUNK
ENSGALG00000008234	5250,562	-0,689849994	0,1273096	-5,41868	6,00E-08	1,01E-06	ORC2
ENSGALG00000016308	5133,4014	-0,690314419	0,1281438	-5,387031	7,16E-08	1,18E-06	MYC
ENSGALG00000014686	1912,6046	-0,691021625	0,1730464	-3,993274	6,52E-05	0,0005167	FBN2
ENSGALG00000025627	543,3063	-0,691903941	0,1957541	-3,534557	0,0004085	0,0025313	
ENSGALG00000017866	156,05125	-0,692156313	0,285038	-2,428295	0,01517	0,0487927	
ENSGALG00000008292	9417,4765	-0,69263042	0,1222675	-5,664879	1,47E-08	2,83E-07	LRP4
ENSGALG00000017857	160,75501	-0,693298969	0,2819397	-2,459032	0,0139312	0,0455946	
ENSGALG00000011679	1487,1899	-0,695574487	0,1740708	-3,995929	6,44E-05	0,0005116	DIRC2
ENSGALG00000007371	25835,451	-0,695805113	0,1165608	-5,969459	2,38E-09	5,36E-08	IGDCC3
ENSGALG00000017220	8610,4222	-0,695886193	0,1257779	-5,532661	3,15E-08	5,61E-07	
ENSGALG00000008758	3528,4952	-0,696392948	0,1183183	-5,88576	3,96E-09	8,56E-08	B3GNT5
ENSGALG00000028415	2970,1144	-0,698216592	0,2041115	-3,42076	0,0006245	0,0036072	
ENSGALG00000016834	1336,2251	-0,698351437	0,1627936	-4,289798	1,79E-05	0,0001697	MCF2L
ENSGALG00000017812	214,0597	-0,698433493	0,2584052	-2,702862	0,0068745	0,0260208	
ENSGALG00000002316	6016,7291	-0,700708149	0,1098757	-6,377279	1,80E-10	4,77E-09	NUBP2
ENSGALG00000002689	3193,9897	-0,701352786	0,134772	-5,203994	1,95E-07	2,96E-06	NUP35
ENSGALG00000012654	165,03411	-0,702415833	0,2836155	-2,476648	0,0132623	0,0439509	NFATC1
ENSGALG00000007501	495,82592	-0,702573548	0,2210424	-3,178456	0,0014806	0,0073545	P2RX3
ENSGALG00000006592	563,46793	-0,703616422	0,1908922	-3,685937	0,0002279	0,0015305	MEIKIN
ENSGALG00000021316	17378,378	-0,704586437	0,1023184	-6,886217	5,73E-12	1,88E-10	SLC12A4
ENSGALG00000012153	842,90994	-0,706186244	0,1736471	-4,066791	4,77E-05	0,0003943	METTL15
ENSGALG00000002762	6873,8438	-0,70662137	0,1320656	-5,350533	8,77E-08	1,43E-06	UHRF1BP1
ENSGALG00000002726	8639,3567	-0,708755608	0,1395335	-5,079466	3,78E-07	5,38E-06	KIF1B
ENSGALG00000007141	2245,3684	-0,70907976	0,1459375	-4,858791	1,18E-06	1,52E-05	LRRK1
ENSGALG00000011680	3554,5974	-0,709602993	0,1836532	-3,86382	0,0001116	0,0008235	EPHA5
ENSGALG00000012848	3745,194	-0,711139877	0,1738156	-4,091346	4,29E-05	0,0003598	KDM7A
ENSGALG00000022857	997,97159	-0,711204976	0,1675693	-4,244244	2,19E-05	0,000203	
ENSGALG00000015733	14565,427	-0,711632203	0,1740069	-4,089677	4,32E-05	0,0003619	MDN1
ENSGALG00000017365	2855,5664	-0,712049377	0,1517404	-4,692551	2,70E-06	3,17E-05	REEP2
ENSGALG00000009562	5126,9731	-0,714412903	0,1287612	-5,548356	2,88E-08	5,20E-07	PLEK2
ENSGALG00000003970	25720,818	-0,714513793	0,1240731	-5,758812	8,47E-09	1,70E-07	TJP1
ENSGALG00000017871	177,34037	-0,715510132	0,2848578	-2,511815	0,0120112	0,0406308	
ENSGALG00000003161	1569,9242	-0,715518291	0,1658137	-4,315193	1,59E-05	0,0001539	GDF3
ENSGALG00000016927	1190,189	-0,715628819	0,1764386	-4,055965	4,99E-05	0,0004112	KLF5
ENSGALG00000013615	780,84937	-0,715808204	0,1833387	-3,904293	9,45E-05	0,0007136	MTUS1
ENSGALG00000011076	244,64999	-0,715907242	0,2845675	-2,515773	0,0118772	0,0402859	SYNE3
ENSGALG00000010036	359,24032	-0,717787007	0,2219662	-3,233767	0,0012217	0,0063086	FOSL2
ENSGALG00000015122	2528,5766	-0,719672556	0,1390865	-5,174279	2,29E-07	3,42E-06	TAF4B
ENSGALG00000020805	5789,8017	-0,719835862	0,1139176	-6,318916	2,63E-10	6,72E-09	LRRC8D
ENSGALG00000005430	950,09062	-0,72235333	0,2022537	-3,571522	0,0003549	0,0022423	ALS2CL
ENSGALG00000002553	2782,5411	-0,723490416	0,1606217	-4,504313	6,66E-06	7,10E-05	PCDH1
ENSGALG00000007242	1612,8307	-0,724284111	0,1436296	-5,04272	4,59E-07	6,42E-06	SLITRK4
ENSGALG00000008078	7758,4981	-0,725207523	0,1252194	-5,791493	6,98E-09	1,42E-07	FUT8
ENSGALG00000006978	1693,5845	-0,725851222	0,16966	-4,27827	1,88E-05	0,0001778	APOOL
ENSGALG00000004004	8836,3289	-0,725989536	0,1219894	-5,951253	2,66E-09	5,95E-08	PHKB

ENSGALG00000005246	9658,48	-0,7272373	0,1233553	-5,895469	3,74E-09	8,11E-08	PAX3
ENSGALG00000028294	3609,4147	-0,727698928	0,1304345	-5,579039	2,42E-08	4,40E-07	VWA5B2
ENSGALG00000006939	26463,229	-0,728311968	0,1092521	-6,666343	2,62E-11	7,76E-10	PFKP
ENSGALG00000004519	237,39511	-0,728806195	0,2713012	-2,686336	0,007224	0,02714	TRPV2
ENSGALG00000006555	1381,6916	-0,729109536	0,1554307	-4,690898	2,72E-06	3,19E-05	IGF2
ENSGALG00000025743	3141,8277	-0,729896626	0,1215881	-6,003027	1,94E-09	4,42E-08	CDR2
ENSGALG00000016078	860,37098	-0,73004938	0,1938639	-3,765783	0,000166	0,0011664	ZFPM2
ENSGALG00000014419	9837,2387	-0,730417334	0,1131065	-6,457784	1,06E-10	2,87E-09	SLIT2
ENSGALG00000020479	3560,7787	-0,731996794	0,1299087	-5,634701	1,75E-08	3,31E-07	RP11-872D17.8
ENSGALG00000003378	7303,9139	-0,732735156	0,1242316	-5,89814	3,68E-09	8,01E-08	USP42
ENSGALG00000015627	1040,2204	-0,73285779	0,2129145	-3,442028	0,0005774	0,0033796	HAPLN1
ENSGALG00000026349	29196,624	-0,736634172	0,0999371	-7,370975	1,69E-13	6,80E-12	HSD17B12
ENSGALG00000007069	592,20837	-0,738101541	0,2325984	-3,173288	0,0015072	0,0074595	ADAMTS17
ENSGALG00000016537	11629,834	-0,739933749	0,1619397	-4,569193	4,90E-06	5,45E-05	SCML2
ENSGALG00000010448	40598,94	-0,742255902	0,1099244	-6,752421	1,45E-11	4,48E-10	LTBP1
ENSGALG00000002679	1869,603	-0,743662338	0,1515547	-4,90689	9,25E-07	1,21E-05	GLT1D1
ENSGALG00000007331	18072,434	-0,744880346	0,1084385	-6,869149	6,46E-12	2,09E-10	SVIL
ENSGALG00000027639	27019,601	-0,74689172	0,1081151	-6,908304	4,90E-12	1,61E-10	RGMA
ENSGALG00000015077	367,6495	-0,747092368	0,2656853	-2,811945	0,0049243	0,0198356	ARHGAP31
ENSGALG00000010522	11132,1	-0,747786716	0,1199212	-6,23565	4,50E-10	1,11E-08	ALDH18A1
ENSGALG00000008741	273,37173	-0,747824755	0,2458046	-3,042355	0,0023474	0,0107607	
ENSGALG00000003975	3354,1135	-0,748226558	0,1450875	-5,15707	2,51E-07	3,71E-06	CEP112
ENSGALG00000008910	360,62302	-0,749041615	0,2212545	-3,385431	0,0007107	0,0040265	CERKL
ENSGALG00000003149	1878,4222	-0,749443102	0,1529534	-4,899812	9,59E-07	1,25E-05	ITPR3
ENSGALG00000012932	193,43191	-0,749547645	0,2920342	-2,566643	0,0102688	0,0359195	FGD4
ENSGALG00000016144	227,03636	-0,75041933	0,282532	-2,656051	0,0079062	0,0291714	TMPRSS2
ENSGALG00000007011	11491,263	-0,750635431	0,1125238	-6,670901	2,54E-11	7,56E-10	ZBTB16
ENSGALG00000023464	2176,7949	-0,751478603	0,162842	-4,614771	3,94E-06	4,45E-05	
ENSGALG00000018479	498,05044	-0,753094112	0,2693867	-2,795587	0,0051806	0,0206479	
ENSGALG00000019961	8882,8923	-0,7532835	0,1292302	-5,829007	5,58E-09	1,16E-07	SLC18B1
ENSGALG00000003430	9094,4226	-0,754857044	0,1082845	-6,971053	3,15E-12	1,08E-10	PMS2
ENSGALG00000015862	12207,703	-0,756350316	0,1499013	-5,045657	4,52E-07	6,34E-06	CA2
ENSGALG00000008318	18620,526	-0,756789619	0,1179267	-6,41746	1,39E-10	3,71E-09	SLK
ENSGALG00000010412	643,75824	-0,756929566	0,1797201	-4,211714	2,53E-05	0,00023	WWTR1
ENSGALG00000004981	3521,9026	-0,757070103	0,1329306	-5,695226	1,23E-08	2,40E-07	TSEN2
ENSGALG00000006981	1290,8961	-0,757579596	0,1692071	-4,477234	7,56E-06	8,01E-05	MCTP2
ENSGALG00000007667	1024,279	-0,757882718	0,1665229	-4,551224	5,33E-06	5,86E-05	GPR158
ENSGALG00000000742	7852,9776	-0,757900463	0,1311808	-5,777528	7,58E-09	1,53E-07	PIM1
ENSGALG00000001419	2086,701	-0,758492411	0,1407402	-5,389307	7,07E-08	1,17E-06	DAB2IP
ENSGALG00000016294	651,26015	-0,761040192	0,2034016	-3,741564	0,0001829	0,0012678	HMGCLL1
ENSGALG00000009748	12417,603	-0,762363604	0,1219472	-6,251585	4,06E-10	1,01E-08	ASNS
ENSGALG00000006167	4665,1269	-0,763429455	0,1613684	-4,730971	2,23E-06	2,66E-05	LRRC8B
ENSGALG00000017462	3834,7093	-0,763892042	0,1177124	-6,489476	8,61E-11	2,38E-09	FNIP1
ENSGALG00000028254	428,45012	-0,764100303	0,2118876	-3,606159	0,0003108	0,0019995	
ENSGALG00000007300	11025,546	-0,764508198	0,1238668	-6,172016	6,74E-10	1,63E-08	PYCR2
ENSGALG00000026862	22574,745	-0,764882325	0,1624148	-4,709438	2,48E-06	2,94E-05	CLDN1
ENSGALG00000008621	9873,262	-0,76612038	0,131649	-5,819416	5,91E-09	1,22E-07	NRP2
ENSGALG00000027389	3852,8154	-0,766788012	0,1308552	-5,85982	4,63E-09	9,83E-08	RGS7BP
ENSGALG00000010598	220,78888	-0,767088888	0,2736996	-2,802667	0,0050682	0,0202892	KCNK10
ENSGALG00000013782	3281,2809	-0,769533335	0,1760353	-4,371472	1,23E-05	0,0001244	CDH7
ENSGALG00000026897	591,30007	-0,770711311	0,2031153	-3,794453	0,000148	0,0010531	
ENSGALG00000028737	5045,3512	-0,771568179	0,1454081	-5,306224	1,12E-07	1,78E-06	
ENSGALG00000000317	6552,3317	-0,771905791	0,3101223	-2,489037	0,012809	0,0427961	NEFM
ENSGALG00000026518	766,81567	-0,773283921	0,178411	-4,334283	1,46E-05	0,000144	RUNDC3A
ENSGALG00000005610	640,25249	-0,773621314	0,242648	-3,188245	0,0014314	0,0071802	SLC44A3
ENSGALG00000004594	1038,5627	-0,774792529	0,2089107	-3,708727	0,0002083	0,0014145	CD74
ENSGALG00000013627	453,51839	-0,775913381	0,2108029	-3,680753	0,0002325	0,0015558	SLC7A2
ENSGALG00000015887	12708,103	-0,776552733	0,129241	-6,008564	1,87E-09	4,28E-08	URB1
ENSGALG00000015208	6752,9307	-0,778489641	0,1611505	-4,830824	1,36E-06	1,72E-05	ROR2
ENSGALG00000006258	9759,6638	-0,778635815	0,1224981	-6,356312	2,07E-10	5,40E-09	SPOCK1
ENSGALG00000009514	5928,6059	-0,779501664	0,1147864	-6,790888	1,11E-11	3,50E-10	NRCAM
ENSGALG00000001020	7177,3339	-0,780112538	0,1084839	-7,191044	6,43E-13	2,41E-11	KIFC3

ENSGALG00000013683	18236,905	-0,780228434	0,1531512	-5,094499	3,50E-07	5,02E-06	ARID1B
ENSGALG00000008961	32098,143	-0,780468343	0,1668861	-4,676652	2,92E-06	3,39E-05	PTPRZ1
ENSGALG00000012834	693,3013	-0,78090617	0,243104	-3,212231	0,0013171	0,0067111	AKR1D1
ENSGALG00000024964	133,81844	-0,781593119	0,3102875	-2,518932	0,0117711	0,0399966	
ENSGALG00000009393	806,58478	-0,782303357	0,1913491	-4,088356	4,34E-05	0,0003633	CAV1
ENSGALG00000012329	10571,297	-0,782765701	0,1633862	-4,790892	1,66E-06	2,05E-05	GLI3
ENSGALG00000017864	140,93672	-0,783664392	0,2920232	-2,683569	0,0072841	0,0272978	
ENSGALG00000026970	1108,6536	-0,785311616	0,1563721	-5,022071	5,11E-07	7,09E-06	
ENSGALG00000009252	7419,3312	-0,788959505	0,1242797	-6,348257	2,18E-10	5,65E-09	PLD1
ENSGALG00000012808	5957,06	-0,789210521	0,1360454	-5,801083	6,59E-09	1,35E-07	CDYL
ENSGALG00000006908	14243,327	-0,789724754	0,1486312	-5,313318	1,08E-07	1,72E-06	DLG1
ENSGALG00000000639	611,51653	-0,79001541	0,2261059	-3,494006	0,0004758	0,0028767	LRRN2
ENSGALG00000008411	91,4625	-0,790605178	0,3210824	-2,462313	0,0138044	0,0452558	GHRL
ENSGALG00000016412	2161,4314	-0,791360693	0,1346388	-5,877656	4,16E-09	8,94E-08	MBOAT2
ENSGALG00000005785	2650,4252	-0,791416097	0,1851664	-4,274081	1,92E-05	0,0001807	UBAP2
ENSGALG00000009428	6528,6197	-0,792261942	0,1416583	-5,592767	2,23E-08	4,11E-07	SUSD6
ENSGALG00000004662	3631,9487	-0,792400882	0,1664941	-4,759334	1,94E-06	2,35E-05	DISP3
ENSGALG00000001995	9089,1491	-0,794307619	0,1100185	-7,219767	5,21E-13	1,99E-11	LIMS2
ENSGALG00000012317	261,45531	-0,794799886	0,2552487	-3,113826	0,0018468	0,008807	ADGB
ENSGALG00000003470	221,25413	-0,795180239	0,2878091	-2,762873	0,0057295	0,0223837	ZMAT4
ENSGALG00000025464	234,06627	-0,795229903	0,2531202	-3,141708	0,0016797	0,0081615	
ENSGALG00000009808	209,51024	-0,795296038	0,2773049	-2,867948	0,0041314	0,0171336	AVPR1A
ENSGALG00000012178	117,35655	-0,79634844	0,3097478	-2,570958	0,0101418	0,0355602	PDGFB
ENSGALG00000004132	38489,958	-0,797075856	0,1145646	-6,957436	3,47E-12	1,17E-10	UHRF1
ENSGALG00000026486	578,46167	-0,797913674	0,2123431	-3,757662	0,0001715	0,0012006	
ENSGALG00000008378	25019,971	-0,798096236	0,1224811	-6,516075	7,22E-11	2,01E-09	SRGAP3
ENSGALG00000009932	6037,7104	-0,798314026	0,1391751	-5,736039	9,69E-09	1,92E-07	THADA
ENSGALG00000011045	4900,9878	-0,799075807	0,1382254	-5,780962	7,43E-09	1,50E-07	AFF1
ENSGALG00000014907	2917,0076	-0,80019622	0,1504507	-5,318662	1,05E-07	1,68E-06	DCBLD1
ENSGALG00000010910	3629,9835	-0,801671392	0,1211047	-6,619657	3,60E-11	1,04E-09	RAPGEF5
ENSGALG00000012576	1359,5745	-0,801916632	0,1553997	-5,160349	2,46E-07	3,65E-06	RASEF
ENSGALG00000000583	9340,9356	-0,802342978	0,1195873	-6,709264	1,96E-11	5,94E-10	SOX13
ENSGALG00000017044	315,56866	-0,803518305	0,2643771	-3,039289	0,0023714	0,0108489	TRPC4
ENSGALG00000002900	2509,7754	-0,803716995	0,1458872	-5,509169	3,61E-08	6,34E-07	NOTUM
ENSGALG00000027521	307,65741	-0,803725098	0,23936	-3,357809	0,0007856	0,0043909	ANKRD33B
ENSGALG00000013212	21085,124	-0,805085082	0,1156431	-6,961806	3,36E-12	1,14E-10	TMEFF1
ENSGALG00000001883	2479,1556	-0,805297687	0,1269406	-6,343891	2,24E-10	5,80E-09	ST6GALNAC2
ENSGALG00000003105	820,42554	-0,805784417	0,1904502	-4,230946	2,33E-05	0,0002134	ANKFN1
ENSGALG00000002461	9290,0239	-0,808165073	0,1405519	-5,749941	8,93E-09	1,77E-07	MMP17
ENSGALG00000002242	344,64399	-0,808545932	0,229428	-3,524182	0,0004248	0,0026217	GALNT9
ENSGALG00000017103	2337,4348	-0,811501513	0,1279991	-6,339902	2,30E-10	5,93E-09	WASF3
ENSGALG00000014957	24709,132	-0,81285735	0,1124851	-7,226354	4,96E-13	1,90E-11	WDR1
ENSGALG00000003427	8550,6609	-0,813323099	0,1234103	-6,590399	4,39E-11	1,25E-09	USP3
ENSGALG00000001136	15067,474	-0,813551169	0,3048155	-2,668996	0,0076078	0,0283008	
ENSGALG00000011089	415,15739	-0,814043658	0,2251011	-3,616347	0,0002988	0,0019353	IFIH1
ENSGALG00000011643	20679,582	-0,816325927	0,1468695	-5,558172	2,73E-08	4,94E-07	UTP20
ENSGALG00000010785	498,93848	-0,818155142	0,1993699	-4,103705	4,07E-05	0,0003453	ACOT11
ENSGALG00000001220	3875,7891	-0,818155857	0,1299079	-6,297969	3,02E-10	7,62E-09	HERPUD1
ENSGALG00000002598	7963,9328	-0,82047281	0,1085944	-7,555386	4,18E-14	1,82E-12	TPST1
ENSGALG00000011511	101055,81	-0,820640197	0,126541	-6,485172	8,86E-11	2,44E-09	CKB
ENSGALG00000003160	1417,9918	-0,823423786	0,1758829	-4,68166	2,85E-06	3,32E-05	CERS1
ENSGALG00000026370	123,7781	-0,823508807	0,3145625	-2,61795	0,008846	0,0318788	
ENSGALG00000006872	8998,3245	-0,823650254	0,1148886	-7,169119	7,55E-13	2,79E-11	PISD
ENSGALG00000007039	11318,955	-0,823801989	0,1338606	-6,154178	7,55E-10	1,82E-08	IGF1R
ENSGALG00000004569	2222,4465	-0,82424563	0,1654621	-4,981477	6,31E-07	8,53E-06	UNC5B
ENSGALG00000010507	1443,24	-0,824303692	0,1501991	-5,488074	4,06E-08	7,08E-07	FCRL2
ENSGALG00000011623	9810,9245	-0,826439103	0,3226307	-2,561564	0,0104202	0,0363586	ADAMTS3
ENSGALG00000003391	10590,452	-0,827807721	0,1094916	-7,56047	4,02E-14	1,76E-12	EIF2AK1
ENSGALG00000017855	167,53221	-0,830680639	0,3021834	-2,748929	0,005979	0,0232054	
ENSGALG00000009546	1643,0136	-0,832251183	0,1524822	-5,45802	4,81E-08	8,27E-07	HTRA1
ENSGALG00000007717	242,86019	-0,834765713	0,2552035	-3,270981	0,0010718	0,0056624	GCNT7
ENSGALG00000014954	7661,4102	-0,835213371	0,1158293	-7,210726	5,57E-13	2,10E-11	ZNF518B

ENSGALG00000008469	4008,4217	-0,83875822	0,1497579	-5,600763	2,13E-08	3,94E-07	SPINT1
ENSGALG00000002543	5887,0903	-0,840502021	0,1240047	-6,777985	1,22E-11	3,81E-10	CSGALNACT2
ENSGALG00000015980	410,78574	-0,841274637	0,2500328	-3,364658	0,0007664	0,004294	
ENSGALG00000011388	12926,485	-0,842306194	0,1210688	-6,957254	3,47E-12	1,17E-10	RABGGTB
ENSGALG00000004627	119,19825	-0,843337638	0,3097612	-2,722542	0,0064782	0,0248312	LAMC2
ENSGALG00000015519	4471,9334	-0,844394446	0,1245073	-6,781886	1,19E-11	3,71E-10	ROBO2
ENSGALG00000015656	12813,534	-0,849003497	0,1245832	-6,814749	9,44E-12	3,00E-10	AKAP2
ENSGALG00000012998	2617,3459	-0,849149352	0,1768308	-4,802043	1,57E-06	1,96E-05	WNT5B
ENSGALG00000024984	247,00772	-0,849437052	0,2463411	-3,448214	0,0005643	0,0033217	
ENSGALG00000023609	5841,3037	-0,855145185	0,1351075	-6,329368	2,46E-10	6,31E-09	
ENSGALG00000014178	3963,5155	-0,855544377	0,1502564	-5,693895	1,24E-08	2,41E-07	SCUBE1
ENSGALG00000012394	13948,867	-0,855781498	0,1036166	-8,259118	1,47E-16	8,54E-15	NID2
ENSGALG00000012873	33,50313	-0,858492519	0,3501442	-2,451826	0,0142133	0,0463619	SERPINB5
ENSGALG00000003668	1594,4405	-0,85921128	0,1422579	-6,039814	1,54E-09	3,57E-08	PITPNC1
ENSGALG00000007846	1019,2888	-0,863598394	0,2308913	-3,740281	0,0001838	0,0012722	
ENSGALG00000010311	430,97249	-0,865329105	0,2059571	-4,201502	2,65E-05	0,0002391	NAV3
ENSGALG00000015681	7286,4362	-0,869123123	0,1385988	-6,270786	3,59E-10	9,02E-09	NRIP1
ENSGALG00000010010	1250,7492	-0,869227214	0,1534764	-5,66359	1,48E-08	2,84E-07	RHOQ
ENSGALG00000008815	1450,4816	-0,86979829	0,1663609	-5,22838	1,71E-07	2,63E-06	LRRN4
ENSGALG00000011376	402,72686	-0,870171475	0,2115735	-4,112858	3,91E-05	0,0003342	ANKRD9
ENSGALG00000020703	83,715675	-0,87414394	0,3266079	-2,676432	0,0074411	0,0277943	GRB14
ENSGALG00000016896	2175,6547	-0,874558686	0,1602235	-5,458368	4,81E-08	8,26E-07	ABCC4
ENSGALG00000016981	36,264584	-0,876405769	0,3539545	-2,476041	0,0132848	0,0439973	SPERT
ENSGALG00000023581	218,88299	-0,876769713	0,2786993	-3,145934	0,0016556	0,0080646	
ENSGALG00000009129	1209,8208	-0,878296092	0,1715571	-5,119557	3,06E-07	4,43E-06	DLX5
ENSGALG00000004542	7447,8359	-0,879244031	0,1445917	-6,080875	1,20E-09	2,81E-08	ZMYND8
ENSGALG00000016462	9552,6988	-0,880157248	0,1399598	-6,288642	3,20E-10	8,07E-09	MYCN
ENSGALG00000028602	5443,1954	-0,881510014	0,1576328	-5,592172	2,24E-08	4,12E-07	
ENSGALG00000011120	494,96982	-0,88227849	0,2357487	-3,742453	0,0001822	0,0012646	SLC4A10
ENSGALG00000003657	764,547	-0,88270712	0,1987799	-4,440625	8,97E-06	9,38E-05	UNC119
ENSGALG00000027633	124,92778	-0,88394595	0,3059642	-2,88905	0,0038641	0,0162676	RASSF9
ENSGALG00000028320	417,90377	-0,88451445	0,2720255	-3,251586	0,0011476	0,0059985	
ENSGALG00000001749	4549,2907	-0,88875485	0,1275791	-6,966303	3,25E-12	1,11E-10	ACSBG2
ENSGALG00000005723	2016,6496	-0,890647194	0,1398661	-6,367856	1,92E-10	5,03E-09	RP11-96O20.4
ENSGALG00000009360	602,38543	-0,890830846	0,1924613	-4,628624	3,68E-06	4,19E-05	CAPN2
ENSGALG00000002555	1358,1498	-0,892902927	0,2116768	-4,218237	2,46E-05	0,0002247	RET
ENSGALG00000000307	8913,0835	-0,894951246	0,1134627	-7,887626	3,08E-15	1,50E-13	CERS4
ENSGALG00000012012	5383,1455	-0,895703636	0,1725569	-5,190772	2,09E-07	3,15E-06	CMIP
ENSGALG00000017870	493,11834	-0,896459218	0,2052027	-4,368652	1,25E-05	0,0001254	
ENSGALG00000008547	11178,2	-0,896850751	0,1262491	-7,103817	1,21E-12	4,41E-11	ZBED4
ENSGALG00000008509	336,23193	-0,897122538	0,2669373	-3,360799	0,0007772	0,004349	SEMA3E
ENSGALG00000012814	2520,1319	-0,898069575	0,1279271	-7,020165	2,22E-12	7,76E-11	PXDC1
ENSGALG00000017838	224,75144	-0,898451215	0,2607674	-3,445413	0,0005702	0,0033455	
ENSGALG00000027269	123,982	-0,898861923	0,3460342	-2,59761	0,0093875	0,0333927	GUCA2B
ENSGALG00000009687	2155,9656	-0,89897016	0,1572674	-5,71619	1,09E-08	2,13E-07	KCNK2
ENSGALG00000014645	469,37544	-0,89901657	0,2175335	-4,132773	3,58E-05	0,0003098	MEF2C
ENSGALG00000006498	20254,306	-0,899416586	0,1691498	-5,317278	1,05E-07	1,69E-06	PTPRN2
ENSGALG00000013149	142,93297	-0,899640208	0,2949853	-3,04978	0,0022901	0,0105372	MOCOS
ENSGALG00000025723	123,40432	-0,903219239	0,3196649	-2,825519	0,0047204	0,0191048	NKX1-1
ENSGALG00000026790	419,69129	-0,903869706	0,2560648	-3,529848	0,0004158	0,0025729	DMRT2
ENSGALG00000002704	377,64333	-0,906514113	0,2414033	-3,755185	0,0001732	0,0012112	AFAP1L1
ENSGALG00000027960	55,099779	-0,908090032	0,3479917	-2,609516	0,009067	0,0324691	GRPR
ENSGALG00000017876	51,084697	-0,908586905	0,3511323	-2,587592	0,0096649	0,0342054	
ENSGALG00000026258	289,1614	-0,909500707	0,2404705	-3,782171	0,0001555	0,0011001	
ENSGALG00000004231	640,94875	-0,911405982	0,183472	-4,967547	6,78E-07	9,10E-06	IFNLR1
ENSGALG00000013861	1063,5397	-0,913164571	0,1795586	-5,085609	3,66E-07	5,22E-06	TNFAIP3
ENSGALG00000020723	828,74872	-0,913543779	0,1755447	-5,204053	1,95E-07	2,96E-06	NOG2
ENSGALG00000019622	6843,9055	-0,918336915	0,3199295	-2,870435	0,0040991	0,0170409	TRIM71
ENSGALG00000005994	969,59836	-0,91862167	0,1776308	-5,171523	2,32E-07	3,46E-06	DERL3
ENSGALG00000010683	599,9885	-0,919156764	0,2019322	-4,551809	5,32E-06	5,85E-05	S1PR3
ENSGALG00000001656	26148,058	-0,922800056	0,1104664	-8,35367	6,62E-17	4,00E-15	sept-09
ENSGALG00000009540	5865,4242	-0,9229711	0,1165261	-7,920723	2,36E-15	1,17E-13	YAF2

ENSGALG00000025166	165,3461	-0,924288426	0,2874597	-3,215367	0,0013028	0,0066557	
ENSGALG00000001000	110996,8	-0,924720639	0,1374659	-6,726907	1,73E-11	5,28E-10	HSPA5
ENSGALG00000008305	1229,5865	-0,925580955	0,1854564	-4,990827	6,01E-07	8,18E-06	CASP10
ENSGALG00000016843	7409,4478	-0,926939677	0,1285541	-7,210501	5,57E-13	2,10E-11	COL4A2
ENSGALG00000023100	28,255969	-0,928527718	0,3431483	-2,705908	0,0068118	0,025848	
ENSGALG00000014961	70,382782	-0,928667888	0,3536337	-2,626073	0,0086376	0,0312686	FAM26E
ENSGALG00000009262	127,08834	-0,928869092	0,3039249	-3,056245	0,0022413	0,0103476	FGB
ENSGALG00000012911	418,92558	-0,929872331	0,2601161	-3,574836	0,0003504	0,0022213	SYT10
ENSGALG00000008811	6177,8009	-0,931412058	0,1489053	-6,255065	3,97E-10	9,87E-09	SLC1A4
ENSGALG00000007952	17655,501	-0,932963194	0,1344416	-6,939543	3,93E-12	1,32E-10	AMOT
ENSGALG00000023241	1184,6759	-0,938819916	0,1882587	-4,986861	6,14E-07	8,32E-06	
ENSGALG00000028708	6182,0112	-0,938854547	0,1258631	-7,459329	8,70E-14	3,59E-12	
ENSGALG00000009415	20765,099	-0,939656238	0,1402127	-6,701647	2,06E-11	6,21E-10	SMOC1
ENSGALG00000012726	95084,968	-0,941672551	0,1261141	-7,466831	8,21E-14	3,42E-12	HSP90B1
ENSGALG00000015622	657,0594	-0,944352456	0,2173775	-4,344298	1,40E-05	0,0001385	EYA1
ENSGALG00000015028	404,3337	-0,946739996	0,2500725	-3,785863	0,0001532	0,0010858	RLN1
ENSGALG00000011258	44996,798	-0,946927699	0,1449344	-6,533494	6,43E-11	1,81E-09	ATP2B1
ENSGALG00000011068	1649,8566	-0,947751489	0,1445198	-6,557937	5,46E-11	1,54E-09	COBLL1
ENSGALG00000005601	522,73024	-0,948098308	0,2254007	-4,206279	2,60E-05	0,0002346	DNAH12
ENSGALG00000006808	9236,5969	-0,948868926	0,1071422	-8,856165	8,28E-19	6,00E-17	TSPAN6
ENSGALG00000013193	667,61334	-0,948945842	0,2164896	-4,383332	1,17E-05	0,0001183	IRX2
ENSGALG00000023608	3676,9857	-0,950348621	0,134551	-7,06311	1,63E-12	5,82E-11	
ENSGALG00000027260	44,084585	-0,951699131	0,3538862	-2,68928	0,0071606	0,0269519	MC3R
ENSGALG00000005805	4566,7573	-0,954718819	0,1455167	-6,560889	5,35E-11	1,52E-09	PLCD1
ENSGALG00000023463	1598,1146	-0,956997326	0,1712907	-5,586978	2,31E-08	4,24E-07	
ENSGALG00000017875	78,294933	-0,958367374	0,3317498	-2,888825	0,0038668	0,0162692	
ENSGALG00000002577	53,909401	-0,959612138	0,3537853	-2,712414	0,0066795	0,025481	STARD10
ENSGALG00000004393	1366,8686	-0,962441655	0,2126961	-4,524962	6,04E-06	6,51E-05	
ENSGALG00000004284	19106,512	-0,967926178	0,1544238	-6,267988	3,66E-10	9,15E-09	LFNG
ENSGALG00000027342	11330,786	-0,968330352	0,1154603	-8,386697	5,00E-17	3,08E-15	METRN
ENSGALG00000015908	1062,9623	-0,970529728	0,2034554	-4,770232	1,84E-06	2,24E-05	COL12A1
ENSGALG00000011833	13327,666	-0,971207285	0,1137465	-8,538346	1,36E-17	8,83E-16	SPATA5
ENSGALG00000009381	15338,368	-0,972226453	0,1330298	-7,308336	2,70E-13	1,06E-11	SIPA1L1
ENSGALG00000015940	2397,4582	-0,972506334	0,1424398	-6,827491	8,64E-12	2,76E-10	TMEM67
ENSGALG00000007367	230,09823	-0,973082329	0,2646976	-3,676204	0,0002367	0,0015799	WFIKKN2
ENSGALG00000010597	2446,5354	-0,973101915	0,127224	-7,64873	2,03E-14	9,27E-13	CRIM1
ENSGALG00000027497	292,35552	-0,973179721	0,2340886	-4,157313	3,22E-05	0,0002823	CFD
ENSGALG00000019397	12676,413	-0,975439675	0,1148854	-8,490543	2,06E-17	1,31E-15	CERK
ENSGALG00000026995	97,429549	-0,97623355	0,3272256	-2,983365	0,002851	0,0126313	RHOG
ENSGALG00000002103	289,28254	-0,979001464	0,2861261	-3,421574	0,0006226	0,0035994	TPPP3
ENSGALG00000015624	34457,429	-0,97910183	0,1758979	-5,566309	2,60E-08	4,72E-07	VCAN
ENSGALG00000012418	35,255982	-0,979401863	0,3538633	-2,767741	0,0056446	0,0221474	KYNU
ENSGALG00000015164	3228,2862	-0,982829392	0,1480222	-6,639743	3,14E-11	9,24E-10	PCSK5
ENSGALG00000020788	1411,7356	-0,986044977	0,148882	-6,622998	3,52E-11	1,02E-09	CCDC141
ENSGALG00000001371	2323,3283	-0,990350626	0,1647412	-6,011554	1,84E-09	4,21E-08	
ENSGALG00000009758	793,28474	-0,991410492	0,1927041	-5,144731	2,68E-07	3,94E-06	SLC7A11
ENSGALG00000015914	35,887211	-0,993778818	0,3538558	-2,808429	0,0049784	0,0200099	CALB1
ENSGALG00000015440	170,65631	-0,996123586	0,2860343	-3,482532	0,0004967	0,0029843	RCSD1
ENSGALG00000010692	6955,6689	-0,996294098	0,1389198	-7,171722	7,41E-13	2,76E-11	LRP8
ENSGALG00000026582	1194,487	-0,997100015	0,1907735	-5,226618	1,73E-07	2,64E-06	SOX18
ENSGALG00000002532	116,30592	-0,997428466	0,3168923	-3,147531	0,0016466	0,0080322	GJA4
ENSGALG00000010163	10928,524	-0,998496131	0,1269077	-7,867891	3,61E-15	1,74E-13	LGR5
ENSGALG00000007483	18280,16	-0,999963194	0,1499281	-6,669618	2,56E-11	7,61E-10	LRIG1
ENSGALG00000005483	1190,7719	-1,005472997	0,1552955	-6,474578	9,51E-11	2,59E-09	CDH13
ENSGALG00000012454	37,219552	-1,005696393	0,3507698	-2,867112	0,0041424	0,017158	CYTH4
ENSGALG00000011295	1389,1449	-1,007421144	0,1580798	-6,372863	1,86E-10	4,90E-09	SOCS2
ENSGALG00000016627	15863,827	-1,008110823	0,1082287	-9,314637	1,22E-20	1,03E-18	FZD3
ENSGALG00000008281	355,49596	-1,009962725	0,2355243	-4,288147	1,80E-05	0,0001708	NEURL1
ENSGALG00000015078	5440,7576	-1,010765666	0,1823319	-5,543548	2,96E-08	5,32E-07	MLLT3
ENSGALG00000015112	8576,8627	-1,013051509	0,1332664	-7,601703	2,92E-14	1,31E-12	ZNF521
ENSGALG00000000949	297,0835	-1,013784064	0,2329319	-4,352276	1,35E-05	0,0001336	HBEGF
ENSGALG00000016616	1435,9249	-1,01551489	0,1675974	-6,059252	1,37E-09	3,18E-08	ANOS1

ENSGALG00000009450	2394,0442	-1,016064164	0,1296479	-7,837106	4,61E-15	2,19E-13	DUSP10
ENSGALG00000001009	527,81732	-1,018620159	0,2467658	-4,127883	3,66E-05	0,0003151	
ENSGALG00000015896	2100,3865	-1,023057651	0,1679504	-6,091426	1,12E-09	2,64E-08	MMP16
ENSGALG00000010156	274,63907	-1,025083823	0,2479625	-4,134028	3,56E-05	0,0003089	DOCK8
ENSGALG00000013124	3974,9034	-1,026819624	0,1623011	-6,326635	2,51E-10	6,40E-09	FHOD3
ENSGALG00000006138	2396,0525	-1,026936244	0,150452	-6,825674	8,75E-12	2,79E-10	HELZ2
ENSGALG00000010579	4573,0234	-1,028169899	0,1257094	-8,178939	2,86E-16	1,62E-14	STON2
ENSGALG00000012439	1314,5759	-1,028638845	0,2279249	-4,513061	6,39E-06	6,86E-05	IGFBP3
ENSGALG00000019077	11786,668	-1,030078317	0,1385218	-7,436218	1,04E-13	4,25E-12	
ENSGALG00000012522	26,038382	-1,03063871	0,345927	-2,979353	0,0028886	0,0127647	PVALB
ENSGALG00000000977	7324,7206	-1,032464943	0,1097312	-9,409039	5,01E-21	4,29E-19	MAPKAP1
ENSGALG00000016754	1717,8597	-1,034769759	0,1770332	-5,845059	5,06E-09	1,06E-07	MGAT4A
ENSGALG00000012412	7272,9925	-1,036824012	0,1452529	-7,138059	9,47E-13	3,47E-11	MTHFD1L
ENSGALG00000027213	84,217699	-1,037130822	0,3364624	-3,082456	0,002053	0,0096518	ALX1
ENSGALG00000007555	13407,29	-1,038877738	0,1147853	-9,050617	1,42E-19	1,11E-17	CCND1
ENSGALG00000025284	64,080075	-1,04409147	0,3409884	-3,061956	0,002199	0,0102076	
ENSGALG00000008465	8001,1338	-1,045042375	0,1298809	-8,046158	8,54E-16	4,60E-14	SORCS1
ENSGALG00000022800	1489,665	-1,045072381	0,145669	-7,174293	7,27E-13	2,72E-11	KCNB2
ENSGALG00000011336	49,782944	-1,048887527	0,3535455	-2,966768	0,0030095	0,0132004	TECRL
ENSGALG00000015008	37,265424	-1,049053854	0,3418003	-3,069201	0,0021463	0,0100076	ZNF366
ENSGALG00000023355	72,955518	-1,050070076	0,34512	-3,042623	0,0023453	0,0107547	TMEM154
ENSGALG00000017714	665,00414	-1,052113718	0,2083331	-5,050151	4,41E-07	6,20E-06	FHAD1
ENSGALG00000015975	5069,4191	-1,056406602	0,1284565	-8,223848	1,97E-16	1,13E-14	DPY19L4
ENSGALG00000011581	10286,837	-1,059360643	0,338584	-3,128797	0,0017552	0,0084616	KIF26A
ENSGALG00000015736	279,62808	-1,060236168	0,3192341	-3,321187	0,0008964	0,0048849	STMN2
ENSGALG00000003295	2261,7027	-1,063705711	0,1585827	-6,707576	1,98E-11	6,00E-10	ADAMTS12
ENSGALG00000009740	783,87139	-1,063900656	0,1815827	-5,859043	4,66E-09	9,86E-08	RASGRP1
ENSGALG00000013604	164,1582	-1,065244459	0,2852669	-3,734202	0,0001883	0,0012975	VIP
ENSGALG00000016470	856,35527	-1,066533888	0,3521055	-3,029018	0,0024535	0,0111534	KCNS3
ENSGALG00000011236	190,29168	-1,070413941	0,315055	-3,397546	0,0006799	0,0038831	BEGAIN
ENSGALG00000017135	1406,4237	-1,071209509	0,1612008	-6,645186	3,03E-11	8,92E-10	CRYL1
ENSGALG00000002988	7561,0439	-1,072357906	0,152099	-7,050395	1,78E-12	6,33E-11	PHGDH
ENSGALG00000001370	1171,2311	-1,073396904	0,1915947	-5,602436	2,11E-08	3,91E-07	ADAMTS8
ENSGALG00000010707	29,836376	-1,074169362	0,351316	-3,057559	0,0022315	0,0103145	GPR68
ENSGALG00000027757	10827,926	-1,076478944	0,1080166	-9,965866	2,15E-23	2,27E-21	ARL4A
ENSGALG00000010891	1573,4126	-1,079203445	0,1845381	-5,848133	4,97E-09	1,04E-07	ABCB11
ENSGALG00000027707	112,77294	-1,082314729	0,3118106	-3,471064	0,0005184	0,0030902	ESM1
ENSGALG00000016899	32,641313	-1,08275528	0,3526736	-3,070135	0,0021396	0,0099866	DCT
ENSGALG00000000177	4145,0213	-1,083701806	0,1219668	-8,885217	6,38E-19	4,70E-17	KLF3
ENSGALG00000025770	345,96122	-1,089960517	0,2525089	-4,316523	1,59E-05	0,0001532	
ENSGALG00000028046	6843,8734	-1,098357134	0,1139847	-9,636006	5,63E-22	5,11E-20	INSIG2
ENSGALG00000012917	4548,5783	-1,102419391	0,1907106	-5,780587	7,44E-09	1,50E-07	CDH6
ENSGALG00000005842	443,93623	-1,104947048	0,2118757	-5,215072	1,84E-07	2,80E-06	GDPD2
ENSGALG00000012095	202,10968	-1,108243378	0,2716377	-4,079858	4,51E-05	0,0003753	C14orf105
ENSGALG00000002518	128,6744	-1,109129449	0,3369249	-3,291919	0,0009951	0,0053108	
ENSGALG00000009567	5248,5052	-1,113206952	0,1654659	-6,727712	1,72E-11	5,27E-10	TLL1
ENSGALG00000011439	1241,3905	-1,116853678	0,1622406	-6,883936	5,82E-12	1,90E-10	RBMS3
ENSGALG00000013548	35,231247	-1,118469658	0,3386376	-3,302851	0,0009571	0,0051565	GZMA
ENSGALG00000014852	15743,642	-1,119019515	0,1113638	-10,04833	9,34E-24	1,05E-21	
ENSGALG00000009179	4347,973	-1,119360692	0,1337923	-8,366405	5,94E-17	3,63E-15	TNFSF10
ENSGALG00000011172	2541,5225	-1,121518184	0,1274182	-8,801871	1,35E-18	9,35E-17	MYO10L
ENSGALG00000013892	1340,4062	-1,122648268	0,1612643	-6,961542	3,37E-12	1,14E-10	MAP3K5
ENSGALG00000001079	117,89095	-1,122757301	0,3356009	-3,345513	0,0008213	0,0045345	WNT3
ENSGALG00000016680	793,76813	-1,12428894	0,234202	-4,80051	1,58E-06	1,97E-05	TFAP2B
ENSGALG00000008014	284,58059	-1,126068911	0,2641663	-4,262727	2,02E-05	0,0001892	CEBPB
ENSGALG00000006337	35,98151	-1,128489411	0,3491935	-3,231702	0,0012306	0,0063486	K123
ENSGALG00000020151	65,103733	-1,129234483	0,3522369	-3,205895	0,0013464	0,0068343	NMU
ENSGALG00000006376	107,4182	-1,138122247	0,3273909	-3,476341	0,0005083	0,0030393	TMC3
ENSGALG00000027210	288,32435	-1,142415301	0,2380637	-4,798779	1,60E-06	1,98E-05	
ENSGALG00000001085	2988,7865	-1,143093103	0,1311931	-8,71306	2,96E-18	1,98E-16	ADGRG1
ENSGALG00000001276	18773,518	-1,144775826	0,131613	-8,698048	3,38E-18	2,24E-16	HOXB9
ENSGALG00000004155	218,30979	-1,147734088	0,2762334	-4,154943	3,25E-05	0,0002845	MYOM3

ENSGALG00000027107	1206,9544	-1,149521147	0,1567788	-7,332123	2,27E-13	9,02E-12	PROCA1
ENSGALG00000014864	769,88057	-1,150010609	0,170127	-6,759718	1,38E-11	4,28E-10	SMPDL3A
ENSGALG00000027885	31,983561	-1,151437329	0,3494378	-3,295114	0,0009838	0,0052611	C4orf19
ENSGALG00000010926	145,65	-1,152009571	0,3159768	-3,645868	0,0002665	0,0017503	SPP1
ENSGALG00000008875	1752,669	-1,156831558	0,1589922	-7,276029	3,44E-13	1,34E-11	PLCB1
ENSGALG00000007268	3702,3293	-1,168892699	0,1417063	-8,248697	1,60E-16	9,28E-15	CADPS
ENSGALG00000006584	535,75964	-1,169466644	0,2313724	-5,054478	4,32E-07	6,07E-06	
ENSGALG00000016114	1343,2019	-1,174926553	0,198016	-5,933492	2,97E-09	6,53E-08	TNFRSF11B
ENSGALG00000022871	818,02057	-1,176605905	0,1867706	-6,299737	2,98E-10	7,55E-09	HHLA2
ENSGALG00000026763	724,90274	-1,177313255	0,2069483	-5,688924	1,28E-08	2,48E-07	
ENSGALG00000017411	73,326206	-1,17758282	0,3486458	-3,377591	0,0007312	0,0041242	RXFP3
ENSGALG00000016998	449,62073	-1,177970615	0,2144018	-5,494221	3,92E-08	6,86E-07	
ENSGALG00000008941	29,808839	-1,184874519	0,3508426	-3,377225	0,0007322	0,0041263	FEZF1
ENSGALG00000002509	14719,435	-1,185807324	0,1326958	-8,936281	4,02E-19	2,98E-17	COL5A2
ENSGALG00000012357	5240,5441	-1,187472008	0,1225271	-9,691503	3,28E-22	3,16E-20	CXCR4
ENSGALG00000015665	1052,1425	-1,188036426	0,1715856	-6,923872	4,39E-12	1,45E-10	SH3BP2
ENSGALG00000005619	863,25589	-1,189698364	0,2239703	-5,311858	1,09E-07	1,73E-06	F3
ENSGALG00000005685	3302,1261	-1,189953127	0,1570606	-7,576394	3,55E-14	1,57E-12	KSR1
ENSGALG00000016083	216,72251	-1,190269979	0,2711704	-4,38938	1,14E-05	0,0001155	ANGPT1
ENSGALG00000000936	1054,1306	-1,193444435	0,1573416	-7,585055	3,32E-14	1,47E-12	LMX1B
ENSGALG00000006170	3710,8245	-1,194070483	0,1488786	-8,020428	1,05E-15	5,52E-14	LRRC8C
ENSGALG00000015433	4115,112	-1,199115509	0,1254233	-9,560547	1,17E-21	1,05E-19	ABCA1
ENSGALG00000002923	1146,0005	-1,199213505	0,1576913	-7,60482	2,85E-14	1,29E-12	
ENSGALG00000011104	29,588884	-1,203717219	0,3516022	-3,42352	0,0006182	0,0035829	GCG
ENSGALG00000012224	1928,9682	-1,20528636	0,1513933	-7,961295	1,70E-15	8,65E-14	PDE1C
ENSGALG00000003582	6407,4212	-1,218636528	0,1784672	-6,828349	8,59E-12	2,75E-10	SLC1A3
ENSGALG00000009667	660,39309	-1,219318734	0,2495248	-4,886564	1,03E-06	1,33E-05	MFAP3L
ENSGALG00000002632	1307,7165	-1,220611399	0,1739574	-7,016727	2,27E-12	7,93E-11	CALCRL
ENSGALG00000009215	122,78745	-1,220936874	0,3057355	-3,993442	6,51E-05	0,0005167	PDE11A
ENSGALG00000000141	479,97527	-1,223136562	0,2587692	-4,726747	2,28E-06	2,72E-05	BLB1
ENSGALG00000006473	340,04191	-1,223226887	0,2654847	-4,607524	4,07E-06	4,58E-05	PLXNA4B
ENSGALG00000007660	3604,7946	-1,228207524	0,1637881	-7,49876	6,44E-14	2,69E-12	CHIC1
ENSGALG00000013239	67578,529	-1,231185989	0,1344215	-9,159144	5,23E-20	4,24E-18	NES
ENSGALG00000008615	1056,6531	-1,233824562	0,1860614	-6,631275	3,33E-11	9,74E-10	LTK
ENSGALG00000003739	39854,675	-1,237172502	0,1212391	-10,2044	1,89E-24	2,28E-22	SALL1
ENSGALG00000029023	187,05762	-1,242039059	0,2692492	-4,612972	3,97E-06	4,48E-05	DMRTB1
ENSGALG00000009532	9821,7952	-1,242374232	0,113576	-10,9387	7,53E-28	1,13E-25	GXYLT1
ENSGALG00000011867	2059,3195	-1,242989678	0,1421856	-8,742025	2,29E-18	1,56E-16	SERHL2
ENSGALG00000005427	736,09619	-1,25622691	0,1850037	-6,790278	1,12E-11	3,51E-10	
ENSGALG00000013237	693,51796	-1,260226776	0,2152859	-5,853736	4,81E-09	1,01E-07	BCAN
ENSGALG00000010858	12929,131	-1,268608971	0,3211828	-3,949803	7,82E-05	0,0006051	LRP2
ENSGALG00000003353	12512,084	-1,269046359	0,175098	-7,247634	4,24E-13	1,63E-11	RAI14
ENSGALG00000019552	24,982545	-1,269295425	0,3440776	-3,688981	0,0002252	0,0015146	SERPINB2
ENSGALG00000004361	87800,454	-1,276561341	0,1268377	-10,06452	7,93E-24	8,99E-22	PRTG
ENSGALG00000008352	151,36281	-1,283975751	0,2919953	-4,397248	1,10E-05	0,0001121	CKMT1A
ENSGALG00000002397	837,30231	-1,291814757	0,2176946	-5,934069	2,96E-09	6,52E-08	PSPH
ENSGALG00000006401	4187,8898	-1,299022844	0,1294305	-10,03645	1,05E-23	1,16E-21	OSBPL5
ENSGALG00000014866	52452,789	-1,312013425	0,1313405	-9,989404	1,70E-23	1,83E-21	FABP7
ENSGALG00000005442	270,98777	-1,327990169	0,2831728	-4,689682	2,74E-06	3,20E-05	PALMD
ENSGALG00000016602	1659,682	-1,339565689	0,1637218	-8,181961	2,79E-16	1,58E-14	ARHGAP6
ENSGALG00000021879	40,043275	-1,34129965	0,3484949	-3,848836	0,0001187	0,0008659	
ENSGALG00000002443	492,98343	-1,353430658	0,2272211	-5,956448	2,58E-09	5,77E-08	FBXL16
ENSGALG00000007771	2716,4461	-1,353742442	0,1514834	-8,936574	4,01E-19	2,98E-17	FAM210B
ENSGALG00000008491	3635,9278	-1,354708919	0,1393335	-9,72278	2,41E-22	2,38E-20	XIAP
ENSGALG00000009947	4729,0815	-1,358616298	0,1191908	-11,39867	4,25E-30	7,32E-28	PLEKHH2
ENSGALG00000001346	421,97082	-1,362109597	0,2357782	-5,77708	7,60E-09	1,53E-07	
ENSGALG00000028048	17805,974	-1,362450241	0,1038968	-13,1135	2,76E-39	8,73E-37	PTN
ENSGALG00000028047	613,76602	-1,369008166	0,2271663	-6,026458	1,68E-09	3,86E-08	RHOV
ENSGALG00000010148	96,068007	-1,379685752	0,3285947	-4,198746	2,68E-05	0,0002417	PTPRR
ENSGALG00000003706	2880,2185	-1,390323026	0,1522007	-9,134799	6,55E-20	5,25E-18	DUSP1
ENSGALG00000009405	351,46777	-1,391691484	0,2252283	-6,179026	6,45E-10	1,57E-08	GRIA2
ENSGALG00000014983	1427,7077	-1,39278689	0,1752564	-7,947137	1,91E-15	9,66E-14	F2R

ENSGALG00000002993	1938,9589	-1,397301236	0,1581691	-8,834225	1,01E-18	7,22E-17	CA10
ENSGALG00000015256	9909,979	-1,397562325	0,1233891	-11,32647	9,70E-30	1,61E-27	PAM
ENSGALG00000003114	426,74171	-1,398035927	0,2162026	-6,466322	1,00E-10	2,72E-09	NOG
ENSGALG00000011306	2909,7094	-1,403496478	0,1324006	-10,60038	2,97E-26	4,17E-24	DES
ENSGALG00000027133	162,08932	-1,405319409	0,31973	-4,395332	1,11E-05	0,000113	CD83
ENSGALG00000001768	6648,8124	-1,406520646	0,156826	-8,968671	3,00E-19	2,27E-17	TENM2
ENSGALG00000005123	593,9961	-1,408198856	0,2224863	-6,329374	2,46E-10	6,31E-09	WNT7A
ENSGALG00000000680	6447,7598	-1,411570576	0,1125835	-12,53799	4,63E-36	1,19E-33	NUAK2
ENSGALG00000009530	697,31067	-1,411853584	0,1982451	-7,121758	1,07E-12	3,88E-11	PDZRN4
ENSGALG00000014343	783,22971	-1,412639292	0,1787261	-7,903934	2,70E-15	1,32E-13	ARAP2
ENSGALG00000026557	168,82638	-1,412974627	0,2911389	-4,853266	1,21E-06	1,55E-05	PGF
ENSGALG00000011319	10478,782	-1,42017882	0,126589	-11,21882	3,30E-29	5,34E-27	LPHN3
ENSGALG00000014823	40,51625	-1,422401118	0,3456571	-4,115064	3,87E-05	0,0003312	C6orf58
ENSGALG00000011468	1760,2982	-1,424650779	0,1875286	-7,596979	3,03E-14	1,36E-12	IGFBP5
ENSGALG00000016297	52,464778	-1,427639697	0,3539064	-4,033947	5,48E-05	0,0004458	HCRTR2
ENSGALG00000019054	529,71662	-1,427749976	0,2415069	-5,911839	3,38E-09	7,38E-08	NAALAD2
ENSGALG00000011110	296,25723	-1,431359085	0,2488071	-5,752887	8,77E-09	1,75E-07	DPP4
ENSGALG00000011207	13680,733	-1,434035643	0,1207094	-11,88007	1,50E-32	3,30E-30	DUSP6
ENSGALG00000012018	2349,8518	-1,435600968	0,1869145	-7,680524	1,58E-14	7,29E-13	UGT8
ENSGALG00000013402	51,692715	-1,446510354	0,3538534	-4,087881	4,35E-05	0,0003636	AHRR
ENSGALG00000011164	2326,4168	-1,446841804	0,2083124	-6,945539	3,77E-12	1,26E-10	CHN2
ENSGALG00000028821	11178,852	-1,46102831	0,1488545	-9,815142	9,69E-23	9,70E-21	GLIPR2
ENSGALG00000001169	20569,632	-1,469628578	0,1453759	-10,10916	5,03E-24	5,85E-22	CRB2
ENSGALG00000006072	43,451296	-1,471939488	0,3539382	-4,158747	3,20E-05	0,0002809	SCN10A
ENSGALG00000005855	6149,8313	-1,488520056	0,1624534	-9,162749	5,06E-20	4,12E-18	CA5B
ENSGALG00000026262	5433,5657	-1,4922838	0,1545273	-9,657086	4,59E-22	4,25E-20	PMP22
ENSGALG00000001621	458,45489	-1,495017381	0,2209806	-6,765379	1,33E-11	4,12E-10	CLDN5
ENSGALG00000011940	614,50292	-1,496267605	0,2191218	-6,828475	8,58E-12	2,75E-10	CCK
ENSGALG00000008978	15989,385	-1,505709648	0,1318417	-11,42059	3,30E-30	5,76E-28	ITGA4
ENSGALG00000025773	391,15903	-1,506718569	0,2193821	-6,868009	6,51E-12	2,11E-10	
ENSGALG00000015388	3557,5816	-1,507384513	0,1325751	-11,37004	5,90E-30	1,00E-27	PRDM1
ENSGALG00000007171	156,08231	-1,518198388	0,2937383	-5,168541	2,36E-07	3,50E-06	CIITA
ENSGALG00000010258	1087,7077	-1,528410178	0,2320488	-6,58659	4,50E-11	1,28E-09	LTBP2
ENSGALG00000006084	149,65329	-1,531133293	0,3082185	-4,967689	6,78E-07	9,10E-06	WNT8A
ENSGALG00000005499	52125,792	-1,531899327	0,1548752	-9,891183	4,55E-23	4,65E-21	IL17RD
ENSGALG00000001383	88,961213	-1,543511015	0,3420022	-4,513161	6,39E-06	6,86E-05	HTR4
ENSGALG00000017179	397,23584	-1,548130217	0,2756174	-5,616954	1,94E-08	3,65E-07	PDGFD
ENSGALG00000000388	10384,966	-1,555950834	0,1505748	-10,33341	4,98E-25	6,33E-23	LIN28A
ENSGALG00000016293	1486,8847	-1,561397543	0,1523922	-10,24591	1,23E-24	1,51E-22	BMP5
ENSGALG00000014657	1240,6807	-1,561923173	0,2131383	-7,328215	2,33E-13	9,26E-12	ADGRV1
ENSGALG00000003957	1745,618	-1,565814231	0,1495869	-10,46759	1,22E-25	1,66E-23	APOH
ENSGALG00000017242	1988,3309	-1,579394776	0,1359327	-11,61895	3,30E-31	6,42E-29	FZD4
ENSGALG00000009170	1955,3023	-1,581054934	0,1355952	-11,66011	2,04E-31	4,02E-29	NCEH1
ENSGALG00000003323	100,58975	-1,591926379	0,3209027	-4,960776	7,02E-07	9,41E-06	OSGIN1
ENSGALG00000001645	733,71496	-1,608468631	0,2050944	-7,842576	4,41E-15	2,10E-13	KCNT1
ENSGALG00000011609	3466,2373	-1,61436812	0,3265448	-4,943788	7,66E-07	1,02E-05	ABTB2
ENSGALG00000003126	1209,6364	-1,615974863	0,1617448	-9,990893	1,67E-23	1,82E-21	ERBB4
ENSGALG00000019716	86,528621	-1,618712644	0,3285879	-4,926269	8,38E-07	1,10E-05	KRT20
ENSGALG00000016102	256,08529	-1,630912312	0,2481459	-6,572392	4,95E-11	1,41E-09	CYP26B1
ENSGALG00000010468	1107,4297	-1,6309453	0,1573602	-10,36441	3,60E-25	4,67E-23	ISM2
ENSGALG00000023521	1102,9061	-1,651708778	0,1982919	-8,329684	8,11E-17	4,84E-15	ADRA2A
ENSGALG00000019261	1018,7346	-1,655584971	0,2240687	-7,388737	1,48E-13	6,04E-12	ARHGAP8
ENSGALG00000026892	80,007612	-1,6734567	0,3494002	-4,789512	1,67E-06	2,06E-05	
ENSGALG00000008353	2780,0954	-1,679225856	0,1922691	-8,733726	2,46E-18	1,67E-16	MAGI2
ENSGALG00000006997	745,7891	-1,684979264	0,1736039	-9,705885	2,85E-22	2,77E-20	BRINP1
ENSGALG00000029175	12384,251	-1,685259625	0,1660405	-10,14969	3,32E-24	3,90E-22	EFNB2
ENSGALG00000008918	724,31518	-1,685987772	0,1865069	-9,039814	1,57E-19	1,22E-17	LAMP5
ENSGALG00000014930	4308,5867	-1,696897635	0,1564042	-10,84944	2,01E-27	2,94E-25	ENC1
ENSGALG00000005498	1151,8463	-1,707286081	0,2153293	-7,928721	2,21E-15	1,11E-13	
ENSGALG00000014235	549,44433	-1,721709963	0,2641679	-6,517485	7,15E-11	1,99E-09	WNT7B
ENSGALG00000013102	1811,9189	-1,724325032	0,1396681	-12,34587	5,13E-35	1,25E-32	COBL
ENSGALG00000007218	6209,2721	-1,732428983	0,1735379	-9,982999	1,81E-23	1,94E-21	MB21D2

ENSGALG00000014908	2604,0347	-1,732825245	0,1633068	-10,61086	2,65E-26	3,76E-24	FST
ENSGALG00000005476	213,71162	-1,752170936	0,3180675	-5,508801	3,61E-08	6,35E-07	PTH1R
ENSGALG00000011148	2471,4965	-1,754606037	0,1499315	-11,70272	1,23E-31	2,51E-29	HHIPL1
ENSGALG00000027874	793,36454	-1,754956403	0,232655	-7,54317	4,59E-14	1,97E-12	CHAC1
ENSGALG00000012635	1110,8535	-1,755097361	0,1664549	-10,54398	5,42E-26	7,45E-24	BTBD11
ENSGALG00000006995	175,82725	-1,759714805	0,3023936	-5,819285	5,91E-09	1,22E-07	APOD
ENSGALG00000027316	126,11379	-1,764476803	0,3121114	-5,653356	1,57E-08	3,00E-07	CRNN
ENSGALG00000005519	2075,7509	-1,767947956	0,3335103	-5,30103	1,15E-07	1,82E-06	TLL2
ENSGALG00000009282	5108,519	-1,769054515	0,16415	-10,77706	4,42E-27	6,40E-25	ELMSAN1
ENSGALG00000007113	2619,5663	-1,77145981	0,1909494	-9,277115	1,74E-20	1,45E-18	TNC
ENSGALG00000015148	101,3242	-1,772924708	0,3468771	-5,111103	3,20E-07	4,62E-06	ANXA1
ENSGALG00000023406	3748,5665	-1,774894708	0,1767754	-10,04039	1,01E-23	1,12E-21	
ENSGALG00000004930	259,83629	-1,782009807	0,3260345	-5,465709	4,61E-08	7,94E-07	HRH1
ENSGALG00000009920	1810,7316	-1,80046145	0,2059481	-8,742306	2,28E-18	1,56E-16	COCH
ENSGALG00000014159	18203,792	-1,81424576	0,1061679	-17,08846	1,81E-65	1,30E-62	PACSIN2
ENSGALG00000016875	1624,3852	-1,825016077	0,1855323	-9,836647	7,83E-23	7,89E-21	CLYBL
ENSGALG00000004306	1596,7644	-1,830041848	0,1566925	-11,67919	1,63E-31	3,26E-29	SYT12
ENSGALG00000011708	13494,005	-1,833902608	0,1212478	-15,12524	1,10E-51	6,01E-49	MYLK
ENSGALG00000002714	18305,731	-1,839377527	0,1231358	-14,93779	1,87E-50	9,10E-48	TMEM132C
ENSGALG00000007963	269,37035	-1,84239576	0,2523089	-7,302143	2,83E-13	1,11E-11	NFATC2
ENSGALG00000014935	24043,958	-1,864724119	0,1640609	-11,36605	6,17E-30	1,04E-27	GREB1L
ENSGALG00000015089	1244,3881	-1,867848339	0,2166645	-8,620924	6,64E-18	4,39E-16	GAP43
ENSGALG00000016906	16521,342	-1,870006943	0,1023605	-18,26883	1,47E-74	2,00E-71	SPRY2
ENSGALG00000012392	124,6248	-1,879813042	0,3053078	-6,157108	7,41E-10	1,79E-08	IYD
ENSGALG00000007786	809,87356	-1,895122662	0,2583339	-7,335942	2,20E-13	8,79E-12	DOK5
ENSGALG00000003437	453,52356	-1,896224168	0,2236049	-8,480244	2,25E-17	1,43E-15	ADAM8
ENSGALG00000009693	162,98636	-1,908196694	0,2849148	-6,69743	2,12E-11	6,38E-10	CPXM2
ENSGALG00000026366	659,49696	-1,956428809	0,2559307	-7,644371	2,10E-14	9,56E-13	TTN
ENSGALG00000016377	39318,721	-1,984049771	0,1516015	-13,08727	3,89E-39	1,20E-36	PXDN
ENSGALG00000007646	1162,4308	-1,999886692	0,2341543	-8,540893	1,33E-17	8,72E-16	
ENSGALG00000014727	707,4954	-2,02826974	0,2257943	-8,982819	2,64E-19	2,02E-17	PDE4D
ENSGALG00000025941	508,27465	-2,032822244	0,2108225	-9,642339	5,30E-22	4,84E-20	RGS20
ENSGALG00000016455	36148,301	-2,034647249	0,1415614	-14,37289	7,66E-47	3,07E-44	GREB1
ENSGALG00000015332	1189,9865	-2,044938557	0,2318737	-8,819191	1,15E-18	8,09E-17	CPLX1
ENSGALG00000001608	473,98158	-2,081100043	0,2297131	-9,059563	1,31E-19	1,03E-17	UNC5D
ENSGALG00000006797	719,73345	-2,093089949	0,1825483	-11,46595	1,96E-30	3,60E-28	ADARB2
ENSGALG00000005347	2547,968	-2,102469906	0,1728132	-12,16614	4,71E-34	1,11E-31	ADAMTS18
ENSGALG00000005061	4294,1757	-2,114741441	0,1575348	-13,42396	4,38E-41	1,53E-38	PPM1E
ENSGALG00000006822	6099,6969	-2,117548167	0,190863	-11,0946	1,33E-28	2,06E-26	PCDH19
ENSGALG00000002540	1690,5022	-2,132167559	0,1444055	-14,76514	2,46E-49	1,08E-46	RGS2
ENSGALG00000014872	706,84998	-2,137267919	0,2117349	-10,09407	5,87E-24	6,77E-22	FGF10
ENSGALG00000005000	10982,035	-2,158647655	0,1512305	-14,27389	3,18E-46	1,24E-43	COMTD1
ENSGALG00000002816	1397,7493	-2,170634895	0,1725497	-12,57976	2,73E-36	7,28E-34	STK10
ENSGALG00000024083	2014,3842	-2,175833689	0,1641192	-13,25765	4,07E-40	1,39E-37	C8orf4
ENSGALG00000017244	1712,4698	-2,189436741	0,1475328	-14,84034	8,03E-50	3,65E-47	PRSS23
ENSGALG00000012106	326,65016	-2,236831516	0,2846604	-7,857895	3,91E-15	1,87E-13	SCTR
ENSGALG00000005689	443,46806	-2,271345118	0,2372034	-9,575515	1,01E-21	9,14E-20	PAX2
ENSGALG00000000600	217,11331	-2,27272638	0,2827556	-8,037777	9,15E-16	4,85E-14	PTPN7
ENSGALG00000019426	136,01564	-2,280006749	0,3003394	-7,591434	3,16E-14	1,41E-12	TMEM71
ENSGALG00000012594	465,97876	-2,283939186	0,2248463	-10,15778	3,06E-24	3,62E-22	NTRK2
ENSGALG00000012732	358,70918	-2,298252682	0,2277106	-10,09287	5,94E-24	6,80E-22	PHACTR1
ENSGALG00000004376	1102,6462	-2,323750078	0,1982175	-11,72323	9,69E-32	2,00E-29	KCNJ2
ENSGALG00000008118	2184,0618	-2,340544818	0,1326409	-17,64572	1,10E-69	1,15E-66	RFTN2
ENSGALG00000010435	1040,6262	-2,340611141	0,2214259	-10,57063	4,08E-26	5,66E-24	RASGRP3
ENSGALG00000007346	9971,3513	-2,361764382	0,1565921	-15,08227	2,12E-51	1,11E-48	KIAA1462
ENSGALG00000017091	828,29835	-2,363903034	0,1707454	-13,84461	1,37E-43	5,05E-41	FLT1
ENSGALG00000012278	11240,166	-2,383840075	0,163892	-14,54519	6,27E-48	2,67E-45	EMCN
ENSGALG00000016785	93,113308	-2,405348378	0,3296339	-7,297029	2,94E-13	1,15E-11	IL1RL1
ENSGALG00000013033	107,44587	-2,410715686	0,3195602	-7,543854	4,56E-14	1,97E-12	CMBL
ENSGALG00000007657	4917,8686	-2,412172788	0,3343808	-7,213849	5,44E-13	2,06E-11	CDX4
ENSGALG00000012927	231,31802	-2,419967236	0,2657891	-9,104839	8,64E-20	6,84E-18	CDH10
ENSGALG00000002183	431,19833	-2,438499895	0,2660795	-9,164552	4,98E-20	4,08E-18	PRR16

ENSGALG00000015947	963,91091	-2,438977585	0,1891685	-12,89315	4,92E-38	1,46E-35	CDH17
ENSGALG00000008893	301,74621	-2,444595373	0,2469193	-9,900382	4,15E-23	4,31E-21	SYT4
ENSGALG00000008890	114,85977	-2,449553325	0,3261648	-7,51017	5,91E-14	2,49E-12	ADGRL4
ENSGALG00000008905	317,21852	-2,456637668	0,2373167	-10,35173	4,11E-25	5,28E-23	HABP2
ENSGALG00000026663	524,42245	-2,478656591	0,2092266	-11,84675	2,24E-32	4,76E-30	
ENSGALG00000012623	104,28468	-2,482707417	0,3217829	-7,715473	1,21E-14	5,58E-13	CFC1B
ENSGALG00000015826	274,53091	-2,484029553	0,3182796	-7,804553	5,97E-15	2,81E-13	HTR1E
ENSGALG00000010224	221,98807	-2,486198557	0,2911733	-8,538554	1,36E-17	8,83E-16	GLIPR1L
ENSGALG00000007940	128,05228	-2,542363859	0,32048	-7,932988	2,14E-15	1,07E-13	RAG2
ENSGALG00000001560	195,37323	-2,555527127	0,3382967	-7,554101	4,22E-14	1,83E-12	KCNU1
ENSGALG00000008581	16731,944	-2,566439645	0,1474527	-17,40517	7,54E-68	5,70E-65	DUSP5
ENSGALG00000016784	1039,8515	-2,607549769	0,1741423	-14,97367	1,09E-50	5,50E-48	IL1RL2
ENSGALG00000006112	988,65304	-2,692857061	0,1899063	-14,17993	1,22E-45	4,61E-43	SCN5A
ENSGALG00000016154	140,89016	-2,742713554	0,306496	-8,948611	3,60E-19	2,71E-17	ARC
ENSGALG00000016145	1265,1012	-2,754137035	0,1745953	-15,7744	4,67E-56	2,76E-53	RIPK4
ENSGALG00000011099	581,91676	-2,760215905	0,269967	-10,22427	1,54E-24	1,88E-22	FAP
ENSGALG00000005209	4151,2431	-2,791291062	0,3487778	-8,003063	1,21E-15	6,28E-14	RP5-877J2.1
ENSGALG00000011562	1031,64	-2,817896047	0,1809122	-15,57604	1,06E-54	6,01E-52	PARK2
ENSGALG00000007656	17151,759	-2,823200316	0,3012681	-9,371057	7,18E-21	6,11E-19	
ENSGALG00000012051	829,36725	-2,878544854	0,2482483	-11,59542	4,35E-31	8,22E-29	STAC
ENSGALG00000028250	102,79679	-2,884651456	0,3308091	-8,719988	2,78E-18	1,88E-16	ANKRD22
ENSGALG00000007701	2491,5718	-2,906313035	0,1654067	-17,57071	4,13E-69	3,75E-66	B3GNT7
ENSGALG00000014984	5902,5896	-2,931476354	0,1495694	-19,59944	1,56E-85	3,04E-82	F2RL1
ENSGALG00000009704	18346,867	-2,934403789	0,1676164	-17,50666	1,27E-68	1,02E-65	CHST15
ENSGALG00000016140	355,67412	-2,998033733	0,2492573	-12,02787	2,54E-33	5,76E-31	FAM3B
ENSGALG00000015358	3246,1808	-3,063209848	0,145761	-21,0153	4,75E-98	1,29E-94	MYH15
ENSGALG00000002562	2027,1289	-3,12939254	0,1764516	-17,73513	2,25E-70	2,55E-67	ARAP3
ENSGALG00000007538	106,89217	-3,275953369	0,3461412	-9,464212	2,96E-21	2,60E-19	IRG1L
ENSGALG00000022962	106,15694	-3,362304357	0,3346563	-10,04704	9,47E-24	1,06E-21	OVST
ENSGALG00000002594	1003,2406	-3,661637709	0,2050909	-17,85373	2,70E-71	3,35E-68	TFPI
ENSGALG00000013624	4838,4306	-3,6799039	0,1462758	-25,15731	1,18E-139	5,34E-136	FAM65B
ENSGALG00000006421	916,75433	-3,742853665	0,2370467	-15,78952	3,67E-56	2,27E-53	SLC16A12
ENSGALG00000020359	246,79437	-3,821393345	0,2845023	-13,43185	3,93E-41	1,41E-38	SIX6
ENSGALG00000003456	187,12467	-3,934712358	0,3247803	-12,115	8,80E-34	2,03E-31	CA12
ENSGALG00000023379	453,27145	-3,949811074	0,2313834	-17,07042	2,46E-65	1,68E-62	F2RL2
ENSGALG00000016431	670,26005	-4,37340061	0,2358196	-18,54553	8,86E-77	1,34E-73	PAX5
ENSGALG00000014994	310,70765	-5,043766073	0,2868267	-17,58472	3,23E-69	3,14E-66	CRHBP
ENSGALG00000016783	9121,1085	-5,301446621	0,1450351	-36,55285	1,61E-292	2,19E-288	IL1R1
ENSGALG00000004221	898,01485	-5,338632777	0,2699166	-19,77882	4,53E-87	1,03E-83	IL22RA1
