# Supplementary material for: HOXB8 Counteracts MAPK/ERK Oncogenic Signaling in a Chicken Embryo Model of Neoplasia
Source: Int J Mol Sci. 2021 Aug 18;22(16):8911. doi: 10.3390/ijms22168911 (PMC8396257; doi:10.3390/ijms22168911)
Supplement: Supplementary file 1 [file ijms-22-08911-s001.zip › ijms-1277141 Table S5.pdf]

Table S5: Classification of the 2316 genes deregulated in the "MEK1ca versus pCIG" condition (False Discovery Rate (padj) <= 0.05) clustered using "kmeans" with k=12, with the mean TPM (transcripts per kilobase million) of each gene for the two replicates of pCIG, HOXB8, MEK1ca, and MEK1ca+HOXB8 in each condition.

ID	Symbol	pCIG	HOXB8	MEK1ca	MEK1_HOXB8	Cluster
ENSGALG00000000141	BLB1	4,346461397	6,936083838	25,83463981	9,813152958	1
ENSGALG00000000307		59,23843877	89,71588529	143,6917892	78,57624532	1
ENSGALG00000000600		0,16716819	0,219662917	4,032909459	0,585105026	1
ENSGALG00000000639	LRRN2	4,627904717	4,790564693	9,521707125	5,306668428	1
ENSGALG00000000761	TSKU	53,35356817	54,75872543	78,02304089	50,7231382	1
ENSGALG00000000795	MFSD12	15,27116365	19,51741298	24,13081695	17,43156709	1
ENSGALG00000000977	MAPKAP1	76,21721102	75,42044153	139,8918526	69,33283402	1
ENSGALG00000001094	ADGRD2	102,7073676	111,4654329	121,4385963	94,7567043	1
ENSGALG00000001220	HERPUD1	52,87970256	43,77611515	97,02489846	55,98900064	1
ENSGALG00000001346		12,45034341	13,79821487	25,17364864	8,821835481	1
ENSGALG00000001370	ADAMTS8	10,00645846	11,44367995	17,37313634	8,098120229	1
ENSGALG00000001383	HTR4	0,223346685	0,462240728	2,654657367	0,480455141	1
ENSGALG00000001405	IRF6	2,701639368	2,9520233	6,01110282	3,704203216	1
ENSGALG00000001419	DAB2IP	8,238485904	12,58819262	17,26179821	10,32238732	1
ENSGALG00000001560		0,150607399	0,295573757	2,997594118	0,175011822	1
ENSGALG00000001596	MLLT1	104,5141917	111,7769614	144,5899475	114,1039573	1
ENSGALG00000001621	CLDN5	1,779507208	3,559103071	26,91827318	8,738372257	1
ENSGALG00000002183	PRR16	1,802939556	2,415784551	21,12574473	2,851415468	1
ENSGALG00000002242		3,30906785	4,319855305	5,828198288	3,158054392	1
ENSGALG00000002509		30,0871481	32,18952881	61,1232203	26,93140662	1
ENSGALG00000002532	GJA4	0,544399491	0,638100325	1,969673798	0,784806618	1
ENSGALG00000002540		6,446932133	5,308050588	40,08364447	8,825438475	1
ENSGALG00000002543	CSGALNACT2	32,12663012	34,93337178	76,29195476	43,26033436	1
ENSGALG00000002555	RET	2,88163933	4,228435821	7,895631031	4,06619279	1
ENSGALG00000002562	ARAP3	1,48713749	1,131854243	19,88581577	2,017191883	1
ENSGALG00000002594	TFPI	1,818415284	2,178414033	30,95014095	1,96144887	1
ENSGALG00000002598	TPST1	110,6957744	124,8080538	194,3906783	111,9974286	1
ENSGALG00000002623	PCOLCE2	6,900390576	7,625754792	13,68975598	9,209524817	1
ENSGALG00000002632	CALCRL	12,01510153	10,78717477	32,20239461	13,53387588	1
ENSGALG00000002646	FAM171B	70,39669635	76,78219835	117,3361401	83,48810086	1
ENSGALG00000002655	ITGAV	34,70042214	37,88033757	56,07206538	41,54256787	1
ENSGALG00000002704		1,560758451	1,645911429	3,272911068	1,63570101	1
ENSGALG00000002763	FRZB	88,08590473	101,8551818	118,1762783	90,87514056	1
ENSGALG00000002816	STK10	1,302638213	1,070607568	9,306934994	1,934117709	1
ENSGALG00000002830	NUDT3	82,47138192	83,39720868	119,0949223	90,87056175	1
ENSGALG00000002863	ERGIC1	200,1815036	199,8499674	251,621888	188,1263931	1
ENSGALG00000002954	MBTD1	61,91506849	61,41366817	80,11960497	59,8379799	1
ENSGALG00000002993	CA10	4,907630575	7,330232074	13,05830949	4,830057404	1
ENSGALG00000003114	NOG	8,459709423	11,93287775	23,8221868	8,350925143	1
ENSGALG00000003149		3,188704926	3,828601125	6,728823346	4,067555236	1
ENSGALG00000003323	OSGIN1	0,150652789	0,306431517	2,76792484	0,600147405	1
ENSGALG00000003339	MTOR	32,72614893	33,27392871	40,05816094	32,03402147	1
ENSGALG00000003353	RAI14	72,22040829	61,3924759	156,0160059	63,07779452	1
ENSGALG00000003437		1,140967301	0,975600437	7,404177579	1,755276738	1
ENSGALG00000003456	CA12	0,043477625	0,052506397	4,443271062	0,056186982	1
ENSGALG00000003668		0,107991316	0,101421289	0,499834388	0,201779775	1
ENSGALG00000003706		7,669767671	8,241795488	28,6640262	10,83001181	1
ENSGALG00000004155	MYOM3	0,565715899	0,738462272	1,297153697	0,505525313	1
ENSGALG00000004221		0,270203292	0,285792101	18,79414137	0,190172822	1
ENSGALG00000004273	RALGPS2	68,26603769	74,39473921	93,44497048	67,30976616	1
ENSGALG00000004306	SYT12	2,605589645	4,144014971	15,45617568	4,186305684	1
ENSGALG00000004376	KCNJ2	3,77289362	4,959356504	22,31025244	3,944582	1
ENSGALG00000004393		4,430270701	6,514512501	10,5028138	5,156954248	1
ENSGALG00000004531	PLOD1	39,43375984	39,16887142	53,49951129	39,85679447	1
ENSGALG00000004569	UNC5B	7,868192231	10,03498033	22,37566356	12,64304576	1
ENSGALG00000004612	MTHFR	29,88510462	33,82285398	48,61122399	33,33543084	1
ENSGALG00000004624		38,51562207	38,74443918	46,92510617	37,97246389	1
ENSGALG00000004638		12,39324598	12,9301946	17,06424419	12,4188215	1

ENSGALG00000004696	LEO1	51,73562652	54,64381268	67,49391493	53,30887079	1
ENSGALG00000004879	SLC6A11	6,044456134	7,986360168	9,907299162	6,80721088	1
ENSGALG00000004930	HRH1	2,252570121	1,790119278	7,87682517	1,349179431	1
ENSGALG00000005000	COMTD1	48,07598474	44,35755518	397,3367537	85,33221268	1
ENSGALG00000005061	PPM1E	15,5883157	12,4158043	76,17698494	16,84323915	1
ENSGALG00000005134	ZSWIM8	11,30147215	11,70309029	15,86549961	11,59078122	1
ENSGALG00000005160	VMP1	97,80171612	94,91460927	126,6233829	94,36577445	1
ENSGALG00000005209		0,978757944	1,181001848	116,1518425	3,250210874	1
ENSGALG00000005347	ADAMTS18	7,626809484	5,838020535	25,8863696	5,61477326	1
ENSGALG00000005430	ALS2CL	1,955531218	2,01749045	3,820475596	2,280218129	1
ENSGALG00000005442	PALMD	1,233769732	2,376997769	5,258268724	1,702027405	1
ENSGALG00000005468	SYNRG	31,68189573	33,65304843	40,16103869	31,72045473	1
ENSGALG00000005476	PTH1R	0,874654422	0,830470831	5,138549733	0,976037129	1
ENSGALG00000005498		6,346864963	6,410134604	28,72716752	8,222793763	1
ENSGALG00000005499	IL17RD	42,29096857	36,47768079	270,3943487	91,41665589	1
ENSGALG00000005519	TLL2	1,410283264	2,247551296	22,60288502	3,669028574	1
ENSGALG00000005610	SLC44A3	5,452687132	6,470508823	10,61156478	5,917968895	1
ENSGALG00000005619		12,91699497	13,14521332	36,29311148	14,78644626	1
ENSGALG00000005785		6,273844084	6,527878689	9,293607737	4,778628942	1
ENSGALG00000005805	PLCD1	8,133157801	12,3694805	21,47493828	11,05897104	1
ENSGALG00000005855		17,59991732	15,64179009	118,2991947	40,94877771	1
ENSGALG00000006002	WHAMM	15,4050577	16,68613201	22,65836529	17,34219926	1
ENSGALG00000006072	SCN10A	0,071733051	0,072511186	0,322233693	0,040260369	1
ENSGALG00000006084		0,415711399	1,339960031	3,460295102	0,856058604	1
ENSGALG00000006112	SCN5A	0,916499676	0,661017755	4,51117604	0,605407018	1
ENSGALG00000006121	PIK3C2A	42,74947084	49,54970032	60,78554868	45,17039169	1
ENSGALG00000006337		0,002439521	0,023624003	0,312529764	0,041882001	1
ENSGALG00000006376	TMC3	0,192169524	0,233676578	1,09689024	0,353408492	1
ENSGALG00000006401	OSBPL5	11,36431471	10,26220066	31,66422311	12,85811602	1
ENSGALG00000006421	SLC16A12	0,786470857	0,789157454	28,31054622	1,468096746	1
ENSGALG00000006609	EPHB1	70,32545848	78,56087564	89,8486204	58,97301751	1
ENSGALG00000006652	DIP2C	13,37555911	12,67551415	17,61300034	13,378876	1
ENSGALG00000006808	TSPAN6	84,8160072	92,4891866	182,1056518	96,16407149	1
ENSGALG00000006975		35,48790893	37,00507441	45,63493758	29,37760443	1
ENSGALG00000006995	APOD	0,696051145	1,390188703	7,733888215	1,572610716	1
ENSGALG00000006997	BRINP1	3,499835162	4,356493158	11,27517561	3,348538768	1
ENSGALG00000007039		40,28481666	45,9836234	79,80599717	45,70745825	1
ENSGALG00000007069		1,998973396	3,269505285	4,779320231	2,74022749	1
ENSGALG00000007113		4,572530679	5,629890394	15,02562976	4,089934281	1
ENSGALG00000007171	CIITA	0,022403266	0,204546083	2,706469378	0,728094044	1
ENSGALG00000007206		0	0,022465315	0,940527994	0,230962067	1
ENSGALG00000007218	MB21D2	21,50353374	22,18131924	63,39535546	18,17572811	1
ENSGALG00000007242	SLITRK4	11,61340101	13,68905429	17,8729469	10,94148732	1
ENSGALG00000007268		12,02218487	17,33922043	29,52538581	13,1036655	1
ENSGALG00000007346	KIAA1462	9,556019949	13,87679845	75,69402273	13,92754889	1
ENSGALG00000007501		6,923403837	6,775032581	12,8684032	7,692808633	1
ENSGALG00000007538		0	0,011572698	2,680467049	0,023686378	1
ENSGALG00000007646		1,828426892	2,407614248	12,04323697	2,574591371	1
ENSGALG00000007656		9,354467191	12,73350311	306,4981787	24,13865507	1
ENSGALG00000007657	CDX4	10,43751312	5,656329149	135,1902583	10,65993568	1
ENSGALG00000007660		18,53508552	20,01450682	57,59481836	23,79729358	1
ENSGALG00000007667	GPR158	2,017006699	2,351089848	4,019170374	2,390691361	1
ENSGALG00000007701	B3GNT7	1,965758713	2,885006525	58,70594437	7,123195196	1
ENSGALG00000007771	FAM210B	48,42301262	61,59675671	141,9653945	54,87237005	1
ENSGALG00000007786	DOK5	1,639190441	2,831374722	12,89140399	2,824710311	1
ENSGALG00000007940		0,298085716	0,405740152	3,03794633	0,245682446	1
ENSGALG00000007952	AMOT	42,59469668	43,129628	81,92458191	43,27930325	1
ENSGALG00000007963	NFATC2	0,197351415	0,379445389	3,998563902	0,912021662	1
ENSGALG00000008118		11,21822805	8,858651443	37,96056253	7,261176994	1
ENSGALG00000008195	NPHP1	52,32037043	55,15367663	70,06251176	55,17279702	1
ENSGALG00000008293	SH3PXD2A	4,963643686	5,106021399	7,660740968	4,819388759	1
ENSGALG00000008318	SLK	85,01647415	80,15010998	134,3520566	81,18806127	1

ENSGALG00000008353		4,025184036	3,680815167	16,66369921	4,874571991	1
ENSGALG00000008469	SPINT1	14,24665038	16,90480956	32,8484745	18,45751953	1
ENSGALG00000008542	ADD3	118,4151596	124,28577	157,6607813	123,3415266	1
ENSGALG00000008581		10,16528829	6,061157018	256,373084	41,13464142	1
ENSGALG00000008621	NRP2	78,31928008	89,14932983	118,8571698	70,77451755	1
ENSGALG00000008739	FAM188A	46,04693561	50,96110843	56,92147735	48,6759362	1
ENSGALG00000008875	PLCB1	4,345271238	4,743918313	14,04603032	6,225376121	1
ENSGALG00000008890	ADGRL4	0,129602088	0,092978733	0,996601503	0,083175824	1
ENSGALG00000008905	HABP2	0,266437294	0,48347075	6,734457842	0,981954515	1
ENSGALG00000009170	NCEH1	26,8945161	27,63392523	60,16087129	19,95188217	1
ENSGALG00000009179	TNFSF10	50,54952664	51,97607136	96,43379558	44,37200827	1
ENSGALG00000009222	FNDC3B	20,05392267	20,78387393	24,33913641	20,24031624	1
ENSGALG00000009252		17,05340609	20,22552019	30,26838438	17,79052756	1
ENSGALG00000009282	ELMSAN1	12,79207245	10,87027045	43,26764769	12,23866375	1
ENSGALG00000009300	NUMB	37,95931361	39,65737919	52,66309106	40,50552035	1
ENSGALG00000009360	CAPN2	3,497797582	4,055116565	9,127908666	4,85175942	1
ENSGALG00000009415	SMOC1	117,4087959	134,7914352	180,6575257	94,49588202	1
ENSGALG00000009450	DUSP10	9,354175589	11,43364627	21,919628	10,97128745	1
ENSGALG00000009562		71,61834554	77,27651814	93,73781016	58,03501792	1
ENSGALG00000009567	TLL1	12,75490022	14,9239491	30,19675472	13,74189903	1
ENSGALG00000009667	MFAP3L	4,900753944	6,755229067	18,92590259	7,235188798	1
ENSGALG00000009693	CPXM2	0,297057574	0,224259756	1,916995035	0,375685436	1
ENSGALG00000009704	CHST15	26,42019968	31,60061585	300,8079085	35,72264945	1
ENSGALG00000009740	RASGRP1	2,238442875	3,221407126	8,304577968	3,876544109	1
ENSGALG00000009752	C1GALT1	73,87944835	76,00031211	88,11179224	77,6483376	1
ENSGALG00000009850	NOVA1	65,62071803	72,5812928	91,8483665	60,49260852	1
ENSGALG00000009855	TBC1D9	35,32107704	35,40997725	52,56579067	36,25236136	1
ENSGALG00000009920	COCH	13,26044931	11,28208934	38,09312731	9,926593491	1
ENSGALG00000009947	PLEKHH2	8,548346442	7,655850005	20,90561351	8,219735522	1
ENSGALG00000010010	RHOQ	22,36249765	32,86035501	57,1305441	31,25736751	1
ENSGALG00000010061	LRBA	13,57799133	15,84454033	19,19400383	15,01651499	1
ENSGALG00000010148		0,376485353	0,303044356	1,090711174	0,276420272	1
ENSGALG00000010156		0,484158088	0,407906519	1,056535994	0,478788157	1
ENSGALG00000010163	LGR5	29,22401237	42,84673911	58,12291645	29,33576971	1
ENSGALG00000010204	KCNC2	8,315391818	9,598554626	14,43598051	9,193014079	1
ENSGALG00000010224		0,29053295	0,779126106	7,883361459	0,88877587	1
ENSGALG00000010258	LTBP2	1,700141471	1,689599283	7,343564576	2,281390244	1
ENSGALG00000010282	TMEM63B	53,53998682	52,35379756	67,85386895	55,3585158	1
ENSGALG00000010318	TMED10	380,0260003	378,4420008	456,2386739	383,0242366	1
ENSGALG00000010412	WWTR1	7,372640458	8,212018771	15,65426723	9,207581713	1
ENSGALG00000010435	RASGRP3	0,951722698	1,288332265	16,04572445	2,667849388	1
ENSGALG00000010468	ISM2	6,961603369	5,451403991	28,88288749	9,016585179	1
ENSGALG00000010522		52,19125685	45,4437284	88,95246599	53,90163476	1
ENSGALG00000010642	IRF2	14,12465929	15,27261891	20,26270283	12,94760591	1
ENSGALG00000010683		1,286887376	2,082891848	4,318730895	2,217138302	1
ENSGALG00000010688	SHC3	0,377086245	0,473231752	1,28457644	0,656462583	1
ENSGALG00000010692	LRP8	17,33417611	14,1459726	39,14255891	19,74069674	1
ENSGALG00000010707	GPR68	0	0,159372632	1,201854665	0,204226743	1
ENSGALG00000010785	ACOT11	3,3120391	3,449588553	7,445190708	4,169283534	1
ENSGALG00000010926		1,182613457	1,250155615	4,861891125	1,667707956	1
ENSGALG00000011099	FAP	0,524872973	0,627940647	8,568804109	0,852104076	1
ENSGALG00000011110		1,049280017	1,070578153	3,569578228	1,155450113	1
ENSGALG00000011120		0,903887543	1,717288396	2,968494683	1,501450921	1
ENSGALG00000011148		10,04557421	13,49083348	31,35368024	9,031119595	1
ENSGALG00000011172		7,443800272	6,657256083	14,36492915	6,634505832	1
ENSGALG00000011207	DUSP6	38,54581524	33,84315846	198,3618888	73,6726962	1
ENSGALG00000011236	BEGAIN	0,363396332	1,174831529	2,353729205	0,884245626	1
ENSGALG00000011246		34,4951174	37,48548023	47,02695891	34,71936867	1
ENSGALG00000011258		115,2808442	105,6819299	225,5885638	117,6950123	1
ENSGALG00000011336		0,100777952	0,154515943	0,745110987	0,176260961	1
ENSGALG00000011376	ANKRD9	5,699927441	6,47582585	10,76381249	5,70463788	1
ENSGALG00000011439		11,48455465	11,97057423	28,13859794	12,9402475	1

ENSGALG00000011468	IGFBP5	22,18372906	31,8043152	67,63240972	24,25782653	1
ENSGALG00000011548	PPP1R13B	17,59096224	18,42868364	27,31810324	20,71203786	1
ENSGALG00000011562	PARK2	2,307166008	2,209921812	14,10410652	1,800517372	1
ENSGALG00000011581	KIF26A	4,38085147	8,865650561	49,95121099	16,48311585	1
ENSGALG00000011609		9,843500616	8,417625726	40,27301602	8,233732841	1
ENSGALG00000011618	ADSSL1	8,786398246	10,75524319	18,1647197	11,87110001	1
ENSGALG00000011623	ADAMTS3	24,331848	27,29215191	78,31157876	35,78867135	1
ENSGALG00000011707		53,24117172	52,60615779	73,60572574	52,46461184	1
ENSGALG00000011762	UBA6	45,15145647	45,49374679	54,61323703	46,15736387	1
ENSGALG00000011770	UMPS	129,7844633	135,257901	207,8820378	147,8994428	1
ENSGALG00000011867	SERHL2	29,43272669	33,34712098	71,24407539	29,95176995	1
ENSGALG00000011940		2,58677773	5,2650269	34,93790074	11,23222133	1
ENSGALG00000012018	UGT8	3,339674913	5,868867271	29,87840982	10,482522	1
ENSGALG00000012051		4,041058882	2,27262952	31,35683641	3,147836465	1
ENSGALG00000012106	SCTR	0,647796476	1,062662762	4,974582478	0,728529092	1
ENSGALG00000012439		5,987859862	8,057194256	15,71567866	7,213637245	1
ENSGALG00000012454	CYTH4	0,040933627	0,148295635	0,646186586	0,122442754	1
ENSGALG00000012522	PVALB	0,10415029	0,018320357	1,845669062	0,264205542	1
ENSGALG00000012533		82,14235353	89,9220224	161,7487773	106,8315371	1
ENSGALG00000012608	DAPK1	18,63601716	17,25242431	24,89907156	19,04902754	1
ENSGALG00000012623		0,308723784	0,17685494	2,783031501	0,236507182	1
ENSGALG00000012635		2,956578448	4,062704068	12,89063246	3,64967825	1
ENSGALG00000012732	PHACTR1	1,002723787	0,92758825	5,310014441	0,874633616	1
ENSGALG00000012808	CDYL	18,02096582	21,34276206	37,17353321	21,75666775	1
ENSGALG00000012848		20,64815843	19,22874436	38,64533737	23,70889337	1
ENSGALG00000012849	SLC37A3	25,5841736	29,25564121	46,57867282	31,70186837	1
ENSGALG00000012911	SYT10	1,659882445	2,809814019	5,964646057	2,833486186	1
ENSGALG00000012979		22,57549197	22,92713118	34,79249726	24,59961237	1
ENSGALG00000012998	WNT5B	32,51568668	42,44877134	79,13605981	43,44051816	1
ENSGALG00000013033		0,623034671	0,523981207	6,611512329	0,624842166	1
ENSGALG00000013102	COBL	1,89987265	2,53428906	12,26309687	3,628837952	1
ENSGALG00000013149	MOCOS	0,31264351	0,69552403	1,148089973	0,526605797	1
ENSGALG00000013406	EOGT	8,979738398	9,102324177	13,64010251	10,0250873	1
ENSGALG00000013548	GZMA	0,244325018	0,143724945	1,250994806	0,095859771	1
ENSGALG00000013624		2,042343298	4,425049521	70,07747071	5,032989524	1
ENSGALG00000013661	MICU3	16,86990908	18,35601379	24,39035699	18,72105289	1
ENSGALG00000013683	ARID1B	50,21288267	47,56895592	91,55652092	53,76612448	1
ENSGALG00000013892	MAP3K5	3,106959802	3,891469812	7,477861463	3,385152426	1
ENSGALG00000014159	PACSIN2	46,93642045	52,69096243	231,699989	66,05762406	1
ENSGALG00000014165		34,4949156	36,21634446	46,38287449	35,66651524	1
ENSGALG00000014213	PRR5	45,61960191	49,63409703	60,32943821	40,69527126	1
ENSGALG00000014337	NWD2	4,063259347	3,87414171	6,001585157	3,902325985	1
ENSGALG00000014370		25,6946956	26,45741685	32,9477035	27,52616136	1
ENSGALG00000014688	ZYX	73,08300226	79,10890291	102,6158311	80,01024875	1
ENSGALG00000014783		22,3498316	24,871629	42,37641705	29,08458804	1
ENSGALG00000014823		0	0,035377845	0,977269445	0,046829328	1
ENSGALG00000014852		58,83953299	69,54541567	178,8283916	76,07766834	1
ENSGALG00000014855		27,82040525	38,69420984	56,11193291	36,63572912	1
ENSGALG00000014860	YES1	130,3824873	130,6854913	192,983834	128,1750395	1
ENSGALG00000014864		3,731602592	5,046367263	9,359766476	4,111091192	1
ENSGALG00000014866		1161,822033	1014,002441	2650,648004	1063,272464	1
ENSGALG00000014872	FGF10	1,077530612	0,935843353	31,13159693	6,221341059	1
ENSGALG00000014877	EMB	13,70340987	18,5752455	42,8108232	19,61514892	1
ENSGALG00000014907	DCBLD1	14,02744527	19,4714576	31,49340589	18,13536669	1
ENSGALG00000014930	ENC1	32,6260464	23,96935622	95,15822903	28,5198255	1
ENSGALG00000014953	RWDD1	34,3211322	35,70428677	48,85056151	36,89366799	1
ENSGALG00000014957	WDR1	149,3387297	141,564327	241,426839	140,1462197	1
ENSGALG00000014961		0,030063775	0,212281951	0,53262582	0,159553166	1
ENSGALG00000014983	F2R	11,99732554	10,51644411	30,61968372	11,21982229	1
ENSGALG00000014984	F2RL1	9,191228695	7,297000019	60,18915078	7,334530663	1
ENSGALG00000014994	CRHBP	0,094206422	0,123018797	8,155312142	0,069432825	1
ENSGALG00000015008	ZNF366	0,025612818	0,007984965	0,218726746	0,02805032	1

ENSGALG00000015028		5,215749923	6,301235219	16,40492276	7,825223421	1
ENSGALG00000015033	REV3L	14,90469289	16,2960558	20,42678903	15,73038983	1
ENSGALG00000015089	GAP43	10,73590963	9,303884931	33,04526973	8,030676006	1
ENSGALG00000015208	ROR2	17,52597629	20,97201143	36,98791655	21,6549491	1
ENSGALG00000015252	ST3GAL6	50,15692337	52,54417622	62,67006181	44,72893	1
ENSGALG00000015256	PAM	37,6976856	29,97280604	96,16475159	36,54825142	1
ENSGALG00000015332	CPLX1	1,265996124	4,716193333	92,44153266	18,97429343	1
ENSGALG00000015358	MYH15	2,502155814	2,562264429	24,52524418	2,753567038	1
ENSGALG00000015388		0,998528448	2,80672025	22,77458166	7,945924483	1
ENSGALG00000015439	SLC44A1	37,1490751	42,22953839	47,91821194	36,92039081	1
ENSGALG00000015440		0,649690061	0,632601394	1,775464964	0,785617529	1
ENSGALG00000015458	ILDR2	95,9535283	99,09522051	139,284134	104,0358316	1
ENSGALG00000015624	VCAN	33,86551135	34,75318733	94,61693612	47,34830969	1
ENSGALG00000015634		0,190327277	0,320177513	1,214767371	0,43293815	1
ENSGALG00000015665	SH3BP2	3,286250973	2,895344261	9,524027287	4,083976177	1
ENSGALG00000015826	HTR1E	0,33449219	0,44224444	11,45369572	1,027953514	1
ENSGALG00000015896		6,388186822	7,197999605	12,51939522	6,10029628	1
ENSGALG00000015947		0,048261531	0,051324674	8,627025826	1,412173952	1
ENSGALG00000015975		46,39913828	46,69943177	77,97746804	37,87795667	1
ENSGALG00000016074		2,965663767	3,518415951	4,36951983	3,22716103	1
ENSGALG00000016102		1,474592182	2,407557081	8,858325248	2,452322765	1
ENSGALG00000016140	FAM3B	1,345511674	1,561374658	14,90793732	1,339776747	1
ENSGALG00000016145		0,594265981	1,380102509	16,360371	2,19880009	1
ENSGALG00000016226		82,42631993	87,8153227	97,22152217	82,97244346	1
ENSGALG00000016236		79,83962807	81,6981472	94,32375099	74,79813666	1
ENSGALG00000016297	HCRTR2	0,172470075	0,189519273	0,91709899	0,109463532	1
ENSGALG00000016377	PXDN	48,44717284	47,31975564	221,1364094	53,85481487	1
ENSGALG00000016431	Pax5B	0,759670645	1,184984006	24,88772514	0,76701086	1
ENSGALG00000016470	KCNS3	0,820548787	1,835471231	21,82469461	5,812307253	1
ENSGALG00000016537	SCML2	23,96103489	29,8195471	50,09057341	29,94459128	1
ENSGALG00000016602		3,664177334	3,917626255	12,58470849	4,88155337	1
ENSGALG00000016630	EXTL3	78,945634	78,16521266	100,6801361	82,53544774	1
ENSGALG00000016736	CYFIP1	115,4849451	117,5058947	151,3780148	125,0449365	1
ENSGALG00000016751		74,54427214	79,14506937	102,3489998	80,62212588	1
ENSGALG00000016754		6,519633751	9,835468627	20,77499853	9,953787953	1
ENSGALG00000016768	LONRF2	44,67796838	42,46064416	63,91927301	44,69491354	1
ENSGALG00000016781	MAP4K4	80,99823221	79,95663486	120,0802219	86,66172865	1
ENSGALG00000016783		0,807775429	0,850085852	114,9806394	2,530944487	1
ENSGALG00000016784		0,558045454	0,472553521	7,610637786	1,133090803	1
ENSGALG00000016785	IL1RL1	0,070474547	0	1,971779618	0,162809382	1
ENSGALG00000016834	MCF2L	4,225420748	4,647949468	6,579407696	4,100185155	1
ENSGALG00000016835		19,91459988	25,58593002	33,27889918	23,30715898	1
ENSGALG00000016883	DOCK9	10,45434421	10,83646735	15,52539363	10,77548298	1
ENSGALG00000016893		30,0481437	30,75098399	36,9917239	30,50750363	1
ENSGALG00000016899	DCT	0,082019435	0,067986713	0,424223435	0,074974082	1
ENSGALG00000016906	SPRY2	22,79491815	23,80423911	369,1836197	101,4167028	1
ENSGALG00000016911	RNF219	35,29870957	34,99094267	44,02485713	33,4703359	1
ENSGALG00000016998	LPAR6	5,959033195	5,246978391	17,44700786	7,250724714	1
ENSGALG00000017044	TRPC4	0,533180653	0,692407894	1,395530609	0,748381254	1
ENSGALG00000017091	FLT1	1,390041035	0,693832705	8,294413787	1,492735256	1
ENSGALG00000017135	CRYL1	4,9261885	4,709864357	10,17588307	4,813391563	1
ENSGALG00000017179	PDGFD	2,173029014	3,505795831	14,26690549	3,894835223	1
ENSGALG00000017186		69,04285483	68,94924482	88,45648678	73,22242114	1
ENSGALG00000017242	FZD4	4,01523892	5,003599185	20,09846171	6,618181415	1
ENSGALG00000017244	PRSS23	12,28501908	11,45283797	64,02403712	13,46351005	1
ENSGALG00000017714		2,458678092	3,270301188	9,373616504	4,3477783	1
ENSGALG00000018479		19,30607593	27,19022439	34,32889124	17,26565929	1
ENSGALG00000019261		6,423634206	5,898459466	17,86083615	5,032292518	1
ENSGALG00000019397	CERK	65,45684989	75,63715367	102,1461158	52,57771362	1
ENSGALG00000019426		1,895961953	1,778982812	8,767179842	1,135546894	1
ENSGALG00000019552	SERPINB2	0,03276452	0,012366785	0,305019782	0,020558652	1
ENSGALG00000019716	KRT20	0,427331711	0,54228415	2,142286477	0,421447442	1

ENSGALG00000020057	PIGF	47,49119487	53,61856839	63,6504531	43,06229201	1
ENSGALG00000020359	SIX6	0,147864328	0,28328832	7,275442564	0,245244412	1
ENSGALG00000020723		23,95654484	27,95652952	51,89848439	27,20320324	1
ENSGALG00000021238		3,377343555	3,609177279	6,58959715	4,12451232	1
ENSGALG00000021879		0,060680129	0,02783627	4,532687997	0,361707989	1
ENSGALG00000022871		9,149877167	11,59069382	20,94301968	8,951752944	1
ENSGALG00000022962		0,005547593	0	1,110790954	0,021210214	1
ENSGALG00000023355	TMEM154	0,274198999	0,242777653	1,595469388	0,503883204	1
ENSGALG00000023379	F2RL2	0,699357483	0,206509379	17,20987384	0,770639649	1
ENSGALG00000023406		7,189218224	27,88560585	237,0573377	66,09046748	1
ENSGALG00000023430		0,204871896	0,107896168	5,766235294	0,103839874	1
ENSGALG00000023510	ERP29	244,8276365	243,7579874	286,6547438	250,6109926	1
ENSGALG00000023521	ADRA2A	2,990826686	6,4207327	33,46692465	9,885743021	1
ENSGALG00000023581		2,293508573	3,632286312	6,833722653	3,344298943	1
ENSGALG00000024083	C8orf4	24,35008397	20,56522548	90,61964264	18,98861546	1
ENSGALG00000025723		0,558060232	0,679196686	0,810932422	0,573634828	1
ENSGALG00000025773		12,25494928	11,44694026	30,15620585	9,759339323	1
ENSGALG00000025941	RGS20	0,992958806	1,120198002	4,961879868	1,076166451	1
ENSGALG00000026262		23,02651413	19,44179408	89,23902687	31,06609278	1
ENSGALG00000026366		0,032450796	0,03560783	0,297910888	0,062257621	1
ENSGALG00000026518	RUNDC3A	10,57114969	16,46754486	25,12660637	14,66077777	1
ENSGALG00000026557	PGF	1,365378945	1,285576654	6,812213801	1,989316648	1
ENSGALG00000026582		6,114977984	5,267516926	12,31174473	6,045225829	1
ENSGALG00000026663	CX3CL1	0,28585624	0,517672331	5,224016511	0,810407917	1
ENSGALG00000026753		4,373338809	4,733385198	6,354129053	4,718191959	1
ENSGALG00000026763		5,105466174	4,675186082	12,43728303	5,145242205	1
ENSGALG00000026790	DMRT2	2,44415194	3,013071402	9,651534911	4,741219823	1
ENSGALG00000026892		0,630709919	0,49426779	2,669713204	0,343635403	1
ENSGALG00000026970		19,9958411	29,56495965	47,72673087	27,76473883	1
ENSGALG00000026995	RHOG	2,125612682	1,771546724	5,966320997	2,254397519	1
ENSGALG00000027133		0,276818664	0,334539577	1,403416102	0,364180584	1
ENSGALG00000027210		0,626776305	0,767218066	2,251672836	0,943311678	1
ENSGALG00000027497		4,239074936	4,349860411	10,12003694	4,862962086	1
ENSGALG00000027864	NFKBIA	5,145580181	5,317887462	7,594774216	5,76461762	1
ENSGALG00000027885	C4orf19	0,006715072	0,052136259	0,371881758	0,049775385	1
ENSGALG00000028046	INSIG2	47,56660897	56,64100654	107,3362206	50,7535001	1
ENSGALG00000028047	RHOV	2,271837176	2,028232459	7,468783549	2,647132976	1
ENSGALG00000028250	ANKRD22	0,112085424	0,162263291	0,899150004	0,041047018	1
ENSGALG00000028267		76,90546701	75,83970622	103,3050563	76,29588931	1
ENSGALG00000028291		96,11071203	93,78119127	144,7372696	92,58940493	1
ENSGALG00000028294	VWA5B2	14,51886882	15,87519135	30,51008951	18,72172895	1
ENSGALG00000028637		37,10791538	39,94856328	50,36322451	41,0166067	1
ENSGALG00000028708		24,99318737	36,73989626	54,90494637	28,8846325	1
ENSGALG00000028938		0,513837926	0,624776649	0,839919222	0,435033808	1
ENSGALG00000029175	EFNB2	30,56530828	27,21275717	113,8041773	33,97932717	1
ENSGALG00000000195	SVCT2	19,10462588	17,48116214	25,91142744	17,63462036	2
ENSGALG00000000208	MCC	25,83310361	20,09476945	33,82276296	23,91037382	2
ENSGALG00000000258		75,85689269	54,92413434	95,52174596	62,32390544	2
ENSGALG00000000275	KCTD9	37,75610815	32,89373192	45,75008795	35,24739442	2
ENSGALG00000000332		72,56124041	58,37784284	85,08368566	66,53036384	2
ENSGALG00000000346		75,70929419	72,18519081	95,30895247	71,18874501	2
ENSGALG00000000388		173,5309683	83,88403469	265,5077681	88,686351	2
ENSGALG00000000680		65,55002426	35,12675538	98,37170682	37,34008803	2
ENSGALG00000000936		18,6039239	13,94226071	27,03075663	11,68456073	2
ENSGALG00000001000		903,1481891	710,3094654	1580,638121	839,8439333	2
ENSGALG00000001003	SEPN1	54,28402873	50,00244607	70,19927743	47,23413232	2
ENSGALG00000001169	CRB2	129,1232018	81,31040842	173,364492	61,90998035	2
ENSGALG00000001192		56,80326116	47,24213542	66,68178678	43,96666792	2
ENSGALG00000001276	HOXB9	440,9241521	286,4552462	833,8511125	381,4973378	2
ENSGALG00000001363	MYBBP1A	108,2631392	74,57137633	129,5761289	87,68932285	2
ENSGALG00000001608	UNC5D	1,806421005	1,336857976	5,395223323	1,082125149	2
ENSGALG00000001645	KCNT1	3,342845418	2,419654306	6,945138399	2,085754068	2

ENSGALG00000001656		187,6401815	129,4403167	226,396159	121,0670172	2
ENSGALG00000001749	ACSBG2	25,77749834	18,04184176	33,32440771	18,2584482	2
ENSGALG00000001768	TENM2	13,34961314	7,569539699	19,32663023	7,163569584	2
ENSGALG00000001957		30,17853095	28,11283549	41,98257618	29,43069139	2
ENSGALG00000002119	C16orf62	53,57445126	51,96391471	66,2690369	50,53612293	2
ENSGALG00000002316	NUBP2	79,87979134	66,76426362	113,9079487	71,91374142	2
ENSGALG00000002397	PSPH	16,90500002	11,13674451	38,37209435	14,53627012	2
ENSGALG00000002443	FBXL16	5,482262774	3,393627985	10,2747505	3,721226607	2
ENSGALG00000002460		169,5656604	134,6549735	199,9940187	147,1796955	2
ENSGALG00000002461		85,54594955	74,49743937	129,1652031	74,457145	2
ENSGALG00000002714	TMEM132C	81,17568898	53,4953987	178,5879937	49,72398397	2
ENSGALG00000002726	KIF1B	37,34066731	35,19418972	52,6551427	32,73497754	2
ENSGALG00000002923		42,50041281	25,78454685	58,32063392	24,5275742	2
ENSGALG00000002985	UTP18	59,80283029	50,99843338	74,38386445	58,7986555	2
ENSGALG00000003052	AGFG1	53,98350241	52,12101214	63,9794555	53,31865967	2
ENSGALG00000003078	MMD	166,6104847	155,0883677	202,3376044	148,475486	2
ENSGALG00000003180		107,8427517	81,65197474	140,4725058	96,80702055	2
ENSGALG00000003277	SUZ12	67,78173578	55,86030796	80,4002124	62,8663404	2
ENSGALG00000003295		11,95332143	9,200781695	16,90039539	8,047388377	2
ENSGALG00000003375		0,555521759	0,504517259	0,641921993	0,451150123	2
ENSGALG00000003391	EIF2AK1	120,3525644	98,69879665	149,048697	85,56996995	2
ENSGALG00000003427	USP3	144,6869467	126,2563974	186,8105836	108,1291213	2
ENSGALG00000003430	PMS2	83,93336261	66,63608403	99,6035854	60,47076563	2
ENSGALG00000003566	SMURF2	22,13122327	21,05696082	28,29312846	20,18762732	2
ENSGALG00000003739		107,7186679	79,57676443	217,3446885	92,92358891	2
ENSGALG00000003767	NKD1	64,58229372	56,04338743	82,66219062	54,31127427	2
ENSGALG00000003835		95,26245909	80,97210783	135,4218017	94,66924992	2
ENSGALG00000003957		7,497005362	7,280862747	12,32775655	4,077169463	2
ENSGALG00000004049	ITFG1	143,3658699	143,1696704	169,0452251	126,3388766	2
ENSGALG00000004121	LPAR4	57,41190777	51,21366709	73,35902376	56,01058859	2
ENSGALG00000004132		313,0280743	222,5345954	396,1457648	232,4516201	2
ENSGALG00000004144		230,9295811	165,493935	322,5375425	219,6317991	2
ENSGALG00000004169		52,0041871	45,80321214	71,82848643	48,04815439	2
ENSGALG00000004231	IFNLR1	13,20283795	12,15973039	22,07165879	11,62321498	2
ENSGALG00000004416	TTC19	35,18587556	33,81562749	46,08392978	33,45574377	2
ENSGALG00000004435	RAB27A	37,01217138	30,70100511	46,40397428	36,08556376	2
ENSGALG00000004474	PLEKHF1	16,35912847	14,33700972	22,89893142	16,04362022	2
ENSGALG00000004813	IARS	121,2613046	91,77804842	145,089873	101,0949477	2
ENSGALG00000005123	WNT7A	12,40158553	7,623339638	21,37318743	7,362852482	2
ENSGALG00000005177		102,6782147	98,37619348	120,4338593	98,08612871	2
ENSGALG00000005243	PPP3CB	90,43009538	80,11505572	110,9801319	75,05008011	2
ENSGALG00000005425	OGT	218,3232715	170,7793164	269,4024715	191,9762176	2
ENSGALG00000005427		2,231810001	1,699165441	2,481654149	1,214604019	2
ENSGALG00000005601		0,874226757	0,788269723	1,394307851	0,68739779	2
ENSGALG00000005685	KSR1	28,99559087	18,12035724	39,2301589	17,04233962	2
ENSGALG00000005689	Pax-2	2,427170557	1,41090904	9,598393861	1,637819447	2
ENSGALG00000005723		20,77746675	15,4473937	28,34501666	15,42480292	2
ENSGALG00000005796	XBP1	178,2861977	149,6817159	240,7969177	155,1233149	2
ENSGALG00000005815	TMEM41B	103,3957725	81,41002257	126,0115268	83,74442495	2
ENSGALG00000005845	SLC7A5	122,4599338	88,23427206	160,3761058	103,8001208	2
ENSGALG00000005854	CLUH	53,12207763	46,43056255	64,52154409	48,66611182	2
ENSGALG00000005945		312,5580735	266,4501752	377,6849137	289,237717	2
ENSGALG00000005966	SEC61A1	146,4192137	135,8133269	172,2380178	140,0010865	2
ENSGALG00000006080	GPC4	336,1866881	277,2326888	398,7191432	255,9354884	2
ENSGALG00000006138		5,3558423	4,222816208	9,559136283	4,692914386	2
ENSGALG00000006237	PKN2	94,14155442	80,04499622	133,7485665	89,13597808	2
ENSGALG00000006254	ERCC6	20,09113345	17,29890431	24,82822974	19,87584242	2
ENSGALG00000006256		81,30960437	70,77595812	104,1957411	69,99809839	2
ENSGALG00000006336		24,78170301	21,0855019	32,50165931	24,28317002	2
ENSGALG00000006378		46,64016235	43,92374135	60,27231414	45,47990755	2
ENSGALG00000006437		21,70370986	15,95524381	28,04172555	17,79255113	2
ENSGALG00000006797	ADARB2	2,386525484	1,723097368	5,609955946	1,217344116	2

ENSGALG00000006822	PCDH19	27,11782022	17,17899723	78,54913552	16,55189982	2
ENSGALG00000006856	CD151	66,08781237	56,83548982	81,90386525	57,64667081	2
ENSGALG00000006872	PISD	43,47551418	38,78352232	70,45275748	40,42254893	2
ENSGALG00000006895	CEP131	90,4838243	72,05921603	111,7343496	78,83214694	2
ENSGALG00000006935		41,19849281	36,85474382	50,28356686	36,31503506	2
ENSGALG00000006939	PFKP	159,5195891	114,2592094	248,2482576	153,3129457	2
ENSGALG00000006971		39,6698917	31,40577651	49,95181554	34,88964426	2
ENSGALG00000007141	LRRK1	8,471065321	7,23115594	11,52135294	7,124193969	2
ENSGALG00000007145	ITGB1	273,7290161	235,6454154	355,8809593	269,2892165	2
ENSGALG00000007234	CLCN5	26,22166298	22,62208897	33,75980899	24,31186251	2
ENSGALG00000007555	CCND1	306,4125901	154,2072849	365,1007316	180,3456931	2
ENSGALG00000007703		9,24001434	8,395210751	12,59696711	8,329025499	2
ENSGALG00000007870		87,32940421	79,94947931	110,2506191	70,91164374	2
ENSGALG00000007992	GGA3	36,45435135	33,68958392	46,79638773	32,15023613	2
ENSGALG00000008014	CEBPB	4,510096151	2,905838226	10,42101228	4,244775839	2
ENSGALG00000008088	ACSL4	42,77845399	39,53431922	53,52228442	40,19984773	2
ENSGALG00000008132		65,00574864	54,88816517	78,94598152	54,53613107	2
ENSGALG00000008240	CFLAR	18,56362706	16,85330063	24,63252397	15,72359014	2
ENSGALG00000008265		99,00016091	72,4970655	124,8646432	83,07442811	2
ENSGALG00000008352		2,250088005	1,0956429	4,791149948	1,598463212	2
ENSGALG00000008376	EIF2B5	94,27576083	72,47419873	115,9006191	78,67487542	2
ENSGALG00000008420	CAND2	23,37386731	21,50334329	30,87205611	22,23847698	2
ENSGALG00000008491		17,15532317	13,9477056	36,83972093	14,33742773	2
ENSGALG00000008547		64,24771593	51,30719206	82,19229622	44,61001661	2
ENSGALG00000008615		5,06223098	3,526819344	8,116738936	3,342214797	2
ENSGALG00000008705		70,28714953	55,76149858	84,361465	62,76428579	2
ENSGALG00000008811	SLC1A4	58,52949193	43,75140023	86,41435492	45,37141837	2
ENSGALG00000008837	PELI1	94,23917029	88,93657106	118,7910083	87,71875282	2
ENSGALG00000008848		228,2464595	192,5521067	285,6860563	202,9251398	2
ENSGALG00000008893		0,788890271	0,471371295	2,736222886	0,392092612	2
ENSGALG00000008918	LAMP5	14,93803939	12,88300339	33,54629897	9,821656942	2
ENSGALG00000009156	CDAN1	24,72426506	21,37975812	38,04864069	23,8875669	2
ENSGALG00000009157		87,96461416	76,74072672	108,7893047	79,4696583	2
ENSGALG00000009325		294,7081113	263,852235	362,0594636	288,1555786	2
ENSGALG00000009381		32,68110455	28,74970946	55,76103582	28,68002235	2
ENSGALG00000009405	GRIA2	1,407370493	1,108824914	2,875596002	1,012364018	2
ENSGALG00000009428	SUSD6	57,11791895	42,67061228	75,42226656	43,93842172	2
ENSGALG00000009448	IFRD1	202,5579686	184,5575928	256,5170811	193,8512693	2
ENSGALG00000009503	RAB3GAP2	27,58247884	24,32314054	39,58475158	27,79781709	2
ENSGALG00000009530	PDZRN4	4,501893374	4,232231688	8,897453929	3,14255931	2
ENSGALG00000009532	GXYLT1	80,28520746	50,71712672	113,1318587	48,13296544	2
ENSGALG00000009540	YAF2	59,24038791	44,15994271	76,99125086	41,14854067	2
ENSGALG00000009598	EPRS	197,6861501	158,8121803	232,2356108	176,423736	2
ENSGALG00000009748	ASNS	97,82806709	83,16578594	131,8122178	78,83074589	2
ENSGALG00000009782	NAA15	266,9481403	207,4047491	327,0135056	235,6097075	2
ENSGALG00000009899		236,5885982	203,9108633	284,3763821	211,3515889	2
ENSGALG00000009932	THADA	22,14429481	19,57605019	29,02730205	16,89609973	2
ENSGALG00000009992	ARHGAP5	58,77117318	59,03006064	73,92712	52,73589474	2
ENSGALG00000010185	HSPA4L	163,6669681	145,5240912	193,6623799	158,5908649	2
ENSGALG00000010344		156,9519341	147,9541769	185,7383997	153,6414155	2
ENSGALG00000010481	CMPK1	87,69769168	80,49136199	107,5540033	88,01504488	2
ENSGALG00000010536	FAF1	88,30370611	72,66469157	110,2520086	71,68965841	2
ENSGALG00000010603		84,1905846	68,02532034	109,3218398	73,68783444	2
ENSGALG00000010609	NDUFAF7	58,29146836	51,96812842	72,7689523	57,11232085	2
ENSGALG00000010975	TMTC2	23,82759321	19,13077263	32,54483622	21,60811044	2
ENSGALG00000010976	CCDC126	54,6027561	47,2667848	70,21267556	52,96789525	2
ENSGALG00000011045		26,46809802	23,19298715	40,20404704	23,42656319	2
ENSGALG00000011101	URB2	25,42984582	20,93888932	34,16483045	25,23812695	2
ENSGALG00000011162	CYP46A1	13,41648813	11,27861549	19,78883987	12,85367535	2
ENSGALG00000011228	WARS	106,1162631	99,35563125	133,5656722	91,79714635	2
ENSGALG00000011280	EEA1	45,16187273	37,63511378	56,03426363	37,5455685	2
ENSGALG00000011295	SOCS2	35,2007449	27,48311534	54,87659139	27,05575114	2

ENSGALG00000011319	ADGRL3	45,1852269	32,57841533	77,39214523	28,90106474	2
ENSGALG00000011351		1685,984803	1051,80178	2090,592418	1455,900582	2
ENSGALG00000011357	VEZT	46,17530033	43,8453212	57,83328907	44,85175026	2
ENSGALG00000011388	RABGGTB	110,4609778	75,06399545	135,3567109	76,64938861	2
ENSGALG00000011400	USP44	105,190674	89,81710894	130,7144947	91,51130572	2
ENSGALG00000011449		22,58118535	18,20250459	33,94025319	22,81520682	2
ENSGALG00000011464	EIF5	917,8237588	741,5110635	1254,948905	864,0505476	2
ENSGALG00000011541	BIN1	42,772136	38,21688698	55,05676669	37,86617878	2
ENSGALG00000011596		57,99322619	44,45105626	70,97049646	50,67792225	2
ENSGALG00000011630	GLI2	38,42268045	36,6914924	48,8882817	35,92337252	2
ENSGALG00000011643	UTP20	49,30168208	34,35447527	68,15857187	39,08059154	2
ENSGALG00000011708	MYLK	42,12023537	23,42722651	64,28850589	17,96485165	2
ENSGALG00000012012		37,18646392	31,88793184	65,7915782	35,088643	2
ENSGALG00000012135	ATF4	797,1581709	658,7569115	982,7650311	706,8909729	2
ENSGALG00000012138		509,5484881	456,9957633	634,8041273	493,2502814	2
ENSGALG00000012147	DDX18	65,05833895	45,55953755	80,75087614	54,70066068	2
ENSGALG00000012224		12,79412136	10,47045165	26,42294332	11,28060466	2
ENSGALG00000012278		69,38557328	35,10562561	151,1486425	26,90469823	2
ENSGALG00000012394	NID2	68,69887592	50,72697074	103,5972114	58,40743145	2
ENSGALG00000012412	MTHFD1L	68,14080099	40,88211832	86,10529775	42,0382293	2
ENSGALG00000012447		30,18390029	29,50018616	37,62561165	24,8779874	2
ENSGALG00000012594	NTRK2	2,037686782	1,161148992	4,803911879	0,826826974	2
ENSGALG00000012726		731,3536309	562,0189803	1152,278498	610,6379714	2
ENSGALG00000012807		41,90128094	38,20539641	52,18993579	41,4992427	2
ENSGALG00000012814	PXDC1	18,15321703	15,98836675	24,69297093	13,47136744	2
ENSGALG00000012834	AKR1D1	11,22123762	9,012546744	17,6164965	9,658495181	2
ENSGALG00000012916	DROSHA	22,62795344	20,36463746	27,36524709	21,85837675	2
ENSGALG00000012927		0,883855398	0,584154188	2,63363293	0,355129019	2
ENSGALG00000013124	FHOD3	15,42123161	11,36420449	27,34521932	13,3362274	2
ENSGALG00000013203		24,74101095	18,16871784	30,60325872	21,74233169	2
ENSGALG00000013480		63,83136272	43,0080344	89,59781528	60,13279899	2
ENSGALG00000013861	TNFAIP3	10,67839855	9,456256342	14,89948611	7,83500739	2
ENSGALG00000014343		2,461528107	1,903728583	5,19294882	1,881861539	2
ENSGALG00000014419		28,48257578	26,61598867	39,61862765	24,2985378	2
ENSGALG00000014426	KPNA1	75,53504815	58,7970706	92,09409276	65,46068843	2
ENSGALG00000014686	FBN2	3,824183353	3,617210217	5,312013174	3,28868843	2
ENSGALG00000014727	PDE4D	3,095516476	2,120314733	9,004244594	1,906866262	2
ENSGALG00000014784	MAST4	7,644433311	6,829537967	10,49021956	7,439951494	2
ENSGALG00000014873	GJA1	151,5200503	125,8535441	213,8081133	139,1697079	2
ENSGALG00000014902		36,12871703	36,02307188	48,16761796	31,1982202	2
ENSGALG00000015049		107,1047214	88,92587671	135,8651695	102,4480149	2
ENSGALG00000015112		52,46535646	48,47995695	77,60078191	38,76419637	2
ENSGALG00000015164		8,124039618	6,253772744	15,92231741	8,079666445	2
ENSGALG00000015472		103,7455883	84,8260084	125,5639489	82,46251332	2
ENSGALG00000015765		57,24314739	44,13731219	76,00962273	51,54508175	2
ENSGALG00000015862		174,4258517	150,9505279	265,4828154	159,1555857	2
ENSGALG00000015887	URB1	25,51642297	20,47718015	41,57109807	25,13000945	2
ENSGALG00000015912		32,16222318	29,58832844	40,98127953	26,04493072	2
ENSGALG00000015940		20,73939007	19,85946192	29,04560533	14,93146904	2
ENSGALG00000016412	MBOAT2	20,66710203	20,80239859	26,79137108	15,68219706	2
ENSGALG00000016455	GREB1	71,30258407	44,76556849	171,8842869	40,7988828	2
ENSGALG00000016480	SDC1	67,79712381	46,12962808	101,6542603	65,96909209	2
ENSGALG00000016808	RANBP2	88,68408088	76,82428287	126,4454672	83,37223599	2
ENSGALG00000016875		35,81511404	23,39580851	81,48141489	21,24280635	2
ENSGALG00000016896	ABCC4	9,804323199	7,015685475	14,63344086	7,987146561	2
ENSGALG00000016897	GPR180	25,35505379	21,30681925	31,82543062	22,00243895	2
ENSGALG00000016910	RBM26	141,5608071	114,5852063	174,753134	120,2734436	2
ENSGALG00000017085	SLC7A1	40,05394599	35,00050584	54,14362264	38,81530443	2
ENSGALG00000017111	NUP58	63,43269188	54,90711803	79,68018034	60,76739727	2
ENSGALG00000017365		22,49317964	17,18921742	33,78900494	20,82699363	2
ENSGALG00000017388		186,1814132	172,046714	268,9548589	178,5764003	2
ENSGALG00000017462	FNIP1	23,16041033	20,29852756	34,80619268	20,97040204	2

ENSGALG00000019077		75,80953752	63,82999065	101,13045	49,73846401	2
ENSGALG00000019622	TRIM71	63,53466997	45,08282347	138,6372475	59,01429912	2
ENSGALG00000019961	SLC18B1	94,7023836	82,41511543	144,636966	86,89618913	2
ENSGALG00000020479		23,15151259	19,80281907	35,36804246	21,72259355	2
ENSGALG00000020805	LRRC8D	52,23662484	47,43227859	70,9545852	43,87669915	2
ENSGALG00000021316		95,97879893	77,14187045	118,2758942	74,31764513	2
ENSGALG00000023451	TRPM7	25,75064391	21,92560329	34,73493332	22,8366183	2
ENSGALG00000023576	PIM3	38,39240852	36,6823612	50,24199049	36,47779083	2
ENSGALG00000024039		104,7252252	86,46498974	126,7964154	101,8972811	2
ENSGALG00000025770		5,489257369	3,00959702	12,02421921	5,106431368	2
ENSGALG00000025884	NABP1	53,416184	46,44733288	68,89014188	48,27093752	2
ENSGALG00000026349		276,236352	197,8659478	354,8447655	217,6076426	2
ENSGALG00000027316		2,126519018	1,150096401	4,599057408	0,899918777	2
ENSGALG00000027342		54,55996923	43,14565226	88,63782029	46,00922333	2
ENSGALG00000027355	TPBG	77,04597047	65,11404966	96,88531936	76,00938707	2
ENSGALG00000027389	RGS7BP	16,07367378	12,09558999	26,33652515	15,7130332	2
ENSGALG00000027757	ARL4A	92,36112237	89,07729168	139,8864616	67,39567749	2
ENSGALG00000028462		49,20550825	40,59815974	63,79574475	48,48730827	2
ENSGALG00000028821	GLIPR2	73,84703902	51,69185499	185,78976	66,36412003	2
ENSGALG00000029023	DMRTB1	0,460922202	0,311878344	1,17310912	0,418308344	2
ENSGALG00000000054		40,61041039	77,77927106	101,7475053	79,46683091	3
ENSGALG00000000162		0,462119365	0,938839881	1,64917459	0,88469459	3
ENSGALG00000000385		31,22124771	34,99404406	41,45690236	38,58983564	3
ENSGALG00000000409	CHMP7	79,5962766	87,42800269	94,16850493	90,97109387	3
ENSGALG00000000546		13,1839576	15,33846461	17,00256672	16,22873609	3
ENSGALG00000000721	SLC41A1	10,56410509	14,67315988	25,86101352	19,0181628	3
ENSGALG00000001007		249,8579466	266,9325144	356,1755387	297,3682242	3
ENSGALG00000001482		39,02660104	42,05429417	46,83086881	45,01081844	3
ENSGALG00000001789		5,660481882	5,998946978	8,128074203	6,887258106	3
ENSGALG00000001962	PTBP1	232,0447099	243,7695722	332,3908518	271,6208432	3
ENSGALG00000002232		38,27382102	41,1639609	46,66833375	42,91091967	3
ENSGALG00000002248		31,30319367	32,96247972	49,67058945	40,65598095	3
ENSGALG00000002294	SEMA6A	10,49550142	13,58348745	24,74662011	18,10914393	3
ENSGALG00000002326		1,47250781	2,63934996	4,3086282	2,825363581	3
ENSGALG00000002335	DOCK3	1,86448435	2,175929741	3,65664367	2,786015113	3
ENSGALG00000002336		31,11744712	33,42002163	37,20470924	36,29167307	3
ENSGALG00000002521		18,85761021	20,11966762	24,33563548	21,95444037	3
ENSGALG00000002604		56,3890493	58,74501369	73,02098131	67,097638	3
ENSGALG00000002745		150,7994546	155,5390263	180,6431172	172,3193816	3
ENSGALG00000003113	RGS14	32,70926878	38,37080551	45,95420135	39,64318938	3
ENSGALG00000003488		1,062824324	2,489829504	4,41900121	3,4655683	3
ENSGALG00000003634	PTEN	109,6705257	112,8873555	130,5075766	116,9977413	3
ENSGALG00000003701	SGMS1	9,258518873	11,10229042	15,94820162	13,26938247	3
ENSGALG00000003948	ALAS1	49,59705625	55,47941093	71,59274829	59,09107457	3
ENSGALG00000004088	FNBP1	24,99237284	26,63166615	41,69591151	33,84795116	3
ENSGALG00000004158		10,55351282	11,6407991	18,6163799	13,95560906	3
ENSGALG00000004181		10,65424926	12,03452833	16,95695499	13,00094636	3
ENSGALG00000004384		69,61318883	72,98146284	91,46967536	84,65324587	3
ENSGALG00000004509		973,1657851	1201,316866	1361,543225	1311,496279	3
ENSGALG00000004517	PARP3	6,608038224	7,4623483	10,339109	8,753186004	3
ENSGALG00000004573	LRRC8A	0,06745578	0,071659756	0,107430357	0,088247902	3
ENSGALG00000004594	CD74	4,406632912	8,750656652	24,51781075	14,099464	3
ENSGALG00000004661		55,81884913	71,36370724	89,93526626	84,78952034	3
ENSGALG00000004730		2,547946628	4,415127922	9,113776156	6,91193394	3
ENSGALG00000004917		2,896199012	3,859495387	6,644848881	4,974527218	3
ENSGALG00000004974	PPARG	1,096913427	1,387820745	3,376628365	2,480137643	3
ENSGALG00000005310		4,865705062	6,154572974	9,226338957	6,706461454	3
ENSGALG00000005492		10,69479144	14,25428111	24,12257422	16,1669692	3
ENSGALG00000005678	FLNB	60,41912172	63,75180733	77,98540804	71,20030379	3
ENSGALG00000005692		3,071738847	4,652874224	7,830445668	5,084013812	3
ENSGALG00000005990		0,702479687	0,971890089	2,092257816	1,181810364	3
ENSGALG00000006097	PLXNA1	90,9441087	93,67817119	112,3883703	102,2276242	3

ENSGALG00000006252	LMO4	39,86339327	44,78129011	86,70760634	62,88011361	3
ENSGALG00000006258	SPOCK1	35,25706314	52,38093264	93,08564779	55,08217348	3
ENSGALG00000006314		2,490008645	4,28813785	7,168503958	6,122938793	3
ENSGALG00000006417	SEC24A	58,74346813	64,91074036	75,01570455	66,74698384	3
ENSGALG00000006638	GRIK4	10,90795756	15,65522755	23,06474952	17,01050771	3
ENSGALG00000006717		0,341376117	3,858277819	12,25143393	5,62632686	3
ENSGALG00000006786		9,374384568	13,921691	19,00994587	16,68642534	3
ENSGALG00000006845		2,670363578	3,667942238	4,464215057	3,687958638	3
ENSGALG00000006928		28,56711841	31,59803573	37,31258837	32,05630269	3
ENSGALG00000007140	NRP1	6,786804779	9,937661813	29,18904165	20,3705717	3
ENSGALG00000007608	ARHGEF9	19,95586878	33,81382649	49,630279	46,62145769	3
ENSGALG00000007707	TRAPPC4	89,44703604	93,8442147	110,2275432	97,87185693	3
ENSGALG00000007712	MYO1B	15,61469896	20,14680824	26,22598793	22,24150912	3
ENSGALG00000007920	PRR5L	4,129042771	4,972105698	7,776312534	6,401104872	3
ENSGALG00000008018	SNAI1	4,838614652	6,837750638	10,0616924	8,716434475	3
ENSGALG00000008128		75,28877718	82,09362626	103,3431878	93,65315249	3
ENSGALG00000008206	TESC	3,628035071	6,771511577	8,739719175	7,526555407	3
ENSGALG00000008321	RNPEPL1	22,85721363	26,24567137	33,01671824	30,40006594	3
ENSGALG00000008359	CD93	1,29957602	1,606526404	2,626230481	1,994368504	3
ENSGALG00000008445	SLC24A3	7,111549609	8,166183777	13,26231133	11,30603962	3
ENSGALG00000008623	TOR4A	2,885819993	3,8654735	7,453674406	5,093186098	3
ENSGALG00000009145		1,464711516	1,626807682	2,784818386	2,107621671	3
ENSGALG00000009293	CHN1	67,90118322	74,31595236	82,89622495	76,74118835	3
ENSGALG00000009393		6,562399308	15,29420916	43,36617594	24,96798456	3
ENSGALG00000009808		1,416304393	2,421872238	5,557291922	2,900189483	3
ENSGALG00000010005	EPAS1	1,665911612	3,648797813	8,298542118	6,247343231	3
ENSGALG00000010035	NR3C2	6,203251658	9,632638993	13,68730791	9,212635965	3
ENSGALG00000010081	SH3D19	9,151663444	12,27542336	21,57827913	14,7478447	3
ENSGALG00000010090	FAM160A1	2,972923275	4,146500246	6,55742234	5,035528514	3
ENSGALG00000010186		26,1218641	34,11416012	41,89387746	40,73222649	3
ENSGALG00000010518		0,144840281	0,417602932	0,617619694	0,379300378	3
ENSGALG00000010597		4,611954486	9,57844156	19,361201	10,00647113	3
ENSGALG00000010780		14,90096854	16,74023252	20,06717072	17,00357201	3
ENSGALG00000010910		5,56043356	11,09159137	18,79138106	10,94754966	3
ENSGALG00000010996	IRF2BP2	74,78507418	89,5496145	142,7037833	109,2878448	3
ENSGALG00000011061		0	0,089561338	0,194908996	0,087418642	3
ENSGALG00000011076	SYNE3	0,555939022	0,853639426	2,736597554	1,544551691	3
ENSGALG00000011220	SLC25A29	13,82485106	20,4490233	37,69585477	30,16838999	3
ENSGALG00000011232		8,944661229	10,28962659	16,59653933	12,22287995	3
ENSGALG00000011241	RFTN1	4,68880177	5,893184053	7,360556398	6,707841258	3
ENSGALG00000011550		40,28733517	55,05082442	67,35622718	54,65736176	3
ENSGALG00000011613		4,739203641	5,310547233	9,592133476	7,686518403	3
ENSGALG00000011831	ANKRD50	13,06309751	25,09232183	32,26143246	25,9987912	3
ENSGALG00000011963	PRDM5	2,076763979	3,308086443	4,532414832	3,751882733	3
ENSGALG00000012033	SGSM3	12,06891416	14,05699702	17,1981993	14,96637778	3
ENSGALG00000012111	TMEM37	3,030173772	3,743044977	7,331132284	5,289018921	3
ENSGALG00000012407	LRP1B	0,189428611	0,330664455	0,574724991	0,367586865	3
ENSGALG00000012449	MBD5	36,89274386	42,54116552	53,76802751	44,82551839	3
ENSGALG00000012462	KIF5C	65,21507153	73,27025091	90,38041221	78,71948215	3
ENSGALG00000012739	HIVEP1	15,82020811	17,77656476	21,4826725	19,34743987	3
ENSGALG00000012850	RAB19	3,884293705	4,594800469	7,321110934	5,597645823	3
ENSGALG00000013060	ADCY2	1,064982925	1,936215345	2,96245522	2,691019878	3
ENSGALG00000013085	PTPRO	8,814882072	9,635541753	16,31598922	11,48544595	3
ENSGALG00000013204		11,67890458	12,83559276	15,73940433	14,21451059	3
ENSGALG00000013605	LRRC16A	24,93445177	27,46094865	32,7533139	29,62259302	3
ENSGALG00000014139		48,56470481	50,53915714	62,44067282	56,14372316	3
ENSGALG00000014371	LGALS8	42,99482411	51,47866425	59,99952802	56,0859926	3
ENSGALG00000014441		10,1718582	12,15414806	16,15178081	12,91195223	3
ENSGALG00000014545		5,504651212	7,224808336	8,631274642	6,873714829	3
ENSGALG00000014834	NCOA7	17,62360916	20,65742055	26,25090959	22,94517424	3
ENSGALG00000014841	HDDC2	155,8070969	174,4976494	189,514832	184,7270869	3
ENSGALG00000015196	ASXL3	12,54053734	13,3023841	16,82687164	15,16050633	3

ENSGALG00000015355	CD47	76,26899784	118,0330955	173,0471949	137,0561941	3
ENSGALG00000015653		3,796339821	6,553253791	10,50484987	6,581133531	3
ENSGALG00000015667		15,19349969	17,21199916	19,65556076	17,55600721	3
ENSGALG00000015721	SVEP1	1,737960293	2,245209146	3,02298257	2,299009295	3
ENSGALG00000015858	DOPEY1	10,26362443	12,06032829	15,43817177	12,56861101	3
ENSGALG00000015865		8,149500097	14,67315572	23,51658618	18,36456973	3
ENSGALG00000015995	PTPRA	111,1055577	127,974971	137,2289686	127,4894494	3
ENSGALG00000016285		18,08199823	20,16991605	23,48414703	21,6556274	3
ENSGALG00000016329	AGPAT5	54,88045679	57,69984224	65,85606335	59,46993298	3
ENSGALG00000016350	ARHGEF10	13,20943184	16,47206961	19,97855358	16,74087613	3
ENSGALG00000016356	TDRP	19,61514474	24,85234346	31,91658421	26,97607924	3
ENSGALG00000016443	HPCAL1	45,49523459	60,94224423	91,38350236	82,37034179	3
ENSGALG00000016719	TNFRSF21	32,04606952	36,62371331	51,90970368	46,80574718	3
ENSGALG00000016746		6,325273962	6,601627683	8,839401918	7,699088309	3
ENSGALG00000016779	RNF149	40,32057066	43,89003588	53,7136991	48,40680769	3
ENSGALG00000016909	NDFIP2	79,60051734	96,09155119	149,2317403	108,9565955	3
ENSGALG00000016927		4,423680336	5,818898304	11,01889315	6,732269887	3
ENSGALG00000017112	MTMR6	27,31233071	28,93025574	35,58260609	30,94460943	3
ENSGALG00000017283	CCND2	58,83812867	71,53308904	78,60163688	74,96269338	3
ENSGALG00000017335		49,94809765	58,41748149	65,31328378	61,44158464	3
ENSGALG00000017715	TINAGL1	0,390432351	0,886129706	1,981143569	1,219554501	3
ENSGALG00000021812		1,33055689	2,218290992	4,819468653	3,797137631	3
ENSGALG00000022887	KBTBD11	9,128381161	10,94614726	24,8936814	16,96298322	3
ENSGALG00000022909	PTPN3	1,705551313	2,69191956	5,75680131	4,605522677	3
ENSGALG00000023027		5,819536568	8,734844706	12,14989903	8,702812739	3
ENSGALG00000023193		22,82628116	26,83374341	46,03186329	34,00628372	3
ENSGALG00000023857		23,87214226	28,64172799	31,048651	28,83857214	3
ENSGALG00000026093		5,630092855	7,023865753	9,045481093	6,813892067	3
ENSGALG00000026484		0,135024738	0,263566954	0,838178415	0,546375363	3
ENSGALG00000026728	AMMECR1L	29,84210004	31,09949395	35,82038624	32,81834419	3
ENSGALG00000026776	TIGAR	13,56828479	16,77103564	21,89957869	19,88878977	3
ENSGALG00000026795		0,217228639	0,495115948	2,256733718	1,191236832	3
ENSGALG00000027070	TIMP2	63,034473	97,3863476	154,0452617	134,7888119	3
ENSGALG00000027241		0,952560408	1,466719985	3,039748701	2,207159329	3
ENSGALG00000027255	NRXN3	0,181357946	0,830745305	2,361320482	1,14505164	3
ENSGALG00000027521		0,404163847	0,994883411	2,02843061	1,126800761	3
ENSGALG00000027835	PCBD1	94,80491215	113,5855851	151,1573142	114,9552317	3
ENSGALG00000027983	TNK2	4,828489899	7,027495256	8,351294461	7,398414931	3
ENSGALG00000028242		6,695143255	8,395783618	9,169385188	8,751478338	3
ENSGALG00000028935	BCL10	31,45990136	34,35834737	53,25704847	43,70890102	3
ENSGALG00000000177		3,672925592	4,7133494	28,69442229	13,64228432	4
ENSGALG00000000581	DDX42	79,71475033	75,27436341	94,20135848	80,66368967	4
ENSGALG00000000638	UTP4	130,0676455	96,70332836	165,3136722	132,8315835	4
ENSGALG00000000652	ACOT7	91,0618764	84,44688017	110,5059683	102,0297592	4
ENSGALG00000000875	PHLPP2	20,54132997	19,47427289	25,59885106	23,08633198	4
ENSGALG00000000890		115,4827014	99,93575405	161,284949	135,4581557	4
ENSGALG00000000891	NSUN5	53,01503719	44,67338132	77,96581988	55,35290206	4
ENSGALG00000000949	HBEGF	1,41847004	1,85041107	12,65482264	5,806391814	4
ENSGALG00000001020		29,88405432	26,56154792	64,98663159	38,60596779	4
ENSGALG00000001025	GOLGA1	22,45573384	18,51150657	29,42118809	24,79417751	4
ENSGALG00000001194		212,5442816	205,1105713	287,0990995	265,262679	4
ENSGALG00000001364	CSNK1A1	415,1207983	409,9539609	501,5029834	437,3747681	4
ENSGALG00000001382	CDC2L1	72,94889565	67,80678106	88,43393376	81,89331757	4
ENSGALG00000001546	ZZEF1	12,49878851	12,56771979	17,15985806	15,54466993	4
ENSGALG00000001551	CCNL2	122,413604	108,8267176	162,3532986	139,7824135	4
ENSGALG00000001876		9,90311991	9,249057674	14,02958487	10,97007453	4
ENSGALG00000002198	FHOD1	5,26743915	4,954885979	9,38561863	6,836917746	4
ENSGALG00000002222	NOC2L	118,3584902	95,02050883	142,0720819	116,3738506	4
ENSGALG00000002362	MANF	364,1212991	353,3282859	462,1312606	409,2625306	4
ENSGALG00000002364		32,94239485	32,71450827	39,97668091	35,39664528	4
ENSGALG00000002399		52,36416197	46,22509547	70,78493069	56,5784979	4
ENSGALG00000002471		16,17379426	15,90253266	23,9774057	19,02253072	4

ENSGALG00000002709	MYO1C	16,40141681	14,00693265	25,42132948	18,29325483	4
ENSGALG00000002759	PEAK1	12,11070695	12,37294828	18,07755992	15,14928933	4
ENSGALG00000003039	SRM	110,2374056	95,18663787	138,4535202	109,8554395	4
ENSGALG00000003043		22,75792127	21,97305286	39,26576174	30,68440091	4
ENSGALG00000003082	DPH1	43,35222965	39,90415548	56,22303159	46,64836959	4
ENSGALG00000003130	DDX49	65,08104231	64,76205282	78,85615956	71,53496308	4
ENSGALG00000003181	SURF2	68,66649596	63,22622727	93,24040328	77,05363503	4
ENSGALG00000003401	FBXL22	1,842586497	2,069041413	5,714050546	4,460181323	4
ENSGALG00000003563	YARS	124,5878605	100,806762	161,5804147	129,3590756	4
ENSGALG00000003726	EGFLAM	3,127640917	2,882156629	6,50447413	3,899600608	4
ENSGALG00000003830		56,83320796	53,87981451	71,17442725	65,73104839	4
ENSGALG00000004042		38,46767509	32,97068481	61,87801155	49,83556181	4
ENSGALG00000004063	SLC25A14	52,88533686	51,42823816	66,67629574	61,35330486	4
ENSGALG00000004089		17,77256296	16,78271592	24,42500908	19,86038229	4
ENSGALG00000004098	STK4	78,3035915	77,0974588	96,93702951	88,80516834	4
ENSGALG00000004200	DPP9	53,75067211	45,69989026	71,29460307	57,55657601	4
ENSGALG00000004295	CPD	62,31416544	60,36967779	77,78486944	67,0127332	4
ENSGALG00000004437	LHFPL2	20,63775236	19,68831498	34,32158717	26,52799986	4
ENSGALG00000004625	ATXN2	88,67526317	82,01836417	109,5403049	102,5728716	4
ENSGALG00000004662	DISP3	14,38521556	11,55502683	29,05261461	16,89063595	4
ENSGALG00000004691		56,33686982	40,4318144	71,73587757	59,00776865	4
ENSGALG00000004820	FAM57A	12,31601366	10,07982253	19,55856559	14,55945516	4
ENSGALG00000004938		54,1479251	51,08009956	67,80947377	62,11469064	4
ENSGALG00000005139	CLTC	200,1181658	191,6793455	253,6981165	209,8034908	4
ENSGALG00000005318		3,152263541	2,662414727	5,120954069	3,784663378	4
ENSGALG00000005433		36,83290793	32,95618637	55,24825615	43,10703235	4
ENSGALG00000005464		85,83962449	85,8443126	105,9021193	93,37178801	4
ENSGALG00000005582	KLHL18	31,94479309	31,30348133	42,78533543	37,78377686	4
ENSGALG00000005653	NFKB2	19,40013661	19,08669938	32,80863272	23,57889165	4
ENSGALG00000005660	NF1	25,50711294	23,71387031	34,53169071	28,24626635	4
ENSGALG00000005668	WSB1	179,678984	146,1632157	214,4435918	176,4064239	4
ENSGALG00000005679	CDX1	1,304631914	0,905701649	2,563014719	1,644766779	4
ENSGALG00000005691		0,879450332	1,016907284	4,287680194	2,372582554	4
ENSGALG00000005727		87,5175031	54,87972446	124,3665649	87,29261047	4
ENSGALG00000005962		96,05596249	97,74823491	129,0244635	115,5419182	4
ENSGALG00000006225	EFL1	25,89461358	24,34066277	33,72870887	27,27818148	4
ENSGALG00000006430		34,42578206	34,74030494	42,66924729	38,00467817	4
ENSGALG00000006512	HSPA8	1476,479912	1243,966272	1904,025233	1566,091754	4
ENSGALG00000006564		135,1349308	137,694922	164,941611	147,7888853	4
ENSGALG00000006569		30,28902795	30,34830504	44,14456419	34,89953162	4
ENSGALG00000006703	GTPBP4	263,023663	226,6648498	306,5407089	275,5959813	4
ENSGALG00000006908	DLG1	79,94395383	69,9741556	167,5563942	97,80401695	4
ENSGALG00000007056		14,79571572	13,46626993	33,62262451	21,76104972	4
ENSGALG00000007174	TNFSF15	1,102995748	0,448114415	2,915856114	1,855636786	4
ENSGALG00000007241	NUBP1	109,3181308	94,68377161	138,0537018	121,3146554	4
ENSGALG00000007278	GRIN2A	1,410215689	1,099864815	2,433976143	1,483307418	4
ENSGALG00000007312		0,515818805	0,324808533	0,640115412	0,516898894	4
ENSGALG00000007387	GTF3C1	49,4431244	44,10989653	61,73807855	50,46690808	4
ENSGALG00000007434	RHBDF1	8,971085939	8,883515652	14,18157043	11,77887017	4
ENSGALG00000007516		62,43238732	57,39706836	74,52591155	62,64255662	4
ENSGALG00000007603		107,5989261	79,43933012	130,7533918	107,5767645	4
ENSGALG00000007659	HYOU1	247,2830843	202,3775908	337,8470085	253,6943761	4
ENSGALG00000007704		344,7122616	259,8186212	567,3248203	425,2898747	4
ENSGALG00000007793	UPRT	63,74779044	56,0337098	86,10974443	72,54448635	4
ENSGALG00000007845	REL	11,42236566	10,4053628	17,28277767	14,80106617	4
ENSGALG00000007846		0,386598336	0,938692576	50,96425002	26,68289287	4
ENSGALG00000007932	TRAF6	10,42206821	10,02548702	20,10355496	15,77060665	4
ENSGALG00000007957		27,06104057	24,98161486	33,66292504	31,22532025	4
ENSGALG00000008097	CELF1	26,96388959	26,53468446	34,24988615	30,0909987	4
ENSGALG00000008163	PSME4	46,80933972	45,11907125	79,1287345	59,67413509	4
ENSGALG00000008182	CHAC2	6,548181465	7,336110999	17,67917062	12,66985204	4
ENSGALG00000008211	ACP2	64,70328045	58,45883411	80,09941154	66,54844454	4

ENSGALG00000008263	CNTN4	1,106200523	1,176160283	3,185854517	2,100686659	4
ENSGALG00000008348	PDIA3	746,0442099	742,0928002	909,6299808	852,0733662	4
ENSGALG00000008414	DVL3	135,6013387	130,367946	176,8576759	158,0362434	4
ENSGALG00000008520	EIF4G1	229,5376092	185,6983546	301,8025317	265,7430302	4
ENSGALG00000008521		31,18069235	29,21280914	43,31917635	33,76297669	4
ENSGALG00000008680		3,676371429	3,695854361	6,469859596	5,35658988	4
ENSGALG00000008708		37,14584777	32,77792521	49,18084278	37,57520453	4
ENSGALG00000008778	SPRED2	34,65788535	37,41244323	69,179714	49,64306392	4
ENSGALG00000008942	FOXN2	31,91191136	31,77313307	38,7914746	33,70013914	4
ENSGALG00000009129	DLX5	10,01865983	5,040758016	22,8485431	12,34243947	4
ENSGALG00000009172	OSBPL6	2,650564571	2,756249804	5,544541276	3,837527585	4
ENSGALG00000009534		25,76733875	24,62278561	35,59283804	27,46733311	4
ENSGALG00000009546		10,46067246	7,836081786	30,46916994	17,16886901	4
ENSGALG00000009594	RARRES1	9,3163689	9,894726709	19,208027	15,83960019	4
ENSGALG00000009699	CCNL1	91,8351549	82,06759115	115,5386418	102,8629539	4
ENSGALG00000009906	UBR2	22,46888792	22,12414082	27,23693376	24,46789564	4
ENSGALG00000009946	HECTD1	48,79520303	46,18145293	63,02540497	54,54830376	4
ENSGALG00000010036	FOSL2	1,42569897	0,793171681	12,63940938	7,470718426	4
ENSGALG00000010049	PTPRB	0,961664682	1,128851705	3,443635365	2,118202631	4
ENSGALG00000010084	EIF4G3	114,1687657	103,5506191	143,5390186	117,7492928	4
ENSGALG00000010098	SLC6A9	21,12603755	15,71173611	30,01896704	26,093241	4
ENSGALG00000010167	PNN	265,0067262	220,0057882	322,9605906	271,8747079	4
ENSGALG00000010349	POLR1C	117,943623	93,52322591	145,73653	117,5515932	4
ENSGALG00000010361		48,97012579	44,43975772	68,27866824	55,34965362	4
ENSGALG00000010786		4,097877003	2,931870248	5,490876839	4,103642012	4
ENSGALG00000010836		3,785106733	3,728133151	7,708117179	4,823935284	4
ENSGALG00000010967		58,3636706	56,63549114	79,71286329	65,06813368	4
ENSGALG00000011055		3,528425682	2,14343308	3,539716441	3,81794966	4
ENSGALG00000011058	LEPR	0,561037727	0,528085813	2,462715898	1,549528016	4
ENSGALG00000011331	CTH	248,0035867	236,6542483	303,6507875	275,389819	4
ENSGALG00000011419	DUSP4	44,01115652	33,26358857	121,1807682	85,68812834	4
ENSGALG00000011537	PDE10A	17,52900263	13,79619641	27,86596367	20,34592748	4
ENSGALG00000011833		29,27658949	25,74330458	117,5407031	60,95401121	4
ENSGALG00000011962		24,37861772	19,78836623	76,1380067	54,90216003	4
ENSGALG00000012137	KTN1	52,45719129	52,70647636	63,46102016	56,68222967	4
ENSGALG00000012202		52,23663855	47,13644927	66,99015096	54,26052413	4
ENSGALG00000012342	SASH1	23,67583218	24,00657812	31,8459396	26,74112268	4
ENSGALG00000012354		38,36252665	31,9693923	72,19373709	50,92066877	4
ENSGALG00000012404	ERO1A	22,83857753	22,01192185	30,88099896	28,27942365	4
ENSGALG00000012410		67,06196987	58,3818171	81,80427215	74,10521197	4
ENSGALG00000012414	GNPNAT1	66,78273296	56,41713595	92,31540052	73,43548792	4
ENSGALG00000012626		14,77954066	13,73791725	18,79074056	15,62077944	4
ENSGALG00000012696		60,55715685	57,86504	99,67821995	75,06643816	4
ENSGALG00000012702	JARID2	37,33195628	36,20655994	105,618813	76,98869311	4
ENSGALG00000012847		0,986713969	0,845118709	4,302990379	2,59612048	4
ENSGALG00000012852	MKRN1	230,2783715	221,0113476	347,0213055	264,4287667	4
ENSGALG00000013454		182,0155039	123,4948983	229,2326813	190,0588303	4
ENSGALG00000013616	OPRM1	8,498418048	8,602322585	13,37881711	10,98826365	4
ENSGALG00000013677		30,75467791	29,88810558	39,45604034	37,02318823	4
ENSGALG00000014080		0,21595968	0,263148688	1,065482871	0,727785613	4
ENSGALG00000014445		231,7075249	162,1297126	273,7779191	224,85464	4
ENSGALG00000014795	TGIF1	90,42983611	80,36474464	117,3826664	93,7149106	4
ENSGALG00000014880		7,049861195	6,527543364	11,03857212	8,462532694	4
ENSGALG00000014922	ROCK1	58,13294478	55,12527106	91,44699065	66,17491261	4
ENSGALG00000015180	PSAT1	39,87210776	38,25469818	74,32407226	52,26746661	4
ENSGALG00000015184		18,40942927	17,96441873	31,32066199	23,65139942	4
ENSGALG00000015271	FILIP1L	1,507114952	0,978526739	2,740725006	1,723861266	4
ENSGALG00000015284	RGMB	11,87565903	10,63508532	17,84314469	15,52968056	4
ENSGALG00000015310		26,26727406	23,40911182	43,92421285	29,60330699	4
ENSGALG00000015820		5,795989287	4,835828284	10,12157143	6,826990388	4
ENSGALG00000015908	COL12A1	1,280525315	0,982791397	3,642005312	1,798732148	4
ENSGALG00000015930		89,77271382	89,18055945	105,8631291	96,52936382	4

ENSGALG00000015942		25,13739734	24,29186435	34,37777216	29,63188224	4
ENSGALG00000015980		0,596196735	0,41874926	1,674898262	0,878230845	4
ENSGALG00000016308		26,44250919	19,32816829	50,33957199	31,74534839	4
ENSGALG00000016387	TRAPPC12	41,96858601	38,40737819	51,57440402	44,44505587	4
ENSGALG00000016506	ZNF512	88,06015215	79,02693958	104,7277824	96,40793662	4
ENSGALG00000016513	PPM1G	230,2916367	196,6821079	271,5136768	260,5494092	4
ENSGALG00000016764	EIF5B	216,2000449	194,9595544	249,9786739	241,9844265	4
ENSGALG00000016803	ST6GAL2	35,94066123	32,07201411	58,08741372	43,47710939	4
ENSGALG00000016807		34,55977522	25,87689197	63,00840571	44,52494993	4
ENSGALG00000016816	CDC16	62,16393798	54,16495998	74,3131724	66,19654442	4
ENSGALG00000016817	RASA3	18,72143367	21,1947238	46,6794009	30,14801484	4
ENSGALG00000016841		23,36476602	15,1644545	49,52648097	33,88640549	4
ENSGALG00000016843	COL4A2	23,50684554	16,00417492	47,20989436	25,07421643	4
ENSGALG00000017185	TMEM123	82,3272294	82,74278569	123,1509651	97,26527034	4
ENSGALG00000017320		172,8178042	158,3012361	208,2067185	188,3508122	4
ENSGALG00000017418	NPTX1	2,015625875	1,372808327	4,33776454	3,060994409	4
ENSGALG00000018879		6,563062255	5,094820232	11,25672365	8,341936654	4
ENSGALG00000019054		0,728717128	0,463400019	6,735112481	2,212402684	4
ENSGALG00000019470		1,444283154	1,413792107	3,796706053	2,057755864	4
ENSGALG00000020899		6,083043676	4,511874861	13,31624301	8,922200915	4
ENSGALG00000022490		3,542294964	4,40309386	17,43885586	11,12767909	4
ENSGALG00000023241		14,69724223	12,08407893	57,94569675	29,73935332	4
ENSGALG00000023725		74,22983509	68,94311731	125,647953	97,24504122	4
ENSGALG00000026034	RGS17	3,024836883	2,866775508	5,550569778	4,08214868	4
ENSGALG00000026251		0,057739324	0	0,072028586	0,0498639	4
ENSGALG00000026379		0,919332817	0,655580292	2,303728052	1,597097731	4
ENSGALG00000026468	CHSY1	71,12020109	60,49110129	111,0111548	79,65498057	4
ENSGALG00000026631	HOXA10	0,174683257	0,06671589	0,623564049	0,246427429	4
ENSGALG00000026862	CLDN1	14,1101416	17,88491877	498,851265	292,4470901	4
ENSGALG00000027057		45,65853393	41,50345279	55,55225266	47,46219036	4
ENSGALG00000027132		21,35779753	20,57984953	27,44116286	24,14699039	4
ENSGALG00000027526	COL9A2	18,60130136	18,58810977	27,51237863	22,80853569	4
ENSGALG00000027696		17,22300656	13,40010504	27,54529579	21,46414333	4
ENSGALG00000027707	ESM1	0,150067881	0,073206173	1,347708981	0,510169001	4
ENSGALG00000028279	SNORD14	59,56148275	36,73539954	85,15192697	70,25858401	4
ENSGALG00000028675		43,07663481	36,65018625	58,259897	43,676594	4
ENSGALG00000028773		88,44681977	88,70929071	105,8823606	98,70306764	4
ENSGALG00000000166		4,929206982	8,868613541	10,16203898	7,489518013	5
ENSGALG00000000874		27,5257408	42,0642166	38,71313331	35,44203257	5
ENSGALG00000001443		61,90702775	100,220611	84,17995064	70,86083476	5
ENSGALG00000001741	NPTN	112,9186561	136,9981308	138,6382684	124,8191766	5
ENSGALG00000001926	HSPB1	26,87936471	51,36562943	52,8540106	47,19220813	5
ENSGALG00000002602		3,588473784	4,875391746	5,77341269	4,458144988	5
ENSGALG00000003126	ERBB4	1,696937945	7,085918708	11,05966427	3,505512894	5
ENSGALG00000003263		76,37963484	99,04824669	105,8462646	85,42780449	5
ENSGALG00000003578	FN1	62,38375818	70,31597704	75,66792654	66,94626169	5
ENSGALG00000003642	PDGF-A	28,92734021	56,03765372	46,12301374	39,27720096	5
ENSGALG00000003748	ANO5	25,09400369	31,77740288	32,82517973	30,40964521	5
ENSGALG00000004071	NETO2	43,78776373	55,53083264	54,37099849	37,75449027	5
ENSGALG00000004118		7,47667819	26,22733823	20,20577066	14,30878756	5
ENSGALG00000004184	SPARC	155,0540441	232,7699504	211,3314171	199,4811275	5
ENSGALG00000004215	WIPI1	33,13808616	43,77015696	42,74430902	40,4803601	5
ENSGALG00000004519	TRPV2	0,822447882	1,60414895	2,273876542	1,268485872	5
ENSGALG00000004686		30,2279262	36,35087632	37,69253986	35,20409282	5
ENSGALG00000004710	TMOD3	46,23733435	62,98353115	66,8079787	60,43811176	5
ENSGALG00000004724		14,31585256	20,5405451	19,66998379	18,70531832	5
ENSGALG00000005223	PAC1	12,65150175	17,99234789	18,81912824	15,2360441	5
ENSGALG00000005230	MED13	32,91669497	36,87653151	39,04153735	36,48895089	5
ENSGALG00000005256	EPHA4	42,66846892	78,56615799	96,36961707	62,46596208	5
ENSGALG00000005512		47,88593846	69,10907971	81,90943527	52,75638588	5
ENSGALG00000006472		1,419681564	4,536593254	3,288727942	2,539433944	5
ENSGALG00000006705	MYOF	2,618645578	7,53302833	6,251568851	4,810717487	5

ENSGALG00000007343		0,49771636	3,066447888	2,335443105	1,682163718	5
ENSGALG00000007766	KIAA1217	5,142338869	9,945849569	7,737757011	6,6003621	5
ENSGALG00000007809	TSHZ2	5,283631458	12,9087792	13,412855	11,27929818	5
ENSGALG00000008053	PRDM11	21,30498282	24,55027354	26,50984007	23,21540024	5
ENSGALG00000008222	SLC26A5	29,61861728	40,31425403	46,54677335	38,47187747	5
ENSGALG00000008281	NEURL1	0,643796003	2,406738252	2,878119044	1,315326778	5
ENSGALG00000008670		60,9188267	92,15789368	112,4269423	81,96474528	5
ENSGALG00000008903	SSFA2	9,137506036	12,52608672	14,56587518	11,95743887	5
ENSGALG00000009163		8,759302894	14,89860879	16,52575933	11,8340751	5
ENSGALG00000009378	PDGFC	12,77846886	24,19271297	24,15556174	22,07089313	5
ENSGALG00000009556	PRICKLE1	10,09396138	19,24833012	25,35963008	17,81681241	5
ENSGALG00000009645	ESRRG	13,80191386	17,39972079	19,42718421	14,40652117	5
ENSGALG00000010311	NAV3	0,615932694	1,312817613	1,821822919	0,975422452	5
ENSGALG00000010470	TCERG1L	2,421080738	7,105845544	7,441496756	5,889451234	5
ENSGALG00000010588		0,618826163	7,910358704	6,674385318	4,860719678	5
ENSGALG00000011047	LEPROT	49,88870182	65,03197595	64,41076564	61,76206291	5
ENSGALG00000011254	SATB1	11,10630754	13,32672382	15,03624216	12,84351007	5
ENSGALG00000011288	DACT2	43,3233096	77,67540397	56,71321695	50,47908136	5
ENSGALG00000011373	TECPR2	14,79142682	18,36494399	18,18702754	17,49834935	5
ENSGALG00000011539		57,67498946	85,42119822	84,42143077	72,20633676	5
ENSGALG00000011542		58,95969276	74,88826879	78,93145405	60,78404348	5
ENSGALG00000011680	EPHA5	8,20621494	24,36580423	24,51946644	14,89187836	5
ENSGALG00000012251		20,12025104	26,36888417	27,50762152	21,56503412	5
ENSGALG00000013031	CECR1	5,057170782	7,128107118	8,312127528	5,595783665	5
ENSGALG00000013134		22,82793318	29,22780923	31,32115935	27,27461137	5
ENSGALG00000013503	FAM149A	1,752925892	4,848988383	4,853083076	3,295521827	5
ENSGALG00000013710	SNX9	28,51304311	33,65160133	35,14415843	30,51214921	5
ENSGALG00000013768	PHACTR2	16,97204936	22,53311454	26,1533975	21,45971135	5
ENSGALG00000015013	MSX1	272,3083235	372,1131576	353,787571	293,7007336	5
ENSGALG00000015096	SH3GL2	6,752855595	9,972778277	10,62531102	9,697118369	5
ENSGALG00000015167	PRUNE2	0,376231707	0,978364886	0,754818696	0,73083871	5
ENSGALG00000015193	CCDC80	14,67870793	25,22532121	21,73900557	21,1053184	5
ENSGALG00000015215	MAPRE2	117,8139702	164,251409	175,9162941	136,884432	5
ENSGALG00000015409	PROS1	45,426329	66,08672514	83,84526057	55,91392118	5
ENSGALG00000015794		5,438251718	7,227879991	7,741266482	6,719009922	5
ENSGALG00000015797		0,53814002	7,856916105	2,807378812	2,732711798	5
ENSGALG00000016160		8,263899107	10,02238742	10,67328648	9,407543829	5
ENSGALG00000016258		0,878895793	3,431767393	3,190781752	1,828922771	5
ENSGALG00000016276	KHDRBS2	17,88353402	32,13830872	27,94112843	21,08977544	5
ENSGALG00000016281	DMD	17,81837254	29,46320013	29,11375678	20,07434969	5
ENSGALG00000016495	KLHL29	7,027776784	9,00783271	9,910837711	8,941313628	5
ENSGALG00000016776	TBC1D8	8,308297301	10,63215235	11,46728711	10,62476118	5
ENSGALG00000016839		23,55110961	30,45895529	34,20721695	28,99322678	5
ENSGALG00000016947	ELF1	16,00351494	19,80439985	20,80439973	18,68259934	5
ENSGALG00000016981		0,057692799	0,773832888	1,091983863	0,323032301	5
ENSGALG00000017246	ME3	13,03979429	22,01093333	24,71107053	22,21530556	5
ENSGALG00000017411	RXFP3	0,51168238	1,422949404	2,090496742	0,518778426	5
ENSGALG00000024035		5,499637289	10,63733364	13,88164669	9,819516571	5
ENSGALG00000025747	NQO2	8,133537598	12,93516452	11,59383961	10,23613308	5
ENSGALG00000026258	MAF	0,778820903	9,324146442	13,1495057	6,561829291	5
ENSGALG00000026370		0,891124883	2,751588481	2,510061088	1,175691721	5
ENSGALG00000026486		4,237996386	7,869569204	6,981784134	3,839481651	5
ENSGALG00000026991		1,07803858	1,847127388	1,949984514	1,262349842	5
ENSGALG00000027627		30,89543317	50,46301542	50,55295596	43,60585579	5
ENSGALG00000027947	GHSR	0,104688776	0,849436519	0,862659404	0,749153227	5
ENSGALG00000028602		13,19864069	32,69987166	40,67321367	22,05665612	5
ENSGALG00000000151	ADAMTS19	11,80611965	12,46514745	7,204855475	9,17871014	6
ENSGALG00000000227	DPYSL2	270,6999858	268,6183531	219,938641	254,0376962	6
ENSGALG00000000280	EFNA5	24,32055926	20,52988368	11,64998519	17,31698677	6
ENSGALG00000000362		37,35064361	32,02718335	27,44376501	33,1655256	6
ENSGALG00000000449		49,2665107	49,10237718	33,20160451	41,94914256	6
ENSGALG00000000529	LIMD2	93,67951598	94,89616479	75,32979772	80,66727455	6

ENSGALG00000000547		3,018242875	2,863860974	1,595558221	2,347638467	6
ENSGALG00000000584		3,261584505	2,895004253	2,038105409	2,118177535	6
ENSGALG00000000607	GPR37L1	5,416118593	4,00865424	2,523565581	3,30135529	6
ENSGALG00000000678	CITED4	15,62473381	11,43958359	7,239375596	9,048783186	6
ENSGALG00000000692	CDK18	22,23095371	21,04019437	14,37658239	15,37207022	6
ENSGALG00000000812	OAZ1	489,9056014	482,0065382	407,200401	467,4568226	6
ENSGALG00000000839	WNT11	2,75489007	2,199440661	1,264090823	1,917561529	6
ENSGALG00000000912		46,3292383	41,75530213	31,67896574	36,03517849	6
ENSGALG00000001264	PLXNA2	58,22770217	53,91327501	24,13638965	32,04174249	6
ENSGALG00000001374		104,8241518	112,9489393	90,04261095	91,50863097	6
ENSGALG00000001472	JAM3	100,011492	93,74068448	82,19173947	92,91975166	6
ENSGALG00000001506		368,3526839	372,9183268	296,0295963	339,4166627	6
ENSGALG00000001567		126,5813492	129,435459	100,5746864	111,6667323	6
ENSGALG00000001611	B3GAT1	7,43006463	8,500157291	3,962658545	5,321053943	6
ENSGALG00000001916	SLIT3	25,76301015	25,02868434	16,99253818	21,07931886	6
ENSGALG00000002005		5,413669282	5,77801867	2,751506276	4,550706014	6
ENSGALG00000002024	COMT	27,25339768	29,45942369	19,71910445	20,44636948	6
ENSGALG00000002028	GPRC5B	14,34185212	14,07342152	5,786874678	9,906480205	6
ENSGALG00000002040		79,93552599	81,85898438	64,79086608	61,98429748	6
ENSGALG00000002041		59,40242733	57,27740971	48,38350615	52,07872637	6
ENSGALG00000002056	TANGO2	45,88582661	46,90045634	35,03961486	37,17107403	6
ENSGALG00000002116	TEN1	20,56756935	19,76690486	13,13442349	13,44002686	6
ENSGALG00000002155		76,89660127	81,52844767	49,12868241	64,57841702	6
ENSGALG00000002377	ENO1	5212,537489	5074,505023	4322,535611	4651,610527	6
ENSGALG00000002552		10,14344218	8,496682359	6,743722308	7,370594553	6
ENSGALG00000002561		27,30204849	27,24602203	20,69160555	25,05439519	6
ENSGALG00000002645		15,71672487	13,70701973	8,631292786	8,68302629	6
ENSGALG00000002897	SLC16A14	27,20069002	23,63489477	17,04976801	16,06268463	6
ENSGALG00000002909		111,6429653	108,8364841	65,45516928	74,21179258	6
ENSGALG00000002911	MOXD1	4,443996829	3,766709566	2,890068858	2,950915419	6
ENSGALG00000002952	NRG4	28,16643542	25,91401013	16,97766028	18,81496187	6
ENSGALG00000003193	CRABP-I	372,078802	401,8286749	212,1153567	250,9542932	6
ENSGALG00000003212	TSPO2	9,375586046	7,497418907	5,455285302	5,632090299	6
ENSGALG00000003322	TBC1D2B	23,0364406	23,04385432	18,28372397	14,42807756	6
ENSGALG00000003374	ESRP2	4,290851004	4,01526902	2,786837195	2,82526351	6
ENSGALG00000003473	SFRP1	198,6174832	161,8765433	117,5653707	121,237755	6
ENSGALG00000003539	KIAA1522	48,54035458	41,79698536	34,13776991	42,25080563	6
ENSGALG00000003550	ARHGAP40	3,317838701	2,653438966	1,952460676	2,348843771	6
ENSGALG00000003602		17,85228161	18,32582058	13,19044577	14,56512893	6
ENSGALG00000003732	UCK1	74,42929071	77,91477397	61,41002009	65,22123954	6
ENSGALG00000003742	CPAMD8	22,90988764	20,34187827	16,93949378	18,38217809	6
ENSGALG00000003829	HPCAL4	9,672599215	8,323189404	5,220820337	7,313009587	6
ENSGALG00000003894		39,49235103	48,65174612	23,31361969	26,19853057	6
ENSGALG00000003965	DBX1	120,9552444	117,7217027	51,14908628	70,42580158	6
ENSGALG00000003973	TLCD1	112,8068814	117,1801993	73,74619124	94,39823275	6
ENSGALG00000004048	PTPRS	155,7119771	161,4097176	128,6427922	143,3686471	6
ENSGALG00000004249	GRHL3	6,349924787	5,24388785	3,935762529	4,71839621	6
ENSGALG00000004270	ALDH1A2	27,69749738	24,65884251	18,46023442	21,00172637	6
ENSGALG00000004370	MAP2K6	29,31711165	33,73015033	16,64289398	21,44172167	6
ENSGALG00000004527		2,134308872	2,240087571	1,027730715	1,26194997	6
ENSGALG00000004598	CUX2	10,91100525	10,38753268	7,769537303	10,06444635	6
ENSGALG00000004849	WSCD2	45,34791706	50,70050864	22,81269623	34,91261466	6
ENSGALG00000004888	RAI1	40,93525626	33,1953892	27,30738815	31,72051679	6
ENSGALG00000005036	SH2D3C	19,05266339	20,32939106	12,13835847	15,76846777	6
ENSGALG00000005064	PRPSAP2	180,1816199	171,8333543	152,3830656	151,8777115	6
ENSGALG00000005107		70,58508054	63,43166224	49,22045308	51,47493334	6
ENSGALG00000005396		24,53292592	23,39277847	16,09342546	13,16614788	6
ENSGALG00000005409		9,602911865	11,56291556	4,665267822	5,939605674	6
ENSGALG00000005608	SLC9A3R2	41,76346794	36,24125491	25,01105653	35,56871411	6
ENSGALG00000005616	ARSI	10,31329519	8,403075571	5,912173057	7,138945964	6
ENSGALG00000005632	MRVI1	1,035430484	0,888938675	0,506588986	0,613816863	6
ENSGALG00000005886	WSCD1	42,42581136	36,95926631	26,36940368	25,78087186	6

ENSGALG00000005976	TCEA2	151,1204022	158,0299347	129,1939558	140,5406725	6
ENSGALG00000005981	GDF2	35,21583503	34,95950736	15,63020753	18,34501736	6
ENSGALG00000005985	GDF10	151,8179242	133,3393762	68,22657308	67,90626041	6
ENSGALG00000006211	BCL2L1	77,71688202	69,24101045	46,01690957	57,52750738	6
ENSGALG00000006277		43,37142919	41,65643624	34,95474443	37,42762996	6
ENSGALG00000006341		2,477772914	1,863998893	1,22355805	1,629215459	6
ENSGALG00000006360		116,2259025	108,0171956	99,60835707	100,7079967	6
ENSGALG00000006420	ABHD17C	43,96192153	44,25815693	36,5118052	39,97659664	6
ENSGALG00000006456	C16orf45	126,2885268	123,5755496	103,0104563	113,0436349	6
ENSGALG00000006539	C21orf2	91,92359624	96,93066537	72,63220666	80,75071846	6
ENSGALG00000006576	GRM3	5,093834571	4,402339701	1,89484859	3,657846954	6
ENSGALG00000006583	LSP1	130,1339628	114,8168042	87,05056318	96,05195296	6
ENSGALG00000006662		4,073009343	3,076860613	1,949960342	2,615131782	6
ENSGALG00000006715	OAF	67,7069681	52,68685915	39,6578323	46,72294434	6
ENSGALG00000006886	DACH2	97,17562698	96,3669717	74,86391205	74,85847215	6
ENSGALG00000006887	FAM174B	113,7792578	123,4632073	89,0301308	92,30488335	6
ENSGALG00000006950	LIMK2	39,24605928	44,80038999	28,89988718	31,66912322	6
ENSGALG00000006982		28,22770649	26,3955686	20,28855166	21,73180767	6
ENSGALG00000007007		3,672988192	3,675424255	2,403461423	2,636506344	6
ENSGALG00000007127		72,79567328	70,87318067	59,36657504	64,29580864	6
ENSGALG00000007131		1741,940063	1718,967686	1405,400638	1577,061404	6
ENSGALG00000007132	ACOX2	3,920015175	3,800327799	2,227110374	3,369924911	6
ENSGALG00000007364	TOB1	25,92250443	23,41006218	15,10205731	18,83966369	6
ENSGALG00000007441		37,78990923	39,61953583	19,20223432	28,70034349	6
ENSGALG00000007719		8,587739597	8,673743587	4,950497049	4,790200635	6
ENSGALG00000007819	PDZRN3	7,790604113	6,689038204	4,884979375	6,326245839	6
ENSGALG00000007839	NCAM1	224,900862	248,3471659	164,3211414	191,3588384	6
ENSGALG00000007912	ATP9A	14,30496017	14,40885784	11,23211708	12,79682857	6
ENSGALG00000007945	CRYAB	28,66809483	20,95669537	10,44303707	14,14521444	6
ENSGALG00000007993	DCX	35,56507544	35,44942575	22,95234521	19,99682824	6
ENSGALG00000008034	CASKIN2	15,6733548	15,28382385	10,1352996	13,19245044	6
ENSGALG00000008060	PRKAR2B	56,53549837	55,35469483	39,37569127	48,11630919	6
ENSGALG00000008136	C2orf69	23,07666382	22,96082979	16,74740759	19,66350146	6
ENSGALG00000008152		44,0565263	40,40259712	32,97020753	34,00914523	6
ENSGALG00000008164		16,82913916	16,88116082	13,13788045	11,98403826	6
ENSGALG00000008297	SEMA4B	63,40218087	60,59213273	34,32024933	46,40412182	6
ENSGALG00000008555	MXI1	62,54223051	63,65172867	39,52038549	50,53829158	6
ENSGALG00000008579	OIP5	80,39985195	71,04664556	62,04889257	65,37711843	6
ENSGALG00000008677	VIM	1934,220794	2091,395034	1528,513774	1633,778736	6
ENSGALG00000008774		240,3536889	235,8958052	155,5629281	192,5922241	6
ENSGALG00000008850	TTLL7	9,855630847	9,638036441	6,465694185	7,831903916	6
ENSGALG00000009004	ZNF804B	0,528433071	0,560499823	0,242320416	0,44430822	6
ENSGALG00000009048	ABLIM1	10,71327222	8,768191528	6,002604343	7,651491713	6
ENSGALG00000009064	FZD1	94,20126626	80,37022502	67,72298945	75,05676907	6
ENSGALG00000009248	HSPA12A	32,76381377	29,41540416	19,35339196	24,0607483	6
ENSGALG00000009280	NPY2R	7,52643434	6,490731964	4,241120616	4,842042787	6
ENSGALG00000009291	EPHX1	24,12810147	21,68268279	16,94897373	14,68925954	6
ENSGALG00000009362		91,11750451	103,1144581	72,59595314	68,03634497	6
ENSGALG00000009475		366,4300954	326,2865393	265,7944967	289,0206255	6
ENSGALG00000009559		62,46382877	63,35737381	50,60514824	51,23070493	6
ENSGALG00000009604	DBX2	188,02469	164,9576559	71,47102484	112,4678314	6
ENSGALG00000009650		40,35619744	41,46793477	31,51222177	31,26515047	6
ENSGALG00000009799		131,9263511	123,4148587	88,50089928	102,2744474	6
ENSGALG00000009905	MDM1	34,94608598	38,49309929	27,93640968	26,68582306	6
ENSGALG00000009948	HHIP	34,10489404	29,81672032	12,8659564	17,40556709	6
ENSGALG00000010055	DAAM2	7,571786163	6,688168824	4,066430756	4,819714522	6
ENSGALG00000010158	KANK1	34,42266386	33,10703465	25,60147101	25,08690202	6
ENSGALG00000010243	PRDX1	1920,279994	1901,867506	1624,165646	1809,468067	6
ENSGALG00000010711	NXPH1	1,336267919	1,08347215	0,412641959	0,793884376	6
ENSGALG00000010745	HMGB2	960,0569077	950,3575761	759,8576275	846,3521199	6
ENSGALG00000010758		80,09122628	85,39793585	63,98441519	61,48005141	6
ENSGALG00000010761	MRPL37	104,7510396	105,0379991	87,00391806	96,37058418	6

ENSGALG00000010854	HDAC9	15,52765661	13,90606664	8,67588231	11,10862945	6
ENSGALG00000010892		94,54684796	97,06101349	74,78496364	77,87489575	6
ENSGALG00000010940	ACSS3	17,0859282	19,73733137	11,03537862	13,6172326	6
ENSGALG00000011019	ROR1	31,89158503	33,92332362	19,77815514	25,04993277	6
ENSGALG00000011083		31,53400696	29,83533586	21,70010114	21,42515155	6
ENSGALG00000011117	AGT	7,011848699	6,40595233	3,225035171	4,179528638	6
ENSGALG00000011205	SMOC2	28,04565303	26,98470908	9,630259431	18,70884659	6
ENSGALG00000011283	ZNF385D	2,605818014	2,669322669	1,67305957	2,163474606	6
ENSGALG00000011296	FRMD1	70,2232087	67,45990844	55,38004143	52,65139895	6
ENSGALG00000011298	RARB	55,69535963	47,68028638	38,04727303	37,68492663	6
ENSGALG00000011442	TGFBR2	2,861068196	2,68853117	1,838107115	2,275891693	6
ENSGALG00000011487		37,86246542	33,98107732	21,65815303	19,53005182	6
ENSGALG00000011518	POMGNT2	15,30539131	14,01183671	10,32877903	9,911142688	6
ENSGALG00000011532		55,69108076	52,76270126	42,60190613	42,40119097	6
ENSGALG00000011558		23,50941161	20,49599556	17,43700181	18,64310313	6
ENSGALG00000011580		116,9362646	111,1956453	101,032554	102,3871869	6
ENSGALG00000011592	MRAS	26,78163289	27,15769346	16,17293361	23,32241226	6
ENSGALG00000011657	EAF2	88,86209982	78,74142964	59,13328659	70,88662684	6
ENSGALG00000011712		27,43874052	26,29278103	16,13911667	16,52237231	6
ENSGALG00000011742	ART4	2,842510701	2,786801503	1,453508753	1,821990845	6
ENSGALG00000011826		24,11633262	24,94919055	16,13711683	18,0713928	6
ENSGALG00000011848		61,83068956	50,7503725	40,99509459	46,82618647	6
ENSGALG00000012006	RTN1	15,5752017	15,69038812	8,141532846	10,07584334	6
ENSGALG00000012010		12,13552249	10,81139678	8,109147776	7,96587428	6
ENSGALG00000012082	FAIM	44,71906582	40,82570202	33,31693495	29,35247649	6
ENSGALG00000012094	SLC35F4	3,504266079	2,479954589	1,868276354	2,82870976	6
ENSGALG00000012158		144,978586	127,8854779	106,8748489	109,7215444	6
ENSGALG00000012219	UNC5C	21,57499882	22,95837716	14,58740088	16,32433091	6
ENSGALG00000012282	L2HGDH	20,93313563	22,32547931	15,22212186	15,47060553	6
ENSGALG00000012308	MANBA	11,89786869	12,7530861	8,200203397	9,308437527	6
ENSGALG00000012346		7,862982605	8,864901197	5,102603107	6,396826485	6
ENSGALG00000012569	FBXO7	73,79359843	76,51114178	60,47045786	56,31379388	6
ENSGALG00000012575	TLE1	42,64744251	40,35590358	26,26275038	30,7525678	6
ENSGALG00000012621		23,12197697	21,50098662	17,51990666	20,73745298	6
ENSGALG00000012747	NEDD9	51,16943075	46,55544661	25,2270541	28,40372824	6
ENSGALG00000012748	ELOVL2	6,703664016	5,77345016	4,279673705	3,893667093	6
ENSGALG00000012796	KIAA1549	51,09108109	44,95257214	38,63565476	39,44896655	6
ENSGALG00000013182		64,54611055	57,87116822	44,54062532	41,58771622	6
ENSGALG00000013194		37,97153678	35,58529342	22,21115632	30,79003586	6
ENSGALG00000013207	CTNNAL1	96,43762659	88,20907865	78,48817168	78,03326849	6
ENSGALG00000013297	BBOX1	23,95421511	20,28591096	13,84454117	17,01177863	6
ENSGALG00000013363		59,76145701	53,34909219	27,20240346	30,97263445	6
ENSGALG00000013505		3,889722018	3,478768697	2,452222386	2,376785773	6
ENSGALG00000013869	IL20RA	6,972034921	7,7365372	3,434256512	4,89649334	6
ENSGALG00000013948	RASL11B	44,13163783	48,97517759	18,53083963	33,62688077	6
ENSGALG00000014033		104,7477162	94,29244852	81,03494353	95,32221883	6
ENSGALG00000014094	STK38L	63,92061824	56,75448411	49,03019712	53,65761291	6
ENSGALG00000014262	APBB2	50,41157639	49,52225086	39,71176444	41,3726837	6
ENSGALG00000014627		6,186249674	6,570860566	3,973407227	5,195659962	6
ENSGALG00000014648	CETN3	120,6378615	124,7051507	91,07219377	107,9915591	6
ENSGALG00000014749	EPHB6	39,83982973	37,84869471	30,92879956	29,01876058	6
ENSGALG00000014853	CLVS2	20,14220368	18,4145602	10,94311348	13,28443107	6
ENSGALG00000014971	SLC2A9	5,990387675	5,899169872	4,692471627	4,901640879	6
ENSGALG00000015037	EVC	23,92704947	26,1129777	17,06719452	14,17492822	6
ENSGALG00000015109		47,46943411	46,36864366	35,65399625	39,11989952	6
ENSGALG00000015349	PIGG	15,28388747	14,19631757	12,44050293	12,25488892	6
ENSGALG00000015368	TMEM175	40,97091402	42,09879659	30,18566566	35,6655243	6
ENSGALG00000015399	FUT10	5,766769243	5,749814663	3,113288123	5,094696357	6
ENSGALG00000015512		12,75994149	13,90362938	8,228539011	10,70286882	6
ENSGALG00000015542	PLPPR1	116,2967487	112,3139725	75,3404969	69,59821363	6
ENSGALG00000015593	EPHA7	23,76270266	25,30203349	15,93621129	16,69520231	6
ENSGALG00000015628	MSANTD1	27,98934213	27,89487479	19,00355994	16,65250297	6

ENSGALG00000015661		25,53972785	27,31771949	16,46254751	21,18719894	6
ENSGALG00000015702		165,4779657	153,2689949	126,1182243	131,223407	6
ENSGALG00000015713	FAM53A	97,02921321	94,00902169	72,8675515	69,6370911	6
ENSGALG00000015749		21,2120221	17,80838104	14,06053735	17,19977004	6
ENSGALG00000015812	SMIM8	22,70170198	23,41415321	15,5950842	19,1637852	6
ENSGALG00000015880	HMGN3	377,9392173	394,1557704	311,1143085	339,3443829	6
ENSGALG00000016027		94,40142812	100,8328093	71,31951897	75,50602922	6
ENSGALG00000016067	HMG14	441,5841588	451,8120476	376,4578579	378,3157428	6
ENSGALG00000016100		16,68836719	14,10822856	9,670667046	14,44480754	6
ENSGALG00000016161		20,22006123	21,56436875	14,06276308	15,46768923	6
ENSGALG00000016222	NDP	75,17879418	59,52253055	36,29361092	33,58711701	6
ENSGALG00000016318		27,27843983	23,46508248	18,58560473	20,47415343	6
ENSGALG00000016390		37,86603951	38,28740842	29,30950252	35,44055736	6
ENSGALG00000016403		266,8591886	263,757375	206,1924561	172,5882396	6
ENSGALG00000016411		14,25340121	13,22735653	5,731016335	9,570264498	6
ENSGALG00000016477		32,59657778	30,23039623	26,7611813	26,75620295	6
ENSGALG00000016511	ADGRG2	2,386466849	2,957863341	0,902910309	1,512144222	6
ENSGALG00000016570		5,798752701	5,090455098	2,401852442	4,386433754	6
ENSGALG00000016636		5,617816157	5,062765544	2,035430911	2,908022952	6
ENSGALG00000016731		239,7537815	224,2247214	208,7586328	215,0388526	6
ENSGALG00000016895	DZIP1	27,04772767	25,34766727	21,42357243	21,88134556	6
ENSGALG00000017015	RNASEH2B	56,39861841	51,8363735	44,83361055	48,79351186	6
ENSGALG00000017032		58,5366442	56,1349687	48,61797983	48,88052127	6
ENSGALG00000017074		4,074357168	3,691118729	1,412239719	2,952723128	6
ENSGALG00000017119	TNFRSF19	58,66491746	57,05921994	42,25493843	32,20227457	6
ENSGALG00000017133	EEF1AKMT1	26,99940931	29,0525506	20,74451252	21,42944072	6
ENSGALG00000017195	PGR	3,608790529	2,632589836	1,517382064	1,94031908	6
ENSGALG00000017229	FAT3	8,89802285	8,313903578	6,609729949	6,008519085	6
ENSGALG00000017247		35,90192669	33,73272811	24,29212924	26,07653344	6
ENSGALG00000017302	SLCO2B1	1,429813469	1,146573087	0,673898438	0,668516203	6
ENSGALG00000017371		51,38692014	50,91225956	38,09521913	45,83153263	6
ENSGALG00000017405	NPR3	23,00399694	22,53783009	13,33544434	18,73145328	6
ENSGALG00000019198		17,59843277	15,08692745	10,51068089	8,621485841	6
ENSGALG00000019514	CNDP2	182,9891291	180,1386682	158,0964217	167,2312883	6
ENSGALG00000019719	KRT19	37,68434018	33,76138595	21,75346913	23,72609315	6
ENSGALG00000020575		8,11802815	7,979917996	3,72206627	6,630432556	6
ENSGALG00000020615	STK32C	11,47277599	10,0537539	4,973755997	7,984705212	6
ENSGALG00000020688		7,755595976	7,74751763	3,581525145	6,288357343	6
ENSGALG00000020876		2,382805079	2,166538193	0,848276969	1,89263607	6
ENSGALG00000020956		38,20475834	33,66535769	30,02776671	33,98390736	6
ENSGALG00000020984	FAM163A	20,23037736	16,5164079	11,68786047	14,02896446	6
ENSGALG00000021285		186,2854475	165,9217969	136,042098	155,4656808	6
ENSGALG00000021653	IFT22	101,3073949	105,5647369	78,19787743	75,492151	6
ENSGALG00000022891		63,01409828	62,3114166	49,31573941	51,86061496	6
ENSGALG00000022967		19,86053476	14,53555584	9,909871032	11,74545585	6
ENSGALG00000023073		75,25252995	70,70073732	46,37176598	61,58963078	6
ENSGALG00000023279	EPN3	18,11156599	18,44465948	11,60965438	15,0198188	6
ENSGALG00000023542		44,21934953	41,11173749	33,22451866	36,77576397	6
ENSGALG00000023683	TCTN2	77,20303141	74,18428746	64,19070348	58,30899806	6
ENSGALG00000023904	FIBIN	19,51600353	16,39135199	8,430598252	11,5313733	6
ENSGALG00000024294		3,558273438	3,080519379	1,266138207	1,949493615	6
ENSGALG00000024481		133,9861745	137,3212927	96,10190154	120,9686545	6
ENSGALG00000024486		127,4593345	112,050928	75,08512045	74,30239813	6
ENSGALG00000025915	ARRDC3	26,46755421	22,42622815	19,05714962	23,18559721	6
ENSGALG00000026055	PALM	62,96970401	58,46982113	37,62754481	51,55881293	6
ENSGALG00000026119		19,50030797	15,03597578	10,89450307	13,56917513	6
ENSGALG00000026153		13,21809934	13,27180391	6,109358729	11,47472691	6
ENSGALG00000026351		7,714996646	7,132791831	4,733453719	4,737550727	6
ENSGALG00000026388		7,176755468	5,70158915	3,356921795	5,377783467	6
ENSGALG00000026424	TOR2A	49,43334651	51,09796496	40,22928877	42,49356237	6
ENSGALG00000026460		35,89548571	39,92113818	22,86822683	27,96586662	6
ENSGALG00000026544		58,65981865	57,10160799	41,59272235	50,81686876	6

ENSGALG00000026560		36,96829613	26,69799391	17,90105267	27,48806165	6
ENSGALG00000026622		3,176811988	2,665491808	1,529135988	2,006855906	6
ENSGALG00000026704	GPX8	14,6970356	14,21488529	8,762242734	6,869731027	6
ENSGALG00000026722	UNC119B	143,9059999	141,8749309	120,3644467	119,8965562	6
ENSGALG00000026941		11,45825275	12,65274753	7,360856213	7,871403016	6
ENSGALG00000027154		32,85298841	26,87435096	15,98536481	21,99669163	6
ENSGALG00000027300		255,9415831	208,7324314	140,1583855	136,9067811	6
ENSGALG00000027454	CENPW	133,7719182	117,0934874	105,1892275	115,178855	6
ENSGALG00000027498	FOXB1	141,2191776	137,5736463	78,19797441	120,6686286	6
ENSGALG00000027648		297,8558779	297,8555526	235,0735679	265,6448529	6
ENSGALG00000027655	TRABD2B	3,732000135	2,929243457	1,949007079	2,75895498	6
ENSGALG00000027714	ATG10	14,65174394	14,12262445	10,09229515	11,13826046	6
ENSGALG00000027891		245,592978	266,7752548	119,3002211	176,3890275	6
ENSGALG00000027907	NR2F1	167,8585793	148,627158	100,6374936	126,2471647	6
ENSGALG00000028005	GADD45G	26,61578991	21,03730188	12,66743452	14,80395902	6
ENSGALG00000028015		40,97729593	39,21000061	27,73413561	27,479133	6
ENSGALG00000028142	HACD4	1,371297566	1,176318136	0,666595527	0,732081177	6
ENSGALG00000028168		21,67520772	22,20929179	18,06713907	16,59662597	6
ENSGALG00000028221		9,866386893	9,886670244	5,735567879	7,645874692	6
ENSGALG00000028506	NHSL2	18,71016948	15,75409848	8,290834081	13,78916943	6
ENSGALG00000028508		19,24169204	18,49325928	11,74503381	15,41263549	6
ENSGALG00000028745		8,953044339	8,076698766	3,902548389	4,23747302	6
ENSGALG00000028931		107,4437216	87,27433427	55,7926251	77,21406238	6
ENSGALG00000029012		14,73441004	15,72458847	5,28285304	8,311412343	6
ENSGALG00000000040		23,44475508	28,21280039	14,22402533	24,43401832	7
ENSGALG00000000209	PRNP	10,74066283	12,46930325	6,402314152	9,988641721	7
ENSGALG00000000231		149,0870697	185,9234478	117,8750199	142,1973043	7
ENSGALG00000000316		33,64295455	43,6406022	24,05590723	36,87840136	7
ENSGALG00000000426		10,27991254	12,41662544	6,880846351	11,34770947	7
ENSGALG00000000674	MYO1D	4,406809187	6,316304931	2,455472407	4,256521683	7
ENSGALG00000000713	ZFHX3	39,06233202	43,14699726	29,26088997	38,05997761	7
ENSGALG00000000897		4,978147826	7,378389371	2,837088455	5,487379033	7
ENSGALG00000000899		0,963046208	5,946365074	0,475413557	2,252274664	7
ENSGALG00000001024	TPRG1L	120,9971135	152,3547079	103,4903126	132,788969	7
ENSGALG00000001100		13,95505436	17,65108414	9,356507162	15,50884815	7
ENSGALG00000001129		23,86423909	32,84739588	12,21283125	23,798218	7
ENSGALG00000001329	EPB41	65,34880758	78,02186828	51,24432241	67,40825669	7
ENSGALG00000001446		64,62473612	87,21072718	48,6331509	76,52155764	7
ENSGALG00000001449	STRA6	45,37016661	58,55812578	27,01253734	47,99719354	7
ENSGALG00000001475		3350,976054	3963,682871	2691,829167	3366,524715	7
ENSGALG00000001492	NDRG3	65,58399327	69,55303732	54,78233205	62,46719773	7
ENSGALG00000001604		52,26872952	56,56561231	40,78881554	53,29231133	7
ENSGALG00000001727	LZTS1	8,163488473	25,58923197	4,764125596	14,44205782	7
ENSGALG00000001795		10,08663077	20,53677	4,664121167	12,34777599	7
ENSGALG00000001816	MAGI3	22,47607338	29,09793529	15,71484571	21,83158076	7
ENSGALG00000001966		25,7785394	36,09230687	17,73218323	27,60168099	7
ENSGALG00000001996	DNALI1	33,7845802	40,3539152	23,78692763	32,34237044	7
ENSGALG00000002016	PRKCD	9,534583077	25,41833693	5,509761135	13,37710012	7
ENSGALG00000002055	hairy1	100,9426832	191,0596931	71,9414387	159,5868237	7
ENSGALG00000002098	GRIK3	3,884532135	6,948087025	1,414247851	2,595929492	7
ENSGALG00000002125	TMOD1	19,78933687	23,97315207	12,05769249	16,67488273	7
ENSGALG00000002169	PLEKHB2	32,6969416	49,0144176	25,10240291	39,31342986	7
ENSGALG00000002225	RFFL	28,82366062	33,4994994	22,79136152	26,46140023	7
ENSGALG00000002437	FAM69B	10,34052265	18,5207246	6,353529848	13,77821286	7
ENSGALG00000002484	CRYM	52,30050528	59,7062453	38,85736704	51,40699758	7
ENSGALG00000002610		13,09856503	27,92032271	8,551552499	20,41307335	7
ENSGALG00000002692		7,601162153	8,489273999	4,867865763	6,523571836	7
ENSGALG00000002707	CHRNB2	22,21560311	33,9138735	14,13296126	22,61297767	7
ENSGALG00000002712		98,06557273	124,0459701	60,94436313	78,26408645	7
ENSGALG00000002742	TMEM132B	21,18839394	28,46994537	12,23650943	23,57680408	7
ENSGALG00000002749		15,4477782	18,83801502	9,210681708	15,54271584	7
ENSGALG00000002777		206,4893527	235,0657741	145,3623973	217,0270845	7

ENSGALG00000002804	RND2	7,564698815	11,37308686	3,691136555	7,382372138	7
ENSGALG00000002818		76,01900762	92,05204683	46,3235432	73,16432864	7
ENSGALG00000002840	GRM4	38,07446155	45,47238554	23,80739886	33,41537259	7
ENSGALG00000002891	ACADL	101,9928444	126,3938118	85,60119687	110,7373997	7
ENSGALG00000002940		63,67353296	73,60062167	49,11114405	52,63840174	7
ENSGALG00000003099	PCTP	55,94909963	68,55104571	39,17056499	50,10135455	7
ENSGALG00000003135	ANK3	29,82803871	36,54263	22,66405296	23,3794697	7
ENSGALG00000003185	SCPEP1	39,41581989	47,08505296	26,77264651	36,20660242	7
ENSGALG00000003345		8,011075734	13,2054126	4,141316066	10,41345166	7
ENSGALG00000003445	MGST3	164,0311496	177,1818225	134,4152099	167,0547988	7
ENSGALG00000003474	MED20	110,444728	116,7026163	88,99338596	109,2480186	7
ENSGALG00000003580	MMP2	7,325674272	32,10481709	4,372959973	11,83675111	7
ENSGALG00000003588		41,80910808	61,28137773	18,99649734	38,34816043	7
ENSGALG00000003589	VTN	114,2225217	150,0745088	69,50664764	114,9001653	7
ENSGALG00000003595	SARM1	30,68252668	34,98549632	21,8893545	27,60935273	7
ENSGALG00000003648	SVIP	22,22982974	29,46021773	15,12642168	17,95720385	7
ENSGALG00000003832	TEX264	15,86809856	27,52547475	11,7554828	23,97522593	7
ENSGALG00000003999		13,72215726	16,36238679	11,0995887	12,90633719	7
ENSGALG00000004038	ADAL	25,17562849	34,56044312	19,02897515	26,71696561	7
ENSGALG00000004236	TRAF3IP1	33,67265581	37,7057156	28,80217302	29,82775189	7
ENSGALG00000004323	RASAL2	17,46458791	24,05795653	11,68718553	21,19348944	7
ENSGALG00000004448	BRINP2	7,422301548	13,10562437	2,669166415	9,017834641	7
ENSGALG00000004472	ASTN1	45,27241295	56,98089896	25,40185133	42,4550224	7
ENSGALG00000004581	NDST1	36,77466572	44,71190372	29,08485213	41,89895888	7
ENSGALG00000004593	SULF2	30,06159727	56,14934678	19,86175058	37,91786843	7
ENSGALG00000004623	AGTRAP	23,55850976	32,14710496	15,71775217	22,70328234	7
ENSGALG00000004676		44,69765223	67,37462526	26,5567982	52,68656003	7
ENSGALG00000004705	BUD31	190,1039203	210,7250422	159,5584737	195,6353874	7
ENSGALG00000004769		231,0703098	265,9633181	188,117676	223,1112578	7
ENSGALG00000004812	FAM129A	3,011515596	4,419696057	1,922761698	3,474926272	7
ENSGALG00000004844		38,76266742	46,85070174	21,59843211	33,82333789	7
ENSGALG00000004851		102,8684263	153,4847972	80,99247061	119,9569497	7
ENSGALG00000004873		21,88445189	28,72943819	17,20757599	23,79151605	7
ENSGALG00000005062	FoxN4	4,362960232	23,41120468	2,672268849	13,15759672	7
ENSGALG00000005076		2,97258771	5,738604455	1,868991974	4,403336028	7
ENSGALG00000005203	OLFM3	9,917656348	19,51400136	6,107981318	12,50996987	7
ENSGALG00000005446		433,5296312	485,9292058	357,6286444	378,096764	7
ENSGALG00000005502	KCNG4	0,912952006	1,203243361	0,297105278	0,60110466	7
ENSGALG00000005539		10,06496779	11,46804537	4,811243877	8,97883854	7
ENSGALG00000005549	CUEDC1	24,9585639	31,73680379	17,96776786	28,07856242	7
ENSGALG00000005626	TPST2	11,80433122	16,81885809	6,363075005	13,39419824	7
ENSGALG00000005657	CRHR2	16,41937448	21,14097462	9,617236635	17,9916876	7
ENSGALG00000005901	PCMTD2	15,03826855	22,94366732	12,20442379	18,1485811	7
ENSGALG00000005967		16,34607915	27,95004368	5,734444886	19,79268951	7
ENSGALG00000006014		11,83239403	17,79556194	8,524616365	16,16215962	7
ENSGALG00000006036		51,78288032	72,39218737	37,65758569	61,49569637	7
ENSGALG00000006083		4,451611687	9,146359521	3,028486674	6,106851928	7
ENSGALG00000006172	ABCC8	10,46414413	12,87547866	7,792903566	8,971268996	7
ENSGALG00000006192		6,56151079	13,70633898	3,575297057	7,644373335	7
ENSGALG00000006233		73,88654127	86,5339275	62,67969355	65,81661353	7
ENSGALG00000006490	SCN3B	48,56309698	61,96270518	30,47526127	36,08774643	7
ENSGALG00000006588	LRRC75B	20,28960188	26,05813662	15,10431218	21,90900245	7
ENSGALG00000006606		6,809091308	32,17770953	5,138243105	16,8368564	7
ENSGALG00000006780		245,3841891	304,5147127	196,4819313	254,7782702	7
ENSGALG00000006792	VSTM2L	14,91896564	24,34060472	8,041765026	19,66450542	7
ENSGALG00000006857	SPATA20	13,000609	15,94295579	10,59989797	14,33927151	7
ENSGALG00000006873	PHRF1	51,794473	56,56115769	28,13525923	43,64214396	7
ENSGALG00000006882	ST8SIA2	43,06730195	67,38681182	26,22541309	54,35238193	7
ENSGALG00000006942		132,4198867	227,8606087	86,86056301	125,7845777	7
ENSGALG00000007076	CCNY	18,51574898	24,71732273	11,69363679	18,88976684	7
ENSGALG00000007078		2,87252386	4,383770523	1,005692341	3,383060819	7
ENSGALG00000007128	SH3BGRL	214,2530874	229,1837125	167,2589166	211,0428624	7

ENSGALG00000007211	CDH22	1,485142793	2,494244579	0,548146547	1,816562264	7
ENSGALG00000007262	SPG21	71,40763929	85,58144568	58,23479154	64,58167991	7
ENSGALG00000007732	RTN4R	3,699735966	6,809590742	2,178228147	5,983400116	7
ENSGALG00000007748	SLC16A2	20,32729004	25,80043621	12,93321002	22,99796604	7
ENSGALG00000007780	EIF4E3	18,03876093	38,22663339	13,64586903	30,5542396	7
ENSGALG00000007908		5,792965987	13,97554053	2,389386077	7,707065248	7
ENSGALG00000007943		19,29727515	23,99833693	15,17755759	15,86135712	7
ENSGALG00000007965	HN1	411,0251371	434,100802	329,5995071	408,922843	7
ENSGALG00000008438	SLC35C1	124,3184986	149,9093654	98,43575195	132,7600199	7
ENSGALG00000008455		32,31963069	57,54084189	14,64828513	25,24367492	7
ENSGALG00000008697	RBM20	3,846768505	4,926267748	2,456023592	3,647659329	7
ENSGALG00000008700	PDCD4	325,6337309	399,5164392	238,9464322	293,0805554	7
ENSGALG00000008728	PTER	6,3399657	8,156910559	4,337055382	7,10541263	7
ENSGALG00000008755		67,34340554	73,50049392	52,70850781	64,66558339	7
ENSGALG00000008818	IDH1	71,66766176	91,61503146	53,6340664	74,86640267	7
ENSGALG00000008865	ALAD	139,8444823	169,0120545	111,5900089	141,221191	7
ENSGALG00000009002		1,28675173	1,674918274	0,613968423	1,412361787	7
ENSGALG00000009042	ISM1	19,65944442	27,17765292	9,838603957	17,08408347	7
ENSGALG00000009052	LBH	37,94812104	96,15335335	19,71154839	42,02747293	7
ENSGALG00000009134	CTTNBP2	11,40771871	15,88432695	8,530144038	10,85876386	7
ENSGALG00000009212	MND1	66,79405001	75,25435741	49,15985084	63,46334427	7
ENSGALG00000009354		11,65567463	14,51617495	8,805310484	9,634955967	7
ENSGALG00000009439	GPR85	17,44009003	22,44330748	10,32374078	18,89710187	7
ENSGALG00000009478		12,99711452	17,0630503	6,327520434	10,68724831	7
ENSGALG00000009536		8,930747832	11,26382701	4,868019414	9,734234384	7
ENSGALG00000009537	PPM1L	15,19652092	19,19973765	9,532288845	11,64296507	7
ENSGALG00000009601	NELL2	110,2961344	154,2484566	51,22048418	99,21561222	7
ENSGALG00000009603		11,49975548	14,99438031	4,75072532	9,623024967	7
ENSGALG00000009670	SGCE	59,43794591	65,07796911	46,83087493	55,09067955	7
ENSGALG00000009959		35,62728541	41,80367214	20,63974566	27,91189617	7
ENSGALG00000009977	SMAD1	78,48081668	94,47814686	67,52132303	77,34497377	7
ENSGALG00000010119	CDKL2	8,919919874	17,83271101	5,399685531	10,94164418	7
ENSGALG00000010127	GLO1	231,8522951	279,4946021	191,8389983	204,8112253	7
ENSGALG00000010137	TTC6	1,287993124	1,948479866	0,779674705	0,991764128	7
ENSGALG00000010139	SSTR1	7,978023763	12,61992979	4,912549304	10,41199792	7
ENSGALG00000010269	KCNAB1	10,86190361	16,59727193	6,653570316	13,2184581	7
ENSGALG00000010320	PAWR	34,60903172	62,08502203	21,78625211	37,50489825	7
ENSGALG00000010422		103,3041586	158,2231145	73,72776386	121,1270632	7
ENSGALG00000010441		56,2447908	64,77433617	43,50838618	56,84118443	7
ENSGALG00000010444		30,2192376	32,99747957	23,77039516	26,95022033	7
ENSGALG00000010533	ELAVL4	101,3698774	123,7671942	80,28112842	100,1644983	7
ENSGALG00000010611	CFAP97	27,26735677	31,60703776	21,00245601	25,6223608	7
ENSGALG00000010797		2,204962564	3,597171829	1,242295373	2,737107629	7
ENSGALG00000010982		34,9067668	47,34640946	23,85218963	36,57797015	7
ENSGALG00000010997	DFNA5	30,98330411	36,05207494	25,73539441	33,02230238	7
ENSGALG00000011078		29,00849301	32,4201808	22,47842078	27,65933043	7
ENSGALG00000011176	SCRN1	73,33672636	113,3873336	57,54804702	76,21746157	7
ENSGALG00000011200	THBS2	4,729378665	17,96316127	2,101963158	7,934820548	7
ENSGALG00000011238	WLS	398,6085265	452,0423133	298,9173487	386,8213626	7
ENSGALG00000011276	BTG1	502,7455821	558,6321082	412,3661019	452,7358963	7
ENSGALG00000011350	NEGR1	20,33143431	23,86084881	12,71729034	19,59581703	7
ENSGALG00000011444	MFHAS1	58,17054063	70,52843469	40,96561842	49,56226035	7
ENSGALG00000011655	IQCB1	23,64106498	26,10604734	13,7260757	19,72872665	7
ENSGALG00000011656		0,973892515	2,15913225	0,52062462	0,800954632	7
ENSGALG00000011709		10,59850516	15,02453029	4,939474354	10,42674333	7
ENSGALG00000011778		23,66787702	28,8235186	17,91116836	26,20801542	7
ENSGALG00000011798	HEBP1	12,49320521	25,56914364	7,850759982	11,62061774	7
ENSGALG00000011810		65,9521746	82,87507381	51,45272236	58,71022908	7
ENSGALG00000012044		9,229584329	12,71447637	7,5350979	9,063435059	7
ENSGALG00000012057		11,18597673	12,61517469	8,164876186	10,80367383	7
ENSGALG00000012092	ESYT3	4,680130309	6,111516607	2,253806553	3,990397078	7
ENSGALG00000012116		9,646935596	13,32089976	6,14467466	11,39954133	7

ENSGALG00000012185	PLA2G12A	102,7974734	112,7403772	84,43323048	105,0643144	7
ENSGALG00000012191		32,82020057	46,37361014	18,84566996	32,6990559	7
ENSGALG00000012362	THSD7B	3,775092996	10,36024079	2,69688283	7,22396186	7
ENSGALG00000012377	HNMT	1,717135166	6,060908343	0,573693554	1,878896194	7
ENSGALG00000012487		24,24348782	31,4734152	16,11839467	23,96854961	7
ENSGALG00000012652		10,7529658	25,54973014	5,663246629	17,81444342	7
ENSGALG00000012658	TCP11L2	15,92414007	33,05220242	11,27908373	28,17571503	7
ENSGALG00000012692	SIRT5	45,36256371	63,38567531	35,0481337	44,42038027	7
ENSGALG00000012714		43,94791511	52,13807433	33,7764496	43,47609852	7
ENSGALG00000012715	CAP2	8,59191649	11,46991422	4,148180559	9,301130175	7
ENSGALG00000012791	TBXAS1	1,864983737	3,103081301	0,74879117	2,141225714	7
ENSGALG00000013002	DAP1	32,2061213	57,91577834	24,03389605	42,05183833	7
ENSGALG00000013067		137,5096802	191,3369487	110,2279785	152,0190566	7
ENSGALG00000013837		59,04391774	71,3460663	48,21803842	49,19810217	7
ENSGALG00000013876		202,1069193	227,9456838	159,7823013	213,2983315	7
ENSGALG00000013964	SPATA18	5,231802216	6,450354247	3,531114535	5,627475195	7
ENSGALG00000013980	SLC2A12	1,779452239	4,860254934	0,730603455	2,144183869	7
ENSGALG00000014004		38,19788449	43,26576373	28,71001642	36,16158832	7
ENSGALG00000014233	FBLN1	100,2616639	153,9996924	85,13157946	116,6781323	7
ENSGALG00000014242		24,47453838	32,12228945	16,30724171	25,76182082	7
ENSGALG00000014258	LIMCH1	11,23039493	16,58171204	4,316562472	9,590761217	7
ENSGALG00000014300		2,585478889	4,334338666	1,496149583	2,982896803	7
ENSGALG00000014496	Prom1	38,69686144	62,3200769	30,55765133	41,6577711	7
ENSGALG00000014537	BMF	66,59173617	87,81261663	47,00373868	44,31366941	7
ENSGALG00000014661	FAM172A	53,62830077	59,84785505	44,237488	54,90827811	7
ENSGALG00000014662	RBP5	94,12229359	110,0266074	71,71186194	92,49555572	7
ENSGALG00000014699	MEGF10	5,113117701	7,375190092	1,931874889	3,210163737	7
ENSGALG00000014812	SIDT1	6,795260519	16,41334784	4,233919069	10,62192737	7
ENSGALG00000014833	RPL37	1596,687135	1720,735968	1387,064356	1578,22352	7
ENSGALG00000014844		19,41962485	24,18777381	13,76691142	17,52298328	7
ENSGALG00000014941		4,499019336	9,516121976	2,714813327	8,305086794	7
ENSGALG00000015075		14,78424772	16,24793751	7,992995674	13,23798293	7
ENSGALG00000015183	RAB13	27,51711853	44,6852246	16,72602217	36,46505352	7
ENSGALG00000015297	FOXO3	24,37599027	33,24133814	13,94064542	26,23468786	7
ENSGALG00000015537	MAN2B2	30,3066011	45,5128118	21,78564425	29,42547877	7
ENSGALG00000015543	MRPL50	97,51826513	108,3654216	76,17159113	102,4663452	7
ENSGALG00000015551	GRIN3A	5,679152207	17,73134218	3,841307699	16,43553314	7
ENSGALG00000015660	SNX30	42,56777807	79,83420568	30,02031755	56,72491395	7
ENSGALG00000015762	LYRM2	37,69692925	47,81440333	26,81605255	32,66012008	7
ENSGALG00000015768		28,37209383	38,55451722	16,41325642	31,2891459	7
ENSGALG00000015774	CYYR1	73,07270964	93,2325043	46,68077123	68,44045186	7
ENSGALG00000015780	PNRC1	344,5438061	552,0456497	280,6093647	398,9770095	7
ENSGALG00000015903		11,13980945	17,28591052	7,220689109	10,09139434	7
ENSGALG00000015967	LRRTM1	3,686312959	5,438787671	1,609278461	3,628427823	7
ENSGALG00000015987		23,3708759	36,39569956	15,7535009	22,36869225	7
ENSGALG00000016223		3,343218403	5,947487934	1,855740174	4,018942369	7
ENSGALG00000016251		93,77894807	167,1461162	59,86796921	128,2348016	7
ENSGALG00000016289	DST	15,90950987	19,20924281	11,20078851	13,75441035	7
ENSGALG00000016322		31,14927638	36,17694833	22,78652856	32,10595086	7
ENSGALG00000016463	FAM49A	89,87725864	97,84634246	78,24594873	86,88895017	7
ENSGALG00000016518	PHKA2	22,70891315	25,28803726	16,10212794	20,50180035	7
ENSGALG00000016543	NHS	23,2035252	27,27258538	13,39269629	22,59847883	7
ENSGALG00000016549	CTPS2	7,332248006	10,148353	5,828515996	8,094284	7
ENSGALG00000016551	AP1S2	150,331608	388,8047531	100,7222479	269,0706899	7
ENSGALG00000016697	MUT	33,31728166	36,06625525	27,10395955	31,40871266	7
ENSGALG00000016870	TMTC4	37,55391684	53,03696736	30,09752082	43,11923341	7
ENSGALG00000016945	LECT1	36,93378975	51,37890081	22,55103978	46,08609363	7
ENSGALG00000017036	LHFP	57,15964101	140,0778702	35,52548196	97,5065896	7
ENSGALG00000017072	N4BP2L1	13,74550565	22,46637191	10,03427041	19,42210549	7
ENSGALG00000017106	ATP8A2	4,502878382	10,93890475	2,617722948	6,161186837	7
ENSGALG00000017219	C11orf54	30,51387908	33,47914202	23,10946363	29,3091597	7
ENSGALG00000017321	PLEKHB1	21,68327263	24,29861118	15,96522023	20,23831437	7

ENSGALG00000017326		51,59859703	55,00738274	41,05442188	47,2276208	7
ENSGALG00000017329	FCHSD2	14,45697367	17,25365137	11,28085158	16,13712426	7
ENSGALG00000017331		3,159907146	5,517577508	1,862654243	4,660927839	7
ENSGALG00000017332	KLHL35	12,22287679	16,1880175	5,959667234	8,934592728	7
ENSGALG00000017334		8,363048271	10,26995253	6,118966477	8,105085971	7
ENSGALG00000017342		3,582341309	4,647630554	1,063461596	2,894726481	7
ENSGALG00000017374		7,866867941	11,57898563	3,96740595	10,0765853	7
ENSGALG00000017926	7SK	13,43570614	17,43926497	5,12370302	13,13564085	7
ENSGALG00000018624		211,612459	247,6923528	121,693404	178,5967543	7
ENSGALG00000019280		31,88812177	75,53173467	15,04830691	37,69512136	7
ENSGALG00000020376		138,5609605	183,9703067	71,81940611	129,9222041	7
ENSGALG00000020528	INIP	136,2608495	170,1147196	109,1941668	152,6630137	7
ENSGALG00000020626		21,17069503	23,62411008	14,87384185	20,507544	7
ENSGALG00000021021	ARL4C	275,3523921	337,3144485	220,9487353	242,8119461	7
ENSGALG00000021344		9,857224916	10,97152481	7,024591987	9,213278791	7
ENSGALG00000021972		101,8915641	119,2500243	83,48016628	106,4157224	7
ENSGALG00000022713		139,0657773	202,1677216	94,76044509	146,646949	7
ENSGALG00000022718		6,91125738	10,33059383	3,599725653	8,616716382	7
ENSGALG00000022838		6,670263015	8,568090621	4,322962094	6,400590802	7
ENSGALG00000022896	IRX4	0,349425233	0,593038773	0,239827947	0,525323997	7
ENSGALG00000023042		11,1327311	13,31359085	7,176717915	11,03847192	7
ENSGALG00000023131	PDXP	56,87831586	59,99147542	45,64640553	52,21241518	7
ENSGALG00000023189	UMAD1	64,4654313	78,99153297	45,33959426	68,96410803	7
ENSGALG00000023621	SFXN3	7,518241748	11,9378823	4,437892273	10,8074635	7
ENSGALG00000023655		27,08945009	36,20494428	18,57596443	31,99177494	7
ENSGALG00000023738		48,2607002	63,37886353	23,3954474	42,14401685	7
ENSGALG00000023751		2,121704326	39,53753646	8,474198433	21,85058761	7
ENSGALG00000023812		15,06664138	19,18104403	10,25652845	15,75468054	7
ENSGALG00000023881	PLXDC1	4,82633085	5,58847381	2,844462158	4,814461859	7
ENSGALG00000025163	gga-mir-1627	110,9384893	123,3023999	64,38404565	94,51602649	7
ENSGALG00000025698	5_8S_rRNA	214,2147206	274,3953045	109,3746589	240,8884729	7
ENSGALG00000025818		34,66056389	52,36778343	27,68112335	42,84794983	7
ENSGALG00000025977	GADD45A	65,82047393	81,42861239	46,15966381	67,89681053	7
ENSGALG00000026042		16,59308302	28,8879479	8,277397044	18,77618411	7
ENSGALG00000026103		9,300012163	12,28619669	3,508789346	8,103143537	7
ENSGALG00000026113		55,31332896	69,44178259	41,36543103	62,22853953	7
ENSGALG00000026180		43,38860094	74,08650761	27,9624619	44,23281888	7
ENSGALG00000026437		53,32978665	106,3553563	42,99932252	73,23714682	7
ENSGALG00000026505		6,840702248	11,78765652	4,728224671	8,990305294	7
ENSGALG00000026600	C1orf198	62,51064096	97,06115423	52,49014482	69,67854538	7
ENSGALG00000026870		22,81793746	48,40836989	17,04944091	35,73639395	7
ENSGALG00000027027		12,17687155	19,36749454	8,327209902	16,49890464	7
ENSGALG00000027058	EMP2	9,670752435	14,47252177	6,676394562	11,60111876	7
ENSGALG00000027173	TMEM159	101,3603219	133,3366653	66,720424	98,09898988	7
ENSGALG00000027337		35,30559561	46,3009554	28,71685084	40,66049371	7
ENSGALG00000027351	LRRC71	12,41099266	13,81465571	7,63665904	11,62692947	7
ENSGALG00000027377		13,23118924	17,08047841	8,393624566	12,22092941	7
ENSGALG00000027418	HINT2	54,35709786	65,97192429	37,50431959	54,53621881	7
ENSGALG00000027511		94,02464102	203,238416	68,70151952	146,2623945	7
ENSGALG00000027534		22,63698	42,16451109	16,01154358	30,54121339	7
ENSGALG00000027695	PCBP4	162,956744	178,0507957	132,4616368	163,8638337	7
ENSGALG00000027778		0,760117162	7,843427702	2,238665183	3,422613312	7
ENSGALG00000027793	SCN9A	2,260801542	2,687945527	1,301123732	1,749485357	7
ENSGALG00000027904		130,5879679	138,1008328	105,1857775	127,8946055	7
ENSGALG00000027920		8,224921324	11,67541101	4,941372418	10,16266736	7
ENSGALG00000028031	C1QL1	3,855114072	6,583157269	1,783653583	5,460140979	7
ENSGALG00000028187	FUNDC1	50,26900328	65,59407968	42,88306331	53,15622678	7
ENSGALG00000028234		4,065747386	5,588221597	1,848844705	4,176917872	7
ENSGALG00000028281		0,539773136	0,627847197	0,388756816	0,499963427	7
ENSGALG00000028284	PTX3	1,360269657	1,613746408	0,640931131	1,214844992	7
ENSGALG00000028371		1205,986207	2113,34695	600,0776823	1309,782147	7
ENSGALG00000028443		106,3466323	117,1726174	52,65285501	90,77932913	7

ENSGALG00000028567	MYL9	25,71491939	30,84447999	19,34651722	28,9008983	7
ENSGALG00000028605		33,48063752	44,87000567	24,5313162	38,26157979	7
ENSGALG00000028635		36,25602462	40,97336372	25,19241388	34,21441251	7
ENSGALG00000028946		2,681879198	4,752514748	1,279230013	3,51800798	7
ENSGALG00000029037		150,1495751	180,3499614	124,9709847	155,381485	7
ENSGALG00000029102	PXYLP1	43,95109752	57,83768431	29,96371923	35,84248398	7
ENSGALG00000029150	AK1	74,20126426	117,6906446	51,63919399	106,9208251	7
ENSGALG00000000402	LOXL2	5,954483122	5,824945864	3,968242539	6,612512106	8
ENSGALG00000000433		1272,018653	1440,431589	928,8310457	1560,586316	8
ENSGALG00000000921		20,02878103	21,81551147	15,53568376	22,32711039	8
ENSGALG00000000966		20,47256091	19,52267998	15,14294559	19,21668586	8
ENSGALG00000000970		121,5454735	107,7587292	88,7857163	113,9503321	8
ENSGALG00000001026		36,75836285	36,46182397	30,03854447	37,77669558	8
ENSGALG00000001040		2,135610314	2,701891274	1,274424793	3,016335863	8
ENSGALG00000001096		305,8554674	349,0409519	253,9138438	350,2592113	8
ENSGALG00000001144	ZNF346	38,22164066	33,87809183	30,12408592	37,38262219	8
ENSGALG00000001191	MEOX1	7,04229087	7,660896452	4,783886826	10,65337693	8
ENSGALG00000001342	HCN2	3,566827073	3,913196094	2,003233469	3,567430691	8
ENSGALG00000001668		65,31424271	61,00669811	56,15496715	68,17862012	8
ENSGALG00000001879		1,337810461	2,692005897	0,827824211	4,113696738	8
ENSGALG00000002308	COMMD10	64,65931335	63,73727871	52,43554159	65,08224	8
ENSGALG00000002451		7,892336133	8,433004452	5,368002869	9,378353576	8
ENSGALG00000002832	IFI35	13,41565324	19,92428156	5,840906287	20,84894169	8
ENSGALG00000003282	STAT5B	43,47011723	59,21868599	36,01354452	61,71799679	8
ENSGALG00000003404		16,38506125	22,47425288	10,42923248	26,49809005	8
ENSGALG00000003457	CNP	27,91472575	24,76733122	21,15447525	35,31883756	8
ENSGALG00000004309	GDAP1L1	24,07708294	28,27887319	14,31439188	29,41749102	8
ENSGALG00000004518		5,456576175	5,358687351	3,636599085	5,328686101	8
ENSGALG00000004537		180,6757873	161,8033161	121,9790746	175,016988	8
ENSGALG00000004725		82,94245202	95,93644244	69,23109947	97,51285509	8
ENSGALG00000004741	EPHB2	47,80916319	49,45118607	31,7185343	48,00633611	8
ENSGALG00000004775		9,40967762	9,784964463	6,248181276	9,515999548	8
ENSGALG00000004875	PEMT	36,58111755	49,2637599	28,18151635	49,03587614	8
ENSGALG00000004902	TOM1L2	47,90888274	54,14283223	38,4291727	56,34056348	8
ENSGALG00000004936	GLRA4	0,79751784	0,927900085	0,1832244	1,510310992	8
ENSGALG00000005297	HHATL	3,387077303	2,668515935	1,79964287	6,359255378	8
ENSGALG00000005330	SNCAIP	8,488103316	16,17894196	6,249057345	16,83568789	8
ENSGALG00000005628	COL9A3	17,96213617	15,94826923	10,69613986	17,40578323	8
ENSGALG00000005751		5,36854481	5,781142754	3,794119758	5,564424762	8
ENSGALG00000005820	RNF130	115,9755276	132,7019958	96,44159114	135,6127204	8
ENSGALG00000005930	PLS3	207,3820465	213,2667153	176,3834995	205,374161	8
ENSGALG00000006152		21,62598292	24,37801658	13,1869189	22,40566092	8
ENSGALG00000006245		7,594383946	6,44434218	3,155446769	11,38846586	8
ENSGALG00000006346	CXCL14	20,0059794	16,52261414	9,684803529	17,74246896	8
ENSGALG00000006453		23,05683828	28,21009978	15,71140953	28,93303717	8
ENSGALG00000006480	TCF7	40,96204917	40,44824339	28,05334558	52,25914443	8
ENSGALG00000006507	CLMP	11,08247747	10,29291948	5,480177048	10,05172185	8
ENSGALG00000006702	MFGE8	21,72913025	18,25785553	16,69202306	24,14199294	8
ENSGALG00000006793		4,520154861	4,229306859	2,448533311	5,022349685	8
ENSGALG00000006885		193,6654886	228,2465968	140,0387935	217,4857151	8
ENSGALG00000007388		3,861549661	3,783674214	1,646524718	3,861815162	8
ENSGALG00000007447	TUBB1	7,115252041	6,858661343	4,858476059	6,983463694	8
ENSGALG00000007623	CACNA1G	9,626950958	10,78838576	5,504792009	10,1006864	8
ENSGALG00000007981	CLN6	46,70691407	61,89784382	34,14706975	59,56872405	8
ENSGALG00000008188	TRIM36	1,365256301	2,320330846	0,507058176	2,908026625	8
ENSGALG00000008250		2,334382918	2,222336548	1,211757734	2,093247638	8
ENSGALG00000008349	LMCD1	6,381606237	5,950498424	3,175987053	6,800362349	8
ENSGALG00000008367	MDK	538,002912	538,5537481	348,2539918	523,7018112	8
ENSGALG00000008433		49,98160633	63,0842024	33,53067368	59,10964698	8
ENSGALG00000008440	CHST1	6,557087603	7,782559043	2,575428704	8,796610773	8
ENSGALG00000008479	PLEKHM3	7,425884037	8,009813156	4,951758076	8,469638599	8
ENSGALG00000008888		2,717591914	3,726185576	1,209465832	4,278582904	8

ENSGALG00000008953	AASS	2,104289207	3,485597766	1,298976223	3,507044234	8
ENSGALG00000009060		55,52198203	50,99564319	43,7247881	55,32060319	8
ENSGALG00000009237		3,961438611	0,772111634	1,60979255	4,327297542	8
ENSGALG00000009638	ANO6	1,715961455	1,740638076	0,68045043	1,500474705	8
ENSGALG00000010083	ST3GAL3	50,26691798	46,55220626	40,90820647	47,83758969	8
ENSGALG00000010316	FRAS1	14,37701744	14,44809891	10,14312586	16,86422595	8
ENSGALG00000010500	NKX6-2	82,02997841	72,56241153	39,76739828	74,26592223	8
ENSGALG00000010551	CDC42EP3	32,94479902	38,50554976	20,60412631	41,86968279	8
ENSGALG00000010706		15,94984957	17,60812199	13,24497666	20,76705437	8
ENSGALG00000010750		90,15991016	92,3598817	69,55665579	93,56553739	8
ENSGALG00000010811	LGMN	104,6799372	108,867442	72,91205227	103,5052655	8
ENSGALG00000011324		645,0588375	753,4902301	447,1398409	797,1195948	8
ENSGALG00000011409	SNRPF	537,0729519	537,2486928	446,0636939	526,1919388	8
ENSGALG00000011951		4348,85487	4582,468074	3827,371123	4750,322822	8
ENSGALG00000012165	DLGAP5	180,1309956	166,0315001	151,8503163	181,618057	8
ENSGALG00000012546	MCM5	377,5873693	349,3150659	303,0517423	394,3894071	8
ENSGALG00000012577	FRMD3	8,135128562	10,76858863	5,124147526	10,54811856	8
ENSGALG00000012586	GKAP1	49,38065987	48,31048476	35,18915075	46,61458231	8
ENSGALG00000012644		118,2208301	107,161874	82,58924356	124,3352882	8
ENSGALG00000012710	NCS1	53,38605119	59,47857108	35,31604687	59,30108349	8
ENSGALG00000012712	RBM24	11,69804863	12,65506283	7,511658153	13,38509159	8
ENSGALG00000012792	HIPK2	34,10544651	47,51606445	28,0810563	48,57650721	8
ENSGALG00000013370		19,96508379	17,82198179	14,85078035	19,24758448	8
ENSGALG00000013784	CDT1	165,2005614	151,8783838	132,5244372	174,9558592	8
ENSGALG00000013848	MVK	80,28802642	88,74775882	66,89244986	85,71101038	8
ENSGALG00000014408	LLGL1	136,7318583	150,9541375	116,2058785	148,1708454	8
ENSGALG00000014818	TRIM45	13,13116048	11,86036513	9,402249388	13,7018197	8
ENSGALG00000014910	COLEC12	36,98106275	49,28334328	26,80255957	46,75071046	8
ENSGALG00000014970	FSTL1	400,311328	408,6417616	314,2104498	430,8721221	8
ENSGALG00000015082	RPS6	3517,307527	3686,176035	3027,546214	3739,458637	8
ENSGALG00000015084	IGSF11	7,264231584	9,578005382	3,591960257	11,29906875	8
ENSGALG00000015107	PIP5K1B	18,92175169	18,57005395	13,47044353	20,52336847	8
ENSGALG00000015186		14,74535991	21,55944147	7,489188851	20,8815943	8
ENSGALG00000015326	CLTA	267,2978304	294,1857064	219,3695547	329,8482612	8
ENSGALG00000015372	ATP5I	403,7583558	509,6131573	333,1327727	501,0696761	8
ENSGALG00000015379	TAGLN3	87,81368477	100,9944499	62,06751842	113,9094292	8
ENSGALG00000015469		20,37996053	36,86257283	15,57629849	36,01578911	8
ENSGALG00000015597		5,227904206	3,833284117	3,037151249	4,594564421	8
ENSGALG00000015617	RPS23	2617,899457	2862,618867	2286,280255	2782,266287	8
ENSGALG00000015663	HSDL2	133,5386111	139,1086821	101,7937975	136,1974338	8
ENSGALG00000015729	LPAR1	21,41474649	23,35821948	13,55389449	24,06974178	8
ENSGALG00000015895	HTR1B	25,76646903	23,82650311	19,0394431	23,92584621	8
ENSGALG00000015926		5,567947829	7,134824852	3,268581878	6,673131012	8
ENSGALG00000016152		183,9160675	192,1318333	138,2517808	212,5560354	8
ENSGALG00000016328		83,28263556	147,5069832	61,86336813	143,313037	8
ENSGALG00000016665	FDFT1	96,95097122	82,48027441	75,2834769	99,1418902	8
ENSGALG00000016692		85,88865234	83,54926437	67,04372699	83,5908187	8
ENSGALG00000021174		57,3355086	57,82250149	43,91995666	55,54606164	8
ENSGALG00000021365	DCTN3	212,9718058	222,1596474	170,2155642	239,7819731	8
ENSGALG00000022926	GPR162	43,58694006	47,89104575	31,47238754	49,14485034	8
ENSGALG00000023435	GATM	10,90557171	11,56749101	7,424217313	11,9559467	8
ENSGALG00000023577		3,448939012	3,331537676	0,899477294	6,243389678	8
ENSGALG00000023626	NTN1	24,02953412	32,21432394	16,58916643	33,96160684	8
ENSGALG00000023689	ASS1	1,249320069	1,92940501	0,417616289	1,950364347	8
ENSGALG00000023801		3,651253916	2,716726028	0,815991185	2,845431632	8
ENSGALG00000023936		14,3827097	16,65315064	9,644678815	19,86677939	8
ENSGALG00000025764	FKBP10	34,79818805	41,73571023	28,05073896	46,92463723	8
ENSGALG00000025919		20,97538621	16,70085792	12,13546828	23,20345206	8
ENSGALG00000026081	RBM38	44,21346634	48,37930983	34,01608481	53,55787387	8
ENSGALG00000026203	FAM174A	27,05573691	28,32165205	20,54054072	27,04979678	8
ENSGALG00000026291		44,51892603	46,84798811	36,7820635	50,21922775	8
ENSGALG00000026300		417,310051	435,3420646	350,8924467	463,9459697	8

ENSGALG00000026449		234,0396809	311,3452264	183,6563344	320,9495089	8
ENSGALG00000026517	RFC5	156,0885731	158,5361803	126,8196552	169,2035955	8
ENSGALG00000026919		2,441650333	2,953248286	1,625810597	2,961202745	8
ENSGALG00000027462	P3H4	17,15905149	24,46370473	12,9024833	25,92857528	8
ENSGALG00000027501	ANPEP	12,01379711	10,03763968	6,221923555	14,47536557	8
ENSGALG00000027710		118,024832	134,7918283	96,52690233	134,4618088	8
ENSGALG00000027824		76,49717615	91,19420938	59,38902312	87,936403	8
ENSGALG00000027917		29,58120555	28,64651933	24,83745695	28,98249309	8
ENSGALG00000027963	COX7C	177,2982167	215,1096727	146,7488079	208,9018198	8
ENSGALG00000028470		29,51653543	32,02852181	23,47533065	32,21145656	8
ENSGALG00000028543	FGFR4	15,68663076	14,79137651	9,453274085	15,46542694	8
ENSGALG00000028682	CBLN2	14,71375594	13,14190177	4,554452616	12,43147608	8
ENSGALG00000028716		67,55341979	61,61072026	54,87200105	66,7873307	8
ENSGALG00000028795		18,82719941	21,71314579	12,37729026	28,90667954	8
ENSGALG00000029019		0,071552757	0,02569526	0,013204207	0,059434845	8
ENSGALG00000029174		27,89925869	54,48511253	20,36078384	65,69049819	8
ENSGALG00000000044	WFIKKN1	31,25400692	17,05051776	18,3375194	16,23160749	9
ENSGALG00000000161	ISOC1	46,60655276	39,59532572	35,69279471	37,31603953	9
ENSGALG00000000528		57,8074608	48,44024498	44,75009669	37,2413949	9
ENSGALG00000000587		10,87385763	7,033016405	7,125083511	6,717576626	9
ENSGALG00000000619	ANGPTL4	19,0867957	11,9712835	14,11823462	10,50099385	9
ENSGALG00000000623		123,5225802	85,31962828	80,7882465	78,48784073	9
ENSGALG00000000628		30,5489741	20,87801046	22,15847088	20,43363063	9
ENSGALG00000000644		27,34191038	15,24672665	17,77134235	13,4679886	9
ENSGALG00000000681	PAK1	19,8427259	17,46535245	16,20668324	17,09279966	9
ENSGALG00000000684	KLHDC8A	52,21088274	37,62714869	41,16059659	28,61108362	9
ENSGALG00000000690	GDPD4	108,6457182	52,11130181	64,04614044	41,17074748	9
ENSGALG00000000750		5,480945592	4,028561748	3,354812383	4,175923743	9
ENSGALG00000000780		100,949447	93,14132877	82,50393208	63,461697	9
ENSGALG00000000927	ANGPTL2	12,96678898	9,307183466	8,306381085	6,636659284	9
ENSGALG00000000939		88,7332569	65,62779965	64,03714459	58,49544371	9
ENSGALG00000001063		9,161939454	6,091395269	4,295525218	4,422690334	9
ENSGALG00000001141	HES5	1418,60207	1112,884661	1038,241962	898,373404	9
ENSGALG00000001153	AUTS2	115,680856	92,03951073	73,84336585	81,27424337	9
ENSGALG00000001516		36,66443788	25,78277742	22,91121743	22,30922256	9
ENSGALG00000001733	LRIG2	69,99915275	58,54048669	55,02885328	46,10602716	9
ENSGALG00000001774	NEO1	144,6407555	114,9123423	104,6053004	85,78682404	9
ENSGALG00000001954	APTX	80,10762546	60,11975832	63,03128633	55,10270093	9
ENSGALG00000002090	GPSM2	133,1906718	117,2282647	113,4776413	103,8677502	9
ENSGALG00000002091	ZDHHC1	26,3461381	22,81614683	19,80544615	19,55804929	9
ENSGALG00000002228		44,10956358	26,8456593	23,99308478	24,50200802	9
ENSGALG00000002249	AGO1	128,6081271	93,61556828	98,265346	85,67338208	9
ENSGALG00000002268		57,88933765	48,13784859	48,5939597	43,31254971	9
ENSGALG00000002298	COL8A2	33,36976189	19,29568543	15,36725962	21,08774073	9
ENSGALG00000002317		28,69380943	24,97653503	23,05559118	21,56051721	9
ENSGALG00000002375	NOTCH1	137,3911419	112,1394882	91,48126487	99,84782244	9
ENSGALG00000002599	ARHGEF39	41,36660965	30,08437158	31,67263412	32,09717563	9
ENSGALG00000002652		215,5577691	158,3864206	138,1374797	98,78094971	9
ENSGALG00000002774	TSPAN3	599,9764973	447,3646823	467,922637	392,2362777	9
ENSGALG00000002831	SH3PXD2B	43,1122237	31,76322262	32,27401658	27,94680373	9
ENSGALG00000002974	SPHKAP	6,235148986	3,427073538	3,961823218	2,770629109	9
ENSGALG00000003085	CDK1	835,642788	711,6170086	717,3170313	734,33598	9
ENSGALG00000003163	GNAO1	161,9917225	108,4121059	105,2394616	85,1330615	9
ENSGALG00000003320		2,681105339	1,925580202	1,577834384	1,719761201	9
ENSGALG00000003424	LMX1A	14,62572453	6,771825817	7,698712962	5,993144908	9
ENSGALG00000003426	RHBDL3	84,52214462	44,61134271	44,5094291	29,9201131	9
ENSGALG00000003429	PBX1	90,75741527	72,39295828	72,36913039	65,41501567	9
ENSGALG00000003434	C17orf75	116,8784541	77,66935733	68,15217644	55,35753847	9
ENSGALG00000003503	MYBL2	103,3422234	89,91141222	86,47418819	88,49615621	9
ENSGALG00000003551		16,08587485	9,180770702	7,561269976	10,02134429	9
ENSGALG00000003554	OPTC	119,1239593	71,84161518	56,89099392	54,86320698	9
ENSGALG00000003582	SLC1A3	84,64090633	32,60614203	53,95299351	22,44357991	9

ENSGALG00000003601	ATP2B4	84,10436696	68,83661031	57,81129784	65,5936423	9
ENSGALG00000003763		30,39802539	26,39791988	26,01635594	25,84006884	9
ENSGALG00000003782		67,52962601	48,8949534	48,05084689	44,1293765	9
ENSGALG00000003837	ADCY7	11,25806886	7,141289587	6,436046755	7,162858002	9
ENSGALG00000003930	APBA2	56,79764343	48,00506316	44,99572286	37,56595504	9
ENSGALG00000003987	TRAF4	24,93184967	18,42870213	17,6207474	15,76983074	9
ENSGALG00000004028	MATN4	4,142818748	1,759184805	2,092418693	1,787010196	9
ENSGALG00000004256		117,1187154	94,6556023	78,78379073	61,2475351	9
ENSGALG00000004331		78,43473809	47,01093113	44,17985984	35,90771284	9
ENSGALG00000004338		184,3647772	126,1585532	128,6882618	115,000824	9
ENSGALG00000004364	ABR	63,93113798	46,56275273	54,95450035	38,63387769	9
ENSGALG00000004400		22,88103183	17,29827581	15,39979111	11,65059965	9
ENSGALG00000004464		29,2120941	22,14419799	23,17263832	21,18993522	9
ENSGALG00000004516		159,5821658	133,8086625	134,3461022	133,3681191	9
ENSGALG00000004530	MMD2	36,22728323	25,45444714	16,61274281	19,35254048	9
ENSGALG00000004621	PREX1	57,87358886	41,24143534	46,0542225	33,12573231	9
ENSGALG00000004790		654,3246722	352,1800754	269,3322898	224,3252022	9
ENSGALG00000004954		335,5094457	269,4578124	262,8551051	262,2329529	9
ENSGALG00000004986	CERCAM	289,2108064	153,4223911	163,1756543	124,3823144	9
ENSGALG00000005214		22,97422669	17,61003498	17,43784342	14,70240257	9
ENSGALG00000005237	FGD3	100,5472145	86,36549191	75,01190805	60,70387368	9
ENSGALG00000005246		90,58806014	51,35115641	62,64847331	38,43466117	9
ENSGALG00000005319	CDH8	5,445262477	4,173315769	3,017728175	1,866804765	9
ENSGALG00000005403	DHRS11	99,94667899	73,04572469	64,1697335	58,53117191	9
ENSGALG00000005405		31,49344217	26,42825252	25,35628462	25,1695081	9
ENSGALG00000005413		17,63752439	7,770628589	5,025165513	4,739852004	9
ENSGALG00000005426	FREM1	4,04595993	2,468827971	2,431482695	2,135553458	9
ENSGALG00000005680		9,640144343	7,265188978	6,687105253	7,850074723	9
ENSGALG00000005718	BHLHE23	7,660886537	4,527062152	3,141076562	4,207532061	9
ENSGALG00000005739	SCD	120,233915	71,49058858	95,28129954	82,94998739	9
ENSGALG00000005774		107,6754727	75,61866044	66,91043187	46,31621048	9
ENSGALG00000005806	KIF24	14,08523354	11,47064874	9,930660996	9,661822978	9
ENSGALG00000005817		40,19436852	33,50907622	33,14220262	32,53433765	9
ENSGALG00000005892		231,8060061	154,3941218	131,8842244	122,7009004	9
ENSGALG00000005903	NRIP3	13,90935106	8,083012884	7,461017706	7,936189903	9
ENSGALG00000005956		2,030478214	0,939634647	0,819765053	0,774947504	9
ENSGALG00000005961	dAK	237,4982121	209,651884	186,032781	188,3048286	9
ENSGALG00000005975		25,36720101	9,858054738	7,549317126	8,015243217	9
ENSGALG00000005989		67,30399299	61,24008237	57,66738697	52,15578407	9
ENSGALG00000006037	MCM2	331,6274576	265,1735327	287,4206462	284,4425533	9
ENSGALG00000006094	PHF6	205,6581455	163,1742698	166,1087468	124,9981669	9
ENSGALG00000006158	ACVR2B	187,5109516	161,5663473	141,2383772	119,6603309	9
ENSGALG00000006210		681,9171879	337,8310196	285,5834543	265,8640383	9
ENSGALG00000006219	PRKAG2	116,4614015	99,89346909	100,664404	99,31929353	9
ENSGALG00000006235		25,72391513	18,24632753	12,9330952	11,31001119	9
ENSGALG00000006267	TPX2	285,3239263	234,4828814	242,4073029	244,9347288	9
ENSGALG00000006409		98,96573337	59,31551837	64,86590911	48,98443263	9
ENSGALG00000006483		66,59212784	47,861138	53,79593876	41,7959226	9
ENSGALG00000006488	RYK	123,9224556	101,6109651	102,7321424	85,2358062	9
ENSGALG00000006555		36,25083342	15,25441221	21,94626456	13,42875792	9
ENSGALG00000006563	SEMA3D	64,15874594	42,35039981	31,42366476	24,29139394	9
ENSGALG00000006623	ATP11C	84,40040171	75,82040472	72,31961566	76,48580465	9
ENSGALG00000006634		312,6569359	230,7046187	244,5534232	217,9314324	9
ENSGALG00000006666	BPIFB2	12,13866269	7,437423024	6,49942857	7,202762558	9
ENSGALG00000006826	KLHL25	110,0451345	65,01099472	69,38138855	48,75260528	9
ENSGALG00000006859		8,765240974	7,31641822	6,12271801	5,389653833	9
ENSGALG00000007245		27,97164142	18,0114389	18,55377799	13,50467657	9
ENSGALG00000007254	ARHGAP12	45,44941843	33,57720882	36,21730402	30,37036253	9
ENSGALG00000007269	GABRA3	13,96411172	10,86879485	9,501500127	8,542584012	9
ENSGALG00000007304		34,23812047	29,61794147	25,721381	25,45594431	9
ENSGALG00000007419	MASP1	1,57539922	0,938984074	0,512908118	0,711741724	9
ENSGALG00000007668	BMP7	76,36611621	53,6951202	61,40584973	54,13905456	9

ENSGALG00000007710		99,30828406	71,94323354	71,27438268	46,25151534	9
ENSGALG00000007781	GAL3ST1	14,13111353	9,3883292	5,772312125	6,986217646	9
ENSGALG00000007892	SPAG6	61,11626497	47,97350969	39,2464153	36,4836125	9
ENSGALG00000007929	DIXDC1	68,58037619	48,0580845	38,09909643	29,97386629	9
ENSGALG00000008174		124,348004	107,8743784	104,2337788	99,6087797	9
ENSGALG00000008215		50,38257469	31,52731813	20,60181027	23,6834746	9
ENSGALG00000008279		577,5801838	394,4296542	272,5352455	249,8305959	9
ENSGALG00000008425		14,69561107	12,47619676	11,11587652	9,119602956	9
ENSGALG00000008525	ABHD12	51,3904353	43,30549983	40,64464425	38,93553863	9
ENSGALG00000008616		156,5243897	126,1365769	136,68892	104,2885426	9
ENSGALG00000008698	MGME1	71,41290823	58,06460253	56,79294211	53,48784783	9
ENSGALG00000008758		124,4228967	82,10905734	98,0079813	61,64666093	9
ENSGALG00000008871		80,84342079	47,41819894	54,93095143	39,863161	9
ENSGALG00000008873	POLE3	321,2902245	262,7556375	240,5475354	218,3913542	9
ENSGALG00000008887		33,84974642	27,03979324	26,04572531	22,59057103	9
ENSGALG00000008896		6,022737193	3,499565077	2,398429886	2,115469582	9
ENSGALG00000008961		138,0057818	67,99395964	89,6981597	52,304204	9
ENSGALG00000009020	JAG1	124,7023905	75,41664337	74,13724728	55,83821036	9
ENSGALG00000009027	ST6GALNAC3	29,42036569	25,27116058	20,59626824	18,22933473	9
ENSGALG00000009207	TRIM2	30,67711925	25,11356027	21,42653595	15,16568337	9
ENSGALG00000009279	SFMBT2	10,91446674	6,537015591	7,691053349	7,838415429	9
ENSGALG00000009474		165,3825058	136,4697521	134,2634588	138,1460328	9
ENSGALG00000009514	NRCAM	42,45191179	20,00427649	23,8798494	14,18976528	9
ENSGALG00000009516		41,86000548	32,23669772	30,81092781	28,62779004	9
ENSGALG00000009619		40,1118097	32,22391492	31,93240207	32,85425266	9
ENSGALG00000009626	THBS1	45,34052685	25,28042099	27,54267112	21,88710977	9
ENSGALG00000009732	PCDH18	41,0860473	31,86811586	23,36024215	21,9012931	9
ENSGALG00000010097	GATB	32,46219309	27,21579359	23,9674918	19,30756619	9
ENSGALG00000010170	INTU	36,14029054	26,89626511	28,52273715	23,97967786	9
ENSGALG00000010312	PLCH1	44,07417619	31,12405937	36,06839209	29,68185375	9
ENSGALG00000010346	TGFB3	93,98664535	76,02551444	69,58015827	79,88767103	9
ENSGALG00000010448	LTBP1	245,7170762	174,8131199	213,6709569	130,4004299	9
ENSGALG00000010598	KCNK10	7,855388034	3,90410236	4,553758606	2,460899286	9
ENSGALG00000010674	DCTD	192,9283112	144,6806116	152,1865547	117,8680635	9
ENSGALG00000010700	RPA3	260,5499153	214,8768156	223,3044233	200,2168515	9
ENSGALG00000010705	ZBTB18	44,10376849	24,51748535	31,58377038	25,0157285	9
ENSGALG00000010775		12,70190269	9,002694914	7,808452185	6,029336459	9
ENSGALG00000010798	DHCR24	54,13550003	46,53369275	42,79643706	47,16973778	9
ENSGALG00000010818	PLPP3	69,29865779	48,32497394	49,3012161	41,06142891	9
ENSGALG00000010825	AGR2	2,225899698	0,437850605	0,260687102	0,445933444	9
ENSGALG00000010829		167,2465489	136,4200703	138,2033727	116,3988713	9
ENSGALG00000010835	DTX4	1195,686573	840,6890347	831,069909	723,909037	9
ENSGALG00000010903		30,51325584	19,6495363	18,42950404	14,34456426	9
ENSGALG00000010960	GALNT3	20,3476324	13,46078214	12,92983377	9,283859309	9
ENSGALG00000010971	CSRNP3	17,1320569	10,94668327	12,37192759	9,463981496	9
ENSGALG00000011109	MAPK10	10,6059035	8,201546462	6,224593472	6,370787412	9
ENSGALG00000011182	DLL1	115,6739078	83,57962434	66,45209234	63,60645287	9
ENSGALG00000011206	KITLG	22,29167397	16,26019056	12,14019114	12,29957305	9
ENSGALG00000011571	AGPAT4	93,67204267	68,94234947	78,24764951	60,3321412	9
ENSGALG00000011823	FAT4	25,5685505	20,27150072	15,35641303	12,56335526	9
ENSGALG00000012017	DAAM1	66,51638573	51,6956635	46,97005806	42,50632291	9
ENSGALG00000012121	RCN1	128,2812544	85,34230795	104,339948	73,50530684	9
ENSGALG00000012123	PAX6	207,3310704	125,6192176	94,53567349	104,5564319	9
ENSGALG00000012124	PELI2	97,01672853	72,38390338	68,07241714	72,99638735	9
ENSGALG00000012130	DNAJC24	87,42750265	67,82813268	67,26018354	54,6134636	9
ENSGALG00000012184		17,53434364	10,51607147	5,571243627	4,897927265	9
ENSGALG00000012216	BMPR1B	19,73470606	17,41660439	14,638607	11,03804069	9
ENSGALG00000012226		10,14170208	6,091780586	5,829669854	4,876167191	9
ENSGALG00000012332		1,75499313	0,334938857	0,049686386	0,405453413	9
ENSGALG00000012361	NIN	38,54121987	30,6965853	28,85722382	29,36252216	9
ENSGALG00000012388	PCMT1	114,9803287	97,79226505	96,7908776	85,73985935	9
ENSGALG00000012395	PLEKHG1	40,97672463	34,45473513	31,88510956	26,43661125	9

ENSGALG00000012440		126,942135	81,54303435	74,69736259	83,67449226	9
ENSGALG00000012595	AGTPBP1	5,843425877	3,87973109	3,244881839	3,714060964	9
ENSGALG00000012618	FANCC	77,15501466	61,95447317	63,86958208	59,5116733	9
ENSGALG00000012620	PTCH1	128,0187265	79,01327975	78,98591949	63,93344831	9
ENSGALG00000012662	NUAK1	65,54806121	48,25369742	54,49515428	35,50923797	9
ENSGALG00000012684	DEK	213,1310822	191,1534846	182,5897587	191,0382858	9
ENSGALG00000012697	SLC41A2	0,058029502	0,037541976	0,025719414	0,034391684	9
ENSGALG00000012703	DTNBP1	65,11808287	60,06352135	53,570554	45,8855677	9
ENSGALG00000012756	PARPBP	8,741901058	7,493838313	6,914878548	5,358083966	9
ENSGALG00000012775	TFAP2A	8,004715347	5,508538021	5,589140758	6,133219794	9
ENSGALG00000012936		43,2525541	33,16536089	32,41777008	26,8771372	9
ENSGALG00000013155		60,96549085	50,10471915	41,405435	39,39397896	9
ENSGALG00000013211	Sial-T2	53,06870305	39,2868121	36,22341479	29,76988665	9
ENSGALG00000013212		352,0737733	218,2231586	304,4999677	177,1428711	9
ENSGALG00000013357		14,5238095	9,676299999	9,468806965	11,33051766	9
ENSGALG00000013672	TSHZ1	47,63040303	40,01248352	37,12051956	40,35485231	9
ENSGALG00000013722	SEMA3B	67,72409615	45,67603936	47,22283227	43,11405926	9
ENSGALG00000013754		99,90845036	74,32231981	73,38471802	63,38796719	9
ENSGALG00000013756		9,551795017	8,012150023	7,152967635	6,59881996	9
ENSGALG00000013782	CDH7	44,73753961	24,83144713	33,13366661	19,36298515	9
ENSGALG00000013900	MAP7	18,05284523	9,552077542	7,566128142	7,59934912	9
ENSGALG00000013961		56,10973329	47,74639425	42,56173827	38,2782928	9
ENSGALG00000014069	TP53I11	462,0420259	303,2892015	318,9636775	276,2729485	9
ENSGALG00000014100		7,763915083	3,510610015	2,846977556	4,067062932	9
ENSGALG00000014218	PHF21B	44,41267673	32,88106133	33,30902396	23,77376394	9
ENSGALG00000014284		25,24429229	20,35052461	20,85799387	23,81657043	9
ENSGALG00000014347		21,79724499	18,61697194	16,21325842	13,48321788	9
ENSGALG00000014485	LDB2	58,65859245	44,03980556	38,92048977	32,99839391	9
ENSGALG00000014508	CD38	34,52009233	22,68968774	20,56010187	19,01534557	9
ENSGALG00000014620		1,956048892	0,782889319	0,862399115	0,580997363	9
ENSGALG00000014646	SYT11	171,4109919	151,5937547	140,9231322	134,8023289	9
ENSGALG00000014672	ARSK	14,70009358	11,78884508	10,76404571	9,602709952	9
ENSGALG00000014680		24,0520188	19,83747893	17,85066188	16,91394554	9
ENSGALG00000014753	CENPK	106,4544342	83,51630756	81,07856229	90,52213786	9
ENSGALG00000014756	PPWD1	129,0762799	102,4153578	107,5831259	108,0635267	9
ENSGALG00000014829		15,02253293	10,43374439	6,545915045	6,828450455	9
ENSGALG00000014897	TYMS	276,8568161	246,1181255	233,2358706	234,7946898	9
ENSGALG00000014903	ITGA2	19,28102901	12,04416315	14,06402156	10,26187687	9
ENSGALG00000014934	HS3ST1	5,540861535	3,210938472	2,425738011	1,67621552	9
ENSGALG00000015053	GLDC	65,20466566	54,00282438	55,14865591	47,938418	9
ENSGALG00000015091	HAUS6	107,8740638	77,47610692	90,80409523	80,42181649	9
ENSGALG00000015103	CCDC171	9,278746716	7,442349244	6,81158066	5,688912777	9
ENSGALG00000015152	BOC	61,37451521	50,47041324	41,88963775	37,19576843	9
ENSGALG00000015241		34,52994734	20,74355677	21,80562309	14,0162192	9
ENSGALG00000015386	RNF170	48,11695973	40,96561413	36,04033923	29,04558532	9
ENSGALG00000015444		17,77233078	14,64411238	13,67274692	12,36829449	9
ENSGALG00000015448		26,99122737	19,26894575	15,38003926	10,64663709	9
ENSGALG00000015476		14,64646726	7,865669198	7,720149158	5,783165827	9
ENSGALG00000015564		7,156970347	5,334313058	5,500962035	4,919475757	9
ENSGALG00000015673		19,31944035	13,00690578	11,37988935	9,833987567	9
ENSGALG00000015685	CFAP99	9,786223813	7,029241391	6,950135982	6,216729289	9
ENSGALG00000015712	SLBP	102,5760884	74,18453744	83,0184383	76,31708087	9
ENSGALG00000015786	REEP1	53,13915006	42,50403724	36,81544162	36,11332154	9
ENSGALG00000015799	ELAVL2	6,037654055	4,325382874	3,767789255	2,819033678	9
ENSGALG00000015847	PRSS35	35,11704576	25,66370192	24,07123267	19,02952064	9
ENSGALG00000016034	ATRN	111,1123874	81,26651019	87,63945569	67,82246842	9
ENSGALG00000016148	C2CD2	39,27360457	32,23641902	29,98843351	28,60296797	9
ENSGALG00000016171		218,2787932	205,7354159	192,6760184	172,0707128	9
ENSGALG00000016200		51,81097754	39,08952357	37,81925937	29,69260203	9
ENSGALG00000016244		34,80907173	29,60541455	24,53665949	19,37430659	9
ENSGALG00000016326		10,59842146	8,398086434	7,852987771	6,850555245	9
ENSGALG00000016391	CNKSR2	26,29502855	16,46476384	16,96231718	11,5736257	9

ENSGALG00000016394		32,19475368	17,39524143	21,9320163	17,49313991	9
ENSGALG00000016417		131,3461383	103,1768005	104,7836855	88,93333367	9
ENSGALG00000016428		98,90427495	50,27843955	39,10462421	33,95468388	9
ENSGALG00000016442	RRM2	593,9920997	471,2620483	469,5176719	463,1466191	9
ENSGALG00000016456	Lpin1	11,86024943	9,523941198	9,270708107	9,810098096	9
ENSGALG00000016459	FAM84A	7,093676028	4,179695873	3,107376993	3,325425099	9
ENSGALG00000016487	LDAH	39,21408017	36,90700532	33,03840175	29,58276228	9
ENSGALG00000016497	UBXN2A	68,16553674	58,34933795	51,49336847	45,51619727	9
ENSGALG00000016575	GPM6B	267,7110691	174,3280247	163,2139649	132,8150489	9
ENSGALG00000016576	OFD1	80,96250322	62,01808879	54,92287394	47,92994163	9
ENSGALG00000016676	MCM3	220,3958455	163,3016763	186,0877349	184,8337011	9
ENSGALG00000016810		34,46474494	22,03432788	16,29129687	17,02120699	9
ENSGALG00000016811		20,87242426	16,56591395	15,39201879	12,9699234	9
ENSGALG00000017021	ATP7B	3,9869324	2,371265345	1,829014636	1,212619235	9
ENSGALG00000017154	ZC3H12C	16,69008154	11,1294025	10,93196073	9,942631937	9
ENSGALG00000017393		14,97893571	10,68802307	10,58952889	9,672886802	9
ENSGALG00000019987	LRP11	101,4977697	84,74657711	74,00932276	62,64430136	9
ENSGALG00000020003	MAP3K4	101,7199951	71,21199104	84,45899897	57,14632163	9
ENSGALG00000020554		2,745534391	1,931406303	1,752178514	1,782013487	9
ENSGALG00000020788		16,08950004	7,37692355	7,655663237	3,861511503	9
ENSGALG00000020813		171,290155	148,1329936	144,4510861	138,3803558	9
ENSGALG00000021031	ACKR3	197,7454394	96,43341863	106,3679352	85,09027757	9
ENSGALG00000021340	CA9	246,544707	203,2768771	190,5602386	192,1909568	9
ENSGALG00000021592		24,90805272	18,51353834	18,06631656	13,3222017	9
ENSGALG00000021593		21,6290003	14,87215611	11,48507743	16,43767163	9
ENSGALG00000021616		660,7892	560,4597192	492,802757	474,9610111	9
ENSGALG00000022603		127,7440137	94,25649465	98,2989799	83,9412793	9
ENSGALG00000023114	TAL2	10,83090997	4,516618379	3,527657045	4,165674735	9
ENSGALG00000023359	BTBD17	12,37917296	9,215387972	6,210062578	6,211710478	9
ENSGALG00000023546		103,3250361	82,98091285	85,32961236	77,35742371	9
ENSGALG00000023647		16,32978445	10,43731524	9,865902217	8,46856396	9
ENSGALG00000023898		25,15757129	20,28376564	21,16857225	21,71796482	9
ENSGALG00000024047	MYCL	130,6449284	91,27348025	100,1887482	78,95088543	9
ENSGALG00000024120		251,9186613	186,3133705	184,633668	156,1919821	9
ENSGALG00000025796	ADI1	151,9056229	128,8288136	130,0669627	115,3487407	9
ENSGALG00000026085		37,55971233	29,79205273	28,08976508	26,0061611	9
ENSGALG00000026179	FAM163B	12,70392319	7,406176596	6,530189162	10,54638186	9
ENSGALG00000026192	ST8SIA4	26,69578621	14,31843914	14,38997567	10,57310427	9
ENSGALG00000026214	LRTM2	5,889632729	3,621162228	2,979514896	2,858887818	9
ENSGALG00000026313	RND3	54,52252904	42,60080306	41,54066922	34,55755523	9
ENSGALG00000026395	TRABD2A	34,96858896	22,03852277	14,4142307	13,7208985	9
ENSGALG00000026452	MURC	68,5514329	42,65798561	57,55999236	40,22180647	9
ENSGALG00000026741		92,52230306	78,97977067	79,76273285	68,87406566	9
ENSGALG00000026757		115,1492891	83,10995002	78,42339249	85,39515762	9
ENSGALG00000026858	BTG2	73,25524576	56,978272	43,18762707	46,83714731	9
ENSGALG00000026981	NHSL1	90,0052068	79,25555686	72,87488074	70,3460249	9
ENSGALG00000027002		335,0560184	189,0165812	223,361849	151,3843853	9
ENSGALG00000027183		19,96308058	12,60167171	12,49826777	10,81410515	9
ENSGALG00000027198	TMEM47	68,18436645	46,36338174	41,15361629	45,81100202	9
ENSGALG00000027240	TSPAN13	60,13229624	43,7309013	38,32508629	35,23013388	9
ENSGALG00000027373		255,2616537	165,0099254	173,1189979	141,526405	9
ENSGALG00000027472	TMEM38B	18,53265539	16,23438527	14,16492945	12,41744795	9
ENSGALG00000027484	C20orf85	5,62155177	3,44560581	2,174506144	2,913151077	9
ENSGALG00000027514		0,528787008	0,166761415	0,080304322	0,124803265	9
ENSGALG00000027633	RASSF9	2,035731325	0,854912883	0,737308999	0,339184915	9
ENSGALG00000027821		23,11507984	14,67663083	10,50775245	14,49954578	9
ENSGALG00000027897	DTX1	44,84876496	37,26651436	33,21350155	34,29440557	9
ENSGALG00000028043		35,35893431	28,05893819	22,40021823	20,30301901	9
ENSGALG00000028140	CNTFR	21,29176694	15,68480074	14,7877547	16,3046985	9
ENSGALG00000028327	TMEM178A	15,25643824	12,99141162	11,17599999	10,775708	9
ENSGALG00000028415		51,81555289	16,42028113	20,4065691	12,36370773	9
ENSGALG00000028536	MPEG1	20,5427871	13,37260379	15,08320974	11,52346016	9

ENSGALG00000028790	DNASE2B	19,09091612	11,85162585	9,263964302	10,09521056	9
ENSGALG00000029004		0,626344243	0,267590302	0,357524634	0,25546319	9
ENSGALG00000029011	SLC35F1	147,2321386	101,3673948	105,6335088	79,54888119	9
ENSGALG00000029057		6,612061759	3,565809227	3,192304914	3,978423806	9
ENSGALG00000029070	FAM181A	140,8743623	93,20761779	88,5842806	63,71077724	9
ENSGALG00000029072	NTN3	23,04277493	13,47398348	14,06728438	13,23933001	9
ENSGALG00000029106		11,86577158	8,120777874	6,029059075	6,275995647	9
ENSGALG00000029140	CRABP2	943,46397	516,5320484	569,9989128	473,9399887	9
ENSGALG00000029151		3,237130342	2,159102861	1,570247908	2,377856659	9
ENSGALG00000000067	SPR	25,19151507	38,09496599	32,36154561	46,01001796	10
ENSGALG00000000243	RDM1	64,16894169	84,57366491	88,19253189	87,07892618	10
ENSGALG00000000505	KCNH6	1,806346197	3,772203834	3,349604234	3,427118714	10
ENSGALG00000000836	RFX5	0,220047592	0,798370098	0,387008719	0,842809634	10
ENSGALG00000000894	APCDD1	12,17953658	20,37298423	18,67259013	21,14941456	10
ENSGALG00000001376	ARID3B	81,77846269	128,5884065	111,6210537	158,6263766	10
ENSGALG00000001664	PTPN9	27,32342552	45,33442697	41,5208593	49,78702526	10
ENSGALG00000002054		4,463691977	10,11551737	8,122956552	9,768543779	10
ENSGALG00000002210	PPIB	615,7965084	812,398235	771,3773995	889,5451731	10
ENSGALG00000002342		76,97404676	104,3309613	95,54319676	118,6337645	10
ENSGALG00000002922	NOTCH2	34,87569574	45,95827585	44,26184738	50,20078048	10
ENSGALG00000002969	MAU2	41,46144033	47,17099845	48,73910042	47,72736011	10
ENSGALG00000003011	TOM1L1	0,671138223	2,313862165	2,230320103	3,177873963	10
ENSGALG00000003019		7,412203933	9,138713853	9,422655346	10,02764078	10
ENSGALG00000003340	GBGT1	1,301060604	4,566529257	2,549542908	4,535141421	10
ENSGALG00000003562	NPTX2	1,276374613	2,747257273	2,562428679	3,583310899	10
ENSGALG00000003640		9,84244551	40,12648844	16,73702806	37,56346879	10
ENSGALG00000003841	DGKK	1,170538651	1,987308984	2,249687426	2,15756725	10
ENSGALG00000004083	GRIA1	6,30072857	18,26990907	15,82341279	17,5097179	10
ENSGALG00000004719	SPTAN1	93,79037656	111,3665527	109,9098282	108,9079703	10
ENSGALG00000004740	TMOD2	7,782204442	14,88555351	10,95510486	12,75531378	10
ENSGALG00000004805		5,914997516	7,271558567	7,670585037	7,762114467	10
ENSGALG00000004909		59,70087412	104,0709532	86,70697475	99,82170433	10
ENSGALG00000005065	PLA2G4A	4,27041438	8,286182082	7,268353555	8,503620832	10
ENSGALG00000005389		0,141856332	3,826903443	1,782989285	2,582117723	10
ENSGALG00000005861		2,00701187	3,862514989	3,827339633	5,164782157	10
ENSGALG00000005906		25,34069165	46,7681257	40,66967147	52,53393838	10
ENSGALG00000006313		7,796058512	11,54622893	10,90957967	13,40926496	10
ENSGALG00000006641		15,64772345	21,61228415	20,72433764	23,81644591	10
ENSGALG00000006659		40,08388889	51,59649186	52,50217807	58,09914445	10
ENSGALG00000006729		14,16368852	150,8222642	64,1838708	77,30692972	10
ENSGALG00000007412	MPZL2	1,426673901	2,603616958	3,116406355	3,436684581	10
ENSGALG00000007543	APCDD1L	1,285779838	10,76373217	10,1632961	15,43303975	10
ENSGALG00000007565	PPP2R2B	4,498606815	16,55729926	7,013796757	13,48424013	10
ENSGALG00000007833		22,46417563	30,2369186	29,28165289	32,02014786	10
ENSGALG00000008009	BCAP29	54,09129251	74,79823611	73,83395516	82,24575148	10
ENSGALG00000008181	MADD	15,2172411	21,6887672	21,73612765	25,35030935	10
ENSGALG00000008999	AFAP1L2	5,746909958	16,02416497	14,60767657	23,10791907	10
ENSGALG00000009155		6,468639196	11,71015107	12,0614327	12,97982335	10
ENSGALG00000009433	BAG3	43,32330391	89,33785815	65,96819578	99,83819404	10
ENSGALG00000009505	PNPLA8	33,57778621	40,98407666	40,30681413	42,47879678	10
ENSGALG00000009570	ATP6V1D	74,62845941	128,6859866	102,1283936	130,2908971	10
ENSGALG00000009718	FAM53B	5,787080902	16,4568785	11,11024098	15,08477821	10
ENSGALG00000009759	SLC16A7	0,371037879	0,893895773	0,86989287	1,026405516	10
ENSGALG00000009783		10,2405462	14,28065595	13,0313556	14,17793631	10
ENSGALG00000009792		10,84341484	21,73021356	16,86750659	17,88482058	10
ENSGALG00000009896	TRERF1	4,808170267	13,5104085	7,397743277	15,27344774	10
ENSGALG00000009972		8,233254639	14,77085833	10,96112463	12,48984776	10
ENSGALG00000009988	ELOVL1	24,54970046	40,25627092	37,95280868	40,63830581	10
ENSGALG00000010164	SMARCA2	31,48210827	48,72221196	46,7844494	57,39705613	10
ENSGALG00000010268		4,921325317	13,01196961	15,00161538	16,21986662	10
ENSGALG00000010664	KIF26B	0,871077545	3,300649183	1,910395118	3,87997435	10
ENSGALG00000010911		1,617219387	3,748364438	2,982354432	4,754746898	10

ENSGALG00000010934		1,8031112	3,709899605	2,91645905	4,653025085	10
ENSGALG00000011016	PGM1	23,44574688	32,53423526	32,62243154	31,85192134	10
ENSGALG00000011286	UBE2E1	62,79994395	81,23871675	90,03965196	96,89006504	10
ENSGALG00000011488	CMTM7	7,277427951	20,1323349	11,74053362	16,23963969	10
ENSGALG00000011690		0,103946776	0,939637581	0,88149688	0,834198832	10
ENSGALG00000011972		21,41005163	26,662867	28,93241264	30,75647977	10
ENSGALG00000012093		12,1904691	23,2396876	16,99874812	22,36987224	10
ENSGALG00000012118	TMEM260	10,53321393	13,22765331	14,21926447	15,08071395	10
ENSGALG00000012211	PDLIM5	16,67664874	23,83760349	27,59486714	28,08174448	10
ENSGALG00000012280		32,6377987	47,09813408	43,51845385	46,57456552	10
ENSGALG00000012304	NFKB1	18,92353309	36,72187679	23,71285028	29,81253681	10
ENSGALG00000012345	UST	25,98919869	34,81550817	36,11724943	34,83622704	10
ENSGALG00000012877	CREB3L2	14,15122188	29,46359767	22,89406436	24,70796898	10
ENSGALG00000013738	OPTN	31,20424125	63,0962501	64,58752569	83,85842847	10
ENSGALG00000013830	PRELID3A	39,10486792	55,02903372	52,71471236	54,50673512	10
ENSGALG00000013902		1,573316302	4,401902139	4,571135381	4,45045502	10
ENSGALG00000014014		0,595599345	2,558484407	0,964881493	2,547986889	10
ENSGALG00000014106	PPFIBP1	48,33798078	79,49747287	78,82503111	87,90269502	10
ENSGALG00000014933	HEXB	13,82875151	27,24707297	21,86614475	28,18919125	10
ENSGALG00000015250		6,860711489	17,44011316	12,3963785	12,88410587	10
ENSGALG00000015424		48,88941571	72,42774461	65,23032901	67,1628436	10
ENSGALG00000015459		19,21668801	24,21055023	25,90194667	25,85794579	10
ENSGALG00000015652	MFSD10	12,11072038	16,15790442	16,48891498	16,37173519	10
ENSGALG00000015677	SUSD1	5,974385986	10,6464791	9,370624852	13,62756519	10
ENSGALG00000015770	APP	380,9874972	492,5151558	458,3835411	482,5133678	10
ENSGALG00000015792		8,931724434	14,36302132	13,26278114	14,78442869	10
ENSGALG00000015958	EIF2AK3	9,629444865	17,72798233	12,0730299	16,39705179	10
ENSGALG00000016050	TTC3	21,38230929	25,43226191	26,64498514	26,08190222	10
ENSGALG00000016069		14,59709436	23,86183631	25,39502668	24,64718079	10
ENSGALG00000016090		2,529020993	11,65505479	5,834035244	6,766647026	10
ENSGALG00000016311		9,237809102	13,39156196	14,74306104	15,77492644	10
ENSGALG00000016341		0,202104856	0,525241112	0,549551156	0,636899768	10
ENSGALG00000017199	MAML2	16,24202136	24,17136146	25,99411058	26,45082516	10
ENSGALG00000017379		1,368912243	3,891543067	3,862137749	5,074133424	10
ENSGALG00000017414	JUP	51,88278313	71,84240343	75,80087103	85,42821	10
ENSGALG00000017652	TTC9	0,468001686	2,538977862	2,85619406	2,815917147	10
ENSGALG00000018634		3,028664762	6,076484682	4,960409416	5,873704945	10
ENSGALG00000019835		5,591831465	7,516668319	8,481902626	8,879886504	10
ENSGALG00000023541	SPHK1	22,91275764	31,05187359	30,31393316	31,95169083	10
ENSGALG00000023806		73,79335109	95,32276617	100,6008133	101,5648268	10
ENSGALG00000023886	ZNF804A	1,685790356	4,57926496	3,192521495	4,573801224	10
ENSGALG00000025780	ITPRIP	5,385578786	9,094153341	10,15330177	10,84817331	10
ENSGALG00000026184		20,45088044	35,45119107	28,32020272	36,40521622	10
ENSGALG00000026694		1,351457644	3,212622506	2,533295638	2,645072591	10
ENSGALG00000026727	SLC30A10	1,391821025	2,828916703	2,502986912	3,564811485	10
ENSGALG00000026822	WDR81	18,46040207	24,87090381	24,50072815	25,59442237	10
ENSGALG00000027764		2,512489522	9,103387834	10,99845138	12,01146352	10
ENSGALG00000027772		0,004892914	0,62961338	0,353233983	0,624609016	10
ENSGALG00000028155		5,782098605	11,47490998	10,05991248	10,8361757	10
ENSGALG00000028354	METRNL	37,35106289	58,93445961	46,00048458	56,99942589	10
ENSGALG00000028582		3,241190702	10,61160058	7,55174664	14,2492626	10
ENSGALG00000000107	TRIM7	0,223879993	3,047486999	1,757372561	5,176952685	11
ENSGALG00000000117		0,040480999	0,220063553	0,353819011	0,526729322	11
ENSGALG00000000464		34,05612158	42,48913859	44,95313416	70,7967403	11
ENSGALG00000000544	RAB44	0,061671392	0,998421135	3,862756333	17,67716506	11
ENSGALG00000000935		5,314625544	7,277338245	8,425064177	13,7096056	11
ENSGALG00000001163	NDEL1	8,521796698	10,78416631	12,97721211	15,40550268	11
ENSGALG00000001189	NCF1	1,08189946	3,436075078	4,646876547	9,23946829	11
ENSGALG00000001281		0,846894805	2,258112365	2,370684136	5,949678488	11
ENSGALG00000001617	CAMKK1	2,604019637	7,289324417	5,664646749	25,44035569	11
ENSGALG00000001728	NACC2	11,80186684	15,10233978	18,75610144	27,14939763	11
ENSGALG00000002221		17,07683677	20,30245478	22,63798635	24,90324994	11

ENSGALG00000002260	CISH	0,663424147	2,139171593	2,487720205	17,431701	11
ENSGALG00000002548	TLN1	20,31627411	22,61612195	25,81496882	28,39885397	11
ENSGALG00000002583	PIK3CD	2,842773013	3,679276554	4,250538255	5,394793108	11
ENSGALG00000002651	ST3GAL2	40,21612852	57,64615583	53,02907343	92,8557766	11
ENSGALG00000003122	ZFAND2A	38,01972748	57,81426075	57,94506614	99,08508814	11
ENSGALG00000003439	PLEKHM2	48,97856485	55,11774842	59,44696304	66,65072501	11
ENSGALG00000003939		0,73347476	1,054393959	2,139119382	3,112879397	11
ENSGALG00000004599		14,05222685	16,58556716	26,26019986	36,29605918	11
ENSGALG00000004626		160,6674727	192,0383383	196,9247106	241,3004908	11
ENSGALG00000004735		18,71576319	24,95209333	27,715552	47,74079099	11
ENSGALG00000005069		0,925868522	2,967476206	3,749577092	6,294077837	11
ENSGALG00000005422	PDLIM1	22,92658872	27,45113237	32,72226862	44,12324064	11
ENSGALG00000006008	HOMER2	9,984368442	15,75188365	20,62165034	40,94770081	11
ENSGALG00000006407		11,54087345	29,36740869	20,30551587	48,42948482	11
ENSGALG00000006614		0,081869495	0,459517562	0,360325202	3,561928827	11
ENSGALG00000006655	MAP3K13	5,192744917	7,000137701	8,021460066	11,36909123	11
ENSGALG00000006947	BDH1	134,1451069	150,8578862	185,9764126	304,1197319	11
ENSGALG00000006958	COL27A1	1,237521755	2,205820699	2,92438058	3,742640145	11
ENSGALG00000006988		0,188436541	0,498050832	0,566069098	1,241061957	11
ENSGALG00000007238		7,877525855	11,00497278	12,15849698	17,65303471	11
ENSGALG00000007253	IL1RAP	5,654414984	5,558750136	15,57106007	34,03095536	11
ENSGALG00000007271		14,84086276	18,48294427	21,14237742	25,377985	11
ENSGALG00000007394		8,540622935	12,18654051	12,89609102	16,54507416	11
ENSGALG00000007415		0,625774296	1,462296833	4,567836451	9,979634759	11
ENSGALG00000007700	CTTN	128,2039185	140,4859116	150,5105378	160,6028241	11
ENSGALG00000008143		54,04131097	43,71598201	70,22117139	96,62123273	11
ENSGALG00000008461		0,10085251	0,358449616	0,976354066	2,087420033	11
ENSGALG00000008855	UGP2	111,7329891	128,4121345	150,4721612	177,2730406	11
ENSGALG00000008860	LONRF3	13,08961567	11,56252738	17,86767603	27,59267882	11
ENSGALG00000008914	NRAP	0,060907983	0,269803827	0,554252687	1,06803981	11
ENSGALG00000009298	TNIK	63,46441582	78,28507302	87,33775935	95,12356324	11
ENSGALG00000009755	LRIG3	8,560302311	9,689196018	13,21297799	16,14258758	11
ENSGALG00000011054		0,288321762	0,262980056	0,523082511	0,94837143	11
ENSGALG00000011435		39,02276939	40,19212106	61,11491938	81,52693241	11
ENSGALG00000011489		14,63826567	23,22066191	21,51808684	52,80909388	11
ENSGALG00000011682	CNOT4	22,61054622	27,70663704	31,04588956	34,4550159	11
ENSGALG00000012425	FERMT2	83,37649219	100,1967554	100,4088724	114,1270352	11
ENSGALG00000012429	BMP4	17,82855398	36,14532957	28,25726137	99,3060206	11
ENSGALG00000012799	RREB1	11,30126957	13,32117937	15,67245479	20,56566068	11
ENSGALG00000012956	TMTC1	3,209890424	8,381486759	5,824287986	16,89837709	11
ENSGALG00000013971	SGK1	1,199458409	4,40482548	2,542307669	10,62932801	11
ENSGALG00000014261		242,5207982	377,9510577	322,2554339	780,0694354	11
ENSGALG00000015351	CBLB	12,07404883	15,15682028	17,17091245	27,17633895	11
ENSGALG00000015764		44,0304829	84,94006302	66,45816223	168,7490346	11
ENSGALG00000015842	TIAM1	20,70041796	28,78174708	32,87000523	38,20472121	11
ENSGALG00000015952		4,032607318	5,861909098	8,43091945	13,8938431	11
ENSGALG00000016143		17,59022168	32,08840457	37,51357006	60,99757206	11
ENSGALG00000016245	BCOR	28,64786295	33,51699852	36,2266639	41,65368829	11
ENSGALG00000016608	ADCY3	2,853041335	6,651742728	4,591714727	10,19414025	11
ENSGALG00000016728	RP2	25,23490471	36,91489398	30,38401635	48,54519915	11
ENSGALG00000016782		0,10442844	0,304036925	0,846006738	3,678282371	11
ENSGALG00000017053	SPG20	11,56761385	17,96862498	16,31923789	24,44057475	11
ENSGALG00000023768		12,58827437	14,79960637	29,53884984	46,46901449	11
ENSGALG00000023876		18,91142705	31,12230827	30,65086759	49,13801227	11
ENSGALG00000023950		16,76930744	21,1204594	24,25813505	28,37848226	11
ENSGALG00000024094	UBAP2L	121,4816481	122,1153283	148,8144652	174,3988709	11
ENSGALG00000024346		7,457246685	8,304577678	12,23979195	18,63106951	11
ENSGALG00000024357		4,028752212	4,219547662	5,072418923	7,277278899	11
ENSGALG00000026998		3,550274189	4,733865265	5,160014923	6,176494911	11
ENSGALG00000027168		0,136502676	0,267739429	0,637942204	1,071922405	11
ENSGALG00000027218		0,155738515	0,513268647	2,446272203	7,015905066	11
ENSGALG00000027275		0,679644543	3,953449831	4,492768067	7,153248591	11

ENSGALG00000028130		1,446421878	5,232635436	4,495461427	10,72481048	11
ENSGALG00000028318	CDKN1A	11,85233268	16,60453562	17,57147394	39,65416547	11
ENSGALG00000028326	HSD11B2	17,52552612	21,01950351	22,5837827	24,73250896	11
ENSGALG00000028478		8,846493717	10,6899465	14,16438457	22,92265049	11
ENSGALG00000028908	HOXA6	0,080342382	0,199969978	0,088329259	0,326037306	11
ENSGALG00000029104	gga-mir-1732-3	0	0	0	0,104480823	11
ENSGALG00000000146	SUPT6H	75,73757331	73,2737215	93,33629554	93,02462799	12
ENSGALG00000000156	BRD2	395,5412624	375,8987072	462,9625348	448,7455005	12
ENSGALG00000000239		0,126231267	0,270426788	1,459421001	1,749971744	12
ENSGALG00000000363		17,72172408	18,08275976	21,91750525	24,64111121	12
ENSGALG00000000452		13,88409826	12,9025315	23,92675951	31,4506522	12
ENSGALG00000000669	ETV3	55,2844349	51,98736662	82,87935161	85,15029012	12
ENSGALG00000001076	LIMK1	27,37079683	33,09902324	41,69368553	48,09803052	12
ENSGALG00000001108		36,7691568	37,71543648	46,04875359	44,15642446	12
ENSGALG00000001160		15,70235161	23,82939143	39,42507425	37,6126867	12
ENSGALG00000001188		26,63368303	16,27228527	87,3108226	78,12265485	12
ENSGALG00000001258	NUMA1	75,21228323	80,47740842	88,36224786	93,51829499	12
ENSGALG00000001434	STOM	1,111021049	1,193655921	3,310272534	3,967409139	12
ENSGALG00000001439	RANBP10	20,85370319	20,3325829	24,978668	24,63196551	12
ENSGALG00000001629		0,1320548	0,348616407	1,058011551	1,157701708	12
ENSGALG00000001786		0,066749272	0,014283645	0,159044053	0,214445459	12
ENSGALG00000002203	FGF-18	1,02113805	2,989854627	7,968073498	11,18804202	12
ENSGALG00000002219	WBP2	14,30140436	15,18900359	20,5204241	18,87317075	12
ENSGALG00000002672		12,23172351	13,31637203	17,11505231	17,08473791	12
ENSGALG00000002710	IPMK	25,59409512	25,81557827	33,58394728	37,18293113	12
ENSGALG00000002892		3,768927028	3,881473842	6,678895746	8,225997381	12
ENSGALG00000002941		31,89980035	29,96014663	38,7163801	38,24721579	12
ENSGALG00000002955	ADAMTSL2	7,228745086	6,663532384	13,21950783	14,13514694	12
ENSGALG00000003329	GORAB	29,21089981	30,59281993	35,78325922	36,27201232	12
ENSGALG00000003669		30,87085295	31,84817216	37,92728777	40,73316328	12
ENSGALG00000003770	ANXA2	34,30091985	36,59000096	49,56068963	57,6372842	12
ENSGALG00000003774	ATP13A2	32,84399915	32,52396046	56,0962193	53,02322702	12
ENSGALG00000003862		14,85029412	13,21434675	20,79018931	19,72952996	12
ENSGALG00000003977	TWF2	111,4566955	113,279942	130,8659209	133,7761085	12
ENSGALG00000004002	LYPLA2	150,7495569	166,7823137	199,4046703	212,3326077	12
ENSGALG00000004189	MAFK	18,5742469	15,59354155	38,4494806	41,1022777	12
ENSGALG00000004209	ELFN1	0,814582164	1,927779755	2,943360603	2,951185081	12
ENSGALG00000004365	WDR66	2,811763915	2,218322715	7,695524749	7,52136835	12
ENSGALG00000004620	LAMC1	67,46199036	66,61106568	80,89251769	77,47594626	12
ENSGALG00000004644		29,84829422	33,04333611	40,59987383	41,34144336	12
ENSGALG00000004853	LRP3	14,18967878	14,79468174	18,33567747	17,50060833	12
ENSGALG00000005027		72,94509595	75,84276563	86,87569122	95,21946926	12
ENSGALG00000005095	SLC25A25	35,12452075	31,73824572	50,94724862	51,41349297	12
ENSGALG00000005176		3,413908368	6,654325261	18,68400718	17,79864417	12
ENSGALG00000005181	SEC24C	112,2572831	109,0976774	135,2911855	130,1713029	12
ENSGALG00000005249	BICD2	41,4406151	47,18645604	56,32728041	59,8048339	12
ENSGALG00000005263	SOX8	9,020400549	10,58055905	34,04858434	38,05051596	12
ENSGALG00000005397	GGNBP2	38,09740839	43,89356976	47,73002156	49,9842953	12
ENSGALG00000005410		30,14761432	40,13307191	57,20488775	54,56007134	12
ENSGALG00000005423	GRHPR	33,87507342	41,46612067	57,59587439	60,8918674	12
ENSGALG00000005475		42,00791977	40,17065565	51,63447746	53,70763465	12
ENSGALG00000005505	PIK3CB	5,618150407	6,581557383	7,805316892	8,557807703	12
ENSGALG00000005624		40,16275662	42,1965956	48,86600313	48,24907824	12
ENSGALG00000005768	ETV5	17,84320142	19,27243279	32,56938156	36,72354266	12
ENSGALG00000006310		2,754882711	5,047741943	6,407070844	6,761563236	12
ENSGALG00000006340		7,323548685	5,786364119	11,16780943	11,8700516	12
ENSGALG00000006372	RBM33	60,01467349	56,67563861	72,27843028	72,37077428	12
ENSGALG00000006447		12,1905467	16,08270401	60,23850836	48,57012952	12
ENSGALG00000006757	XYLT1	21,69948906	21,78358564	28,28419731	28,13276454	12
ENSGALG00000006855	MELTF	7,135006571	6,352915053	18,88027596	17,81802431	12
ENSGALG00000006863		42,12487657	42,61257462	56,69086954	58,62079625	12
ENSGALG00000006919	POF1B	10,72105321	9,803658308	14,95587342	15,84499082	12

ENSGALG00000007016	FAM169B	16,76630825	19,67869242	24,92697293	23,82466955	12
ENSGALG00000007144	SIK3	2,474106812	3,27210838	3,763083294	4,031320651	12
ENSGALG00000007416	CD3E	0,796692068	0,963660773	2,18018488	1,892526906	12
ENSGALG00000007512		62,47306751	60,26951594	76,45241873	83,74261589	12
ENSGALG00000007514		40,05564722	43,21170517	53,37200909	50,85259172	12
ENSGALG00000007530		2,185558313	3,093638992	7,22955912	8,390199431	12
ENSGALG00000007669	EGR1	20,96798972	15,10254827	112,2799795	93,85732019	12
ENSGALG00000008099		38,73018399	38,99872595	49,856415	56,42754101	12
ENSGALG00000008315		48,98731667	49,55807408	61,57033181	59,50346104	12
ENSGALG00000008369	ABCC5	21,72606872	30,01042058	54,12213459	53,90325716	12
ENSGALG00000008403	PHF21A	43,18235274	47,88824436	51,58618832	51,77574719	12
ENSGALG00000008676	RRBP1	23,1998561	23,99502204	31,01580542	29,92130683	12
ENSGALG00000008856		1,031931903	1,608558804	2,634592306	3,16631245	12
ENSGALG00000008883	TCF7L2	20,496599	30,99622973	37,62172759	37,77014196	12
ENSGALG00000009034		0,801126927	0,932946754	1,753738946	1,779517801	12
ENSGALG00000009086	EHD3	146,0272989	157,6078485	205,2381547	195,3390011	12
ENSGALG00000009396		13,79079951	19,66608855	26,51338215	25,68230408	12
ENSGALG00000010030		41,45059966	41,25182098	57,13689599	55,53405584	12
ENSGALG00000010166	VLDLR	4,067429632	6,335447679	9,796757208	10,20517655	12
ENSGALG00000010291	RBPMS	40,68094996	40,8529908	75,23070837	65,91476169	12
ENSGALG00000010385	TJAP1	28,82460773	27,21958624	36,81273204	35,93680702	12
ENSGALG00000010778	CHRM3	2,273839966	2,32517469	6,105235943	5,391486719	12
ENSGALG00000011025		11,49510331	13,44047249	19,03749752	17,51789112	12
ENSGALG00000011137		0,621854178	0,557885914	2,441826917	1,948944133	12
ENSGALG00000011266	PPM1K	3,953648629	4,152677018	6,846909686	7,380787396	12
ENSGALG00000011608	INF2	1,633620915	6,49652447	13,65966714	14,33444624	12
ENSGALG00000011944		29,7277375	25,20155428	47,10159373	48,8566892	12
ENSGALG00000012277		35,33038415	33,48644634	63,08082454	82,91803991	12
ENSGALG00000012380	SPOPL	26,64799127	26,90568857	37,32642684	35,31642033	12
ENSGALG00000012538	NR4A2	0,505244825	0,625081889	1,1713555	1,07035435	12
ENSGALG00000012885	BCL2	12,34404469	14,24953187	16,80990968	17,44414904	12
ENSGALG00000013021		30,98315435	33,14000451	66,60766145	57,42790105	12
ENSGALG00000013342	GBX2	104,3637185	76,23496549	163,838919	164,7512996	12
ENSGALG00000013628	C6orf62	110,0447131	111,9104576	145,7924007	158,3265634	12
ENSGALG00000013757	TMX3	82,04092518	84,73616801	98,56482332	96,04531272	12
ENSGALG00000014084	FGFR1OP2	38,22441741	44,06957863	58,20825246	56,45097665	12
ENSGALG00000014349		8,560237913	9,973650087	16,79967362	17,03527571	12
ENSGALG00000014355	STIM2	27,91184823	26,64561514	35,21479281	40,31897857	12
ENSGALG00000014516	CPEB2	3,813359717	7,524646883	11,31353119	13,35774029	12
ENSGALG00000014598		121,596947	119,4705335	150,3934093	156,4723329	12
ENSGALG00000014687	EPHA1	0,875109773	0,887791079	2,439656862	2,902792716	12
ENSGALG00000014719		53,36926679	56,13568368	75,95786608	70,89585202	12
ENSGALG00000014815		7,514303562	7,928521182	16,27836163	14,27131615	12
ENSGALG00000014854		235,2101919	242,2048178	262,4097502	261,6360768	12
ENSGALG00000015025	VANGL1	14,75235588	14,95356303	18,5151432	19,32445822	12
ENSGALG00000015036	RIC1	24,6564351	26,54672688	34,77569889	32,31957583	12
ENSGALG00000015943		22,32640939	23,59775922	28,84956292	31,67339581	12
ENSGALG00000016086	EGR4	0,312754609	0,349739454	3,626256956	2,868130472	12
ENSGALG00000016105	DYSF	11,86244012	10,67649701	17,62292607	18,03089	12
ENSGALG00000016283	BEND6	8,65472961	10,21102149	12,41413372	13,84260597	12
ENSGALG00000016348	SAT1	65,846725	146,5356664	203,5314021	222,2269098	12
ENSGALG00000016457	TRIB2	147,3950287	132,4539352	194,1354664	187,1424656	12
ENSGALG00000016500	FKBP12.6	12,53712907	24,62077173	47,98704792	47,94808325	12
ENSGALG00000016508		27,46728733	30,92244173	37,55597573	37,58110828	12
ENSGALG00000017117		0,247769645	0,503287821	1,181898236	1,186818356	12
ENSGALG00000020719		0,7762351	0,748726958	3,053419651	2,604518111	12
ENSGALG00000021041		48,79130614	47,81392988	60,79348483	57,94430863	12
ENSGALG00000023347	PTCHD3	1,787758741	2,844053111	5,289343591	6,207946544	12
ENSGALG00000023468	VPS9D1	20,7533336	23,64061043	26,5205822	26,21200221	12
ENSGALG00000023648	PPP3R1	108,4834821	119,5709887	131,3437155	137,8492022	12
ENSGALG00000025887	ATF3	6,584528653	6,144744457	11,98779346	16,71462268	12
ENSGALG00000025911		7,173430124	8,328187345	12,41146067	14,53810568	12

ENSGALG00000026077	ALDOC	1287,720681	1303,453672	1587,752058	1585,907858	12
ENSGALG00000026154	CARD19	2,519303597	5,718212359	10,62772597	11,77837126	12
ENSGALG00000026231	ARID3A	55,59951305	68,67400855	79,54534838	84,31937056	12
ENSGALG00000026237		197,6339803	214,683724	292,3753153	315,5343838	12
ENSGALG00000026263	RGS8	0,38280482	0,973247805	10,59393568	8,731063711	12
ENSGALG00000026355		2,797729238	3,795263667	5,576978031	6,019303511	12
ENSGALG00000026534		0,434765116	0,435780888	1,090824905	1,486910738	12
ENSGALG00000026850		19,3448547	23,33560488	26,08136183	27,49169005	12
ENSGALG00000026989	HOXA7	1,842634302	1,750162784	3,503069673	3,914427	12
ENSGALG00000027208		33,6710471	32,97550046	43,12346055	42,66001317	12
ENSGALG00000027331		1,065988859	1,550790838	11,17131653	17,68458576	12
ENSGALG00000027352		283,8386064	243,9179572	481,0308996	471,5712627	12
ENSGALG00000027365	HIVEP3	1,475692748	1,810205476	5,644935787	4,967885552	12
ENSGALG00000027554		0,179438786	0,053342062	1,868827591	2,239675166	12
ENSGALG00000027684		17,74702399	21,59636073	35,22185222	45,64200281	12
ENSGALG00000027761	TNFAIP8	5,255419926	6,047134406	7,671477467	7,823425868	12
ENSGALG00000027805		3,22591528	3,316534643	8,789132761	9,027754662	12
ENSGALG00000027925		2,246110271	1,656033894	2,090585784	2,40260606	12
ENSGALG00000028095		116,6593576	116,1033043	153,7263599	150,47256	12
ENSGALG00000028128		48,54899189	31,62799235	69,21356455	93,53834461	12
ENSGALG00000028203	SPRED1	21,61093344	21,70393773	28,89856099	28,51271586	12
ENSGALG00000028726		128,7914409	85,78500371	167,5124021	189,4637389	12
ENSGALG00000028749		2923,755457	3158,474332	3440,767081	3520,806683	12
ENSGALG00000028759	LYPD6B	3,251758824	4,924290781	7,287330896	9,009994746	12
ENSGALG00000028785		0,606392138	1,210251308	2,188601903	2,472445717	12
ENSGALG00000029090		169,0725833	156,4931705	386,9724027	460,4292989	12
